# DNA-encoded chemical libraries yield non-covalent and non-peptidic SARS-CoV-2 main protease inhibitors

**DOI:** 10.1038/s42004-023-00961-y

**Published:** 2023-08-04

**Authors:** Ravikumar Jimmidi, Srinivas Chamakuri, Shuo Lu, Melek Nihan Ucisik, Peng-Jen Chen, Kurt M. Bohren, Seyed Arad Moghadasi, Leroy Versteeg, Christina Nnabuife, Jian-Yuan Li, Xuan Qin, Ying-Chu Chen, John C. Faver, Pranavanand Nyshadham, Kiran L. Sharma, Banumathi Sankaran, Allison Judge, Zhifeng Yu, Feng Li, Jeroen Pollet, Reuben S. Harris, Martin M. Matzuk, Timothy Palzkill, Damian W. Young

**Affiliations:** 1 Center for Drug Discovery, Department of Pathology & Immunology, Baylor College of Medicine, Houston, Texas 77030 USA; 2 Verna and Marrs McLean Department of Biochemistry and Molecular Pharmacology, Baylor College of Medicine, Houston, Texas 77030 USA; 3 Department of Biochemistry, Molecular Biology, and Biophysics, University of Minnesota–Twin Cities, Minneapolis, Minnesota 55455 USA; 4 Department of Pediatrics, National School of Tropical Medicine, Baylor College of Medicine, Houston, Texas 77030 USA; 5 Center for Vaccine Development, Texas Children’s Hospital, 1102 Bates Street, Houston, Texas 77030 USA; 6 Department of Molecular Biophysics and Integrated Bioimaging, Berkeley Center for Structural Biology, Lawrence Berkeley National Laboratory, Berkeley, California 94720 USA; 7 Department of Biochemistry and Structural Biology, University of Texas Health San Antonio, San Antonio, Texas 78229 USA; 8 Howard Hughes Medical Institute, University of Texas Health San Antonio, San Antonio, Texas 78229 USA

**Keywords:** Drug discovery and development, Drug discovery and development, Chemical libraries, Screening

## Abstract

The development of SARS-CoV-2 main protease (M^pro^) inhibitors for the treatment of COVID-19 has mostly benefitted from X-ray structures and preexisting knowledge of inhibitors; however, an efficient method to generate M^pro^ inhibitors, which circumvents such information would be advantageous. As an alternative approach, we show here that DNA-encoded chemistry technology (DEC-Tec) can be used to discover inhibitors of M^pro^. An affinity selection of a 4-billion-membered DNA-encoded chemical library (DECL) using M^pro^ as bait produces novel non-covalent and non-peptide-based small molecule inhibitors of M^pro^ with low nanomolar *K*_i_ values. Furthermore, these compounds demonstrate efficacy against mutant forms of M^pro^ that have shown resistance to the standard-of-care drug nirmatrelvir. Overall, this work demonstrates that DEC-Tec can efficiently generate novel and potent inhibitors without preliminary chemical or structural information.

## Abstract (web summary)

Conventional structure-based design of M^pro^ inhibitors of SARS-CoV-2 often starts from the structural information of M^pro^ and their binders; however, the continual rise of resistant strains requires innovative routes to discover new inhibitors. Here, the authors develop a DNA-encoded chemical library screening to produce non-covalent, non-peptidic small molecule inhibitors for SARS-CoV-2 M^pro^ independently of preliminary knowledge regarding suitable starting points.

## Introduction

Worldwide, the COVID-19 pandemic has claimed nearly 7 million lives. Fortunately, the rapid emergence of the pandemic was met with breakneck scientific efforts to develop first-in-class mRNA vaccines for SARS-CoV-2, the viral agent responsible for COVID-19. These vaccines have spared countless human lives; however, they have also illuminated specific challenges associated with their use which include their high cost of development, equitable global distribution, and public skepticism concerning their safety. Additionally, rapid virus evolution rendered first-generation vaccines ineffective and necessitated the development of variant-specific booster shots, which may require periodic redevelopment analogous to seasonal influenza vaccines. Small molecule drugs offer some advantages in these areas and are deemed as a necessary complement in the COVID-19 treatment arsenal. Unfortunately, de novo small molecule drug development is a slow process and typically requires 3–5 years to reach a clinical candidate^1,2^. This protracted timeline is ill-suited for the fast onset of infectious human pathogens, and strategies for the more rapid development of antiviral agents are critically needed.

The SARS-CoV-2 main protease (M^pro^) is a cysteine protease that induces scission at 11 sites within polyproteins ppla and pplab, and its inhibition blocks the liberation and maturation of proteins required for viral propagation^3,4^. Viral proteases are a validated class of drug targets whose successes date historically back to the approval of HIV protease inhibitors^5^. Fortuitously, efforts within the pharmaceutical industry to develop M^pro^ inhibitors of SARS-CoV of 2002 had been made. While the fast decline of SARS-CoV infection did not warrant the continued development of clinical candidates, certain molecular scaffolds proved to be opportunistic starting points toward tackling SARS-CoV-2 M^pro^, which shares 96% sequence homology. X-ray and in silico approaches in concert with medicinal chemistry led to the efficient delivery of potent and drug-like SARS-CoV-2 M^pro^ compounds in rapid fashion^6,7^. Pfizer’s M^pro^ inhibitor nirmatrelvir was developed from this opportunistic path, and its clinical candidacy was announced in an unprecedented 16 months after the first reported case of COVID-19. Currently, nirmatrelvir is having a major societal impact by diminishing the morbidity and mortality associated with SARS-CoV-2 infection. Unfortunately, this impact may be limited given that M^pro^ mutations causing nirmatrelvir resistance have already been observed in the clinic, underscoring the likely continual need to develop new inhibitors to keep pace with evolving viral strains^8–13^.

Nirmatrelvir’s record-breaking pace of development was largely based on extensive preexisting knowledge surrounding known small molecule inhibitors of M^pro^. The development of protease inhibitors has benefitted from generating peptidomimetics based on knowledge of the sequences of native substrates of proteases. Ensitrelvir, a non-peptidic M^pro^ inhibitor, was recently approved in Japan, demonstrating that expanded structural diversity is warranted^14,15^. Furthermore, the catalytic nucleophilic residues within the active site of proteases have routinely been exploited by including covalent warheads within the inhibitors to capture them. These rational design elements have resulted in scores of protease inhibitor drugs^6,7,16^, including nirmatrelvir. It would be prudent, however, to validate an alternative approach to antiviral drugs where little information about inhibitors to the target is known since a scenario of this type is likely to characterize a future pandemic. DNA-encoded library technology (DEC-Tec) is a platform that can enable de novo small molecule inhibitor development rapidly, economically, and agnostic of detailed structural information related to the target protein or known binders. DEC-Tec relies on the use of DNA-encoded chemical libraries (DECLs), which are typically comprised of billions of small molecules covalently attached to a unique DNA sequence encoding their structures. Using an affinity-based selection procedure, a soluble epitope-tagged (i.e., 6X-His) target protein is incubated with the entire multi-billion-membered DECL and then removed from the solution along with any library molecules that bind to the target. PCR amplification of the DNA tags, followed by next-generation sequencing, leads to the structural identity of the binders. Exemplary target binders inferred from the sequence analysis are then synthesized without the DNA barcode for activity testing. The DEC-Tec platform allows for the efficient sampling of more than 10^9^ different molecules in a single test tube, which is over 1000 times more compounds than a traditional automated high-throughput screening (HTS) campaign. Since a binding-based selection is employed, DEC-Tec alleviates the need to develop a high-throughput functional screen. Moreover, the selection assay itself requires only sub-milligram quantities of the target protein. Thus, DEC-Tec offers the advantage of screening substantially larger compound libraries in a faster and more practical format which are clear advantages for its application to important antiviral targets. We previously reported the use of DEC-Tec to identify a covalent inhibitor of SARS-CoV-2 M^pro^ (ORF1ab residues 3264 to 3569, GenBank code: MN908947.3)^17^. Notably, this screen also led to the identification of more structurally unique non-peptide and non-covalent M^pro^ inhibitors, which we describe herein. These efforts corroborate DEC-Tec as a viable approach for delivering non-traditional types of inhibitors to important antiviral targets in an efficient manner and separate from knowledge of preexisting inhibitors.

## Results and discussion

**Fig. 1:**
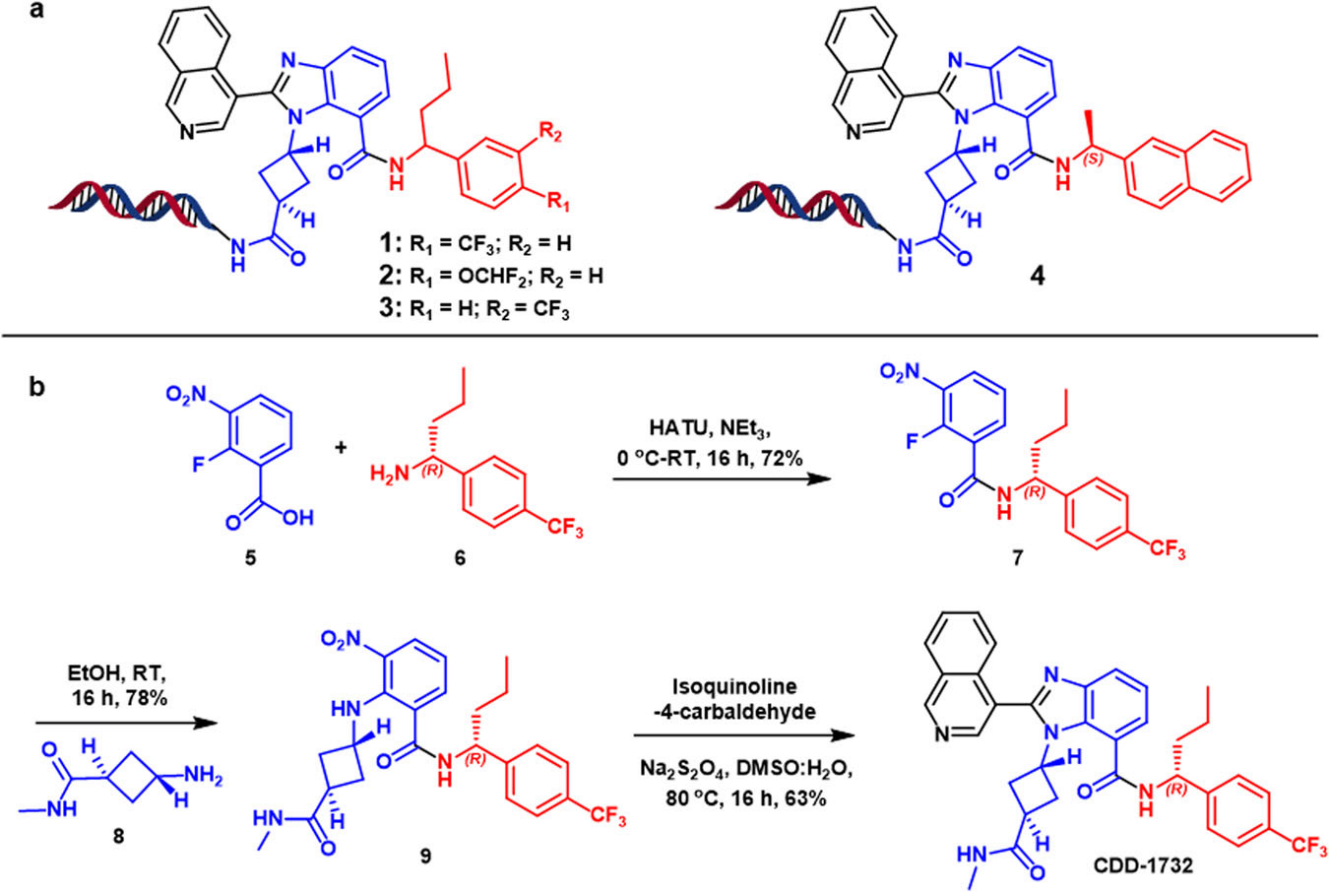
DNA-encoded chemical library selections of SARS-CoV-2 M^pro^. **a** Hit compounds enriched from selections. **b** Off-DNA synthesis of CDD-1732.

The major prerequisite for a DECL selection is a well-folded epitope-tagged target protein. Details about a protein’s three-dimensional structure or a high-throughput assay to evaluate large numbers of compounds are not required, facilitating rapid entry to the selection. Our studies thus began with the expression of a SUMO-M^pro^-His-tag fusion protein as described^17^. The SUMO-M^pro^ fusion junction contains an endogenous M^pro^ cleavage site (SAVLQ ↓ SGFRK) to create an authentic M^pro^ N-terminal sequence while retaining the C-terminal His-tag. The purified target was submitted to an affinity selection with the Baylor College of Medicine DECL repository consisting of 55 individual libraries totaling 4 billion DNA-barcoded compounds^17–23^. Sequencing and informatic analysis^24^ revealed that the selection enriched for compounds comprised of several structural classes, including a covalent inhibitor that we previously reported^17^. Covalent inhibitors are well-known mechanism-based inhibitors of protease enzymes, and it was gratifying that the DECL selection recapitulated binders bearing this structural feature. However, to be applicable to a broader swath of pathogenic targets, it would be desirable if DECL selections could also identify active structures beyond known chemotypes. To these ends, we were intrigued that among the top enriched structures were those arising from a 3-cycle library (qDOS18_2) represented by compound **1** (Fig. 1a). Interestingly, **1** is a racemic compound that belongs to the benzimidazole family and contains no discernable covalent warhead. Moreover, **1** is not a linear peptide scaffold, a characteristic structural feature of many protease inhibitors, such as nirmatrelvir, that are designed to mimic the enzyme’s natural substrates. Therefore, **1**, discovered directly out of the DECL selection, represented a potential novel M^pro^ inhibitory chemotype.

Within the DECL workflow, the next step after selection involves validating the highly enriched compounds by off-DNA synthesis and activity testing. The structures encoded from the qDOS18_2 library, including **1** and closely related structures, are characterized by a benzimidazole core scaffold bearing substitutions at the N1, C2, and C7 positions. Notably, the C7 amide substituent contained a benzylic chiral center which was incorporated as a racemic mixture in the library. Realizing the possibility for differential binding of the enantiomers to M^pro^, we synthesized both antipodes of **1** employing a 3-step synthesis from commercially available starting materials (Fig. 1b, shown for **CDD-1732**, Supplementary Scheme 2). The synthetic route was initiated with 2-fluoro-3-nitrobenzoic acid **5**, which was subjected to an amidation reaction with chiral amine **6** to yield amide intermediate **7**. Next, we performed an S_N_Ar reaction with primary amine **8** to obtain the penultimate intermediate **9**. Finally, the final benzimidazole **CDD-1732** was delivered in a one-pot dithionate-mediated nitro reduction and condensation with isoquinoline-4-carbaldehyde.

To interrogate the M^pro^ inhibitory activity of the synthesized compounds, we measured the proteolytic cleavage of a fluorescent peptide reporter by M^pro^ (Supplementary Table 1). **CDD-1732**, having an *R*-configuration of the chiral center, displayed moderate inhibition with a *K*_i_ of 657 nM. In contrast, it was discovered that **CDD-1733**, harboring the *S*-configuration, gave a *K*_i_ of 12 nM. This result underscores the importance of asymmetric recognition of chiral compounds by proteins. It further highlights that if compounds are screened as racemic mixtures, efficient access to either antipode is advantageous for off-DNA synthesis. Additional synthesis of enriched compounds revealed some structure-activity relationships (SAR) compared to **CDD-1733**. Placement of the trifluoromethyl group in the meta position (**CDD-1795**) decreased activity by approximately 2-fold (*K*_i_ of 29 nM). However, the conversion of the para-trifluoromethyl group to a para-difluoromethyoxy group (**CDD-1780**) yielded a similar activity *K*_i_ of 14 nM. Finally, we synthesized **CDD-1819**, in which a methyl group replaced the larger propyl group at the benzylic position and a naphthyl group replaced the substituted benzyl group. These tandem substitutions resulted in *K*_i_ of 5 nM. Because these compounds were decoded from the affinity selection, these studies illuminate that DECLs can provide high-affinity inhibitors to important viral targets directly. Moreover, the non-peptide and non-covalent nature of the benzimidazole compounds discovered by this screen further highlights that DECLs can provide relevant inhibitors apart from preexisting knowledge, which would be valuable in a pandemic setting.

After successfully validating the structures from the selection, we synthesized a limited number of analogs toward the goal of generating a modicum of structure-activity relationships (SAR). Because the DECL selection process often leads to high-affinity binders directly, extensive medicinal chemistry to explore SAR is generally not required. Nonetheless, even when potent structures are identified, it is useful to understand which structural features of the compounds are critical to the binding event. **CDD-1733** and **CDD-1819** were chosen as parent compounds based on their potency, and our efforts were directed toward determining the importance of specific structural features to their activity (a complete list of analogs is shown in Supplementary Figs. 1 and 2, Supplementary Data 1). We resorted to using the same modular synthesis of the benzimidazole scaffold (Fig. 1b) that was used for the construction of the DECL (Supplementary Scheme 1). This synthesis is ideal for SAR investigation since it enabled substitutions at each position of the benzimidazole simply by incorporating a building block containing a particular structural feature of interest at the appropriate step in the synthesis. Initially, we focused on the N1-amidocyclobutane substituent of the benzimidazole scaffold. Removal of the methylamide substituent on the cyclobutane ring (**CDD-1806**), which mimicked the DNA-attachment site, resulted in a more than 14-fold (*K*_i_ = 165 nM) diminution of activity. When the N1 substituent was changed to an isopropyl group in **CDD-1804**, M^pro^ inhibition was completely abolished, but substitution of the linear *N*-methyl-butylamide in **CDD-1830** gave a *K*_i_ of 6 nM. Taken together, these results indicate that a 4-carbon chain terminating in an amide substituent is optimal at the N1 position of the benzimidazole.

**Fig. 2:**
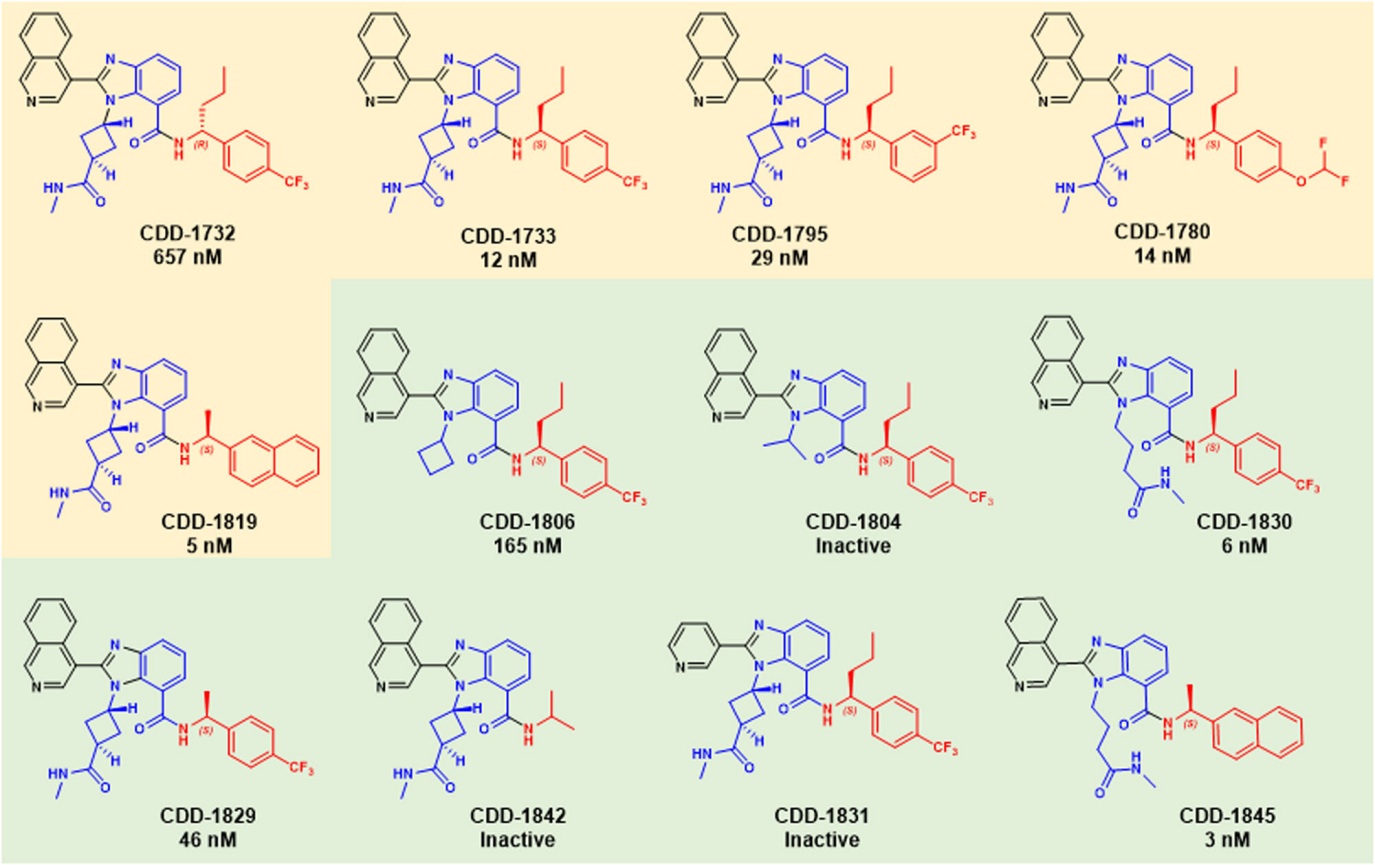
SARS-CoV-2 M^pro^ hits, analogs synthesized off-DNA. Numbers indicate *K*_i_ values determined as described in methods. Inactive: compounds that inhibited M^pro^ activity by less than 90% with 25 μM. Yellow background; M^pro^ inhibitors that are enriched in DEL selections. Green background; M^pro^ inhibitors that are analogs synthesized based on DEL hits.

We next focused on the C7 position of the benzimidazole scaffold. We generated **CDD-1829** to determine whether the smaller chiral methyl group in **CDD-1819** would yield similar activity in the context of the para-trifluoromethylphenyl group; however, this resulted in a diminished *K*_i_ of 46 nM (Fig. 2). To examine the contribution of an arene in general, we generated the *N*-isopropyl amide **CDD-1842**, which is completely inactive. We next turned to the C7 position of the benzimidazole, which was occupied by an isoquinoline group in the parent compound **CDD-1733**. Replacing the isoquinoline for a C3 substituted pyridine (**CDD-1831**) resulted in total loss of activity, suggesting that the larger isoquinoline, as opposed to the pyridine, is forced into an orientation where one atropisomer allows the nitrogen to more optimally participate in hydrogen bonding to H163^25–27^. A similar group of analogs based on **CDD-1819** were also synthesized (Supplementary Fig. 1). Among these, **CDD-1845**, which contains the linear N1-butylamide in place of the N1 cyclobutylamide group of **CDD-1819**, gave the most potent M^pro^ inhibition (*K*_i_ = 3 nM).

These SAR studies provide several noteworthy conclusions. First, the modularity of the DECL synthesis enabled rapid and comprehensive analog synthesis for compound optimization and SAR studies. Synthetic practicality is an important consideration for the efficiency of generating a lead compound. Second, among all the analogs synthesized, two of the three most potent M^pro^ inhibitors (**CDD-1733** and **CDD-1819**) came directly from the DECL synthesis. This underscores that DECLs can sample enough compounds to afford nearly optimal compounds in terms of potency. The latter is important because it diminishes the need for extensive medicinal chemistry around potency allowing chemical optimization to quickly center on other pharmacologically important properties. Synthetic practicality and the reduced need for medicinal chemistry focused on potency are clear advantages to the application of DEC-Tec to targets of viruses that pose an imminent threat to human infection.

**Fig. 3:**
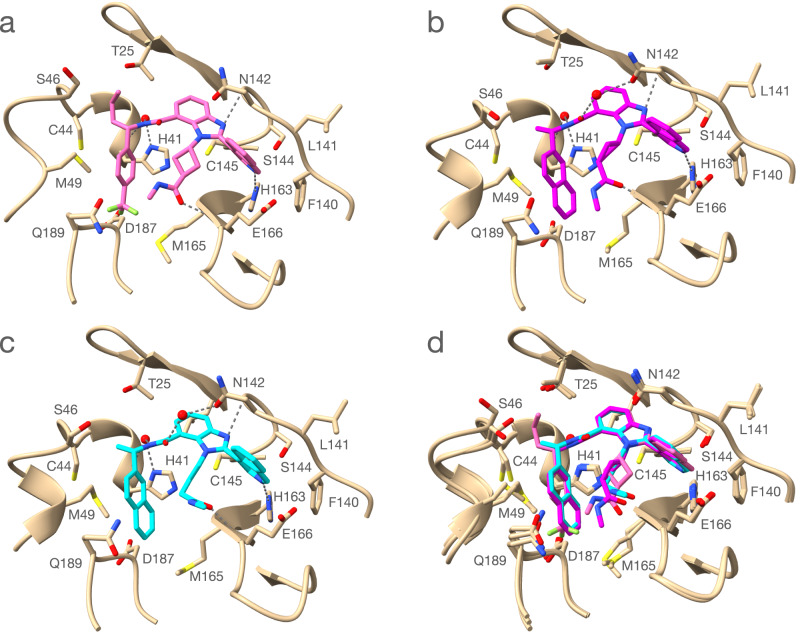
Crystal structure of M^pro^ in complex with CDD-1733 (PDB: 7URB), CDD-1819 (PDB: 7US4) and CDD-1845 (PDB: 7UR9). **a** Structure of M^pro^ (Tan) with CDD-1733 (pink). Carbon atoms of the inhibitor are pink, nitrogen atoms are blue and oxygen atoms are red. The M^pro^ amino acid residues involved in CDD-1733 binding are shown as stick models and labeled. Hydrogen bonds are indicated with dashed gray lines. A water molecule (red sphere) involved in a hydrogen bond from CDD-1733 bridging to the carbonyl oxygen of His41 is shown. **b** Structure of M^pro^ (Tan) with CDD-1819 (magenta). Carbon atoms of the inhibitor are magenta, nitrogen atoms are blue and oxygen atoms are red. The M^pro^ amino acid residues involved in CDD-1819 binding are shown as stick models and labeled. Two water molecules involved in hydrogen bond (colored red) from bridging hydrogen bonds to His41 O and the side chain O of Asn142. **c** Structure of M^pro^ (Tan) with CDD-1845 (light blue). Carbon atoms of the inhibitor are light blue, nitrogen atoms are blue and oxygen atoms are red. The M^pro^ amino acid residues involved in CDD-1845 binding are shown as stick models and labeled. Two water molecules involved in hydrogen bond (colored red) from bridging hydrogen bonds to His41 O and the side chain O of Asn142. **d** Alignment of M^pro^ structures with bound CDD-1733, CDD-1819, and CDD-1845. Hydrogen bonds are omitted for clarity.

As the DECL selection yielded novel M^pro^ inhibitors of a non-covalent and non-peptide nature, we were curious to investigate the structural basis for their activity. To examine the interactions made by these small molecules, we obtained the X-ray crystal structure of the M^pro^ in a complex with potent inhibitors. We were able to produce small molecule-bound M^pro^ co-crystals of our potent inhibitors **CDD-1733**, **CDD-1819**, and **CDD-1845** (Fig. 3a–c). The 2.14 Å resolution structure from space group C121 has a single monomer in the asymmetric unit, and the biological dimer is formed by the monomer and the symmetry-related monomer across the crystallographic two-fold axis. The structure shows **CDD-1733** within the active site where enzyme binding is driven by direct and water-mediated hydrogen bonds and the hydrophobic effect (Fig. 3). The active site of M^pro^ contains four subsites (S1’, S1, S2, S3) that accommodate the amino acids of the peptide substrate (P1’, P1, P2, and P3). The M^pro^ enzyme has a stringent requirement for glutamine in the P1 position, which occupies the S1 subsite. The isoquinoline unit of **CDD-1733** occupies the S1 pocket where the isoquinoline nitrogen makes a hydrogen bond to the NE2 of His163, analogous to the hydrogen bonding of the substrate P1 glutamine (Fig. 3). Hydrophobic effects lead to contacts between the isoquinoline unit and other S1 residues including Phe140, Leu141, and Asn142. SAR results are consistent with the importance of the interactions of the isoquinoline group with the enzyme in that compounds containing modifications of the group, including **CDD-1831**, **CDD-1846**, **CDD-1884**, **CDD-1924**, and **CDD-1926**, are inactive or exhibit greatly decreased potency (Fig. 2, Supplementary Fig. 1, and Supplementary Table 1). It is noteworthy that the isoquinoline group was found to bind similarly in M^pro^ inhibitors discovered from a 235-million compound virtual screen^25^. The five-membered ring of benzimidazole is directly above the catalytic Cys145 and the N3 hydrogen bonds to the main chain *N* of Gly143. The six-membered ring of the hydrophobic benzimidazole extends into the S1’ subsite, where it makes contact with Thr25 and Leu27. The cyclobutane methylamide, which represents the DNA-attachment point in library synthesis, extends toward the S3 and S4 subsites and the C3 carbonyl oxygen hydrogen bonds with the main chain N of Glu166. These findings are consistent with the SAR results wherein truncation of the C3 carbonyl in **CDD-1804** and **CDD-1806** greatly reduces potency (Fig. 2). In addition, the exposed position of the methyl group is consistent with the DNA attachment not causing steric constraints for binding. The tri-fluorophenyl occupies the deep hydrophobic S2 subsite on the enzyme. The chiral n-propyl substitution extends into the S1’ subsite, where it makes contact with the main chain of Thr45 and the side chain of Met49.

We also determined structures of M^pro^ in complex with **CDD-1819** and **CDD-1845** (Fig. 3, Supplementary Data 2). Of note, the unchanged benzimidazole ring is in the identical position in the S1 subsite for all three compounds. Both **CDD-1819** and **CDD-1845** contain a truncation of the n-propyl chain to a methyl group that resides in the S1’ pocket. The most significant change is the replacement of the tri-fluorophenyl with naphthalene in both **CDD-1819** and **CDD-1845**. The naphthalene group more fully occupies the S2 subsite, where increased hydrophobicity is consistent with the increased potency of these compounds compared to CDD-1733. We previously reported the discovery of a covalent inhibitor (**CDD-1713**) from this DEC-Tec approach^17^. While the isoquinoline ring in **CDD-1733**, **CDD-1819**, and **CDD-1845** engages H163 of M^pro^ in a hydrogen bond similar to the indazole ring of the covalent inhibitor **CDD-1713**, no other interactions are common between the two series of compounds (Supplementary Fig. 3). This underscores that DEC-Tec can yield compounds having distinct modes of binding which may lead to important functional differences.

To confirm the potential off-target effect of our M^pro^ inhibitors **CDD-1733**, **CDD-1819**, and **CDD-1845** with other human proteases, we tested against four important proteases such as thrombin (a serine protease), cathepsin B (a cysteine protease like M^pro^), renin (an aspartic protease), and matrix metallopeptidase-1 (MMP-1). None of these inhibitors blocked the enzymatic activity (Supplementary Fig. 4). Thus, the compounds do not have obvious off-target effects toward other protease classes. We also evaluated the cytotoxicity and cell uptake of the compounds using HepG2 cells, all three inhibitors displayed no obvious cytotoxicity and good permeability (Supplementary Figs. 5 and 6). Further, these compounds are relatively stable in human and mouse plasma: after 2 h incubation, 80% of the compounds remained in plasma (Supplementary Fig. 7). Next, we evaluated the in vivo pharmacokinetic properties of **CDD-1733**, **CDD-1819**, and **CDD-1845** in mice, which yielded 2.1 ± 1.4, 1.2 ± 0.1, 1.1 ± 0.3 h half-life (T_1/2_), respectively (Supplementary Fig. 8).

**Fig. 4:**
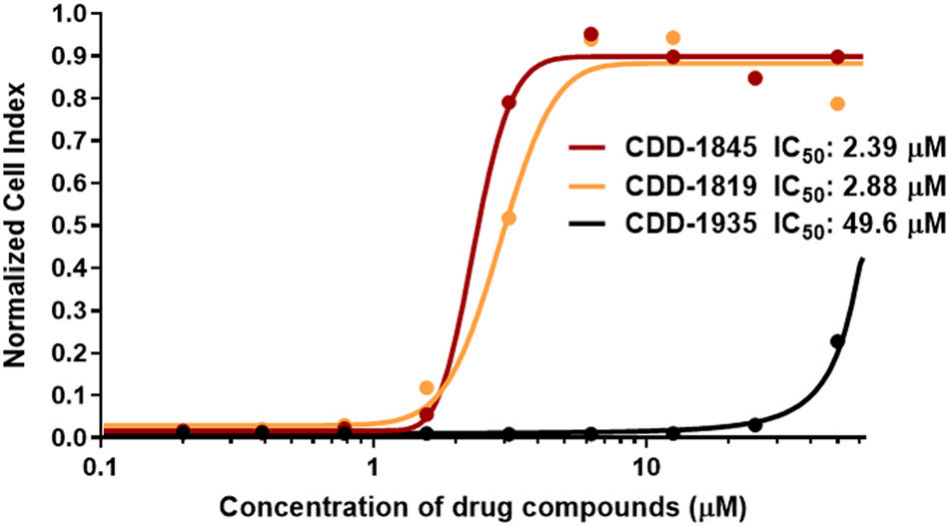
Concentration of M^pro^ drug compounds versus normalized cell index at 75 h incubation measured by the xCELLigence RTCA system. Average data points from duplicate measurements. A sigmoidal dose-response curve was fitted to determine IC_50_ values for each M^pro^ drug compound (lines).

We next examined the capacity of compounds **CDD-1819**, **CDD-1845**, and **CDD-1935** to inhibit viral replication in cells. Real-time cell analysis (RTCA) assay was used to compare the viral inhibitory capacity of compounds **CDD-1819**, **CDD-1845**, and **CDD-1935**, as reported in our previous study^17^. RTCA is an extensively validated and label-free system for real-time monitoring of viral infections in vitro^28–30^. Healthy cells were given a normalized cell index of 1, indicating normal cell proliferation. For infected cells, we observed a significant decrease (99%) in the normalized cell index. Figure 4 shows a dose-response curve of the M^pro^ inhibitors in relation to the normalized cell index. The addition of low micromolar concentrations of **CDD-1845** (IC_50_ = 2.39 μM) and **CDD-1819** (IC_50_ = 2.88 μM) was found effective in restoring the normalized cell index, indicating that cell proliferation was unaffected and virus replication was completely prevented. **CDD-1935** only partially restored the natural cell growth at higher concentrations.

**Fig. 5:**
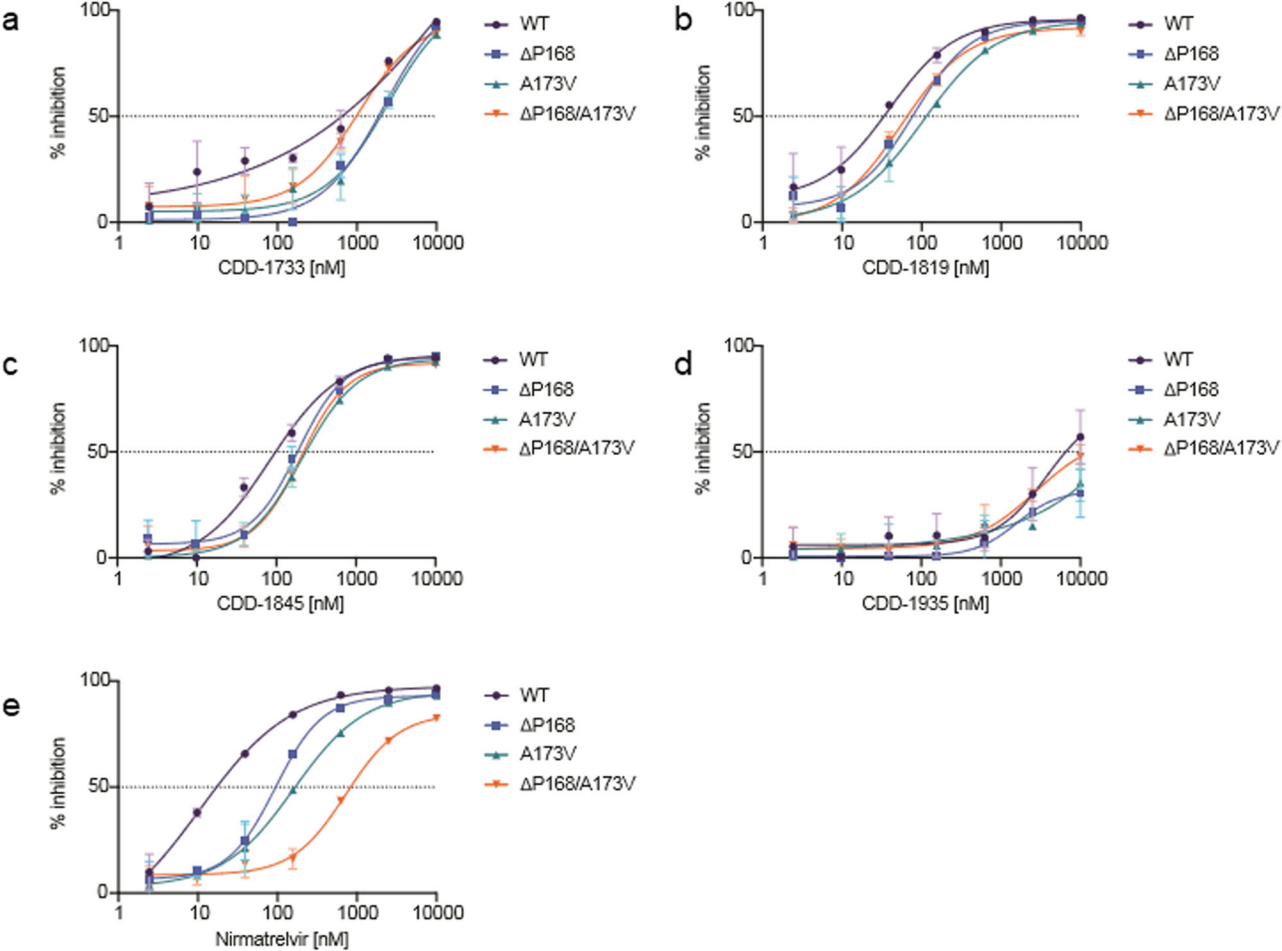
Dose-response curves of M^pro^ inhibitors with WT, ΔP168, A173V, and ΔP168/A173V M^pro^ variants using the live cell Src-M^pro^-Tat-fLuc assay with 4-fold serial dilution of inhibitor beginning at 10 µM (data are mean +/- SD of biologically independent triplicate experiments). **a** Dose-response curves of CDD-1733. **b** Dose-response curves of CDD-1819. **c** Dose-response curves of CDD-1845. **d** Dose-response curves of CDD-1935. **e** Dose-response curves of Nirmatrelvir.

Finally, we wanted to explore the activity of the most active compounds to relevant M^pro^ mutants. We recently used our gain-of-signal live cell assay to identify ΔP168 and A173V as naturally occurring variants of SARS-CoV-2 M^pro^ capable of conferring resistance to nirmatrelvir^10^. Given the distinct binding modes of the inhibitors described here, we hypothesized that they might show different resistance profiles to that of nirmatrelvir in this assay. In line with in vitro results above, **CDD-1935** showed little inhibitory activity at the concentrations tested in contrast to **CDD-1733**, **CDD-1819**, and **CDD-1845** which all showed potent activity in a cellular context with IC_50_ values of 648, 34, and 98 nM respectively (Fig. 5). While ΔP168 caused a 5.5-fold decrease in potency to nirmatrelvir, **CDD-1733**, **CDD-1819**, and **CDD-1845** all show greater resilience with all IC_50_ values remaining within 3-fold of WT (Supplementary Fig. 9). A173V shows a greater resistance phenotype to nirmatrelvir with a nearly 10-fold decrease in potency, whereas again **CDD-1733**, **CDD-1819**, and **CDD-1845** all maintain IC_50_ values within 4-fold of WT. Most strikingly, ΔP168 and A173V cause a synergistic 48-fold resistance to nirmatrelvir, and, in contrast, all three compounds show a modest diminution (<2.2-fold) in potency. These results are further corroborated using the in vitro biochemical protease inhibition assay. The ΔP168 variant was unstable upon expression in *E. coli* and thus could not be included in this analysis. We had previously shown that A173V and ΔP168/A173V increase the *K*_i_ of nirmatrelvir by 50- and 600-fold, respectively^10^. Although A173V and ΔP168/A173V cause a 50- and 10-fold increase in *K*_i_ for **CDD-1733**, respectively, **CDD-1819** and **CDD-1845** maintain *K*_i_ values within 4-fold of WT for both single and double mutants (Supplementary Fig. 10). Together, these results demonstrate that the inhibitors identified here show a distinct resistance profile in comparison to that of nirmatrelvir.

## Conclusions

In summary, we utilized DECLs to produce novel non-peptidic, non-covalent small molecule inhibitors for SARS-CoV-2 M^pro^ without the aid of preliminary knowledge regarding suitable starting points. By performing an affinity selection with M^pro^ using 4-billion DNA-barcoded molecules, we enriched for compounds having a benzimidazole core structure and no discernable covalent warhead. Validation of the compounds by off-DNA synthesis and a minimal medicinal chemistry campaign rapidly led to the synthesis of the high-affinity M^pro^ inhibitors **CDD-1733**, **CDD-1819**, and **CDD-1845**. These compounds were further shown to prevent viral replication in cells using an in vitro viral replication assay. Finally, the compounds identified in this study demonstrated a greater ability to inhibit clinically isolated mutants associated with resistance to nirmatrelvir, which is now the leading drug used clinically to treat severe SARS-CoV-2 infections. Notably, the lead structures identified from these studies are structurally distinct from other M^pro^ inhibitors that are based on conventional covalent warheads and peptide-based scaffolds.

Nirmatrelvir is heralded as a tour de force of modern pharmaceutical development; however, its rapid clinical deployment was buoyed by nearly two decades of prior research aimed at the 2002 SARS-CoV virus. It is likely an inevitability that future pandemics will arise where scant information is known about the viral pathogen and its therapeutically targetable genes. In such a scenario, a rapid, non-biased, and diverse chemical screen would be required to discover active small molecule candidates for putative targets. Additionally, the rapid mutation and selection of novel viral strains puts a continual burden on the need to synthetically “evolve” better drugs to keep pace. The studies described here illuminate DEC-Tec as an effective and rapid platform for pursuing inhibitors of important viral targets apart from preexisting biases related to the chemical matter. We therefore contend that DECLs are an important tool in the arsenal aimed at preserving human health in the face of future pandemics.

## Methods

### Plasmid and mutant construction

*E. coli* XL1-Blue (recA1, endA1, gyrA96, thi-1, hsdR17, supE44, relA1, lac, [F9 proAB lacIq lacZΔM15, Tn10 (tetr)]) (Stratagene, Inc.) was used as the host for plasmid and mutant variant construction. *E. coli* BL21(DE3) (fhuA2 [lon] omp Tgal (λDE3) [dcm] ΔhsdS λ DE3=λ sBamHIo ΔEcoRI-B int::(lacI::PlacUV5::T7gene1) i21 Δnin5) was used as the host for protein expression. The construction of the SARS-CoV-2 M^pro^ protein expression plasmid has been described (Chamakuri et al.). Briefly, the gene for M^pro^ (ORF1ab residues 3264 to 3569, GenBank code: MN908947.3) was codon-optimized for *E. coli* expression, synthesized, and amplified by PCR to introduce an N-terminal M^pro^ cleavage site (SAVLQ ↓ SGFRK) and a C-terminal PreScission protease cleavage site (SGVTFQ ↓ GP) followed by a 6xHis-tag^31^. The PCR product was digested with NdeI and BamHI restriction enzymes and ligated into the pSumo plasmid to generate an N-terminal Sumo-M^pro^ fusion construct pSUMO-SARS-CoV-2-M^pro^ (Chamakuri et al.). The DNA sequence of the resulting plasmid was confirmed by Sanger sequencing.

To create the M^pro^ variants, primers containing the desired mutations were designed and received from Integrated DNA Technologies (Coralville, IA). A173V primer (5’- GAATTGCCTACG GGCGTCCATGTCGGGACTGACTTGGAGGG-3’), del-P168 primer (5’-CTGTTATATGCATCACATGGA ATTGACGGGCGTCCATGCCGGGACTG-3’) and A173V-del-P168 primer (5’-GTTATATGCATCACATGG AATTGACGGGCGTCCATGTCGGGACTGACTTGGAGGG-3’) were phosphorylated using 10 units of T4 Polynucleotide Kinase (New England Biolabs, Ipswich, MA) and 25 µM primer stock solutions. By QuikChange mutagenesis, the desired DNA sequences were generated using 1 unit of Phusion DNA polymerase (New England Biolabs), the corresponding phosphorylated primer, and the pSUMO-SARS-CoV-2 M^pro^ plasmid as the template. The resulting thermocycler products were digested with 20 units of DpnI (New England Biolabs). Electrocompetent *E. coli* XL1-Blue cells (Agilent, Santa Clara, CA) were transformed with the DpnI-digested DNA, and the plasmids were selected on LB agar supplemented with 25 µg/mL kanamycin. LB media containing 25 µg/mL kanamycin was inoculated with a single colony, and the liquid cultures were incubated at 37 °C overnight. The next day, the plasmid DNA was isolated using the Zyppy Plasmid Miniprep Kit (Zymo Research, Irvine, CA). The DNA sequences with the desired mutations were confirmed by Sanger Sequencing (Genewiz, Plainfield, NJ).

### Protein expression and purification

SARS-CoV-2 M^pro^ with a C-terminal 6xHistag was expressed and purified from the *E. coli* BL21(DE3) strain as previously described (Chamakuri et al.). *E. coli* cells were cultured in LB medium at 37 °C to an optical density at 600 nm absorbance of 0.8, and enzyme expression was induced with 0.4 mM isopropyl β-D-1-thiogalactopyranoside for 20 h at 20 °C. The N-terminal Sumo-fusion was auto-cleaved in *E. coli* to generate an authentic M^pro^ N terminus. Cells were collected by centrifugation and resuspended in binding buffer (25 mM Tris·HCl pH = 8.0, 150 mM NaCl, 5 mM β-mercaptoethanol, 20 mM imidazole, and Xpert protease inhibitor mixture [GenDEPOT]). Cells were disrupted by sonication, and debris was cleared by centrifugation at 10,000×*g* for 10 min at 4 °C. The supernatant was fractionated using a Ni^2+^ Sepharose 6 Fast Flow resin (GE Healthcare) column. After washing, the enzyme was eluted with buffer A (25 mM Tris·HCl, pH 8.0, 150 mM NaCl, 5 mM β-mercaptoethanol, and Xpert protease inhibitor) containing 40, 60, 80, and 100 mM imidazole, respectively. The purity of M^pro^ in each fraction was assessed by sodium dodecyl sulfate-polyacrylamide gel electrophoresis (SDS/PAGE). Fractions with >95% pure M^pro^ were pooled and buffer-exchanged with buffer B (20 mM Tris·HCl, pH 7.3, 150 mM NaCl, 1 mM ethylenediaminetetraacetic acid [EDTA], and 1 mM dithiothreitol [DTT]) with an Amicon Ultra-15 Centrifugal Filter (MilliporeSigma). Purified M^pro^ was then cleaved with PreScission protease to generate M^pro^ with authentic N and C termini. GST Bind Resin (Novagen) and Ni^2+^ Sepharose 6 Fast Flow resin were used sequentially to remove GST-tagged PreScission protease and M^pro^-His. Uncleaved M^pro^-His and M^pro^ were fractionated on a Superdex 75 increase 10/300GL size-exclusion column (GE Healthcare) preequilibrated with buffer B fractions were analyzed by SDS/PAGE, and pure M^pro^ protein was pooled for further use.

### DEC-Tec affinity selection with M^pro^

The DEC-Tec selection and sequencing were performed exactly as we described in our previous reports (Supplementary methods)^17^.

### Enzyme inhibition assay and determination of *K*_i_ values

The inhibition potency of compounds for M^pro^ was evaluated by *K*_i_ measurements using the fluorescent peptide Dabcyl-KTSAVLQSGFR KM-E(Edans)-NH2 (GenScript Biotech) as the reporter substrate at a concentration of 15 μM. Increasing concentrations of compounds (from 4 to 4000 nM with twofold dilutions) were incubated with 25 nM M^pro^ for 20 min at RT in reaction buffer composed of 20 mM Tris·HCl, pH 7.3, 100 mM NaCl, 1 mM EDTA, 1 mM DTT, and 0.02% Tween-20. Hydrolysis of the fluorescent peptide was monitored at an emission wavelength of 460 nm with an excitation wavelength of 360 nm using a TECAN M200 plate reader. Initial hydrolysis rates of fluorescent peptide were plotted as a function of compound concentrations, and *K*_i_ values were obtained by fitting the data into the Morrison equation^32^ with SE from triplicates. The Michaelis–Menten constant of the enzyme (*K*_m_) value used for *K*_i_ calculations is 17 μM^33^.

The Morrison equation used was

$$Y=	\,Vo*(1-((((Et+X+(Ki*(1+(S/Km))))\\ 	-(((Et+X+(Ki*(1+(S/Km))))^2)-4*Et*X)^{.5}))/(2*Et)))$$

where Y is enzyme activity, X is the concentration of inhibitor, Et is enzyme concentration, and S is substrate concentration.

### Human protease assays

These assays were done exactly as we described earlier^17^. In brief, commercial kits were used for all four human proteases as follows: Cathepsin B (BPS Bioscience, #79590), Renin (BPS Bioscience, #80211), Thrombin (Sigma-Aldrich, #MAK243), and MMP-1 (Biovision, #K794-100). All assays were run in kinetic mode and at RT, except MMP-1, which was run at 37 °C. Positive controls were used for all assays, i.e., E64 for Cathepsin B, Aliskiren for Renin, CDD-1472 for Thrombin, and GM 6001 for MMP-1. Activities were obtained from linear initial progress curves, and fractional activities were calculated from reactions with no inhibitors^17,19,34^.

### Crystallography and data collection

To obtain the structure of M^pro^ in complex with CDD-1733, CDD-1819, and CDD-1845, M^pro^ and all three inhibitors were mixed at a 1:2 molar ratio and incubated at 4 °C overnight to facilitate complex formation. Crystal screening was performed using commercially available crystal screening suites PEGs, PEGII, PACT, and JCSGI from Qiagen in 96-well format. Hanging drops were set up by an in-house TTP LabTech Mosquito instrument, and crystals were obtained through the vapor diffusion method. Crystals were obtained in a condition of 20% (wt/vol) PEG3350 and 0.2 M sodium acetate at RT and 25% glycerol was used as the cryoprotectant. X-ray diffraction data were collected at the Berkeley Center for Structural Biology using the Advanced Light Source synchrotron beamline. Reflection data were indexed, integrated, and scaled using the iMosflm and the CCP4i Suite^35^. Molecular replacement was performed using the M^pro^ structure (Protein Data Bank ID code 7K3T) as the search model. Structures were further refined several rounds using PHENIX.refine and Crystallography Object-Oriented Toolkit (Coot)^36^. The data collection and refinement statistics are listed in Supplementary Data 2. The UCSF Chimera program was used to generate Fig. 3. The electron densities of CDD-1733 and the non-covalent bond between residue M^pro^ Cys145 and CDD-1733 in the crystal structure were further examined by creating a polder OMIT map using the PHENIX software^37,38^.

### Cell uptake assay and cytotoxicity assay in HepG2

The HepG2 cells were maintained in Dulbecco’s modified Eagle’s medium (DMEM, containing 1 g/L glucose, 10% fetal bovine serum [FBS], and 1% penicillin/streptomycin). For cell uptake assay, the cells were seeded in a 12-well plate at a final density of 5 × 10^5^ per well and incubated at 37 °C for 24 h before drug treatment.

#### Cell uptake

The cells were treated with CDD-1733 or CDD-1819 or CDD-1845 (final concentration 10 μM and final DMSO concentration 0.1%). The plate was incubated at 37 °C for another 2 h, and the medium in the plate was then decanted. The cells were rinsed with 1 mL of 1× DPBS three times, trypsinized at 37 °C for 3.5 min, centrifuged, and the cell pellets from each well were reconstituted in 500 μL of MeOH/H_2_O (vol/vol 1/1). CDD-1733 or CDD-1819 or CDD-1845 was extracted by mixing 100 μL of the cell lysate with 100 μL of ice-cold methanol. The mixtures were centrifuged at 15,000 rcf for 15 min. Three microliters of the supernatant were injected into UHPLC-Q Exactive Orbitrap MS (Thermo Fisher Scientific) equipped with a 50 × 4.6-mm column (XDB C-18; Agilent Technologies) for analysis. The column temperature was set at 40 °C. The mobile phase system was (A) water (containing 0.1% formic acid) and (B) acetonitrile (containing 0.1% formic acid), with a flow rate of 0.3 mL/min. The gradient elution program was set as follows: 0 to 0.25 min, 40% B; 0.25 to 1.5 min, 40 to 98% B; 1.5 to 3.5 min, 98% B; 3.5 to 3.8 min, 98 to 40% B; 3.8 to 5 min, 40% B. Ultrapure nitrogen was used as the sheath (45 arbitrary units), auxiliary (10 arbitrary units), and sweep (1.0 arbitrary unit) gas. The capillary and auxiliary gas temperatures were 275 °C and 380 °C, respectively. The spray voltage was 3.75 kV. Positive full-scan MS data were acquired from 80 to 1,200 Da in profile mode. Doxorubicin and dacarbazine were used as positive and negative controls, respectively. The experiments were performed in triplicate.

#### Cytotoxicity assay

For cytotoxicity assay, HepG2 cells were seeded in 96-well plates at a final density of 5 × 10^4^ per well and incubated at 37 °C for 24 h before drug treatment. The cells were treated with CDD-1733 or CDD-1819 or CDD-1845 at the final concentrations of 0, 50, 75, or 100 μM (3 replicates per concentration level per drug). After 24 h incubation at 37 °C, the medium in the plate was decanted. The cell viability was measured with an XTT kit (Biotium) by mixing 100 μL of DMEM (without phenol red) with 25 μL of XTT reagent and adding it into each well. The absorbance at 475 nm was read with a Tecan M1000 pro plate reader, with a reference wavelength of 660 nm. The reading was normalized by vehicle with the final 0.5% dimethylsulfoxide (DMSO) for each drug.

### Plasma stability assay

Compounds CDD-1733, CDD-1819, and CDD-1845 at 10 μM were incubated in human and mouse plasma in duplicate at 37 °C, respectively. At time points of 0, 30, 60, and 120 min, 15 μL of the samples were taken out, and the reactions were terminated by adding 75 μL of ice-cold methanol. The reaction mixtures were then vortexed for 30 s and centrifuged at 15,000 rcf for 15 min. Following centrifugation, 3 μL of the supernatant was injected into the UHPLC-Q Exactive Orbitrap MS and analyzed with the same parameters used in the cell uptake assay above. The percentage of test compound remaining at the individual time points was normalized by the 0-min.

### Pharmacokinetics of CDD-1733, CDD-1819, or CDD-1845 in mice

WT mice (*n* = 4) were administered with CDD-1733, CDD-1819, or CDD-1845 (at a dose of 50 mg/kg, intraperitoneal injection) dissolved in 0.5% (w/v) methyl cellulose (injection volume 10 µL/g), individually. About 20 µL of blood (anticoagulated by sodium heparin) were collected via the tail vein at 0 (pre-dose), 5 min, 10 min, 0.25, 0.5, 1.0, 2.0, 4.0, 6.0, 8.0, and 24 h time points post-dose. The whole blood samples were centrifuged at rcf 2000 for 5 min for the plasma separation of plasma. The plasma samples were stored at −80 °C before analysis. All the samples were analyzed with a Q Exactive orbitrap MS coupled with UHPLC (Thermo Fisher Scientific, San Jose, CA). LC-Q Exactive MS was operated in the same parameters used in the cell uptake assay above. The concentration of these compounds in plasma was quantified using individual calibration curves. The calibration curve was regressed with a weight of 1/x^2^ with high linearity (r^2^ > 0.99) and accuracy (RSD < 15%).

PK parameters such as half-time (T_1/2_), area under the plasma concentration–time curve during the period of observation (AUC_0–t_), area under the plasma concentration–time curve from zero to infinity (AUC_0–∞_), clearance normalized by the bioavailability (CL/F), and the mean residence time (MRT) were calculated by WinNonlin software (Certara, Princeton, NJ) by noncompartmental analysis. The plasma concentration–time curves were plotted in Prism 7 (GraphPad, San Diego, CA) as mean ± S.D.

### RTCA assay for evaluating in vitro inhibition of SARS-CoV-2 replication by M^pro^ inhibitors

xCELLigence RTCA (Agilent Technologies) was used to indirectly quantify the in vitro inhibition of SARS-CoV-2 replication in VERO E6 cells by monitoring the virus-induced cytopathic effect on the cell index (a measure for cell proliferation). The protocol was recently described elsewhere^39^. Briefly, 1 ×10^4^ VERO E6 cells were seeded in each well of an xCELLigence E-plate. The cells were allowed to settle for 30 min at room temperature before monitoring cell proliferation for 24 h in the RTCA HT Analyzer (baseline for normal cell growth). Serial dilutions of the M^pro^ drug candidates were prepared in cell media. Diluted drug candidates and controls were mixed in a 1:1 ratio with 55 plaque-forming units of SARS-CoV-2 (USnA-WA1/2020) in cell media. The final concentrations of the M^pro^ drug candidates ranged from 0.2 to 50 μM. The drugs/virus mixtures were incubated for 1 h at 37 °C 5% CO_2_ before being added to the VERO E6 cells in the xCeLLigence instrument. Subsequently, the cell index of the infected cells was monitored for 75 h. Controls included virus only, cell culture media only, and DMSO^40^.

Data were analyzed using the RTCA software (Agilent Technologies). Duplicate wells were averaged, and the cell index at each time was normalized to the cell index before the addition of the drugs/virus mixture. Using Prism9 software (GraphPad), the normalized cell index data was plotted versus the concentration of the drug compound, and the half-maximal inhibitory concentration (IC_50_) of each compound was calculated.

### Gain of signal live cell SARS-CoV-2 M^pro^ assay

The cell based reporter assays for comparing efficacy of the selected compounds against WT and mutant M^pro^ were performed using our previously described gain-of-signal luciferase based assay^10^. The ΔP168, A173V and ΔP168/A173V mutants were generated by site-directed mutagenesis in the pcDNA5/TO-Src-M^pro^-Tat-fLuc plasmid^10^ and confirmed by Sanger sequencing. 293T cells were maintained at 5% CO_2_ and 37 °C in DMEM (Gibco #11875093) supplemented with 10% fetal bovine serum (ThermoFisher #11965084) and penicillin-streptomycin (Gibco # 15140122). To perform the assay, 3 × 10^6^ 293T cells were seeded in a 10 cm dish and transfected 24 h later with corresponding 2 µg of the Src-M^pro^-Tat-fLuc plasmid (WT or mutant) using TransIT-LT1 transfection reagent (Mirus #MIR 2304). At 4h post-transfection, cells were washed once with PBS-EDTA, trypsinized, resuspended in fresh media and diluted to a concentration of 4 × 10^5^ cells/mL. Dilution series of inhibitors were prepared using fresh media and added to a 96-well flat clear bottom white plate (Corning #3610) and 50 µL of the cell suspension was subsequently added to the plate to yield a final cell density of 2 × 10^6^ cells/well. At 48h post transfection, media was removed from the wells and 50 µL of Bright-Glo reagent (Promega #E2620) was added to each well and incubated for 5 m before measuring luminescence using the Biotek Synergy H1 plate reader. Percent inhibition at each concentration of inhibitor was derived with the formula below using the relative luminescence (RL) of an inhibitor treated sample to the untreated control. % inhibition = 100-(100/(RL)) Results were plotted using GraphPad Prism 9 and fit using a four-parameter non-linear regression to calculate IC_50_. Resistance of mutants was calculated by the fold change in IC_50_ of the mutant relative to WT M^pro^.

### Reporting summary

Further information on research design is available in the Nature Portfolio Reporting Summary linked to this article.

### Supplementary information

Supplementary Information

Description of Additional Supplementary Files

Supplementary Data 1

Supplementary Data 2

Reporting Summary

## Data Availability

The corresponding data of this study are available within the paper and the supplementary information, Supplementary Data 1 and 2. Crystal structure of M^pro^ in complex with inhibitors described in this study are available at PDB: 7URB, PDB: 7US4, and PDB: 7UR9.
