# Supplementary material for: DNA-encoded chemical libraries yield non-covalent and non-peptidic SARS-CoV-2 main protease inhibitors
Source: Commun Chem. 2023 Aug 4;6:164. doi: 10.1038/s42004-023-00961-y (PMC10403511; doi:10.1038/s42004-023-00961-y)
Supplement: Supplementary file 1 — Supplementary Information [file 42004_2023_961_MOESM1_ESM.pdf]

DNA-Encoded Chemical Libraries Yield Non-covalent and Non-peptidic SARS-CoV-2 Main Protease Inhibitors

Ravikumar Jimmidi[1,#], Srinivas Chamakuri[1,#,*], Shuo Lu[2, #], Melek Nihan Ucisik[1,§], Peng-Jen Chen[1,§], Kurt M. Bohren[1], Seyed Arad Moghadasi[3], Leroy Versteeg[4,5], Christina Nnabuife[2], Jian-Yuan Li[1], Xuan Qin[1], Ying-Chu Chen[1], John C. Faver[1], Pranavanand Nyshadham[1], Kiran L. Sharma[1], Banumathi Sankaran[6], Allison Judge[2], Zhifeng Yu[1], Feng Li[1,2], Jeroen Pollet[4,5], Reuben S. Harris[7,8], Martin M. Matzuk[1,2], Timothy Palzkill[2]*, and Damian W. Young[1,2]*

[1]Center for Drug Discovery, Department of Pathology & Immunology, Baylor College of Medicine, Houston, Texas 77030 USA

[2]Verna and Marrs McLean Department of Biochemistry and Molecular Pharmacology, Baylor College of Medicine, Houston, Texas 77030 USA

[3]Department of Biochemistry, Molecular Biology, and Biophysics, University of Minnesota – Twin Cities, Minneapolis, Minnesota 55455 USA

[4]Department of Pediatrics, National School of Tropical Medicine, Baylor College of Medicine, Houston, Texas 77030 USA

[5]Center for Vaccine Development, Texas Children's Hospital, 1102 Bates Street, Houston, Texas 77030 USA

[6]Department of Molecular Biophysics and Integrated Bioimaging, Berkeley Center for Structural Biology, Lawrence Berkeley National Laboratory, Berkeley, California 94720 USA

[7]Department of Biochemistry and Structural Biology, University of Texas Health San Antonio; San Antonio, Texas 78229 USA

[8]Howard Hughes Medical Institute, University of Texas Health San Antonio; San Antonio, Texas 78229 USA

#, equal first author

§, equal contribution

*, communicating authors, Email: srinivas.chamakuri@bcm.edu or timothyp@bcm.edu or damian.young@bcm.edu

Table of Contents

Content	Page
Supplementary figures	S2-S16
DECL general methods, experimental procedures and NMR data	S17-S45

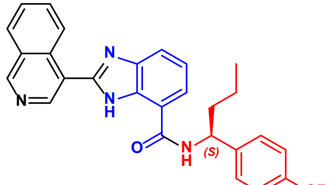

CDD-1789
Inactive

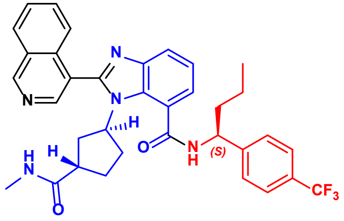

CDD-1790
620 nM

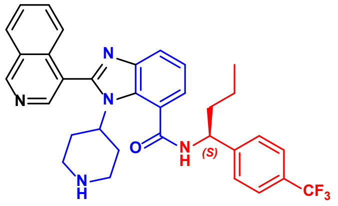

CDD-1818
1470 nM

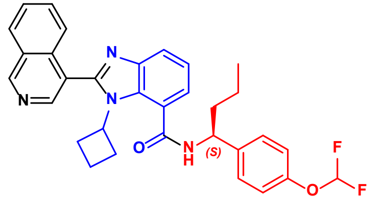

CDD-1820
67 nM

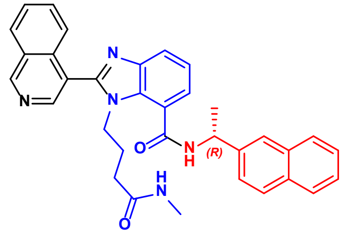

CDD-1935
2275 nM

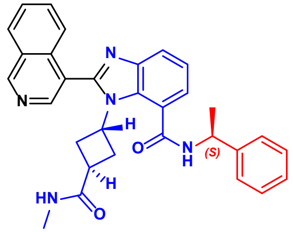

CDD-1841
571 nM

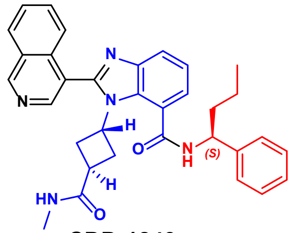

CDD-1843
Inactive

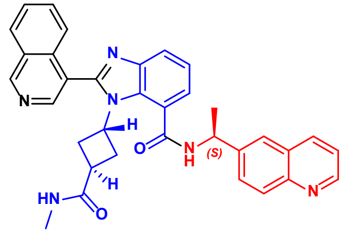

CDD-1907
1187 nM

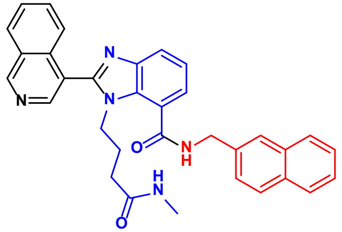

CDD-1925
51 nM

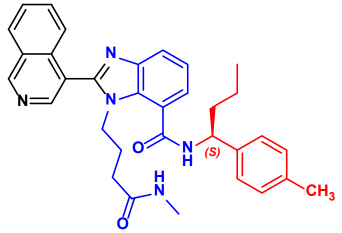

CDD-1934
29 nM

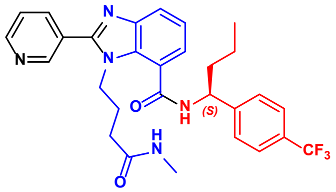

CDD-1846
670 nM

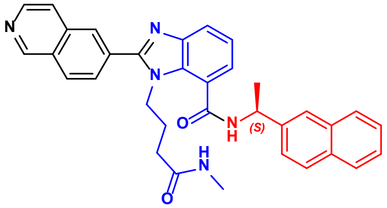

CDD-1884
Inactive

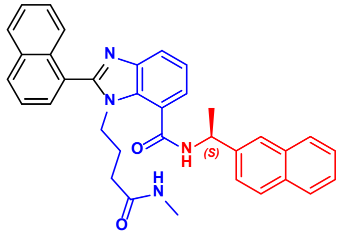

CDD-1885
Inactive

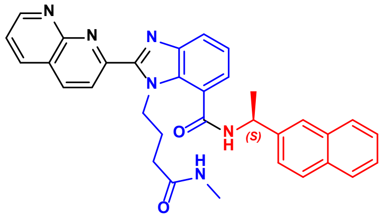

CDD-1905
Inactive

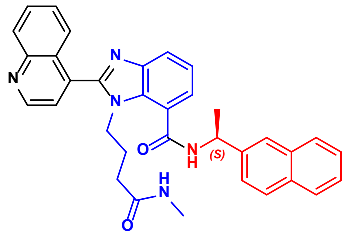

CDD-1906
Inactive

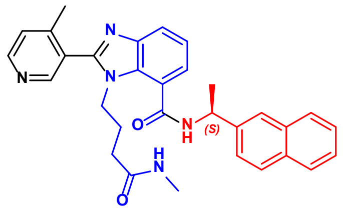

CDD-1924
374 nM

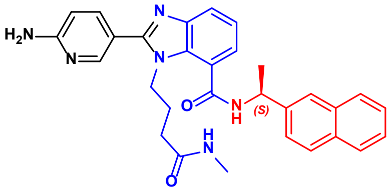

CDD-1926
Inactive

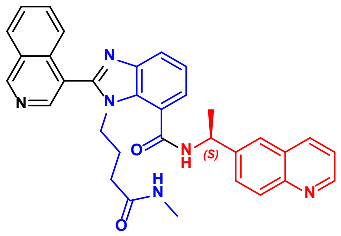

CDD-1908
764 nM

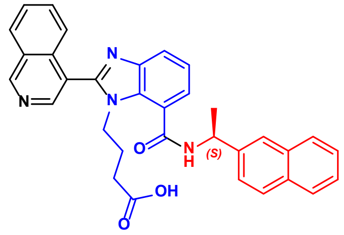

CDD-1972
85 nM

Supplementary Figure 1: Synthesized analogs

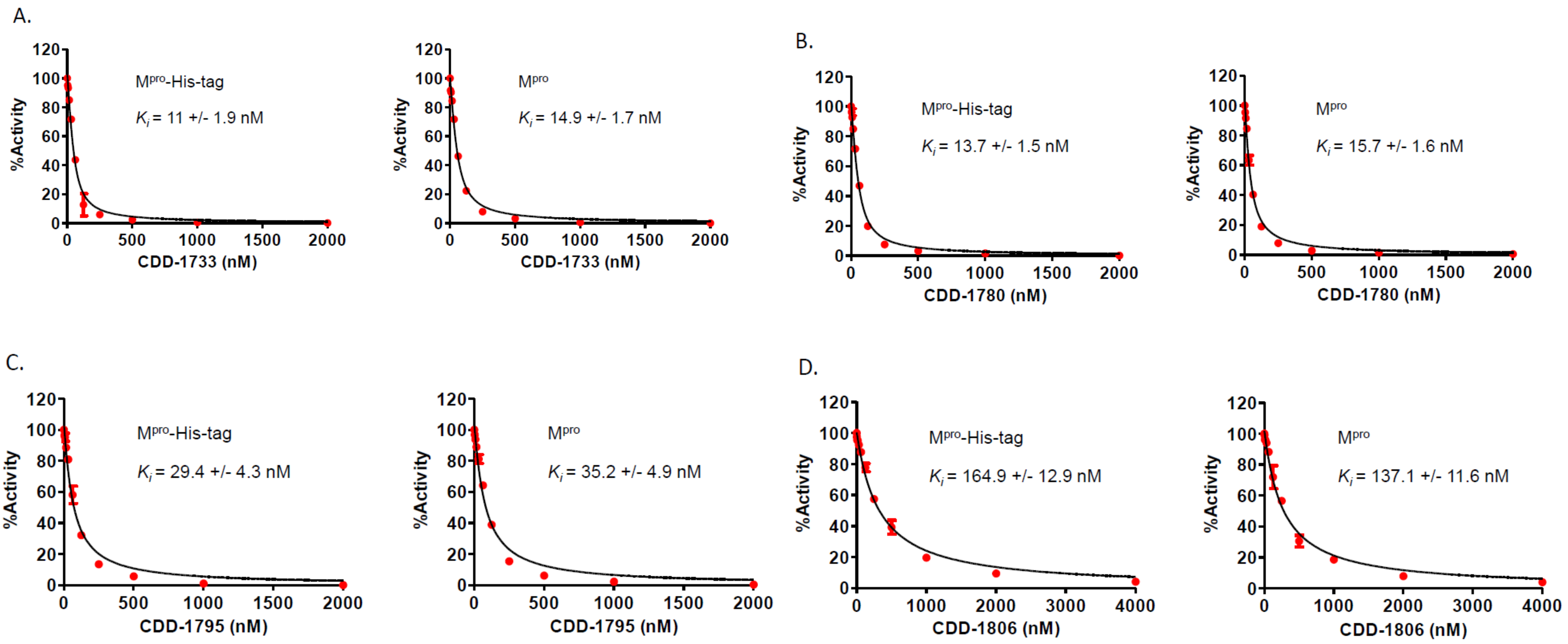

A.
120
100
80
60
40
20
0
%Activity
0
500
1000
1500
2000
CDD-1733 (nM)
Mpro-His-tag
K_i = 11 +/- 1.9 nM
Mpro
K_i = 14.9 +/- 1.7 nM
B.
CDD-1780 (nM)
Mpro-His-tag
K_i = 13.7 +/- 1.5 nM
Mpro
K_i = 15.7 +/- 1.6 nM
C.
CDD-1795 (nM)
Mpro-His-tag
K_i = 29.4 +/- 4.3 nM
Mpro
K_i = 35.2 +/- 4.9 nM
D.
3000
4000
CDD-1806 (nM)
Mpro-His-tag
K_i = 164.9 +/- 12.9 nM
Mpro
K_i = 137.1 +/- 11.6 nM

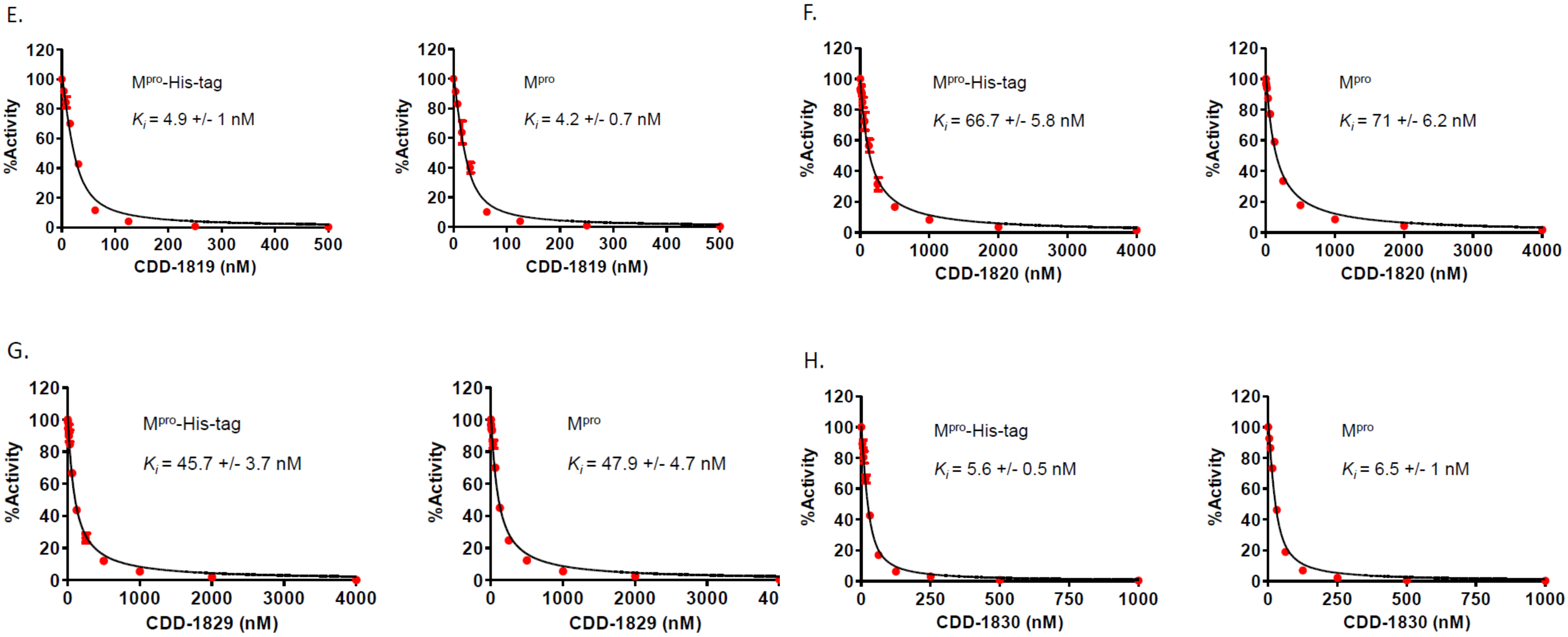
E.
Mpro-His-tag
Ki = 4.9 +/- 1 nM
%Activity
CDD-1819 (nM)
Mpro
Ki = 4.2 +/- 0.7 nM
%Activity
CDD-1819 (nM)
F.
Mpro-His-tag
Ki = 66.7 +/- 5.8 nM
%Activity
CDD-1820 (nM)
Mpro
Ki = 71 +/- 6.2 nM
%Activity
CDD-1820 (nM)
G.
Mpro-His-tag
Ki = 45.7 +/- 3.7 nM
%Activity
CDD-1829 (nM)
Mpro
Ki = 47.9 +/- 4.7 nM
%Activity
CDD-1829 (nM)
H.
Mpro-His-tag
Ki = 5.6 +/- 0.5 nM
%Activity
CDD-1830 (nM)
Mpro
Ki = 6.5 +/- 1 nM
%Activity
CDD-1830 (nM)

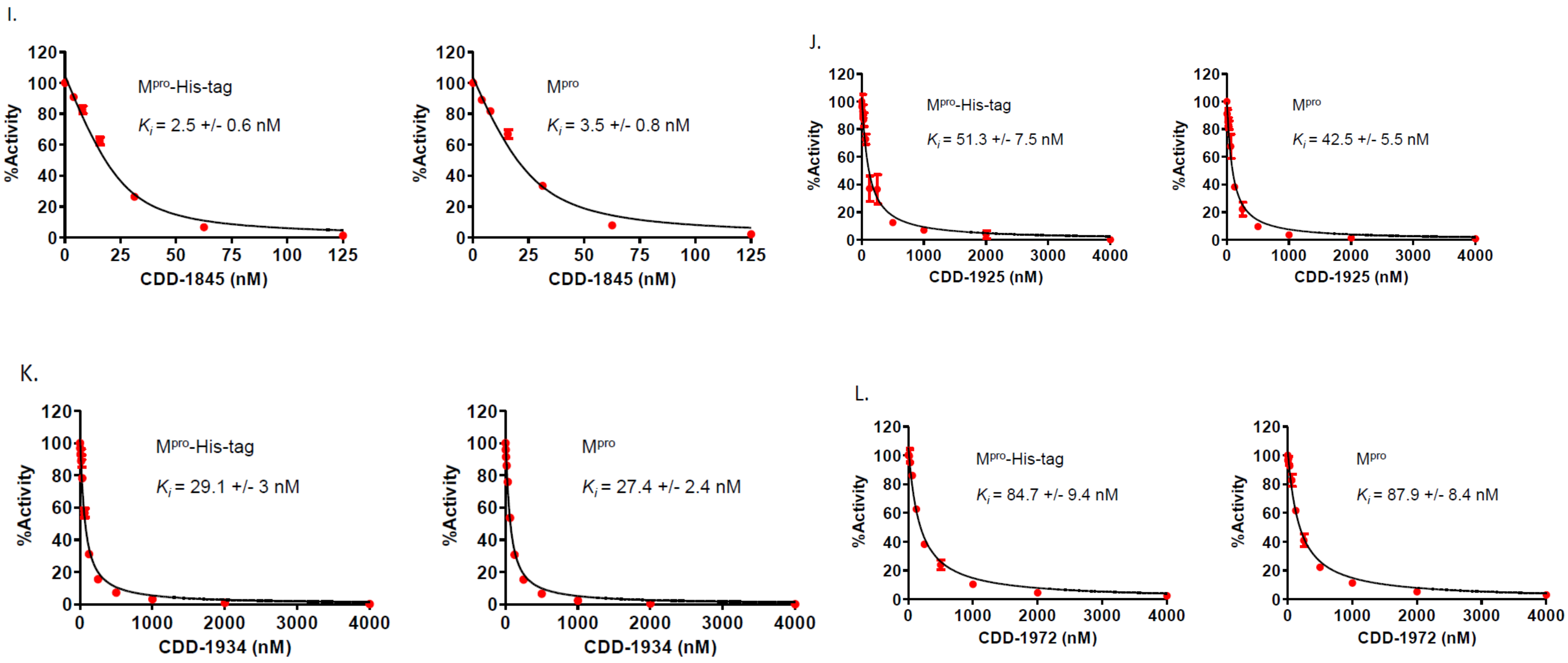

Supplementary Figure 2: Inhibition K_i value determination for CDD-1733 series against M^{pro}. A-L: Concentration-dependent inhibition curves of individual inhibitors. 25 nM of M^{pro}-6-His or M^{pro} was mixed with increasing concentrations of inhibitors. The remaining activities (red dots) of M^{pro} towards fluorescent peptide were plotted as a function of compound concentrations and K_i values was calculated by fitting the data into Morrison equation with standard error from triplicates.

Compound	M^{pro}-His K_i (nM)	M^{pro} K_i (nM)
CDD-1732	657±56	806±124
CDD-1733	12±2	18±4
CDD-1780	14±1	16±2
CDD-1789	Inactive[a]	Inactive
CDD-1790	620±46	664±66
CDD-1795	29±4	35±5
CDD-1804	Inactive	Inactive
CDD-1806	165±13	137±12
CDD-1818	1470±152	1714±128
CDD-1819	5±1	4±1
CDD-1820	67±6	71±6
CDD-1829	46±4	48±5
CDD-1830	6±1	6±1
CDD-1831	Inactive	Inactive
CDD-1841	571±73	565±55
CDD-1842	Inactive	Inactive
CDD-1843	Inactive	Inactive
CDD-1845	3±1	3±1

Compound	M^{pro}-His K_i (nM)	M^{pro} K_i (nM)
CDD-1846	670±60	805±65
CDD-1884	Inactive	Inactive
CDD-1885	Inactive	Inactive
CDD-1905	Inactive	Inactive
CDD-1906	Inactive	Inactive
CDD-1907	1187±120	1330±84
CDD-1908	764±66	852±74
CDD-1924	374±23	426±47
CDD-1925	51±7	42±5
CDD-1926	Inactive	Inactive
CDD-1934	29±3	27±2
CDD-1935	2275±197	2301±201
CDD-1972	85±9	88±8

Supplementary Table 1. Enzymatic activity ; [a] Less than 50% inhibition observed with 25 μM compound added for initial screening

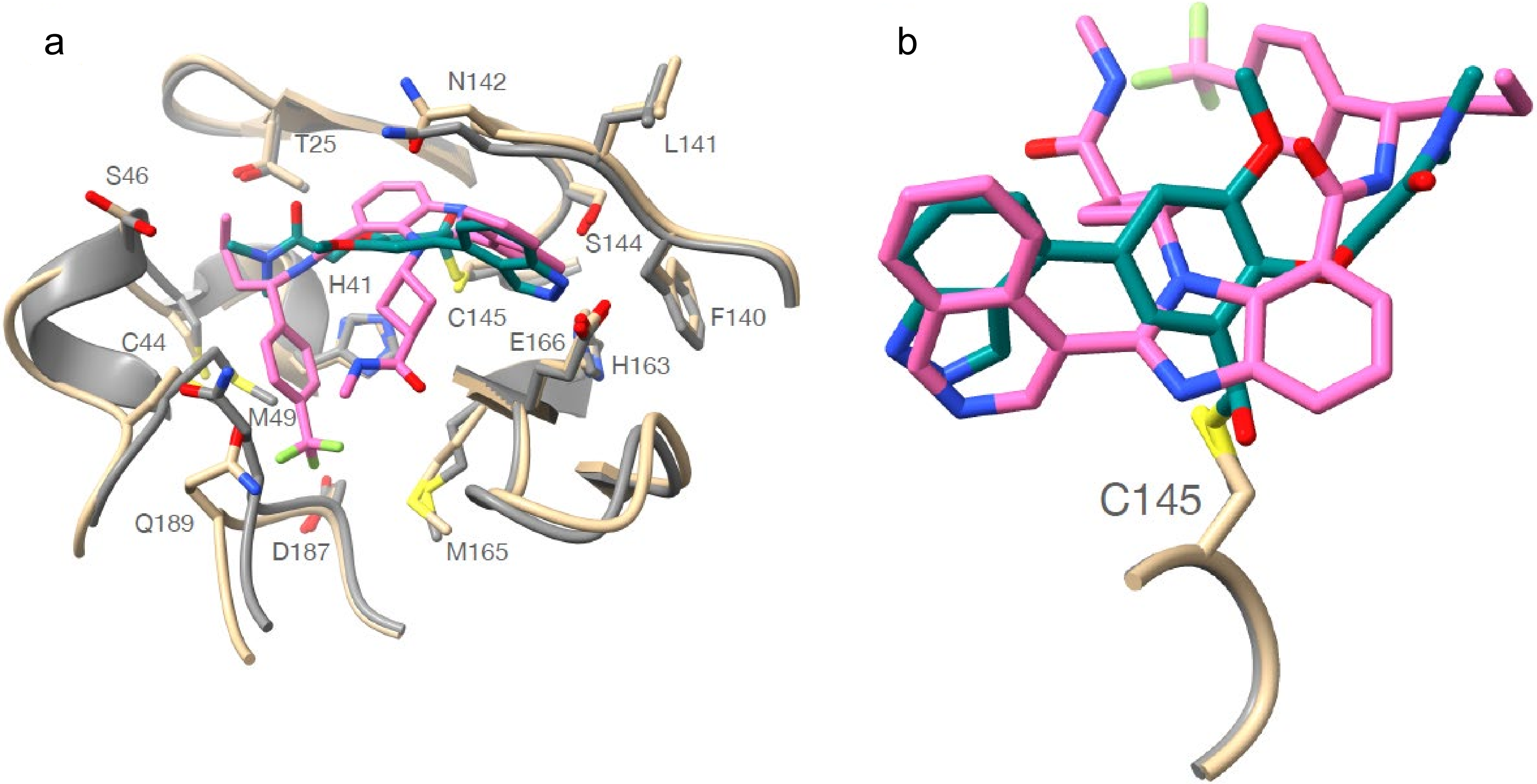

Supplementary Figure 3. Crystal structure of M^{pro} in complex with CDD-1733 (PDB: 7URB) and M^{pro} in complex with CDD-1713 (PDB: 7LTN). a. Structural alignment of M^{pro} (Tan) with CDD-1733 (pink) and Mpro (gray) with the covalent inhibitor CDD-1713 (dark green). Nitrogen atoms are shown in blue and oxygen atoms are red. **b**. Structural alignment of M^{pro} (Tan) with CDD-1733 (pink) and M^{pro} (gray) with the covalent inhibitor CDD-1713 (dark green) showing only Cys-145 and CDD-1733 (pink) and CDD-1713 (dark green). Note the covalent linkage of CDD-1713 with M^{pro}. No atom in CDD-1733 is within 3 angstroms of the sulfur of Cys145.

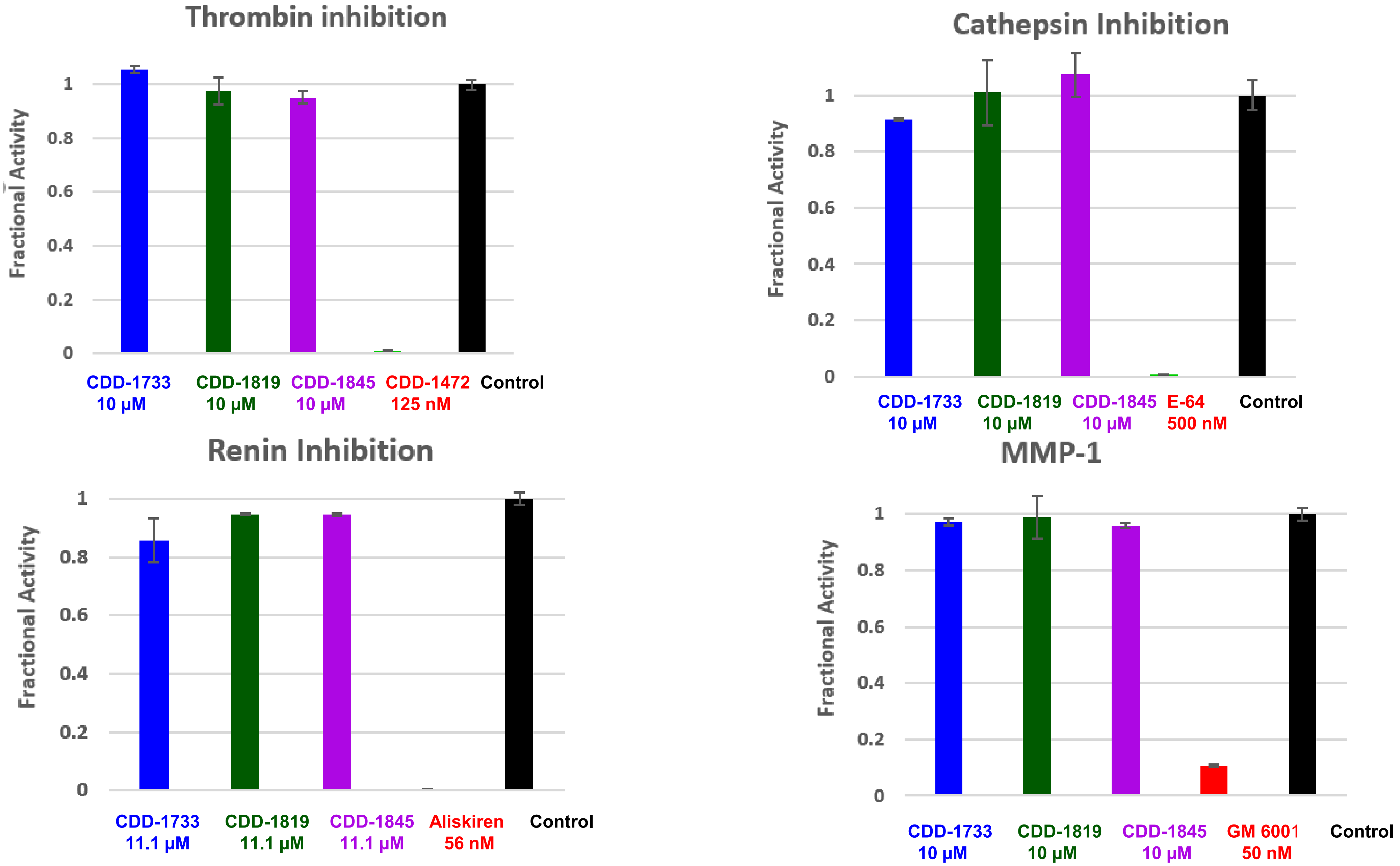

Supplementary Figure 4. Potential off-target inhibition of major proteases, i.e. thrombin (a serine protease), cathepsin B (a cysteine protease like M^{pro}), renin (an aspartic protease), and matrix metallopeptidase 1 (MMP-1), was tested with all active compounds shown here with CDD-1733, CDD-1819, and CDD-1845 and control inhibitors as indicated.

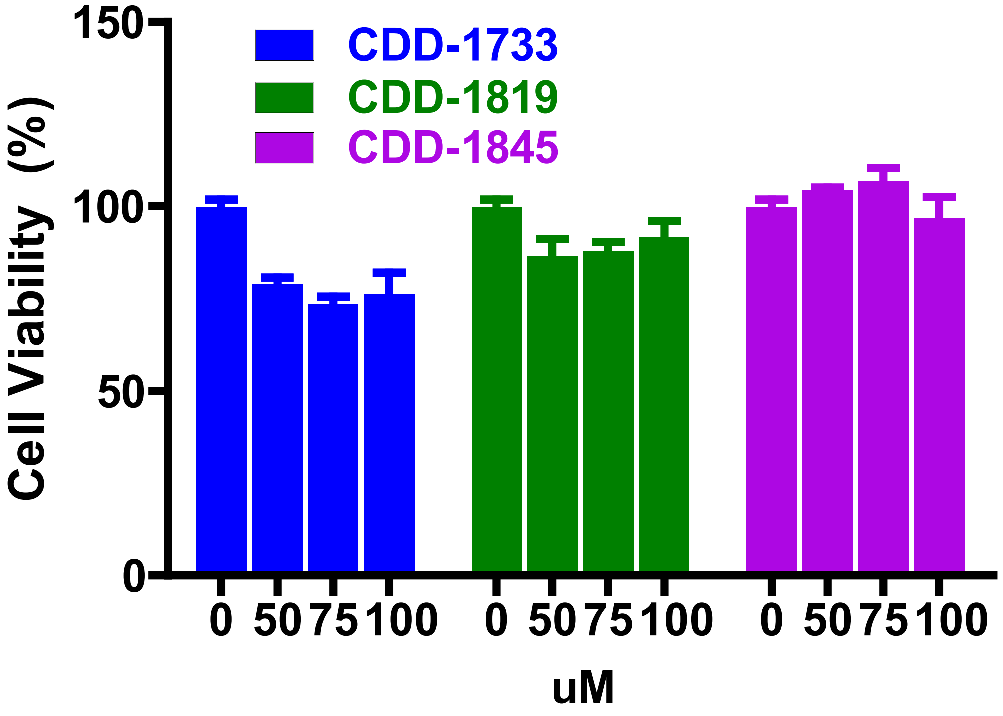

Supplementary Figure 5. HepG2 cell viability after incubated with CDD-1733, CDD-1819, and CDD-1845 for 24 hours; The HepG2 cells were incubated with CDD-1733, CDD-1819, and CDD-1845 (0-100 µM) for 24 hours at 37 °C, induvidually. Cell viability was measured with XTT assay. XTT readings were normalized by the control group (DMSO) to give the normalized viabilities. The IC_{50} values were expressed as “>100 µM” for CDD-1733, CDD-1819, and CDD-1845, as the cell viabilities were larger than 80% in the 100 µM group for these compounds.

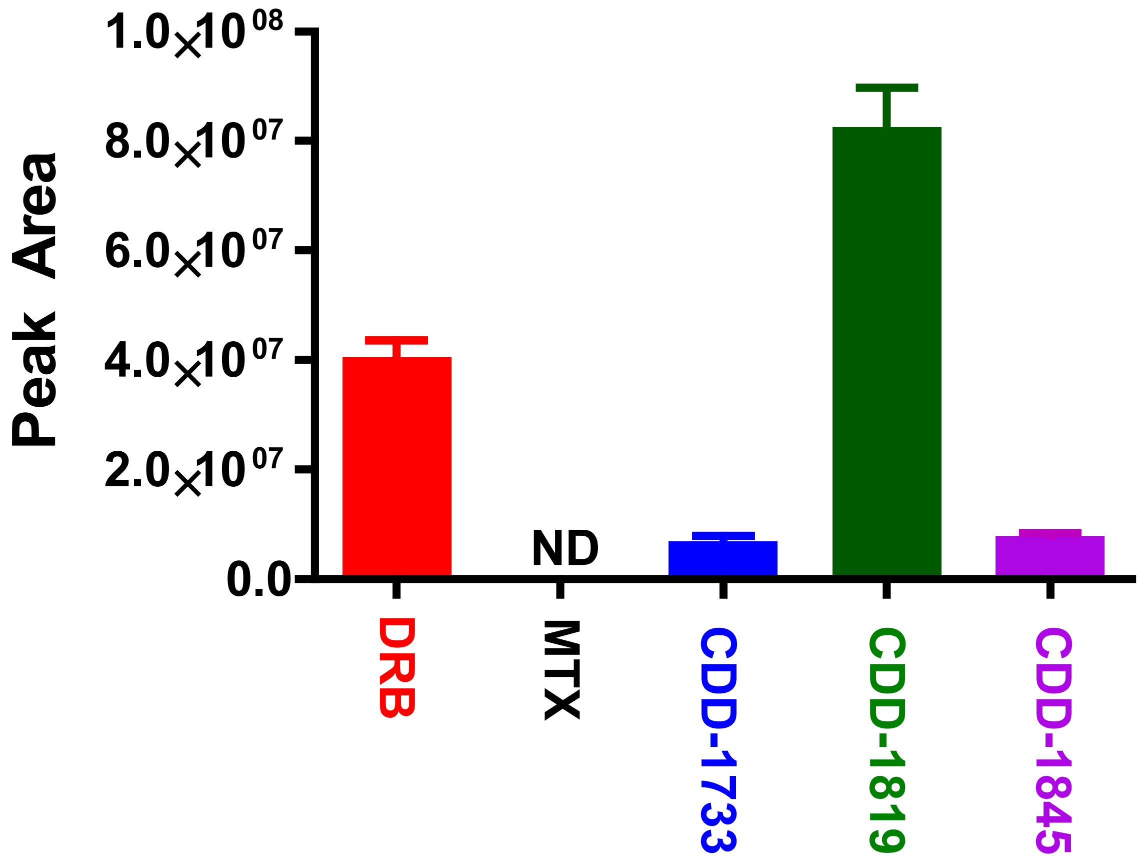

Supplementary Figure 6. HepG2 cell uptake of CDD-1733, CDD-1819, and CDD-1845;The HepG2 cell uptake capacities of CDD-1733, CDD-1819, and CDD-1845 were expressed as the intracellular concentrations (peak areas) of these compounds. Methotrexate (MTX) and doxorubicin (DRB) were used as the negative and positive controls, respectively. The HepG2 cells were incubated with the compounds and controls (final concentration 10 μM) for 2 hours at 37 °C, harvested and homogenized. The intracellular concentrations were measured with UHPLC-Q Exactive Orbitrap MS. ND stands for “not detected”.

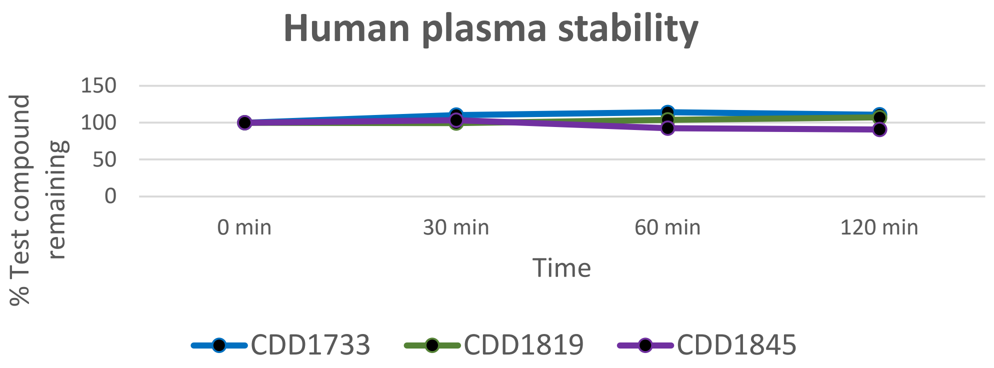

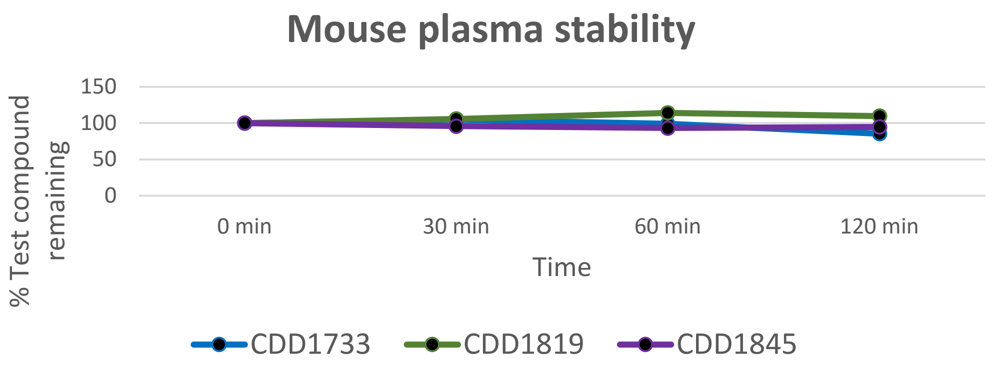

Supplementary Figure 7. Plasma stability of CDD-1733, CDD-1819, and CDD-1845 in human and mouse plasma. CDD-1733, CDD-1819, and CDD-1845 were incubated in human and mouse plasma respectively at a concentration of 10 µM in duplicate (n=2) at 37 °C. The reactions were terminated at time points of 0, 30, 60 and 120 min by adding ice-cold methanol. Following centrifugation, the supernatant was analyzed using UHPLC-Q Exactive Orbitrap MS. The percentage of test compound remaining at the individual time points relative to the 0 min sample was determined

a)

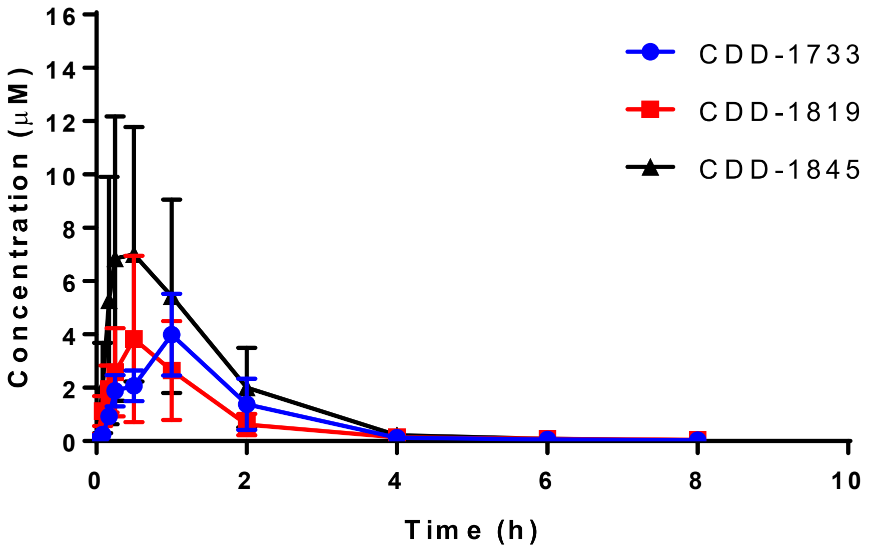

Mouse PK of CDD compounds, 50 mg/kg (po.), n=4, mean ± SD

b)

Noncompartmental parameters of CDD pharmacokinetics (n=4, mean ± SD)

Parameter	Unit	CDD-1733	CDD-1819	CDD-1845
T_{max}	hr	1.00	0.33 ± 0.14	0.33 ± 0.14
C_{max}	μmol/L	4.21 ± 1.80	5.14 ± 2.57	8.93 ± 4.55
$T_{1/2}$	hr	2.15 ± 1.46	1.16 ± 0.12	1.14 ± 0.32
$AUC_{0\text{-}t}$	hr*μmol/L	7.80 ± 3.22	7.09 ± 1.76	14.87 ± 7.79
$AUC_{0\text{-}\infty}$	hr*μmol/L	7.22 ± 3.19	7.18 ± 1.77	14.93 ± 7.85
CL/F	mg/(hr*μmol/L)/kg	7.73 ± 2.75	7.26 ± 1.81	4.02 ± 1.98
$MRT_{0\text{-}t}$	hr	1.48 ± 0.04	1.31 ± 0.12	1.22 ± 0.03
$MRT_{0\text{-}\infty}$	hr	1.72 ± 0.36	1.42 ± 0.16	1.25 ± 0.02

Supplementary Figure 8: a) 4 male mice (8-12 weeks old) received 50 mg/kg of CDD-1733, CDD-1819 or CDD-1845 per oral gavage at the dose of 10 mL/kg. Blood samples were collected at 0, 5, 10, 15, 30 min, 1, 2, 4, 6 and 8 h after dosing. Plasma samples were separated, prepared and analyzed with LC-MS. The compound concentrations were calculated with calibration curves, and the time-concentration profiles are presented as mean ± SD for CDD-1733 (●), CDD-1819 (■) and CDD-1845 (▲). **b**) The pharmacokinetic parameters are calculated with the data from individual mice with noncompartmental analysis, and the data were expressed as mean ± SD (n=4). T_{max}, time to reach the maximum plasma concentration. C_{max}, maximum plasma concentration. $T_{1/2}$, half-life. AUC, area under the curve. CL, clearance. F, bioavailability. MRT, mean residence time.

M^{pro} construct	Inhibitor IC_{50} (nM)				
	CDD-1733	CDD-1819	CDD-1845	CDD-1935	Nirmatrelvir
WT	648	34	98	6550	17
ΔP168	1863	77	185	>10000	95
A173V	1988	115	234	>10000	167
ΔP168/A173V	979	66	219	>10000	833

Supplementary Figure 9: IC_{50} values of inhibitors for mutations conferring resistance to nirmatrelvir using the live cell gain-of-signal assay

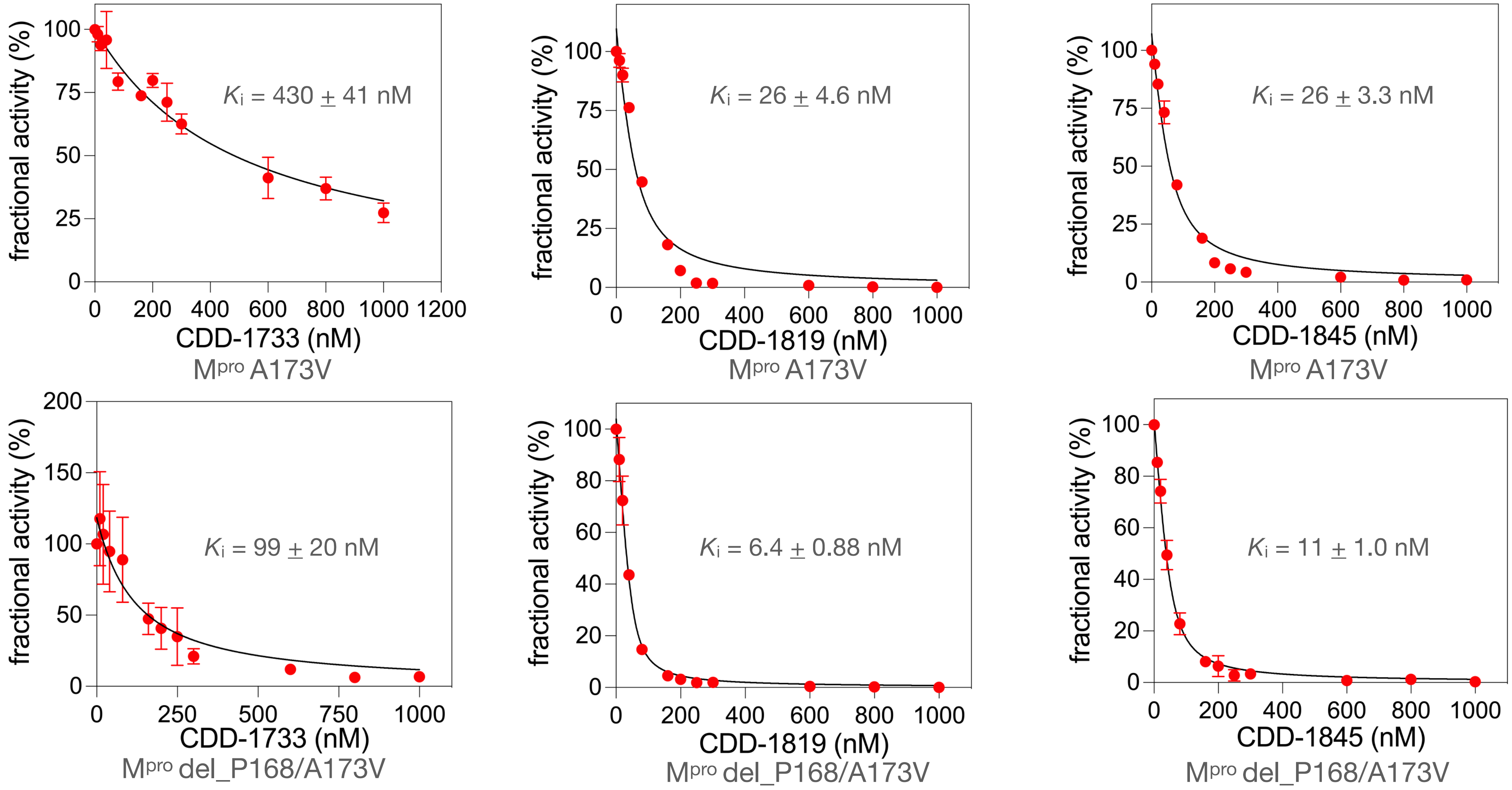

Supplementary Figure 10: Inhibition K_i value determination for CDD-1733, CDD-1819, and CDD-1845 series against M^{pro} variants A173V and ΔP168/A173V. A-L: Concentration-dependent inhibition curves of individual inhibitors. 50 nM of M^{pro} variant enzymes was mixed with increasing concentrations of inhibitors. The remaining activities (red dots) of M^{pro} towards fluorescent peptide were plotted as a function of compound concentrations and K_i values was calculated by fitting the data into Morrison equation with standard error from triplicates.

Supplementary Methods:

General Information of DECL

The general materials, procedures and equipment utilized in this study referenced the related DECL work in our group reported previously.[1-6]

Materials and equipment used for the DNA-encoded chemical libraries. The starting unit dsDNA oligonucleotide with modified phosphates with PEG4 linker and terminal amine (DEC-Tec Starting Unit/DTSU, **S1**) and encoding 5'-phosphorylated oligonucleotides were purchased from LGC Biosearch Technologies. A "spike-in" with 10-mer DNA oligonucleotide featuring a cholesterol tag and terminal amine was purchased from LGC Biosearch Technologies to charge with pooled library to assess chemical reaction progress. T4 DNA ligase in high concentration was obtained from Qiagen Enzymatics. DNAse/RNAse-free ultrapure water from Invitrogen, HPLC-grade acetonitrile from Fisher and high-purity absolute ethanol from Koptec were used to prepare buffer solutions. LC/MS-grade water from Fisher, Optima LC/MS-grade methanol from Fisher, hexafluoroisopropanol (99+% purity) from Sigma-Aldrich and HPLC-grade triethylamine from Fisher were used to prepare LC/MS running solvent. All listed buffer solutions were prepared in-house, including HEPES 10X ligation buffer (300 mM 2-[4-(2-hydroxyethyl)piperazin-1-yl]ethanesulfonic acid, 100 mM adenosine triphosphate, 100 mM dithiothreitol, 10 mM $MgCl_2$, aq. NaCl (5 M), aq. NaOH, and basic borate buffer (250 mM sodium borate/boric acid, pH 10). Chemical building blocks and reagents were purchased from various vendor sources and used without further purification. Building blocks were purchased from a variety of manufacturer and generally prepared in acetonitrile (MeCN), dimethyl sulfoxide (DMSO) or mixed aqueous acetonitrile. The stock solution of building blocks was stored in 2D barcoded tubes from Phenix with septa-caps from Phenix at -80 °C and aliquots were taken for each use. Solutions were transferred using Fisherbrand pipette tips. Polypropylene PCR tubes from Genemate, tubes from Eppendorf, 96-well PCR plates from ThermoFisher and 96-well deep-well plates from USA Scientific were used to perform chemical reactions or DECL production. Large volume of chemical reactions or ethanol precipitations were performed in polypropylene 15-mL, 50-mL centrifuge tubes or 250 mL screw-cap bottles from various manufacturers. Heated reactions were performed on Mastercycler nexus gradient from Eppendorf, benchtop heating blocks from ThermoFisher, or TS-DW deep well plate themoshaker from Grant, or laboratory ovens from Fisher. Solutions were centrifuged in 5424R centrifuge from Eppendorf, or Lynx 4000 centrifuges from ThermoFisher. Optical density measurements were made using a Biophotometer from Eppendorf. A Vanquish

UHPLC system was integrated with LTQ XL ion trap mass spectrometer (ThermoFisher Scientific) for LC/MS analysis of DNA oligonucleotides. DNA ligation was assessed by gel electrophoresis 12 analysis and visualized with Molecular Imager Gel Doc XR system from BIO-RAD after staining in an ethidium bromide solution.

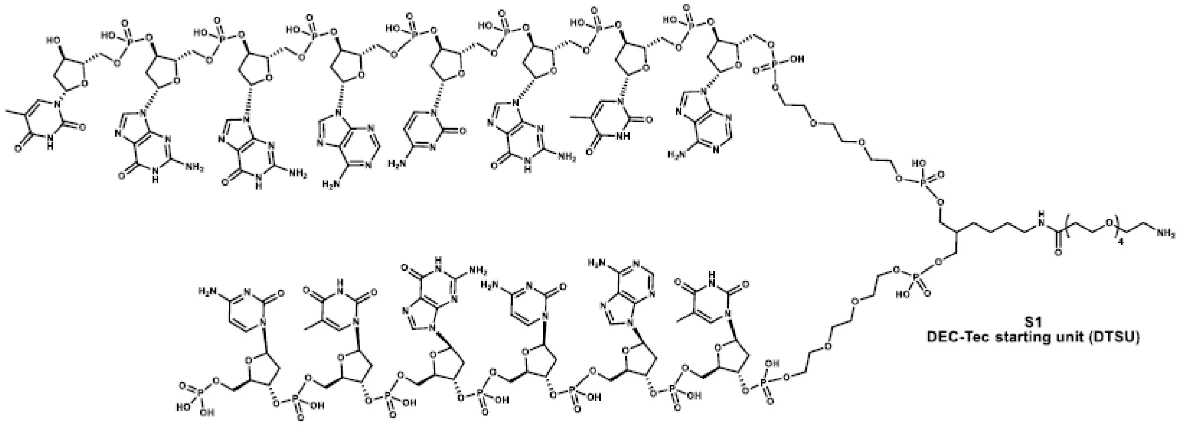

S1
DEC-Tec starting unit (DTSU)

Structure of DTSU **S1** (5'-Phos-CTGCAT-Spacer 9-Amino C7 plus AOP-Spacer 9-ATGCAGGT3').

General procedure for the analysis of DNA oligonucleotides. DNA sample or reaction mixture were diluted to 10 μM final concentration and injected in amounts of 5–10 μL on a Vanquish/LTQ system.

LC/MS Parameters for Thermo Vanquish UHPLC with LTQ Ion Trap MS Instrument

(i) LC settings

Column: Thermo DNAPac RP (2.1 x 50 mm, 4 μm)
Solvent A: 15mM triethylamine (TEA)/100mM hexafluoroisopropanol (HFIP) in water
Solvent B: 15mM TEA/100mM HFIP in 50% methanol
Solvent C: Methanol
Flow rate: 0.65 mL/min
Run time: 2 mins (gradient)
Column temperature: 100 °C (post column cooler at 40 °C)
Eluent: 15 mM TEA/100 mM HFIP in a water/methanol solvent system

(ii) MS settings

Source: ESI in negative mode

Spray voltage: 4100 V

Source heater temperature: 390 °C

Sheath Gas: 28 (instrument units)

Auxiliary Gas: 8 (instrument units)

Sweep Gas: 2 (instrument units)

Capillary temperature: 350 °C

Capillary voltage: -33.0 V

Tube lens: -92.0 V

MS Scan: 500 – 2000 m/z

DNA samples were analyzed on a Thermo Vanquish UHPLC system coupled to an electrospray LTQ ion trap mass spectrometer. An oligonucleotide column (Thermo DNAPac RP, 2.1 x 50 mm, 4 µm) was used with ion-pairing mobile phase for all the separations. Full scan negative-ion mode over the m/z range of 500–2000 was acquired for mass spectra. Data analysis was performed by processing the raw data with the automated biomolecule deconvolution with Promass and reporting software using ZNova novel algorithm to produce artifact-free mass spectra.

General procedures

General procedure for DNA ligation (Preparation of headpiece S2 from DTSU S1)

To DTSU (100 nmol, 100 µL, 1.0 equiv) was added primer foward (5'-ACACTTGCTGGT-3', 105 nmol, 105 µL, 1.05 equiv), primer_reverse (5'-CAGCAAGTGTGA-3', 105 nmol, 105 µL, 1.05 equiv), and nuclease-free water (62.9 µL), followed by the addition of 10X HEPES buffer (41.7 µL) and T4 DNA ligase (2.1 µL) to make the final concentration of DNA 0.24 mM. Incubate the reaction mixture at room temperature overnight. The ligation progress was monitored with LC/MS analysis and gel electrophoresis. Gel electrophoresis was executed with a 12-well 10% TBE acrylamide gel from Invitrogen in 1X TBE buffer prepared in-house. The DNA loading sample was prepared by adding 10 µL of the diluted DNA sample and 2 µL of 6X DNA loading dye to a final concentration of 12 ng/µL. Gels were run at 120 V for 45 min and stained with 0.5 ng/mL ethidium bromide in 1X TBE buffer for 40 min before visualizing. After completion of the ligation, the reaction mixture was purified by ethanol precipitation to yield the headpiece S2 for further use in chemical validation and library production. The ligation provides a final DNA sequence 5' d TAT GAT ACT AAA GTA AGT CAC ACA CAA TTG GAG CAG TCC TGA GTG AAT ACC TGC AT -

Spacer 9-Amino C7- Spacer 9-ATG CAG GTA TTC ACT GAG GAC TGC TCC AAT TGT GTG TGA CTT ACT TTA GTA TCA TAT C 3'.

General procedure for ethanol precipitation and DNA reconstitution. Mixtures from chemical reactions or ligation were added 4% v/v of 5 M NaCl solution and 3 times of the reaction volume of absolute ethanol to crash out the DNA material. The mixture was pipet mixed thoroughly before storing at −20 °C overnight. The slurry was then centrifuged at 4000 x G for an hour, removed the supernatant, and added another portion of pre-chilled 75% ethanol solution to wash the pellet. The pellet was centrifuged at 4000 x G for another hour before decanting the supernatant. The DNA pellet was air-dried and nuclease-free water was added to reconstitute the DNA material. In general, ethanol precipitation was carried out after each chemical reactions or ligation and multiple 75% ethanol wash can be applied while needed.

General procedures for chemical reactions used in library production

Acylation: To a solution of DNA HP (10 nmol, 10 µL, 1.0 mM, 1 equiv) in H_2O was added pH 9.5 borate buffer (4000 nmol, 16 µL, 250 mM, 400 equiv), acid (1000 nmol, 5 µL, 200 mM in MeCN, 100 equiv), and 4-(4,6-dimethoxy-1,3,5-triazin-2-yl)-4-methyl-morpholinium chloride (1000 nmol, 5 µL, 200 mM in H_2O, 100 equiv). The reaction was allowed to sit at room temperature overnight and then quenched by EtOH precipitation.

S_NAr reaction: The reconstituted acylation product (10 nmol, 10 µL, 1.0 mM, 1 equiv) in H_2O was treated with borate buffer (pH 9.5, 2500 nmol, 10 µL, 250mM, 250 equiv) and amine (1000 nmol, 5 µL, 200 mM in MeCN, 100 equiv) at 80 °C overnight. The reaction mixture was cooled to room temperature prior to EtOH precipitation.

Reverse acylation: To a solution of on-DNA carboxylic acid (40.5 µL, 0.9 mM in water) in 72 µL of pH 5.8 MES buffer (250 mM in water, 500 equivalents) was added 18 µL of amine solution (200 mM in MeCN, 100 equivalents), 18 µL of 4-(4,6-dimethoxy-1,3,5-triazin-2-yl)-4-methylmorpholinium chloride (DMTMM, 200 mM in water, 100 equivalents) and 36 µL MeCN to give final 40% v/v MeCN. The reaction was incubated at room temperature for 18 h and then quenched by ethanol precipitation.

Nitro reduction: To a solution of DNA conjugate (10 nmol, 10 µL, 1.0 mM, 1 equiv) in H_2O was added borate buffer (pH 9.5, 2500 nmol, 10 µL, 250 mM, 250 equiv) and viologen (100 nmol, 1 µL, 100 mM in H_2O, 10 equiv), followed by the addition of $Na_2S_2O_4$ (1000 nmol, 5 µL, 200 mM in

H_2O, 100 equiv). The reaction mixture was heated at 80 °C for 20 min. The reaction mixture was cooled to room temperature prior to EtOH precipitation.

Benzimidazole synthesis (aldehyde condensation reaction): For the one-pot synthesis of benzimidazole, the crude reduction product (10 nmol) in borate buffer (pH 9.5, 2500 nmol, 10 μL, 250 mM, 250 equiv) was treated with aldehyde (1000 nmol, 5 μL, 200 mM in MeCN, 100 equiv) at 80 °C overnight. For the step-wise procedure, pH 9.5 borate buffer (2500 nmol, 10 μL, 250 mM, 250 equiv) was added to the reconstituted reduction product (10 nmol, 10 μL, 1.0 mM, 1 equiv). After aldehyde (1000 nmol, 5 μL, 200 mM in MeCN, 100 equiv) was added, the reaction was heated at 80 °C overnight. Both protocols provided the desired product after EtOH precipitation and reconstitution.

Synthesis of a DNA-Encoded Chemical Library (DECL)

Architecture of the main library build and Building block diversity analysis. Similar strategy for the two aspects were adopted from previous reported literature.

Synthetic sequence of the library build.

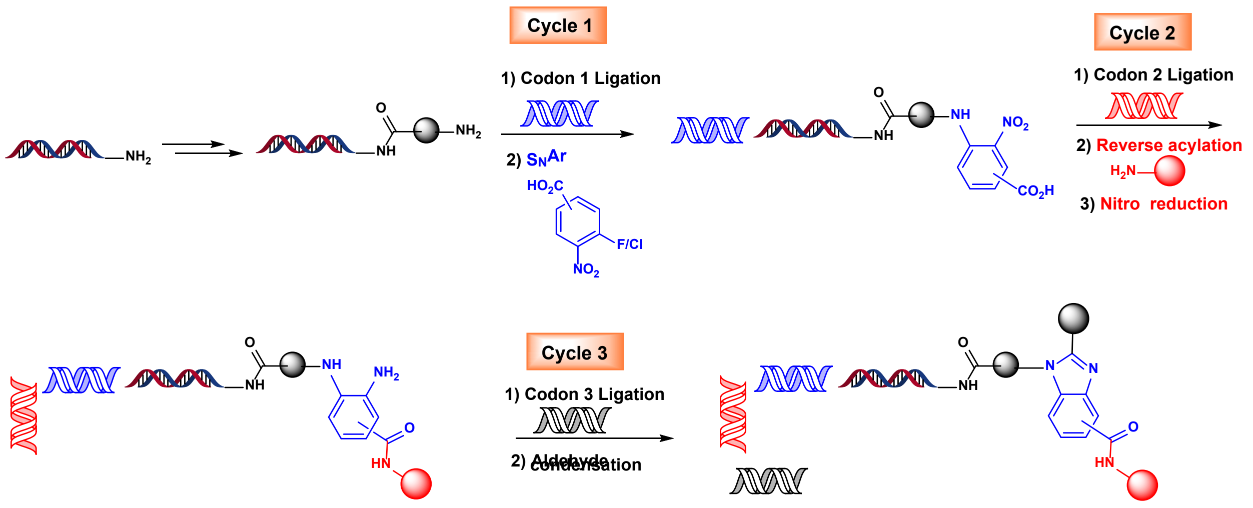

Supplementary Scheme 1: Synthetic scheme of qDOS18_2. Cycle 1 is shown in blue, cycle 2 is shown in red and cycle 3 is shown in black. This library composed of 225 combinations in cycle 1, 1167 building block in cycle 2 and 465 building blocks in cycle 3, which resulted in a library size of 112.09 million.

Preparation of amplifiable DECL samples for further selection experiments. After completion of the main library builds, the entire library material was ligated with a duplexed pair of 12-mer DNA oligonucleotides (library ID) to encode the overall library construct. After ethanol precipitation, the DECL material underwent sequential ligation with DNA oligonucleotides, containing a region to encode selection experiment, a degenerate region as molecular identifier during amplification, and a reverse primer region for post-selection PCR amplification (the purposes/design of these components are discussed in our previous publication).

General Methods for off-DNA Synthesis:

All starting materials and reagents were purchased from commercial sources and used without further purification. Solvents were purchased as either anhydrous grade products in sealed containers or reagent grade and used as received. All reactions were carried out in dry glassware under a nitrogen atmosphere using standard disposable or gastight syringes, disposable or stainless-steel needles, and septa. Stirring was achieved with magnetic stir bars. Flash column chromatography was performed with SiO_2 (230-400 mesh) or by using an automated chromatography instrument with an appropriately sized column. Thin layer chromatography was performed on silica gel 60F_{254} plates (E. Merck). Non-UV active compounds were visualized on TLC using one of the following stains: $KMnO_4$, ninhydrin, and *p*-anisaldehyde. ^{1}H and ^{13}C NMR spectra were recorded on an instrument operating at either 600 MHz or 150 MHz respectively. LCMS data were collected using an HPLC instrument coupled to a low-resolution mass spectrometer with single quadrupole ionization operating in either positive or negative ion mode. The analytical method utilized a C_{18} column (2.1 × 50 mm, 1.8 μm) eluting with a linear gradient of 95%/5% water/CH_3CN (modified with 0.05% formic acid; T = 0 min flow = 0.35 mL/min) to 95%/5% CH_3CN/water (T = 3.5 min flow = 0.5 mL/min) then 95%/5% CH_3CN/water to T = 5min (0.5 mL/min). Peak detection was done at 254 nm and 230 nm for UV-active compounds. High-resolution mass spectrometry (HRMS) spectra were obtained on a Thermo Scientific Q Exactive hybrid quadrupole-Orbitrap mass spectrometer equipped with a HESI source and using lock masses for correction. Samples were introduced into the HRMS via reverse-phase HPLC on an Accucore Vanquish C18+ column (2.1 × 100 mm, 1.5 μm) eluting with a linear gradient of 95%/5% water/acetonitrile (modified with 0.1% formic acid) to 10%/90% water/acetonitrile over 8 min.

Experimental procedures and NMR data:

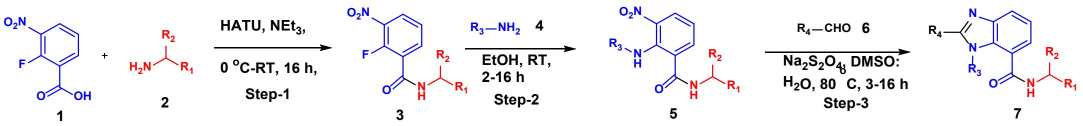

Supplementary Scheme 2: General off-DNA synthetic route for qDOS18_2 library hits, and SAR analogs

General procedure-step-1: Into a round bottom flask equipped with a magnetic stir bar and septum under nitrogen, the carboxylic acid compound (**1 equiv.**) was dissolved in DMF, amine **2** (**1.1 equiv.**) was added followed by HATU (**1.2 equiv.**) and NEt_3 (**1.5 equiv.**) at 0 °C and allowed to room temperature. The reaction was allowed to stir for 16 h, after which time TLC and LCMS indicated complete consumption of starting material. The reaction was worked up by diluting with ethyl acetate and washed with sat. aq $NaHCO_3$ and brine. The organic phase was collected and dried over anhydrous Na_2SO_4, filtered, and concentrated under reduced pressure to give the crude product which was purified by silica gel chromatography (ethyl acetate/ hexanes) to yield the desired product **3**.

General procedure-step-2: Into a round bottom flask equipped with a magnetic stir bar and septum under nitrogen, the compound **3** (**1 equiv.**) was dissolved in ethanol, amine **4** (**1.2 equiv.**) and NEt_3 (**1.5 equiv.**) was added at room temperature. The reaction was allowed to stir for 16 h, after which time TLC and LCMS indicated complete consumption of starting material. Then the solvent was removed under reduced pressure to give the crude residue which was purified by silica gel chromatography (ethyl acetate/ hexanes) to yield the desired product **5**.

General procedure-step-3: Into a round bottom flask equipped with a magnetic stir bar and septum under nitrogen, the compound **5** (**1 equiv.**) was dissolved in DMSO: water (5:1), aldehyde **6** (**1.3 equiv.**) was added followed by $Na_2S_2O_4$ (**6.0 equiv.**) at room temperature. The reaction was allowed to stir for 3-16 h at 80 °C, after which time TLC and LCMS indicated complete consumption of starting material. The reaction was worked up by diluting with ethyl acetate and washed with sat. aq $NaHCO_3$ and brine. The organic phase was collected and dried over anhydrous Na_2SO_4, filtered, and concentrated under reduced pressure to give the crude product which was purified by silica gel chromatography (water/ACN) to yield the desired product **7**.

Synthesized by following general procedure-Step-1:

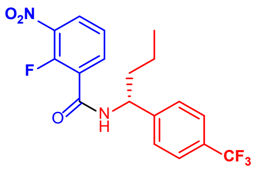

***(R)*-2-fluoro-3-nitro-N-(1-(4-(trifluoromethyl)phenyl)butyl)benzamide**: Molecular Formula: $C_{18}H_{16}F_4N_2O_3$; yield 72%; **^{1}H NMR (600 MHz, $CDCl_3$) δ ppm** 8.32 (t, *J* = 7.0 Hz, 1H), 8.17 (dd, *J* = 11.0, 4.1 Hz, 1H), 7.63 (d, *J* = 8.1 Hz, 2H), 7.49 (d, *J* = 8.1 Hz, 2H), 7.42 (t, *J* = 8.0 Hz, 1H), 7.02 – 6.95 (m, 1H), 5.22 (q, *J* = 7.1 Hz, 1H), 1.97 – 1.83 (m, 2H), 1.52 – 1.43 (m, 1H), 1.43 – 1.35 (m, 1H), 0.99 (t, *J* = 7.4 Hz, 3H). **^{13}C NMR (150 MHz, $CDCl_3$) δ ppm** 160.8, 160.8, 154.3, 152.6, 146.1, 138.1, 138.1, 137.7, 137.7, 130.2, 130.0, 129.8, 129.6, 129.2, 127.0, 125.9, 125.9, 125.9, 125.9, 125.1, 125.0, 124.1, 124.0, 123.2, 54.3, 38.5, 19.5, 13.8. **LCMS** m/z calcd for (M + H)$^+$ 385.3, found 385.2.

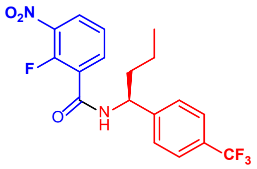

(*S*)-2-fluoro-3-nitro-N-(1-(4-(trifluoromethyl)phenyl)butyl)benzamide: Molecular Formula: $C_{18}H_{16}F_4N_2O_3$; yield 75%; **^{1}H NMR (600 MHz, $CDCl_3$) δ ppm** 8.34-8.33 (m, 1H), 8.17 (td, *J* = 8.1, 1.9 Hz, 1H), 7.62 (d, *J* = 8.1 Hz, 2H), 7.47 (d, *J* = 8.2 Hz, 2H), 7.42 (t, *J* = 7.8 Hz, 1H), 6.93 – 6.88 (m, 1H), 5.22 – 5.18 (m, 1H), 1.95 – 1.83 (m, 2H), 1.48 (d, *J* = 7.4 Hz, 1H), 1.43-1.36 (m, 1H), 0.98 (t, *J* = 7.4 Hz, 3H). **^{13}C NMR (150 MHz, $CDCl_3$) δ ppm** 160.8, 160.8, 154.3, 152.6, 146.1, 138.1, 138.1, 137.7, 137.7, 130.2, 130.0, 129.8, 129.6, 129.2, 127.0, 125.9, 125.9, 125.9, 125.9, 125.1, 125.0, 124.1, 124.0, 123.2, 54.3, 38.5, 19.5, 13.8. **LCMS** m/z calcd for (M + H)$^+$ 385.3, found 385.2.

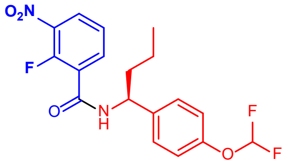

***(S)*-N-(1-(4-(difluoromethoxy)phenyl)butyl)-2-fluoro-3-nitrobenzamide:** Molecular Formula: $C_{18}H_{17}F_3N_2O_4$; yield 60%; **^{1}H NMR (600 MHz, CD_3OD) δ ppm** 9.11 (d, *J* = 8.0 Hz, 1H), 8.20 – 8.15 (m, 1H), 7.84 (dd, *J* = 9.6, 3.7 Hz, 1H), 7.43 (d, *J* = 8.5 Hz, 3H), 7.14 (d, *J* = 8.6 Hz, 2H), 6.81-6.80 (m, 1H), 5.17 – 5.05 (m, 1H), 1.93 – 1.86 (m, 1H), 1.85 – 1.73 (m, 1H), 1.56 – 1.48 (m, 1H), 1.48 – 1.37 (m, 1H), 1.00 (t, *J* = 7.4 Hz, 3H). **^{13}C NMR (150 MHz, CD_3OD) δ ppm** 164.7, 164.6, 154.5, 152.7, 151.9, 151.9, 151.9, 141.2, 139.2, 139.2, 136.1, 136.1, 129.2, 128.9, 128.5, 128.5, 128.4, 128.4, 125.9, 125.9, 120.3, 119.5, 117.8, 116.1, 55.0, 54.9, 39.4, 20.7, 14.0. **LCMS** m/z calcd for (M + H)$^+$ 383.3, found 383.2.

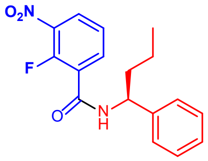

***(S)*-2-fluoro-3-nitro-N-(1-phenylbutyl)benzamide:** Molecular Formula:$C_{17}H_{17}FN_2O_3$; yield 62%; **^{1}H NMR (600 MHz, CDCl$_3$) δ ppm** 8.11 (dd, *J* = 10.1, 3.8 Hz, 1H), 8.00 (t, *J* = 6.8 Hz, 1H), 7.25 – 7.24 (m, 5H), 7.17 (dd, *J* = 8.6, 4.3 Hz, 1H), 6.96 (t, *J* = 7.9 Hz, 1H), 5.12 – 5.01 (m, 1H), 1.88 – 1.72 (m, 2H), 1.37 – 1.22 (m, 2H), 0.86 (t, *J* = 7.4 Hz, 3H). **^{13}C NMR (150 MHz, CDCl$_3$) δ ppm** 160.7, 154.0, 152.3, 141.9, 137.9, 137.9, 137.3, 137.3, 128.8, 128.7, 127.6, 126.6, 124.8, 124.7, 54.4, 38.5, 19.5, 13.8. **LCMS** m/z calcd for (M + H)$^+$ 317.3, found 317.2.

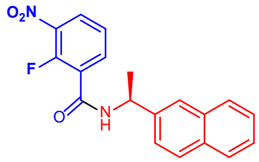

(*S*)-2-fluoro-N-(1-(naphthalen-2-yl)ethyl)-3-nitrobenzamide: Molecular Formula: $C_{19}H_{15}FN_2O_3$; yield 82%; **^{1}H NMR (600 MHz, CDCl$_3$) δ ppm** 8.37 (t, *J* = 7.2 Hz, 1H), 8.15 (t, *J* = 7.6 Hz, 1H), 7.86 (dd, *J* = 13.9, 8.9 Hz, 4H), 7.52 – 7.45 (m, 3H), 7.41 (t, *J* = 8.0 Hz, 1H), 6.96 (s, 1H), 5.55 – 5.48 (m, 1H), 1.72 (d, *J* = 6.9 Hz, 3H). **^{13}C NMR (150 MHz, CDCl$_3$) δ ppm** 160.5, 154.3, 152.6, 139.9, 138.2, 137.7, 137.6, 133.5, 133.0, 129.0, 128.9, 128.1, 127.8, 126.5, 126.2, 124.9, 124.9, 124.9, 124.5, 124.4, 77.4, 77.2, 76.9, 50.3, 22.0. **LCMS** m/z calcd for (M + H)$^+$ 339.3, found 339.2.

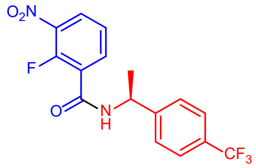

***(S)*-2-fluoro-3-nitro-N-(1-(4-(trifluoromethyl)phenyl)ethyl)benzamide:** Molecular Formula: $C_{16}H_{12}F_4N_2O_3$; yield 82%; **^{1}H NMR (600 MHz, CDCl$_3$) δ ppm** 8.35-8.35 (m, 1H), 8.17 (td, *J* = 8.1, 1.8 Hz, 1H), 7.63 (d, *J* = 8.2 Hz, 2H), 7.50 (d, *J* = 8.2 Hz, 2H), 7.45 – 7.39 (m, 1H), 6.96 – 6.88 (m, 1H), 5.36 (d, *J* = 6.9 Hz, 1H), 1.63 (d, *J* = 7.0 Hz, 3H). **^{13}C NMR (150 MHz, CDCl$_3$) δ ppm** 160.7, 160.7, 154.2, 152.5, 146.8, 138.1, 138.0, 137.5, 137.5, 130.2, 130.0, 129.8, 129.6, 129.2, 129.1, 126.5, 125.9, 125.9, 125.9, 125.9, 125.0, 125.0, 124.2, 124.1, 123.2, 49.9, 22.1. **LCMS** m/z calcd for (M + H)$^+$ 357.2, found 357.2.

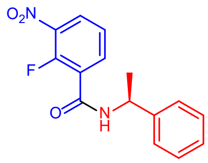

(S)-2-fluoro-3-nitro-N-(1-phenylethyl)benzamide: Molecular Formula: $C_{15}H_{13}FN_2O_3$; yield 64%;**^{1}H NMR (600 MHz, CDCl$_3$) δ ppm** 8.36-8.35 (m, 1H), 8.14 (td, *J* = 8.1, 1.8 Hz, 1H), 7.43 – 7.34 (m, 5H), 7.30 (td, *J* = 6.1, 3.3 Hz, 1H), 6.88 (s, 1H), 5.35-5.34 (m, 1H), 1.62 (d, *J* = 6.9 Hz, 3H). **^{13}C NMR (150 MHz, CDCl$_3$) δ ppm** 160.4, 160.4, 154.3, 152.5, 142.6, 137.7, 137.7, 129.0, 127.9, 126.3, 124.9, 124.9, 124.5, 124.4, 50.2, 22.1. **LCMS** m/z calcd for (M + H)$^+$ 289.2, found 289.2.

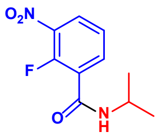

2-fluoro-N-isopropyl-3-nitrobenzamide: Molecular Formula: $C_{10}H_{11}FN_2O_3$; yield 47%;**^{1}H NMR (600 MHz, CDCl$_3$) δ ppm** 8.37 – 8.27 (m, 1H), 8.16 – 8.08 (m, 1H), 7.40 (t, *J* = 8.0 Hz, 1H), 6.45 (s, 1H), 4.30 (dd, *J* = 12.5, 6.5 Hz, 1H), 1.28 (d, *J* = 6.6 Hz, 6H). **^{13}C NMR (150 MHz, CDCl$_3$) δ ppm** 160.4, 160.4, 154.2, 152.4, 137.5, 137.5, 128.8, 128.8, 124.9, 124.8, 42.7, 22.7. **LCMS** m/z calcd for $(M + H)^+$ 227.2, found 227.2.

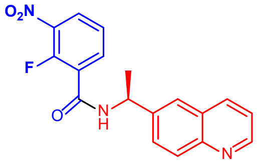

(S)-2-fluoro-3-nitro-N-(1-(quinolin-6-yl)ethyl)benzamide: Molecular Formula: $C_{18}H_{14}FN_3O_3$; yield 70%;**^{1}H NMR (600 MHz, CDCl$_3$) δ ppm** 8.91 (dd, *J* = 4.1, 1.4 Hz, 1H), 8.37 – 8.33 (m, 1H), 8.18 – 8.14 (m, 2H), 8.12 (d, *J* = 8.7 Hz, 1H), 7.82 (d, *J* = 1.5 Hz, 1H), 7.74 (dd, *J* = 8.7, 2.0 Hz, 1H), 7.44 – 7.40 (m, 2H), 7.06 – 6.99 (m, 1H), 5.53 (d, *J* = 6.9 Hz, 1H), 1.73 (d, *J* = 7.0 Hz, 3H). **^{13}C NMR (150 MHz, CDCl$_3$) δ ppm** 160.6, 154.4, 152.6, 150.7, 148.0, 140.9, 137.7, 137.7, 136.2, 130.5, 129.2, 128.3, 128.0, 125.0, 125.0, 124.8, 124.3, 124.2, 121.7, 50.1, 22.1. **LCMS** m/z calcd for $(M + H)^+$ 340.3, found 340.2.

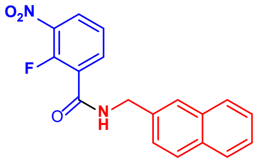

2-fluoro-N-(naphthalen-2-ylmethyl)-3-nitrobenzamide: Molecular Formula: $C_{18}H_{13}FN_2O_3$; yield 68%;**^{1}H NMR (600 MHz, CDCl$_3$) δ ppm** 8.44 – 8.40 (m, 1H), 8.18 – 8.14 (m, 1H), 7.88 – 7.79 (m, 4H), 7.52 – 7.46 (m, 3H), 7.43 (t, *J* = 8.0 Hz, 1H), 7.01 (s, 1H), 4.85 (d, *J* = 5.6 Hz, 2H). **^{13}C NMR (150 MHz, CDCl$_3$) δ ppm** 161.4, 154.3, 152.6, 137.7, 137.7, 134.8, 133.5, 133.1, 129.2, 129.0, 127.9, 127.9, 126.8, 126.6, 126.3, 125.9, 125.0, 125.0, 124.3, 124.2, 44.8. **LCMS** m/z calcd for $(M + H)^+$ 325.3, found 325.2.

Synthesized by following general procedure-Step-2:

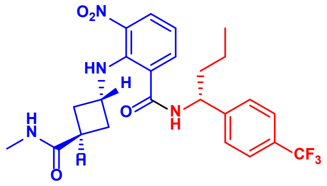

2-(((1r,3R)-3-(methylcarbamoyl)cyclobutyl)amino)-3-nitro-N-((R)-1-(4-(trifluoro methyl) phenyl)butyl)benzamide: Molecular Formula:$C_{24}H_{27}F_3N_4O_4$; yield 78%; **^{1}H NMR (600 MHz, DMSO-d$_6$) δ ppm** 9.11 (d, *J* = 7.4 Hz, 1H), 8.23 (d, *J* = 7.0 Hz, 1H), 8.01 (d, *J* = 8.2 Hz, 1H), 7.71 (d, *J* = 7.5 Hz, 2H), 7.61 (d, *J* = 6.1 Hz, 4H), 6.80 (t, *J* = 7.6 Hz, 1H), 5.01 (d, *J* = 7.0 Hz, 1H), 3.96 (dd, *J* = 13.6, 6.7 Hz, 1H), 2.83 (s, 1H), 2.56 (d, *J* = 3.6 Hz, 3H), 2.34 (s, 2H), 2.05 – 1.96 (m, 2H), 1.88 (d, *J* = 8.6 Hz, 1H), 1.71 (s, 1H), 1.37 (s, 1H), 1.26-1.25 (m, 1H), 0.91 (t, *J* = 6.9 Hz, 3H). **^{13}C NMR (150 MHz, DMSO-d$_6$) δ ppm** 174.8, 167.6, 148.8, 142.6, 136.1, 135.6, 128.6, 127.8, 125.7, 125.7, 124.8, 116.0, 53.4, 49.7, 38.1, 34.6, 34.5, 33.2, 26.0, 19.7, 14.1. **LCMS** m/z calcd for $(M + H)^+$ 393.4, found 393.4.

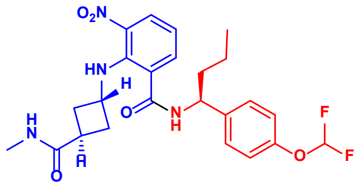

N-((S)-1-(4-(difluoromethoxy)phenyl)butyl)-2-(((1r,3S)-3-(methylcarbamoyl)cyclo butyl)amino)-3-nitrobenzamide: Molecular Formula:$C_{24}H_{28}F_2N_4O_5$; yield 67%;**^{1}H NMR (600 MHz, CDCl$_3$) δ ppm** 9.26 (s, 1H), 8.62 (s, 1H), 8.00 (d, *J* = 7.6 Hz, 1H), 7.89 (d, *J* = 7.7 Hz, 1H), 7.70 (s, 1H), 7.63 (t, *J* = 7.0 Hz, 1H), 7.41 (d, *J* = 6.9 Hz, 1H), 7.35 (d, *J* = 8.0 Hz, 2H), 7.27 (d, *J* = 6.8 Hz, 1H), 7.03 (d, *J* = 7.9 Hz, 2H), 6.40 (d, *J* = 73.8 Hz, 1H), 5.12 (dd, *J* = 14.9, 7.4 Hz, 2H), 2.61 (d, *J* = 2.5 Hz, 3H), 2.20 (s, 1H), 2.04 – 1.94 (m, 3H), 1.83–1.81 (m, 3H), 1.42–1.41 (d, *J* = 7.6 Hz, 1H), 1.36 – 1.26 (m, 1H), 0.93 (t, *J* = 7.3 Hz, 3H). **^{13}C NMR (150 MHz, CDCl$_3$) δ ppm** 174.4, 167.0, 166.9, 150.5, 142.2, 139.4, 128.7, 128.3, 126.3, 126.2, 119.9, 117.7, 117.1, 115.9, 114.2, 53.5, 53.4, 53.3, 51.9, 48.6, 38.2, 35.4, 35.4, 30.9, 30.7, 19.7, 13.8. **LCMS** m/z calcd for $(M + H)^+$ 491.5, found 491.5.

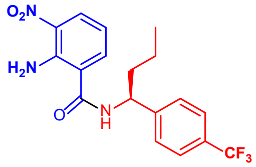

(S)-2-amino-3-nitro-N-(1-(4-(trifluoromethyl)phenyl)butyl)benzamide: Molecular Formula: $C_{18}H_{18}F_3N_3O_3$; yield 50%; **^{1}H NMR (600 MHz, CDCl$_3$) δ ppm** 8.30 (dd, *J* = 8.5, 1.4 Hz, 1H), 8.11 (s, 2H), 7.63 (d, *J* = 8.3 Hz, 3H), 7.46 (d, *J* = 8.1 Hz, 2H), 6.64 (dd, *J* = 8.5, 7.6 Hz, 1H), 6.25 (d, *J* = 7.3 Hz, 1H), 5.14 (q, *J* = 7.4 Hz, 1H), 1.94 – 1.83 (m, 2H), 1.47 – 1.40 (m, 1H), 1.40 – 1.32 (m, 1H), 0.98 (t, *J* = 7.4 Hz, 3H). **^{13}C NMR (150 MHz, CDCl$_3$) δ ppm** 167.6, 146.3, 146.2, 134.1, 133.4, 130.4, 130.1, 129.9, 126.9, 126.0, 126.0, 125.1, 123.2, 119.9, 114.1, 53.7, 38.4, 19.6, 13.9. **LCMS** m/z calcd for $(M + H)^+$ 382.3, found 382.3.

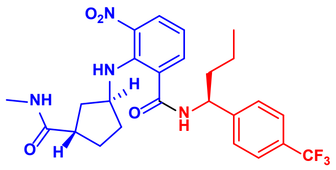

2-(((1S,3S)-3-(methylcarbamoyl)cyclopentyl)amino)-3-nitro-N-((S)-1-(4-(trifluoro methyl)phenyl)butyl)benzamide: Molecular Formula:$C_{25}H_{29}F_3N_4O_4$; yield 98%; **^{1}H NMR(600 MHz, CDCl$_3$) δ ppm** 8.13 (dd, *J* = 8.4, 1.6 Hz, 1H), 7.80 (dd, *J* = 7.5, 1.6 Hz, 1H), 7.64 (d, *J* = 8.1 Hz, 2H), 7.48 (d, *J* = 8.1 Hz, 2H), 7.19 (dd, *J* = 17.0, 8.7 Hz, 2H), 6.95 – 6.91 (m, 1H), 5.43 (d, *J* = 2.5 Hz, 1H), 5.20 (q, *J* = 7.6 Hz, 1H), 3.83 (dt, *J* = 14.4, 7.2 Hz, 1H), 2.78 (d, *J* = 4.8 Hz, 3H), 2.65 – 2.55 (m, 1H), 2.09 – 1.99 (m, 1H), 2.00 – 1.80 (m, 3H), 1.66 (dt, *J* = 22.7, 15.2 Hz, 3H), 1.43 – 1.24 (m, 4H), 0.96 (t, *J* = 7.4 Hz, 3H). **^{13}C NMR (150 MHz, CDCl$_3$) δ ppm** 176.0, 167.4, 146.1, 142.7, 137.8, 136.2, 129.8, 129.6, 128.5, 127.3, 127.2, 125.6, 125.6, 124.9, 123.1, 117.7, 117.5, 58.1, 53.6, 49.4, 49.3, 49.1, 49.0, 42.9, 37.7, 37.1, 33.1, 28.0, 19.5, 13.6. **LCMS** m/z calcd for $(M + H)^+$ 507.5, found 507.4.

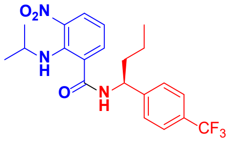

***(S)*-2-(isopropylamino)-3-nitro-N-(1-(4-(trifluoromethyl)phenyl)butyl)benzamide:** Molecular Formula:$C_{21}H_{24}F_3N_3O_3$; yield 90%; **^{1}H NMR (600 MHz, CDCl$_3$) δ ppm** 8.13 (dd, *J* = 8.3, 1.7 Hz, 1H), 7.90 (dd, *J* = 7.5, 1.7 Hz, 1H), 7.62 (d, *J* = 8.1 Hz, 3H), 7.48 (d, *J* = 8.1 Hz, 2H), 6.98 (t, *J* = 7.9 Hz, 1H), 5.19 (q, *J* = 7.6 Hz, 1H), 3.34 (dt, *J* = 12.6, 6.3 Hz, 1H), 1.98 – 1.90 (m, 1H), 1.88 – 1.80 (m, 1H), 1.41–1.35 (m, 1H), 1.34 – 1.27 (m, 1H), 1.03 (d, *J* = 6.3 Hz, 3H), 0.96 (t, *J* = 7.4 Hz, 3H), 0.85 (d, *J* = 6.3 Hz, 3H). **^{13}C NMR (150 MHz, CDCl$_3$) δ ppm** 166.1, 146.0, 142.8, 140.9, 137.0, 130.4, 130.1, 129.9, 129.7, 129.0, 128.6, 127.4, 125.9, 125.9, 125.9, 125.9, 125.0, 123.2, 120.0, 53.7, 50.9, 38.0, 23.2, 23.1, 19.6, 13.9. **LCMS** m/z calcd for $(M + H)^+$ 424.4, found 424.3

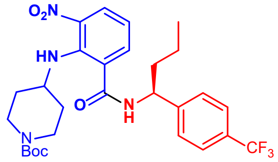

tert-butyl (S)-4-((2-nitro-6-((1-(4-(trifluoromethyl) phenyl)butyl)carbamoyl)phenyl) amino) piperidine-1-carboxylate: Molecular Formula:$C_{28}H_{35}F_3N_4O_5$; yield 95%; **^{1}H NMR (600 MHz, CDCl$_3$) δ ppm** 8.13 (dd, *J* = 8.3, 1.7 Hz, 1H), 7.87 (dd, *J* = 7.5, 1.7 Hz, 1H), 7.64 (d, *J* = 8.1 Hz, 2H), 7.50 (d, *J* = 8.1 Hz, 2H), 7.41 (d, *J* = 8.2 Hz, 1H), 7.03 – 6.97 (m, 1H), 5.22 (dd, *J* = 15.0, 7.4 Hz, 1H), 3.86-3.84 (m, 2H), 3.12 – 3.03 (m, 1H), 2.52 (t, *J* = 12.0 Hz, 1H), 2.36 – 2.28 (m, 1H), 1.92-1.90 (m, 1H), 1.88 – 1.80 (m, 1H), 1.71 (d, *J* = 11.0 Hz, 1H), 1.41 (s, 9H), 1.39 – 1.35 (m, 1H), 1.35 – 1.28 (m, 2H), 1.20 – 1.09 (m, 2H), 0.95 (t, *J* = 7.4 Hz, 3H). **^{13}C NMR (150 MHz, CDCl$_3$) δ ppm** 166.2, 154.5, 145.9, 142.0, 140.6, 136.9, 128.8, 128.7, 127.4, 126.1, 126.1, 120.1, 79.9, 60.5, 55.8, 53.6, 38.1, 32.7, 28.5, 19.7, 13.9. **LCMS** m/z calcd for $(M + H)^+$ 565.6, found 565.5.

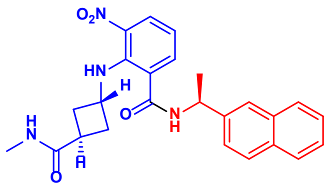

2-(((1r,3S)-3-(methylcarbamoyl)cyclobutyl)amino)-N-((S)-1-(naphthalen-2-yl)ethyl)-3-nitro benzamide: Molecular Formula:$C_{25}H_{26}N_4O_4$; yield 64%; **^{1}H NMR (600 MHz, DMSO-d$_6$) δ ppm** 9.15 (d, *J* = 7.7 Hz, 1H), 8.30 (d, *J* = 7.4 Hz, 1H), 8.01 (dd, *J* = 8.4, 1.3 Hz, 1H), 7.96 – 7.87 (m, 4H), 7.68 (d, *J* = 4.5 Hz, 1H), 7.64 – 7.56 (m, 2H), 7.54 – 7.45 (m, 2H), 6.79 (t, *J* = 7.9 Hz, 1H), 5.32 – 5.23 (m, 1H), 4.13 – 4.04 (m, 1H), 2.86 – 2.78 (m, 1H), 2.56 (d, *J* = 4.6 Hz, 3H), 2.47 – 2.35 (m, 2H), 2.07-2.06 (m, 2H), 1.58 (d, *J* = 7.0 Hz, 3H). **^{13}C NMR (150 MHz, DMSO-d$_6$) δ ppm** 174.5, 174.1, 166.8, 142.2, 141.9, 135.4, 135.3, 132.9, 132.1, 128.0, 128.0, 127.8, 127.5, 126.1, 125.7, 124.9, 124.8, 124.2, 115.5, 49.2, 48.8, 34.5, 34.2, 32.8, 25.6, 21.8. **LCMS** m/z calcd for $(M + H)^+$ 447.5 found 447.4.

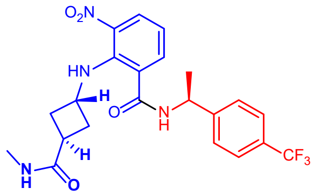

2-(((1r,3S)-3-(methylcarbamoyl)cyclobutyl)amino)-3-nitro-N-((S)-1-(4-(trifluoro methyl) phenyl)ethyl)benzamide: Molecular Formula:$C_{22}H_{23}F_3N_4O_4$; yield 92%;**^{1}H NMR (600 MHz, DMSO-d$_6$) δ ppm** 9.15 (d, *J* = 7.5 Hz, 1H), 8.26 (d, *J* = 7.5 Hz, 1H), 8.03 (dd, *J* = 8.4, 1.3 Hz, 1H), 7.70 (dd, *J* = 21.3, 6.3 Hz, 3H), 7.62 (dd, *J* = 12.7, 4.8 Hz, 3H), 6.81 – 6.77 (m, 1H), 5.17 (d, *J* = 6.9 Hz, 1H), 4.10 – 4.02 (m, 1H), 2.83 (dt, *J* = 13.6, 4.5 Hz, 1H), 2.57 (d, *J* = 4.6 Hz, 3H), 2.44 – 2.34 (m, 2H), 2.10 – 2.01 (m, 2H), 1.50 (d, *J* = 7.1 Hz, 3H). **^{13}C NMR (150 MHz, DMSO-d$_6$) δ ppm** 174.5, 166.9, 149.3, 142.1, 135.5, 135.3, 128.0, 127.5, 127.3, 126.9, 125.3, 125.3, 125.3, 124.7, 123.4, 115.5, 115.5, 49.3, 48.5, 34.4, 34.2, 32.7, 25.5, 21.8. **LCMS** m/z calcd for $(M + H)^+$ 465.5, found 465.4.

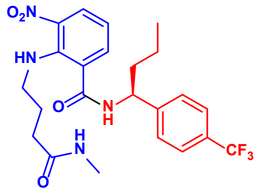

(*S*)-2-((4-(methylamino)-4-oxobutyl)amino)-3-nitro-N-(1-(4-(trifluoromethyl) phenyl) butyl) benzamide: Molecular Formula:$C_{23}H_{27}F_3N_4O_4$; yield 80%;**^{1}H NMR (600 MHz, CD_3OD) δ ppm** 8.08 (d, *J* = 8.5 Hz, 1H), 7.57 (d, *J* = 7.7 Hz, 2H), 7.48 (d, *J* = 7.6 Hz, 2H), 7.44 – 7.40 (m, 1H), 6.67 (t, *J* = 7.9 Hz, 1H), 4.99 – 4.95 (m, 1H), 2.94 – 2.88 (m, 1H), 2.86 – 2.80 (m, 1H), 2.56 (s, 3H), 1.95 – 1.87 (m, 2H), 1.85 – 1.80 (m, 1H), 1.74 – 1.67 (m, 1H), 1.60 (dd, *J* = 9.5, 5.2 Hz, 2H), 1.44 – 1.37 (m, 1H), 1.28 (d, *J* = 6.4 Hz, 1H), 0.90 (t, *J* = 7.3 Hz, 3H). **^{13}C NMR (150 MHz, CD_3OD) δ ppm** 175.3, 170.8, 148.6, 144.9, 137.3, 136.7, 130.6, 130.4, 129.4, 128.7, 127.0, 126.6, 126.6, 124.8, 116.6, 55.1, 46.7, 39.0, 33.9, 27.3, 26.3, 20.9, 14.0. **LCMS** m/z calcd for $(M + H)^+$ 481.4, found 481.3.

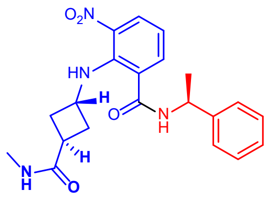

2-(((1r,3S)-3-(methylcarbamoyl)cyclobutyl)amino)-3-nitro-N-((S)-1-phenylethyl) ben zamide: Molecular Formula:$C_{12}H_{24}N_4O_4$; yield 40%; **^{1}H NMR (600 MHz, DMSO-d$_6$) δ ppm** 9.05 (d, *J* = 7.8 Hz, 1H), 8.27 (d, *J* = 7.4 Hz, 1H), 8.01 (d, *J* = 8.3 Hz, 1H), 7.68 (d, *J* = 4.3 Hz, 1H), 7.59 (d, *J* = 7.2 Hz, 1H), 7.41 (d, *J* = 7.5 Hz, 2H), 7.35 (t, *J* = 7.6 Hz, 2H), 7.24 (d, *J* = 7.2 Hz, 1H), 6.78 (t, *J* = 7.9 Hz, 1H), 5.15 – 5.07 (m, 1H), 4.05 (dd, *J* = 14.4, 7.2 Hz, 1H), 2.87 – 2.79 (m, 1H), 2.57 (d, *J* = 4.6 Hz, 3H), 2.47 – 2.34 (m, 2H), 2.06-2.04 (m, 2H), 1.48 (d, *J* = 7.0 Hz, 3H). **^{13}C NMR (150 MHz, DMSO-d$_6$) δ ppm** 174.4, 166.7, 144.5, 142.1, 135.3, 135.3, 128.3, 127.9, 126.7, 126.0, 124.9, 115.4, 49.2, 48.5, 34.4, 34.1, 32.8, 25.6, 22.0. **LCMS** m/z calcd for $(M + H)^+$ 397.4, found 397.4.

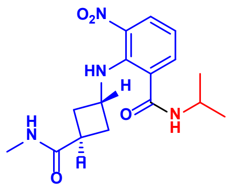

N-isopropyl-2-(((1r,3r)-3-(methylcarbamoyl)cyclobutyl)amino)-3-nitrobenzamide: Molecular Formula:$C_{16}H_{22}N_4O_4$; yield 100%; **^{1}H NMR (600 MHz, CD_3OD) δ ppm** 8.50 (d, *J* = 7.3 Hz, 1H), 8.15 (dd, *J* = 8.5, 1.4 Hz, 2H), 7.75 (s, 1H), 7.50 (dd, *J* = 7.3, 1.3 Hz, 2H), 6.80 – 6.74 (m, 2H), 4.27 (p, *J* = 7.1 Hz, 2H), 4.17-4.16 (m, 2H), 3.00 – 2.94 (m, 2H), 2.71 (d, *J* = 4.7 Hz, 6H), 2.66 – 2.59 (m, 4H), 2.21-2.22 (m, 4H), 1.27 (d, *J* = 6.6 Hz, 12H). **^{13}C NMR (150 MHz, CD_3OD) δ ppm** 178.0, 170.1, 143.7, 137.2, 129.2, 127.5, 117.0, 50.9, 43.2, 35.9, 34.8, 26.4, 22.4. **LCMS** m/z calcd for $(M + H)^+$ 335.3, found 335.2.

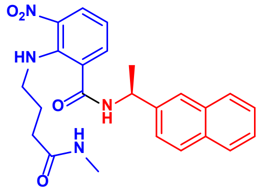

***(S)*-2-((4-(methylamino)-4-oxobutyl)amino)-N-(1-(naphthalen-2-yl)ethyl)-3-nitro benzamide:** Molecular Formula:$C_{24}H_{26}N_4O_4$; yield 84%; **^{1}H NMR (600 MHz, DMSO-d$_6$) δ ppm** 9.18 (s, 1H), 8.07 (s, 2H), 7.90 (s, 4H), 7.62 (d, *J* = 24.8 Hz, 3H), 7.50 (s, 2H), 6.77 (s, 1H), 5.27 (s, 1H), 2.99 (s, 2H), 2.53 (s, 3H), 1.98 (s, 2H), 1.67 (s, 2H), 1.56 (s, 3H). **^{13}C NMR (150 MHz, DMSO-d$_6$) δ ppm** 171.5, 167.1, 143.4, 141.7, 135.9, 134.7, 132.9, 132.1, 128.0, 127.9, 127.7, 127.5, 126.1, 125.7, 125.2, 124.9, 124.3, 115.0, 48.8, 45.0, 32.3, 25.7, 25.4, 21.9. **LCMS** m/z calcd for $(M + H)^+$ 435.4, found 435.3.

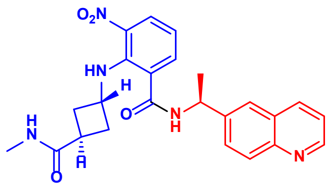

2-(((1r,3S)-3-(methylcarbamoyl)cyclobutyl)amino)-3-nitro-N-((S)-1-(quinolin-6-yl)ethyl)benzamide: Molecular Formula:$C_{24}H_{25}N_5O_4$; yield 79%; **^{1}H NMR (600 MHz, DMSO-d$_6$) δ ppm** 9.20 (d, *J* = 7.6 Hz, 1H), 8.88 (d, *J* = 2.8 Hz, 1H), 8.39 (d, *J* = 8.1 Hz, 1H), 8.29 (d, *J* = 7.4 Hz, 1H), 8.03 (d, *J* = 8.4 Hz, 2H), 7.96 (s, 1H), 7.85 (d, *J* = 8.8 Hz, 1H), 7.69 (d, *J* = 4.3 Hz, 1H), 7.66 (d, *J* = 7.0 Hz, 1H), 7.53 (dd, *J* = 8.2, 4.1 Hz, 1H), 6.79 (t, *J* = 7.9 Hz, 1H), 5.35 – 5.27 (m, 1H), 4.09 (dd, *J* = 14.5, 7.3 Hz, 1H), 2.83 (dd, *J* = 9.0, 4.7 Hz, 1H), 2.57 (d, *J* = 4.5 Hz, 3H), 2.44– 2.37 (m, 2H),2.10–2.02 (m, 2H), 1.59 (d, *J* = 7.0 Hz, 3H). **^{13}C NMR (150 MHz, CDCl$_3$) δ ppm** 174.1, 167.7, 154.3, 150.3, 147.4, 144.9, 144.3, 141.7, 136.7, 134.2, 132.2, 129.9, 128.7, 128.4, 128.3, 125.3, 124.5, 123.2, 122.7, 122.3, 121.6, 51.7, 49.9, 33.7, 29.8, 26.5, 22.1. **LCMS** m/z calcd for $(M + H)^+$ 448.4, found 448.3.

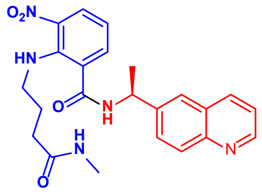

(S)-2-((4-(methylamino)-4-oxobutyl)amino)-3-nitro-N-(1-(quinolin-6-yl)ethyl)benzamide: Molecular Formula:$C_{23}H_{25}N_5O_4$; yield 81%; **^{1}H NMR (600 MHz, DMSO-d$_6$) δ ppm** 9.22 (d, *J* = 7.8 Hz, 1H), 8.88 (dd, *J* = 4.0, 1.4 Hz, 1H), 8.39 (d, *J* = 8.1 Hz, 1H), 8.07 (td, *J* = 8.0, 3.2 Hz, 2H), 8.03 (d, *J* = 8.7 Hz, 1H), 7.95 (s, 1H), 7.84 (dd, *J* = 8.7, 1.7 Hz, 1H), 7.70 – 7.63 (m, 2H), 7.53 (dd, *J* = 8.3, 4.2 Hz, 1H), 6.78 (t, *J* = 7.9 Hz, 1H), 5.30 (d, *J* = 7.1 Hz, 1H), 3.03 – 2.96 (m, 2H), 2.53 (d, *J* = 4.6 Hz, 3H), 1.98 (t, *J* = 7.4 Hz, 2H), 1.67 (d, *J* = 14.2 Hz, 2H), 1.57 (d, *J* = 7.0 Hz, 3H). **^{13}C NMR (150 MHz, DMSO-d$_6$) δ ppm** 171.5, 167.2, 150.2, 147.0, 143.4, 142.3, 135.9, 134.7, 129.1, 128.4, 127.9, 127.7, 125.1, 124.5, 121.6, 115.0, 48.6, 45.0, 32.3, 25.6, 25.4, 21.8. **LCMS** m/z calcd for $(M + H)^+$ 436.4, found 436.4.

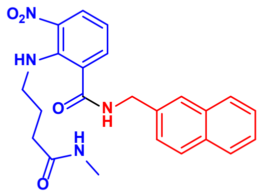

2-((4-(methylamino)-4-oxobutyl)amino)-N-(naphthalen-2-ylmethyl)-3-nitrobenzamide: Molecular Formula:$C_{23}H_{24}N_4O_4$; yield 80%; **^{1}H NMR (600 MHz, DMSO-d$_6$) δ ppm** 9.25 (t, *J* = 5.9 Hz, 1H), 8.15 (t, *J* = 5.0 Hz, 1H), 8.05 (dd, *J* = 8.4, 1.5 Hz, 1H), 7.91 (dd, *J* = 7.8, 5.5 Hz, 3H), 7.84 (s, 1H), 7.69 (dd, *J* = 7.3, 1.5 Hz, 2H), 7.52 – 7.47 (m, 3H), 6.77 (dd, *J* = 8.2, 7.6 Hz, 1H), 4.61 (d, *J* = 5.9 Hz, 2H), 3.01 (dd, *J* = 12.1, 6.8 Hz, 2H), 2.53 (d, *J* = 4.6 Hz, 3H), 2.02 (t, *J* = 7.4 Hz, 2H), 1.71 (m, 2H). **^{13}C NMR (150 MHz, DMSO-d$_6$) δ ppm** 171.6, 167.9, 143.5, 136.5, 135.5, 135.0, 132.9, 132.1, 128.1, 128.0, 127.6, 127.5, 126.2, 126.0, 125.7, 125.7, 124.6, 115.0, 45.1, 42.9, 32.4, 25.7, 25.4. **LCMS** m/z calcd for $(M + H)^+$ 421.4, found 421.4.

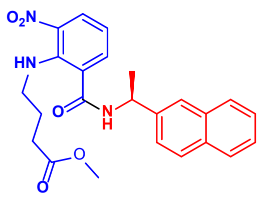

methyl (S)-4-((2-((1-(naphthalen-2-yl)ethyl)carbamoyl)-6-nitrophenyl)amino)butanoate: Molecular Formula:$C_{24}H_{25}N_3O_5$; yield 83%; **^{1}H NMR (600 MHz, DMSO-d$_6$) δ ppm** 9.19 (d, *J* = 8.0 Hz, 1H), 8.07 (dd, *J* = 8.4, 1.2 Hz, 1H), 7.99 (t, *J* = 5.2 Hz, 1H), 7.94 – 7.87 (m, 4H), 7.64 (dd, *J* = 7.2, 1.0 Hz, 1H), 7.59 (dd, *J* = 8.5, 1.0 Hz, 1H), 7.54 – 7.45 (m, 2H), 6.82 – 6.75 (m, 1H), 5.27 (s, 1H), 3.51 (s, 3H), 3.00 – 2.89 (m, 2H), 2.09 (t, *J* = 7.4 Hz, 2H), 1.68 – 1.61 (m, 2H), 1.55 (d, *J* = 7.1 Hz, 3H). **^{13}C NMR (150 MHz, DMSO-d$_6$) δ ppm** 172.0, 166.4, 142.7, 141.0, 135.3, 134.3, 132.3, 131.5, 127.4, 127.3, 127.1, 126.9, 125.6, 125.1, 124.8, 124.3, 123.8, 114.6, 50.7, 48.2, 44.0, 29.8, 24.3, 21.3. **LCMS** m/z calcd for $(M + H)^+$ 436.4, found 436.4.

Synthesized by following general procedure-Step-3:

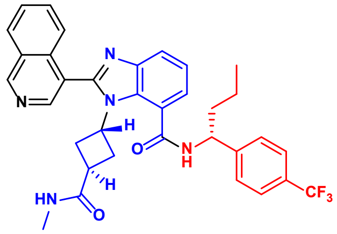

CDD-1732

2-(isoquinolin-4-yl)-1-((1r,3R)-3-(methylcarbamoyl)cyclobutyl)-N-((R)-1-(4-(trifluoro methyl) phenyl)butyl)-1H-benzo[d]imidazole-7-carboxamide: Molecular Formula: $C_{34}H_{32}F_3N_5O_2$; yield 63%; **^{1}H NMR (600 MHz, CDCl$_3$) δ ppm** 9.34 (s, 1H), 8.70 (s, 1H), 8.07 (d, *J* = 7.9 Hz, 1H), 7.97 (d, *J* = 7.8 Hz, 2H), 7.76 (d, *J* = 7.4 Hz, 1H), 7.70 (s, 1H), 7.62 (d, *J* = 8.0 Hz, 2H), 7.55 (d, *J* = 8.0 Hz, 2H), 7.48 (d, *J* = 7.3 Hz, 1H), 7.34 (t, *J* = 7.7 Hz, 1H), 6.67 (d, *J* = 6.5 Hz, 1H), 5.39 (s, 1H), 5.31 – 5.24 (m, 1H), 5.16 (s, 1H), 2.66 (d, *J* = 3.8 Hz, 3H), 2.27 (s, 1H), 2.14 – 1.98 (m, 4H), 1.94 – 1.84 (m, 2H), 1.52 – 1.45 (m, 1H), 1.39 (d, *J* = 7.0 Hz, 1H), 1.00 (t, *J* = 7.3 Hz, 3H). **^{13}C NMR (150 MHz, CD$_3$OD) δ ppm** 176.9, 170.1, 155.6, 148.9, 148.7, 145.1, 144.7, 135.5, 134.0, 133.1, 129.8, 129.8, 128.7, 126.5, 126.5, 125.1, 124.2, 123.7, 55.2, 53.2, 39.3, 34.4, 26.3, 20.9, 14.1. **HRMS (HESI-TOF)** m/z calcd for $(M + H)^+$ 600.2586, found 600.2564.

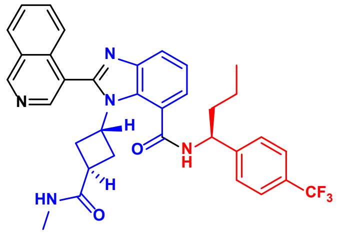

CDD-1733

2-(isoquinolin-4-yl)-1-((1r,3S)-3-(methylcarbamoyl)cyclobutyl)-N-((S)-1-(4-(trifluoro methyl) phenyl)butyl)-1H-benzo[d]imidazole-7-carboxamide: Molecular Formula: $C_{34}H_{32}F_3N_5O_2$; yield 66%;**^{1}H NMR (600 MHz, CDCl$_3$) δ ppm** 9.31 (s, 1H), 8.65 (s, 1H), 8.05 (d, *J* = 8.1 Hz, 1H), 7.95 (d, *J* = 8.0 Hz, 1H), 7.74 (t, *J* = 7.5 Hz, 1H), 7.68 (t, *J* = 7.5 Hz, 1H), 7.60 (d, *J* = 8.1 Hz, 2H), 7.54 (d, *J* = 8.1 Hz, 2H), 7.47 (d, *J* = 7.4 Hz, 1H), 7.31 (t, *J* = 7.8 Hz, 1H), 6.79 (d, *J* = 7.0 Hz, 1H), 5.37 (s, 1H), 5.26 (td, *J* = 13.7, 5.8 Hz, 2H), 2.65 (d, *J* = 4.5 Hz, 3H), 2.25 (s, 1H), 2.11 – 2.00 (m, 4H), 1.90-1.88 (m, 2H), 1.50 – 1.43 (m, 1H), 1.42 – 1.34 (m, 1H), 0.99 (t, *J* = 7.3 Hz, 3H). **^{13}C NMR (150 MHz, CD$_3$OD) δ ppm** 176.9, 170.1, 155.6, 152.2, 148.9, 145.1, 144.7, 135.5, 134.0, 133.1, 130.5, 130.3, 128.7, 126.5, 126.5, 125.1, 124.8, 124.2, 123.7, 123.1, 55.2, 53.2, 39.4, 34.4, 26.3, 20.9, 14.1. **HRMS (HESI-TOF)** m/z calcd for $(M + H)^+$ 600.2586, found 600.2569.

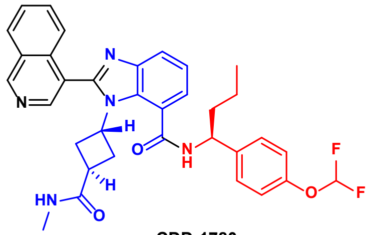

CDD-1780

N-((S)-1-(4-(difluoromethoxy)phenyl)butyl)-2-(isoquinolin-4-yl)-1-((1r,3S)-3-(methylcarbam oyl)cyclobutyl)-1H-benzo[d]imidazole-7-carboxamide: Molecular Formula: $C_{34}H_{33}F_2N_5O_3$; yield 60%; **^{1}H NMR (600 MHz, CD$_3$OD) δ ppm** 9.46 (s, 1H), 8.76 (s, 1H), 8.29 (d, *J* = 8.2 Hz, 1H), 8.04 (d, *J* = 8.2 Hz, 1H), 7.93 (dd, *J* = 17.0, 7.7 Hz, 2H), 7.85 (t, *J* = 7.6 Hz, 1H), 7.58 (s, 1H), 7.50 (d, *J* = 8.4 Hz, 2H), 7.45 (t, *J* = 7.8 Hz, 1H), 7.13 (d, *J* = 6.8 Hz, 2H), 6.81 (t, *J* = 74.3 Hz, 1H), 5.35 (s, 1H), 5.16 (t, *J* = 7.6 Hz, 1H), 2.60 (s, 3H), 2.41 (s, 1H), 2.17 – 1.98 (m, 4H), 1.89 – 1.82 (m, 1H), 1.76 (d, *J* = 48.2 Hz, 1H), 1.52 (s, 1H), 1.41 (s, 1H), 1.02 (t, *J* = 7.3 Hz, 3H). **^{13}C NMR (150 MHz, CD$_3$OD) δ ppm** 176.8, 169.7, 152.1, 134.6, 130.2, 130.1, 129.7, 125.5, 124.6, 124.2, 122.5, 120.2, 119.6, 117.9, 116.2, 54.9, 53.5, 34.4, 26.4, 21.0, 14.1**. HRMS (HESI-TOF)** m/z calcd for $(M + H)^+$ 598.2630, found 598.2613.

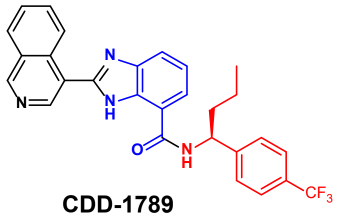

CDD-1789

***(S)*-2-(isoquinolin-4-yl)-N-(1-(4-(trifluoromethyl)phenyl)butyl)-1H-benzo[d]imidazole-7-car boxamide:** Molecular Formula:$C_{28}H_{23}F_3N_4O$; yield 58%; **^{1}H NMR (600 MHz, CDCl$_3$) δ ppm** 12.29 (s, 1H), 10.65 (s, 1H), 9.32 (s, 1H), 9.08 (d, *J* = 17.1 Hz, 2H), 8.18 – 8.06 (m, 2H), 7.77 (d, *J* = 6.8 Hz, 2H), 7.62 (d, J = 5.0 Hz, 1H), 7.46 (t, J = 14.5 Hz, 4H), 7.33 (t, J = 7.7 Hz, 1H), 5.34 (d, *J* = 6.3 Hz, 1H), 1.98 – 1.92 (m, 1H), 1.87-1.83 (m, 1H), 1.47 – 1.37 (m, 2H), 0.88 (d, *J* = 5.5 Hz, 3H). **^{13}C NMR (150 MHz, CDCl$_3$) δ ppm** 165.8, 154.3, 149.6, 147.5, 143.4, 142.0, 134.7, 133.6, 132.1, 128.8, 128.5, 128.4, 126.9, 125.5, 125.3, 125.1, 124.1, 123.5, 123.3, 122.6, 121.7, 121.7, 115.3, 53.7, 39.4, 19.5, 14.0. **HRMS (HESI-TOF)** m/z calcd for $(M + H)^+$ 489.1890, found 489.1882.

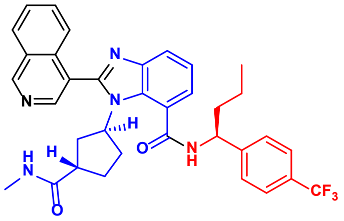

CDD-1790

2-(isoquinolin-4-yl)-1-((1S,3S)-3-(methylcarbamoyl)cyclopentyl)-N-((S)-1-(4-(trifluoro methyl)phenyl)butyl)-1H-benzo[d]imidazole-7-carboxamide: Molecular Formula: $C_{34}H_{33}F_2N_5O_3$; yield 75%; **^{1}H NMR (600 MHz, $CDCl_3$) δ ppm** 9.39 (s, 1H), 8.63 (s, 1H), 8.12 – 8.06 (m, 1H), 7.92 (d, *J* = 7.8 Hz, 1H), 7.72 – 7.66 (m, 2H), 7.63 (d, *J* = 8.0 Hz, 2H), 7.54 (dd, *J* = 18.1, 8.2 Hz, 3H), 7.39 – 7.31 (m, 2H), 6.57 (d, *J* = 4.4 Hz, 1H), 5.27 (dd, *J* = 15.3, 7.7 Hz, 1H), 5.11 – 4.96 (m, 1H), 2.64 (d, *J* = 4.5 Hz, 3H), 2.21 (s, 1H), 1.98-1.96 (m, 2H), 1.89-1.87 (m, 4H), 1.60 (s, 1H), 1.53 – 1.45 (m, 1H), 1.39 (m, 1H), 1.34 – 1.26 (m, 1H), 1.00 (t, *J* = 7.3 Hz, 3H). **^{13}C NMR (150 MHz, $CDCl_3$) δ ppm** 174.9, 168.5, 154.4, 150.9, 146.6, 144.4, 144.2, 135.2, 132.0, 131.7, 130.0, 129.8, 128.4, 128.2, 127.1, 126.0, 125.9, 125.9, 125.9, 125.0, 124.7, 123.2, 123.1, 122.7, 122.5, 122.1, 58.2, 53.9, 43.5, 38.5, 29.1, 26.3, 19.7, 13.9. **HRMS (HESI-TOF)** m/z calcd for $(M + H)^+$ 614.2743, found 614.2726.

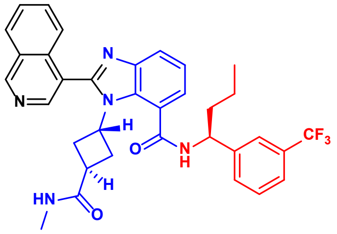

CDD-1795

2-(isoquinolin-4-yl)-1-((1r,3S)-3-(methylcarbamoyl)cyclobutyl)-N-((S)-1-(3-(trifluoro methyl) phenyl)butyl)-1H-benzo[d]imidazole-7-carboxamide: Molecular Formula: $C_{34}H_{32}F_3N_5O_2$; yield 60%; **^{1}H NMR (600 MHz, CD_3OD) δ ppm** 9.42 (s, 1H), 8.72 (s, 1H), 8.25 (d, *J* = 8.3 Hz, 1H), 8.03 (d, *J* = 8.1 Hz, 1H), 7.90 (d, *J* = 7.4 Hz, 2H), 7.79 (dd, *J* = 14.8, 6.9 Hz, 2H), 7.72 (d, *J* = 3.9 Hz, 1H), 7.60 – 7.51 (m, 3H), 7.45 – 7.39 (m, 1H), 5.36 (d, *J* = 11.0 Hz, 1H), 5.21 (t, *J* = 7.5 Hz, 1H), 2.56 (s, 3H), 2.39 (s, 1H), 2.20 – 1.95 (m, 4H), 1.85-1.84 (m, 1H), 1.73 (s, 1H), 1.52 (s, 1H), 1.39 (d, *J* = 13.6 Hz, 1H), 1.00 (t, *J* = 7.3 Hz, 3H). **^{13}C NMR (150 MHz, CD_3OD) δ ppm** 176.8, 170.0, 155.5, 152.1, 145.8, 145.0, 144.6, 135.4, 134.0, 133.0, 132.0, 131.9, 131.7, 130.4, 128.4, 126.6, 125.2, 125.1, 125.0, 124.9, 124.8, 124.6, 124.6, 124.1, 123.6, 123.0, 55.2, 39.4, 34.4, 26.3, 20.9, 14.1. **HRMS (HESI-TOF)** m/z calcd for $(M + H)^+$ 600.2586, found 600.2572.

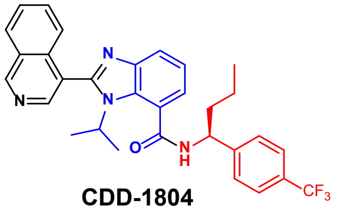

CDD-1804

(*S*)-1-isopropyl-2-(isoquinolin-4-yl)-N-(1-(4-(trifluoromethyl)phenyl)butyl)-1H-benzo [d]imi dazole-7-carboxamide: Molecular Formula: $C_{31}H_{29}F_3N_4O$; yield 70%; **^{1}H NMR (600 MHz, $CDCl_3$) δ ppm** 9.34 (s, 1H), 8.59 (s, 1H), 8.08 – 8.02 (m, 1H), 7.88 (d, *J* = 7.3 Hz, 1H), 7.68 – 7.62 (m,

2H), 7.59 (d, J = 8.1 Hz, 2H), 7.50 (d, J = 8.1 Hz, 3H), 7.34 (d, J = 6.6 Hz, 1H), 7.29 (t, J = 7.7 Hz, 1H), 6.68 (d, J = 8.1 Hz, 1H), 5.24 (q, J = 7.9 Hz, 1H), 4.75 (s, 1H), 1.97-1.92 (m, 1H), 1.90 – 1.83 (m, 1H), 1.53 – 1.45 (m, 1H), 1.43 – 1.34 (m, 1H), 0.99 (t, J = 7.4 Hz, 9H). **^{13}C NMR (150 MHz, $CDCl_3$) δ ppm** 168.4, 154.3, 150.7, 146.4, 144.4, 144.2, 135.4, 131.7, 131.6, 130.0, 129.8, 128.2, 128.1, 128.0, 127.2, 125.8, 125.8, 125.0, 123.2, 122.6, 122.5, 122.3, 121.9, 53.8, 49.9, 38.0, 19.7, 13.9. **HRMS (HESI-TOF)** m/z calcd for $(M + H)^+$ 531.2372, found 531.2363.

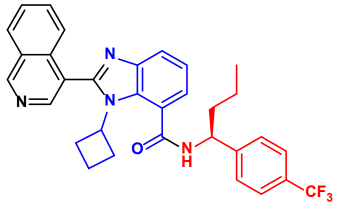

CDD-1806

(*S*)-1-cyclobutyl-2-(isoquinolin-4-yl)-N-(1-(4-(trifluoromethyl)phenyl)butyl)-1H-benzo[d] imidazole-7-carboxamide: Molecular Formula:$C_{32}H_{29}F_3N_4O$; yield 65%; **^{1}H NMR (600 MHz, $CDCl_3$) δ ppm** 9.31 (s, 1H), 8.70 (s, 1H), 8.04 (d, J = 8.1 Hz, 1H), 7.96 (dd, J = 19.6, 8.0 Hz, 2H), 7.73 (s, 1H), 7.67 (t, J = 7.4 Hz, 1H), 7.60 (d, J = 8.1 Hz, 2H), 7.54 (d, J = 8.0 Hz, 2H), 7.46 (d, J = 7.4 Hz, 1H), 7.30 (t, J = 7.7 Hz, 1H), 6.79 (s, 1H), 5.19 (q, J = 7.6 Hz, 1H), 4.95 (d, J = 6.5 Hz, 1H), 2.01-2.00 (m, 1H), 1.94 – 1.87 (m, 1H), 1.49 – 1.44 (m, 1H), 1.39-1.38 (m, 1H), 1.24 – 1.15 (m, 4H), 0.98 (dd, J = 9.4, 5.3 Hz, 3H), 0.96 (t, J = 3.4 Hz, 1H), 0.88 (d, J = 6.8 Hz, 1H). **^{13}C NMR (150 MHz, $CDCl_3$) δ ppm** 167.6, 154.2, 151.3, 146.4, 144.5, 134.4, 132.4, 132.0, 128.2, 128.1, 127.4, 125.8, 124.5, 123.2, 122.9, 122.5, 122.1, 54.0, 53.0, 38.2, 19.7, 15.1, 13.9. **HRMS (HESI-TOF)** m/z calcd for $(M + H)^+$ 543.2352, found 543.2351.

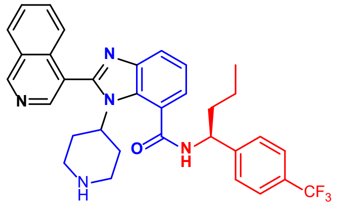

CDD-1818

***(S)*-2-(isoquinolin-4-yl)-1-(piperidin-4-yl)-N-(1-(4-(trifluoromethyl)phenyl)butyl)-1H-benzo[d]imidazole-7-carboxamide:** Molecular Formula:$C_{33}H_{32}F_3N_5O$; yield 43%; **^{1}H NMR (600 MHz, $CDCl_3$) δ ppm** 9.34 (s, 1H), 8.57 (s, 1H), 8.05 (dd, J = 6.5, 2.8 Hz, 1H), 7.88 (d, J = 7.9 Hz, 1H), 7.65-7.64 (m, 4H), 7.54 – 7.46 (m, 3H), 7.38 (d, J = 6.9 Hz, 1H), 7.30 (t, J = 7.7 Hz, 1H), 6.79 (s, 1H), 5.24 (q, J = 7.8 Hz, 1H), 4.51-4.49 (m, 1H), 2.76-2.75 (m, 2H), 2.20 (t, J = 11.8 Hz, 1H), 1.99 – 1.67 (m, 6H), 1.54 – 1.34 (m, 3H), 0.99 (t, J = 7.3 Hz, 3H). **^{13}C NMR (150 MHz, $CDCl_3$) δ ppm** 168.3, 154.4, 150.8, 146.8, 144.4, 144.1, 135.4, 132.0, 131.7, 130.1, 129.9, 128.2, 128.1, 128.1, 127.2, 125.9, 125.9, 124.9, 123.2, 123.0, 122.8, 122.0, 57.2, 53.9, 46.4, 46.1, 38.7, 19.8, 13.9. **HRMS (HESI-TOF)** m/z calcd for $(M + H)^+$ 572.2637, found 572.2631.

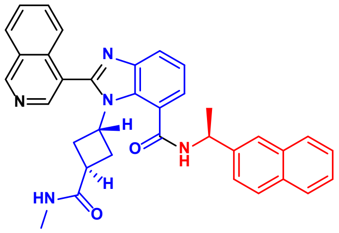

CDD-1819

2-(isoquinolin-4-yl)-1-((1r,3S)-3-(methylcarbamoyl)cyclobutyl)-N-((S)-1-(naphthaln2-yl) ethyl)-1H-benzo[d]imidazole-7-carboxamide: Molecular Formula: $C_{35}H_{31}N_5O_2$; yield 64%; **^{1}H NMR (600 MHz, CDCl$_3$) δ ppm** 9.25 (s, 1H), 8.60 (s, 1H), 8.00 (d, *J* = 8.6 Hz, 1H), 7.94 (d, *J* = 7.7 Hz, 1H), 7.87 (s, 1H), 7.85 – 7.77 (m, 3H), 7.64 (dd, *J* = 17.1, 9.2 Hz, 2H), 7.52 (dd, *J* = 8.5, 1.6 Hz, 1H), 7.49 (dd, *J* = 7.5, 0.8 Hz, 1H), 7.47 – 7.43 (m, 2H), 7.30 (t, *J* = 7.8 Hz, 1H), 6.97 (d, *J* = 5.8 Hz, 1H), 5.50 (dd, *J* = 14.4, 7.1 Hz, 1H), 4.90 (s, 1H), 2.38 (s, 3H), 2.23 – 2.19 (m, 1H), 2.11 (t, *J* = 9.5 Hz, 2H), 2.01 (s, 2H), 1.75 (d, *J* = 6.9 Hz, 3H). **^{13}C NMR (150 MHz, CDCl$_3$) δ ppm** 173.9, 167.7, 154.4, 151.6, 145.0, 144.4, 140.8, 134.3, 133.4, 132.9, 132.1, 128.8, 128.3, 128.2, 128.0, 127.8, 126.7, 126.3, 125.0, 124.7, 123.3, 123.2, 122.7, 122.2, 51.7, 50.1, 33.5, 26.4, 22.5. **HRMS (HESI-TOF)** m/z calcd for $(M + H)^+$ 572.2556, found 554.2551.

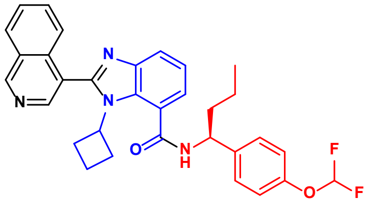

CDD-1820

(S)-1-cyclobutyl-N-(1-(4-(difluoromethoxy)phenyl)butyl)-2-(isoquinolin-4-yl)-1H-benzo[d] imidazole-7-carboxamide: Molecular Formula: $C_{32}H_{30}F_2N_4O_2$; yield 62%; **^{1}H NMR (600 MHz, CDCl$_3$) δ ppm** 9.29 (s, 1H), 8.66 (s, 1H), 8.04 (d, *J* = 8.1 Hz, 1H), 7.94 (dd, *J* = 14.2, 8.2 Hz, 2H), 7.71 (s, 1H), 7.66 (t, *J* = 7.4 Hz, 1H), 7.44 (d, *J* = 7.4 Hz, 1H), 7.40 (d, *J* = 8.6 Hz, 2H), 7.27 (dd, *J* = 14.5, 6.8 Hz, 1H), 7.08 (d, *J* = 8.5 Hz, 2H), 7.03 (d, *J* = 7.8 Hz, 1H), 6.48 (t, *J* = 73.8 Hz, 1H), 5.21 – 5.10 (m, 1H), 4.96 (s, 1H), 2.02 – 1.94 (m, 1H), 1.91 – 1.83 (m, 1H), 1.56 (s, 1H), 1.50 – 1.40 (m, 2H), 1.40 – 1.32 (m, 2H), 1.26 – 1.16 (m, 3H), 0.97 (t, *J* = 7.4 Hz, 3H). **^{13}C NMR (150 MHz, CD$_3$OD) δ ppm** 170.0, 155.4, 152.0, 151.9, 145.0, 141.2, 133.8, 133.1, 129.8, 129.7, 129.5, 125.2, 125.2, 124.3, 123.6, 122.8, 120.4, 119.4, 117.6, 115.9, 54.9, 54.2, 39.0, 20.9, 15.8, 14.1. **HRMS (HESI-TOF)** m/z calcd for $(M + H)^+$ 572.2268, found 572.2261.

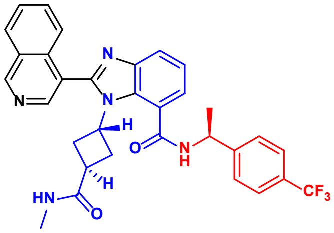

CDD-1829

2-(isoquinolin-4-yl)-1-((1r,3S)-3-(methylcarbamoyl)cyclobutyl)-N-((S)-1-(4-(trifluoro methyl) phenyl)ethyl)-1H-benzo[d]imidazole-7-carboxamide: Molecular Formula: $C_{32}H_{28}F_3N_5O_2$; yield 100%; **^{1}H NMR (600 MHz, CDCl$_3$) δ ppm** 9.30 (d, *J* = 15.6 Hz, 1H), 8.65 (d, *J* = 25.2 Hz, 1H), 8.05 (t, *J* = 7.3 Hz, 1H), 7.99 (s, 1H), 7.94 (t, *J* = 8.6 Hz, 1H), 7.82 – 7.72 (m, 1H), 7.69 (dd, *J* = 7.5, 3.4 Hz, 1H), 7.61 (d, *J* = 6.9 Hz, 2H), 7.57 (t, *J* = 6.6 Hz, 2H), 7.46 (t, *J* = 6.3 Hz, 1H), 7.34 –

7.29 (m, 1H), 6.74-6.73 (m 1H), 5.46 – 5.41 (m, 1H), 5.33-5.31 (m, 1H), 2.67 (d, *J* = 5.4 Hz, 3H), 2.31 (d, *J* = 7.0 Hz, 2H), 2.07 (dd, *J* = 22.5, 11.1 Hz, 3H), 1.71 (t, *J* = 5.7 Hz, 3H). **^{13}C NMR (150 MHz, $CDCl_3$) δ ppm** 174.0, 167.5, 154.4, 151.4, 147.3, 145.0, 144.5, 134.2, 132.2, 128.3, 126.9, 126.0, 125.9, 125.1, 124.5, 123.7, 123.4, 123.1, 122.4, 122.3, 51.8, 49.6, 33.8, 29.8, 26.5, 22.2. **HRMS (HESI-TOF)** m/z calcd for $(M + H)^+$ 572.2268, found 572.2261.

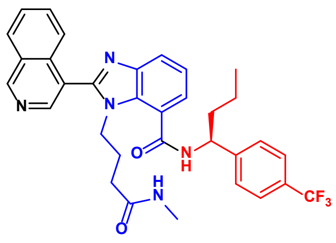

CDD-1830

(S)-2-(isoquinolin-4-yl)-1-(4-(methylamino)-4-oxobutyl)-N-(1-(4-(trifluoromethyl) phenyl) butyl)-1H-benzo[d]imidazole-7-carboxamide: Molecular Formula: $C_{33}H_{32}F_3N_5O_2$; yield 82%;**^{1}H NMR (600 MHz, CD_3OD) δ ppm** 9.38 (s, 1H), 8.67 (s, 1H), 8.10 (d, J = 7.9 Hz, 1H), 7.97 (d, J = 8.0 Hz, 1H), 7.72 (dd, J = 14.3, 6.9 Hz, 3H), 7.60 (d, J = 8.2 Hz, 2H), 7.55 (d, J = 8.1 Hz, 2H), 7.49 (d, J = 7.4 Hz, 1H), 7.37 (t, J = 7.8 Hz, 1H), 6.94 (s, 1H), 5.18 (q, J = 7.6 Hz, 1H), 4.26 – 4.18 (m, 1H), 4.12 – 4.04 (m, 1H), 2.51 (d, J = 4.8 Hz, 3H), 2.04 – 1.96 (m, 1H), 1.90-1.89 (m, 2H), 1.62 (dd, J = 13.3, 6.0 Hz, 1H), 1.53 – 1.47 (m, 3H), 1.42 – 1.35 (m, 1H), 0.99 (t, J = 7.3 Hz, 3H). **^{13}C NMR (150 MHz, CD_3OD) δ ppm** 171.6, 167.6, 154.6, 151.9, 146.6, 144.9, 144.6, 134.6, 132.2, 131.4, 129.8, 129.6, 128.4, 128.3, 127.4, 125.8, 125.8, 125.1, 124.4, 123.3, 123.1, 122.3, 122.2, 54.2, 45.8, 38.4, 32.5, 26.1, 25.9, 19.7, 13.9. **HRMS (HESI-TOF)** m/z calcd for $(M + H)^+$ 572.2586, found 588.2573.

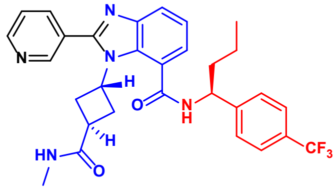

CDD-1831

1-((1r,3S)-3-(methylcarbamoyl)cyclobutyl)-2-(pyridin-3-yl)-N-((S)-1-(4-(trifluoro methyl) phenyl)butyl)-1H-benzo[d]imidazole-7-carboxamide: Molecular Formula: $C_{30}H_{30}F_3N_5O_2$; yield 58%;**^{1}H NMR (600 MHz, CD_3OD) δ ppm** 8.89 (s, 1H), 8.70 (s, 1H), 8.17 (s, 1H), 7.85 (d, *J* = 8.2 Hz, 1H), 7.78 – 7.47 (m, 6H), 7.40 (dd, *J* = 7.4, 3.0 Hz, 1H), 5.17 (s, 1H), 4.93 (d, *J* = 6.7 Hz, 1H), 2.54 (dd, *J* = 6.8, 3.1 Hz, 3H), 2.42 – 2.23 (m, 2H), 2.03 (s, 1H), 1.85-1.83 (m, 4H), 1.49-1.47 (m, 2H), 1.10 – 0.86 (m, 3H). **^{13}C NMR (150 MHz, CD_3OD) δ ppm** 175.2, 170.0, 153.4, 151.7, 150.4, 148.7, 145.0, 138.8, 133.5, 130.6, 130.4, 129.4, 128.9, 126.6, 126.5, 126.5, 125.3, 124.8, 124.0, 123.7, 123.2, 55.1, 50.5, 39.0, 36.4, 35.3, 33.7, 26.2, 20.9, 14.0. **HRMS (HESI-TOF)** m/z calcd for $(M + H)^+$ 550.2430, found 550.2425.

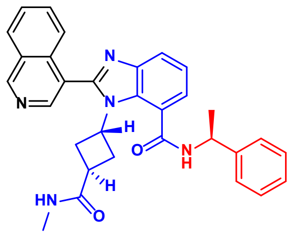

CDD-1841

2-(isoquinolin-4-yl)-1-((1r,3S)-3-(methylcarbamoyl)cyclobutyl)-N-((S)-1-phenylethy l)-1H-benzo[d]imidazole-7-carboxamide: Molecular Formula: $C_{13}H_{29}N_5O_2$; yield 65%; **^{1}H NMR (600 MHz, CD_3OD) δ ppm** 9.45 (s, 1H), 9.23 (d, *J* = 7.4 Hz, 1H), 8.76 (s, 1H), 8.28 (d, *J* = 8.2 Hz, 1H), 8.05 (d, *J* = 8.2 Hz, 1H), 7.91 (t, *J* = 9.0 Hz, 2H), 7.83 (t, *J* = 7.5 Hz, 1H), 7.56 (d, *J* = 7.2 Hz, 1H), 7.49 – 7.39 (m, 4H), 7.35 (t, *J* = 7.4 Hz, 2H), 7.24 (t, *J* = 7.1 Hz, 1H), 5.42 – 5.32 (m, 1H), 5.35 – 5.27 (m, 1H), 2.60 (s, 3H), 2.45 (s, 1H), 2.17-2.18 (m, 3H), 1.84 (dd, *J* = 6.4, 3.8 Hz, 1H), 1.64 (d, *J* = 7.0 Hz, 3H). **^{13}C NMR (150 MHz, CD_3OD) δ ppm** 175.5, 168.4, 154.2, 143.6, 143.3, 132.6, 128.5, 128.4, 128.2, 126.8, 126.0, 123.0, 122.3, 121.4, 51.9, 49.8, 49.7, 33.1, 25.0, 21.1. **HRMS (HESI-TOF)** m/z calcd for $(M + H)^+$ 504.2400, found 504.2379.

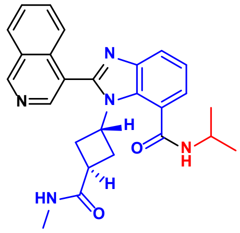

CDD-1842

N-isopropyl-2-(isoquinolin-4-yl)-1-((1r,3r)-3-(methylcarbamoyl)cyclobutyl)-1H-benzo[d] imidazole-7-carboxamide: Molecular Formula: $C_{26}H_{27}N_5O_2$; yield 86%; **^{1}H NMR (600 MHz, $CDCl_3$) δ ppm** 9.32 (s, 1H), 8.69 (s, 1H), 8.04 (dd, *J* = 16.2, 8.3 Hz, 2H), 7.92 (d, *J* = 7.9 Hz, 1H), 7.77 (t, *J* = 7.4 Hz, 1H), 7.69 (t, *J* = 7.5 Hz, 1H), 7.44 (d, *J* = 7.3 Hz, 1H), 7.32 (t, *J* = 7.7 Hz, 1H), 6.18 (d, *J* = 7.7 Hz, 1H), 5.61 (d, *J* = 3.8 Hz, 1H), 5.44 (q, *J* = 8.2 Hz, 1H), 4.35– 4.28 (m, *J* = 13.1, 6.6 Hz, 1H), 2.68 (d, *J* = 4.8 Hz, 3H), 2.40 (d, *J* = 9.8 Hz, 1H), 2.31 – 2.00 (m, 4H), 1.35 (d, *J* = 6.5 Hz, 6H). **^{13}C NMR (150 MHz, $CDCl_3$) δ ppm** 174.1, 167.6, 154.3, 151.1, 144.8, 144.4, 134.3, 132.2, 132.1, 128.3, 124.6, 123.2, 123.2, 122.9, 122.3, 51.8, 42.5, 33.9, 26.5, 22.9. **HRMS (HESI-TOF)** m/z calcd for $(M + H)^+$ 442.2243, found 442.2220.

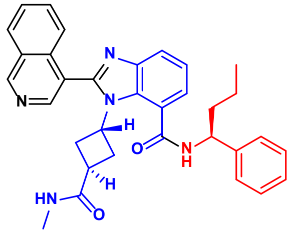

CDD-1843

2-(isoquinolin-4-yl)-1-((1r,3S)-3-(methylcarbamoyl)cyclobutyl)-N-((S)-1-phenylbutyl)-1H-benzo[d]imidazole-7-carboxamide: Molecular Formula: $C_{33}H_{33}N_5O_2$; yield 78%; **^{1}H NMR (600 MHz, $CDCl_3$) δ ppm** 9.21 (s, 1H), 8.58 (d, *J* = 4.6 Hz, 1H), 7.99-7.94 (m, 3H), 7.63 (dd, *J* = 2.2,

1.4 Hz, 2H), 7.46 (d, *J* = 7.0 Hz, 1H), 7.35 (d, *J* = 7.3 Hz, 2H), 7.24-7.23 (m, 6H), 5.41 (d, *J* = 18.3 Hz, 1H), 5.10 (dd, *J* = 15.0, 7.3 Hz, 1H), 4.78 (s, 1H), 2.45-2.43 (m, 4H), 2.02 – 1.77 (m, 4H), 1.69 (s, 1H), 1.55 (m, 2H), 1.41 (s, 1H), 1.32 (d, *J* = 6.4 Hz, 1H), 0.91 (t, *J* = 6.7 Hz, 3H). **^{13}C NMR (150 MHz, $CDCl_3$) δ ppm** 172.5, 167.5, 154.1, 151.0, 144.8, 144.4, 142.4, 132.2, 132.1, 128.6, 128.1, 128.0, 127.2, 127.1, 123.0, 122.5, 122.0, 54.1, 49.0, 38.2, 33.1, 26.1, 19.7, 13.8. **HRMS (HESI-TOF)** m/z calcd for $(M + H)^+$ 532.2692, found 532.2698.

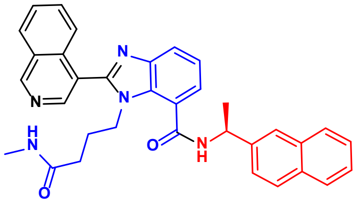

CDD-1845

(*S*)-2-(isoquinolin-4-yl)-1-(4-(methylamino)-4-oxobutyl)-N-(1-(naphthalen-2-yl)ethyl)-1H-benzo[d]imidazole-7-carboxamide: Molecular Formula: $C_{34}H_{31}N_5O_2$; yield 63%; **^{1}H NMR (600 MHz, $CDCl_3$) δ ppm** 9.31 (s, 1H), 8.54 (s, 1H), 8.04 (d, *J* = 7.8 Hz, 1H), 7.94 – 7.87 (m, 2H), 7.83 (dd, *J* = 17.4, 8.3 Hz, 3H), 7.71 – 7.64 (m, 3H), 7.55 (d, *J* = 8.5 Hz, 1H), 7.47 (dd, *J* = 11.5, 6.5 Hz, 3H), 7.31 (t, *J* = 7.8 Hz, 1H), 7.01 (d, *J* = 7.8 Hz, 1H), 5.50 (d, *J* = 7.0 Hz, 1H), 5.12 (d, *J* = 14.0 Hz, 1H), 4.21 – 4.10 (m, 2H), 2.38 (d, *J* = 4.7 Hz, 3H), 2.03 (d, *J* = 7.7 Hz, 1H), 1.75 (d, *J* = 6.9 Hz, 3H), 1.71 – 1.63 (m, 1H), 1.52 (m, 1H), 1.43-1.42 (m, 2H). **^{13}C NMR (150 MHz, $CDCl_3$) δ ppm** 171.5, 167.4, 144.9, 140.5, 133.5, 132.9, 132.1, 131.5, 128.9, 128.3, 128.0, 127.8, 126.6, 126.2, 125.2, 124.7, 124.4, 123.0, 122.4, 122.2, 50.1, 45.9, 32.5, 26.2, 26.1, 22.0. **HRMS (HESI-TOF)** m/z calcd for $(M + H)^+$ 542.2556, found 542.2540.

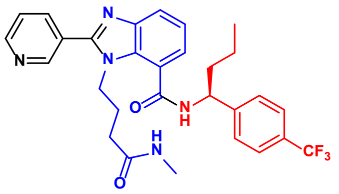

CDD-1846

(S)-1-(4-(methylamino)-4-oxobutyl)-2-(pyridin-3-yl)-N-(1-(4-(trifluoro methyl)phenyl) butyl)-1H-benzo[d]imidazole-7-carboxamide: Molecular Formula: $C_{29}H_{30}F_3N_5O_2$; yield 51%; **^{1}H NMR (600 MHz, CD_3OD) δ ppm** 8.89 (s, 2H), 8.75 (d, *J* = 3.9 Hz, 2H), 8.17 (d, *J* = 7.9 Hz, 2H), 7.91 (s, 1H), 7.87 (d, *J* = 8.1 Hz, 2H), 7.66 (dd, *J* = 19.3, 8.3 Hz, 1H), 7.55 (d, *J* = 7.3 Hz, 2H), 7.43 (t, *J* = 7.8 Hz, 2H), 5.18 (dd, *J* = 8.4, 6.9 Hz, 2H), 4.42 – 4.22 (m, 4H), 2.50 (s, 6H), 2.06 – 1.95 (m, 2H), 1.91 – 1.78 (m, 2H), 1.69 – 1.38 (m, 3H), 1.02 (t, *J* = 7.4 Hz, 3H). **^{13}C NMR (150 MHz, CD_3OD) δ ppm** 174.2, 169.7, 154.0, 151.7, 150.9, 149.0, 144.9, 139.2, 132.5, 128.7, 126.6, 126.6, 125.1, 123.9, 123.8, 122.9, 55.3, 46.5, 39.3, 32.6, 26.2, 21.0, 14.1. **HRMS (HESI-TOF)** m/z calcd for $(M + H)^+$ 542.2426, found 538.2424.

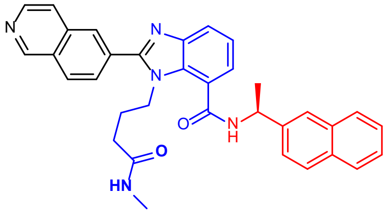

CDD-1884

(*S*)-2-(isoquinolin-6-yl)-1-(4-(methylamino)-4-oxobutyl)-N-(1-(naphthalen-2-yl)ethyl)-1H-benzo[d]imidazole-7-carboxamide: Molecular Formula: $C_{34}H_{31}N_5O_2$; yield 77%;**^{1}H NMR (600 MHz, $CDCl_3$) δ ppm** 9.32 (s, 1H), 8.60 (d, *J* = 5.7 Hz, 1H), 8.26 (s, 1H), 8.11 (d, *J* = 8.5 Hz, 1H), 7.98 – 7.92 (m, 3H), 7.90 – 7.84 (m, 3H), 7.82 (d, *J* = 5.7 Hz, 1H), 7.56 (dd, *J* = 8.5, 1.7 Hz, 1H), 7.53 – 7.48 (m, 2H), 7.45 (dd, *J* = 7.4, 0.9 Hz, 1H), 7.32 (t, *J* = 7.8 Hz, 1H), 6.68 (d, *J* = 7.9 Hz, 1H), 5.51 (d, *J* = 7.1 Hz, 1H), 4.70 (d, *J* = 3.9 Hz, 1H), 4.48 – 4.32 (m, 2H), 2.41 (d, *J* = 4.8 Hz, 3H), 1.92 – 1.83 (m, 1H), 1.76 (d, *J* = 7.0 Hz, 3H), 1.67 (d, *J* = 2.6 Hz, 1H), 1.49 (dt, *J* = 14.4, 7.1 Hz, 1H), 1.38 – 1.31 (m, 1H). **^{13}C NMR (150 MHz, $CDCl_3$) δ ppm** 171.6, 167.4, 154.7, 152.5, 144.8, 143.9, 140.7, 135.6, 133.5, 133.0, 132.2, 132.0, 129.0, 128.6, 128.4, 128.3, 128.3, 128.0, 127.9, 126.7, 126.3, 125.4, 124.7, 123.1, 122.9, 122.3, 122.2, 121.2, 50.1, 45.8, 32.1, 26.1, 26.0, 22.2. **HRMS (HESI-TOF)** m/z calcd for $(M + H)^+$ 542.2542, found 542.2541.

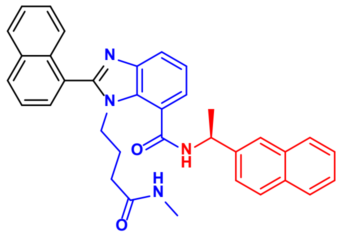

CDD-1885

(*S*)-1-(4-(methylamino)-4-oxobutyl)-2-(naphthalen-1-yl)-N-(1-(naphthalen-2-yl)ethyl)-1H-benzo[d]imidazole-7-carboxamide: Molecular Formula: $C_{34}H_{31}N_5O_2$; yield 93%; **^{1}H NMR (600 MHz, $CDCl_3$) δ ppm** 7.99 (d, *J* = 8.2 Hz, 1H), 7.93 (t, *J* = 8.6 Hz, 2H), 7.89 (s, 1H), 7.83 (dd, *J* = 16.5, 8.0 Hz, 3H), 7.63 (d, *J* = 7.7 Hz, 2H), 7.58 – 7.50 (m, 3H), 7.50 – 7.43 (m, 4H), 7.30 (t, *J* = 7.8 Hz, 1H), 6.78 (d, *J* = 7.2 Hz, 1H), 5.49 (d, *J* = 7.0 Hz, 1H), 4.87 (s, 1H), 4.11 (s, 2H), 2.35 (d, *J* = 4.7 Hz, 3H), 1.76 – 1.65 (m, 4H), 1.59 – 1.52 (m, 1H), 1.42 (dd, *J* = 15.4, 7.7 Hz, 2H). **^{13}C NMR (150 MHz, $CDCl_3$) δ ppm** 171.7, 167.4, 154.6, 144.8, 140.5, 133.6, 133.4, 132.9, 132.2, 131.3, 130.7, 129.1, 128.9, 128.6, 128.0, 127.8, 127.7, 127.5, 126.6, 126.6, 126.2, 125.3, 125.2, 125.1, 124.7, 122.9, 122.6, 122.1, 121.9, 50.0, 45.7, 32.7, 26.1, 26.1, 22.0. **HRMS (HESI-TOF)** m/z calcd for $(M + H)^+$ 541.2589, found 541.2587.

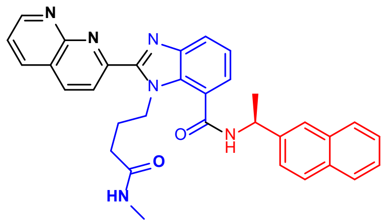

CDD-1905

(*S*)-1-(4-(methylamino)-4-oxobutyl)-N-(1-(naphthalen-2-yl)ethyl)-2-(1,8-naphthyridin-2-yl)-1H-benzo[d]imidazole-7-carboxamide: Molecular Formula: $C_{33}H_{30}N_6O_2$; yield 16%; **^{1}H NMR (600 MHz, DMSO-d_6) δ ppm** 9.41 (d, *J* = 7.9 Hz, 1H), 9.18 (dd, *J* = 4.1, 1.9 Hz, 1H), 8.68 (d, *J* = 8.5 Hz, 1H), 8.60 – 8.54 (m, 2H), 7.98 – 7.93 (m, 4H), 7.93 – 7.89 (m, 2H), 7.74 (dd, *J* = 8.1, 4.2 Hz, 1H), 7.67 (d, *J* = 8.5 Hz, 1H), 7.54 – 7.47 (m, 3H), 7.45 (d, *J* = 7.3 Hz, 1H), 7.39 (t, *J* = 7.7 Hz, 1H), 5.46 – 5.40 (m, 1H), 5.21 (dd, *J* = 13.9, 7.4 Hz, 1H), 5.08 – 5.00 (m, 1H), 2.34 (d, *J* = 4.5 Hz, 3H), 1.92 – 1.81 (m, 4H), 1.61 (d, *J* = 7.0 Hz, 3H). **^{13}C NMR (150 MHz, DMSO-d_6) δ ppm** 171.1, 166.5, 154.5, 154.4, 152.7, 150.3, 143.3, 142.0, 138.8, 137.6, 132.9, 132.2, 132.1, 128.1, 127.8, 127.5, 126.1, 125.7, 125.0, 124.3, 124.0, 123.6, 123.2, 123.0, 122.4, 122.1, 121.7, 48.9, 45.3, 32.1, 26.5, 25.3, 22.0. **HRMS (HESI-TOF)** m/z calcd for $(M + H)^+$ 542.2499, found 542.2498.

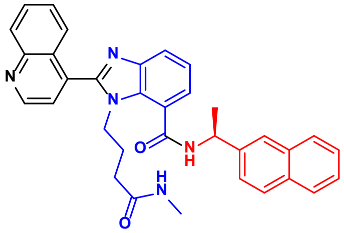

CDD-1906

(*S*)-1-(4-(methylamino)-4-oxobutyl)-N-(1-(naphthalen-2-yl)ethyl)-2-(quinolin-4-yl)-1H-benzo [d]imidazole-7-carboxamide: Molecular Formula: $C_{34}H_{31}N_5O_2$; yield 29%; **^{1}H NMR (600 MHz, $CDCl_3$) δ ppm** 9.05 (d, *J* = 4.3 Hz, 1H), 8.24 (d, *J* = 8.4 Hz, 1H), 7.98 (d, *J* = 8.0 Hz, 1H), 7.91 (s, 1H), 7.87 – 7.81 (m, 3H), 7.80 – 7.75 (m, 2H), 7.60 (d, *J* = 4.2 Hz, 1H), 7.56 (t, *J* = 7.3 Hz, 2H), 7.50-7.48 (m, 3H), 7.36 (t, *J* = 7.8 Hz, 1H), 6.70 (d, *J* = 7.4 Hz, 1H), 5.54 – 5.47 (m, 1H), 4.75 (s, 1H), 4.16-4.14 (m, 2H), 2.38 (d, *J* = 4.8 Hz, 3H), 1.76 (d, *J* = 6.9 Hz, 3H), 1.74 – 1.68 (m, 1H), 1.54 (d, *J* = 7.0 Hz, 1H), 1.44 – 1.34 (m, 2H). **^{13}C NMR (150 MHz, $CDCl_3$) δ ppm** 171.4, 167.2, 140.4, 133.5, 131.5, 130.5, 129.0, 128.3, 128.0, 127.8, 126.7, 126.3, 125.5, 125.3, 124.7, 123.4, 123.2, 122.9, 122.4, 50.2, 45.9, 32.3, 26.2, 26.1, 22.0. **HRMS (HESI-TOF)** m/z calcd for $(M + H)^+$ 542.2533, found 542.2532.

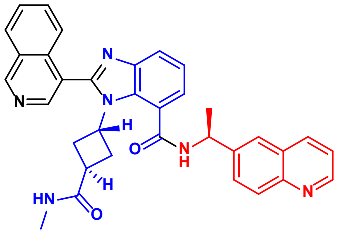

CDD-1907

2-(isoquinolin-4-yl)-1-((1r,3S)-3-(methylcarbamoyl)cyclobutyl)-N-((S)-1-(quinolin-6-yl) ethyl)-1H-benzo[d]imidazole-7-carboxamide: Molecular Formula: $C_{34}H_{30}N_6O_2$; yield 35%; **^{1}H NMR (600 MHz, $CDCl_3$) δ ppm** 9.23 (s, 1H), 8.80 (s, 1H), 8.18 – 8.15 (m, 2H), 8.07 – 7.88 (m, 4H), 7.84 (d, *J* = 8.6 Hz, 1H), 7.72 (s, 1H), 7.66 (t, *J* = 7.3 Hz, 1H), 7.51 (d, *J* = 7.4 Hz, 1H), 7.39 (dd, *J* = 8.2, 4.1 Hz, 1H), 7.34 (d, *J* = 7.7 Hz, 1H), 7.12 (s, 1H), 5.60 (d, *J* = 6.8 Hz, 1H), 5.41 (s, 1H), 5.28 (s, 1H), 2.57 (d, *J* = 4.6 Hz, 3H), 2.25 (s, 1H), 2.14 (s, 1H), 2.02 (s, 3H), 1.82 (d, *J* = 5.7 Hz, 3H). **^{13}C NMR (150 MHz, $CDCl_3$) δ ppm** 174.10, 167.70, 154.33, 150.31, 147.43, 144.90, 144.27, 141.71, 136.67, 134.21, 132.21, 129.88, 128.67, 128.36, 128.26, 125.29, 124.51, 123.21, 122.73, 122.30, 121.65, 51.73, 49.85, 33.70, 29.84, 26.47, 22.12. **HRMS (HESI-TOF)** m/z calcd for $(M + H)^+$ 555.2508, found 555.2510.

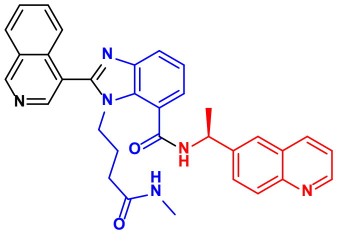

CDD-1908

(*S*)-2-(isoquinolin-4-yl)-1-(4-(methylamino)-4-oxobutyl)-N-(1-(quinolin-6-yl)ethyl)-1H-benzo [d]imidazole-7-carboxamide: Molecular Formula: $C_{33}H_{30}N_6O_2$; yield 31%; **^{1}H NMR (600 MHz, CDCl$_3$) δ ppm** 9.36 (s, 1H), 8.89 (d, *J* = 3.0 Hz, 1H), 8.65 (s, 1H), 8.22 (d, *J* = 8.1 Hz, 1H), 8.15 (d, *J* = 8.7 Hz, 1H), 8.08 (d, *J* = 8.0 Hz, 1H), 7.96 (d, *J* = 8.0 Hz, 1H), 7.92 (s, 1H), 7.85 (d, *J* = 8.7 Hz, 1H), 7.75 – 7.67 (m, 3H), 7.51 (d, *J* = 7.4 Hz, 1H), 7.43 (dd, *J* = 8.2, 4.2 Hz, 1H), 7.35 (t, *J* = 7.8 Hz, 1H), 6.88 (d, *J* = 7.1 Hz, 1H), 5.53 (d, *J* = 7.1 Hz, 1H), 5.20 (s, 1H), 4.20 (s, 2H), 2.49 (d, *J* = 4.7 Hz, 3H), 1.78 (d, *J* = 6.9 Hz, 3H), 1.75-1.71 (m, 1H), 1.57-1.47 (m, 3H). **^{13}C NMR (150 MHz, CDCl$_3$) δ ppm** 171.4, 167.3, 154.5, 151.8, 150.1, 144.9, 144.5, 136.7, 134.6, 132.1, 131.4, 129.8, 128.5, 128.2, 128.2, 125.0, 124.3, 123.1, 122.9, 122.1, 121.5, 49.8, 45.8, 32.5, 26.1, 26.0, 21.9. **HRMS (HESI-TOF)** m/z calcd for $(M + H)^+$ 543.2508, found 543.2499.

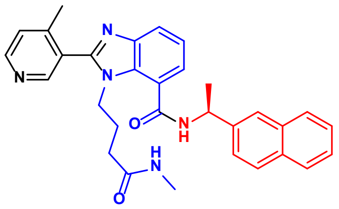

CDD-1924

(*S*)-1-(4-(methylamino)-4-oxobutyl)-2-(4-methylpyridin-3-yl)-N-(1-(naphthalen-2-yl)ethyl)-1H-benzo[d]imidazole-7-carboxamide: Molecular Formula: $C_{31}H_{31}N_5O_2$; yield 60%; **^{1}H NMR (600 MHz, CDCl$_3$) δ ppm** 8.57 (d, *J* = 4.5 Hz, 1H), 8.52 (s, 1H), 7.90 (d, *J* = 5.6 Hz, 2H), 7.85 (dd, *J* = 19.1, 9.8 Hz, 3H), 7.55 (d, *J* = 8.4 Hz, 1H), 7.52 – 7.47 (m, 2H), 7.45 (d, *J* = 7.4 Hz, 1H), 7.31 (t, *J* = 7.7 Hz, 2H), 6.74 (d, *J* = 6.8 Hz, 1H), 5.54 – 5.46 (m, 1H), 5.04 (s, 1H), 4.11 (dd, *J* = 13.9, 5.9 Hz, 1H), 4.06 (d, *J* = 7.1 Hz, 1H), 2.50 – 2.46 (m, 3H), 2.26 (s, 3H), 1.76–1.75 (m, 4H), 1.57–1.42 (m, 3H). **^{13}C NMR (150 MHz, CDCl$_3$) δ ppm** 171.6, 167.3, 152.2, 150.8, 150.4, 147.8, 144.8, 140.6, 133.5, 132.9, 131.2, 128.9, 128.0, 127.8, 127.0, 126.7, 126.3, 125.5, 125.2, 124.6, 123.0, 122.7, 122.1, 122.0, 50.1, 45.4, 32.5, 26.2, 26.1, 22.1, 19.5. **HRMS (HESI-TOF)** m/z calcd for $(M + H)^+$ 506.2556, found 506.2550.

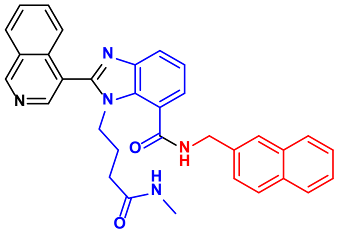

CDD-1925

2-(isoquinolin-4-yl)-1-(4-(methylamino)-4-oxobutyl)-N-(naphthalen-2-ylmethyl)-1H-benzo [d]imidazole-7-carboxamide: Molecular Formula: $C_{33}H_{29}N_5O_2$; yield 80%; **^{1}H NMR (600 MHz, CDCl$_3$) δ ppm** 9.30 (s, 1H), 8.53 (s, 1H), 8.04 (d, *J* = 8.0 Hz, 1H), 7.93 (d, *J* = 7.6 Hz, 1H), 7.88 – 7.81 (m, 4H), 7.75 – 7.66 (m, 3H), 7.56 – 7.45 (m, 4H), 7.32 (t, *J* = 7.8 Hz, 1H), 7.07 (t, *J* = 5.7 Hz, 1H), 5.24 (d, *J* = 4.0 Hz, 1H), 4.85 (d, *J* = 5.8 Hz, 2H), 4.26 (t, *J* = 6.9 Hz, 2H), 2.44 (d, *J* = 4.8 Hz, 3H), 1.74 – 1.69 (m, 2H), 1.61 (t, *J* = 7.6 Hz, 2H). **^{13}C NMR (150 MHz, CDCl$_3$) δ ppm** 171.6, 168.1, 154.6, 151.8, 144.9, 144.5, 135.4, 134.7, 133.5, 132.9, 132.1, 131.4, 128.9, 128.3, 128.2, 127.9, 127.9, 127.1, 126.6, 126.3, 126.2, 124.5, 123.0, 123.0, 122.3, 122.2, 122.0, 45.9, 44.7, 32.7, 26.3, 26.1. **HRMS (HESI-TOF)** m/z calcd for $(M + H)^+$ 528.2388, found 528.2387.

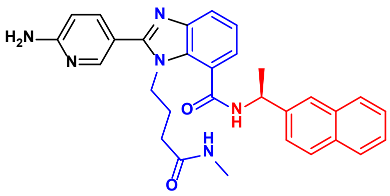

CDD-1926

(*S*)-2-(6-aminopyridin-3-yl)-1-(4-(methylamino)-4-oxobutyl)-N-(1-(naphthalen-2-yl)ethyl)-1H-benzo[d]imidazole-7-carboxamide: Molecular Formula: $C_{30}H_{30}N_6O_2$; yield 43%; **^{1}H NMR (600 MHz, CD_3OD) δ ppm** 8.22 (s, 1H), 7.93 (s, 1H), 7.90 – 7.82 (m, 3H), 7.79 (d, *J* = 7.8 Hz, 1H), 7.71 (d, *J* = 8.1 Hz, 1H), 7.61 (d, *J* = 8.1 Hz, 1H), 7.50 – 7.48 (m, 3H), 7.36 (t, *J* = 7.5 Hz, 1H), 6.69 (d, *J* = 8.5 Hz, 1H), 5.45 (d, *J* = 6.7 Hz, 1H), 4.29 – 4.26 (m, 2H), 2.48 (s, 3H), 1.69 (d, *J* = 6.6 Hz, 4H), 1.54 – 1.48 (m, 3H). **^{13}C NMR (150 MHz, CD_3OD) δ ppm** 174.4, 169.7, 161.9, 155.5, 149.8, 144.8, 142.6, 139.9, 134.9, 134.2, 132.4, 129.5, 128.9, 128.7, 127.3, 126.9, 126.0, 125.8, 124.4, 123.8, 123.4, 122.1, 115.5, 109.7, 51.2, 46.4, 32.8, 26.9, 26.2, 22.3. **HRMS (HESI-TOF)** m/z calcd for $(M + H)^+$ 507.2490, found 507.2498.

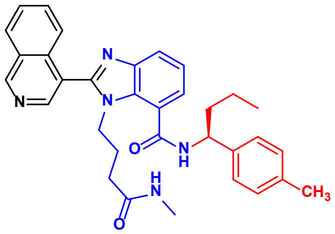

CDD-1934

(*S*)-2-(isoquinolin-4-yl)-1-(4-(methylamino)-4-oxobutyl)-N-(1-(p-tolyl)butyl)-1H-benzo[d] imidazole-7-carboxamide: Molecular Formula: $C_{33}H_{35}N_5O_2$; yield 73%; **^{1}H NMR (600 MHz, $CDCl_3$) δ ppm** 9.37 (s, 1H), 8.60 (s, 1H), 8.08 (d, *J* = 7.8 Hz, 1H), 7.93 (d, *J* = 8.0 Hz, 1H), 7.70 (dt, *J* = 12.2, 3.8 Hz, 3H), 7.44 (d, *J* = 7.4 Hz, 1H), 7.32 (t, *J* = 7.7 Hz, 1H), 7.29 – 7.26 (m, 3H), 7.15 (d, *J* = 7.8 Hz, 2H), 6.75 (t, *J* = 10.9 Hz, 1H), 5.39 (s, 1H), 5.13 (q, *J* = 7.6 Hz, 1H), 4.20 (dd, *J* = 13.7, 6.3 Hz, 1H), 4.10 (d, *J* = 7.1 Hz, 1H), 2.48 (d, *J* = 4.5 Hz, 3H), 2.32 (s, 3H), 2.00 – 1.93 (m, 1H), 1.89 – 1.85 (m, 2H), 1.68 (dd, *J* = 12.2, 5.5 Hz, 1H), 1.55 (d, *J* = 3.5 Hz, 3H), 1.51 – 1.40 (m, 1H), 1.42 – 1.33 (m, 1H), 0.97 (t, *J* = 7.3 Hz, 3H). **^{13}C NMR (150 MHz, $CDCl_3$) δ ppm** 171.7, 167.4, 154.6, 151.8, 145.0, 144.6, 139.3, 137.4, 134.7, 132.1, 131.5, 129.6, 128.3, 128.3, 126.8, 124.5, 123.0, 122.8, 122.5, 122.1, 54.2, 45.9, 38.6, 32.8, 26.3, 26.2, 21.2, 19.8, 14.0. **HRMS (HESI-TOF)** m/z calcd for $(M + H)^+$ 534.2869, found 534.2853.

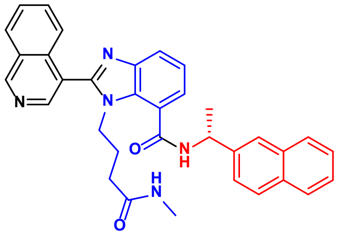

CDD-1935

(*R*)-2-(isoquinolin-4-yl)-1-(4-(methylamino)-4-oxobutyl)-N-(1-(naphthalen-2-yl)ethyl)-1H-benzo[d]imidazole-7-carboxamide: Molecular Formula: $C_{34}H_{31}N_5O_2$; yield 71%; **^{1}H NMR (600 MHz, $CDCl_3$) δ ppm** 9.36 (s, 1H), 8.62 (s, 1H), 8.06 (d, *J* = 7.9 Hz, 1H), 7.95 (d, *J* = 8.0 Hz, 1H), 7.91 (s, 1H), 7.87 – 7.80 (m, 3H), 7.72 – 7.70 (m, 2H), 7.68 (dd, *J* = 7.9, 2.8 Hz, 1H), 7.56 (dd, *J* = 8.4, 1.6 Hz, 1H), 7.49-7.48 (m, 3H), 7.33 (t, *J* = 7.8 Hz, 1H), 6.85 (d, *J* = 7.8 Hz, 1H), 5.51 (d, *J* = 7.0 Hz, 1H), 5.03 (s, 1H), 4.18 (dt, *J* = 14.5, 7.2 Hz, 2H), 2.41 (d, *J* = 4.7 Hz, 3H), 1.76 (d, *J* = 6.9 Hz, 5H), 1.70 (dd, *J* = 14.3, 6.9 Hz, 1H), 1.61 – 1.49 (m, 1H), 1.49 – 1.37 (m, 2H). **^{13}C NMR (150 MHz, $CDCl_3$) δ ppm** 171.4, 167.2, 144.9, 140.4, 134.6, 133.3, 132.8, 131.9, 131.4, 128.8, 128.2, 128.1, 127.9, 127.7, 126.5, 126.1, 125.1, 124.6, 124.4, 123.0, 122.8, 122.2, 122.0, 50.0, 45.7, 32.4, 26.0, 26.0, 21.9. **HRMS (HESI-TOF)** m/z calcd for $(M + H)^+$ 542.2556, found 542.2543.

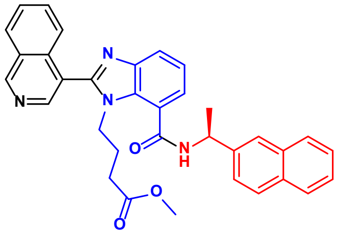

Methyl (*S*)-4-(2-(isoquinolin-4-yl)-7-((1-(naphthalen-2-yl)ethyl)carbamoyl)-1H-benzo [d] imidazol-1-yl)butanoate: Molecular Formula: $C_{34}H_{30}N_4O_3$; yield 53%; **^{1}H NMR (600 MHz, $CDCl_3$) δ ppm** 9.41 (s, 1H), 8.73 (s, 1H), 8.11 (d, *J* = 8.0 Hz, 1H), 8.00 (d, *J* = 8.0 Hz, 1H), 7.90 – 7.81 (m, 4H), 7.74 (d, *J* = 3.6 Hz, 2H), 7.71 (dd, *J* = 8.3, 3.9 Hz, 1H), 7.55 (d, *J* = 8.4 Hz, 1H), 7.51 – 7.45 (m, 3H), 7.37 (t, *J* = 7.8 Hz, 1H), 6.59 (d, *J* = 7.2 Hz, 1H), 5.56 – 5.49 (m, 1H), 4.29 (s, 2H), 3.30 (s, 2H), 1.76 (d, *J* = 6.9 Hz, 3H), 1.71 (d, *J* = 11.0 Hz, 2H), 1.62 – 1.56 (m, 2H). **^{13}C NMR (150 MHz, $CDCl_3$) δ ppm** 172.4, 167.1, 154.6, 152.0, 145.1, 144.7, 140.2, 134.8, 133.5, 133.0, 132.1, 131.5, 128.9, 128.3, 128.2, 128.0, 127.8, 126.6, 126.2, 125.1, 124.7, 124.5, 123.2, 122.8, 122.4, 122.2, 122.0, 51.6, 49.9, 45.6, 30.3, 25.5, 21.9. **LCMS** m/z calcd for $(M + H)^+$ 543.6, found 543.5.

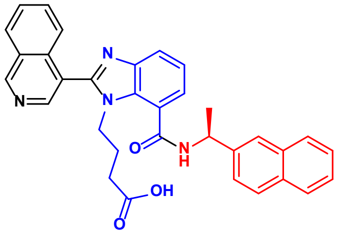

CDD-1972

(*S*)-4-(2-(isoquinolin-4-yl)-7-((1-(naphthalen-2-yl)ethyl)carbamoyl)-1H-benzo[d]imi dazol-1-yl)butanoic acid: Molecular Formula: $C_{33}H_{28}N_4O_3$; yield 11%; **^{1}H NMR (600 MHz, CD_3OD) δ ppm**

9.39 (s, 1H), 8.63 (s, 1H), 8.20 (d, *J* = 8.0 Hz, 1H), 7.88 (d, *J* = 8.2 Hz, 2H), 7.84 – 7.70 (m, 5H), 7.63 (d, *J* = 8.3 Hz, 1H), 7.57 (d, *J* = 7.6 Hz, 2H), 7.43 – 7.35 (m, 3H), 5.42 (q, *J* = 6.8 Hz, 1H), 4.09 (s, 2H), 3.27 (s, 2H), 1.65 (d, *J* = 7.0 Hz, 3H), 1.58 (d, *J* = 6.9 Hz, 2H), 1.50 – 1.46 (m, 2H). **^{13}C NMR (150 MHz, CD_3OD) δ ppm** 168.0, 154.5, 151.4, 143.7, 141.1, 134.6, 133.5, 132.8, 132.5, 130.8, 128.5, 128.4, 128.2, 127.5, 127.3, 125.9, 125.5, 124.6, 124.4, 123.7, 123.5, 122.6, 122.4, 121.9, 121.3, 49.8, 48.5, 45.3, 25.3, 20.9. **HRMS (HESI-TOF)** m/z calcd for $(M + H)^+$ 529.2240, found 529.2216.

Supplementary References:

1 Chamakuri, S. *et al.* DNA-encoded chemistry technology yields expedient access to SARS-CoV-2 M(pro) inhibitors. *Proc Natl Acad Sci U S A* **118**, doi:10.1073/pnas.2111172118 (2021).

2 Chamakuri, S. *et al.* Design and construction of a stereochemically diverse piperazine-based DNA-encoded chemical library. *Bioorg Med Chem* **48**, 116387, doi:10.1016/j.bmc.2021.116387 (2021).

3 Du, H. C. & Huang, H. DNA-Compatible Nitro Reduction and Synthesis of Benzimidazoles. *Bioconjug Chem* **28**, 2575-2580, doi:10.1021/acs.bioconjchem.7b00416 (2017).

4 Du, H. C., Chen, Y. C. & Huang, H. DNA-Compatible Nitro Reduction and Synthesis of Benzimidazoles. *Methods Mol Biol* **2541**, 67-73, doi:10.1007/978-1-0716-2545-3_11 (2022).

5 Du, H. C. *et al.* A Mild, DNA-Compatible Nitro Reduction Using B(2)(OH)(4). *Org Lett* **21**, 2194-2199, doi:10.1021/acs.orglett.9b00497 (2019).

6 Faver, J. C. *et al.* Quantitative Comparison of Enrichment from DNA-Encoded Chemical Library Selections. *ACS Comb Sci* **21**, 75-82, doi:10.1021/acscombsci.8b00116 (2019).
