# Supplementary material for: DNA-encoded chemical libraries yield non-covalent and non-peptidic SARS-CoV-2 main protease inhibitors
Source: Commun Chem. 2023 Aug 4;6:164. doi: 10.1038/s42004-023-00961-y (PMC10403511; doi:10.1038/s42004-023-00961-y)
Supplement: Supplementary file 2 — Description of Additional Supplementary Files [file 42004_2023_961_MOESM2_ESM.pdf]

Description of Additional Supplementary Files

File name: Supplementary Data 1

Description: Spectroscopic data for all newly synthesized small molecules.

File name: Supplementary Data 2

Description: Crystallography data of CDD-1733, CDD-1819, and CDD-1845.
