# Supplementary material for: DNA-encoded chemical libraries yield non-covalent and non-peptidic SARS-CoV-2 main protease inhibitors
Source: Commun Chem. 2023 Aug 4;6:164. doi: 10.1038/s42004-023-00961-y (PMC10403511; doi:10.1038/s42004-023-00961-y)

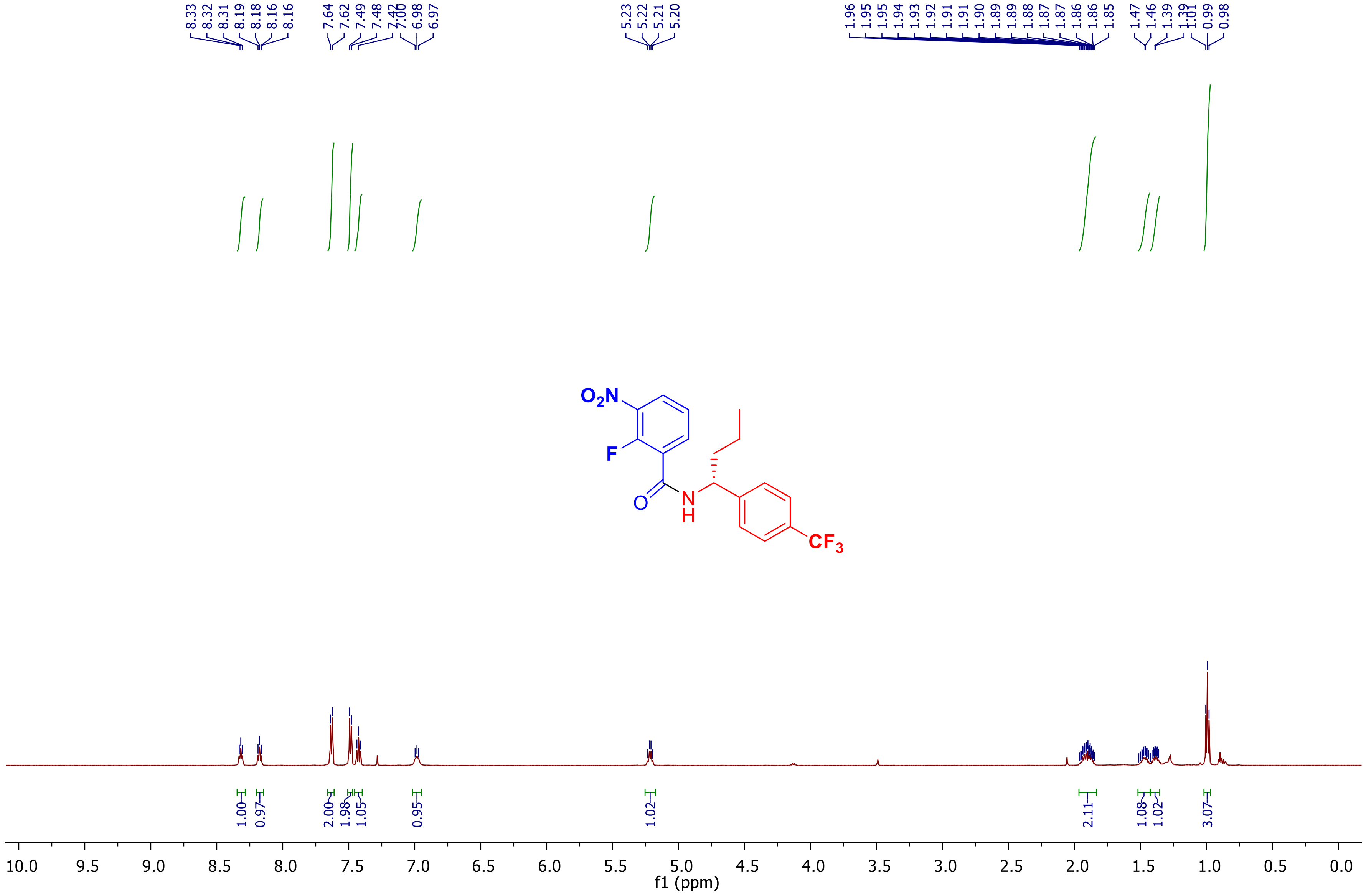
8.33 8.32 8.31 8.19 8.18 8.16 8.16 7.64 7.62 7.49 7.48 7.42 7.00 6.98 6.97
5.23 5.22 5.21 5.20
1.96 1.95 1.95 1.94 1.93 1.92 1.91 1.91 1.90 1.89 1.89 1.88 1.87 1.86 1.86 1.85 1.47 1.46 1.39 1.01 0.99 0.98
O2N
F
O
N
H
CF3
1.00 0.97 2.00 1.98 1.05 0.95 1.02 2.11 1.08 1.02 3.07
10.0 9.5 9.0 8.5 8.0 7.5 7.0 6.5 6.0 5.5 5.0 4.5 4.0 3.5 3.0 2.5 2.0 1.5 1.0 0.5 0.0
f1 (ppm)

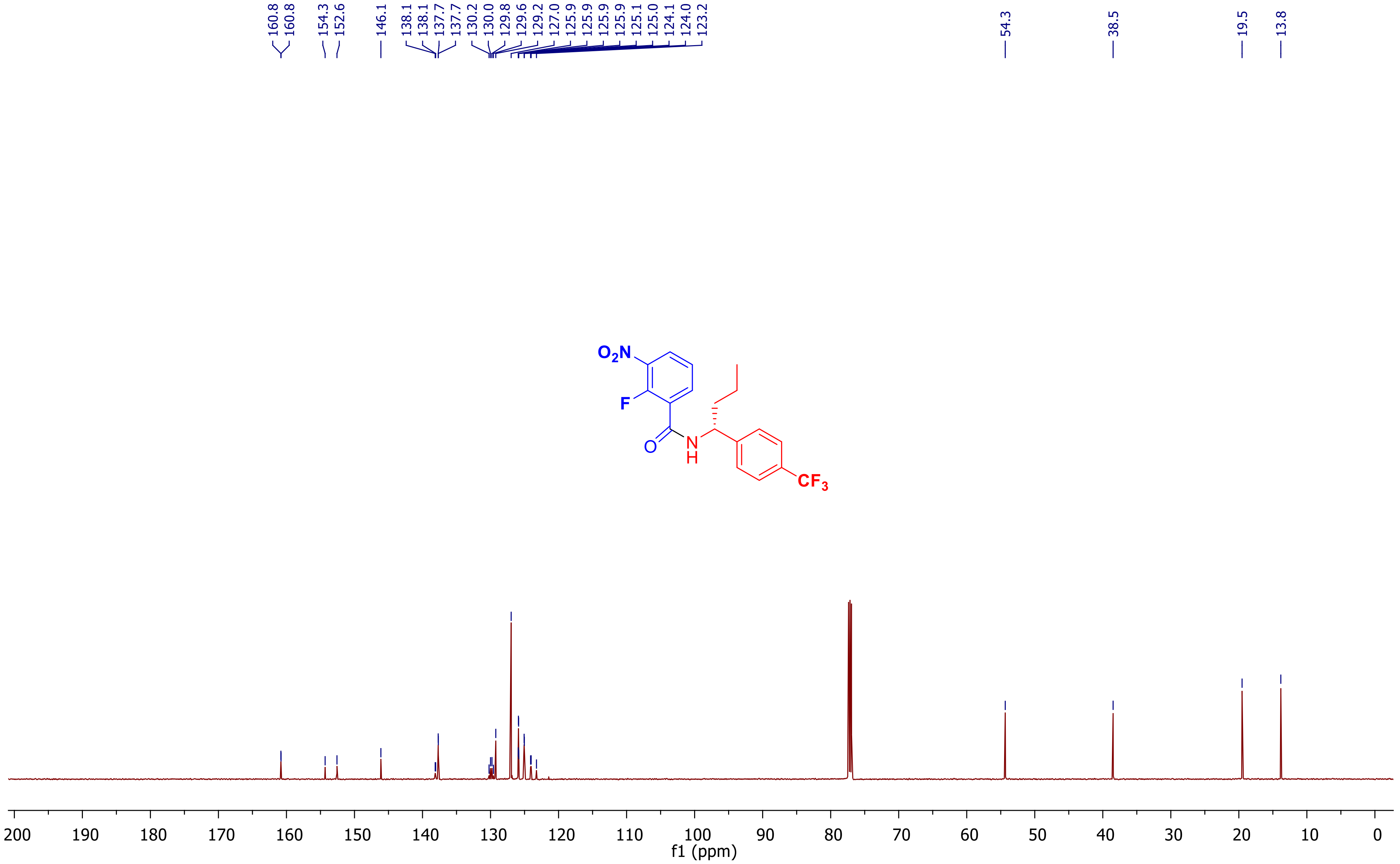
160.8
160.8
154.3
152.6
146.1
138.1
138.1
137.7
137.7
130.2
130.0
129.8
129.6
129.2
127.0
125.9
125.9
125.9
125.9
125.1
125.0
124.1
124.0
123.2
54.3
38.5
19.5
13.8
O_2N
F
O
N
H
CF_3
200
190
180
170
160
150
140
130
120
110
100
90
80
70
60
50
40
30
20
10
0
f1 (ppm)

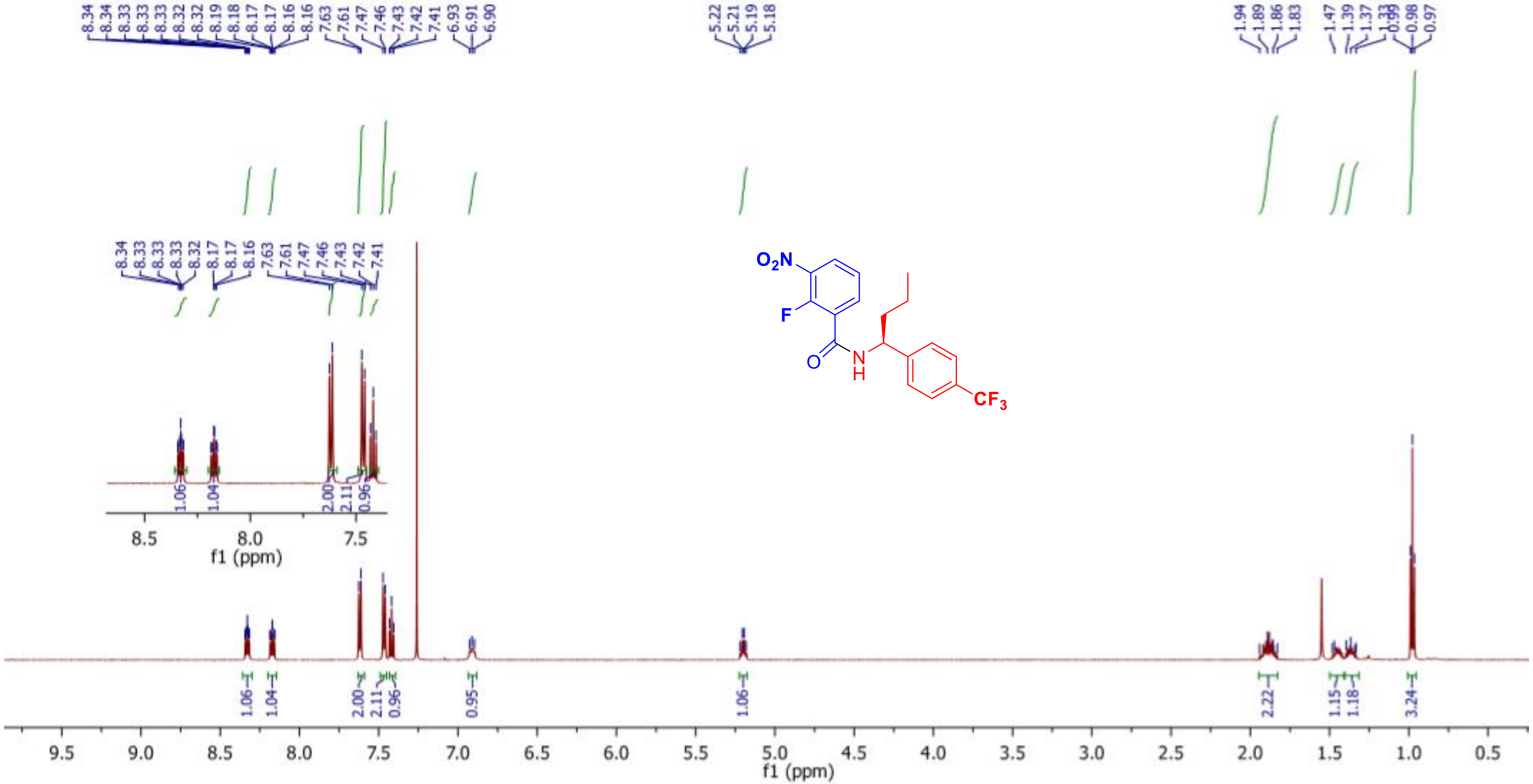
8.34 8.34 8.33 8.33 8.33 8.32 8.32 8.19 8.18 8.17 8.17 8.16 8.16 7.63 7.61 7.47 7.46 7.43 7.42 7.41 6.93 6.91 6.90
5.22 5.21 5.19 5.18
1.94 1.89 1.86 1.83 1.47 1.39 1.37 1.33 0.99 0.98 0.97
8.34 8.33 8.33 8.33 8.32 8.17 8.17 8.16 7.63 7.61 7.47 7.46 7.43 7.42 7.41
1.06 1.04 2.00 2.11 0.96
8.5 8.0 7.5
f1 (ppm)
O_2N
F
O
N
H
CF_3
1.06 1.04 2.00 2.11 0.96 0.95 1.06 2.22 1.15 1.18 3.24
9.5 9.0 8.5 8.0 7.5 7.0 6.5 6.0 5.5 5.0 4.5 4.0 3.5 3.0 2.5 2.0 1.5 1.0 0.5
f1 (ppm)

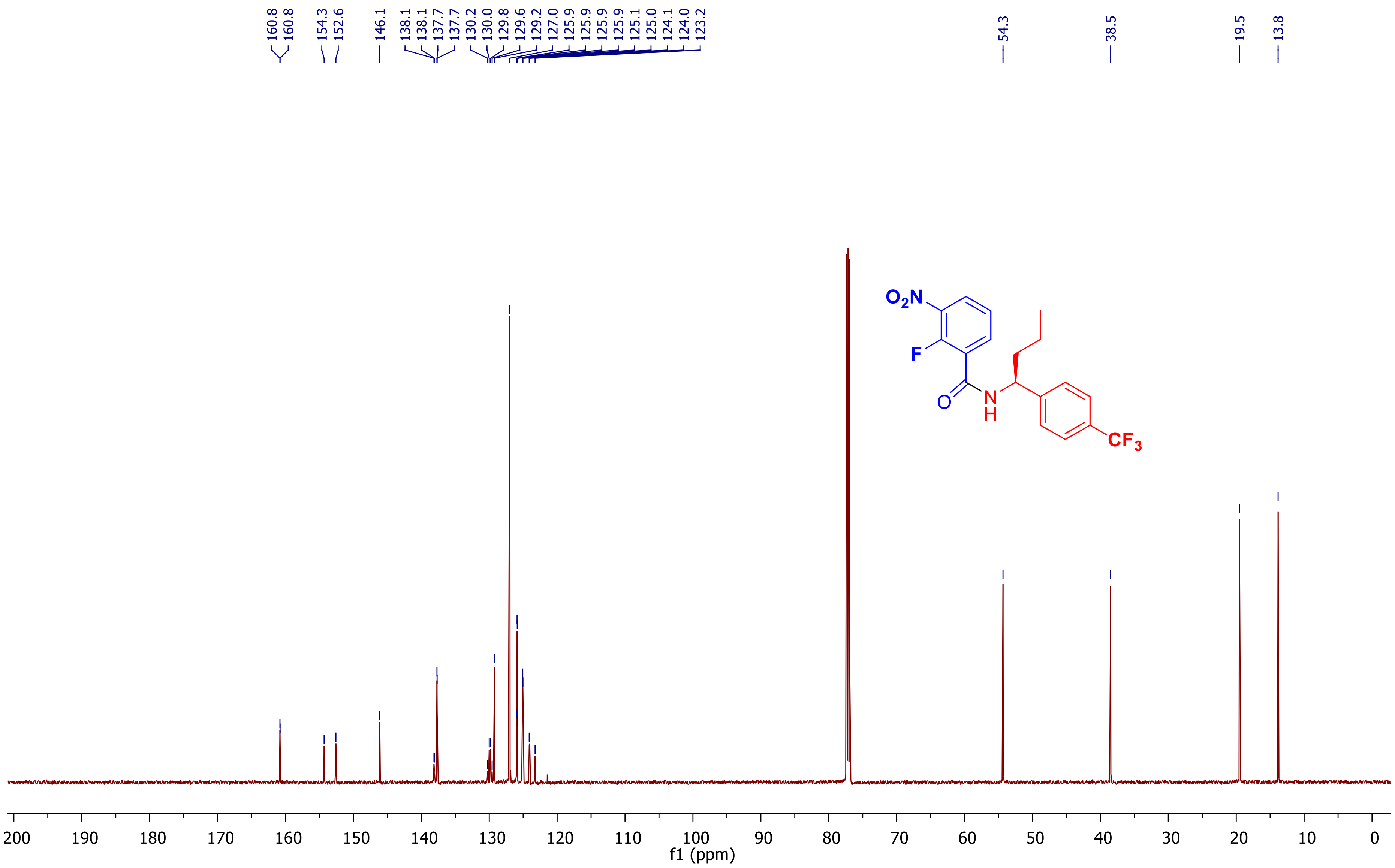
160.8
160.8
154.3
152.6
146.1
138.1
138.1
137.7
137.7
130.2
130.0
129.8
129.6
129.2
127.0
125.9
125.9
125.9
125.9
125.1
125.0
124.1
124.0
123.2
54.3
38.5
19.5
13.8
O2N
F
O
N
H
CF3
200 190 180 170 160 150 140 130 120 110 100 90 80 70 60 50 40 30 20 10 0
f1 (ppm)

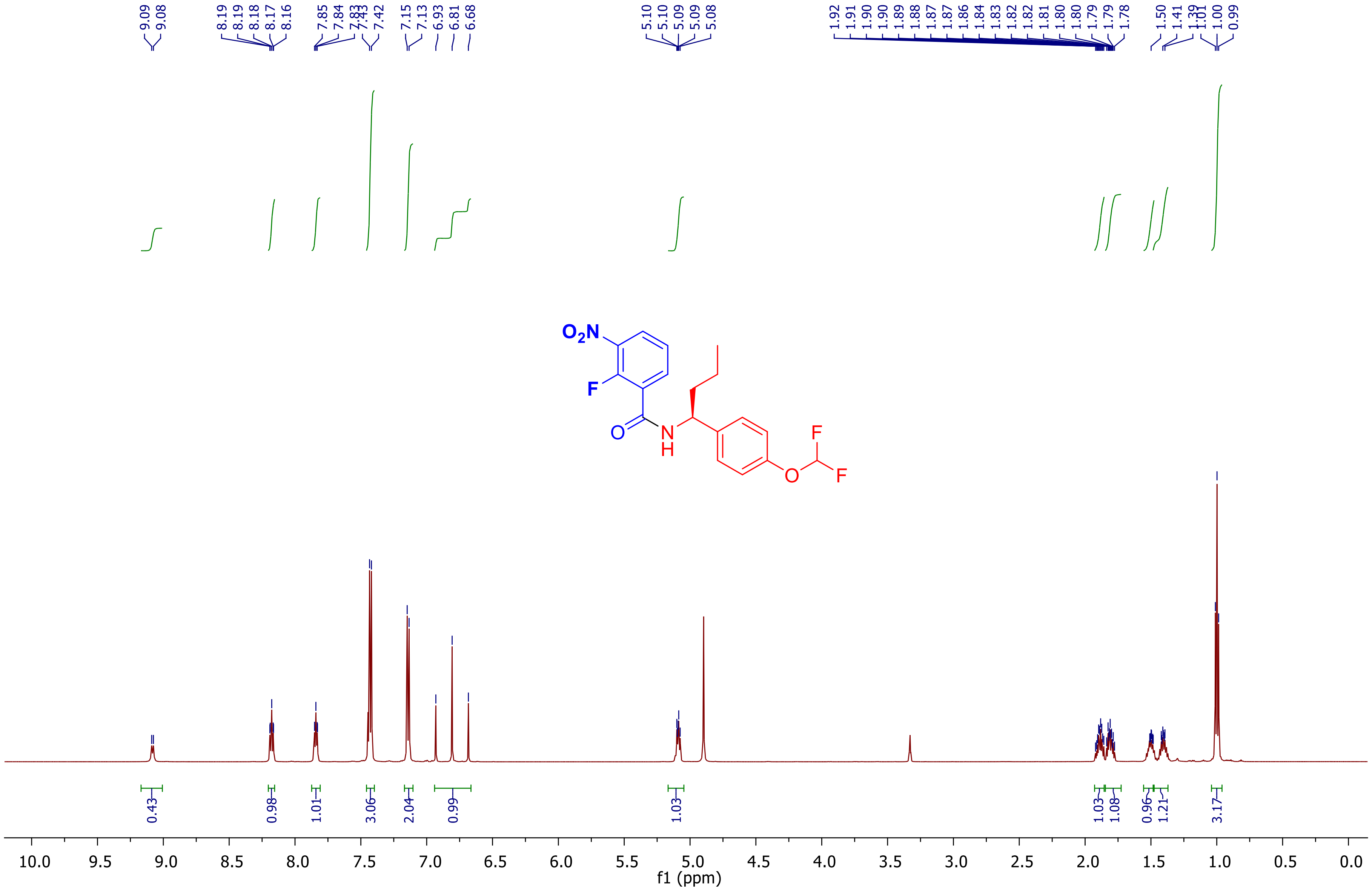
9.09
9.08
8.19
8.19
8.18
8.17
8.16
7.85
7.84
7.83
7.43
7.42
7.15
7.13
6.93
6.81
6.68
5.10
5.10
5.09
5.09
5.08
1.92
1.91
1.90
1.90
1.89
1.88
1.87
1.87
1.86
1.84
1.83
1.82
1.82
1.81
1.80
1.80
1.79
1.79
1.78
1.50
1.41
1.39
1.01
1.00
0.99
O_2N
F
O
N
H
F
O
F
0.43
0.98
1.01
3.06
2.04
0.99
1.03
1.03
1.08
0.96
1.21
3.17
10.0
9.5
9.0
8.5
8.0
7.5
7.0
6.5
6.0
5.5
5.0
4.5
4.0
3.5
3.0
2.5
2.0
1.5
1.0
0.5
0.0
f1 (ppm)

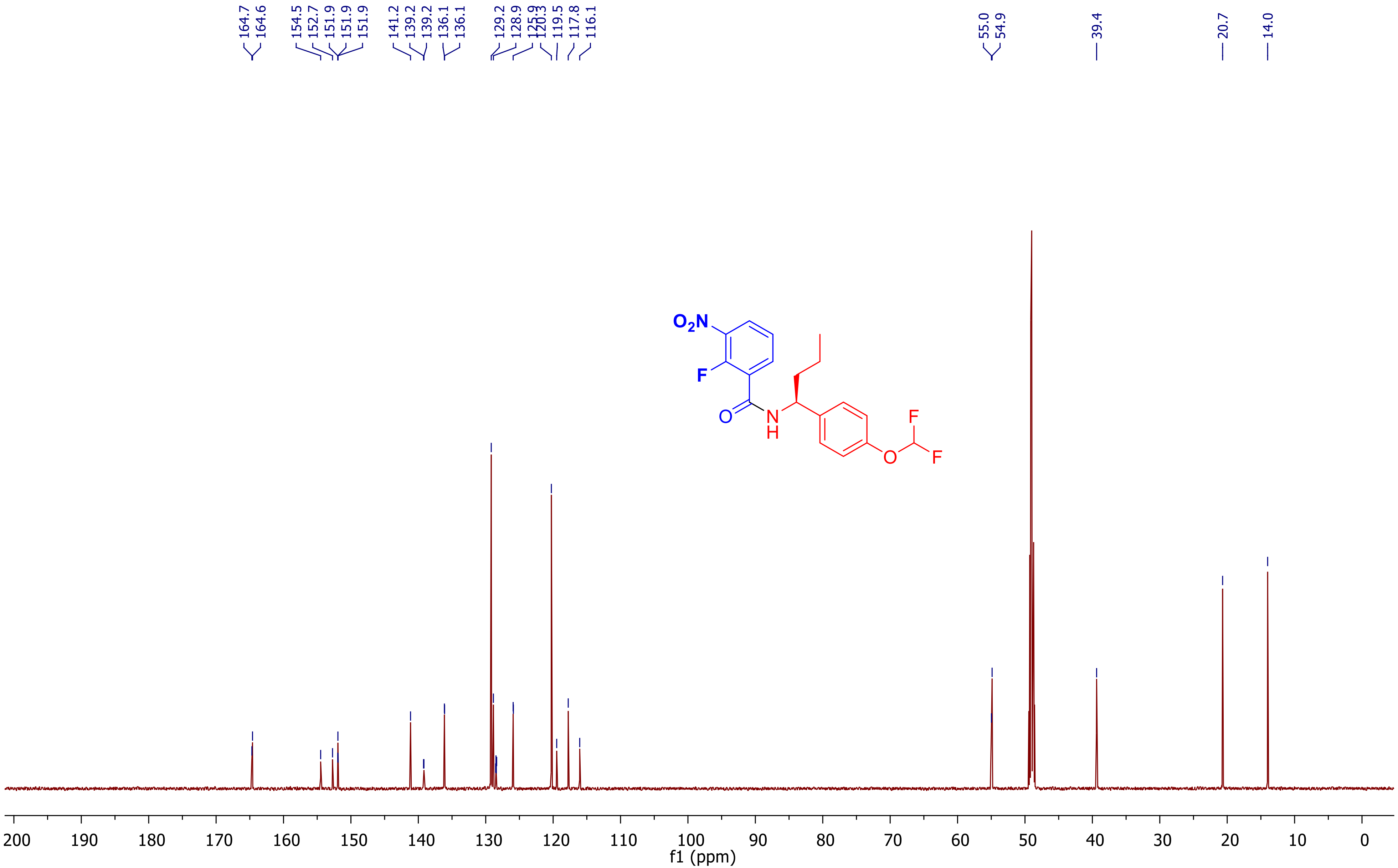
164.7
164.6
154.5
152.7
151.9
151.9
151.9
141.2
139.2
139.2
136.1
136.1
129.2
128.9
125.9
120.3
119.5
117.8
116.1
55.0
54.9
39.4
20.7
14.0
O2N
F
O
N
H
F
O
F
200 190 180 170 160 150 140 130 120 110 100 90 80 70 60 50 40 30 20 10 0
f1 (ppm)

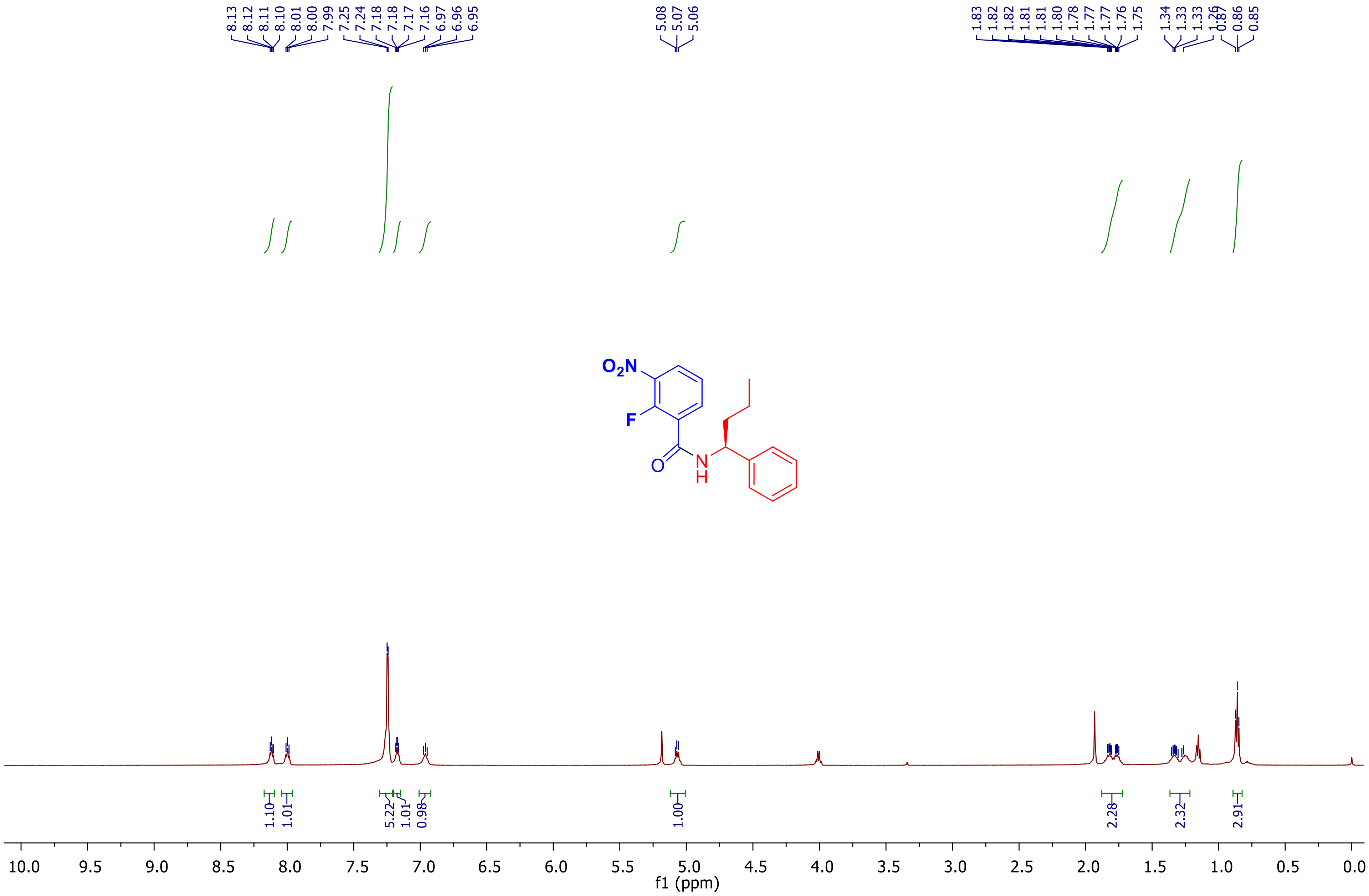
8.13 8.12 8.11 8.10 8.01 8.00 7.99 7.25 7.24 7.18 7.18 7.17 7.16 6.97 6.96 6.95
5.08 5.07 5.06
1.83 1.82 1.82 1.81 1.81 1.80 1.78 1.77 1.77 1.76 1.75 1.34 1.33 1.33 1.26 0.87 0.86 0.85
O2N
F
O
N
H
1.10 1.01 5.22 1.01 0.98 1.00 2.28 2.32 2.91
10.0 9.5 9.0 8.5 8.0 7.5 7.0 6.5 6.0 5.5 5.0 4.5 4.0 3.5 3.0 2.5 2.0 1.5 1.0 0.5 0.0
f1 (ppm)

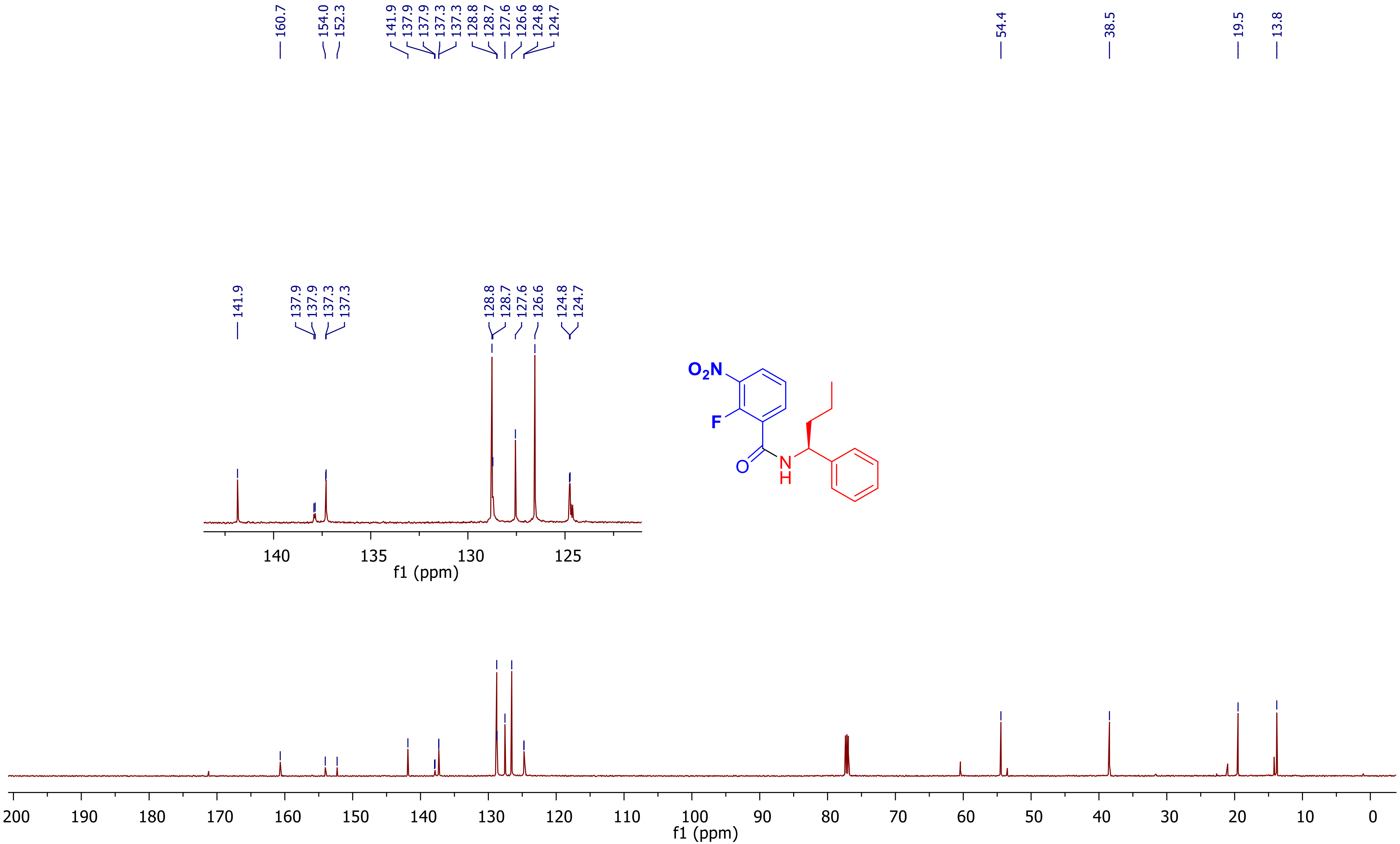

160.7
154.0
152.3
141.9
137.9
137.9
137.3
137.3
128.8
128.7
127.6
126.6
124.8
124.7
54.4
38.5
19.5
13.8
O2N
F
O
N
H
140
135
130
125
f1 (ppm)
200
190
180
170
160
150
140
130
120
110
100
90
80
70
60
50
40
30
20
10
0
f1 (ppm)

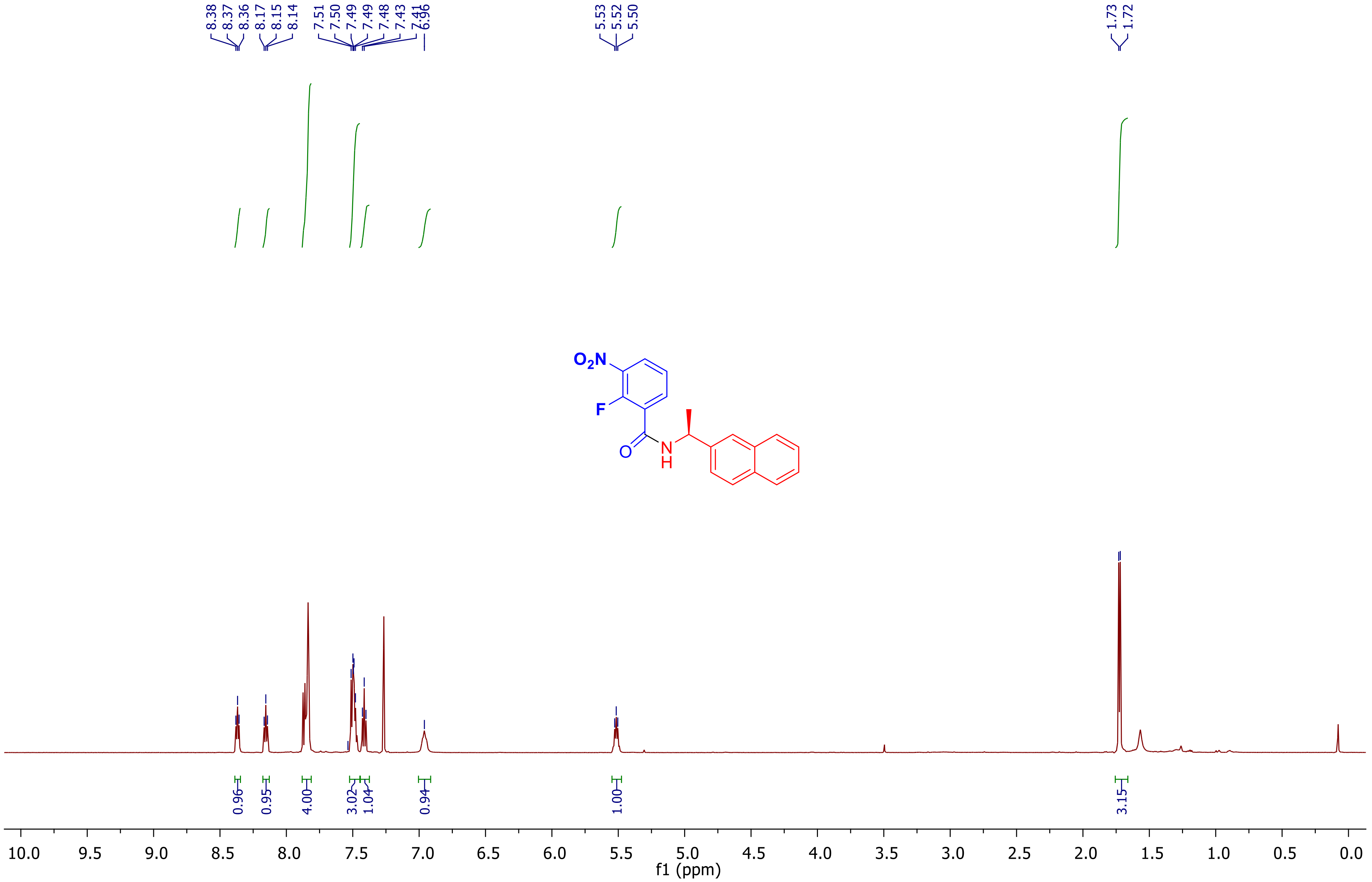
8.38
8.37
8.36
8.17
8.15
8.14
7.51
7.50
7.49
7.49
7.48
7.43
7.41
6.96
5.53
5.52
5.50
1.73
1.72
O2N
F
O
N
H
0.96
0.95
4.00
3.02
1.04
0.94
1.00
3.15
10.0
9.5
9.0
8.5
8.0
7.5
7.0
6.5
6.0
5.5
5.0
4.5
4.0
3.5
3.0
2.5
2.0
1.5
1.0
0.5
0.0
f1 (ppm)

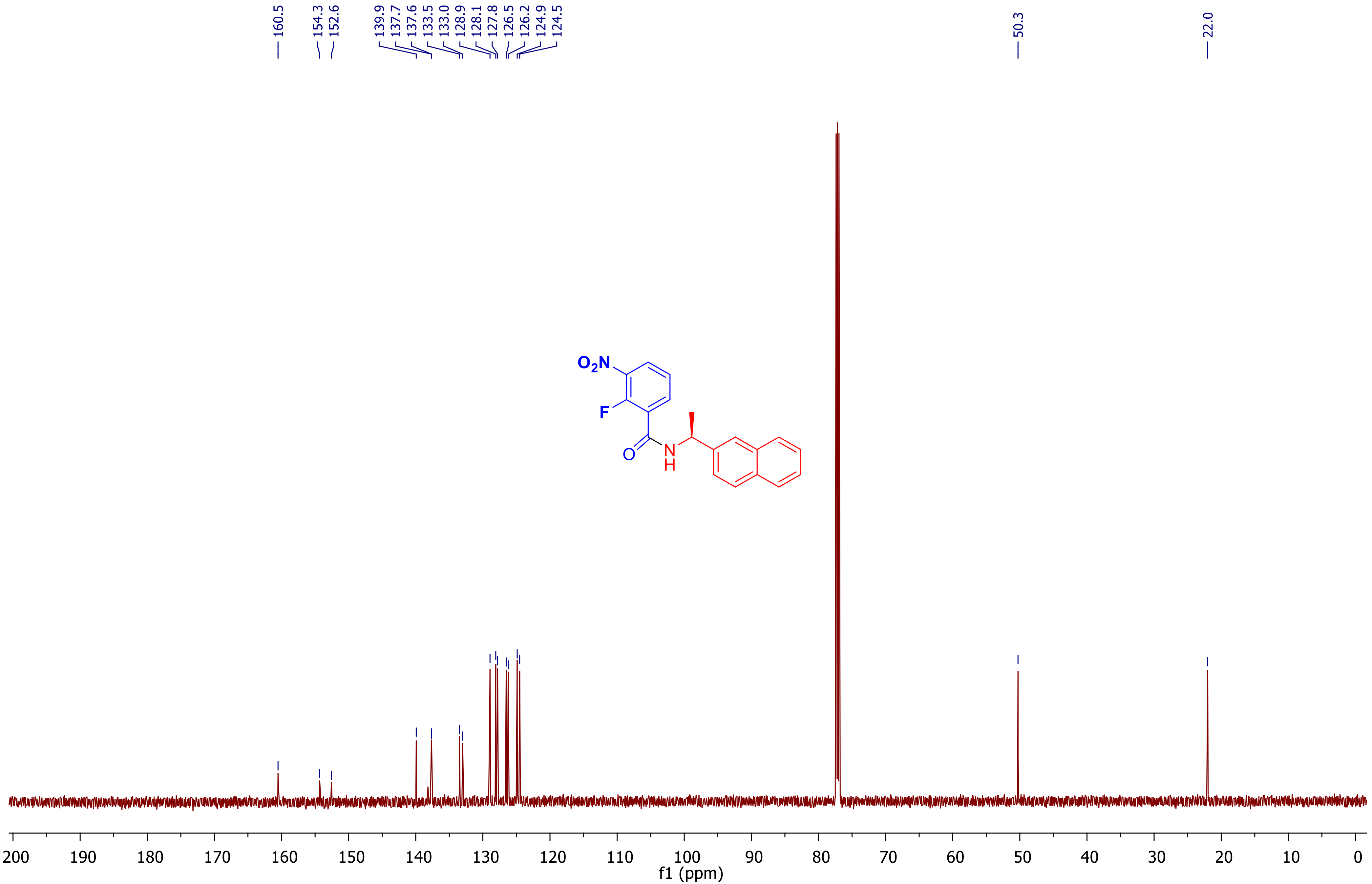

160.5
154.3
152.6
139.9
137.7
137.6
133.5
133.0
128.9
128.1
127.8
126.5
126.2
124.9
124.5
50.3
22.0
O2N
F
O
N
H
200
190
180
170
160
150
140
130
120
110
100
90
80
70
60
50
40
30
20
10
0
f1 (ppm)

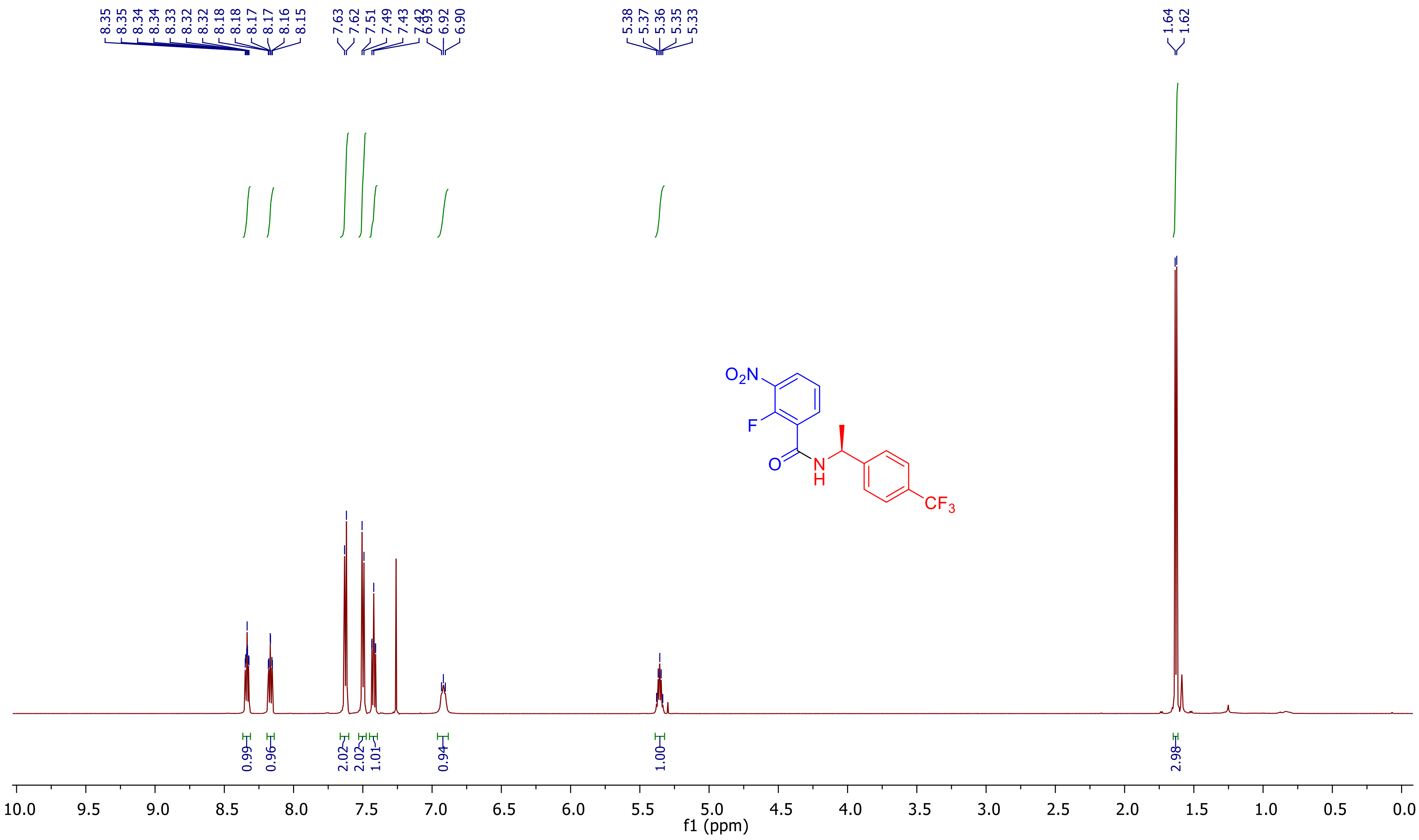

8.35
8.35
8.34
8.34
8.33
8.32
8.32
8.18
8.18
8.17
8.17
8.16
8.15
7.63
7.62
7.51
7.49
7.43
7.42
6.93
6.92
6.90
5.38
5.37
5.36
5.35
5.33
1.64
1.62
0.99
0.96
2.02
2.02
1.01
0.94
1.00
2.98
10.0
9.5
9.0
8.5
8.0
7.5
7.0
6.5
6.0
5.5
5.0
4.5
4.0
3.5
3.0
2.5
2.0
1.5
1.0
0.5
0.0
f1 (ppm)
O2N
F
O
N
H
CF3

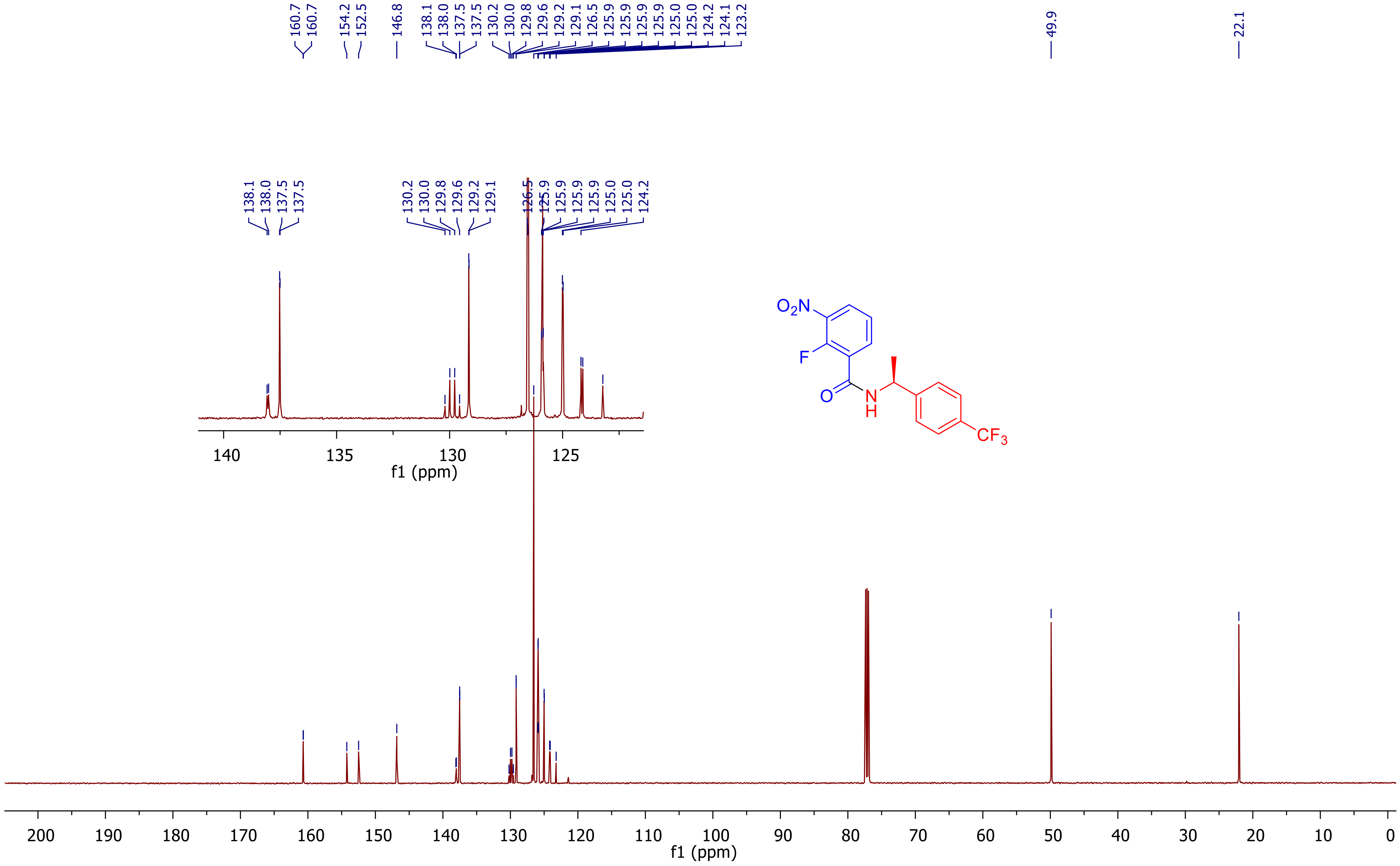
160.7
160.7
154.2
152.5
146.8
138.1
138.0
137.5
137.5
130.2
130.0
129.8
129.6
129.2
129.1
126.5
125.9
125.9
125.9
125.9
125.0
125.0
124.2
124.1
123.2
49.9
22.1
O2N
F
O
N
H
CF3
f1 (ppm)
f1 (ppm)

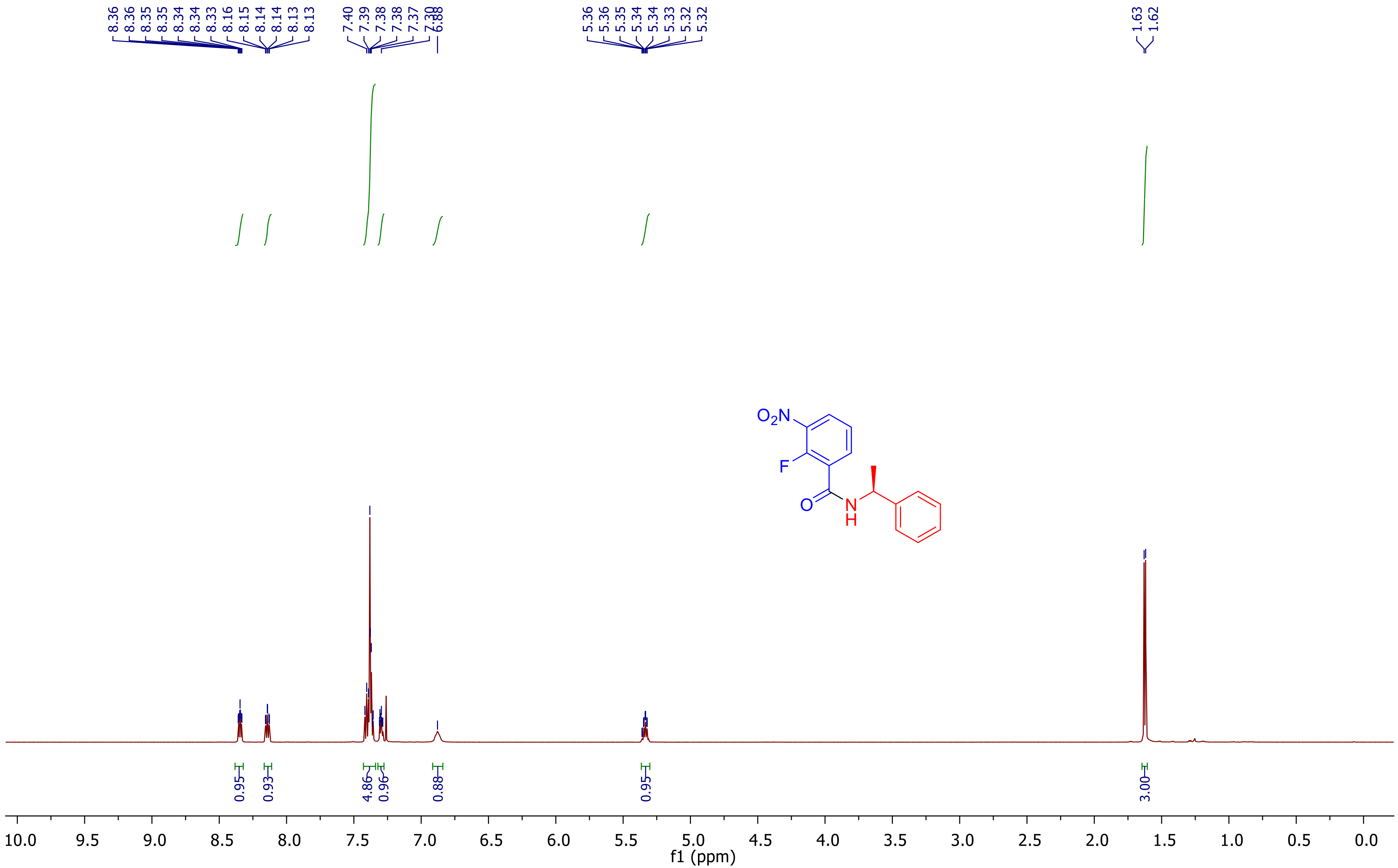
8.36
8.36
8.35
8.35
8.34
8.34
8.33
8.16
8.15
8.14
8.14
8.13
8.13
7.40
7.39
7.38
7.38
7.37
7.30
6.88
5.36
5.36
5.35
5.34
5.34
5.33
5.32
5.32
1.63
1.62
O_2N
F
O
N
H
0.95
0.93
4.86
0.96
0.88
0.95
3.00
10.0
9.5
9.0
8.5
8.0
7.5
7.0
6.5
6.0
5.5
5.0
4.5
4.0
3.5
3.0
2.5
2.0
1.5
1.0
0.5
0.0
f1 (ppm)

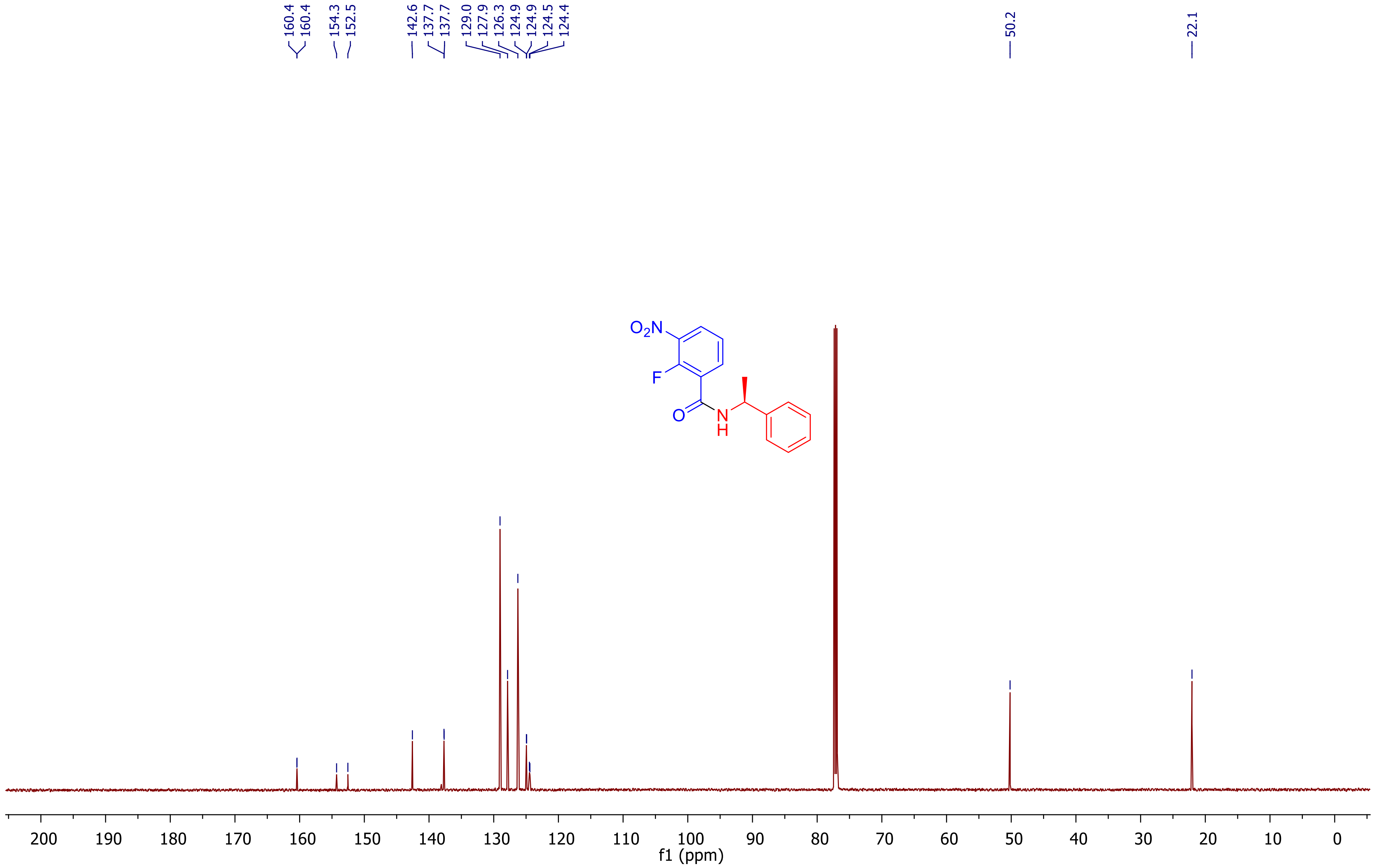
160.4
160.4
154.3
152.5
142.6
137.7
137.7
129.0
127.9
126.3
124.9
124.9
124.5
124.4
50.2
22.1
O2N
F
O
N
H
200 190 180 170 160 150 140 130 120 110 100 90 80 70 60 50 40 30 20 10 0
f1 (ppm)

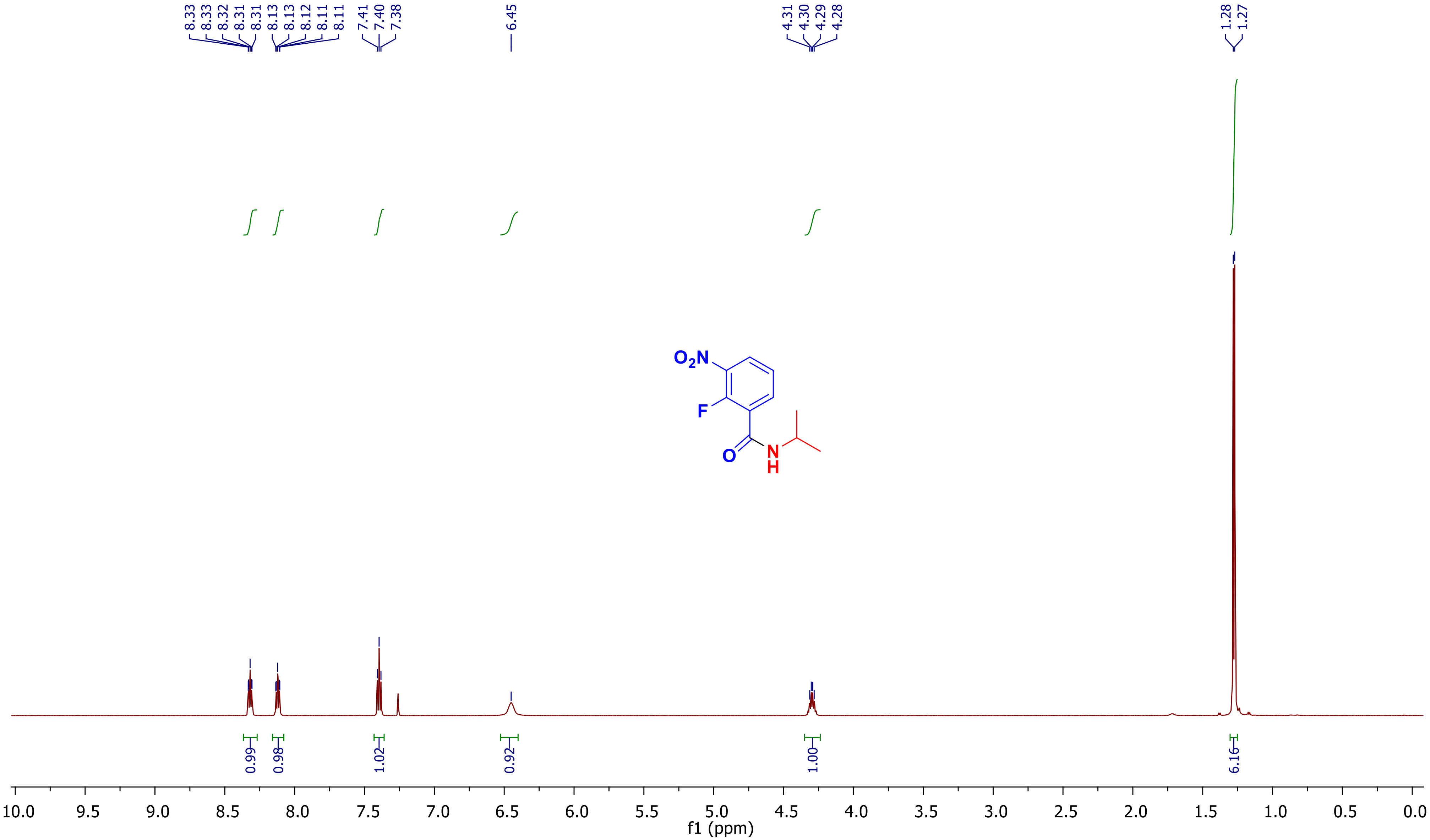
8.33
8.33
8.32
8.31
8.31
8.13
8.13
8.12
8.11
8.11
7.41
7.40
7.38
6.45
4.31
4.30
4.29
4.28
1.28
1.27
O2N
F
O
N
H
0.99
0.98
1.02
0.92
1.00
6.16
10.0 9.5 9.0 8.5 8.0 7.5 7.0 6.5 6.0 5.5 5.0 4.5 4.0 3.5 3.0 2.5 2.0 1.5 1.0 0.5 0.0
f1 (ppm)

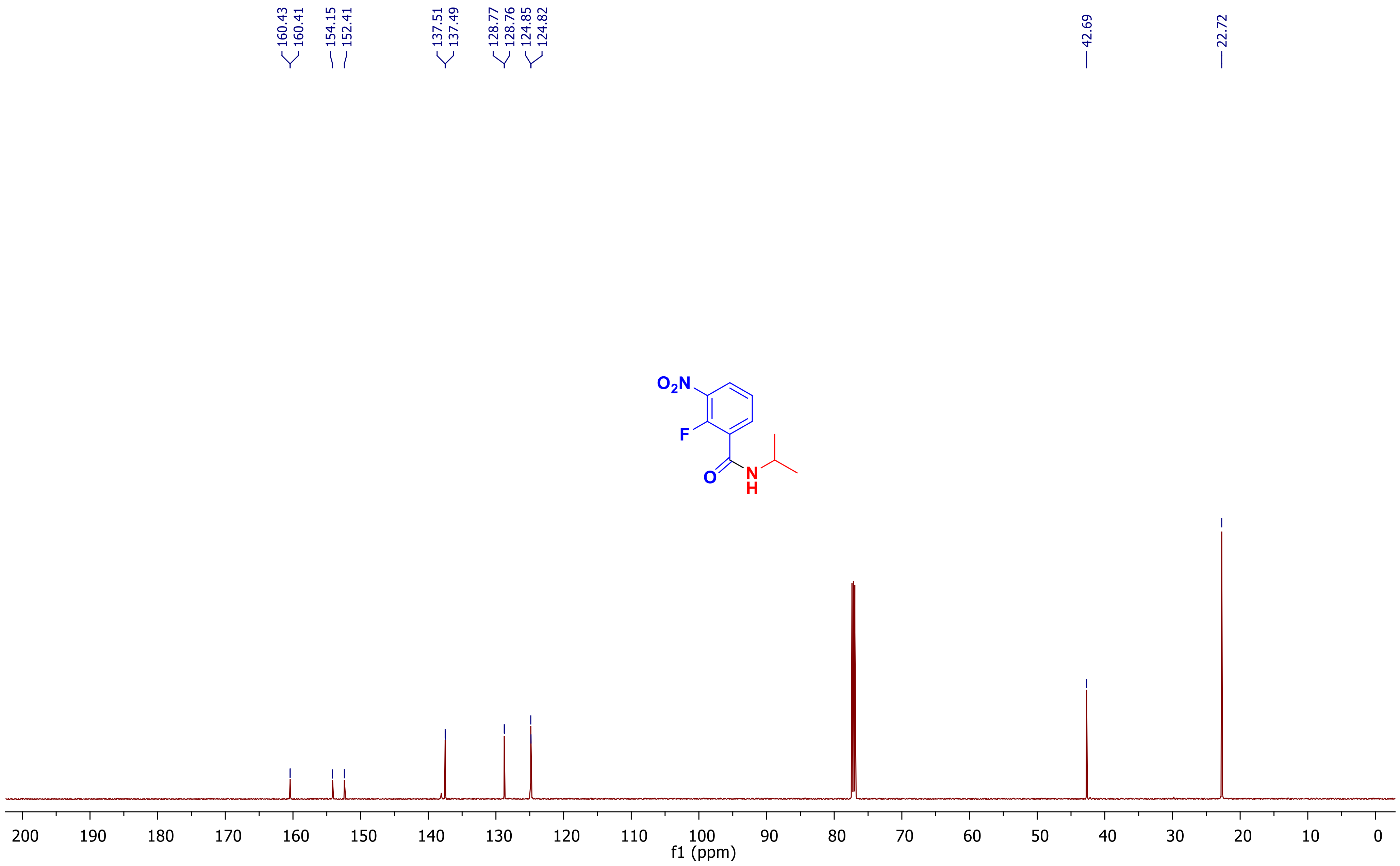
160.43
160.41
154.15
152.41
137.51
137.49
128.77
128.76
124.85
124.82
42.69
22.72
O2N
F
O
N
H
200
190
180
170
160
150
140
130
120
110
100
90
80
70
60
50
40
30
20
10
0
f1 (ppm)

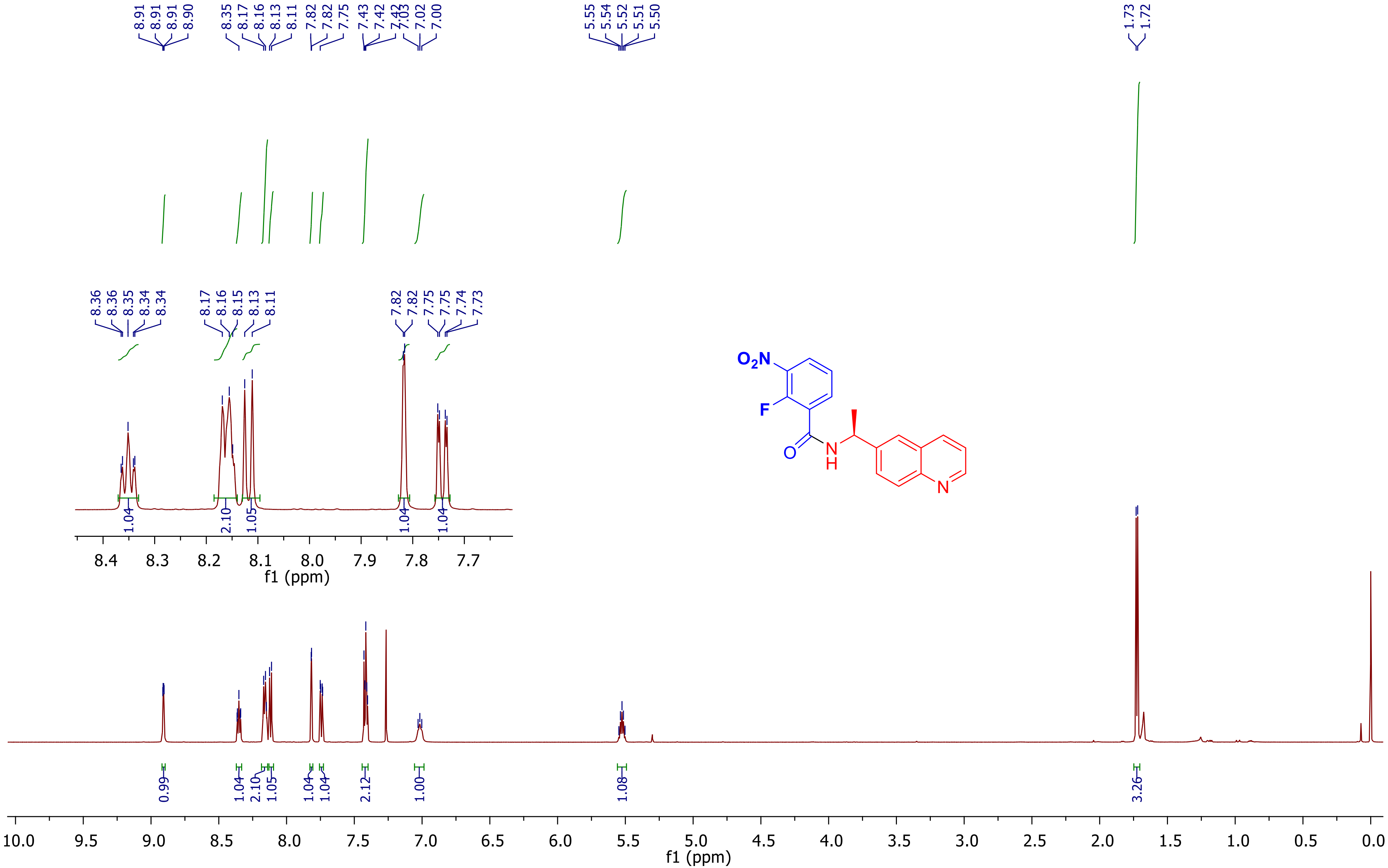
8.91 8.91 8.91 8.90 8.35 8.17 8.16 8.13 8.11 7.82 7.82 7.75 7.43 7.42 7.42 7.03 7.02 7.00
5.55 5.54 5.52 5.51 5.50
1.73 1.72
8.36 8.36 8.35 8.34 8.34 8.17 8.16 8.15 8.13 8.11 7.82 7.82 7.75 7.75 7.74 7.73
1.04 2.10 1.05 1.04 1.04
8.4 8.3 8.2 8.1 8.0 7.9 7.8 7.7
f1 (ppm)
O2N
F
O
N
H
N
0.99 1.04 2.10 1.05 1.04 1.04 2.12 1.00 1.08 3.26
10.0 9.5 9.0 8.5 8.0 7.5 7.0 6.5 6.0 5.5 5.0 4.5 4.0 3.5 3.0 2.5 2.0 1.5 1.0 0.5 0.0
f1 (ppm)

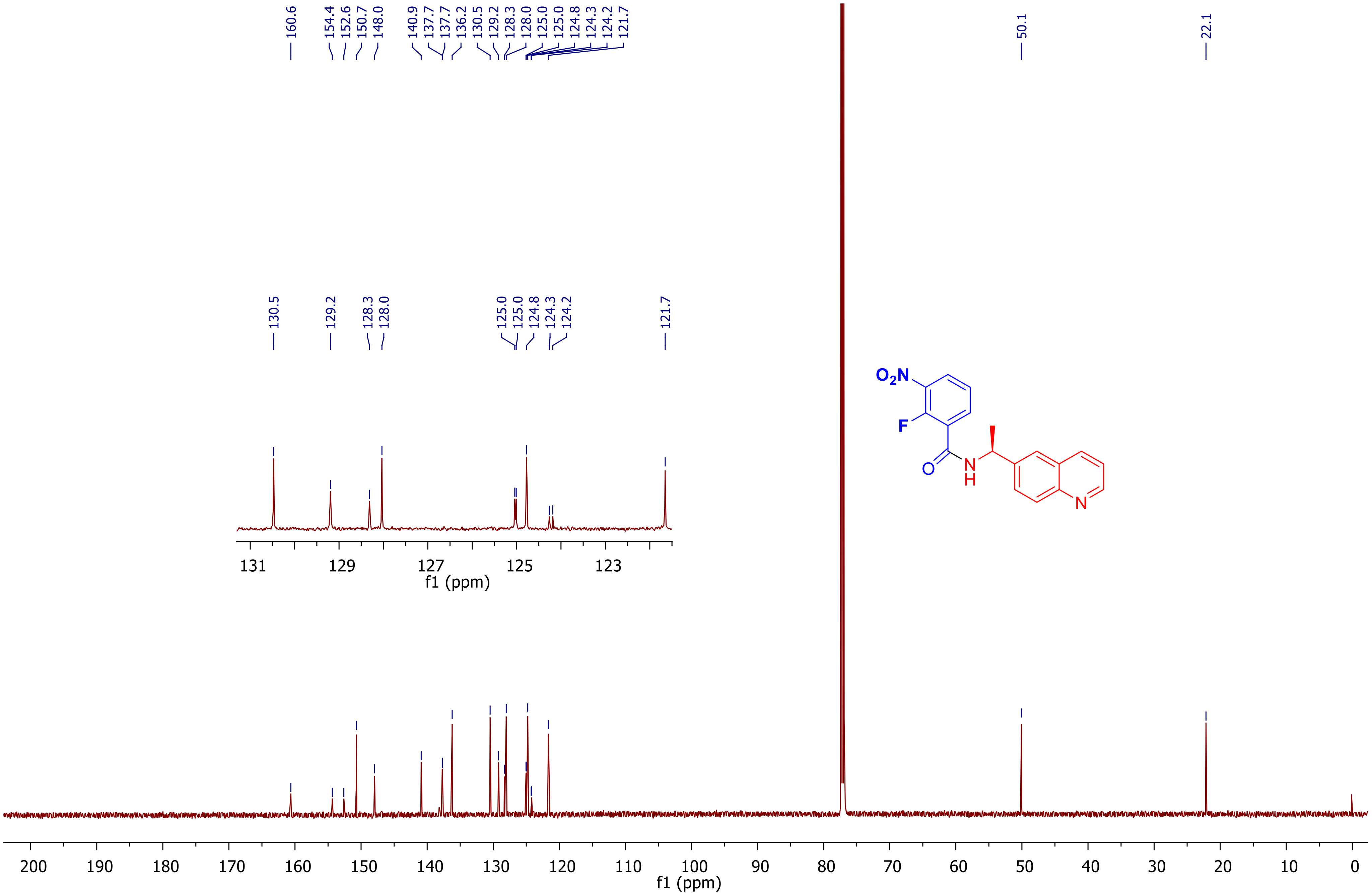
160.6
154.4
152.6
150.7
148.0
140.9
137.7
137.7
136.2
130.5
129.2
128.3
128.0
125.0
125.0
124.8
124.3
124.2
121.7
50.1
22.1
130.5
129.2
128.3
128.0
125.0
125.0
124.8
124.3
124.2
121.7
131
129
127
125
123
f1 (ppm)
O_2N
F
O
N
H
N
200
190
180
170
160
150
140
130
120
110
100
90
80
70
60
50
40
30
20
10
0
f1 (ppm)

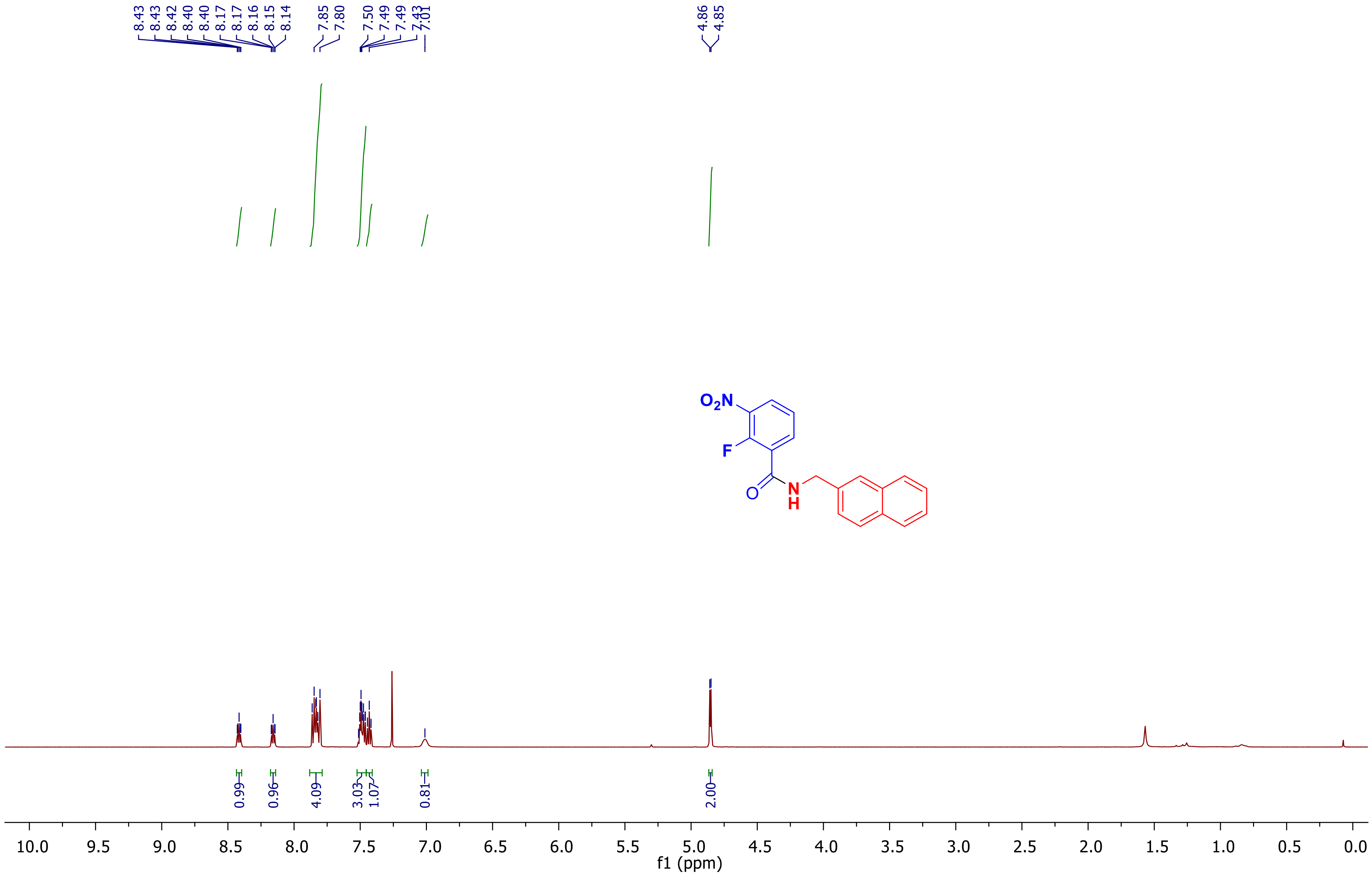
8.43
8.43
8.42
8.40
8.40
8.17
8.17
8.16
8.15
8.14
7.85
7.80
7.50
7.49
7.49
7.43
7.01
4.86
4.85
O2N
F
O
N
H
0.99
0.96
4.09
3.03
1.07
0.81
2.00
10.0
9.5
9.0
8.5
8.0
7.5
7.0
6.5
6.0
5.5
5.0
4.5
4.0
3.5
3.0
2.5
2.0
1.5
1.0
0.5
0.0
f1 (ppm)

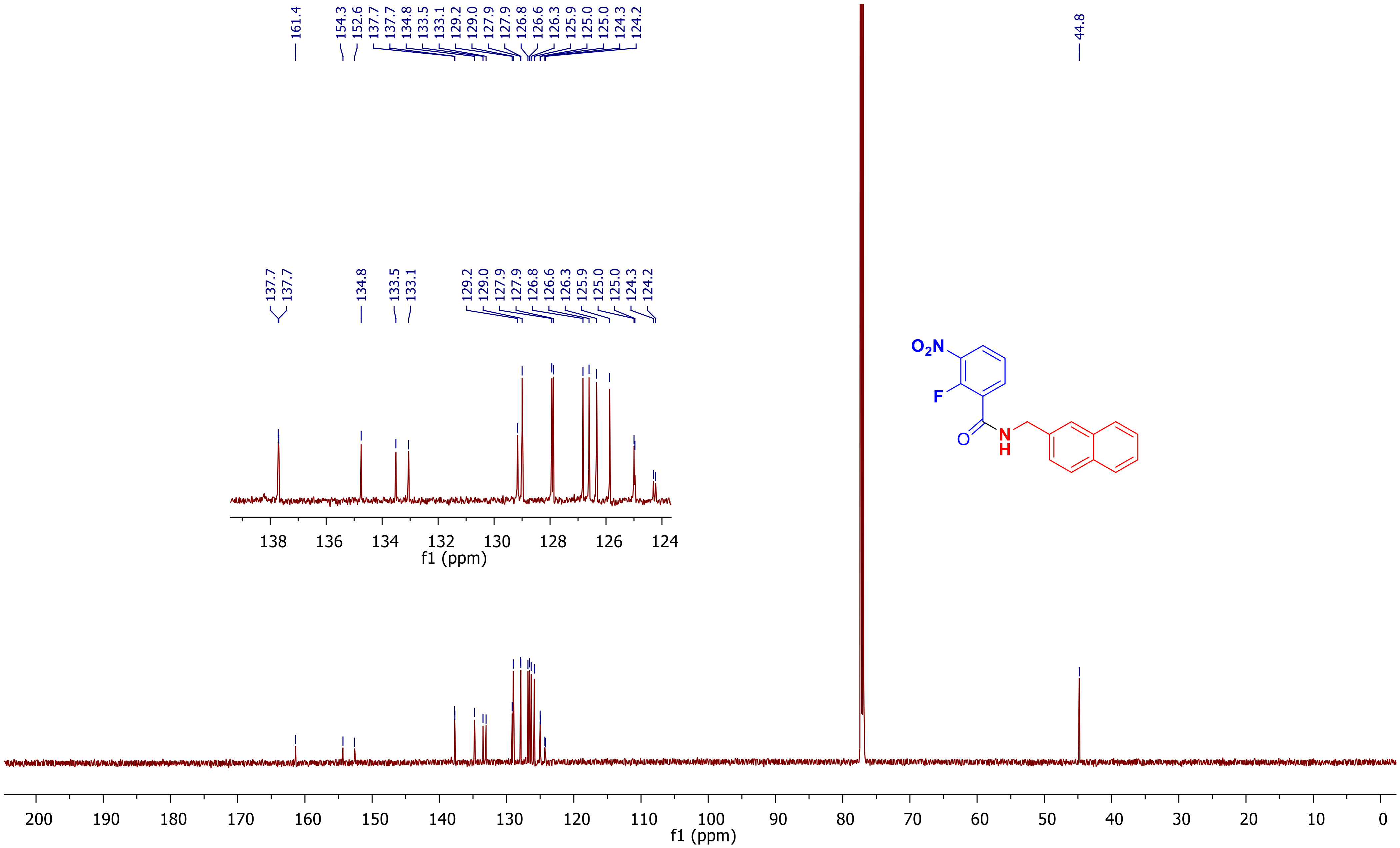
161.4
154.3
152.6
137.7
137.7
134.8
133.5
133.1
129.2
129.0
127.9
127.9
126.8
126.6
126.3
125.9
125.0
125.0
124.3
124.2
44.8
f1 (ppm)
O2N
F
O
N
H

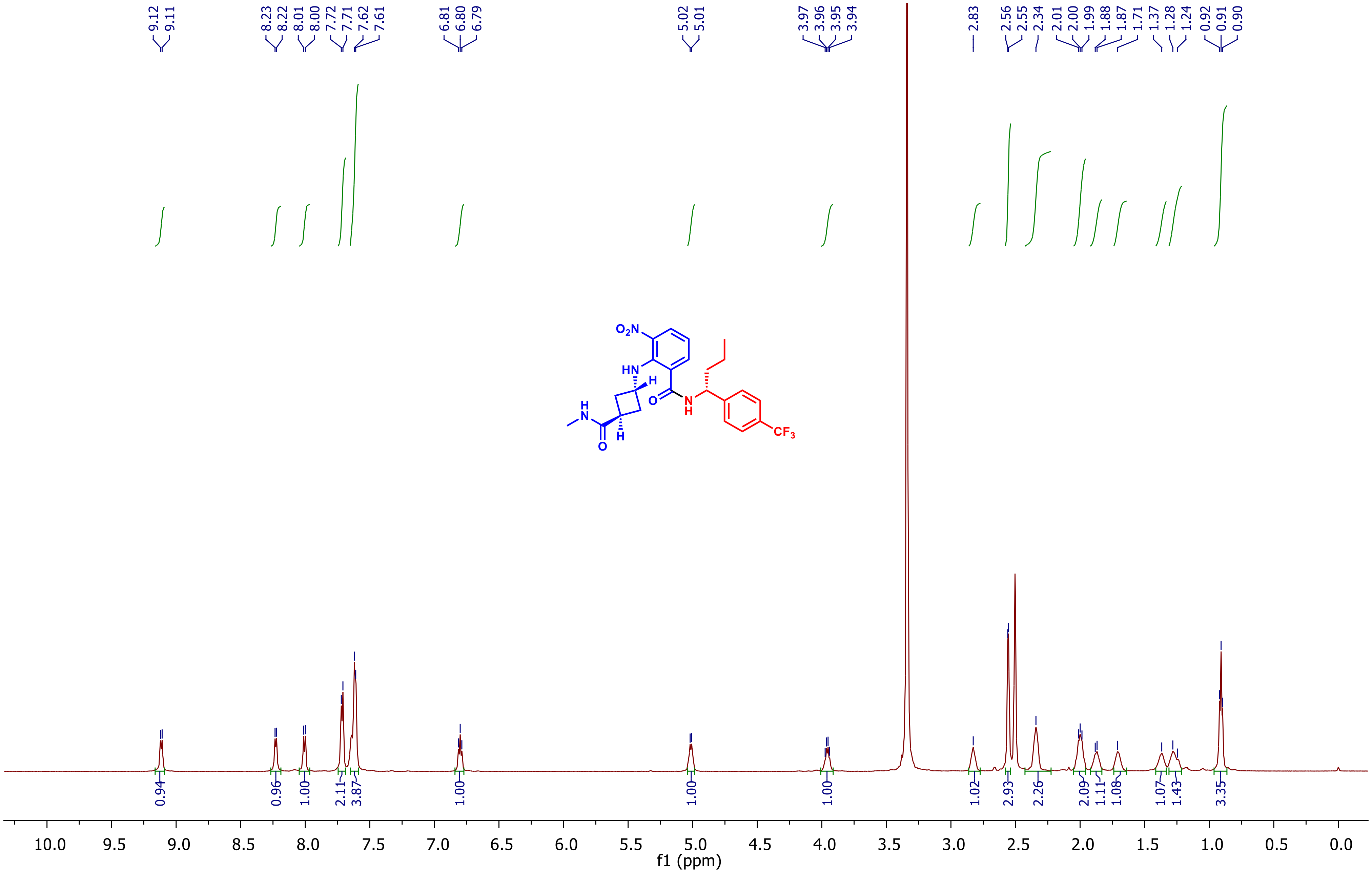
9.12
9.11
8.23
8.22
8.01
8.00
7.72
7.71
7.62
7.61
6.81
6.80
6.79
5.02
5.01
3.97
3.96
3.95
3.94
2.83
2.56
2.55
2.34
2.01
2.00
1.99
1.88
1.87
1.71
1.37
1.28
1.24
0.92
0.91
0.90
O2N
HN
H
H
N
O
H
O
N
H
CF3
0.94
0.96
1.00
2.11
3.87
1.00
1.00
1.00
1.02
2.93
2.26
2.09
1.11
1.08
1.07
1.43
3.35
10.0
9.5
9.0
8.5
8.0
7.5
7.0
6.5
6.0
5.5
5.0
4.5
4.0
3.5
3.0
2.5
2.0
1.5
1.0
0.5
0.0
f1 (ppm)

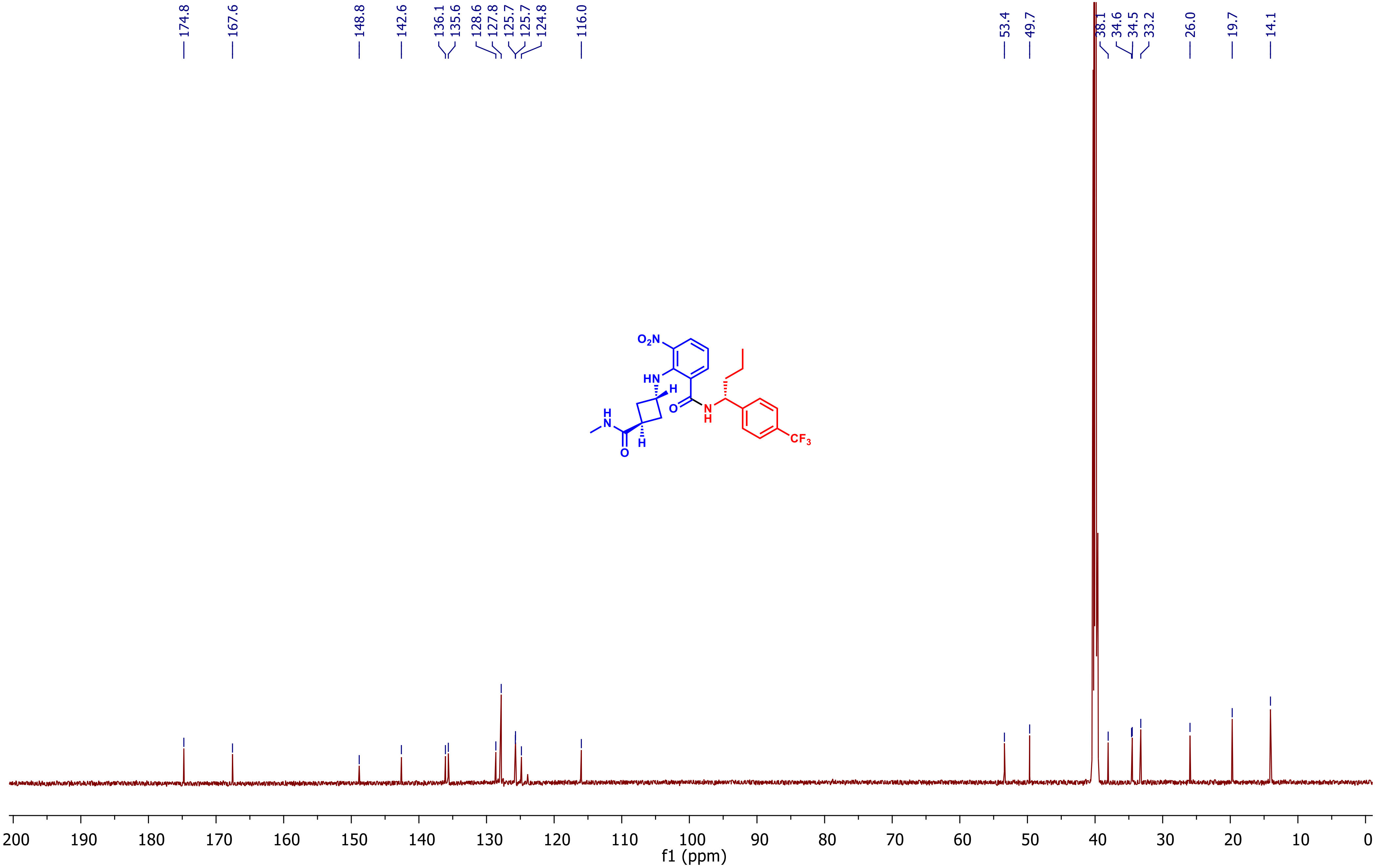
174.8
167.6
148.8
142.6
136.1
135.6
128.6
127.8
125.7
125.7
124.8
116.0
53.4
49.7
38.1
34.6
34.5
33.2
26.0
19.7
14.1
O2N
HN
H
H
N
O
O
N
H
H
CF3
200 190 180 170 160 150 140 130 120 110 100 90 80 70 60 50 40 30 20 10 0
f1 (ppm)

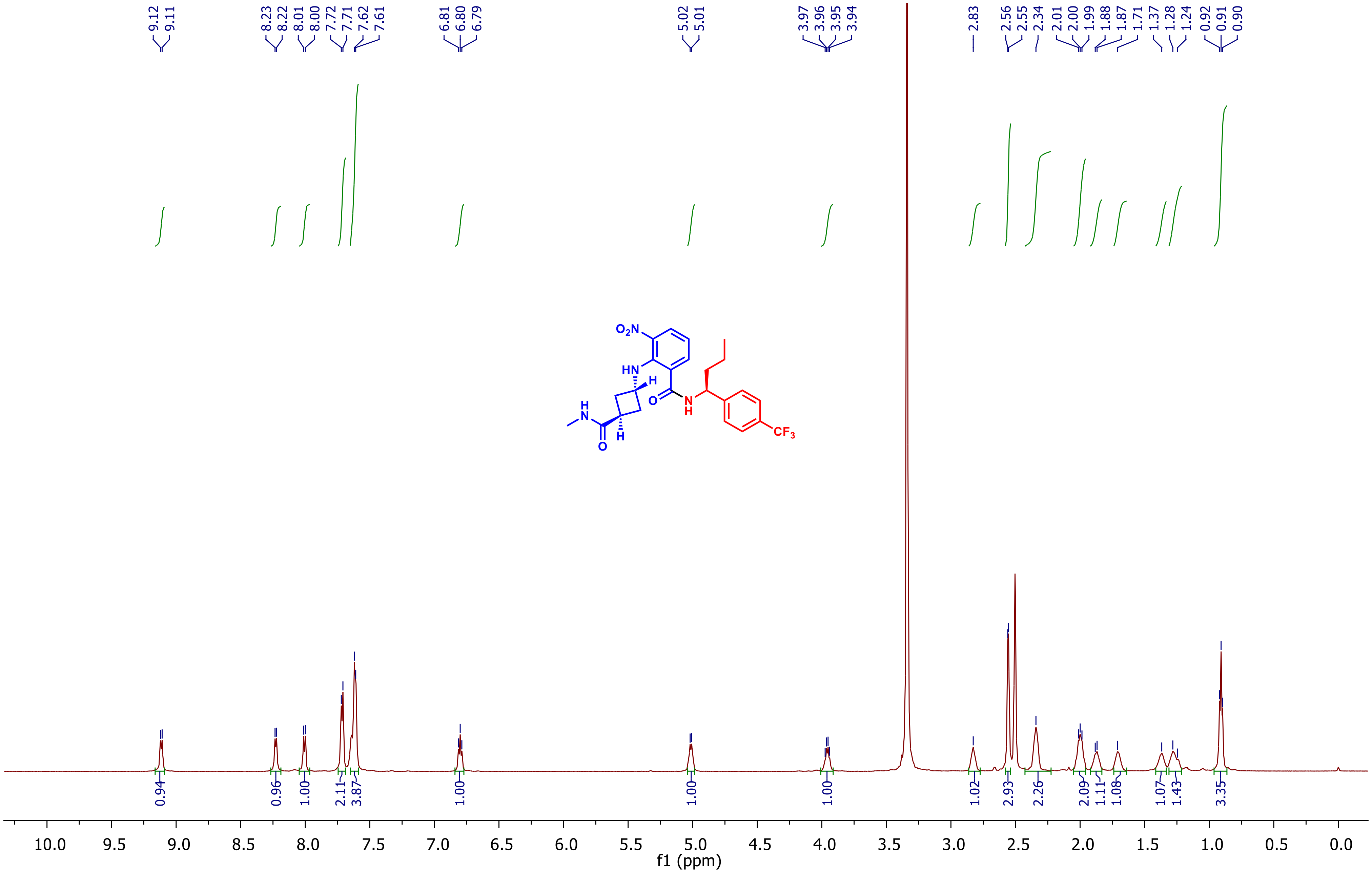

9.12
9.11
8.23
8.22
8.01
8.00
7.72
7.71
7.62
7.61
6.81
6.80
6.79
5.02
5.01
3.97
3.96
3.95
3.94
2.83
2.56
2.55
2.34
2.01
2.00
1.99
1.88
1.87
1.71
1.37
1.28
1.24
0.92
0.91
0.90
O_2N
HN
H
N
H
O
O
CF3
0.94
0.96
1.00
2.11
3.87
1.00
1.00
1.00
1.02
2.93
2.26
2.09
1.11
1.08
1.07
1.43
3.35
f1 (ppm)

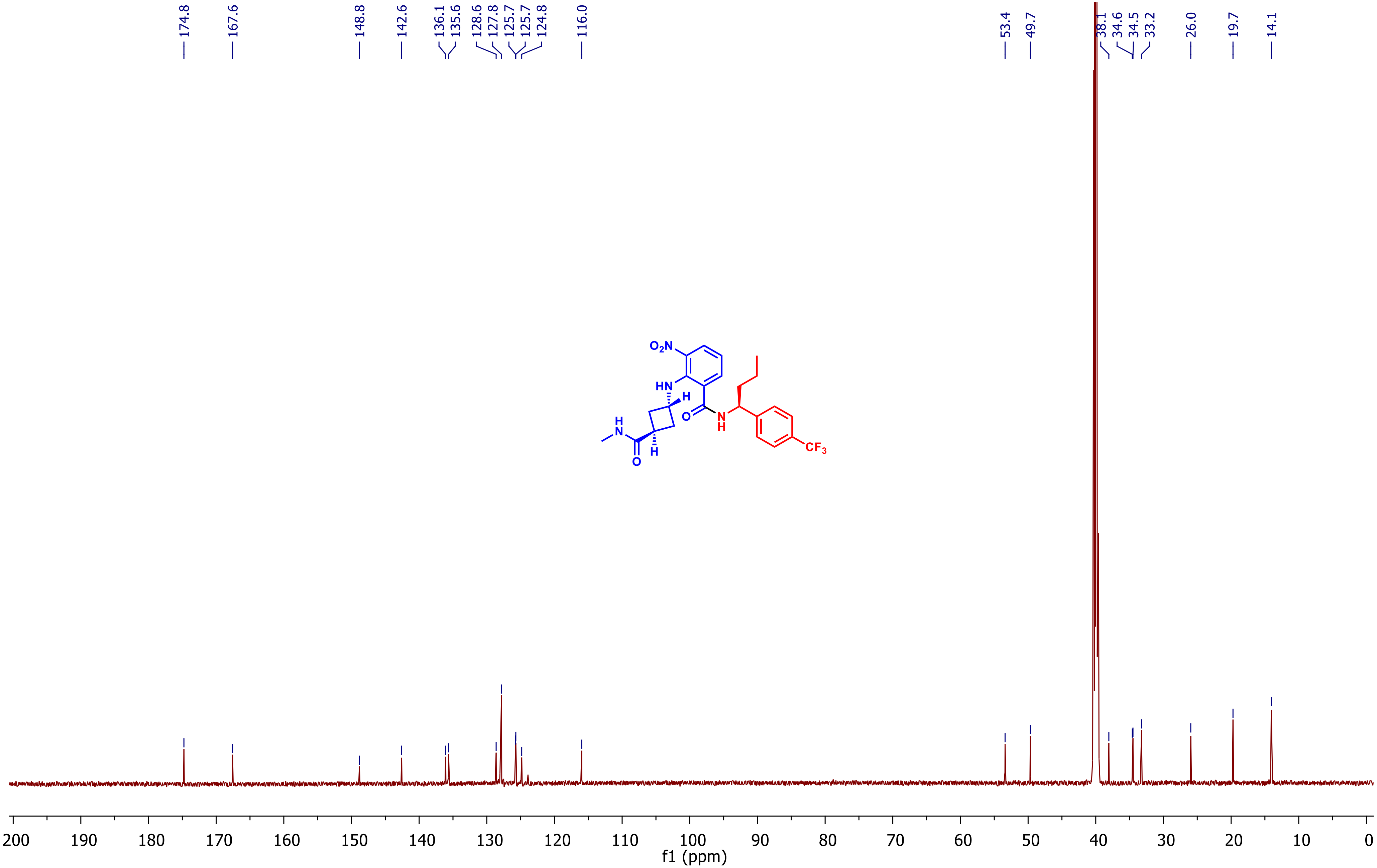
174.8
167.6
148.8
142.6
136.1
135.6
128.6
127.8
125.7
125.7
124.8
116.0
53.4
49.7
38.1
34.6
34.5
33.2
26.0
19.7
14.1
O_2N
HN
H
H
N
H
O
N
H
O
CF_3
200 190 180 170 160 150 140 130 120 110 100 90 80 70 60 50 40 30 20 10 0
f1 (ppm)

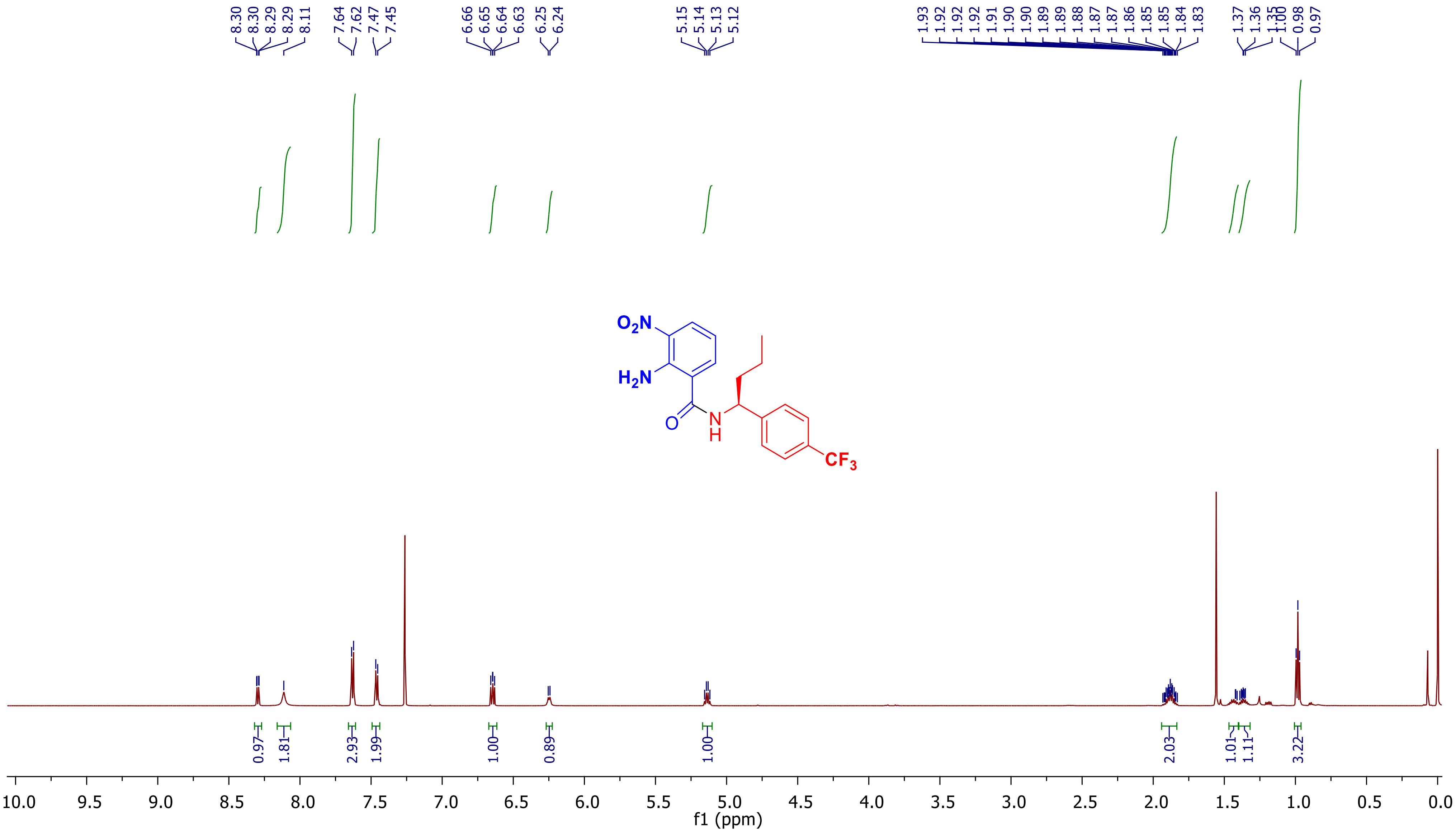
8.30
8.30
8.29
8.11
7.64
7.62
7.47
7.45
6.66
6.65
6.64
6.63
6.25
6.24
5.15
5.14
5.13
5.12
1.93
1.92
1.92
1.92
1.91
1.90
1.90
1.89
1.89
1.88
1.87
1.87
1.86
1.85
1.85
1.84
1.83
1.37
1.36
1.35
1.00
0.98
0.97
O2N
H2N
O
N
H
CF3
0.97
1.81
2.93
1.99
1.00
0.89
1.00
2.03
1.01
1.11
3.22
10.0
9.5
9.0
8.5
8.0
7.5
7.0
6.5
6.0
5.5
5.0
4.5
4.0
3.5
3.0
2.5
2.0
1.5
1.0
0.5
0.0
f1 (ppm)

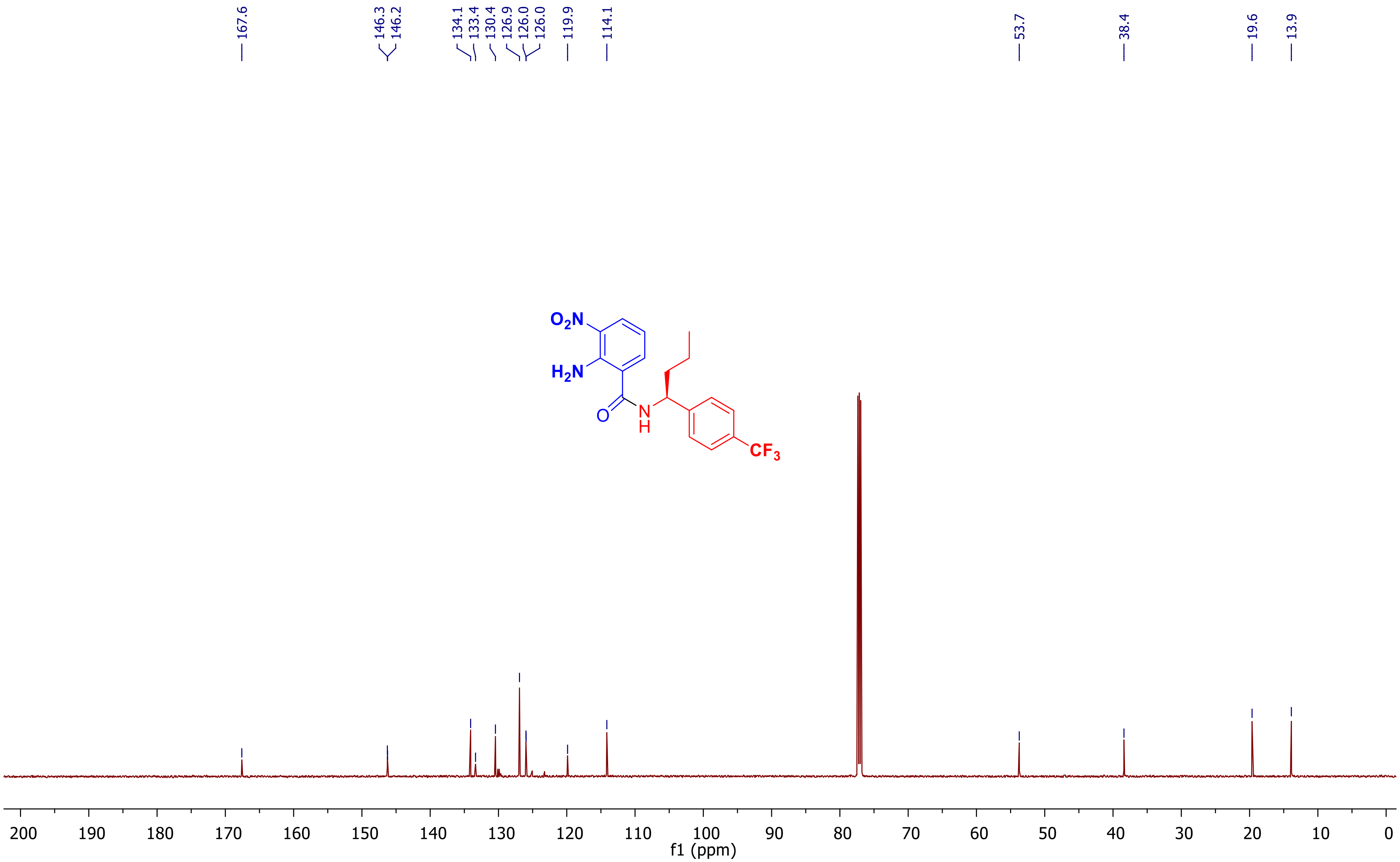
167.6
146.3
146.2
134.1
133.4
130.4
126.9
126.0
126.0
119.9
114.1
53.7
38.4
19.6
13.9
O2N
H2N
O
N
H
CF3
200
190
180
170
160
150
140
130
120
110
100
90
80
70
60
50
40
30
20
10
0
f1 (ppm)

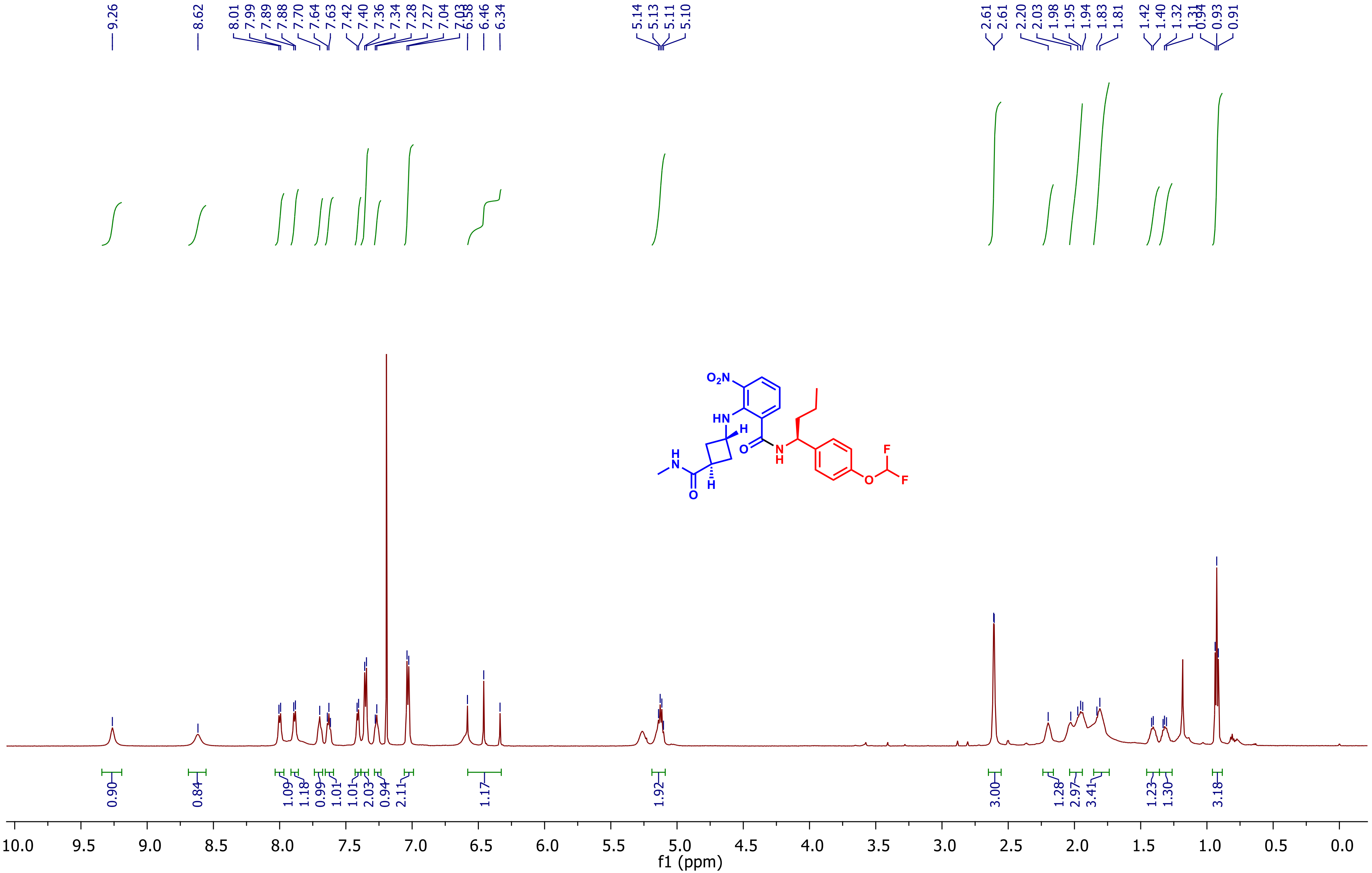
9.26
8.62
8.01
7.99
7.89
7.88
7.70
7.64
7.63
7.42
7.40
7.36
7.34
7.28
7.27
7.04
7.03
6.58
6.46
6.34
5.14
5.13
5.11
5.10
2.61
2.61
2.20
2.03
1.98
1.95
1.94
1.83
1.81
1.42
1.40
1.32
1.31
0.94
0.93
0.91
0.90
0.84
1.09
1.18
0.99
1.01
1.01
2.03
0.94
2.11
1.17
1.92
3.00
1.28
2.97
3.41
1.23
1.30
3.18
O2N
HN
H
H
N
O
O
N
H
H
F
O
F
10.0
9.5
9.0
8.5
8.0
7.5
7.0
6.5
6.0
5.5
5.0
4.5
4.0
3.5
3.0
2.5
2.0
1.5
1.0
0.5
0.0
f1 (ppm)

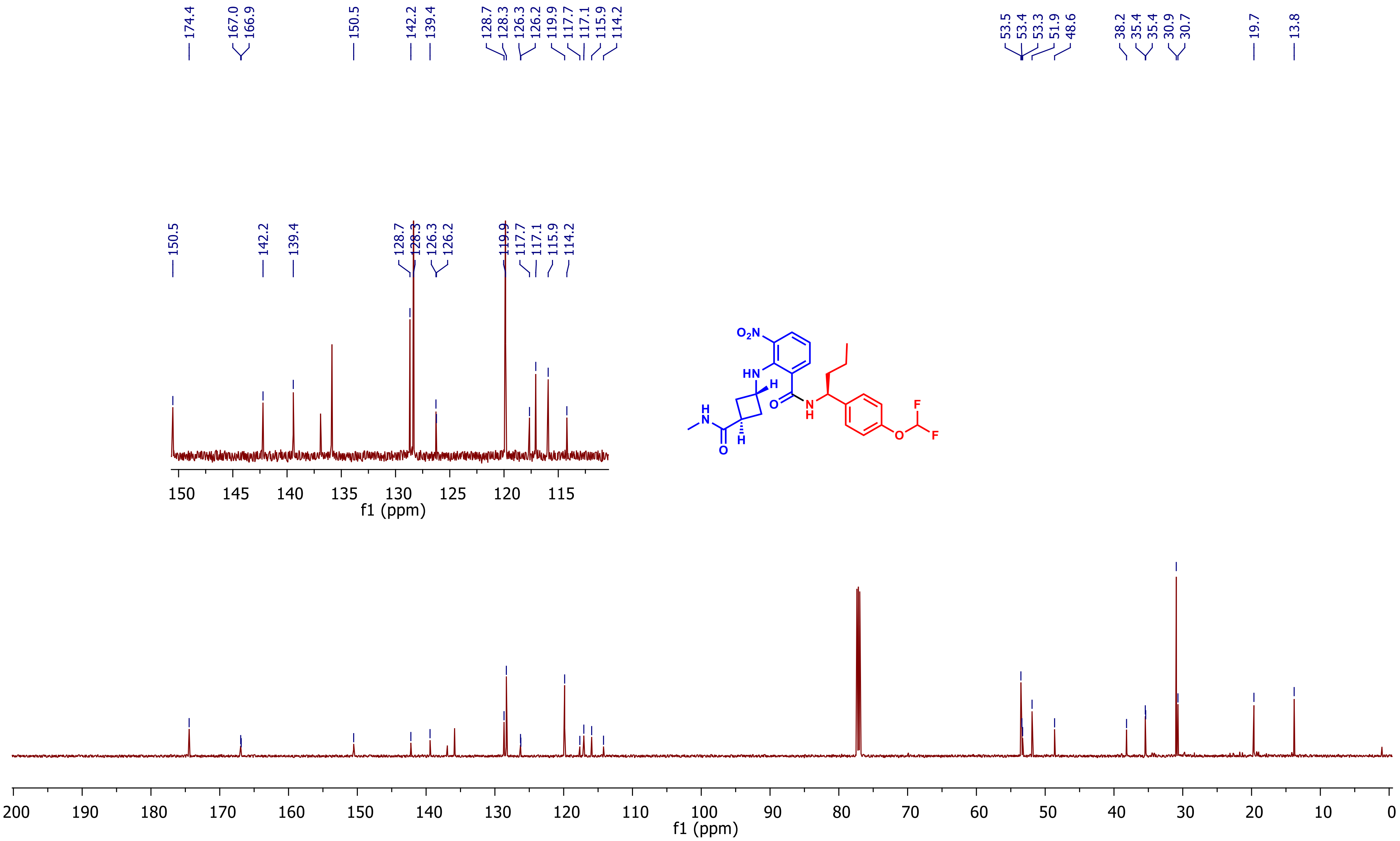
174.4
167.0
166.9
150.5
142.2
139.4
128.7
128.3
126.3
126.2
119.9
117.7
117.1
115.9
114.2
53.5
53.4
53.3
51.9
48.6
38.2
35.4
35.4
30.9
30.7
19.7
13.8
f1 (ppm)
O2N
HN
H
N
O
F
F

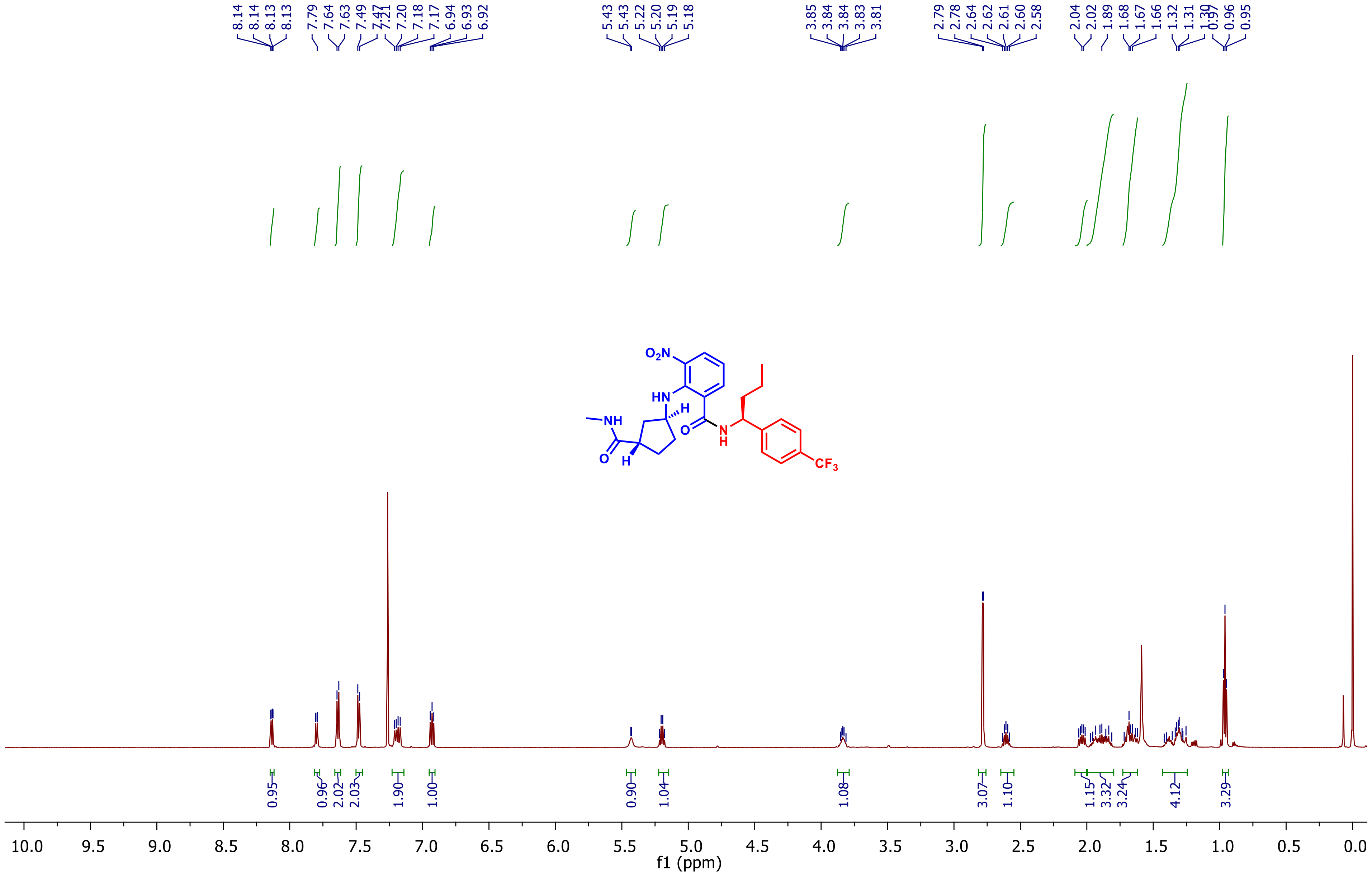
8.14
8.14
8.13
8.13
7.79
7.64
7.63
7.49
7.47
7.21
7.20
7.18
7.17
6.94
6.93
6.92
5.43
5.43
5.22
5.20
5.19
5.18
3.85
3.84
3.84
3.83
3.81
2.79
2.78
2.64
2.62
2.61
2.60
2.58
2.04
2.02
1.89
1.68
1.67
1.66
1.32
1.31
1.30
0.97
0.96
0.95
O2N
HN
H
NH
O
O
N
H
H
CF3
0.95
0.96
2.02
2.03
1.90
1.00
0.90
1.04
1.08
3.07
1.10
1.15
3.32
3.24
4.12
3.29
10.0
9.5
9.0
8.5
8.0
7.5
7.0
6.5
6.0
5.5
5.0
4.5
4.0
3.5
3.0
2.5
2.0
1.5
1.0
0.5
0.0
f1 (ppm)

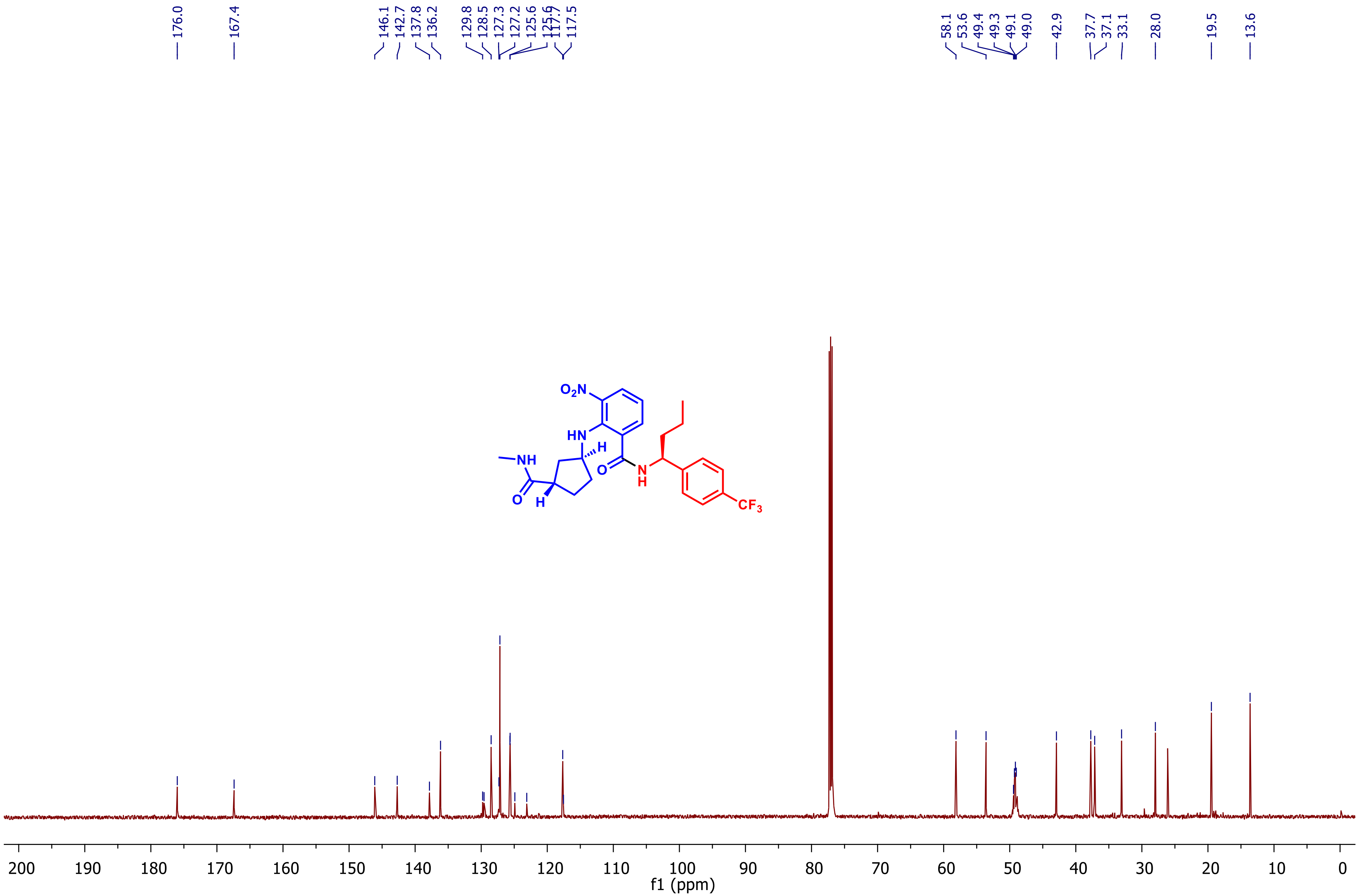
176.0
167.4
146.1
142.7
137.8
136.2
129.8
128.5
127.3
127.2
125.6
117.7
117.5
58.1
53.6
49.4
49.3
49.1
49.0
42.9
37.7
37.1
33.1
28.0
19.5
13.6
O_2N
HN
H
NH
O
H
O
N
H
CF_3
200
190
180
170
160
150
140
130
120
110
100
90
80
70
60
50
40
30
20
10
0
f1 (ppm)

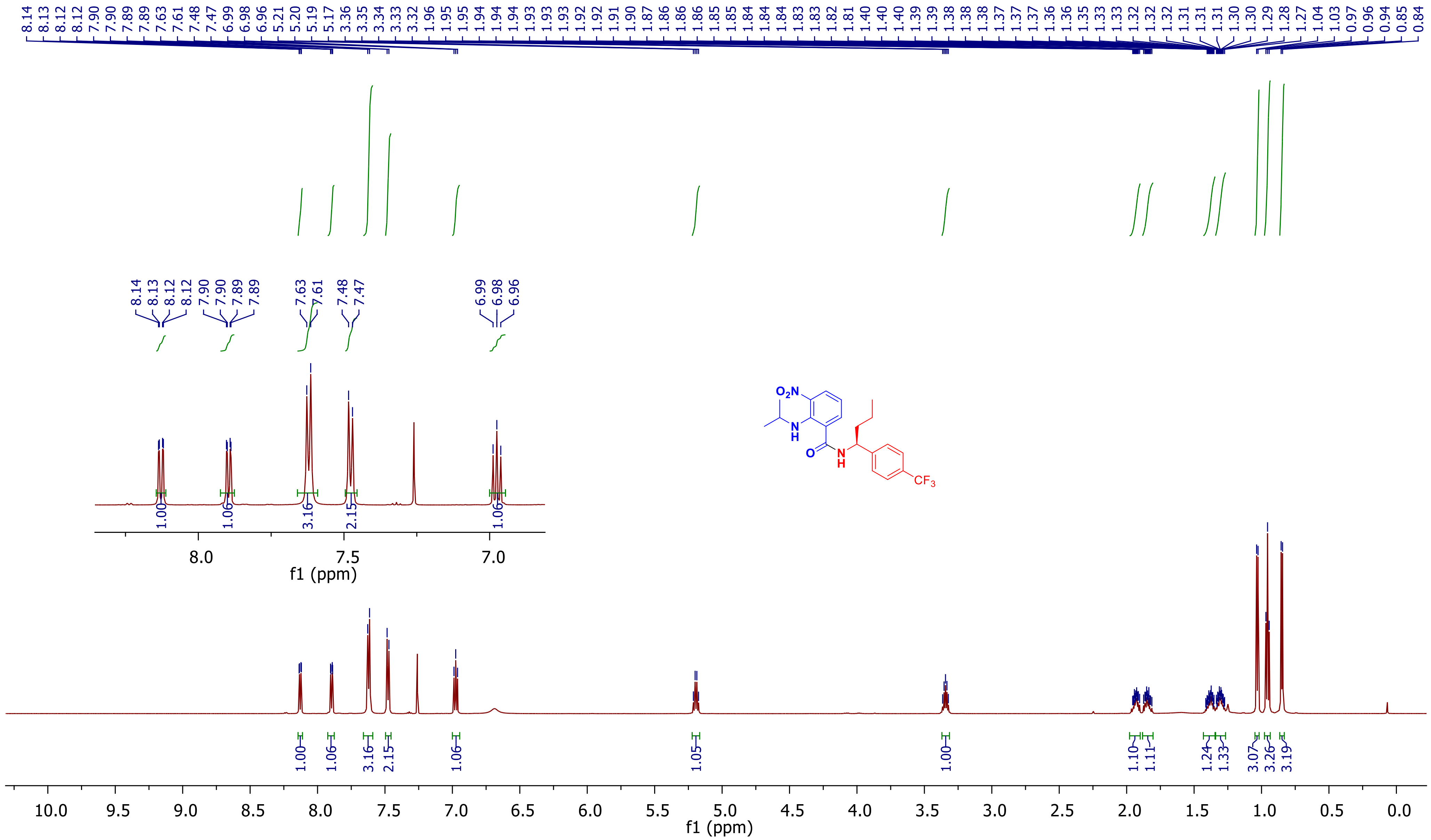

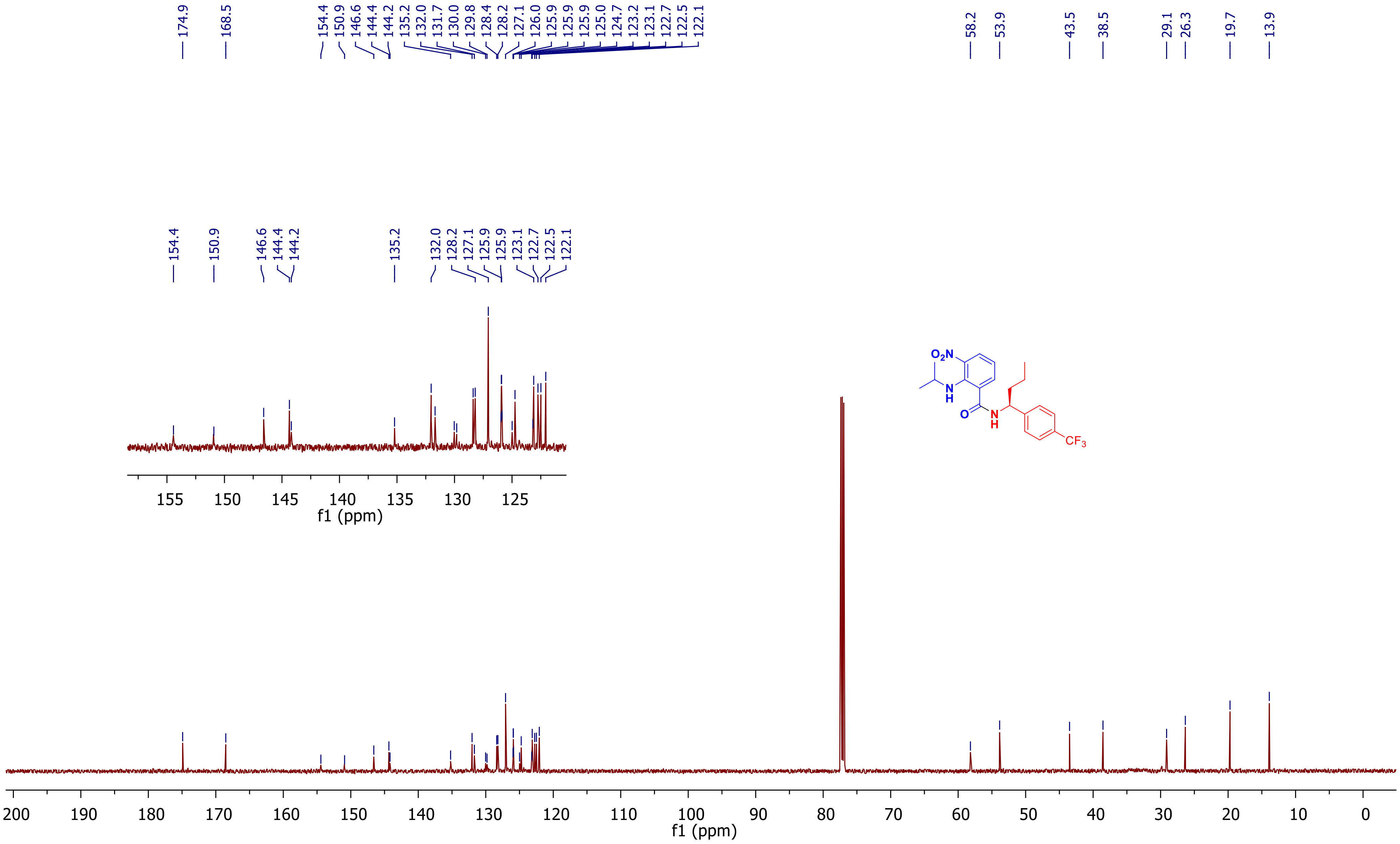
174.9
168.5
154.4
150.9
146.6
144.4
144.2
135.2
132.0
131.7
130.0
129.8
128.4
128.2
127.1
126.0
125.9
125.9
125.9
125.0
124.7
123.2
123.1
122.7
122.5
122.1
58.2
53.9
43.5
38.5
29.1
26.3
19.7
13.9
154.4
150.9
146.6
144.4
144.2
135.2
132.0
128.2
127.1
125.9
125.9
123.1
122.7
122.5
122.1
155
150
145
140
135
130
125
f1 (ppm)
O2N
N
H
O
N
H
CF3
200
190
180
170
160
150
140
130
120
110
100
90
80
70
60
50
40
30
20
10
0
f1 (ppm)

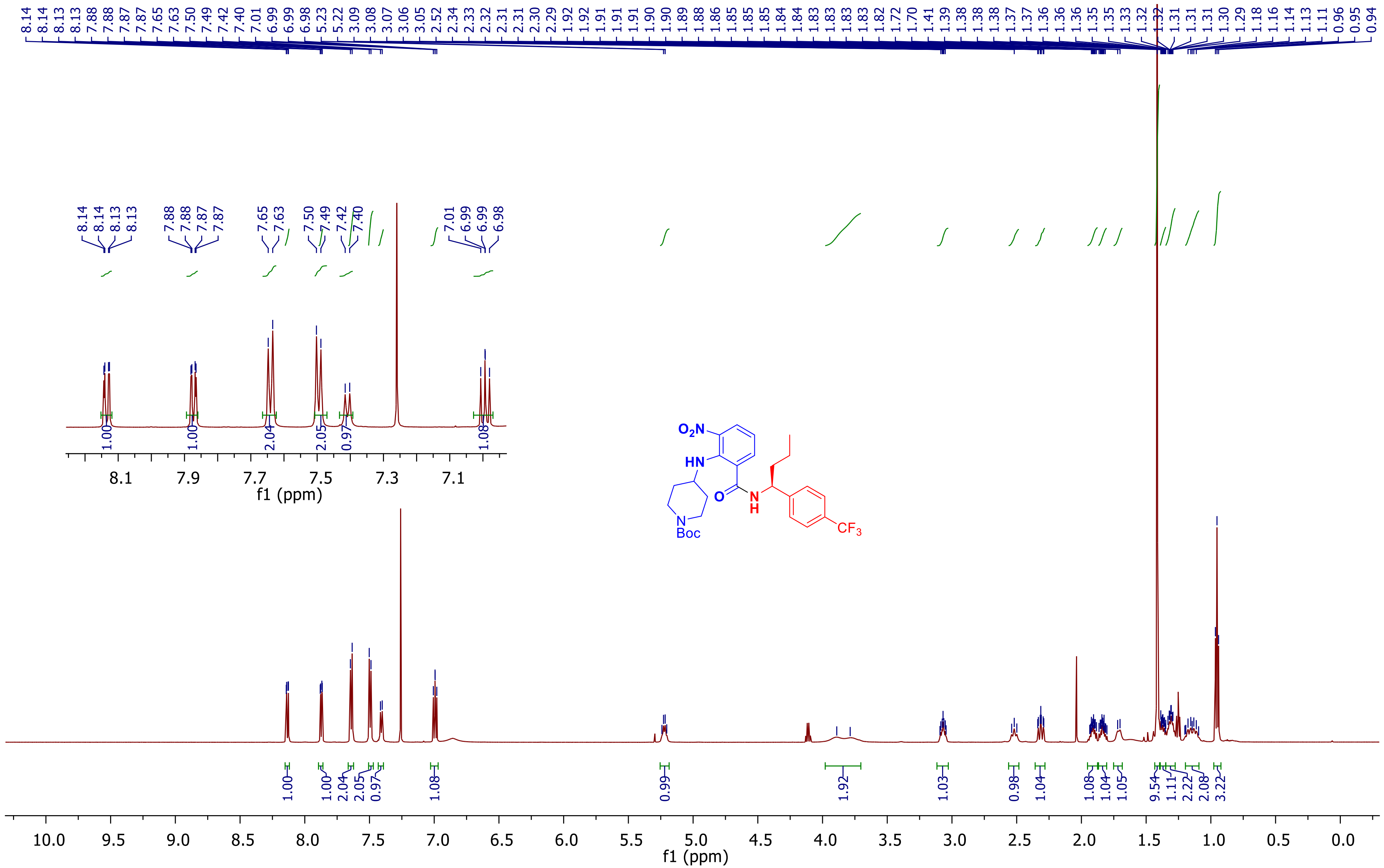
8.14 8.14 8.13 8.13 7.88 7.88 7.87 7.87 7.65 7.63 7.50 7.49 7.42 7.40 7.01 6.99 6.98 5.23 5.22 3.09 3.08 3.07 3.06 3.05 2.52 2.34 2.33 2.32 2.31 2.31 2.30 2.29 1.92 1.92 1.91 1.91 1.91 1.90 1.90 1.89 1.88 1.86 1.85 1.85 1.85 1.84 1.84 1.83 1.83 1.83 1.82 1.72 1.70 1.41 1.39 1.38 1.38 1.38 1.37 1.37 1.36 1.36 1.36 1.35 1.35 1.33 1.32 1.32 1.31 1.31 1.31 1.30 1.29 1.18 1.16 1.14 1.13 1.11 0.96 0.95 0.94
8.14 8.14 8.13 8.13 7.88 7.88 7.87 7.87 7.65 7.63 7.50 7.49 7.42 7.40 7.01 6.99 6.99 6.98
1.00 1.00 2.04 2.05 0.97 1.08
8.1 7.9 7.7 7.5 7.3 7.1
f1 (ppm)
O2N
HN
N
Boc
O
N
H
CF3
1.00 1.00 2.04 2.05 0.97 1.08 0.99 1.92 1.03 0.98 1.04 1.08 1.04 1.05 9.54 1.11 2.22 2.08 3.22
10.0 9.5 9.0 8.5 8.0 7.5 7.0 6.5 6.0 5.5 5.0 4.5 4.0 3.5 3.0 2.5 2.0 1.5 1.0 0.5 0.0
f1 (ppm)

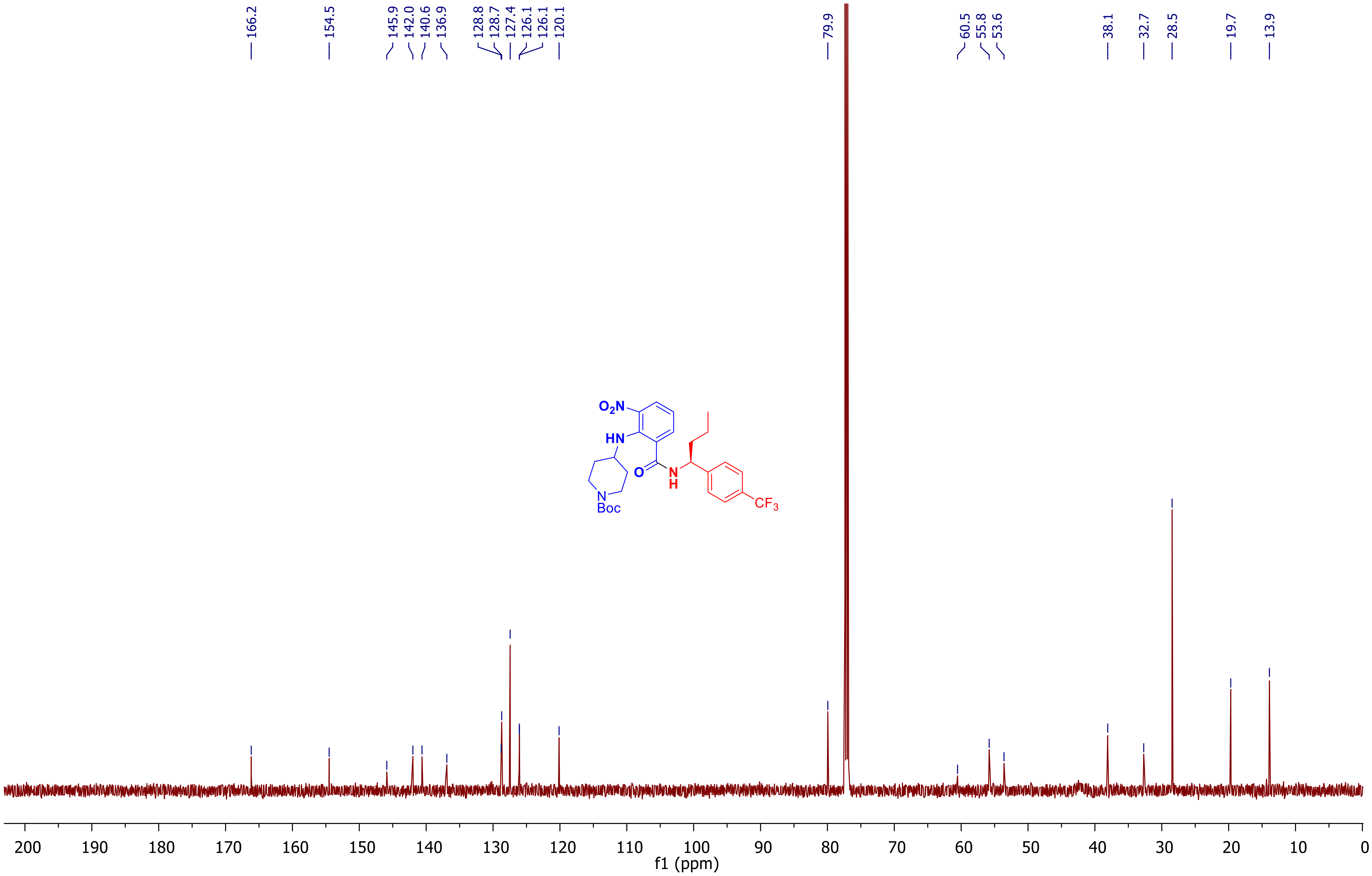
166.2
154.5
145.9
142.0
140.6
136.9
128.8
128.7
127.4
126.1
126.1
120.1
79.9
60.5
55.8
53.6
38.1
32.7
28.5
19.7
13.9
O2N
HN
O
N
H
N
Boc
CF3
200 190 180 170 160 150 140 130 120 110 100 90 80 70 60 50 40 30 20 10 0
f1 (ppm)

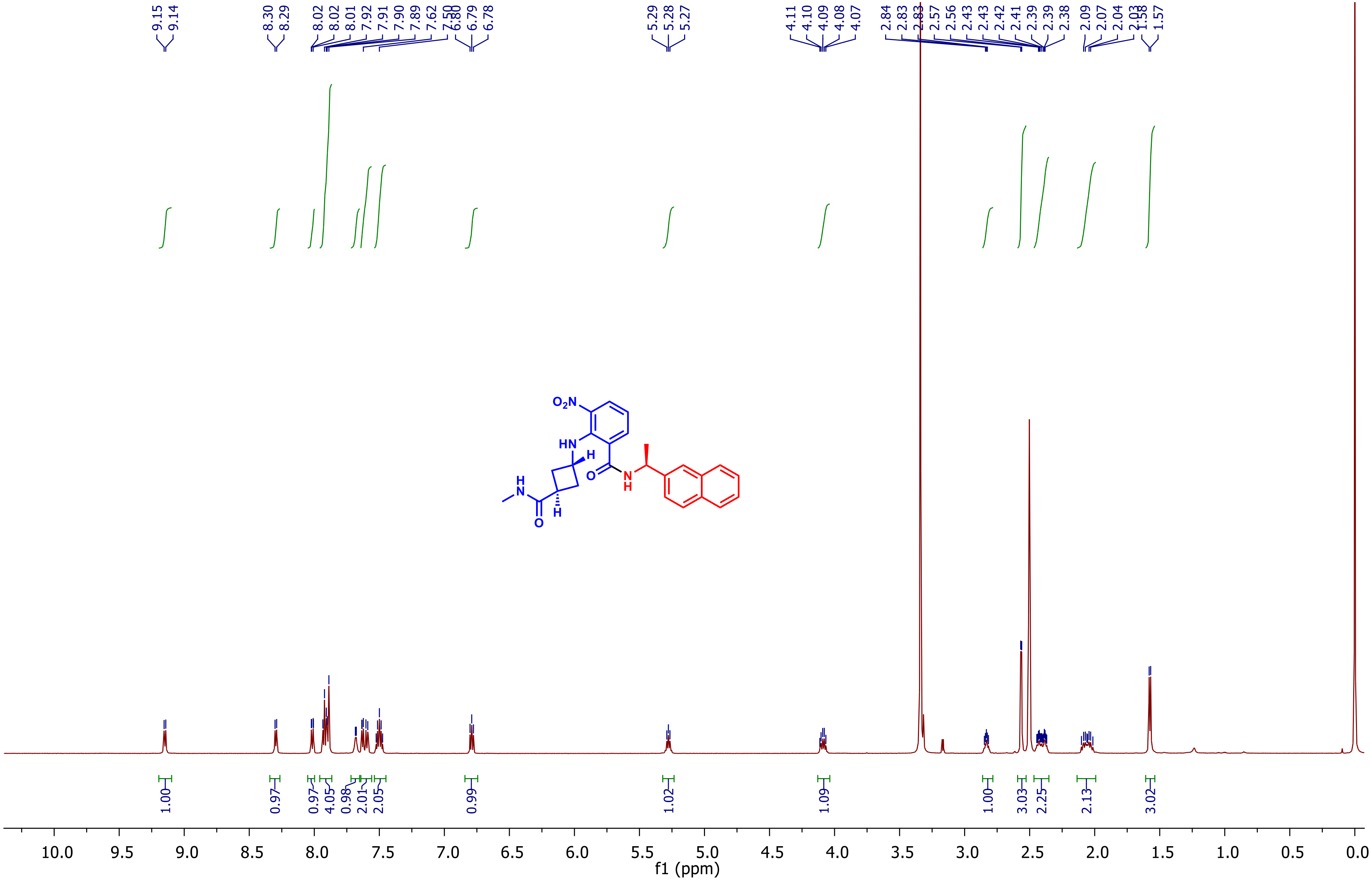
9.15
9.14
8.30
8.29
8.02
8.02
8.01
7.92
7.91
7.90
7.89
7.62
7.50
6.80
6.79
6.78
5.29
5.28
5.27
4.11
4.10
4.09
4.08
4.07
2.84
2.83
2.83
2.57
2.56
2.43
2.43
2.42
2.41
2.39
2.39
2.38
2.09
2.07
2.04
2.03
1.58
1.57
O2N
HN
H
H
N
O
O
N
H
H
1.00
0.97
0.97
4.05
0.98
2.01
2.05
0.99
1.02
1.09
1.00
3.03
2.25
2.13
3.02
10.0 9.5 9.0 8.5 8.0 7.5 7.0 6.5 6.0 5.5 5.0 4.5 4.0 3.5 3.0 2.5 2.0 1.5 1.0 0.5 0.0
f1 (ppm)

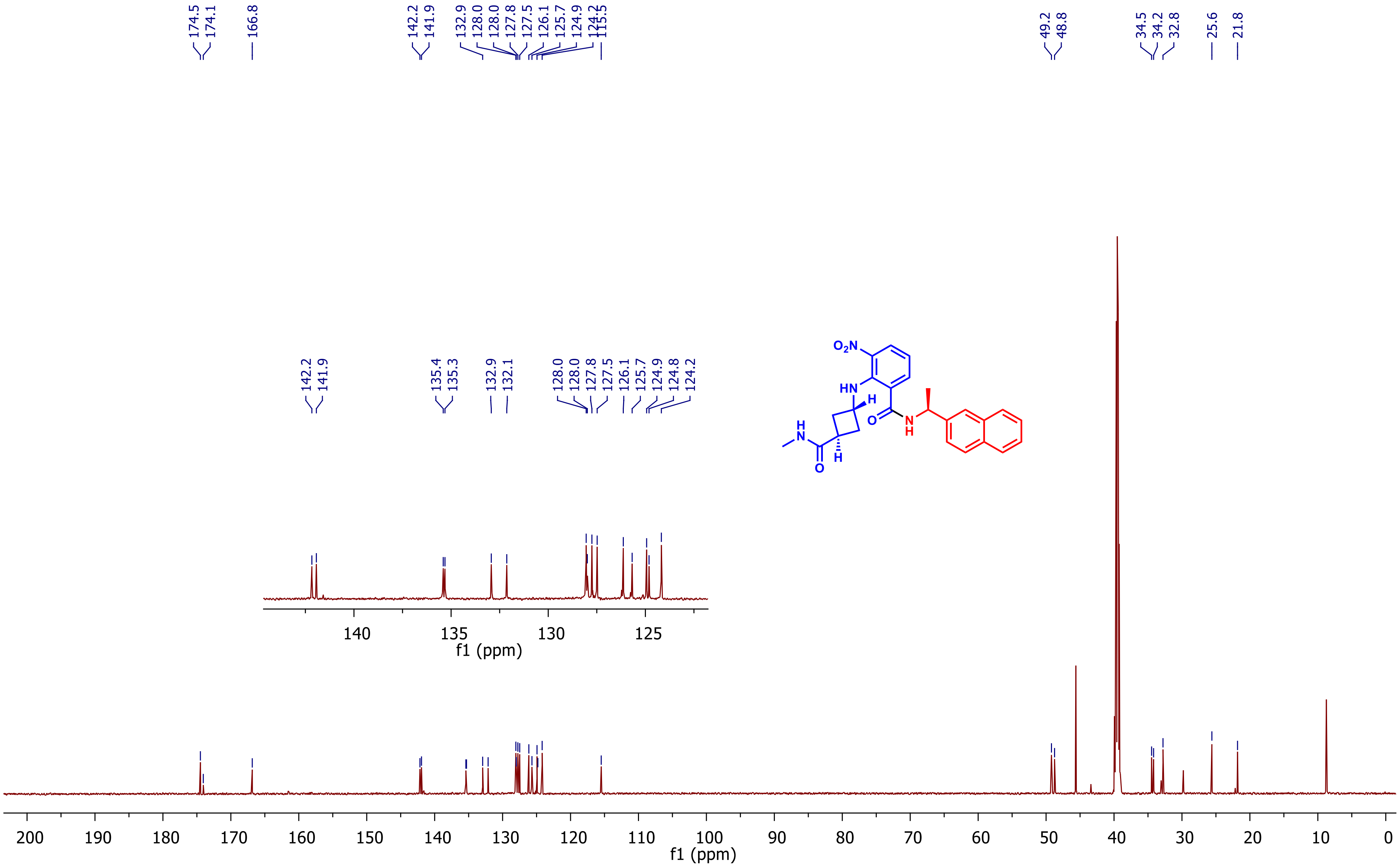
174.5
174.1
166.8
142.2
141.9
132.9
128.0
128.0
127.8
127.5
126.1
125.7
124.9
124.2
115.5
49.2
48.8
34.5
34.2
32.8
25.6
21.8
142.2
141.9
135.4
135.3
132.9
132.1
128.0
128.0
127.8
127.5
126.1
125.7
124.9
124.8
124.2
f1 (ppm)
f1 (ppm)

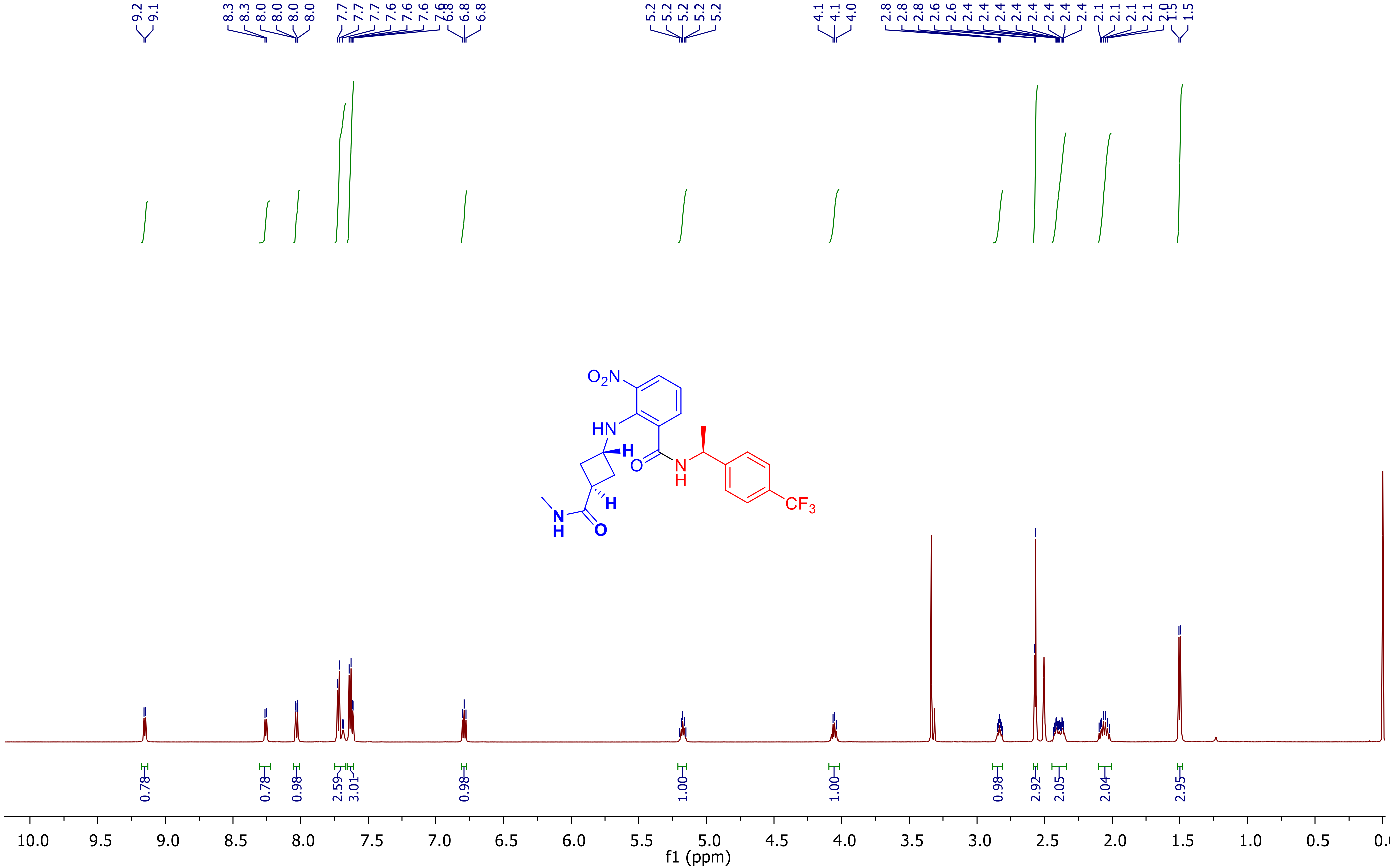
9.2 9.1
8.3 8.3 8.0 8.0 8.0 8.0
7.7 7.7 7.7 7.6 7.6 7.6 7.6 6.8 6.8 6.8
5.2 5.2 5.2 5.2 5.2
4.1 4.1 4.0
2.8 2.8 2.8 2.6 2.6 2.4 2.4 2.4 2.4 2.4 2.4 2.4 2.4
2.1 2.1 2.1 2.1 2.0 1.5 1.5
O2N
HN
H
O
N
H
H
N
H
O
CF3
0.78 0.78 0.98 2.59 3.01 0.98 1.00 1.00 0.98 2.92 2.05 2.04 2.95
10.0 9.5 9.0 8.5 8.0 7.5 7.0 6.5 6.0 5.5 5.0 4.5 4.0 3.5 3.0 2.5 2.0 1.5 1.0 0.5 0.0
f1 (ppm)

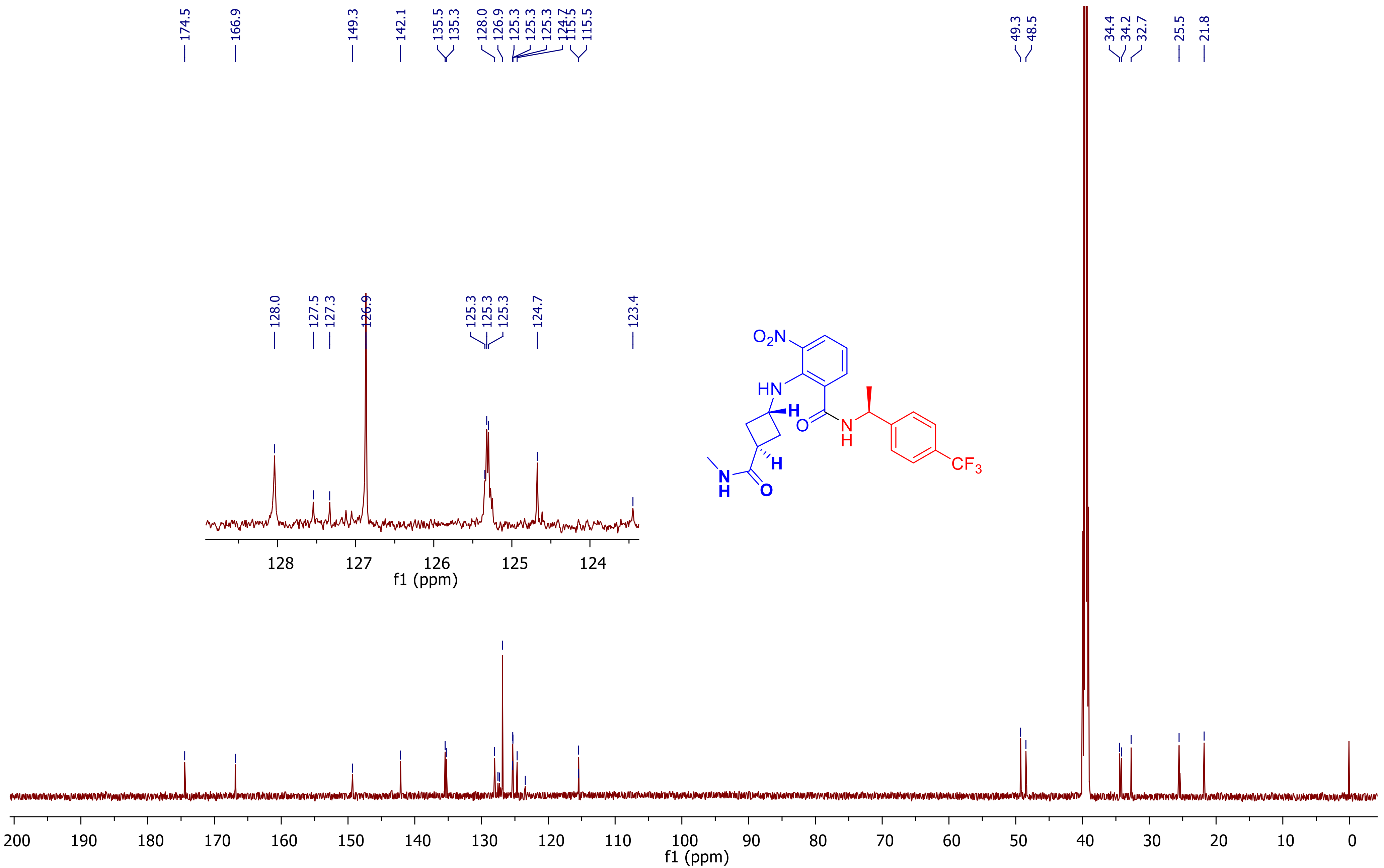
174.5
166.9
149.3
142.1
135.5
135.3
128.0
126.9
125.3
125.3
125.3
124.7
115.5
115.5
49.3
48.5
34.4
34.2
32.7
25.5
21.8
128.0
127.5
127.3
126.9
125.3
125.3
125.3
124.7
123.4
f1 (ppm)
O_2N
HN
H
O
N
H
H
N
H
O
CF_3
f1 (ppm)

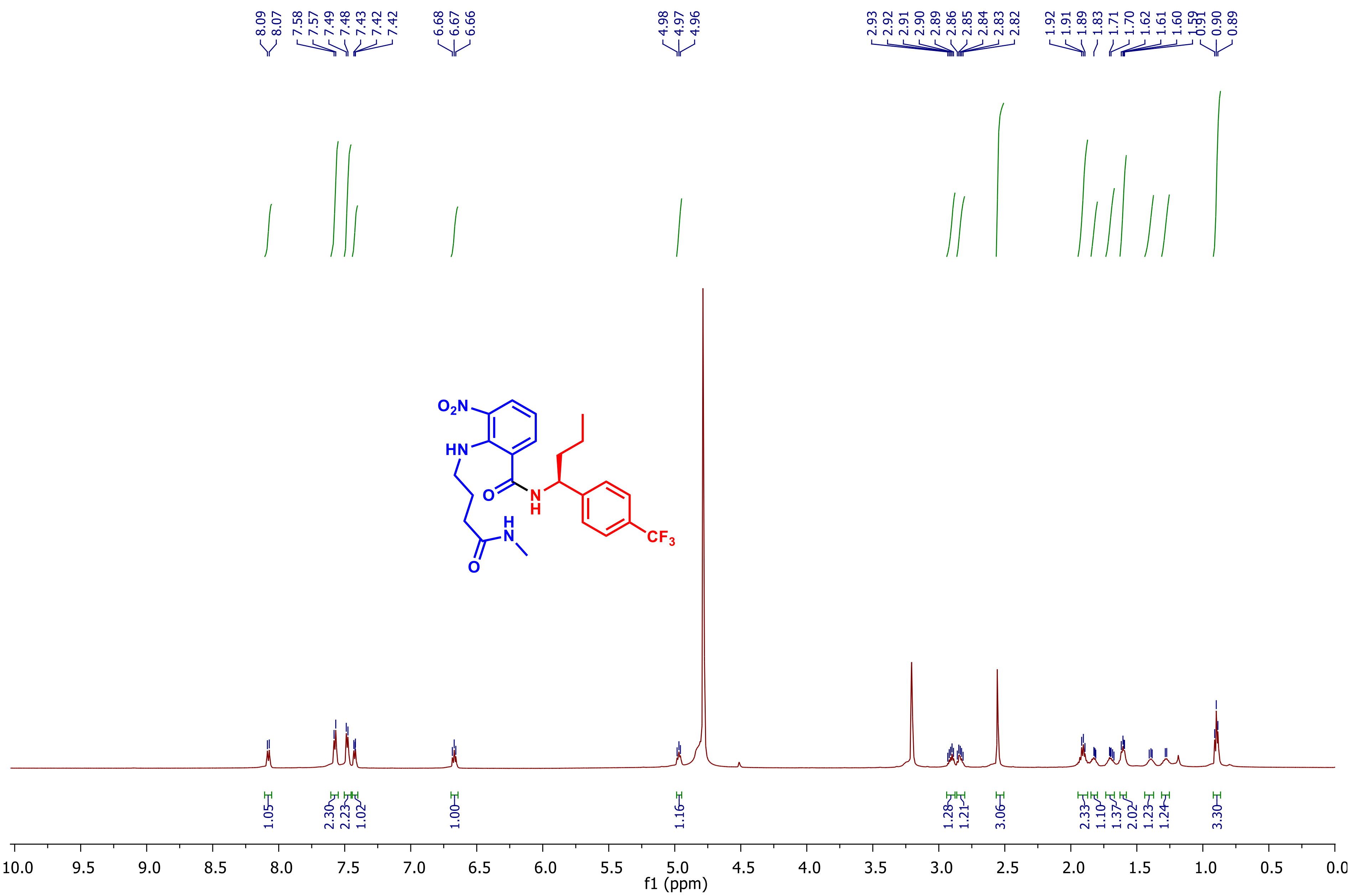

8.09
8.07
7.58
7.57
7.49
7.48
7.43
7.42
7.42
6.68
6.67
6.66
4.98
4.97
4.96
2.93
2.92
2.91
2.90
2.89
2.86
2.85
2.84
2.83
2.82
1.92
1.91
1.89
1.83
1.71
1.70
1.62
1.61
1.60
1.59
0.91
0.90
0.89
O2N
HN
O
N
H
O
H
N
CF3
1.05
2.30
2.23
1.02
1.00
1.16
1.28
1.21
3.06
2.33
1.10
1.37
2.02
1.23
1.24
3.30
10.0
9.5
9.0
8.5
8.0
7.5
7.0
6.5
6.0
5.5
5.0
4.5
4.0
3.5
3.0
2.5
2.0
1.5
1.0
0.5
0.0
f1 (ppm)

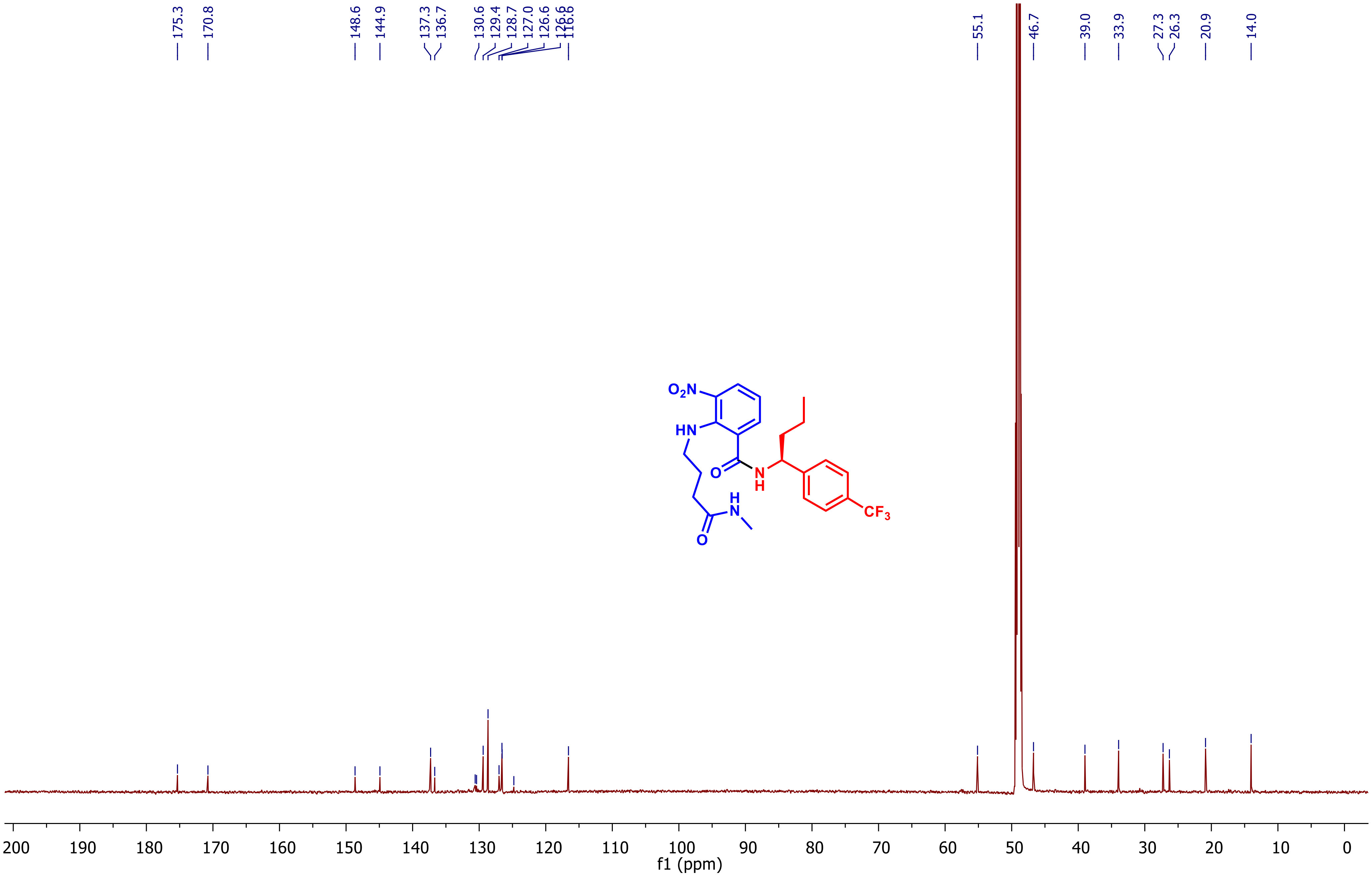
175.3
170.8
148.6
144.9
137.3
136.7
130.6
129.4
128.7
127.0
126.6
126.6
116.6
55.1
46.7
39.0
33.9
27.3
26.3
20.9
14.0
O_2N
HN
O
N
H
H
N
O
CF_3
200
190
180
170
160
150
140
130
120
110
100
90
80
70
60
50
40
30
20
10
0
f1 (ppm)

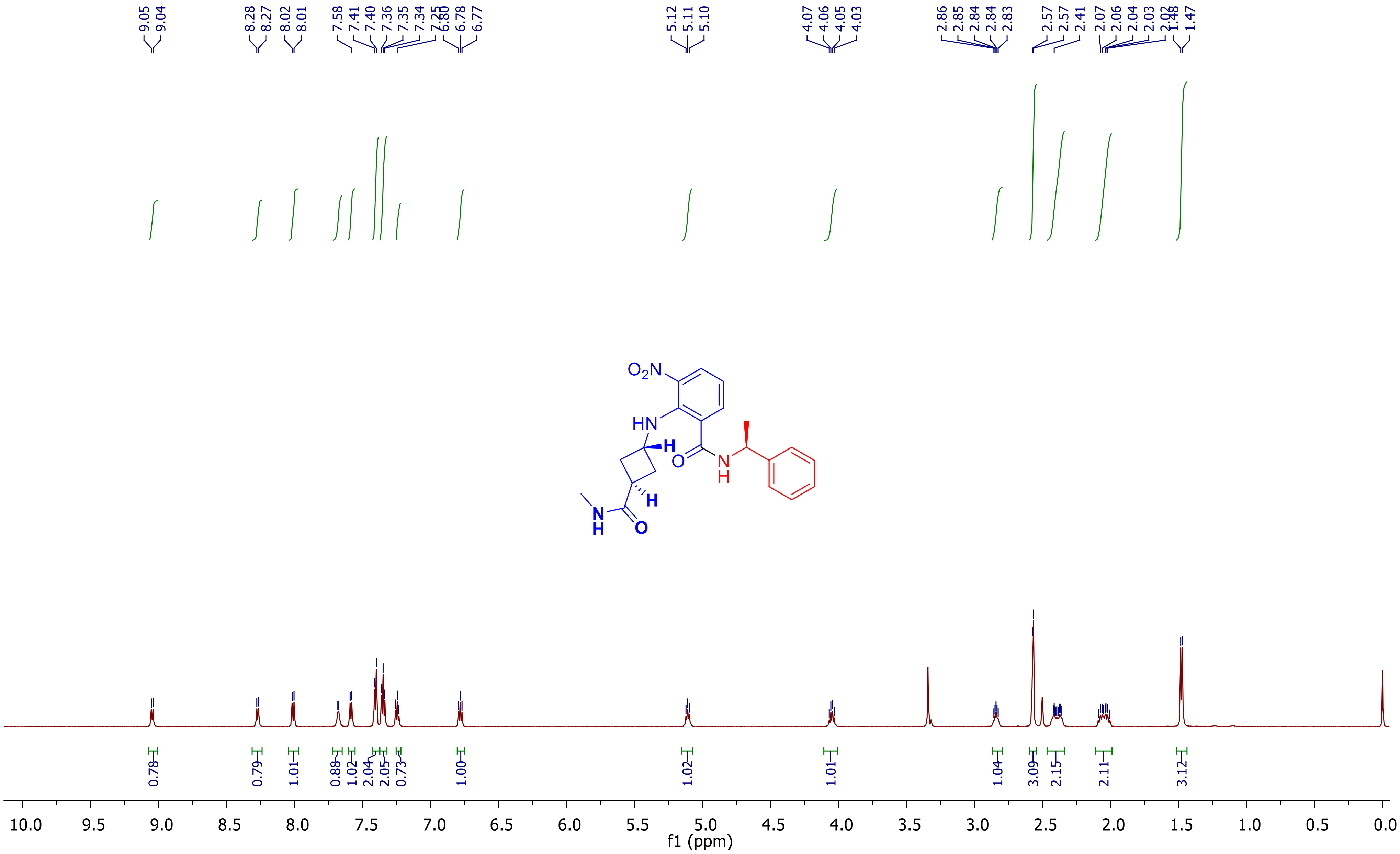
9.05
9.04
8.28
8.27
8.02
8.01
7.58
7.41
7.40
7.36
7.35
7.34
7.25
6.80
6.78
6.77
5.12
5.11
5.10
4.07
4.06
4.05
4.03
2.86
2.85
2.84
2.84
2.83
2.57
2.57
2.41
2.07
2.06
2.04
2.03
2.02
1.48
1.47
O_2N
HN
H
H
O
N
H
N
H
O
0.78
0.79
1.01
0.88
1.02
2.04
2.05
0.73
1.00
1.02
1.01
1.04
3.09
2.15
2.11
3.12
10.0
9.5
9.0
8.5
8.0
7.5
7.0
6.5
6.0
5.5
5.0
4.5
4.0
3.5
3.0
2.5
2.0
1.5
1.0
0.5
0.0
f1 (ppm)

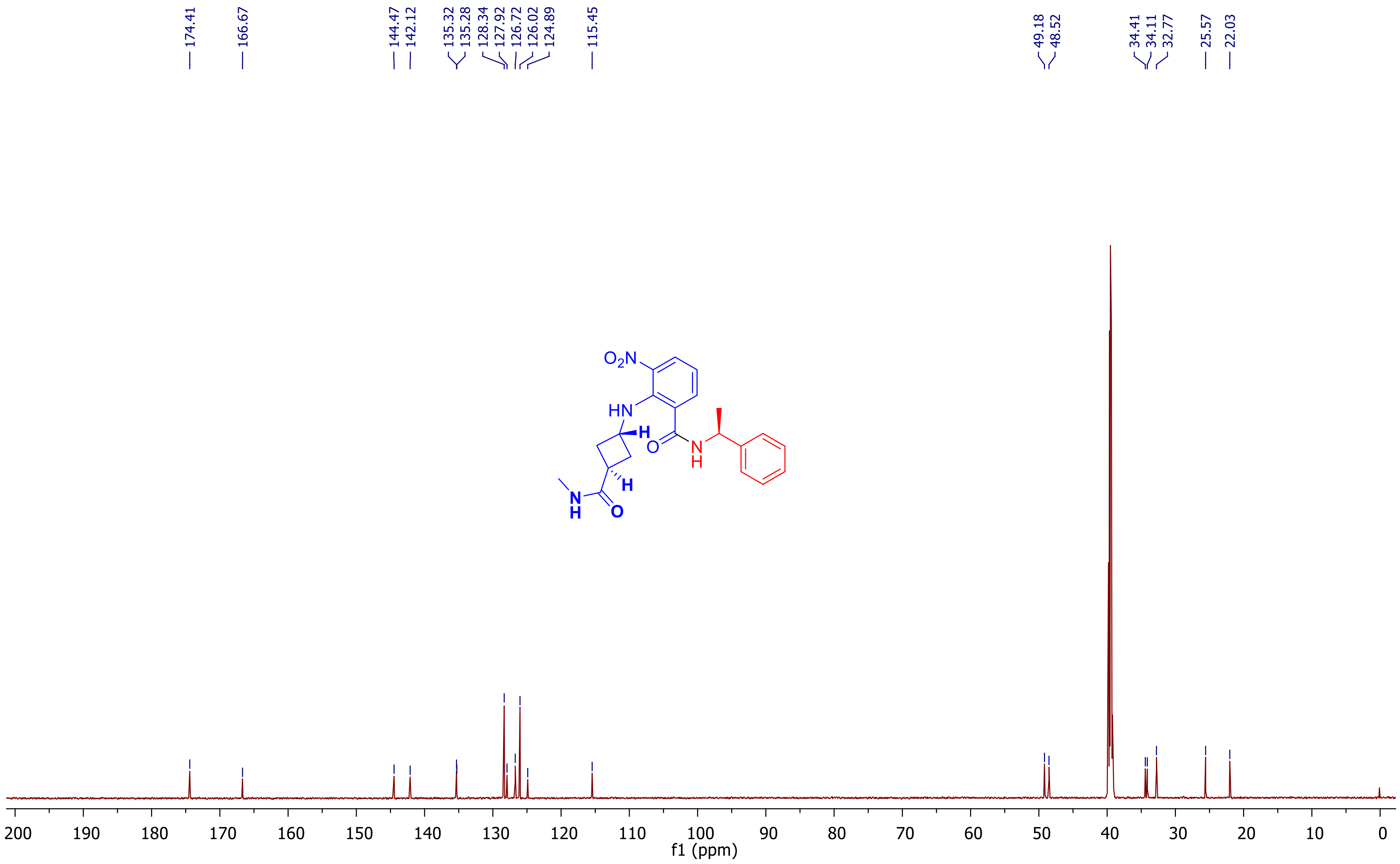
174.41
166.67
144.47
142.12
135.32
135.28
128.34
127.92
126.72
126.02
124.89
115.45
49.18
48.52
34.41
34.11
32.77
25.57
22.03
O2N
HN
H
O
N
H
H
N
H
O
200 190 180 170 160 150 140 130 120 110 100 90 80 70 60 50 40 30 20 10 0
f1 (ppm)

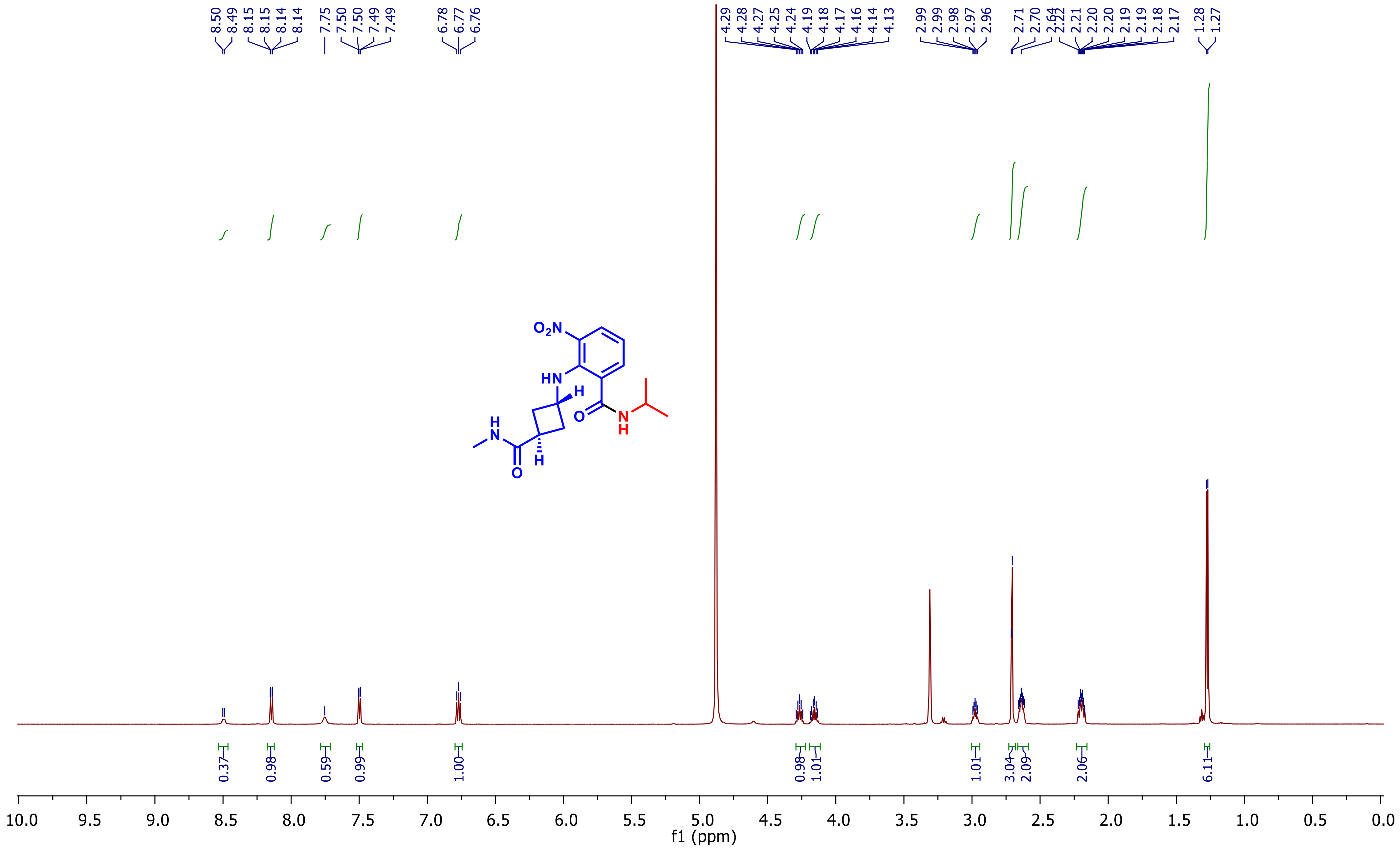
8.50
8.49
8.15
8.15
8.14
8.14
7.75
7.50
7.50
7.49
7.49
6.78
6.77
6.76
4.29
4.28
4.27
4.25
4.24
4.19
4.18
4.17
4.16
4.14
4.13
2.99
2.99
2.98
2.97
2.96
2.71
2.70
2.64
2.22
2.21
2.20
2.20
2.19
2.19
2.18
2.17
1.28
1.27
O2N
HN
H
H
N
H
O
O
N
H
0.37
0.98
0.59
0.99
1.00
0.98
1.01
1.01
3.04
2.09
2.06
6.11
10.0
9.5
9.0
8.5
8.0
7.5
7.0
6.5
6.0
5.5
5.0
4.5
4.0
3.5
3.0
2.5
2.0
1.5
1.0
0.5
0.0
f1 (ppm)

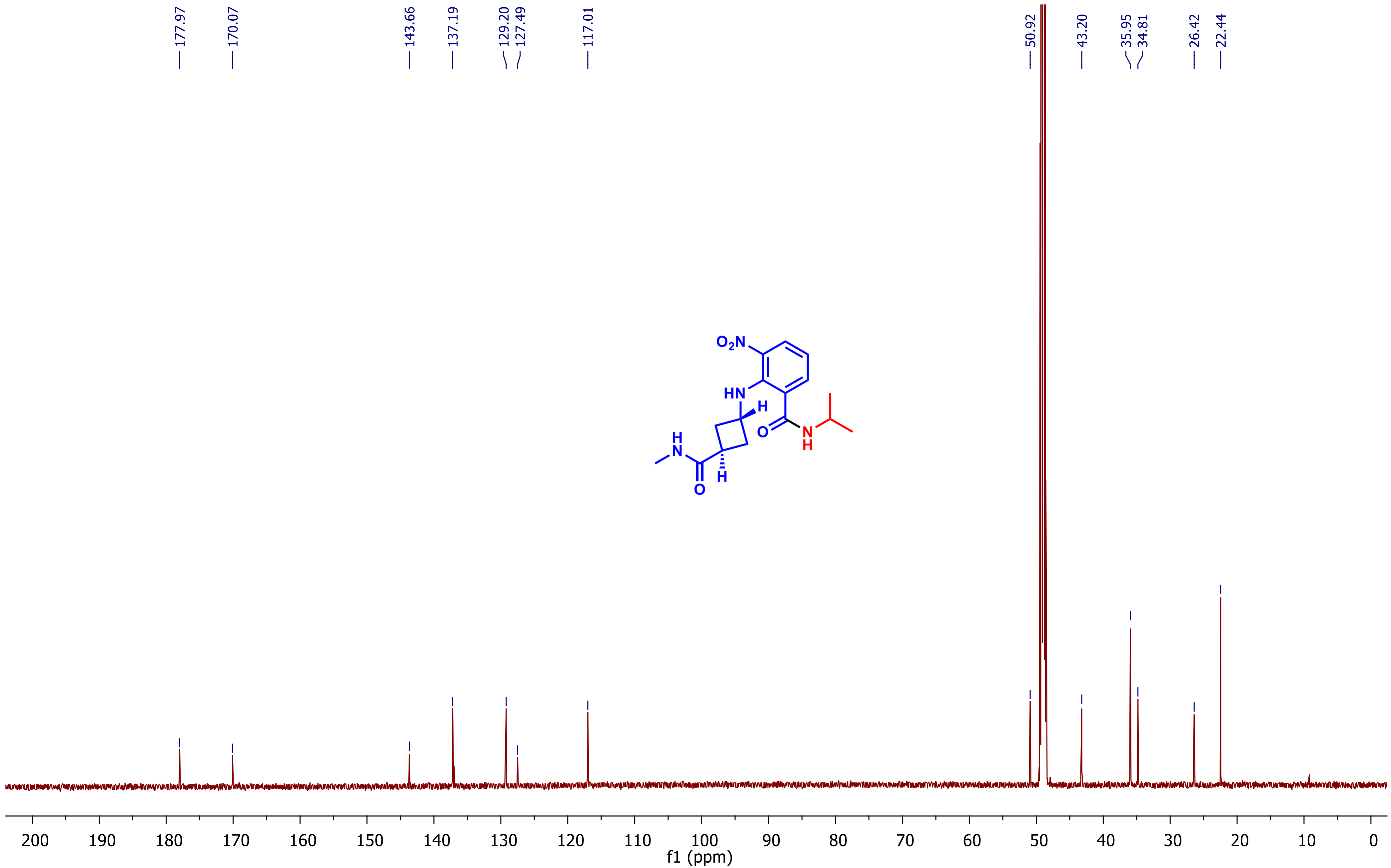
177.97
170.07
143.66
137.19
129.20
127.49
117.01
50.92
43.20
35.95
34.81
26.42
22.44
O2N
HN
H
H
N
O
H
O
N
H
200 190 180 170 160 150 140 130 120 110 100 90 80 70 60 50 40 30 20 10 0
f1 (ppm)

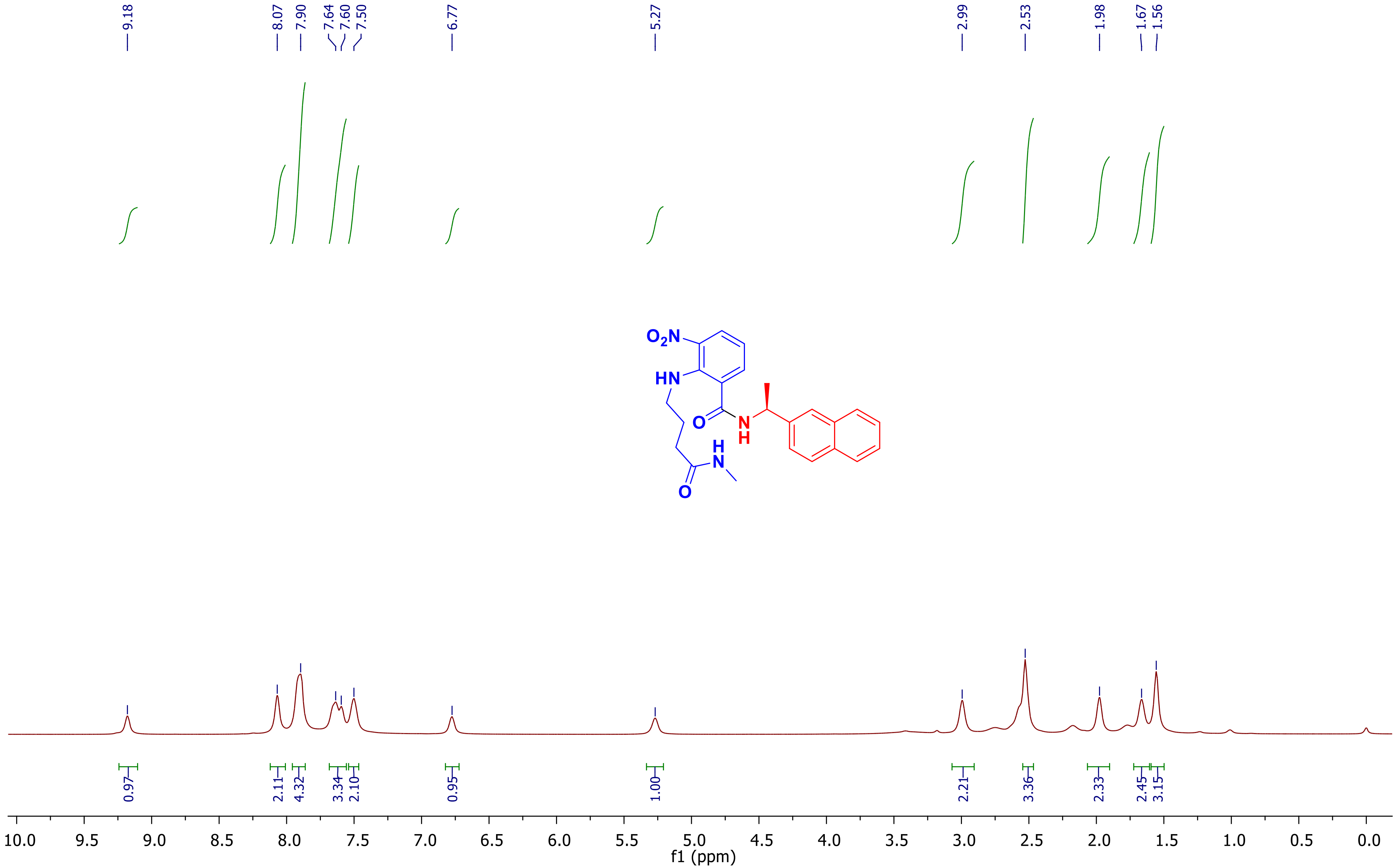
9.18
8.07
7.90
7.64
7.60
7.50
6.77
5.27
2.99
2.53
1.98
1.67
1.56
O2N
HN
O
N
H
H
N
O
0.97
2.11
4.32
3.34
2.10
0.95
1.00
2.21
3.36
2.33
2.45
3.15
10.0
9.5
9.0
8.5
8.0
7.5
7.0
6.5
6.0
5.5
5.0
4.5
4.0
3.5
3.0
2.5
2.0
1.5
1.0
0.5
0.0
f1 (ppm)

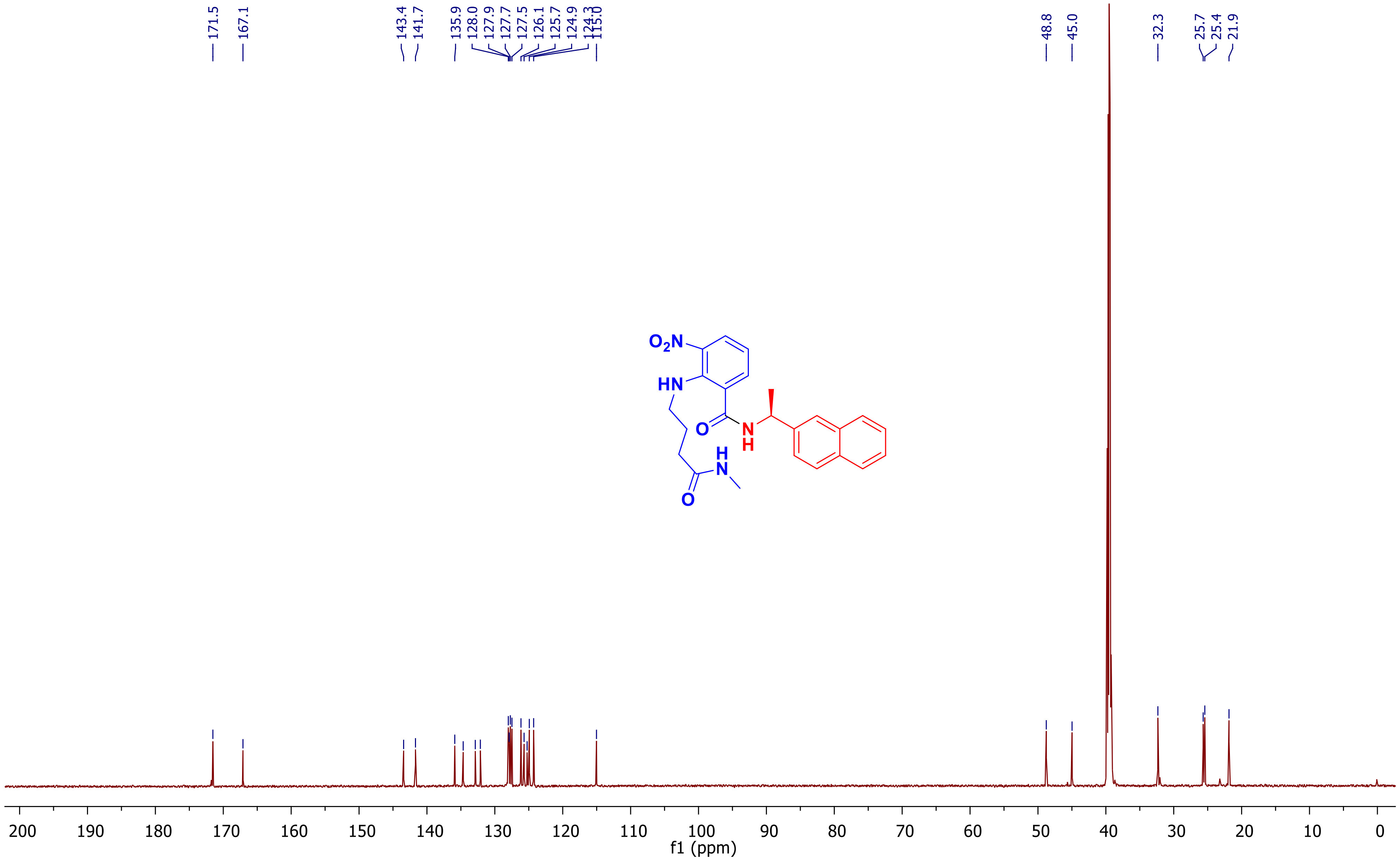

171.5
167.1
143.4
141.7
135.9
128.0
127.9
127.7
127.5
126.1
125.7
124.9
124.3
115.0
48.8
45.0
32.3
25.7
25.4
21.9
O2N
HN
O
N
H
H
N
O
200 190 180 170 160 150 140 130 120 110 100 90 80 70 60 50 40 30 20 10 0
f1 (ppm)

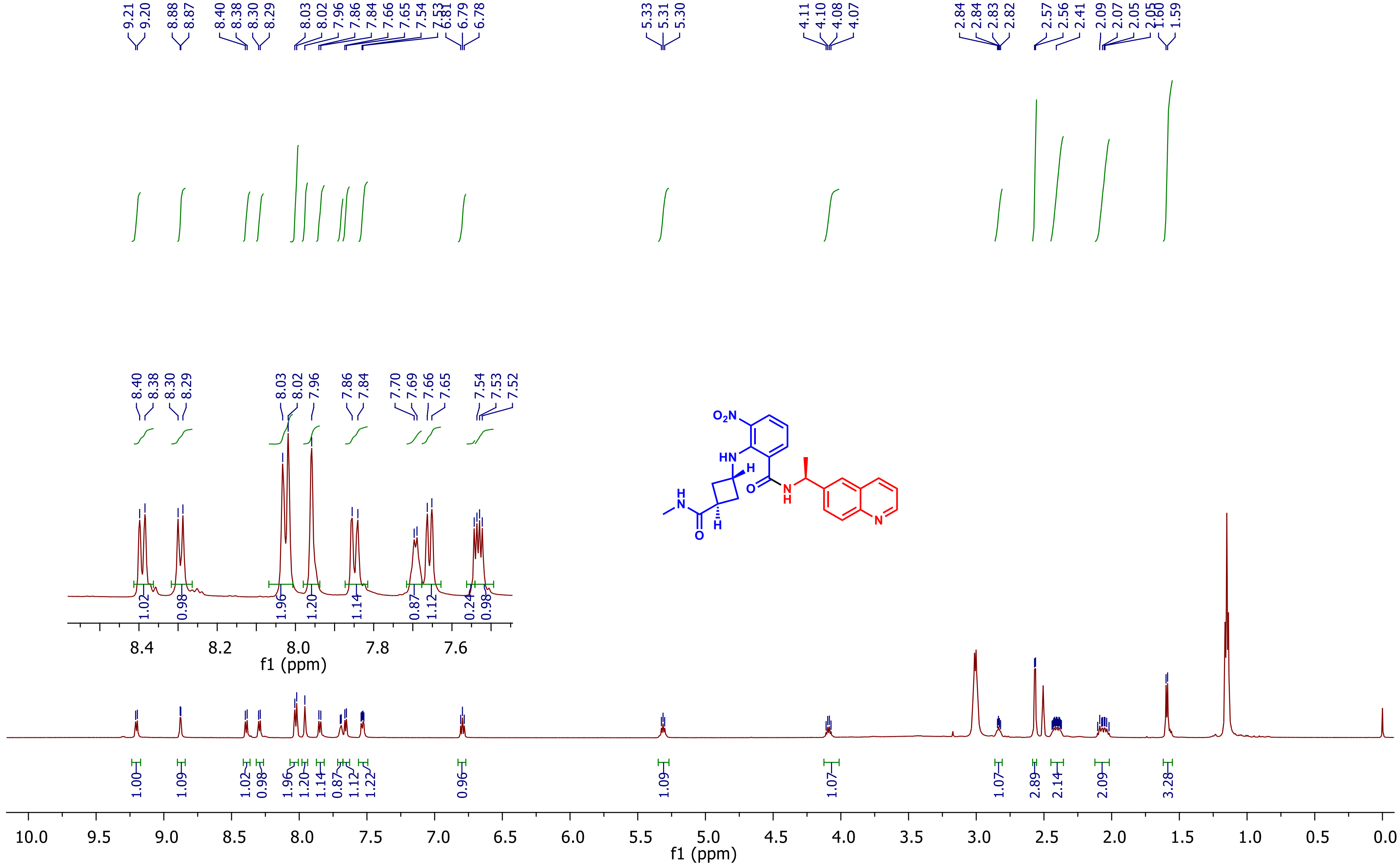
9.21 9.20 8.88 8.87 8.40 8.38 8.30 8.29 8.03 8.02 7.96 7.86 7.84 7.66 7.65 7.54 7.53 6.81 6.79 6.78 5.33 5.31 5.30 4.11 4.10 4.08 4.07 2.84 2.84 2.83 2.82 2.57 2.56 2.41 2.09 2.07 2.05 2.05 1.60 1.59
8.40 8.38 8.30 8.29 8.03 8.02 7.96 7.86 7.84 7.70 7.69 7.66 7.65 7.54 7.53 7.52
1.02 0.98 1.96 1.20 1.14 0.87 1.12 0.24 0.98
8.4 8.2 8.0 7.8 7.6
f1 (ppm)
O_2N
HN
H
N
O
1.00 1.09 1.02 0.98 1.96 1.20 1.14 0.87 1.12 1.22 0.96 1.09 1.07 1.07 2.89 2.14 2.09 3.28
10.0 9.5 9.0 8.5 8.0 7.5 7.0 6.5 6.0 5.5 5.0 4.5 4.0 3.5 3.0 2.5 2.0 1.5 1.0 0.5 0.0
f1 (ppm)

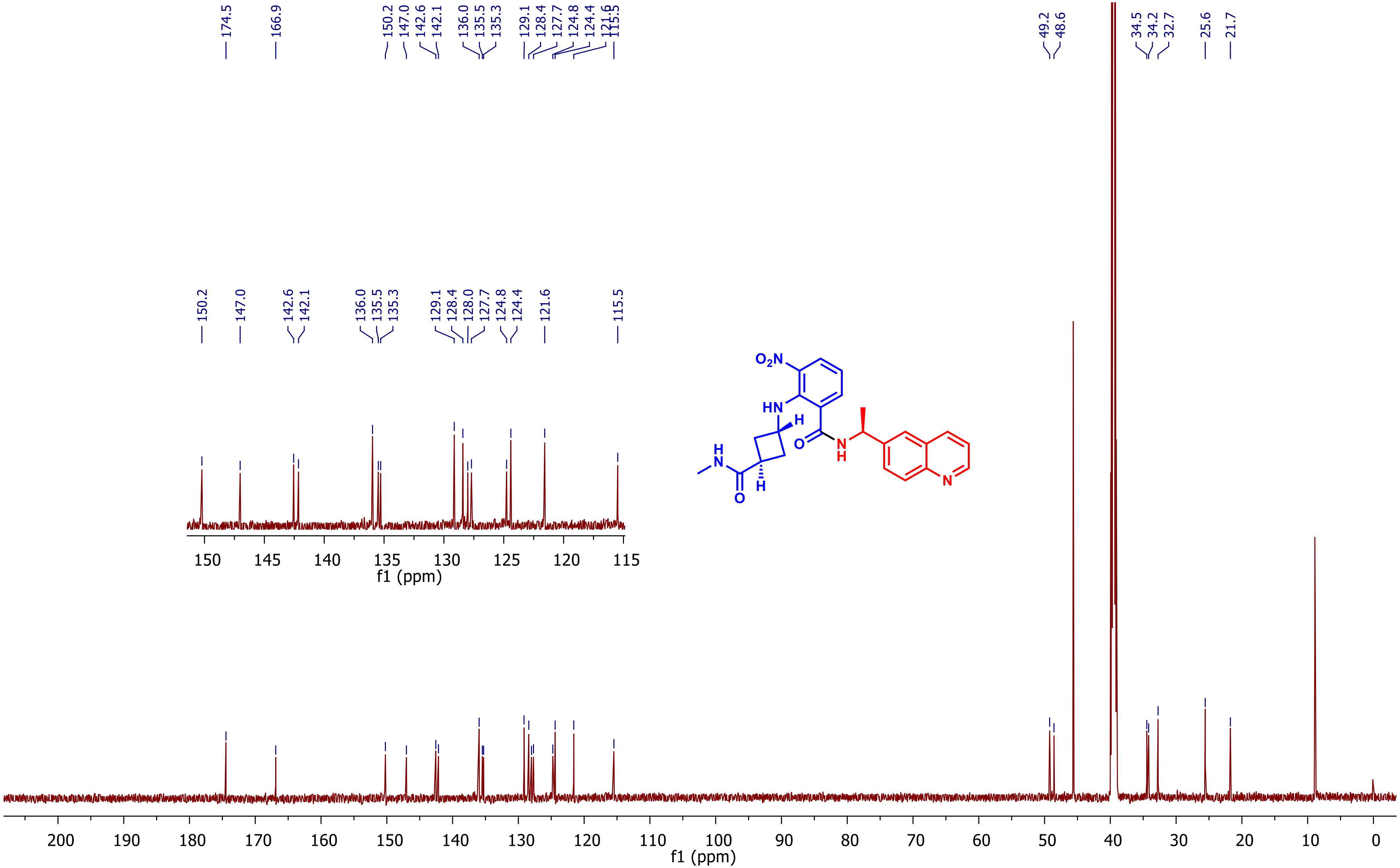
174.5
166.9
150.2
147.0
142.6
142.1
136.0
135.5
135.3
129.1
128.4
127.7
124.8
124.4
121.6
115.5
49.2
48.6
34.5
34.2
32.7
25.6
21.7
150.2
147.0
142.6
142.1
136.0
135.5
135.3
129.1
128.4
128.0
127.7
124.8
124.4
121.6
115.5
150 145 140 135 130 125 120 115
f1 (ppm)
O_2N
HN
H
H
N
O
O
N
H
H
N
200 190 180 170 160 150 140 130 120 110 100 90 80 70 60 50 40 30 20 10 0
f1 (ppm)

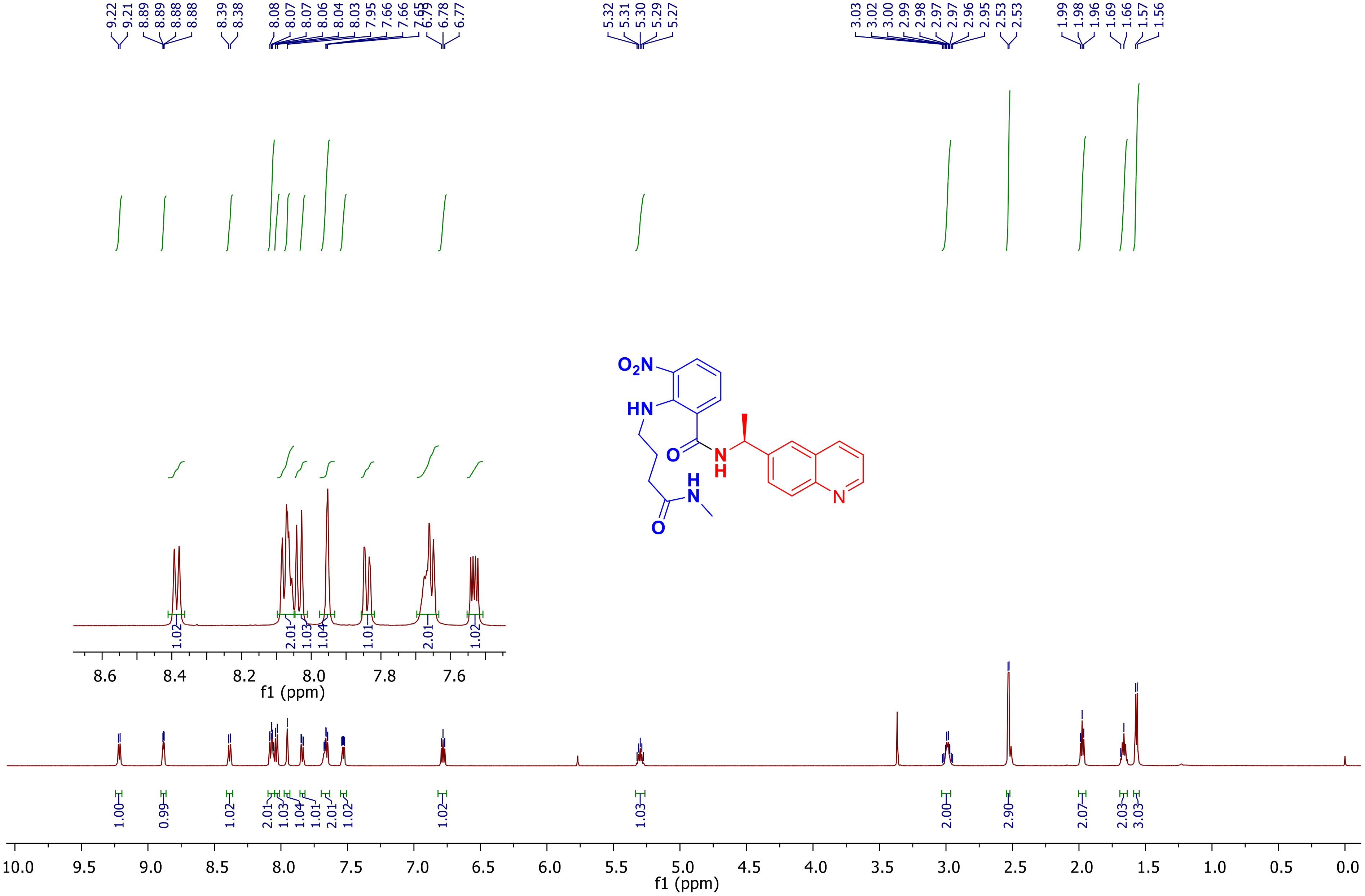

9.22 9.21 8.89 8.89 8.88 8.88 8.39 8.38 8.08 8.07 8.07 8.06 8.04 8.03 7.95 7.66 7.66 7.65 6.79 6.78 6.77
5.32 5.31 5.30 5.29 5.27
3.03 3.02 3.00 2.99 2.98 2.97 2.97 2.96 2.95 2.53 2.53
1.99 1.98 1.96 1.69 1.66 1.57 1.56
O_2N
HN
O
N
H
H
N
O
N
1.02 2.01 1.03 1.04 1.01 2.01 1.02
8.6 8.4 8.2 8.0 7.8 7.6
f1 (ppm)
1.00 0.99 1.02 2.01 1.03 1.04 1.01 2.01 1.02 1.02 1.03 2.00 2.90 2.07 2.03 3.03
10.0 9.5 9.0 8.5 8.0 7.5 7.0 6.5 6.0 5.5 5.0 4.5 4.0 3.5 3.0 2.5 2.0 1.5 1.0 0.5 0.0
f1 (ppm)

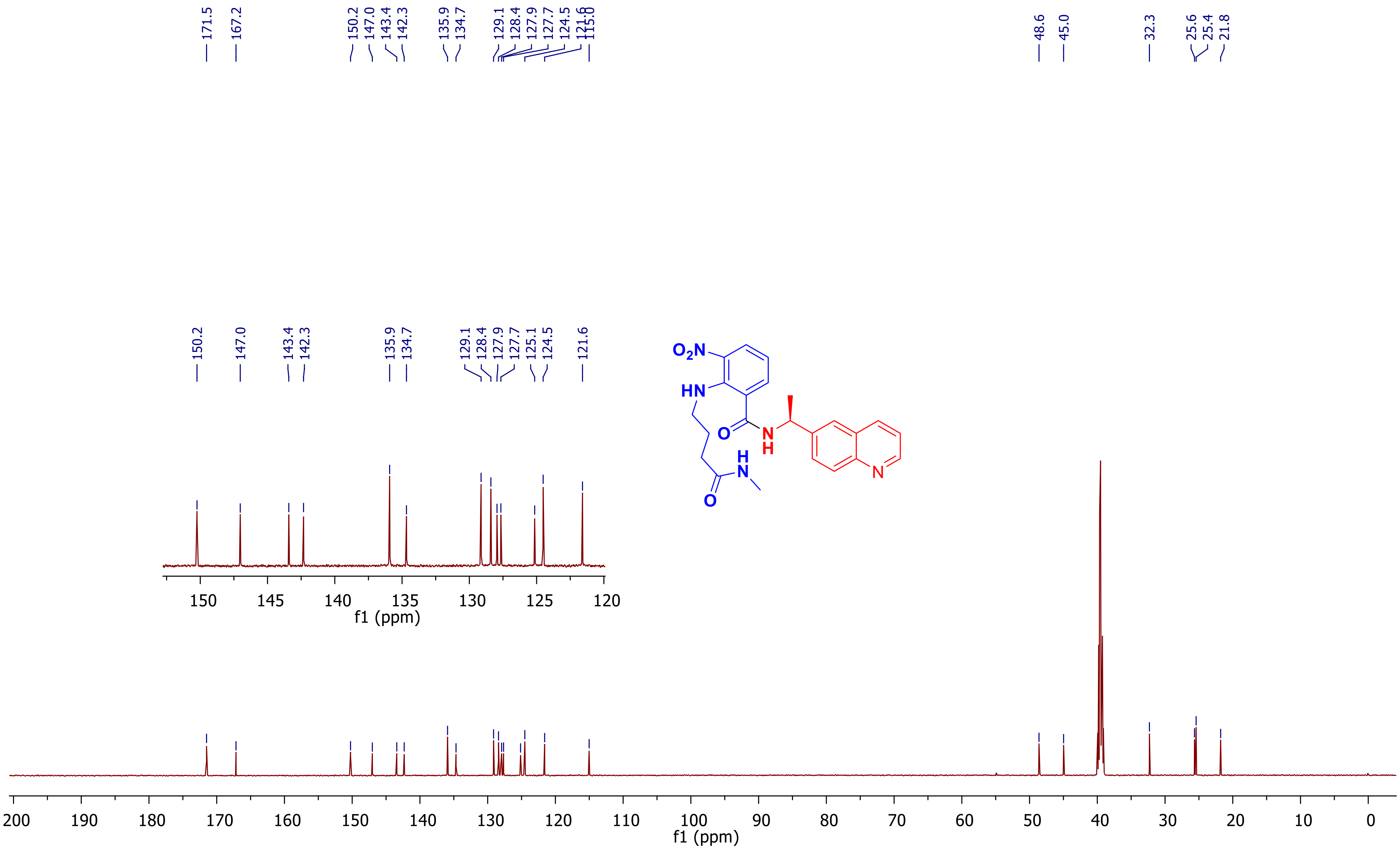
171.5
167.2
150.2
147.0
143.4
142.3
135.9
134.7
129.1
128.4
127.9
127.7
124.5
121.6
115.0
48.6
45.0
32.3
25.6
25.4
21.8
150.2
147.0
143.4
142.3
135.9
134.7
129.1
128.4
127.9
127.7
125.1
124.5
121.6
150
145
140
135
130
125
120
f1 (ppm)
O2N
HN
O
N
H
H
N
O
N
200
190
180
170
160
150
140
130
120
110
100
90
80
70
60
50
40
30
20
10
0
f1 (ppm)

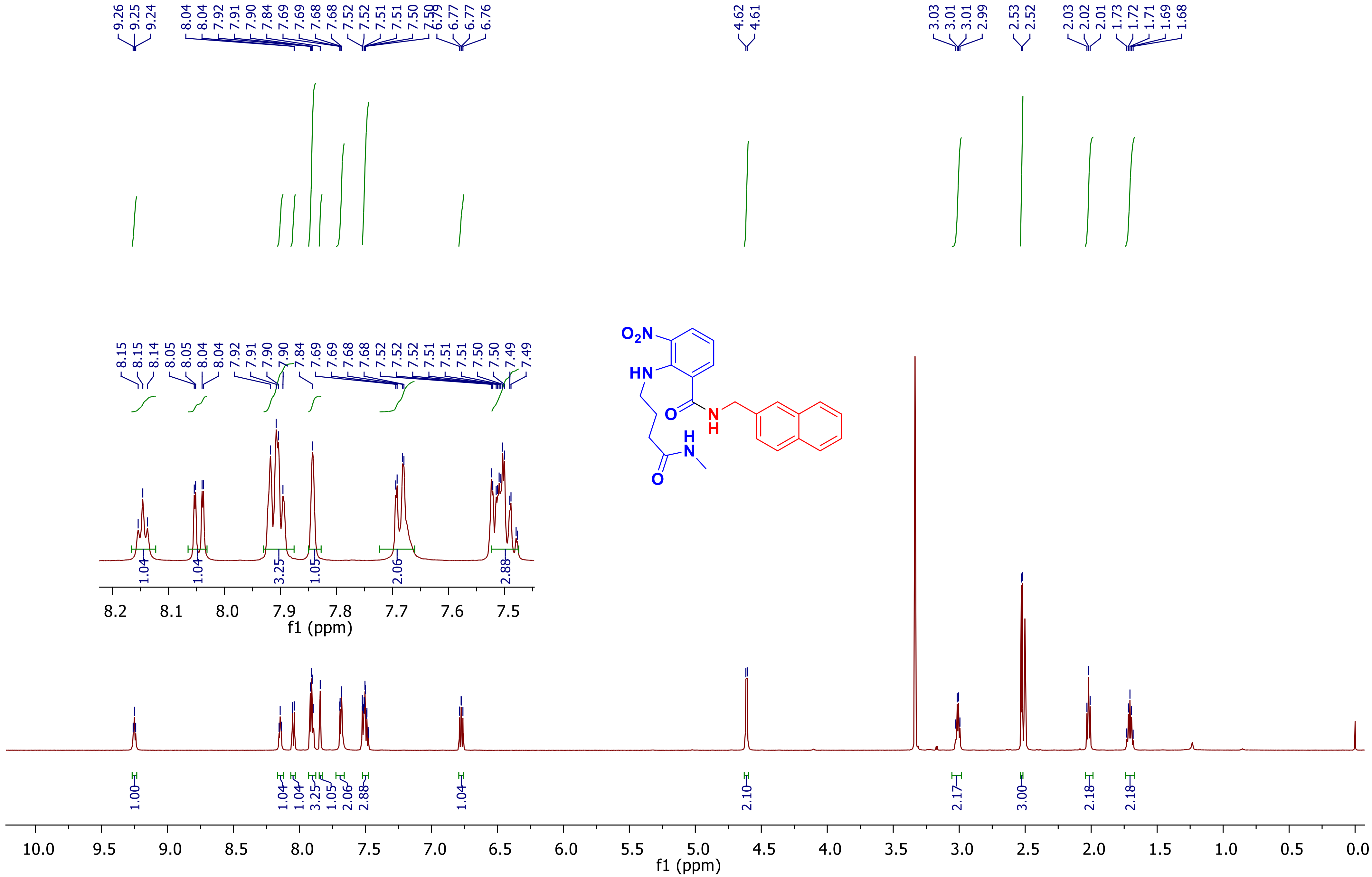
9.26 9.25 9.24 8.04 8.04 7.92 7.91 7.90 7.84 7.69 7.69 7.68 7.68 7.52 7.52 7.51 7.51 7.50 7.50 6.79 6.77 6.77 6.76
4.62 4.61
3.03 3.01 3.01 2.99 2.53 2.52 2.03 2.02 2.01 1.73 1.72 1.71 1.69 1.68
8.15 8.15 8.14 8.05 8.05 8.04 8.04 7.92 7.91 7.90 7.90 7.84 7.69 7.69 7.68 7.68 7.52 7.52 7.52 7.51 7.51 7.51 7.50 7.50 7.49 7.49
1.04 1.04 3.25 1.05 2.06 2.88
8.2 8.1 8.0 7.9 7.8 7.7 7.6 7.5
f1 (ppm)
O2N
HN
O
N
H
H
N
O
1.00 1.04 1.04 3.25 1.05 2.06 2.88 1.04 2.10 2.17 3.00 2.18 2.18
10.0 9.5 9.0 8.5 8.0 7.5 7.0 6.5 6.0 5.5 5.0 4.5 4.0 3.5 3.0 2.5 2.0 1.5 1.0 0.5 0.0
f1 (ppm)

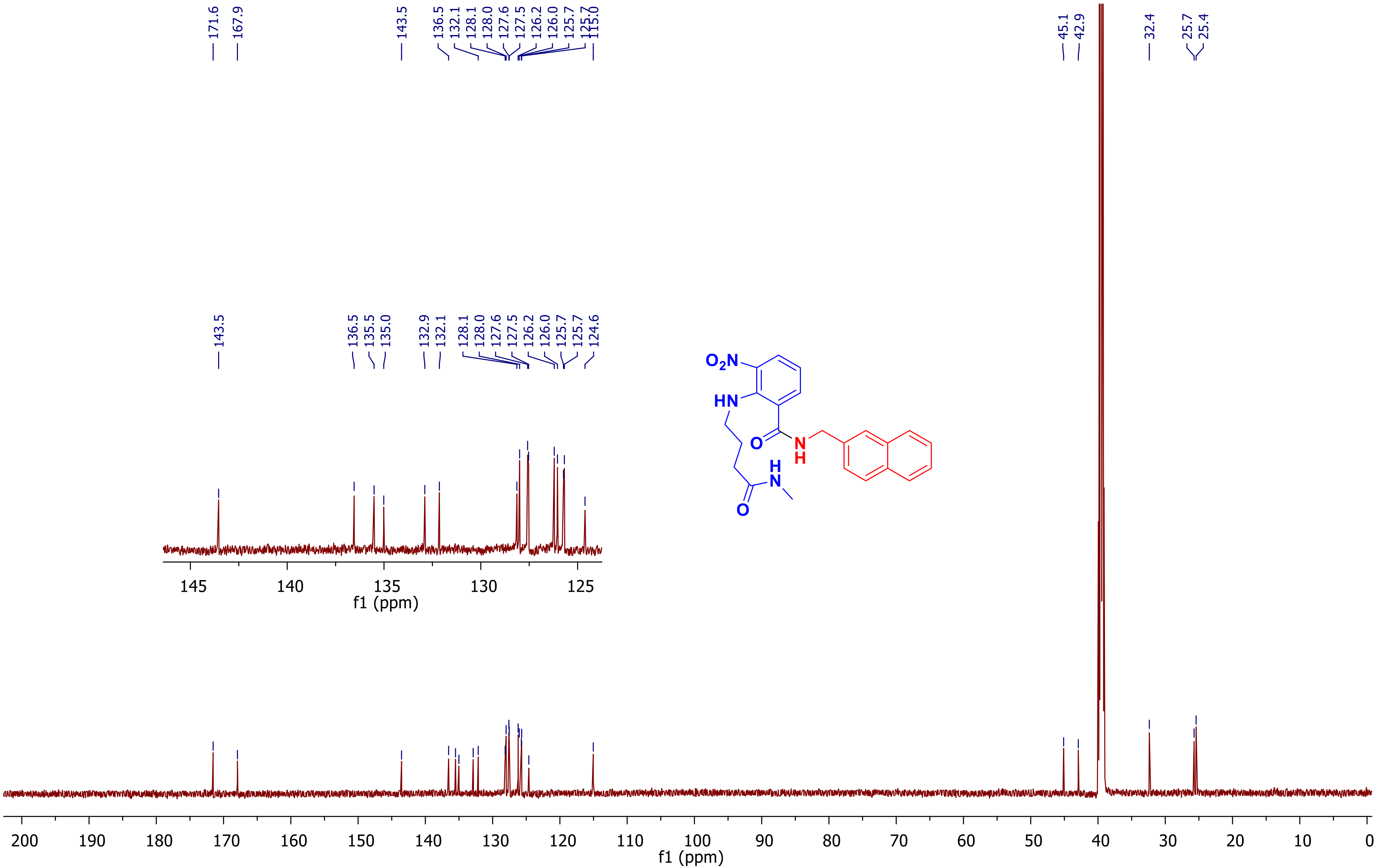
171.6
167.9
143.5
136.5
132.1
128.1
128.0
127.6
127.5
126.2
126.0
125.7
125.7
115.0
45.1
42.9
32.4
25.7
25.4
143.5
136.5
135.5
135.0
132.9
132.1
128.1
128.0
127.6
127.5
126.2
126.0
125.7
125.7
124.6
145
140
135
130
125
f1 (ppm)
O2N
HN
O
N
H
H
N
O
200
190
180
170
160
150
140
130
120
110
100
90
80
70
60
50
40
30
20
10
0
f1 (ppm)

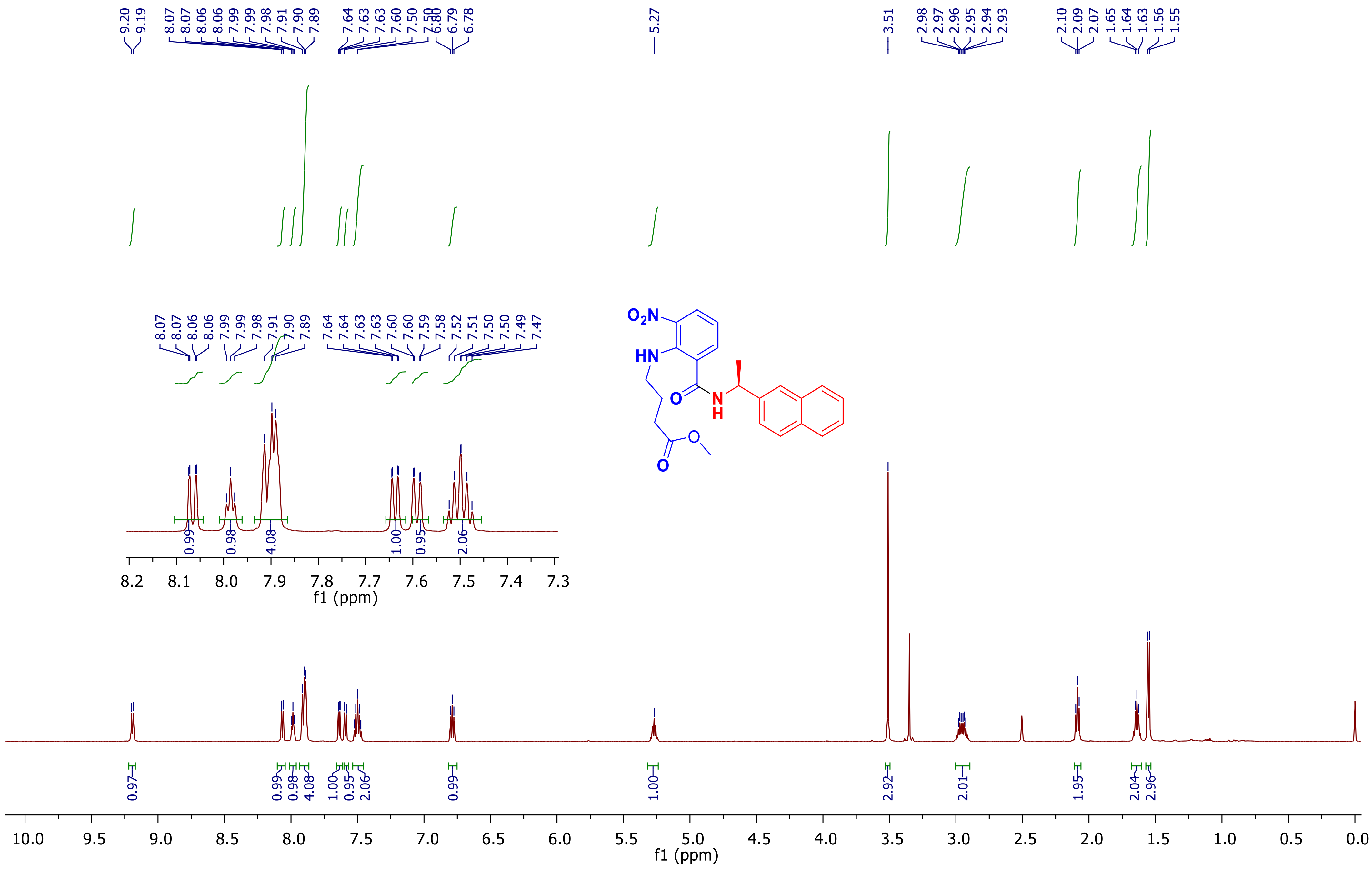
9.20
9.19
8.07
8.07
8.06
8.06
7.99
7.99
7.98
7.91
7.90
7.89
7.64
7.63
7.63
7.60
7.50
7.50
6.80
6.79
6.78
5.27
3.51
2.98
2.97
2.96
2.95
2.94
2.93
2.10
2.09
2.07
1.65
1.64
1.63
1.56
1.55
O2N
HN
O
N
H
O
O
0.99
0.98
4.08
1.00
0.95
2.06
8.2
8.1
8.0
7.9
7.8
7.7
7.6
7.5
7.4
7.3
f1 (ppm)
0.97
0.99
0.98
4.08
1.00
0.95
2.06
0.99
1.00
2.92
2.01
1.95
2.04
2.96
10.0
9.5
9.0
8.5
8.0
7.5
7.0
6.5
6.0
5.5
5.0
4.5
4.0
3.5
3.0
2.5
2.0
1.5
1.0
0.5
0.0
f1 (ppm)

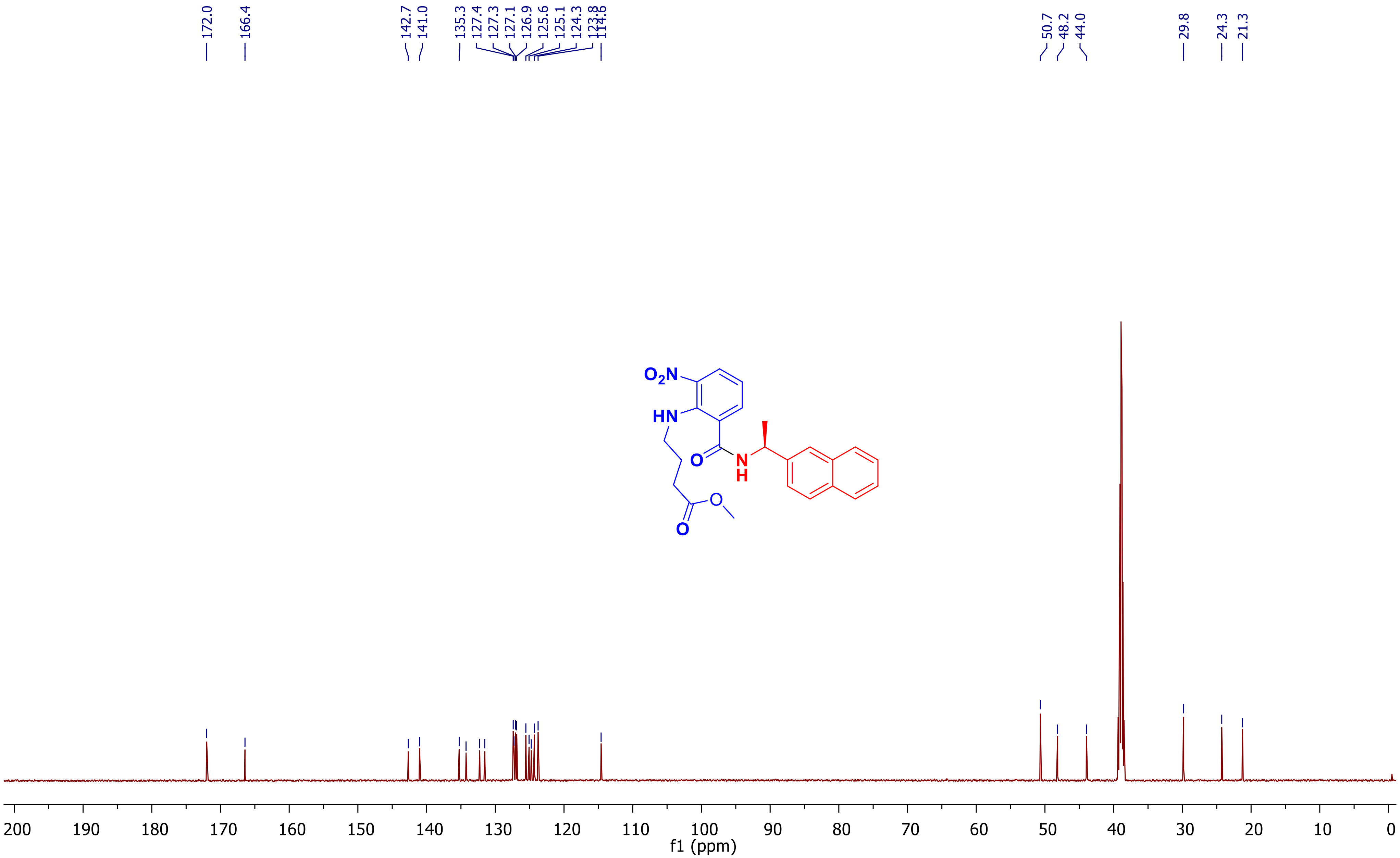
172.0
166.4
142.7
141.0
135.3
127.4
127.3
127.1
126.9
125.6
125.1
124.3
123.8
114.6
50.7
48.2
44.0
29.8
24.3
21.3
O_2N
HN
O
N
H
O
O
200 190 180 170 160 150 140 130 120 110 100 90 80 70 60 50 40 30 20 10 0
f1 (ppm)

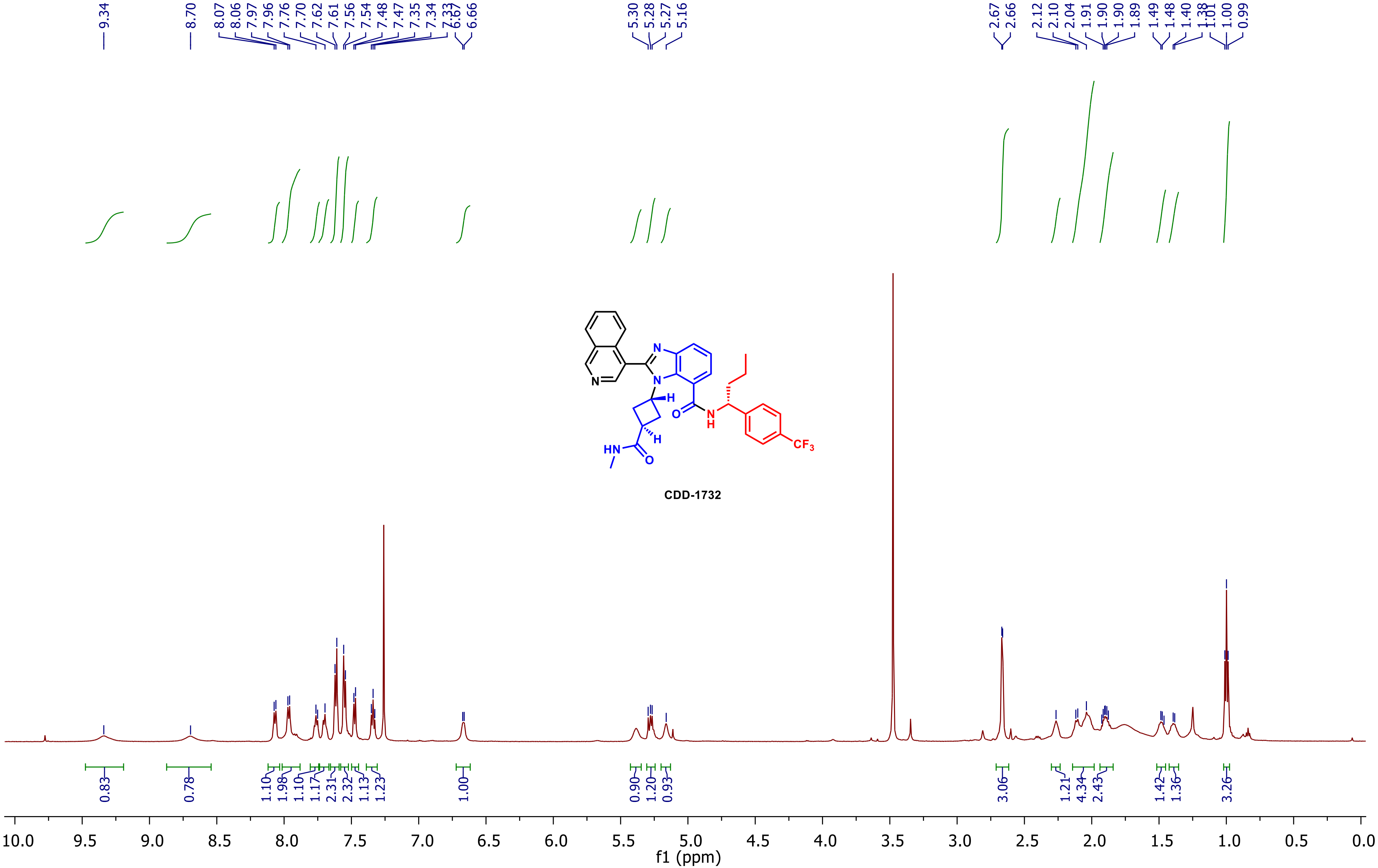

9.34
8.70
8.07 8.06 7.97 7.96 7.76 7.70 7.62 7.61 7.56 7.54 7.48 7.47 7.35 7.34 7.33 6.67 6.66
5.30 5.28 5.27 5.16
2.67 2.66 2.12 2.10 2.04 1.91 1.90 1.90 1.89 1.49 1.48 1.40 1.38 1.01 1.00 0.99
N
N
N
H
O
N
H
H
HN
O
CF_3
CDD-1732
0.83 0.78 1.10 1.98 1.10 1.17 2.31 2.32 1.13 1.23 1.00 0.90 1.20 0.93 3.06 1.21 4.34 2.43 1.42 1.36 3.26
10.0 9.5 9.0 8.5 8.0 7.5 7.0 6.5 6.0 5.5 5.0 4.5 4.0 3.5 3.0 2.5 2.0 1.5 1.0 0.5 0.0
f1 (ppm)

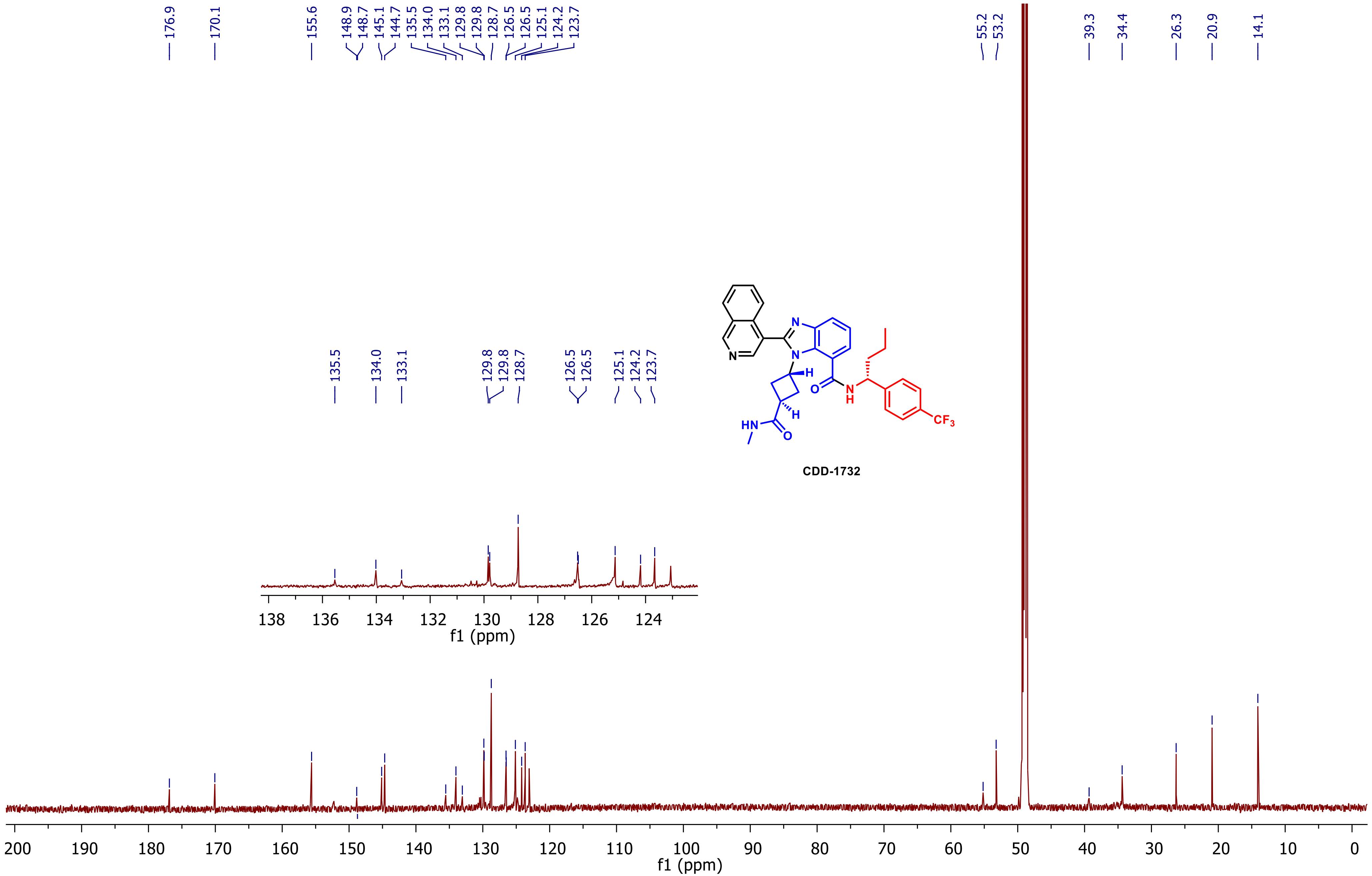
176.9
170.1
155.6
148.9
148.7
145.1
144.7
135.5
134.0
133.1
129.8
129.8
128.7
126.5
126.5
125.1
124.2
123.7
55.2
53.2
39.3
34.4
26.3
20.9
14.1
f1 (ppm)
CDD-1732
N
N
N
H
H
O
N
H
HN
O
CF3

C:\Analytical\...\10162020\CDD1732 10/16/20 10:43:14

RT: 0.00 - 7.00 SM: 7B

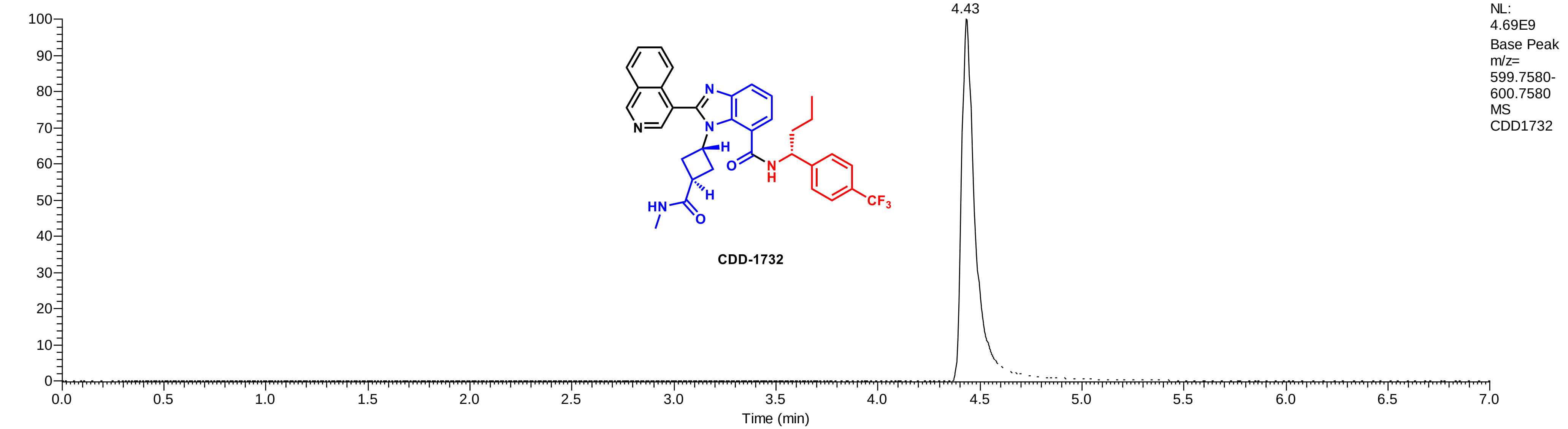

CDD1732 #573 RT: 4.43 AV: 1 NL: 5.10E9
T: FTMS + p ESI Full ms [100.0000-1000.0000]

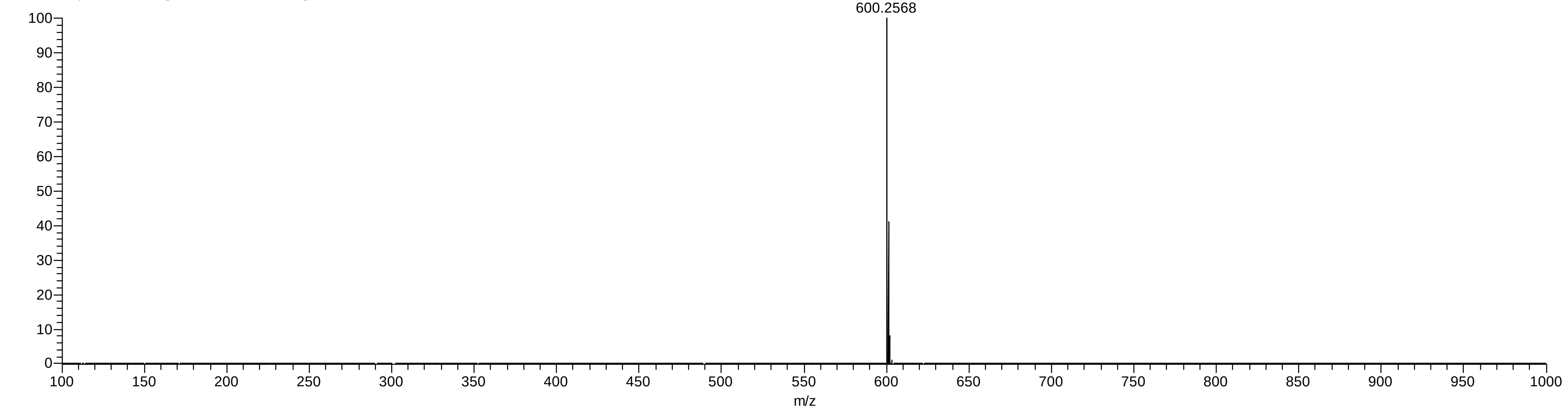

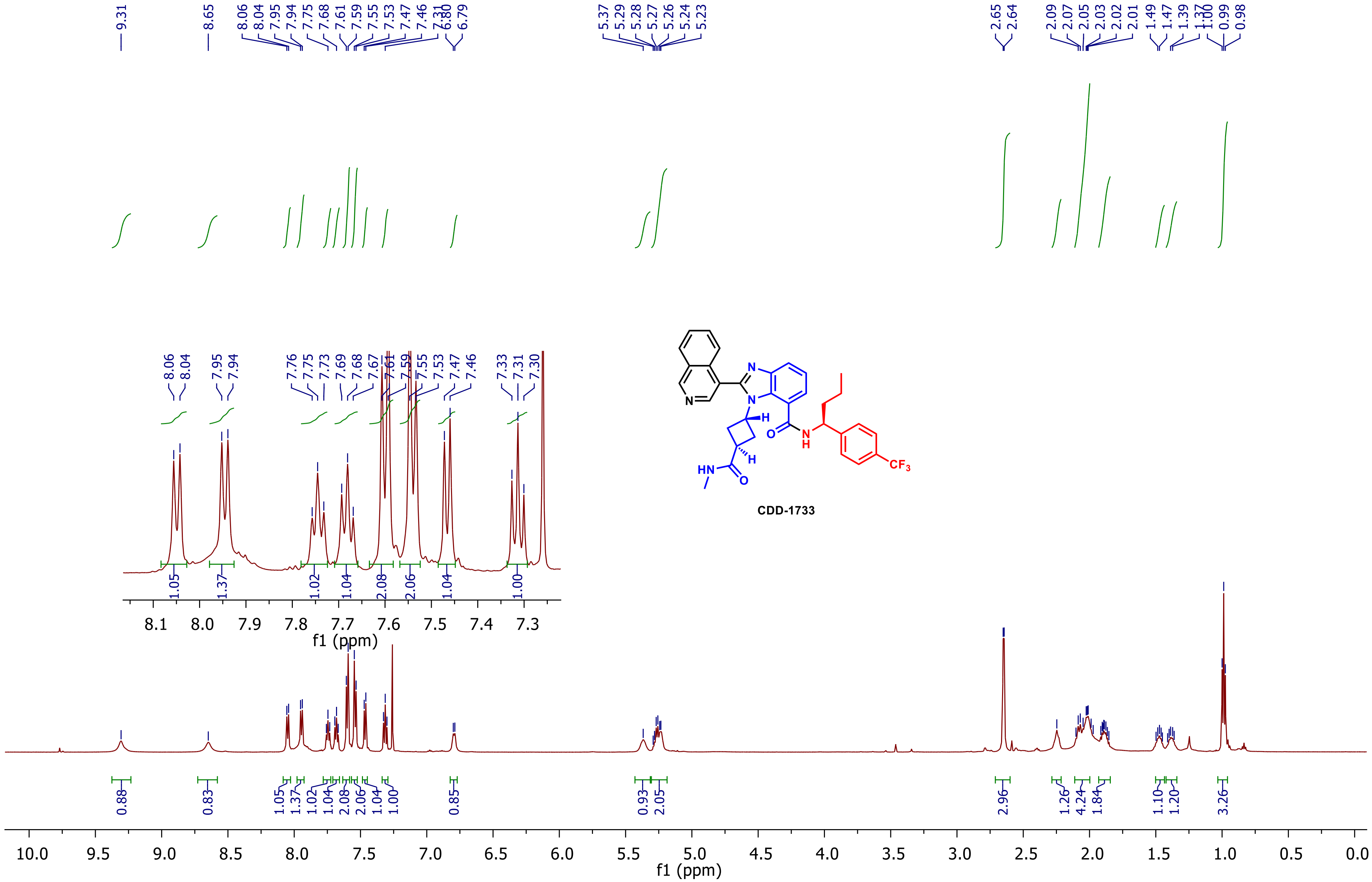
9.31
8.65
8.06 8.04 7.95 7.94 7.75 7.68 7.61 7.59 7.55 7.53 7.47 7.46 7.31 6.80 6.79
5.37 5.29 5.28 5.27 5.26 5.24 5.23
2.65 2.64
2.09 2.07 2.05 2.03 2.02 2.01 1.49 1.47 1.39 1.37 1.00 0.99 0.98
8.06 8.04 7.95 7.94 7.76 7.75 7.73 7.69 7.68 7.67 7.61 7.59 7.55 7.53 7.47 7.46 7.33 7.31 7.30
1.05 1.37 1.02 1.04 2.08 2.06 1.04 1.00
f1 (ppm)
N
N
N
H
H
O
N
H
HN
O
CF3
0.88 0.83 1.05 1.37 1.02 1.04 2.08 2.06 1.04 1.00 0.85 0.93 2.05 2.96 1.26 4.24 1.84 1.10 1.20 3.26
f1 (ppm)
CDD-1733

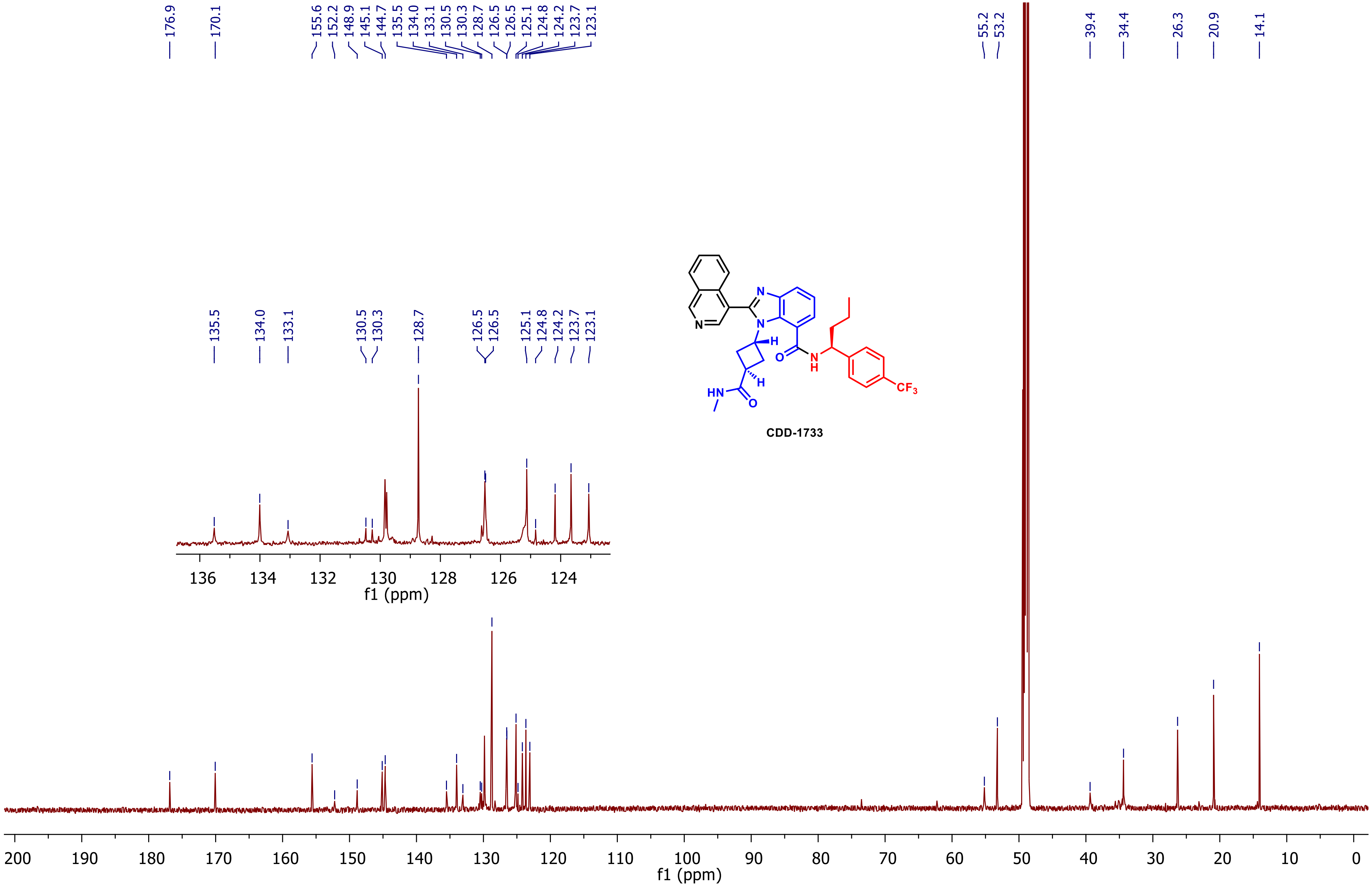

176.9
170.1
155.6
152.2
148.9
145.1
144.7
135.5
134.0
133.1
130.5
130.3
128.7
126.5
126.5
125.1
124.8
124.2
123.7
123.1
55.2
53.2
39.4
34.4
26.3
20.9
14.1
N
N
H
O
N
H
HN
O
H
CF3
CDD-1733
f1 (ppm)
f1 (ppm)

C:\Analytical\...\10132020\CDD1733 10/13/20 12:33:47

RT: 0.00 - 7.00 SM: 7B

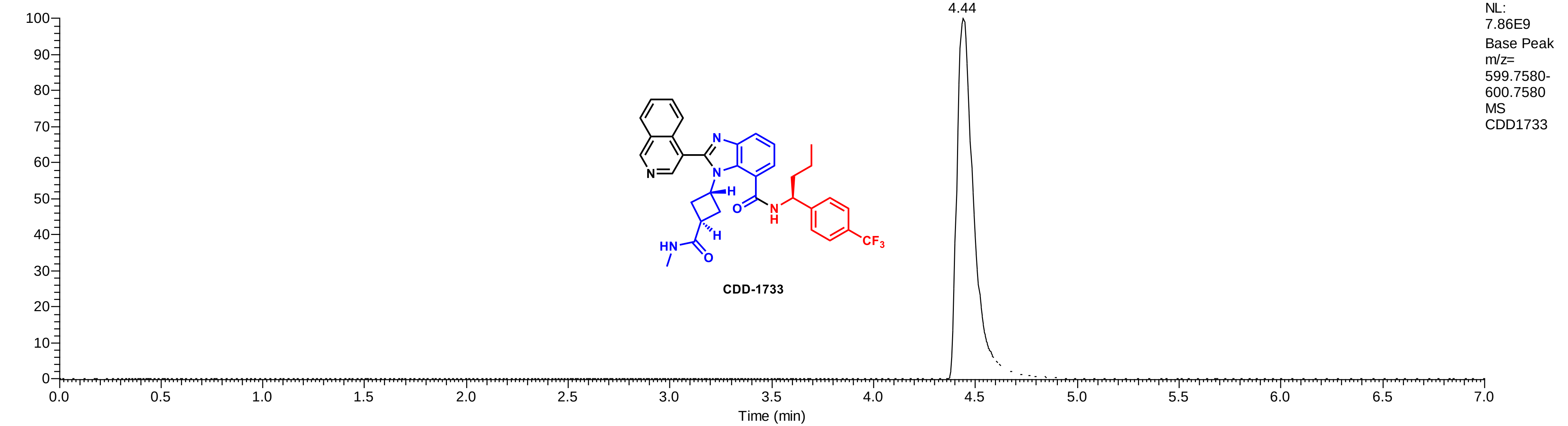

CDD1733 #660 RT: 4.44 AV: 1 NL: 8.21E9
T: FTMS + p ESI Full ms [100.0000-1000.0000]

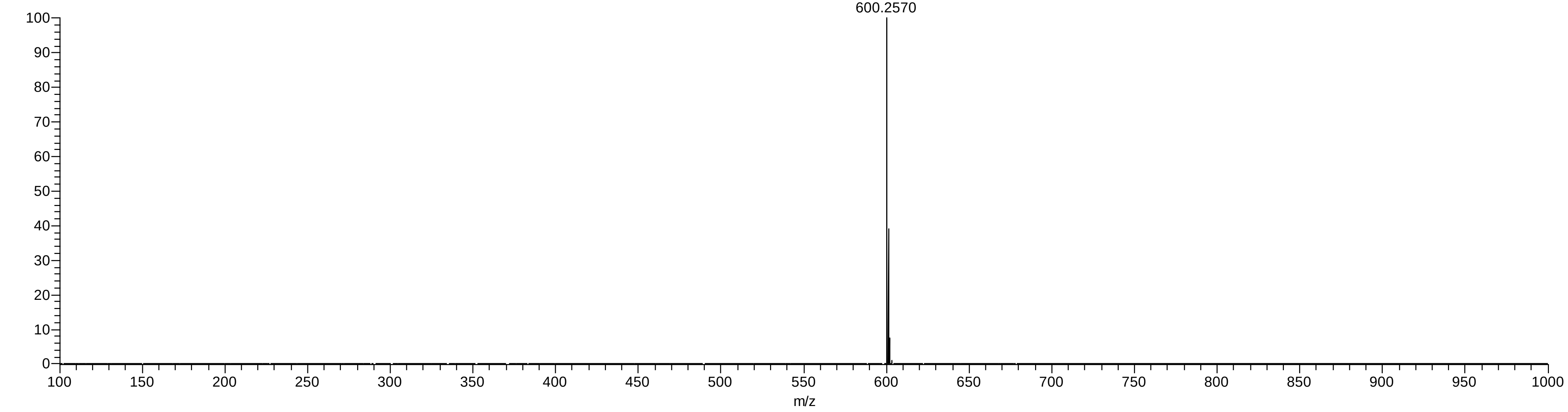

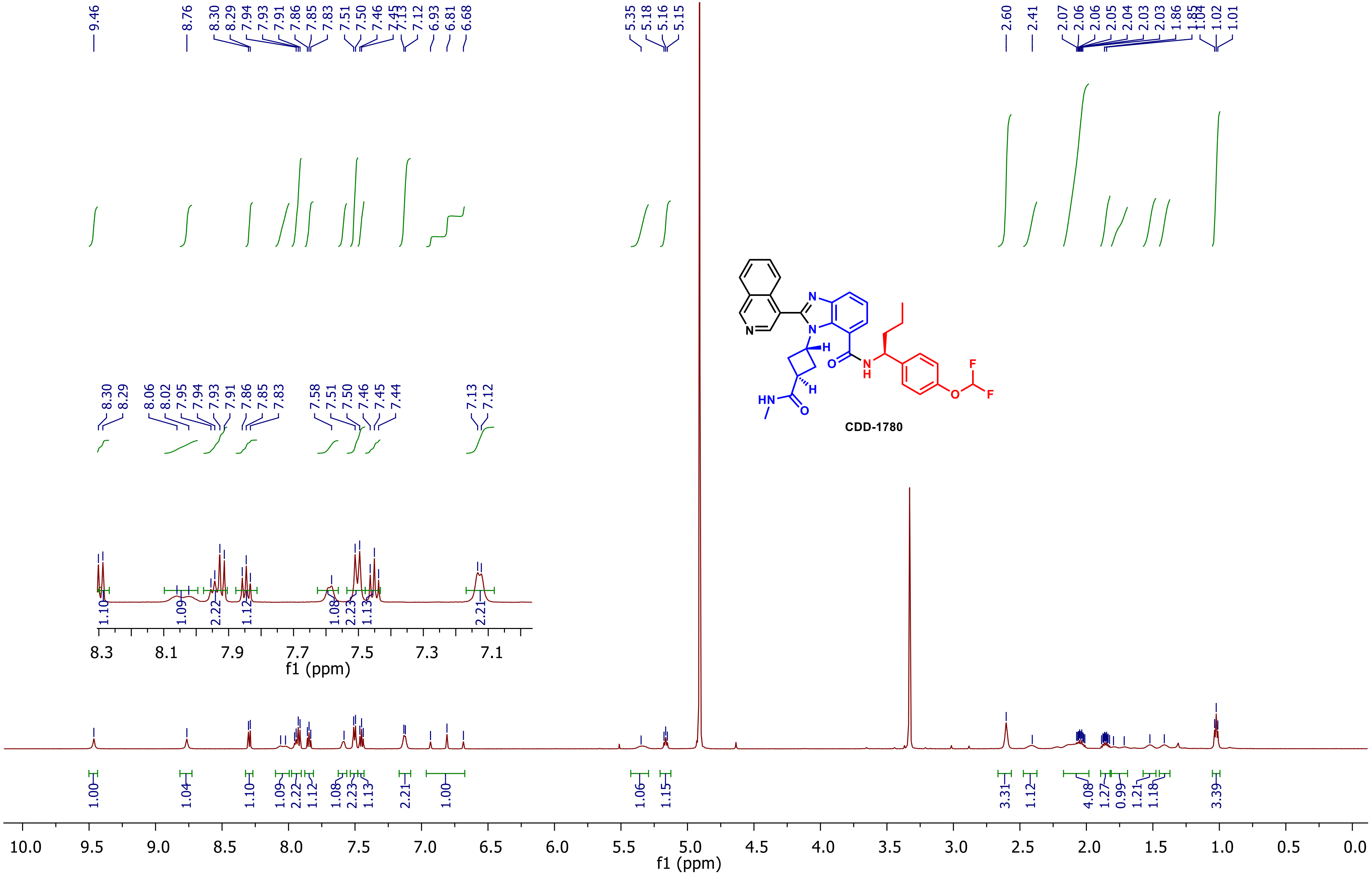

9.46
8.76
8.30
8.29
7.94
7.93
7.91
7.86
7.85
7.83
7.51
7.50
7.46
7.45
7.13
7.12
6.93
6.81
6.68
5.35
5.18
5.16
5.15
2.60
2.41
2.07
2.06
2.06
2.05
2.04
2.03
2.03
1.86
1.85
1.84
1.02
1.01
CDD-1780
f1 (ppm)

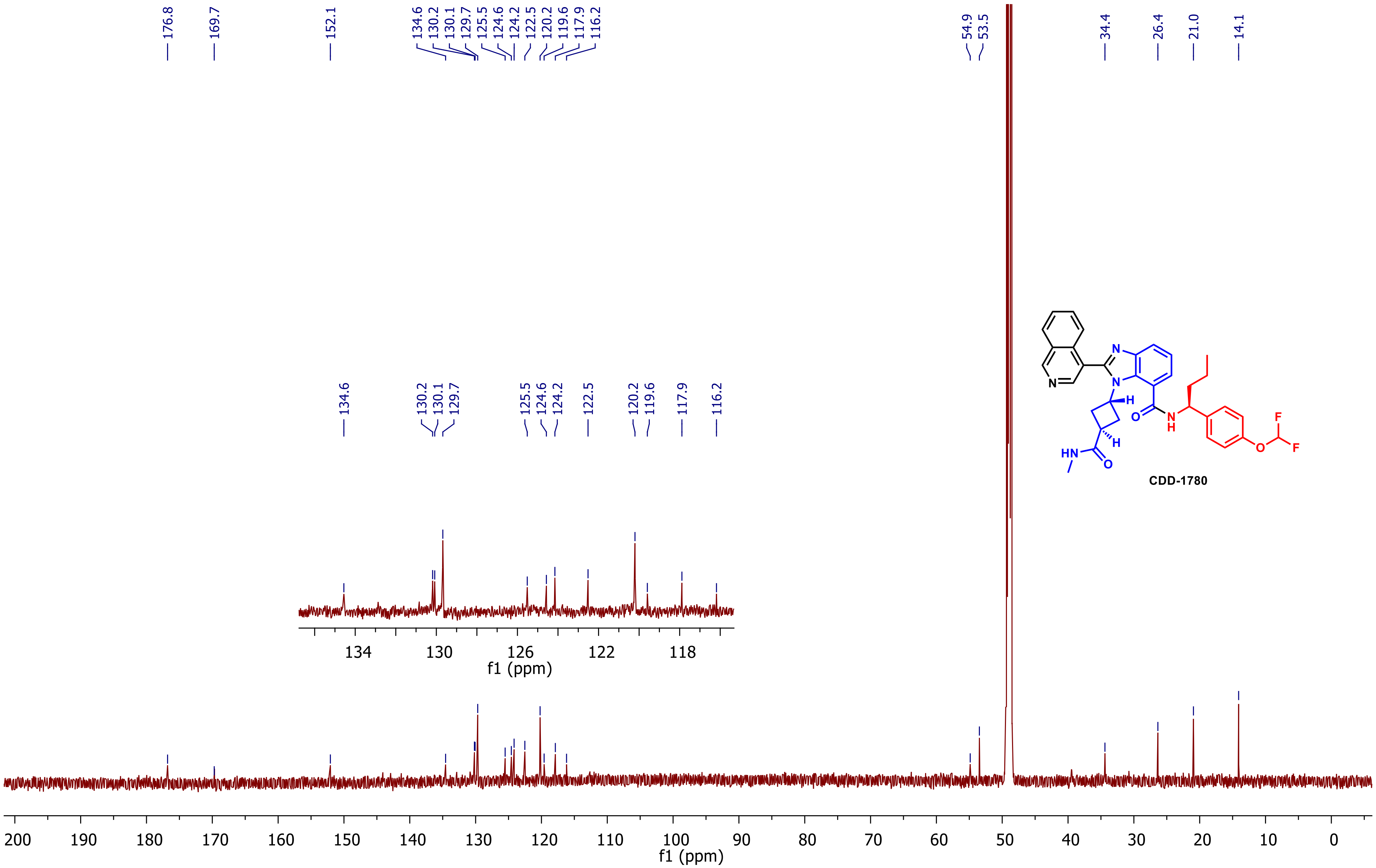
176.8
169.7
152.1
134.6
130.2
130.1
129.7
125.5
124.6
124.2
122.5
120.2
119.6
117.9
116.2
54.9
53.5
34.4
26.4
21.0
14.1
CDD-1780
f1 (ppm)

C:\Analytical\...\10122020\CDD1780 10/12/20 11:49:31

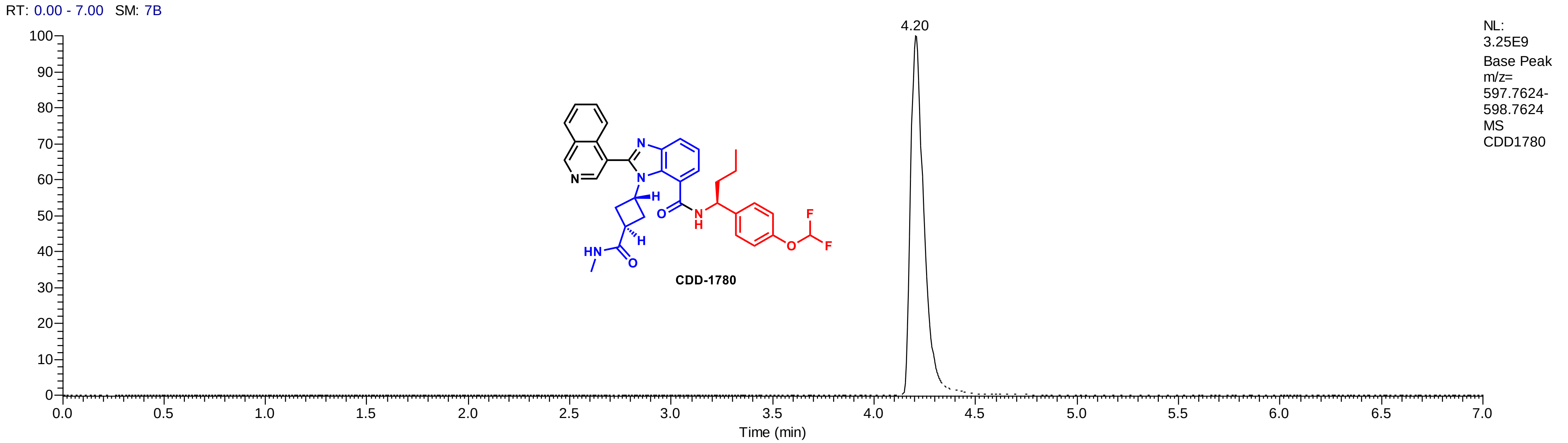

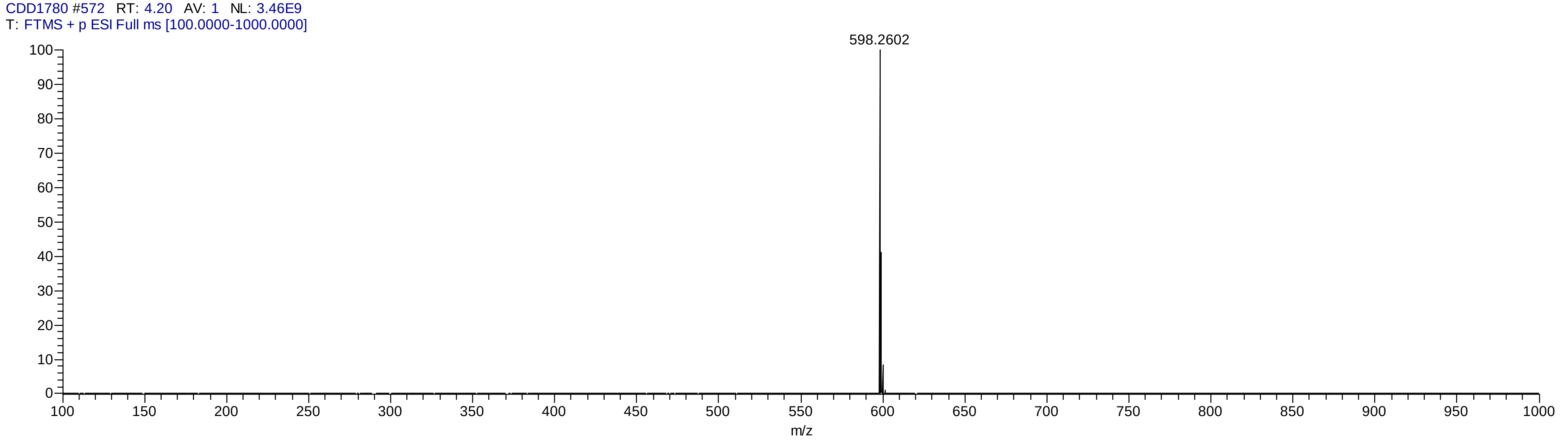

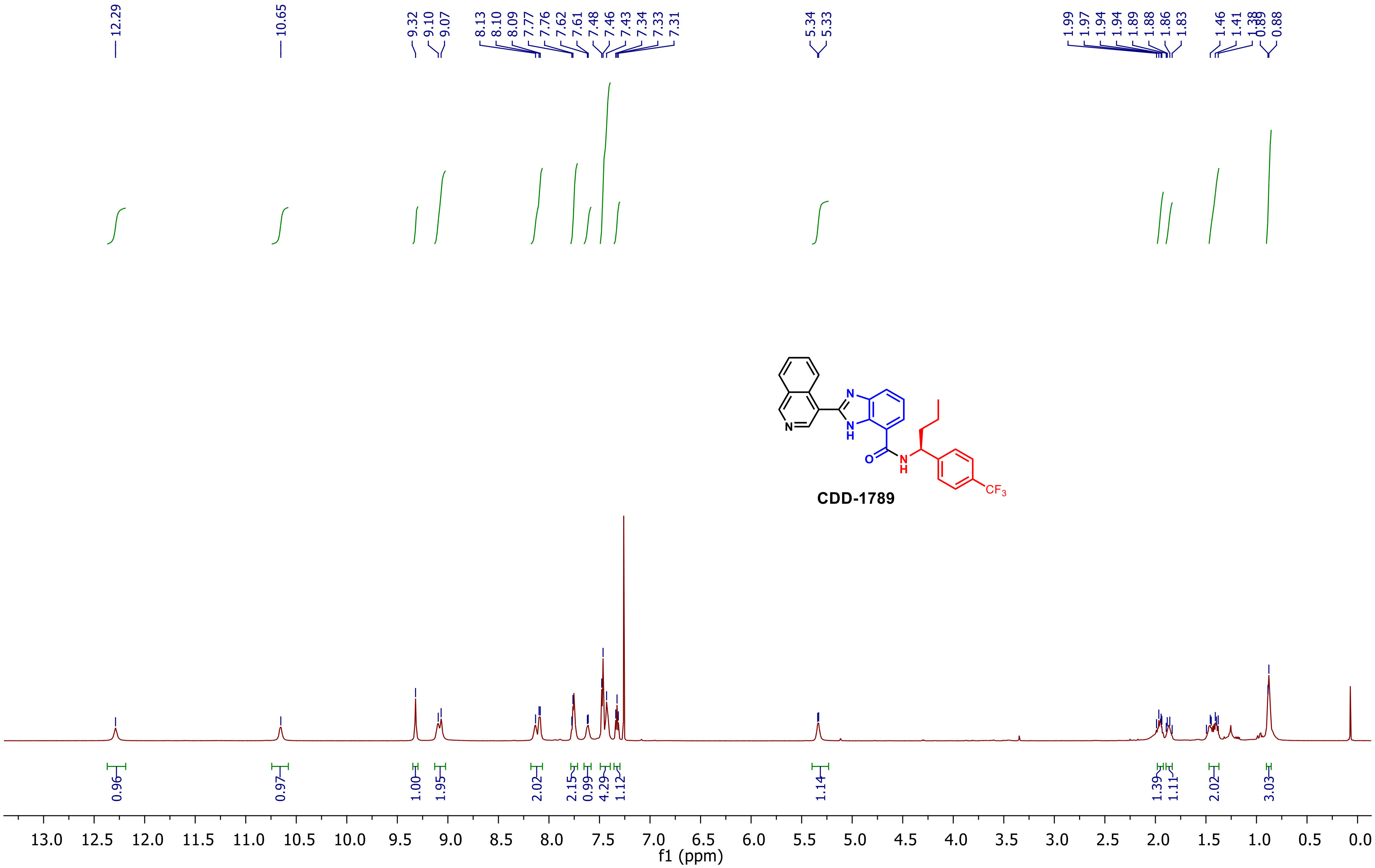

12.29
10.65
9.32
9.10
9.07
8.13
8.10
8.09
7.77
7.76
7.62
7.61
7.48
7.46
7.43
7.34
7.33
7.31
5.34
5.33
1.99
1.97
1.94
1.94
1.89
1.88
1.86
1.83
1.46
1.41
1.38
0.89
0.88
N
N
H
N
O
N
H
CF_3
CDD-1789
0.96
0.97
1.00
1.95
2.02
2.15
0.99
4.29
1.12
1.14
1.39
1.11
2.02
3.03
13.0 12.5 12.0 11.5 11.0 10.5 10.0 9.5 9.0 8.5 8.0 7.5 7.0 6.5 6.0 5.5 5.0 4.5 4.0 3.5 3.0 2.5 2.0 1.5 1.0 0.5 0.0
f1 (ppm)

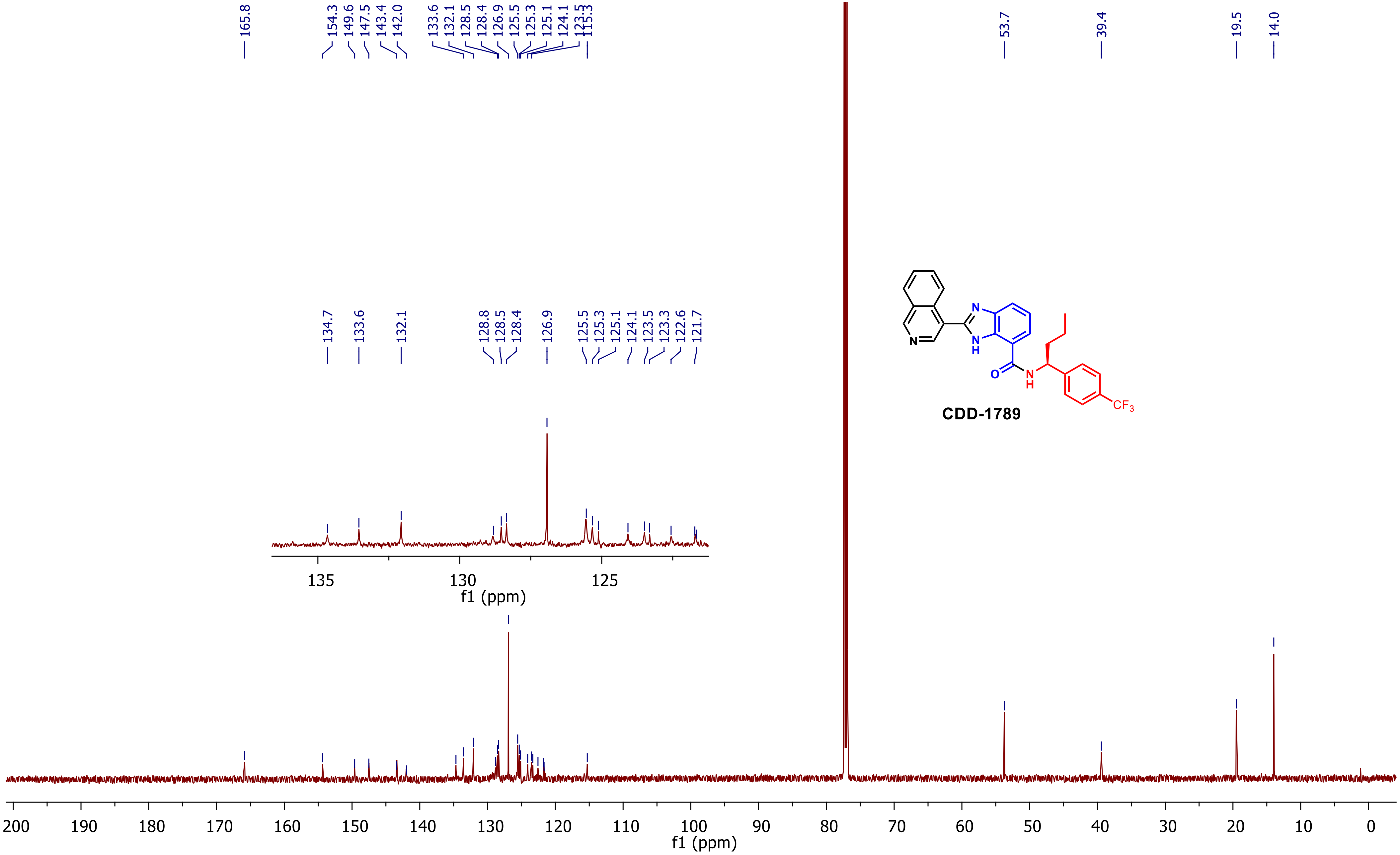
165.8
154.3
149.6
147.5
143.4
142.0
133.6
132.1
128.5
128.4
126.9
125.5
125.3
125.1
124.1
123.5
115.3
53.7
39.4
19.5
14.0
134.7
133.6
132.1
128.8
128.5
128.4
126.9
125.5
125.3
125.1
124.1
123.5
123.3
122.6
121.7
135
130
125
f1 (ppm)
200
190
180
170
160
150
140
130
120
110
100
90
80
70
60
50
40
30
20
10
0
f1 (ppm)
N
N
H
N
O
N
H
CF3

CDD-1789

C:\Analytical\Data\JRK\09252020\CDD1789 09/25/20 15:15:30

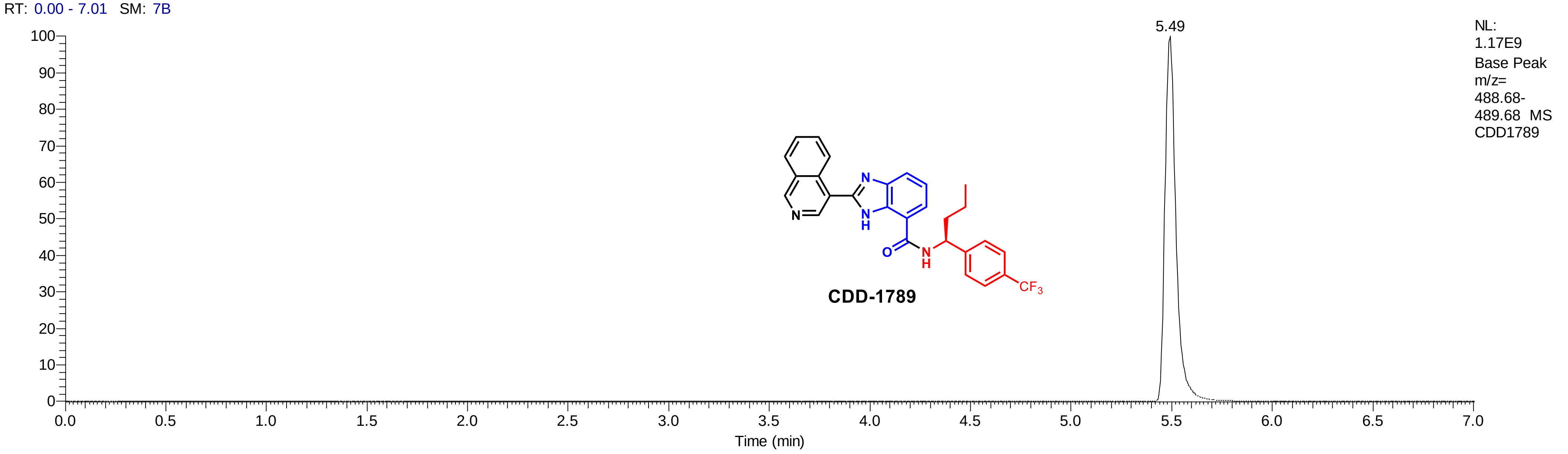

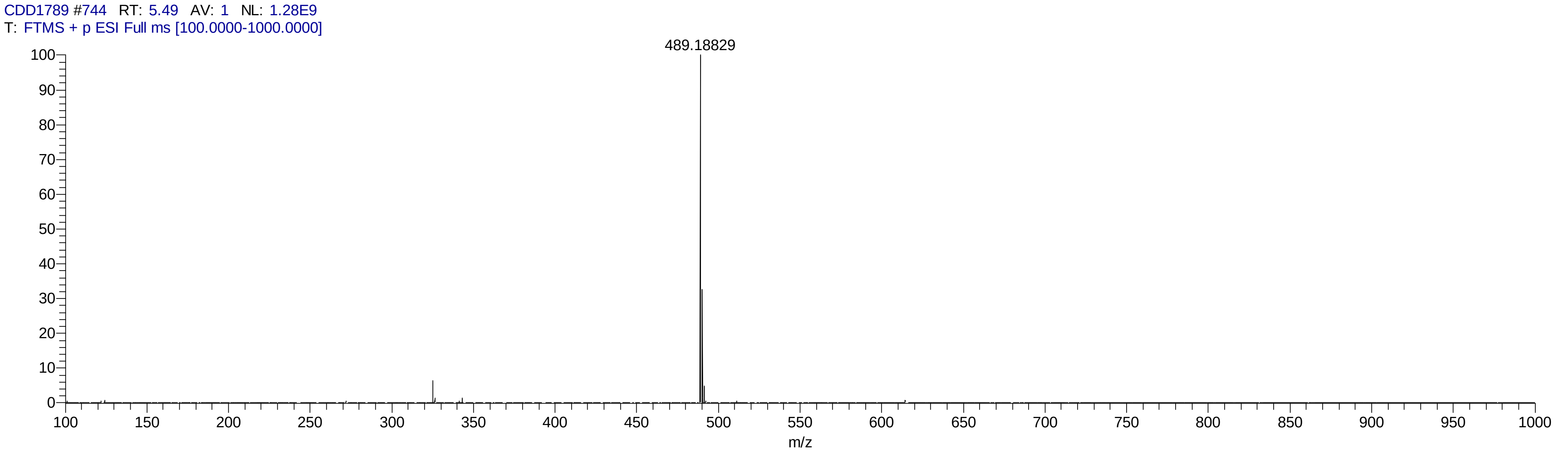

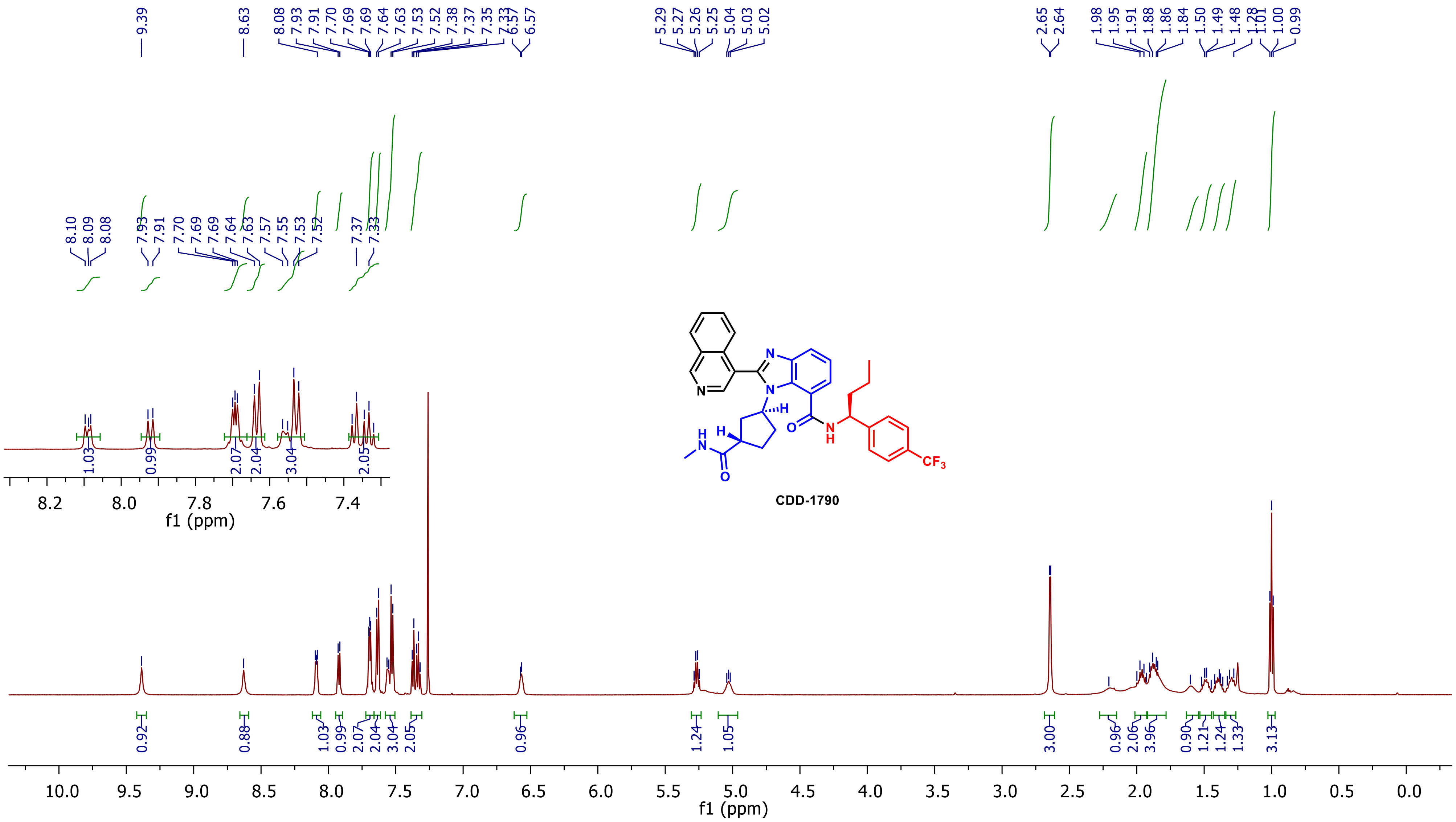

CDD-1790
f1 (ppm)

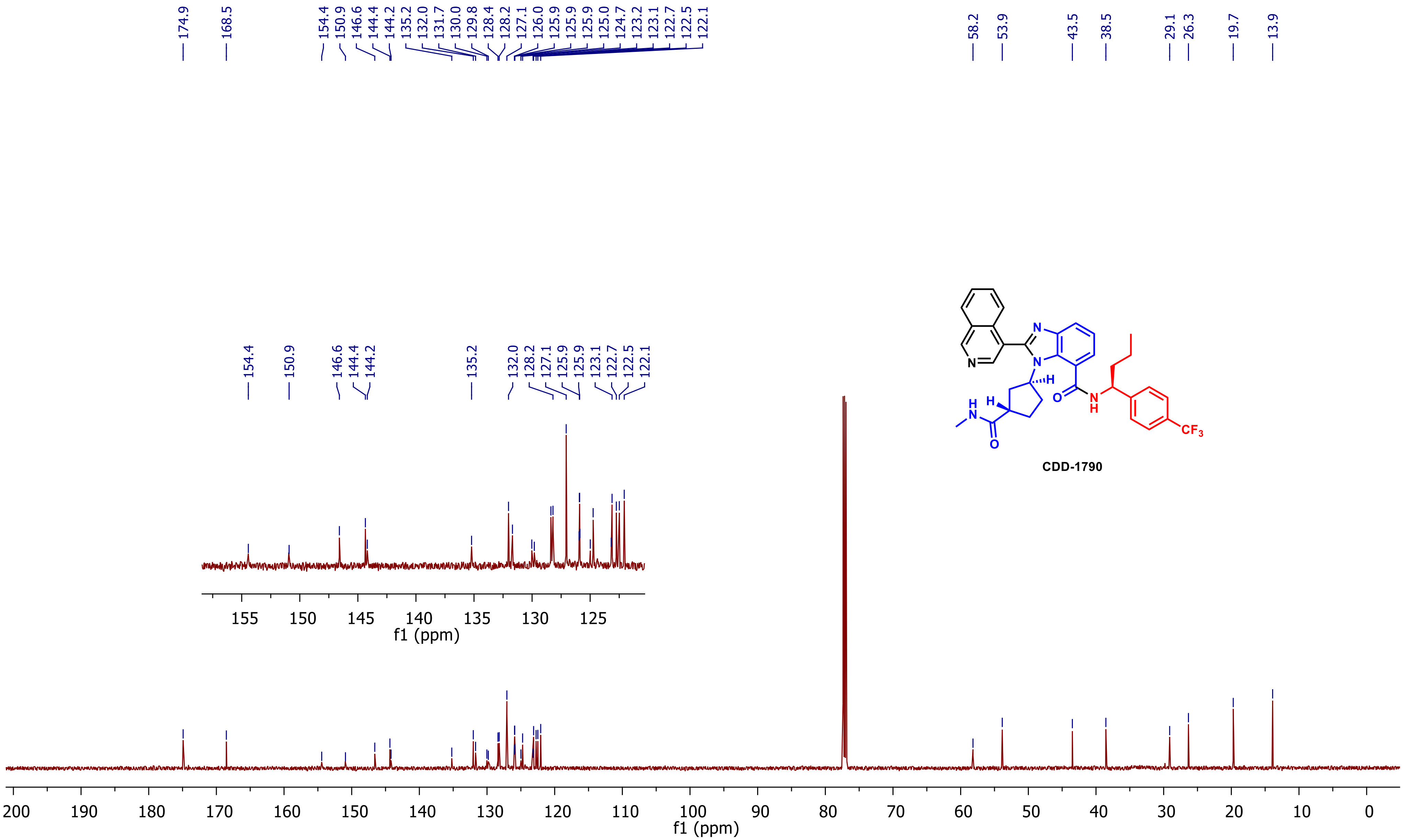

174.9
168.5
154.4
150.9
146.6
144.4
144.2
135.2
132.0
131.7
130.0
129.8
128.4
128.2
127.1
126.0
125.9
125.9
125.9
125.0
124.7
123.2
123.1
122.7
122.5
122.1
58.2
53.9
43.5
38.5
29.1
26.3
19.7
13.9
CDD-1790
f1 (ppm)

C:\Analytical\Data\JRK\09252020\CDD1790 09/25/20 15:23:07

RT: 0.00 - 7.00 SM: 7B

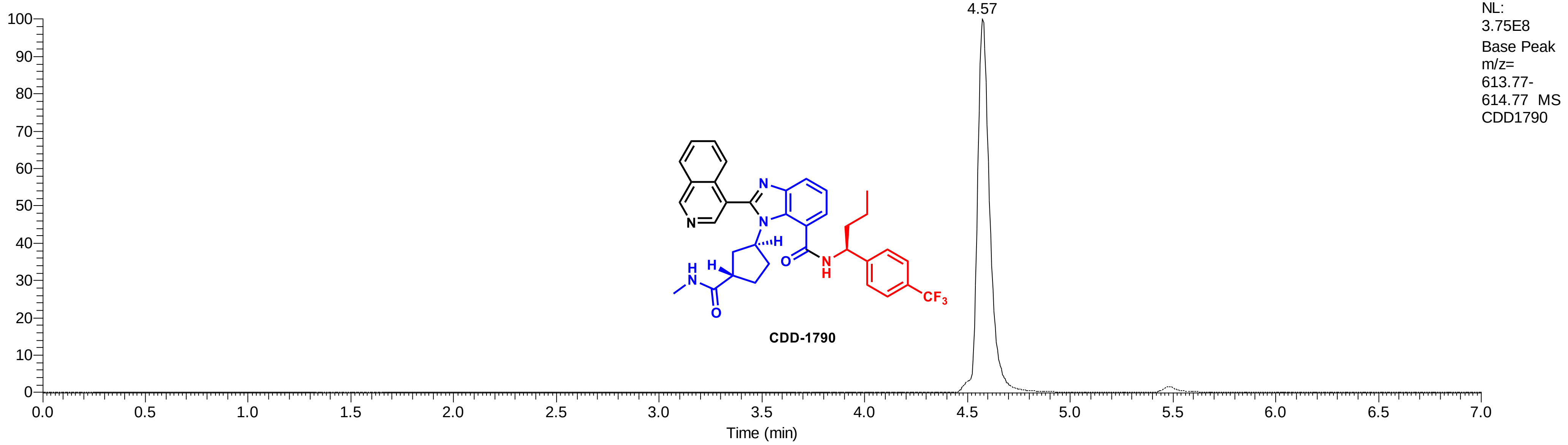

CDD1790 #586 RT: 4.57 AV: 1 NL: 4.30E8
T: FTMS + p ESI Full ms [100.0000-1000.0000]

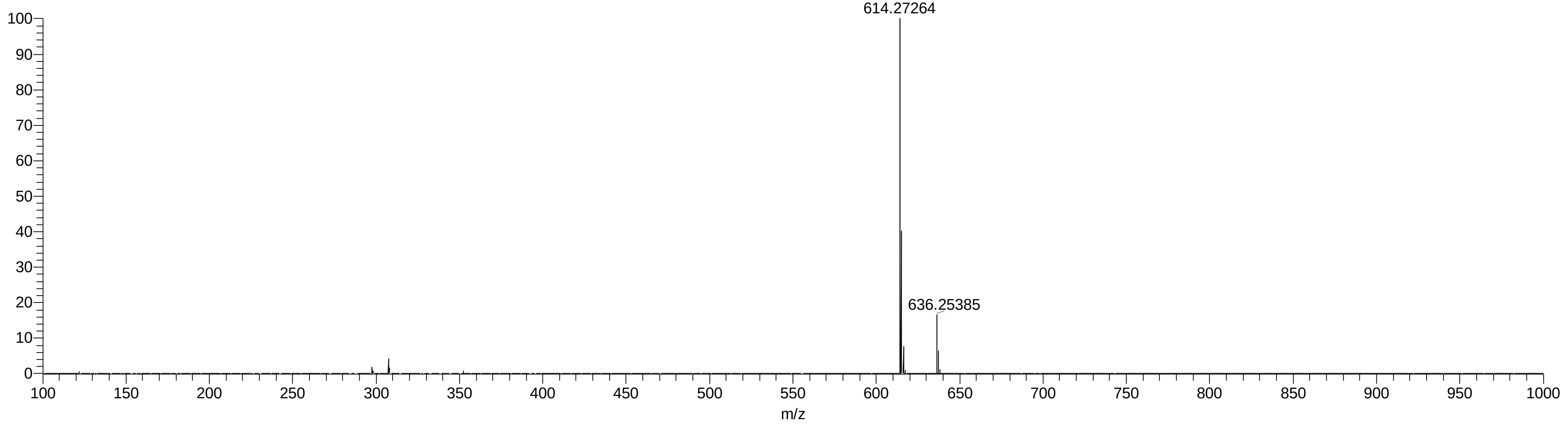

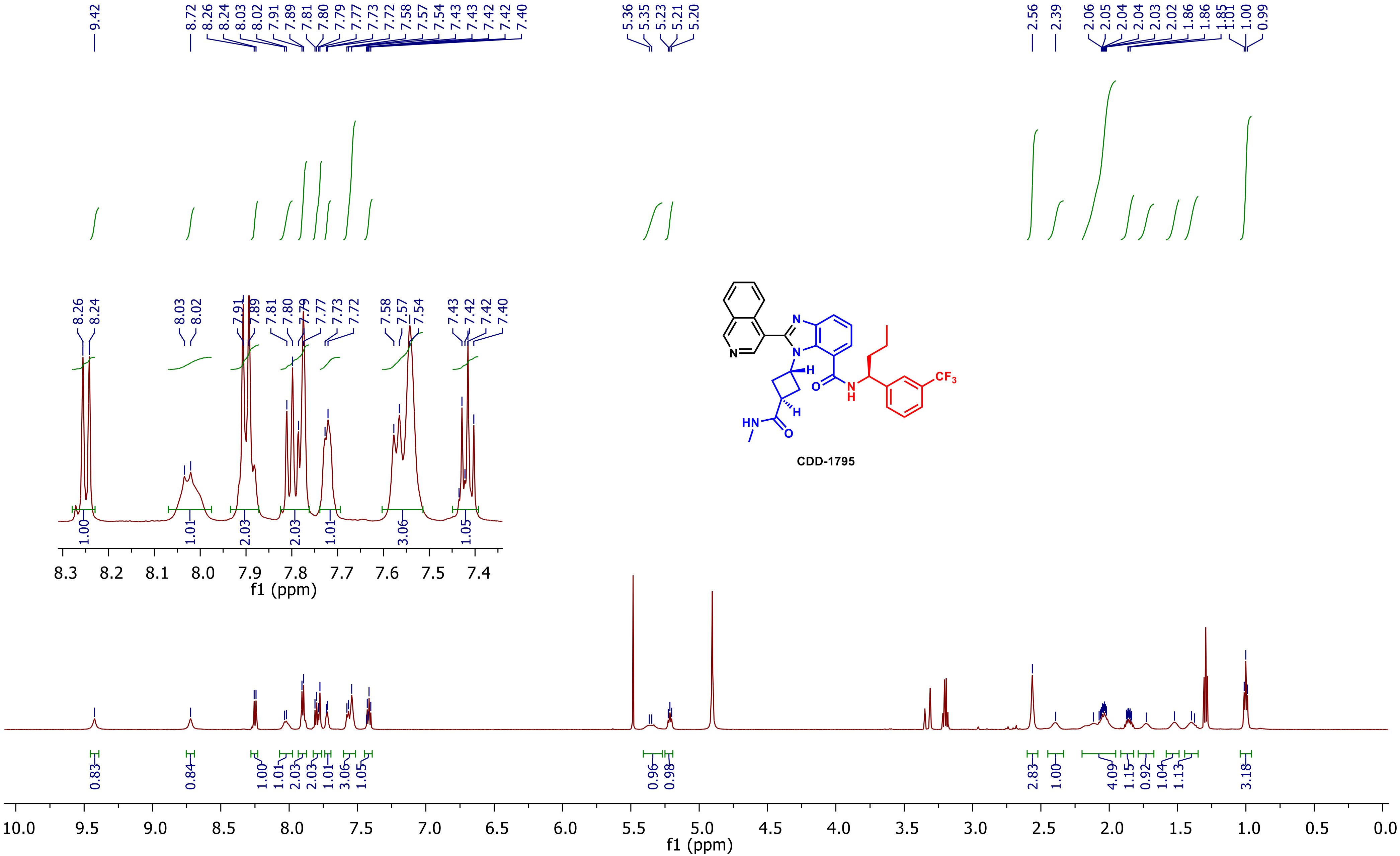
9.42
8.72 8.26 8.24 8.03 8.02 7.91 7.89 7.81 7.80 7.79 7.77 7.73 7.72 7.58 7.57 7.54 7.43 7.43 7.42 7.42 7.40
5.36 5.35 5.23 5.21 5.20
2.56 2.39 2.06 2.05 2.04 2.04 2.03 2.02 1.86 1.86 1.85 1.01 1.00 0.99
8.26 8.24 8.03 8.02 7.91 7.89 7.81 7.80 7.79 7.77 7.73 7.72 7.58 7.57 7.54 7.43 7.42 7.42 7.40
1.00 1.01 2.03 2.03 1.01 3.06 1.05
8.3 8.2 8.1 8.0 7.9 7.8 7.7 7.6 7.5 7.4
f1 (ppm)
0.83 0.84 1.00 1.01 2.03 2.03 1.01 3.06 1.05 0.96 0.98 2.83 1.00 4.09 1.15 0.92 1.04 1.13 3.18
10.0 9.5 9.0 8.5 8.0 7.5 7.0 6.5 6.0 5.5 5.0 4.5 4.0 3.5 3.0 2.5 2.0 1.5 1.0 0.5 0.0
f1 (ppm)
N
N
N
H
H
O
N
H
CF3
HN
O

CDD-1795

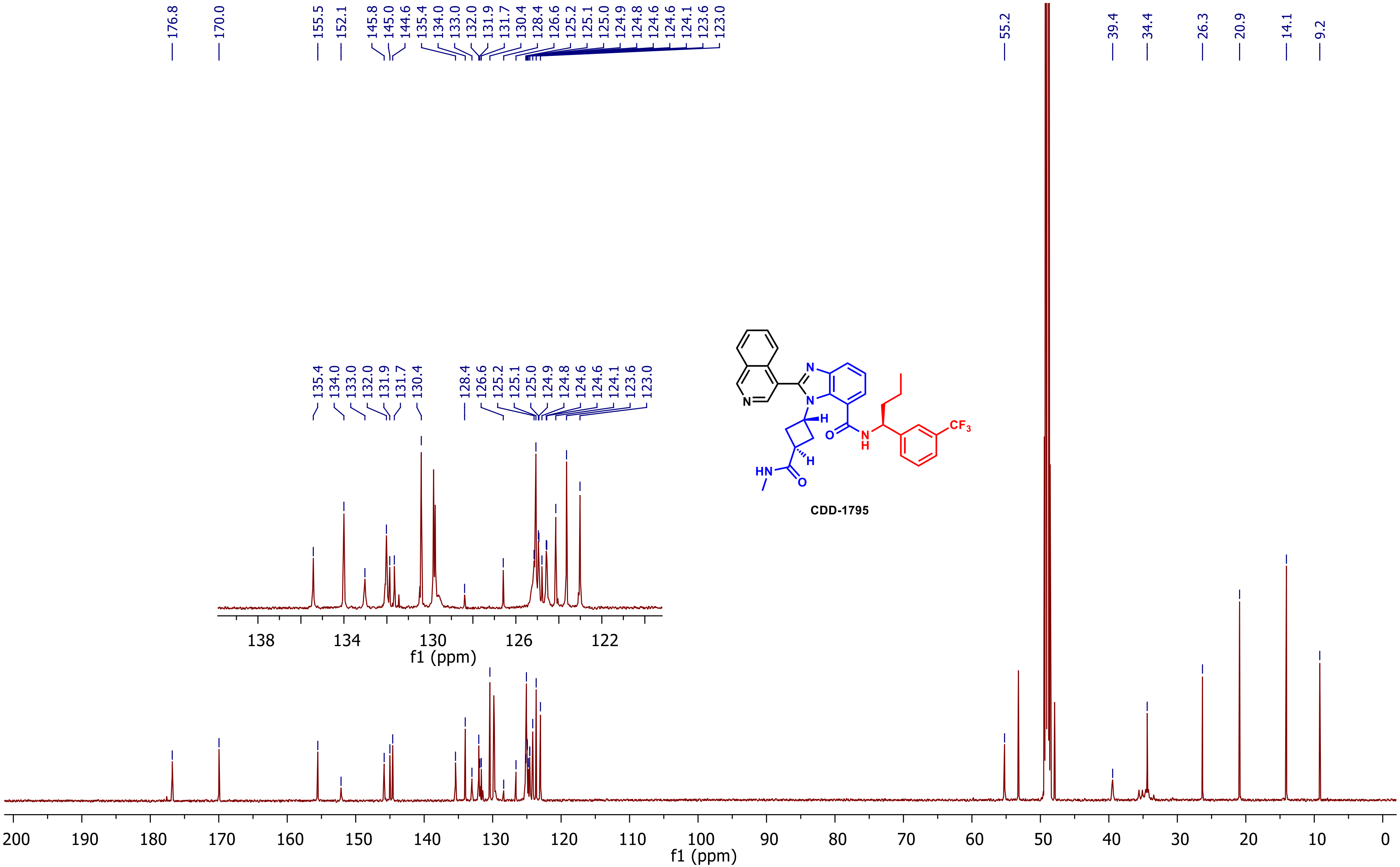
176.8
170.0
155.5
152.1
145.8
145.0
144.6
135.4
134.0
133.0
132.0
131.9
131.7
130.4
128.4
126.6
125.2
125.1
125.0
124.9
124.8
124.6
124.6
124.1
123.6
123.0
55.2
39.4
34.4
26.3
20.9
14.1
9.2
CDD-1795
f1 (ppm)

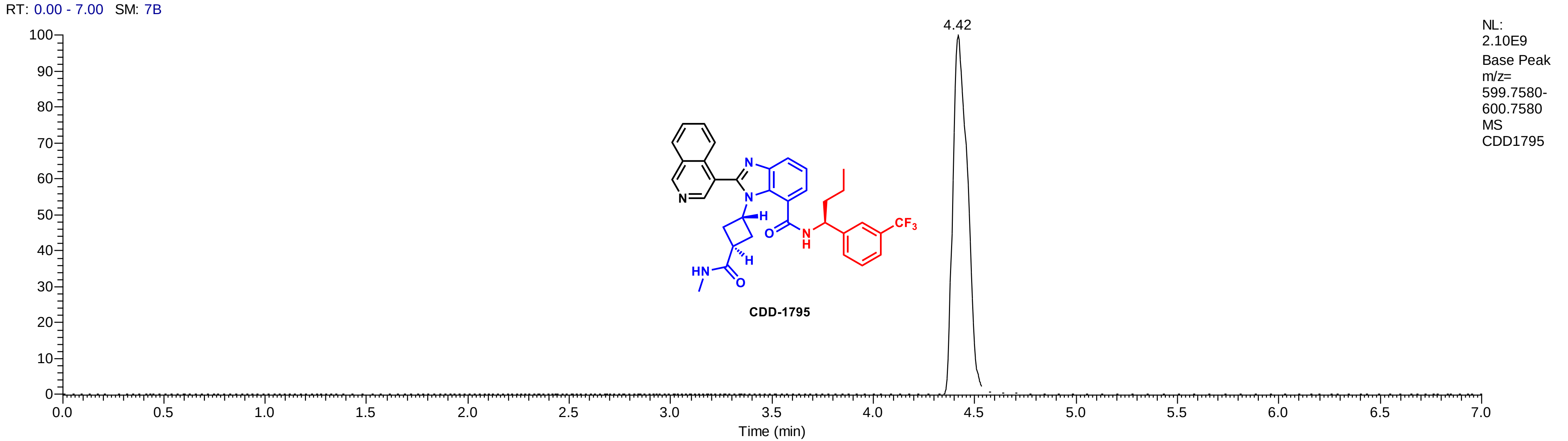
RT: 0.00 - 7.00 SM: 7B
4.42
NL: 2.10E9
Base Peak m/z= 599.7580-600.7580
MS CDD1795
N
N
N
H
H
O
N
H
CF3
HN
O
CDD-1795
Time (min)

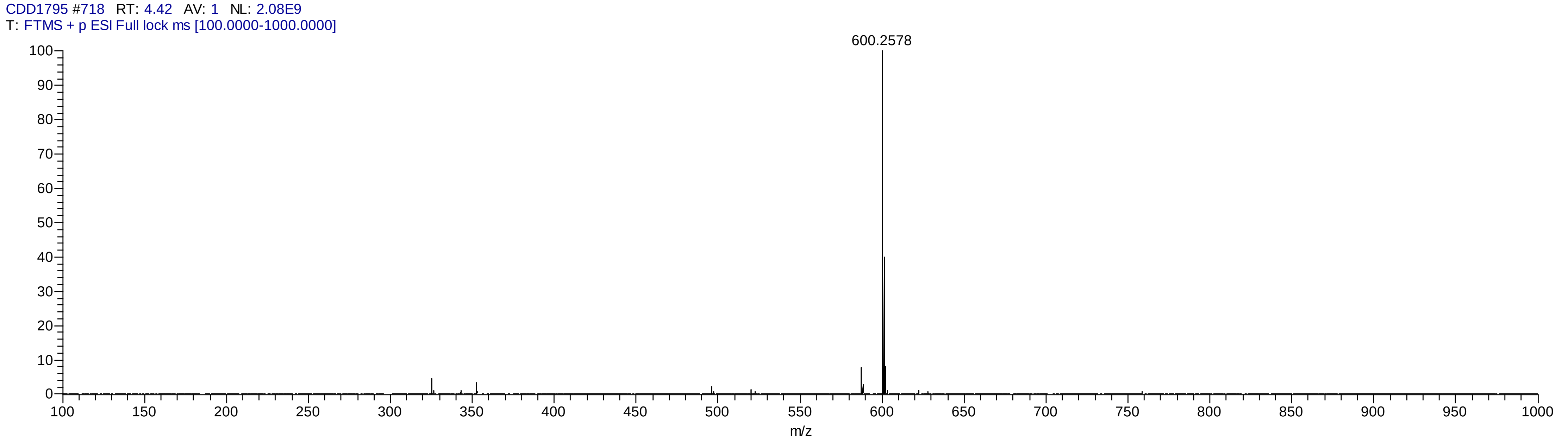
CDD1795 #718 RT: 4.42 AV: 1 NL: 2.08E9
T: FTMS + p ESI Full lock ms [100.0000-1000.0000]
600.2578
m/z

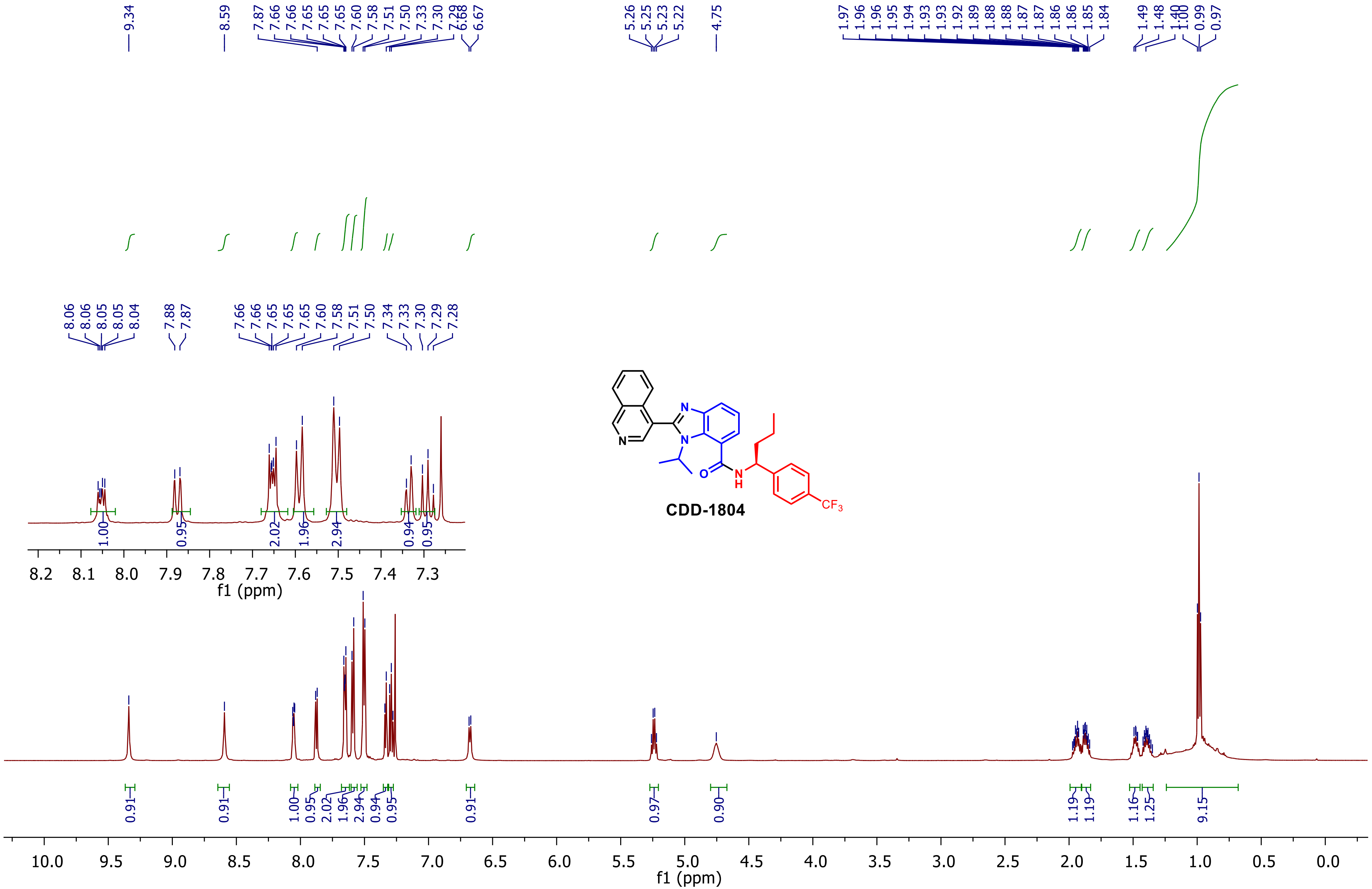
CDD-1804
f1 (ppm)

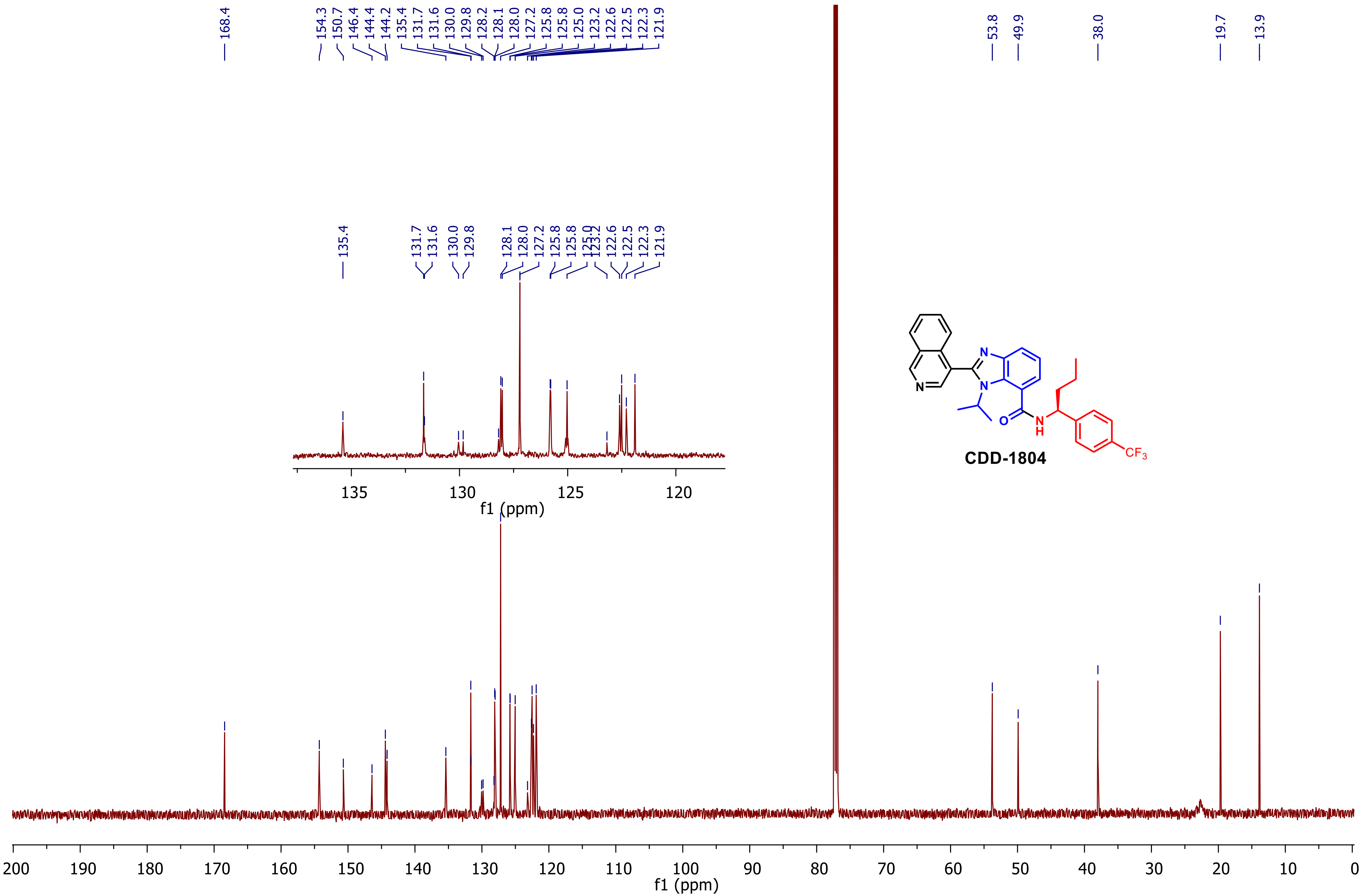
168.4
154.3
150.7
146.4
144.4
144.2
135.4
131.7
131.6
130.0
129.8
128.2
128.1
128.0
127.2
125.8
125.8
125.0
123.2
122.6
122.5
122.3
121.9
53.8
49.9
38.0
19.7
13.9
f1 (ppm)
N
O
H
CF3
CDD-1804

C:\Analytical\...\04012021\CDD1804 04/01/21 16:10:08

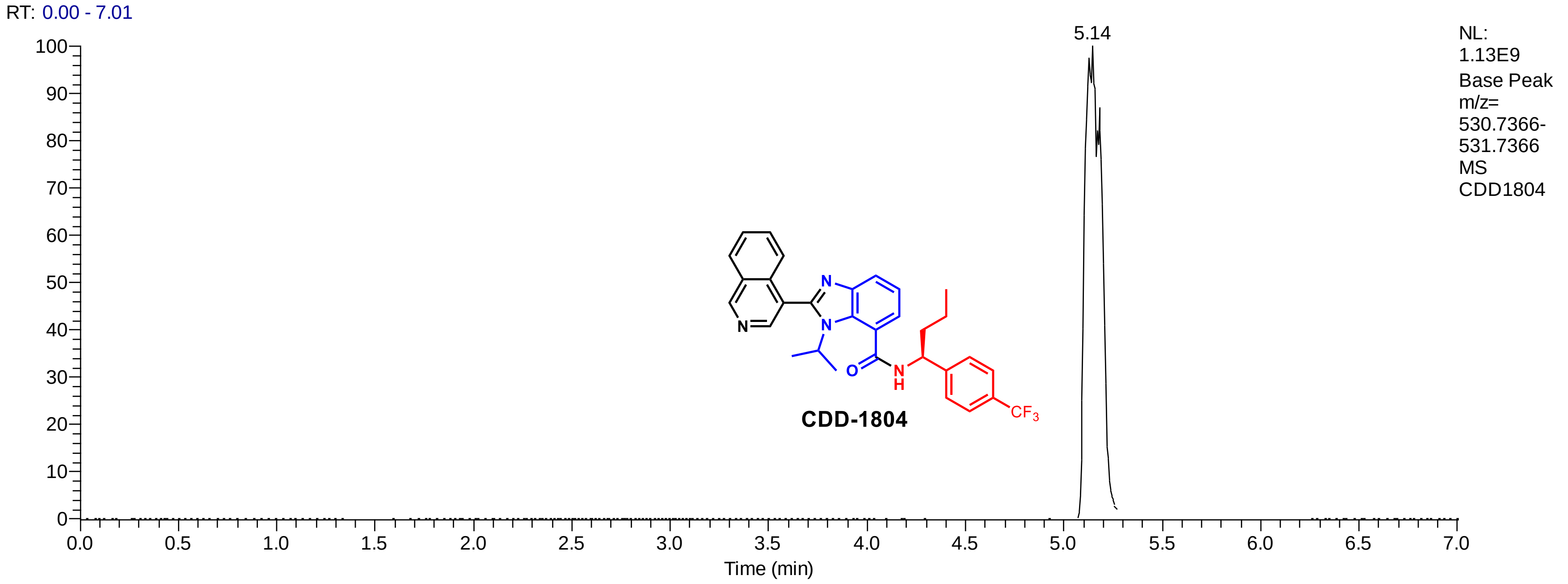

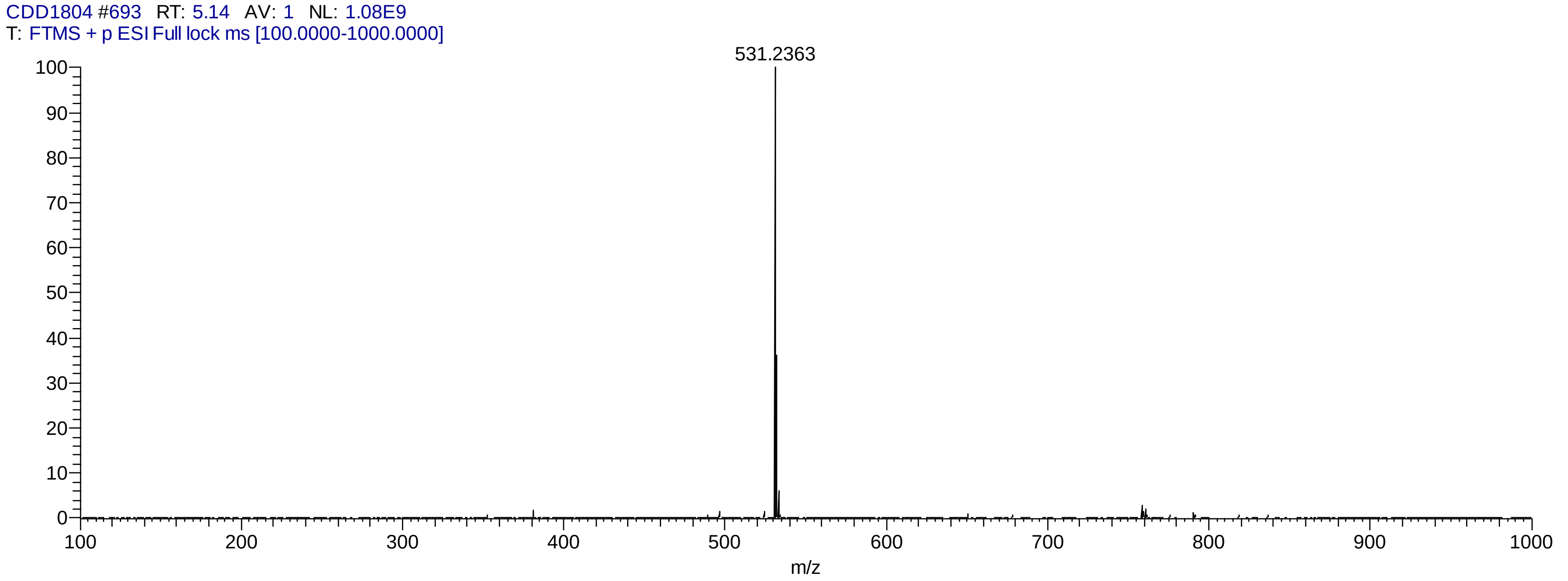

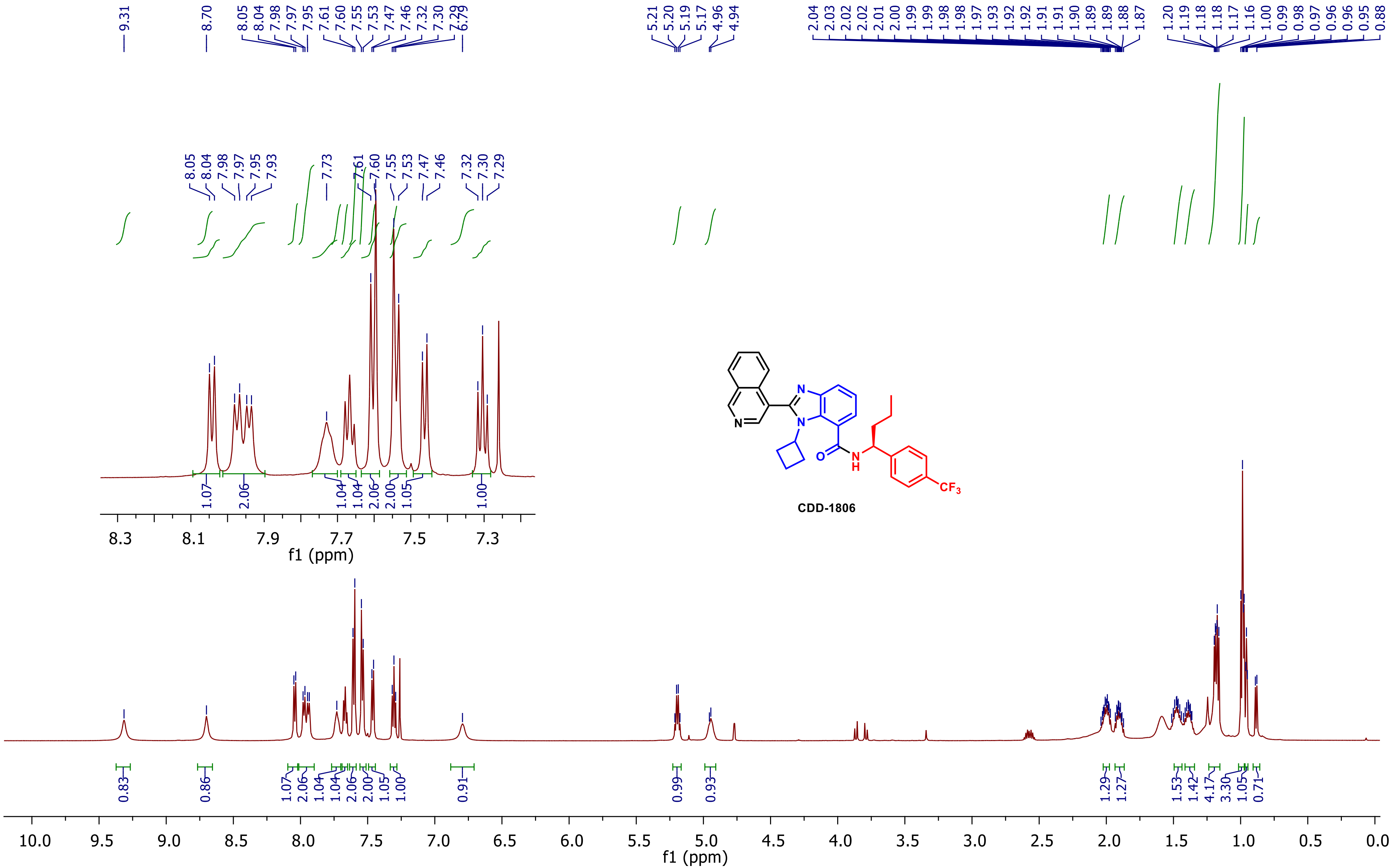
CDD-1806
f1 (ppm)

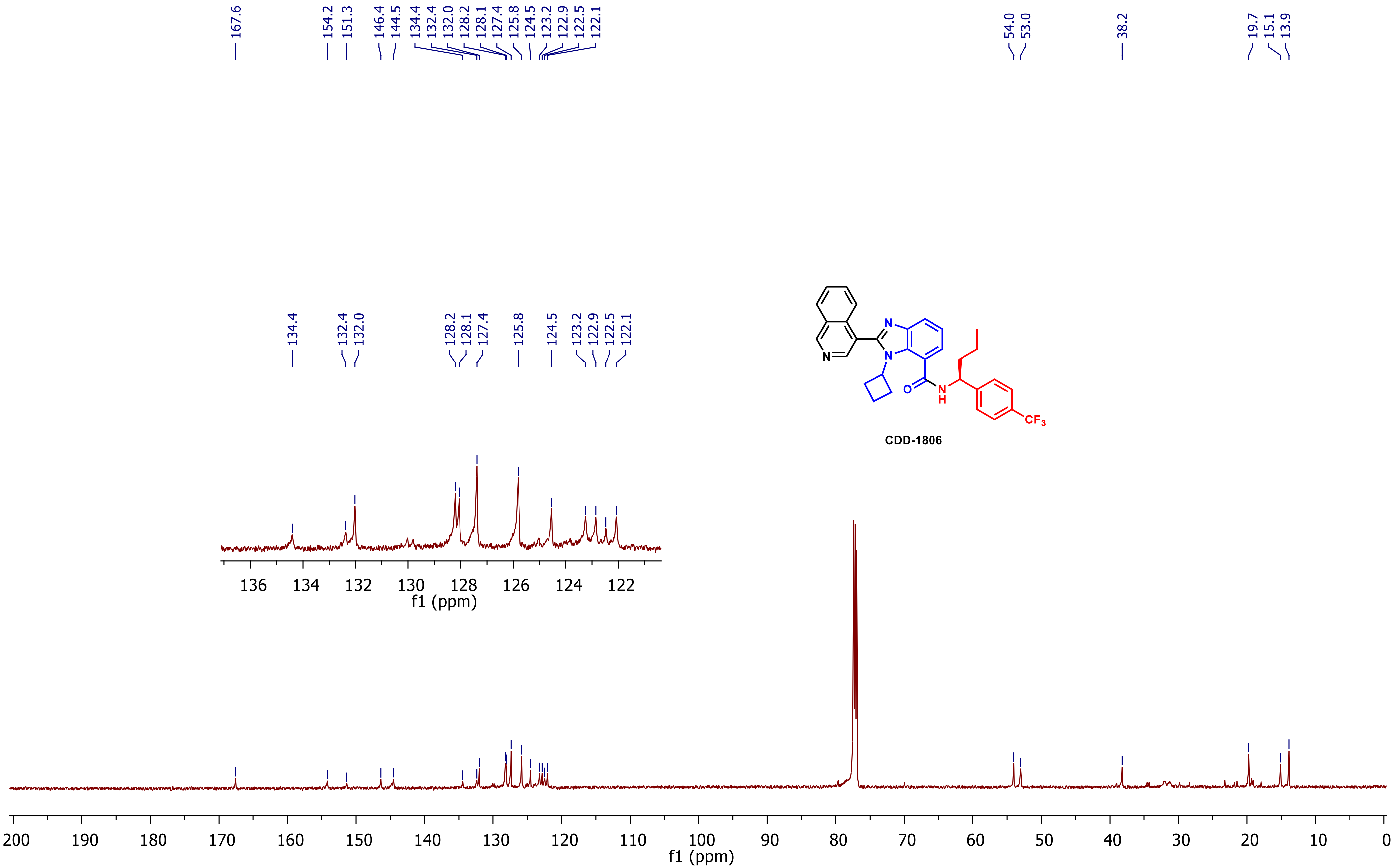
167.6
154.2
151.3
146.4
144.5
134.4
132.4
132.0
128.2
128.1
127.4
125.8
124.5
123.2
122.9
122.5
122.1
54.0
53.0
38.2
19.7
15.1
13.9
N
N
N
O
N
H
CF3
CDD-1806
136 134 132 130 128 126 124 122
f1 (ppm)
200 190 180 170 160 150 140 130 120 110 100 90 80 70 60 50 40 30 20 10 0
f1 (ppm)

RT: 0.00 - 7.01 SM: 7B

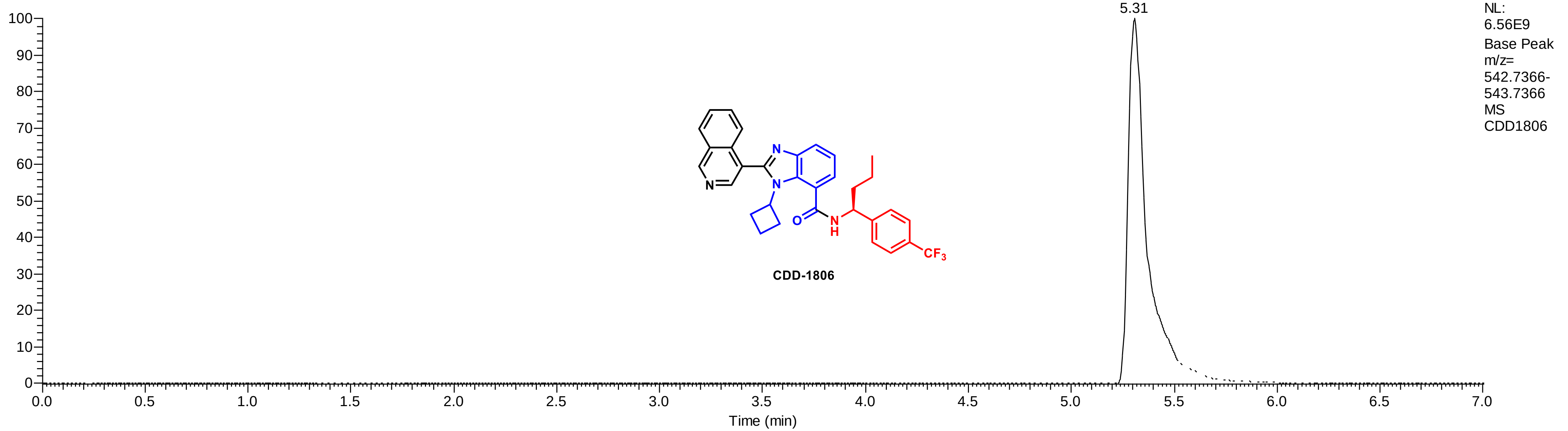

CDD1806 #725 RT: 5.31 AV: 1 NL: 6.78E9
T: FTMS + p ESI Full ms [100.0000-1000.0000]

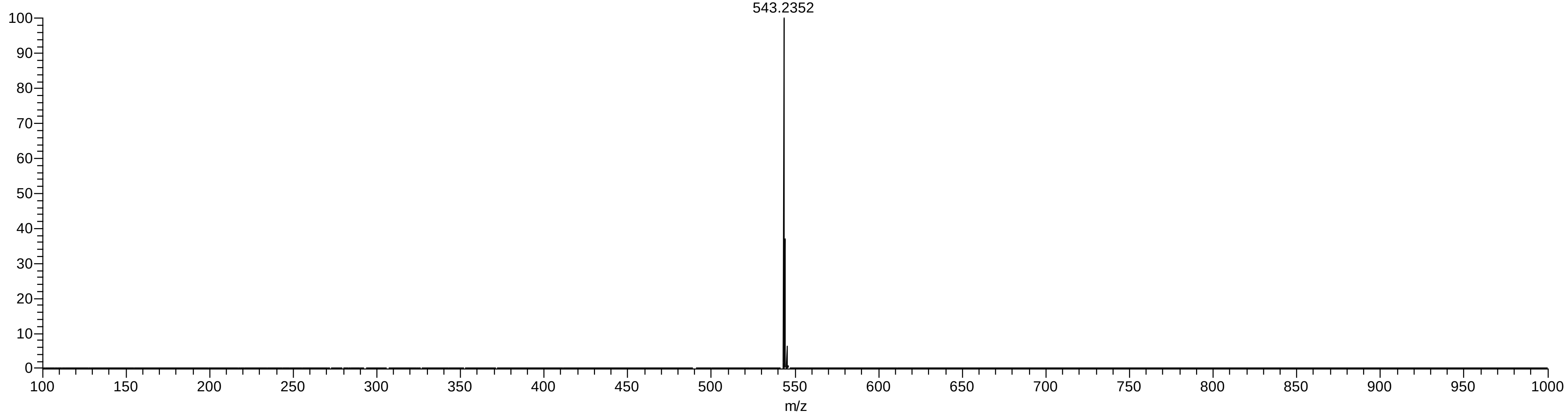

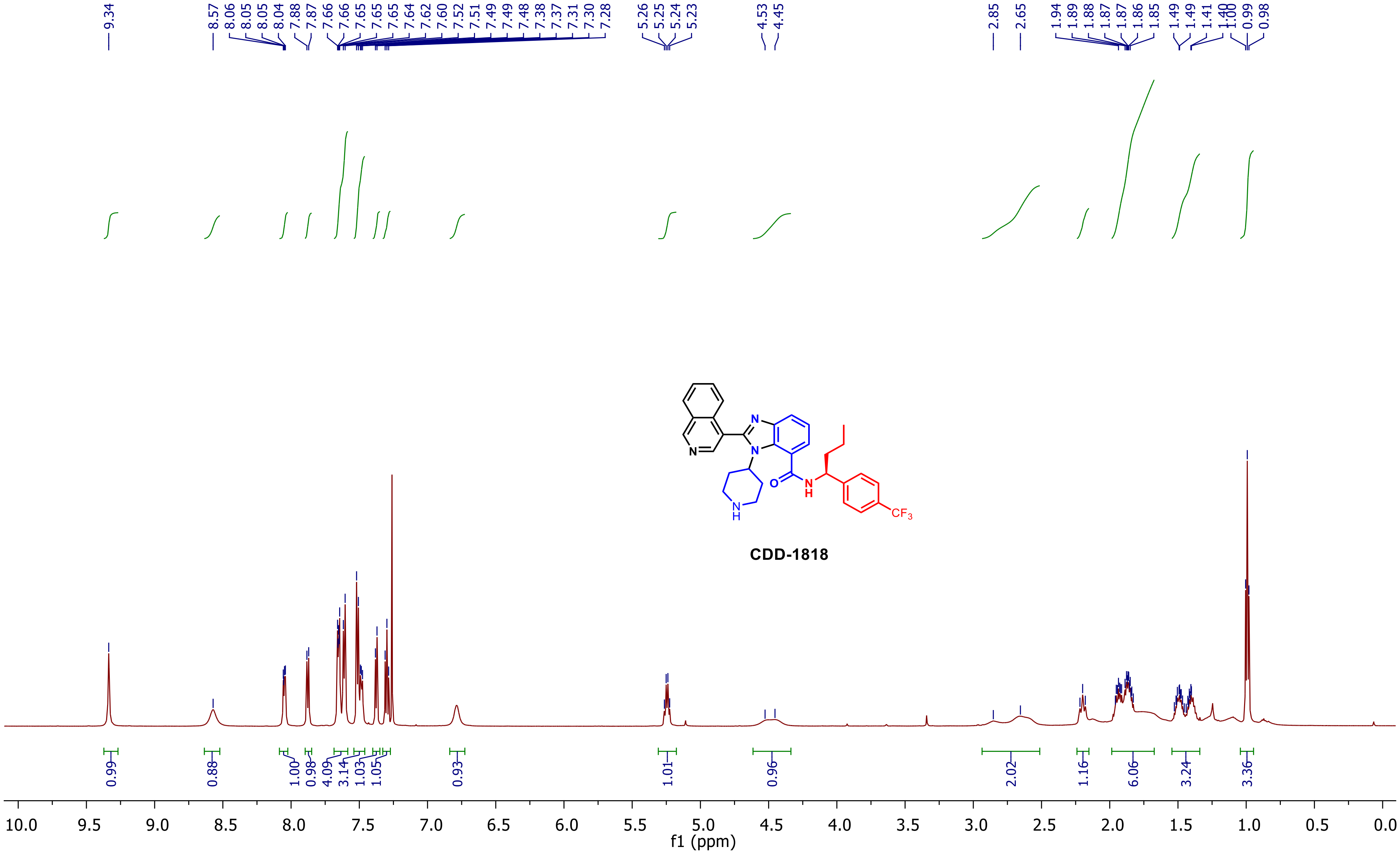
9.34
8.57 8.06 8.05 8.05 8.04 7.88 7.87 7.66 7.66 7.65 7.65 7.64 7.62 7.60 7.52 7.51 7.49 7.49 7.48 7.38 7.37 7.31 7.30 7.28
5.26 5.25 5.24 5.23
4.53 4.45
2.85 2.65
1.94 1.89 1.88 1.87 1.87 1.86 1.85 1.49 1.49 1.41 1.40 1.00 0.99 0.98
0.99 0.88 1.00 0.98 4.09 3.14 1.03 1.05 0.93 1.01 0.96 2.02 1.16 6.06 3.24 3.36
10.0 9.5 9.0 8.5 8.0 7.5 7.0 6.5 6.0 5.5 5.0 4.5 4.0 3.5 3.0 2.5 2.0 1.5 1.0 0.5 0.0
f1 (ppm)
N
N
N
O
N
H
CF3
N
H

CDD-1818

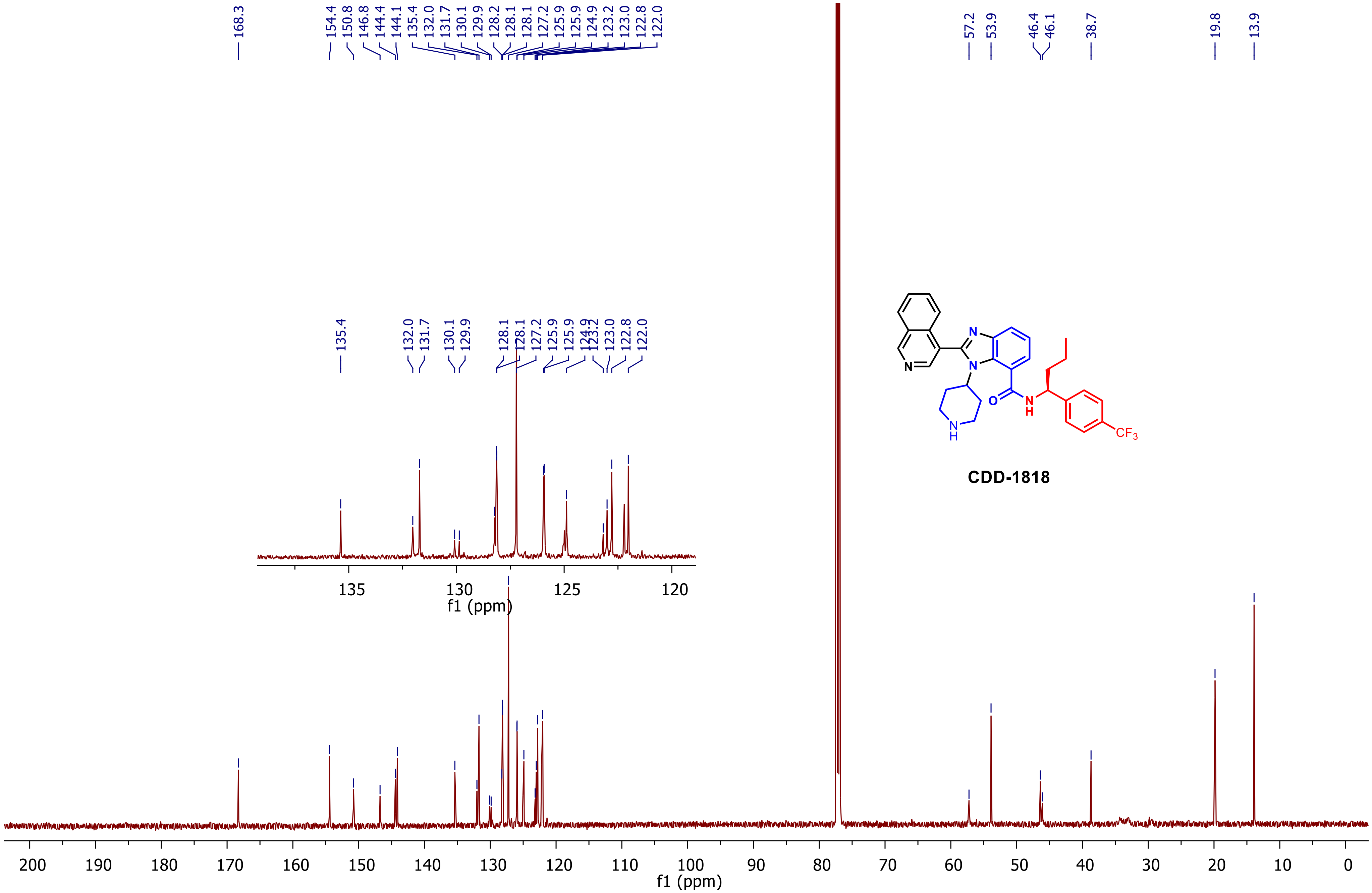
168.3
154.4
150.8
146.8
144.4
144.1
135.4
132.0
131.7
130.1
129.9
128.2
128.1
128.1
127.2
125.9
125.9
124.9
123.2
123.0
122.8
122.0
57.2
53.9
46.4
46.1
38.7
19.8
13.9
f1 (ppm)
CDD-1818

C:\Analytical\...\JRK-HRMS_MPRO\CDD1818 10/19/20 10:23:40

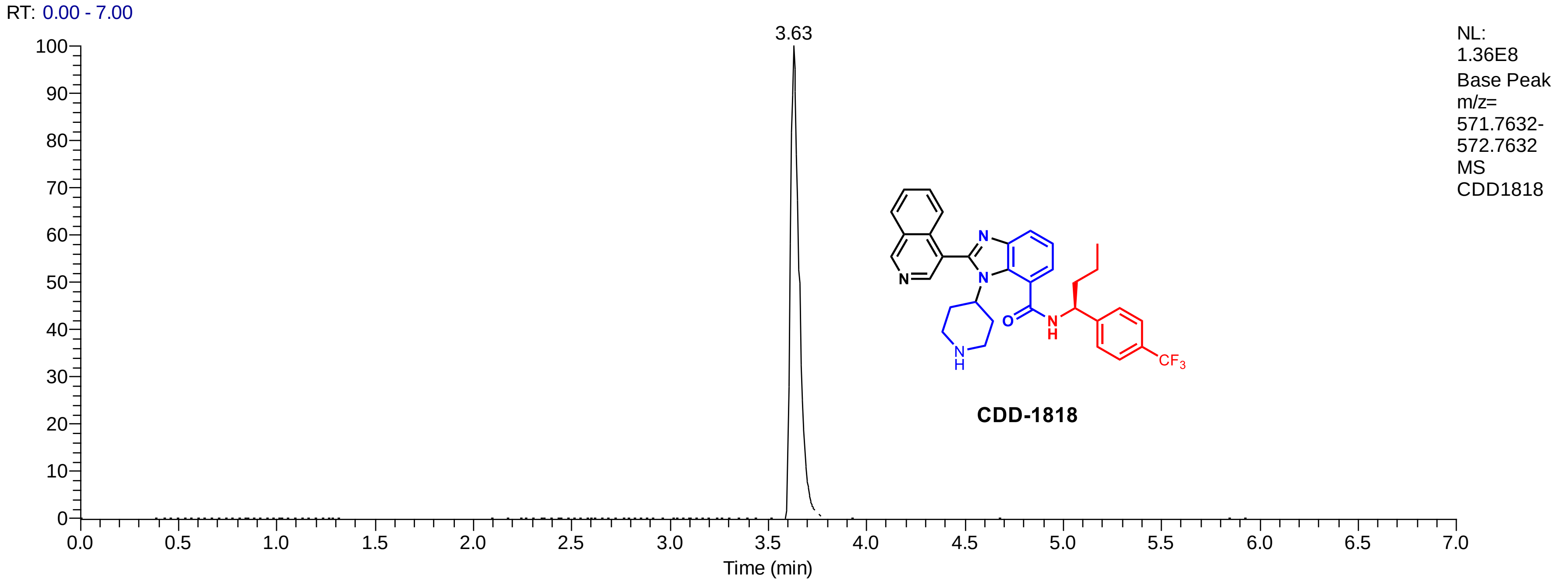

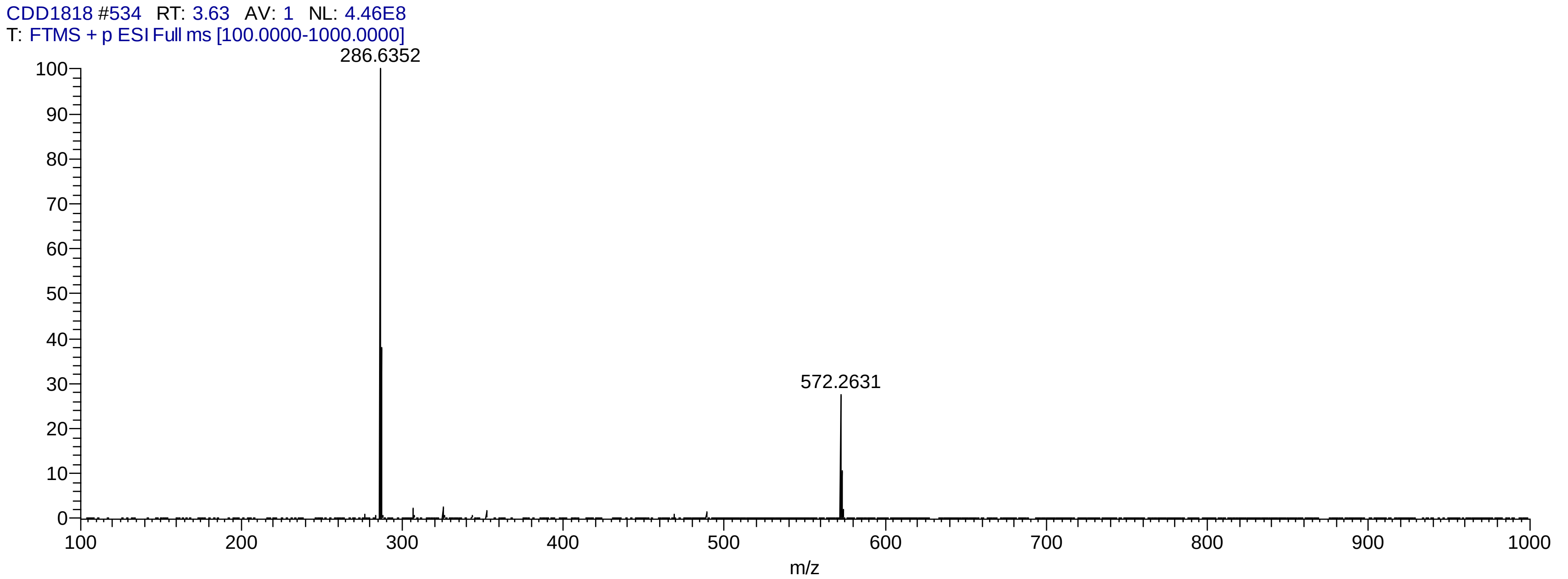

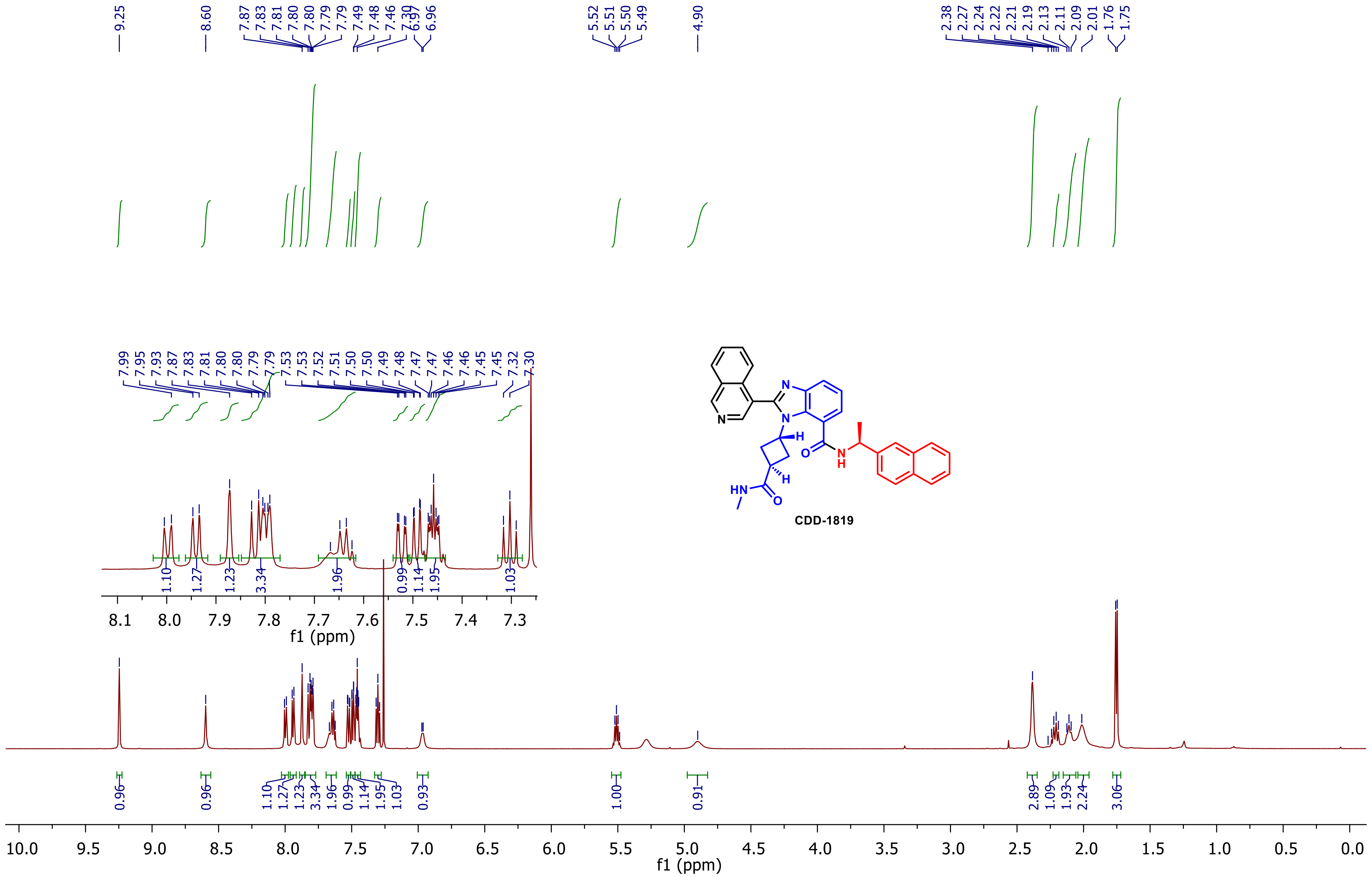
9.25
8.60
7.87 7.83 7.81 7.80 7.80 7.79 7.79 7.49 7.48 7.46 7.30 6.97 6.96
5.52 5.51 5.50 5.49
4.90
2.38 2.27 2.24 2.22 2.21 2.19 2.13 2.11 2.09 2.01 1.76 1.75
7.99 7.95 7.93 7.87 7.83 7.81 7.80 7.80 7.79 7.79 7.53 7.53 7.52 7.51 7.50 7.50 7.49 7.48 7.47 7.47 7.46 7.46 7.45 7.45 7.32 7.30
1.10 1.27 1.23 3.34 1.96 0.99 1.14 1.95 1.03
8.1 8.0 7.9 7.8 7.7 7.6 7.5 7.4 7.3
f1 (ppm)
N
N
N
H
H
O
N
H
HN
O
CDD-1819
0.96 0.96 1.10 1.27 1.23 3.34 1.96 0.99 1.14 1.95 1.03 0.93 1.00 0.91 2.89 1.09 1.93 2.24 3.06
10.0 9.5 9.0 8.5 8.0 7.5 7.0 6.5 6.0 5.5 5.0 4.5 4.0 3.5 3.0 2.5 2.0 1.5 1.0 0.5 0.0
f1 (ppm)

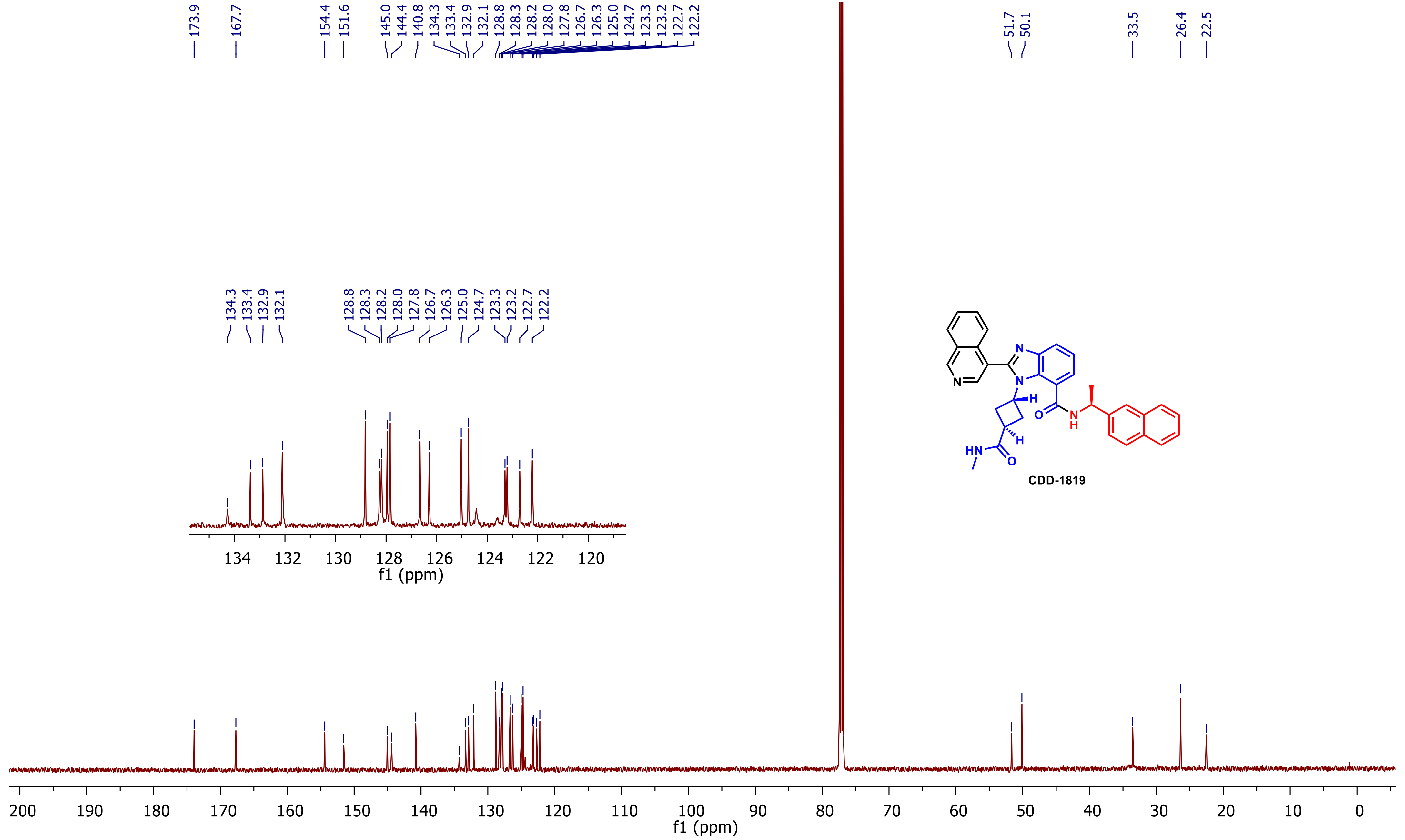
173.9
167.7
154.4
151.6
145.0
144.4
140.8
134.3
133.4
132.9
132.1
128.8
128.3
128.2
128.0
127.8
126.7
126.3
125.0
124.7
123.3
123.2
122.7
122.2
51.7
50.1
33.5
26.4
22.5
f1 (ppm)
N
N
N
H
H
O
N
H
HN
O
CDD-1819

C:\Analytical\...\JRK-HRMS_MPRO\CDD1819 10/19/20 10:31:17

RT: 0.00 - 7.00

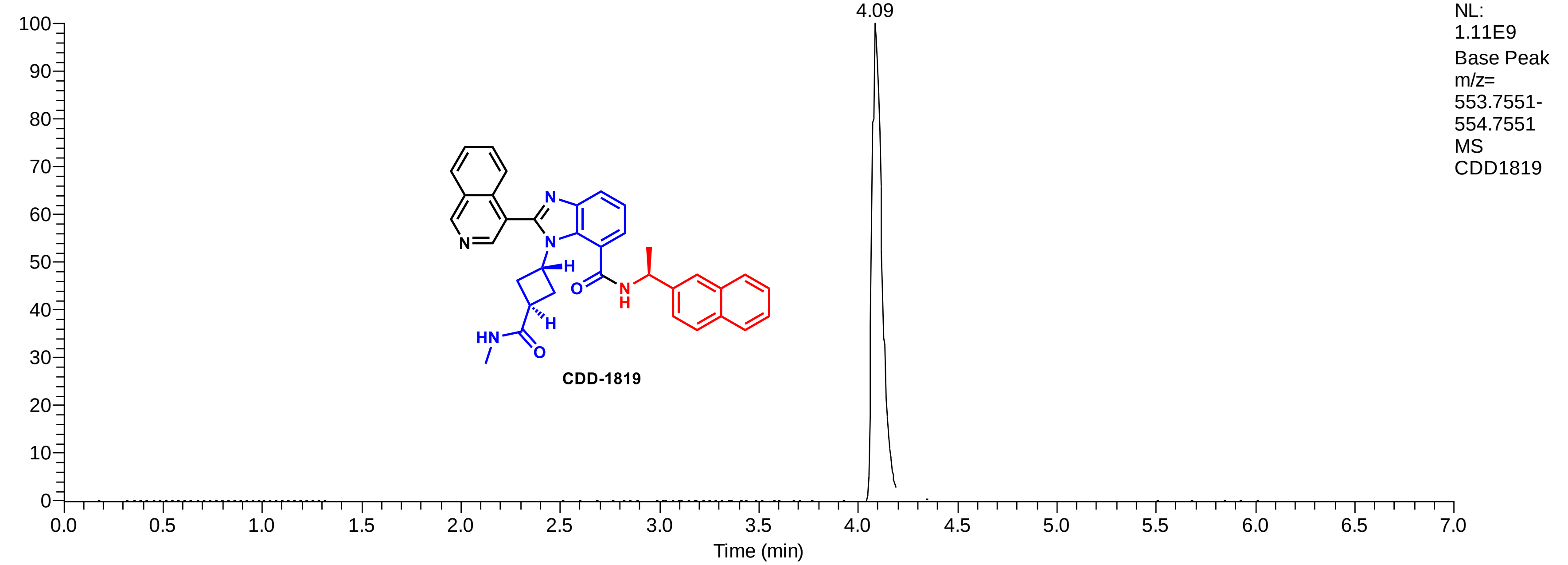

CDD1819 #619 RT: 4.09 AV: 1 NL: 1.10E9
T: FTMS + p ESI Full ms [100.0000-1000.0000]

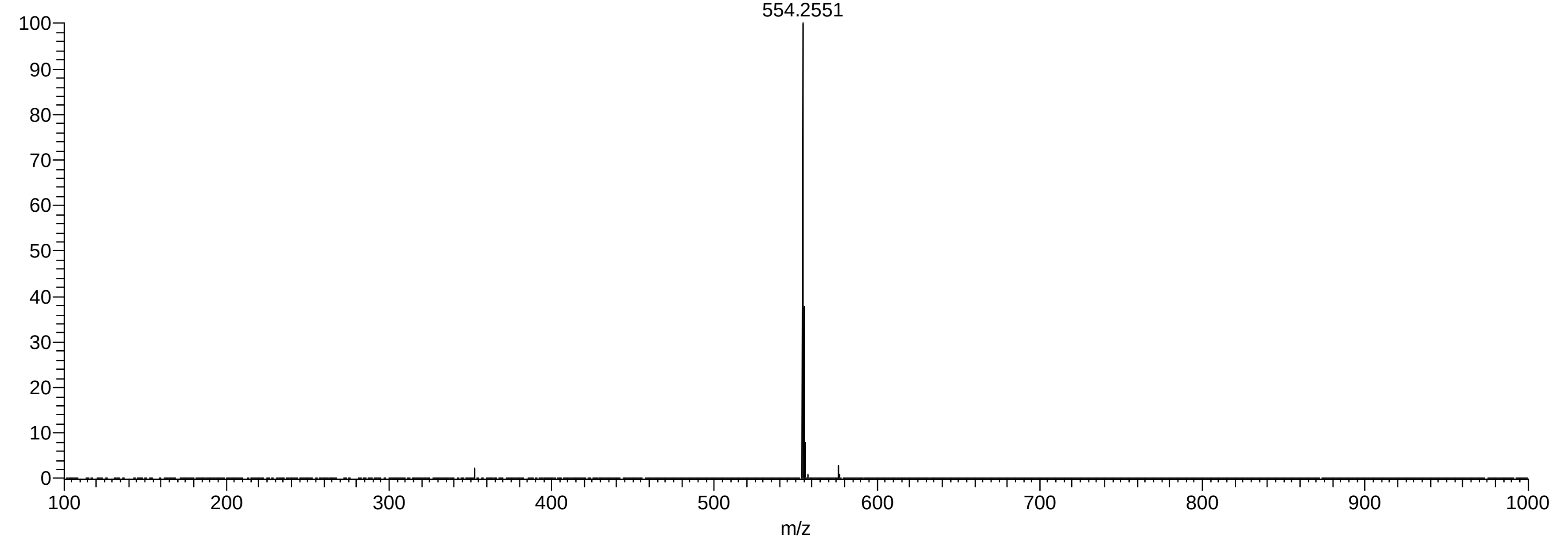

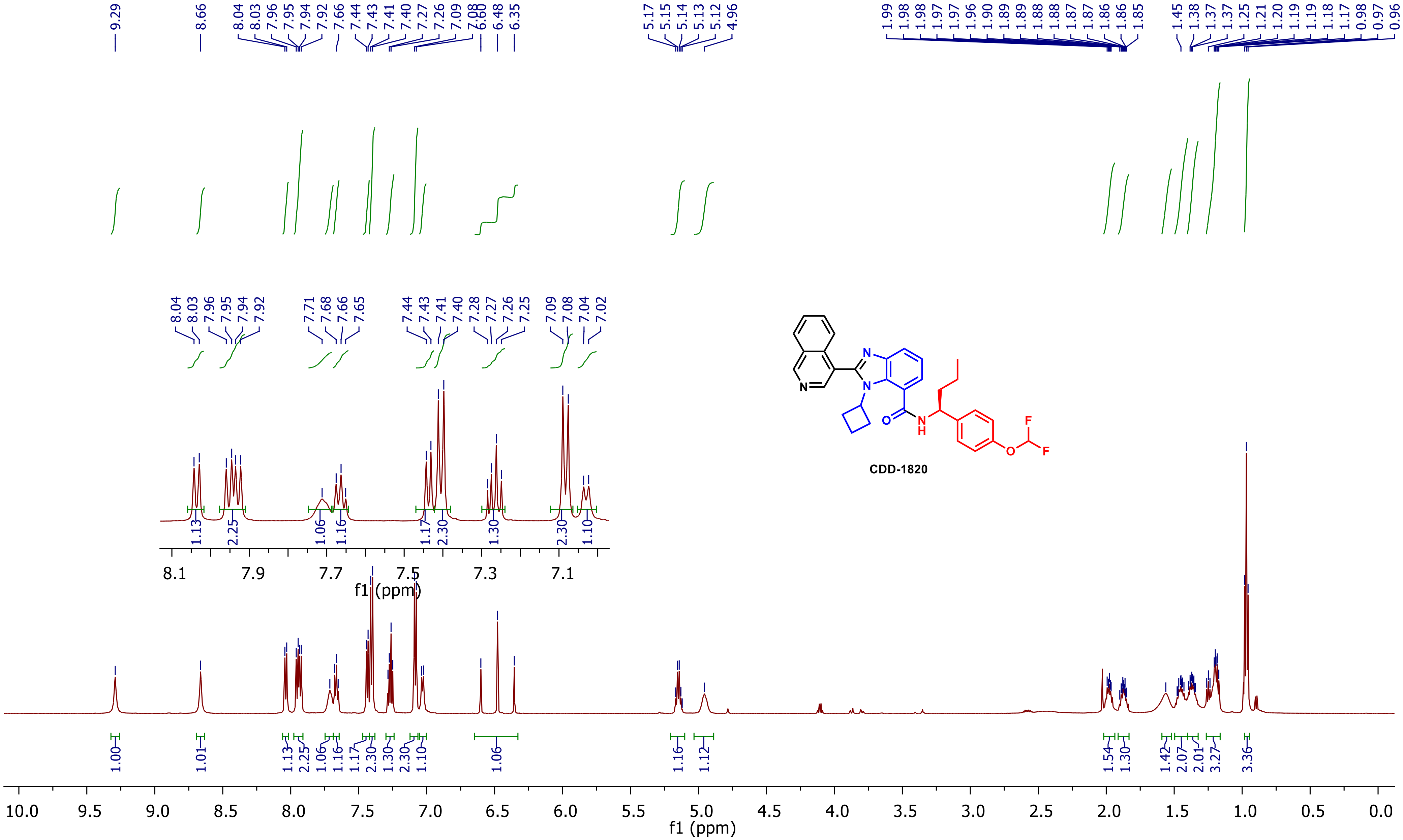
CDD-1820
N
N
N
O
H
F
F
O
f1 (ppm)
f1 (ppm)

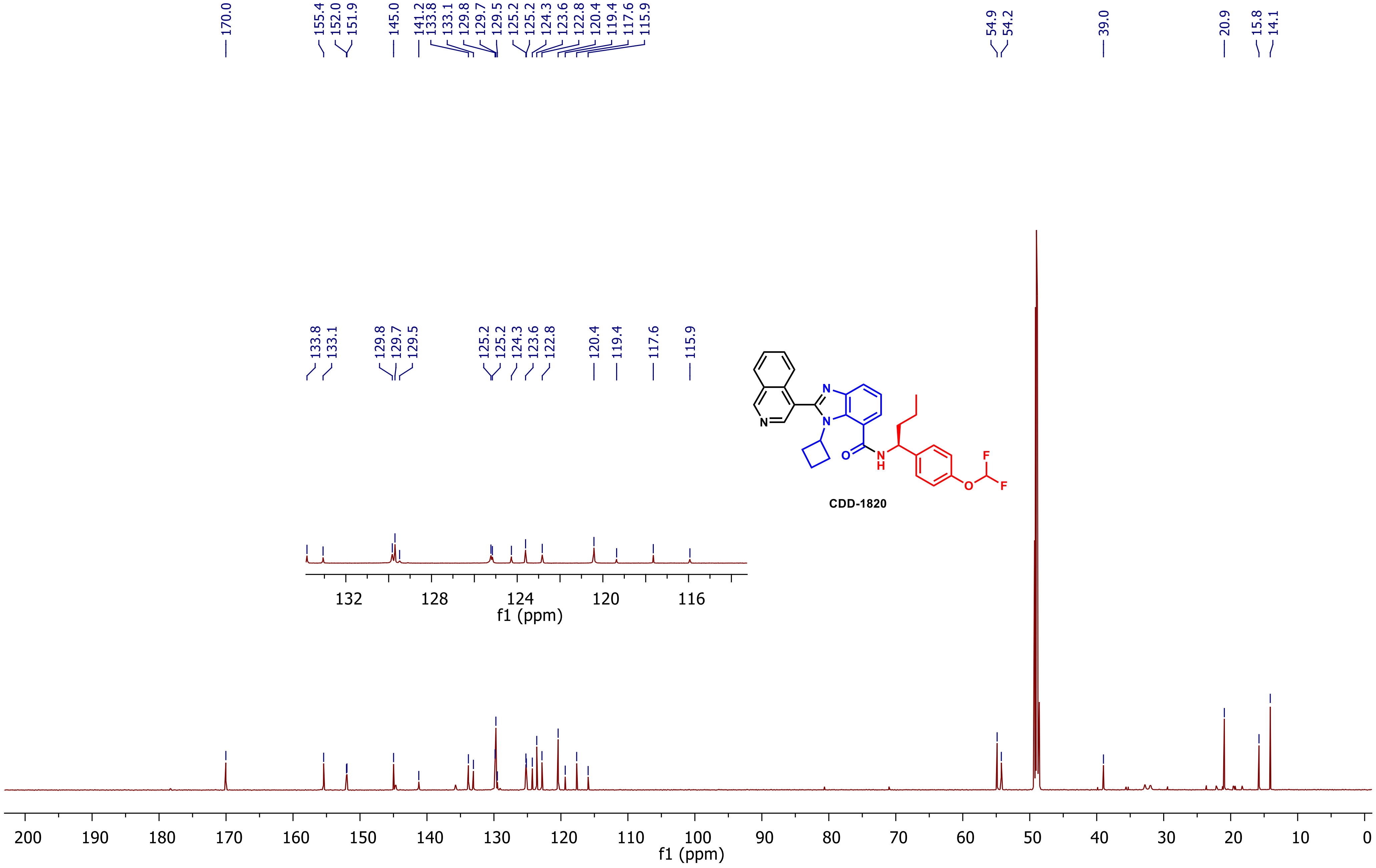
170.0
155.4
152.0
151.9
145.0
141.2
133.8
133.1
129.8
129.7
129.5
125.2
125.2
124.3
123.6
122.8
120.4
119.4
117.6
115.9
54.9
54.2
39.0
20.9
15.8
14.1
CDD-1820
f1 (ppm)
f1 (ppm)

RT: 0.00 - 7.00 SM: 7B

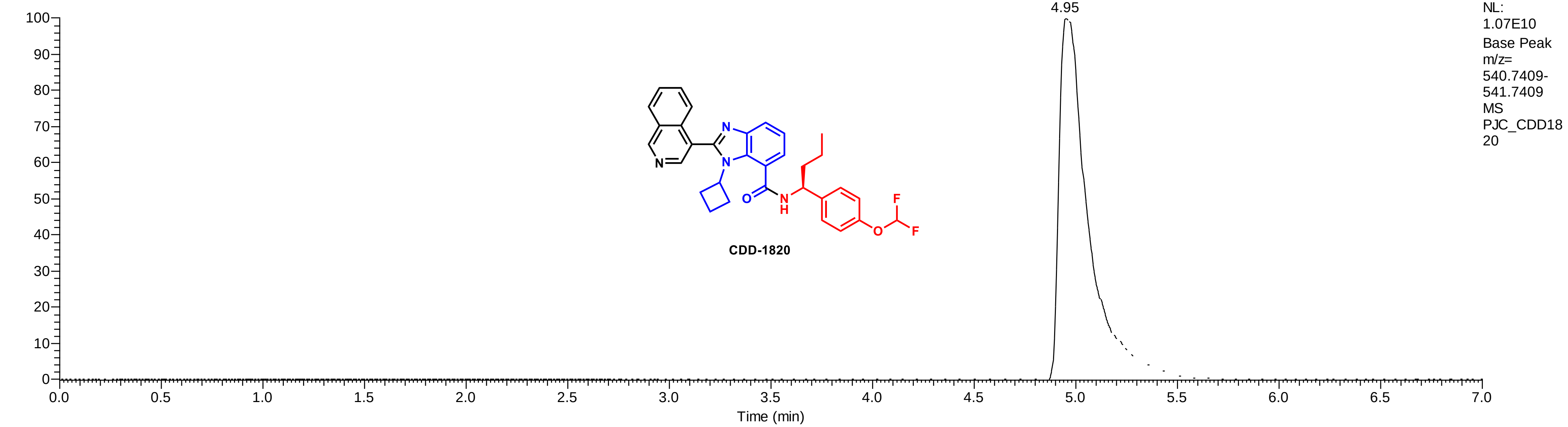

PJC_CDD1820 #742 RT: 4.95 AV: 1 NL: 1.02E10
T: FTMS + p ESI Full ms [100.0000-1000.0000]

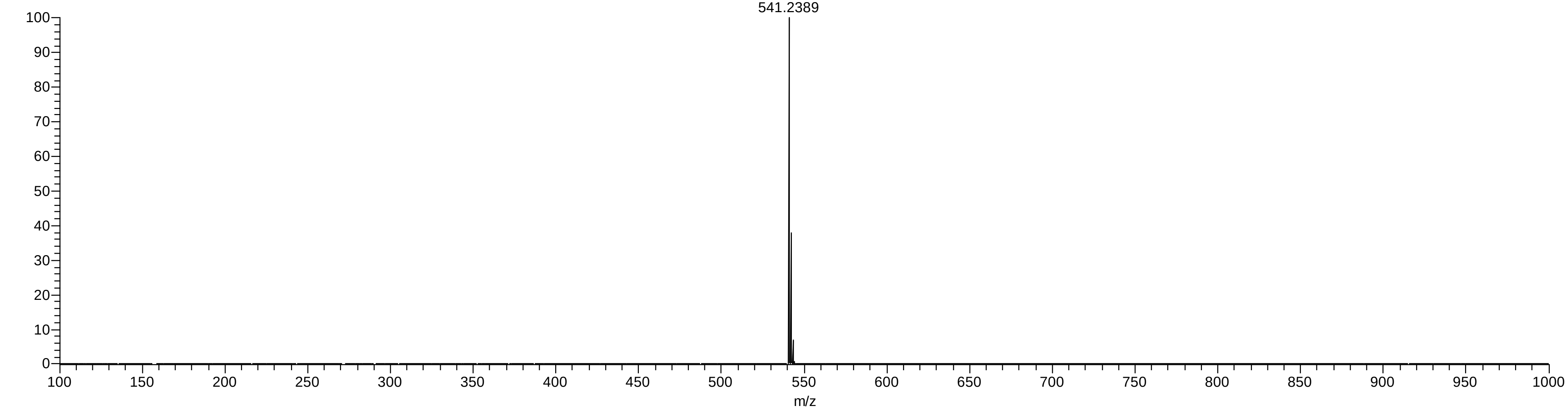

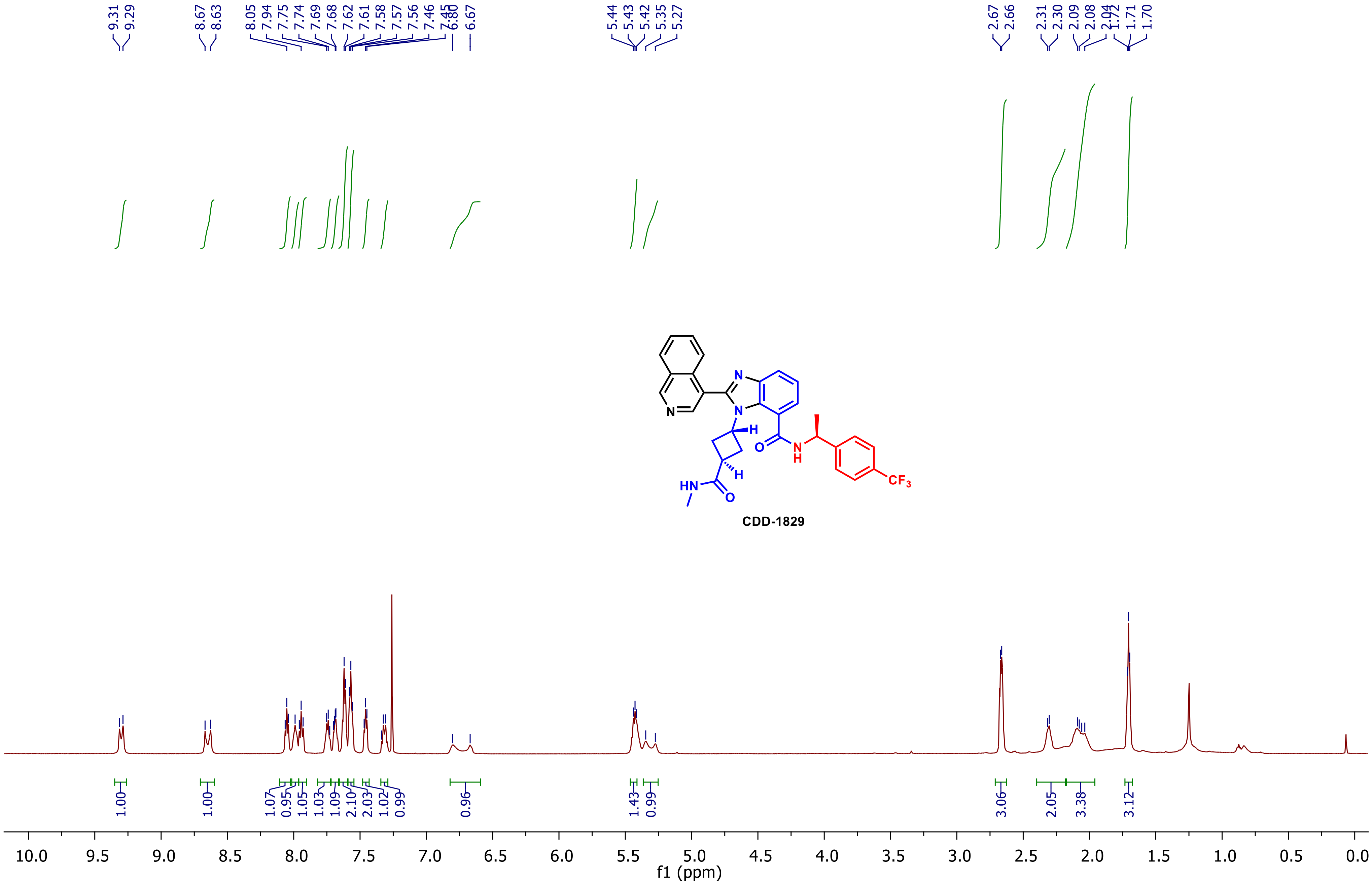

CDD-1829

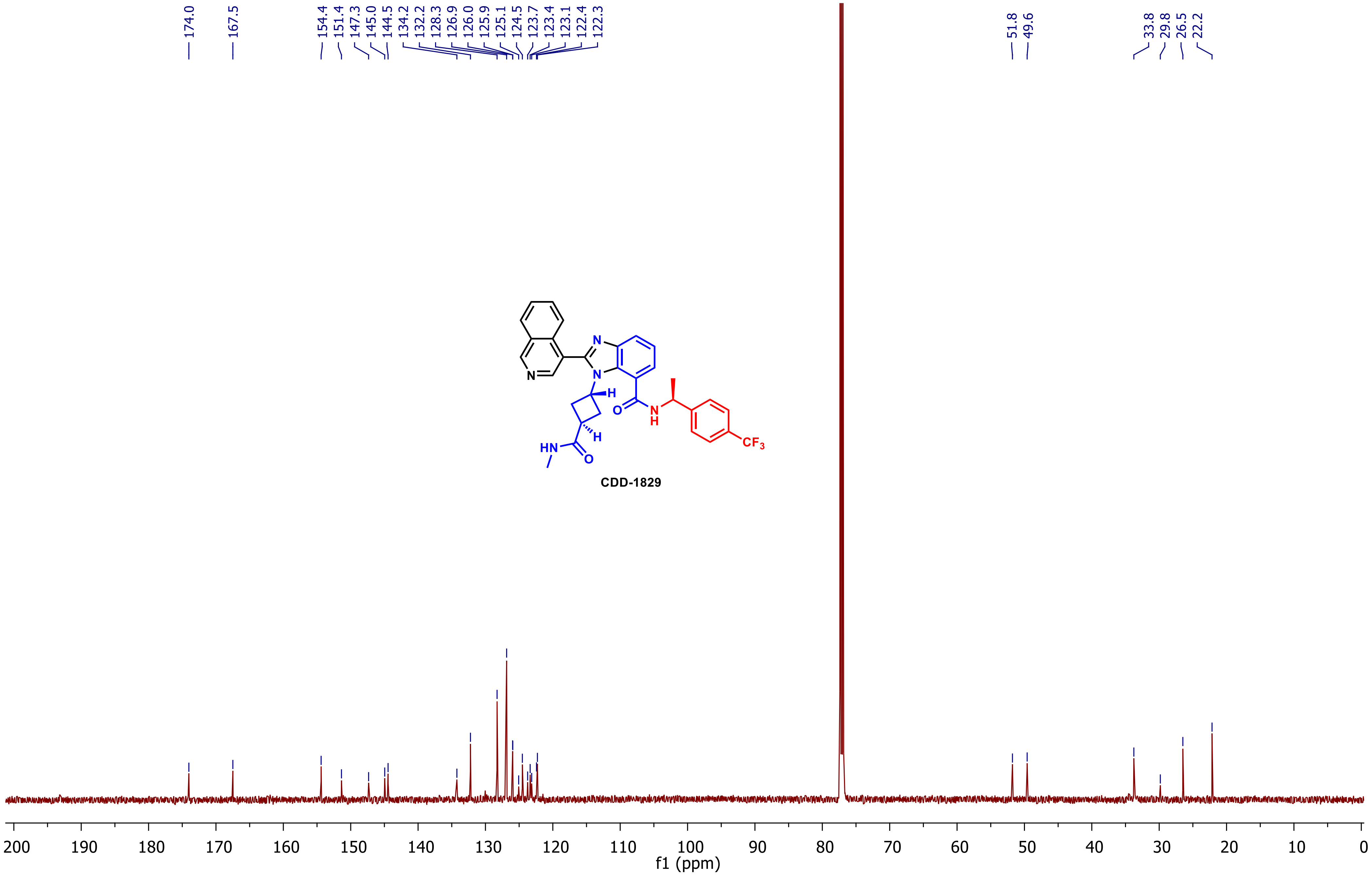
174.0
167.5
154.4
151.4
147.3
145.0
144.5
134.2
132.2
128.3
126.9
126.0
125.9
125.1
124.5
123.7
123.4
123.1
122.4
122.3
51.8
49.6
33.8
29.8
26.5
22.2
CDD-1829
200 190 180 170 160 150 140 130 120 110 100 90 80 70 60 50 40 30 20 10 0
f1 (ppm)

C:\Analytical\...\JRK-HRMS_MPRO\CDD1829 10/22/20 14:48:38

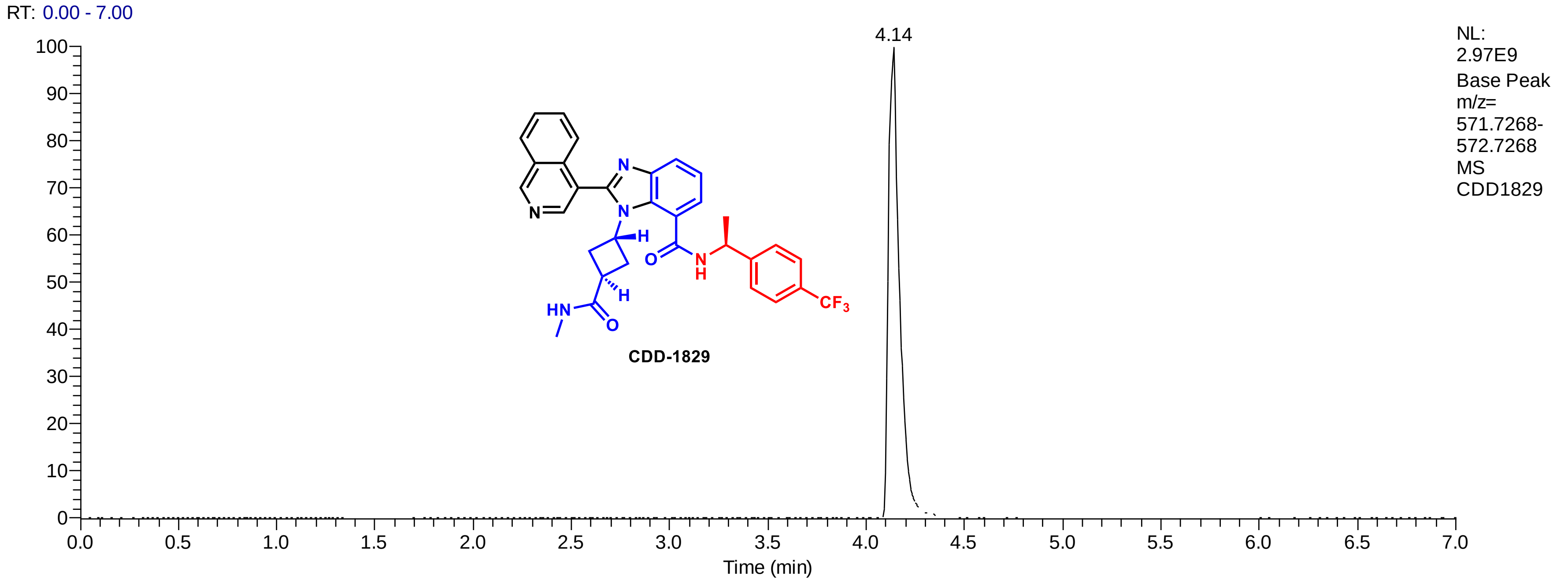

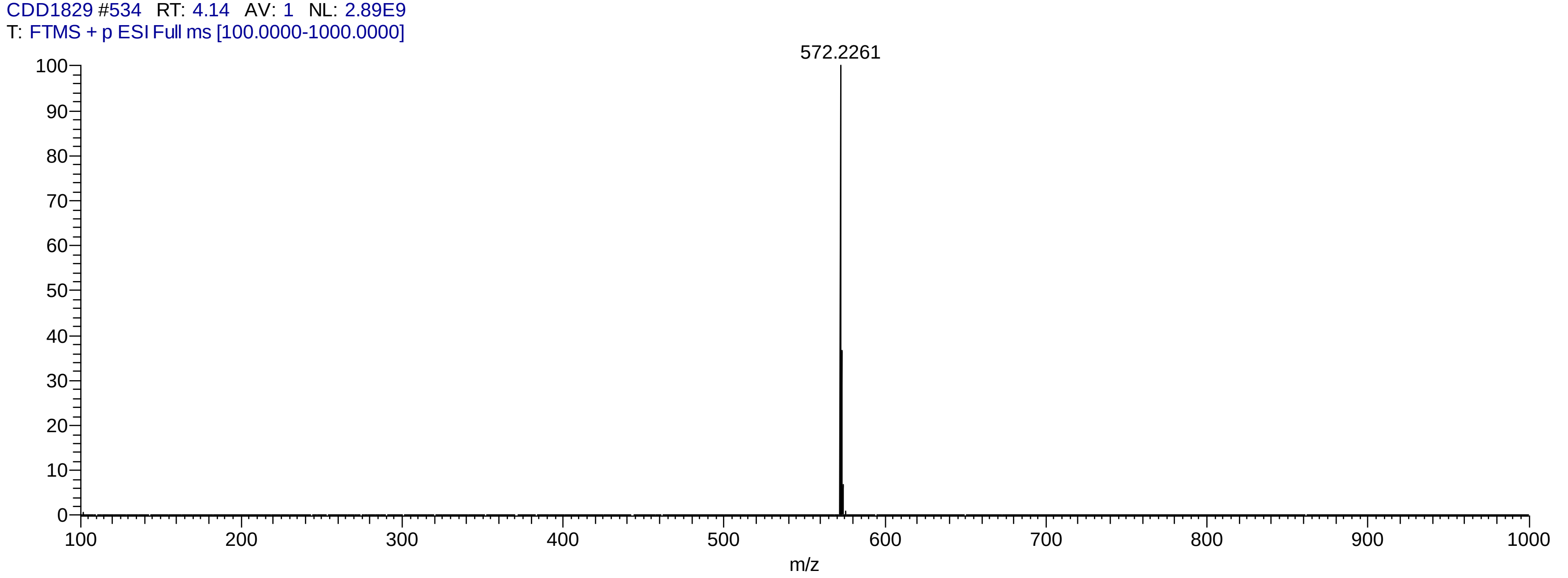

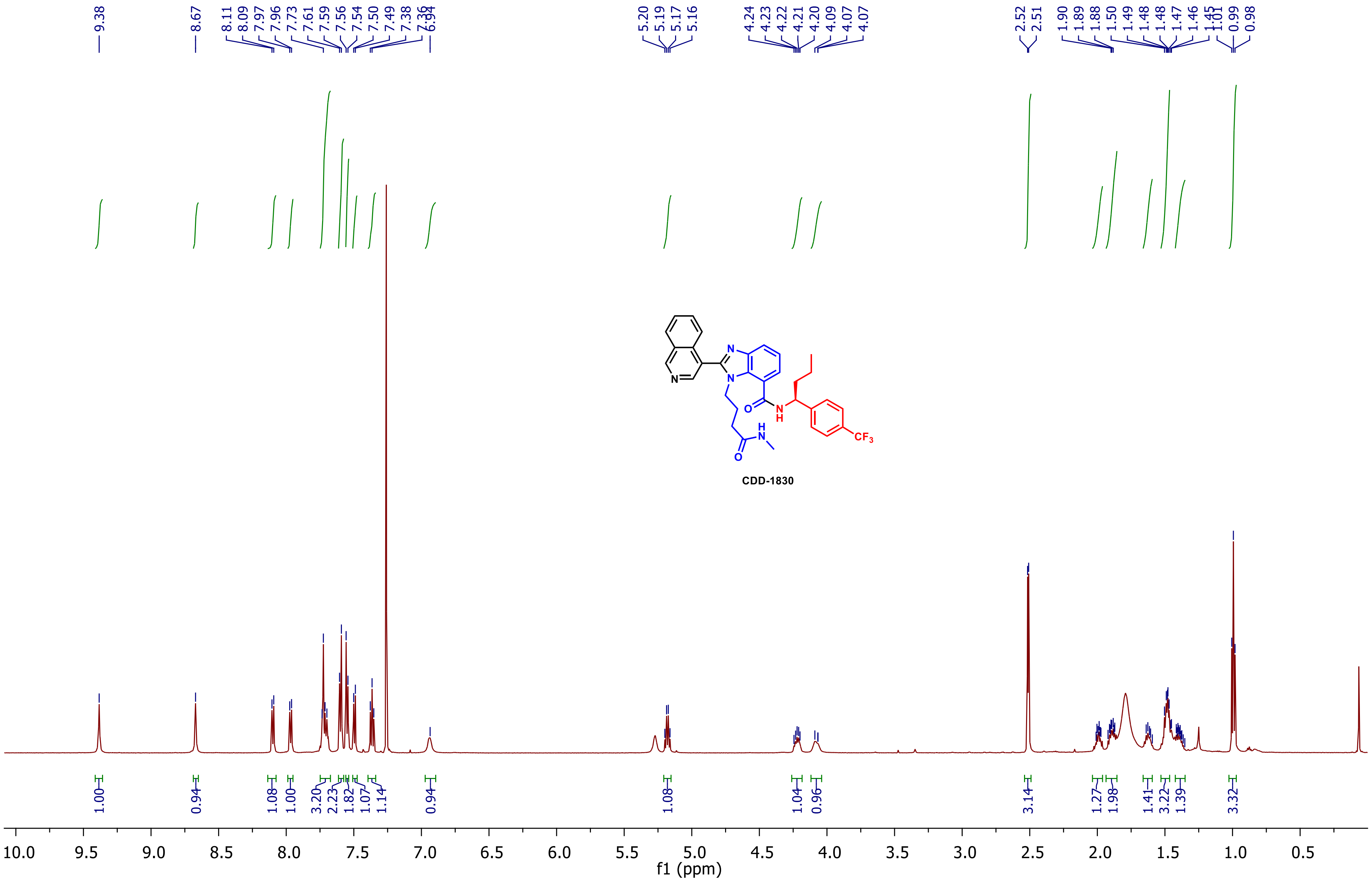
9.38
8.67
8.11 8.09 7.97 7.96 7.73 7.61 7.59 7.56 7.54 7.50 7.49 7.38 7.36 6.94
5.20 5.19 5.17 5.16
4.24 4.23 4.22 4.21 4.20 4.09 4.07 4.07
2.52 2.51
1.90 1.89 1.88 1.50 1.49 1.48 1.48 1.47 1.46 1.45 1.01 0.99 0.98
N
N
N
O
N
H
H
N
O
CF_3
CDD-1830
1.00 0.94 1.08 1.00 3.20 2.23 1.82 1.07 1.14 0.94 1.08 1.04 0.96 3.14 1.27 1.98 1.41 3.22 1.39 3.32
10.0 9.5 9.0 8.5 8.0 7.5 7.0 6.5 6.0 5.5 5.0 4.5 4.0 3.5 3.0 2.5 2.0 1.5 1.0 0.5
f1 (ppm)

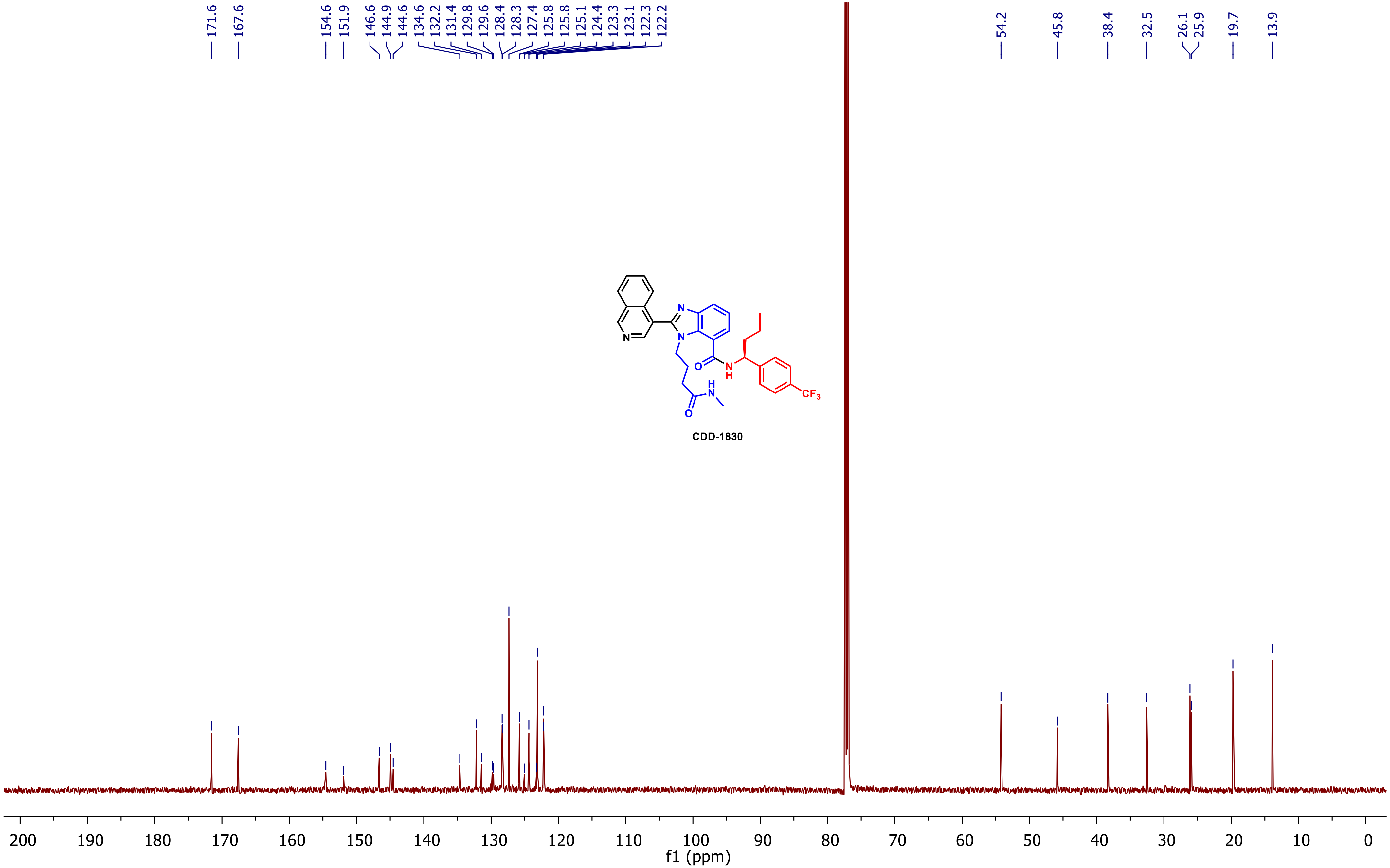
171.6
167.6
154.6
151.9
146.6
144.9
144.6
134.6
132.2
131.4
129.8
129.6
128.4
128.3
127.4
125.8
125.8
125.1
124.4
123.3
123.1
122.3
122.2
54.2
45.8
38.4
32.5
26.1
25.9
19.7
13.9
CDD-1830
200 190 180 170 160 150 140 130 120 110 100 90 80 70 60 50 40 30 20 10 0
f1 (ppm)

C:\Analytical\...\JRK-HRMS_MPRO\CDD1830 10/22/20 14:56:15

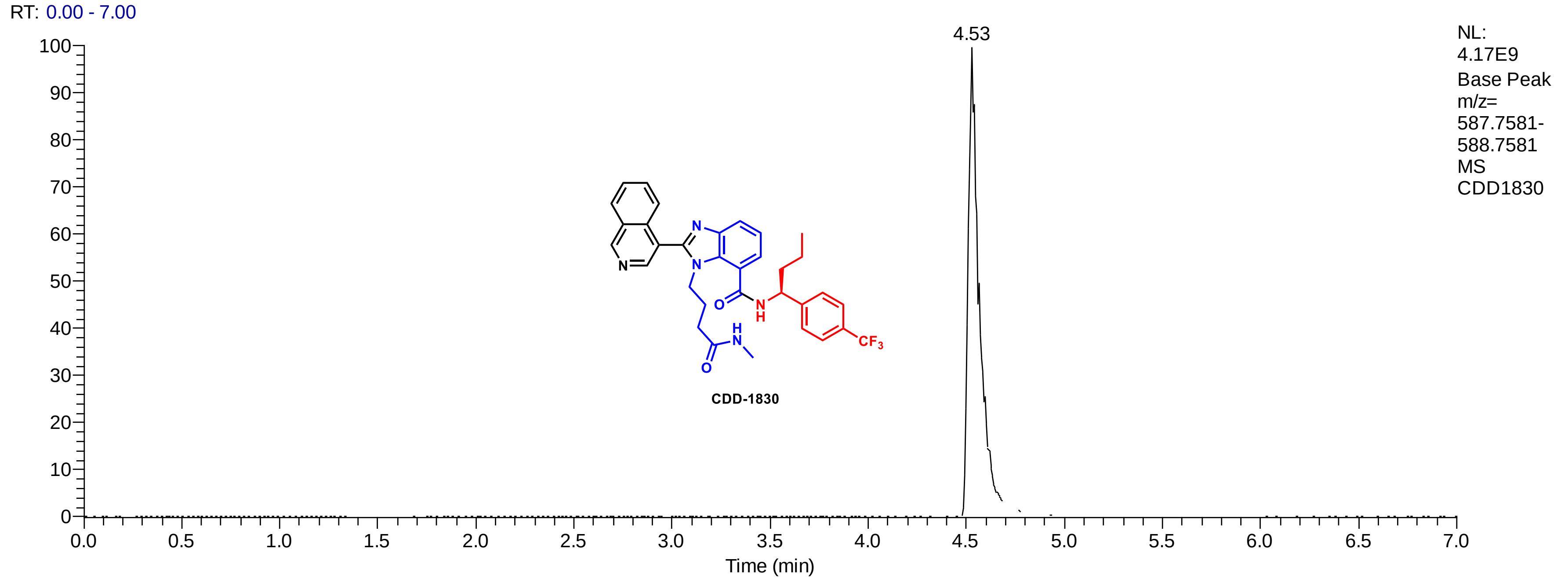

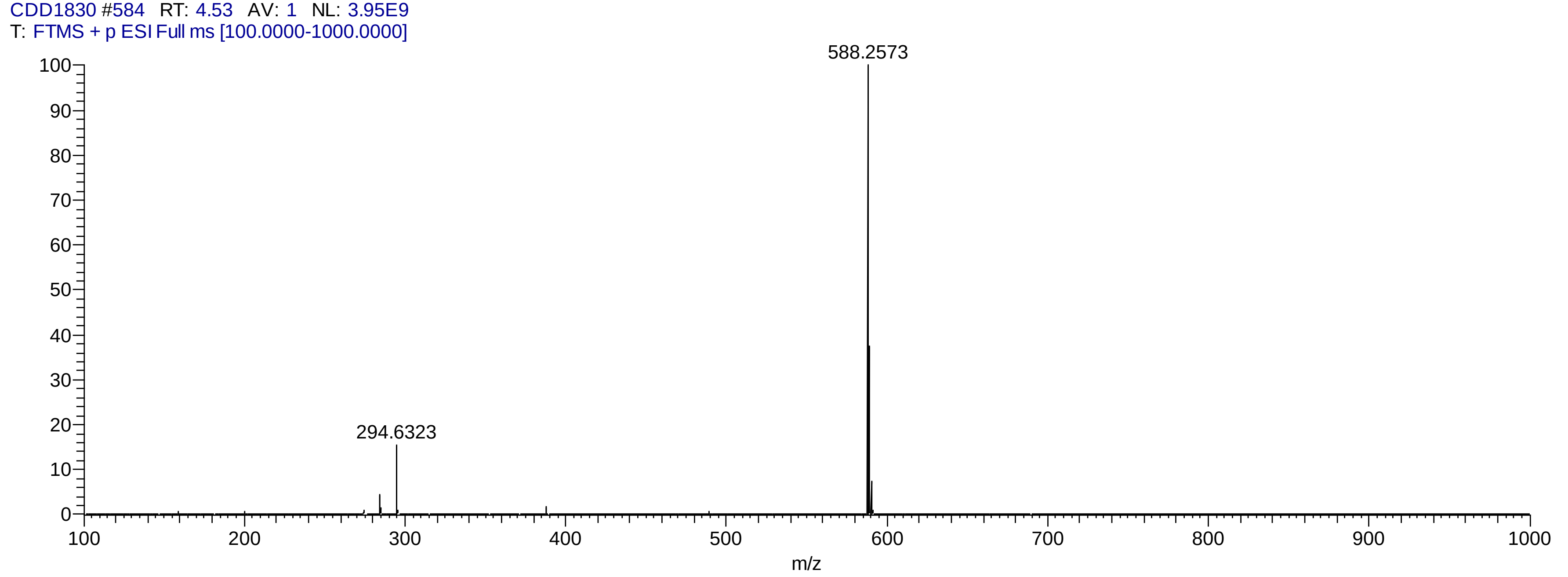

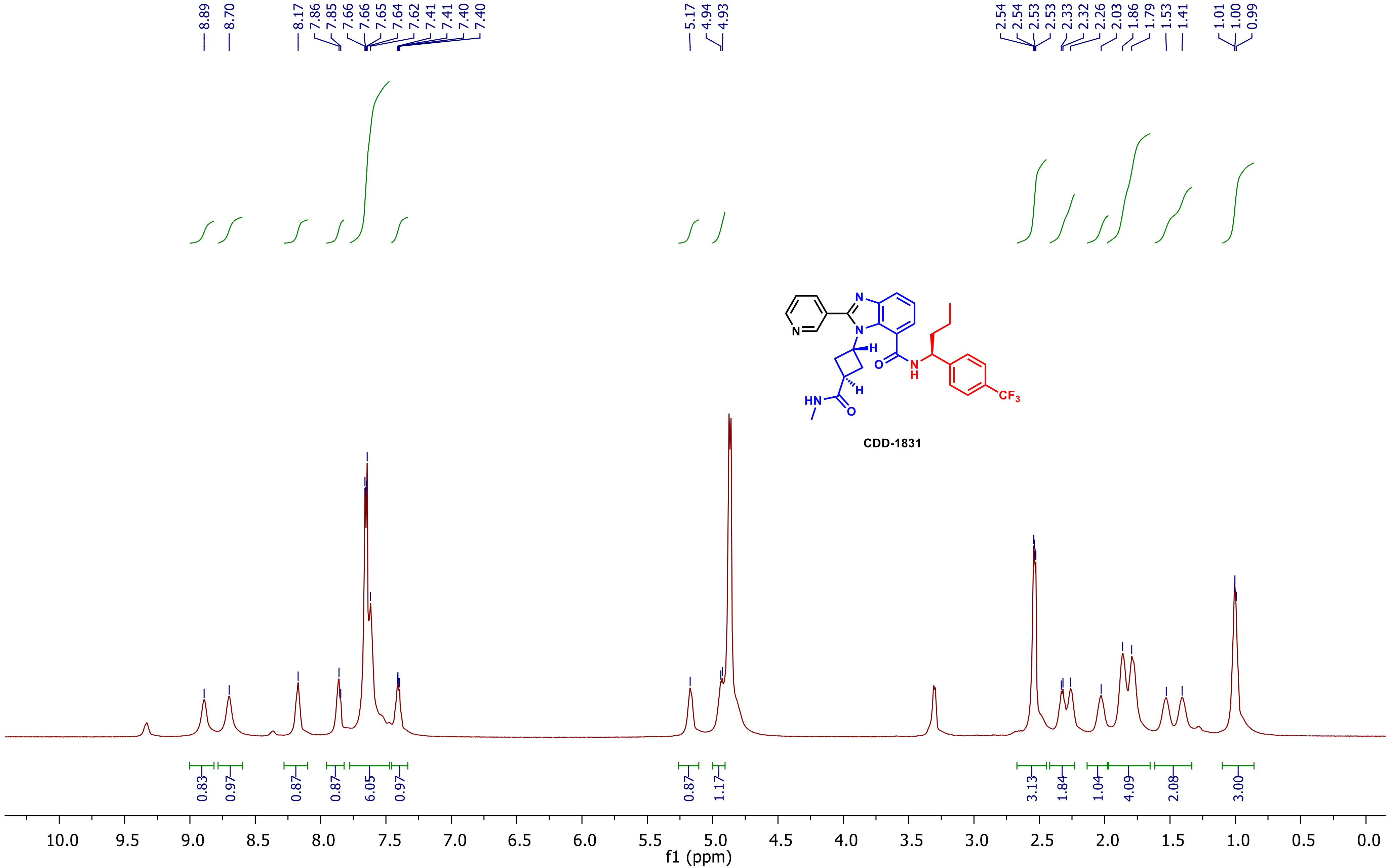
8.89
8.70
8.17
7.86
7.85
7.66
7.66
7.65
7.64
7.62
7.41
7.41
7.40
7.40
5.17
4.94
4.93
2.54
2.54
2.53
2.53
2.33
2.32
2.26
2.03
1.86
1.79
1.53
1.41
1.01
1.00
0.99
N
N
N
H
H
O
N
H
HN
O
CF_3
CDD-1831
0.83
0.97
0.87
0.87
6.05
0.97
0.87
1.17
3.13
1.84
1.04
4.09
2.08
3.00
10.0
9.5
9.0
8.5
8.0
7.5
7.0
6.5
6.0
5.5
5.0
4.5
4.0
3.5
3.0
2.5
2.0
1.5
1.0
0.5
0.0
f1 (ppm)

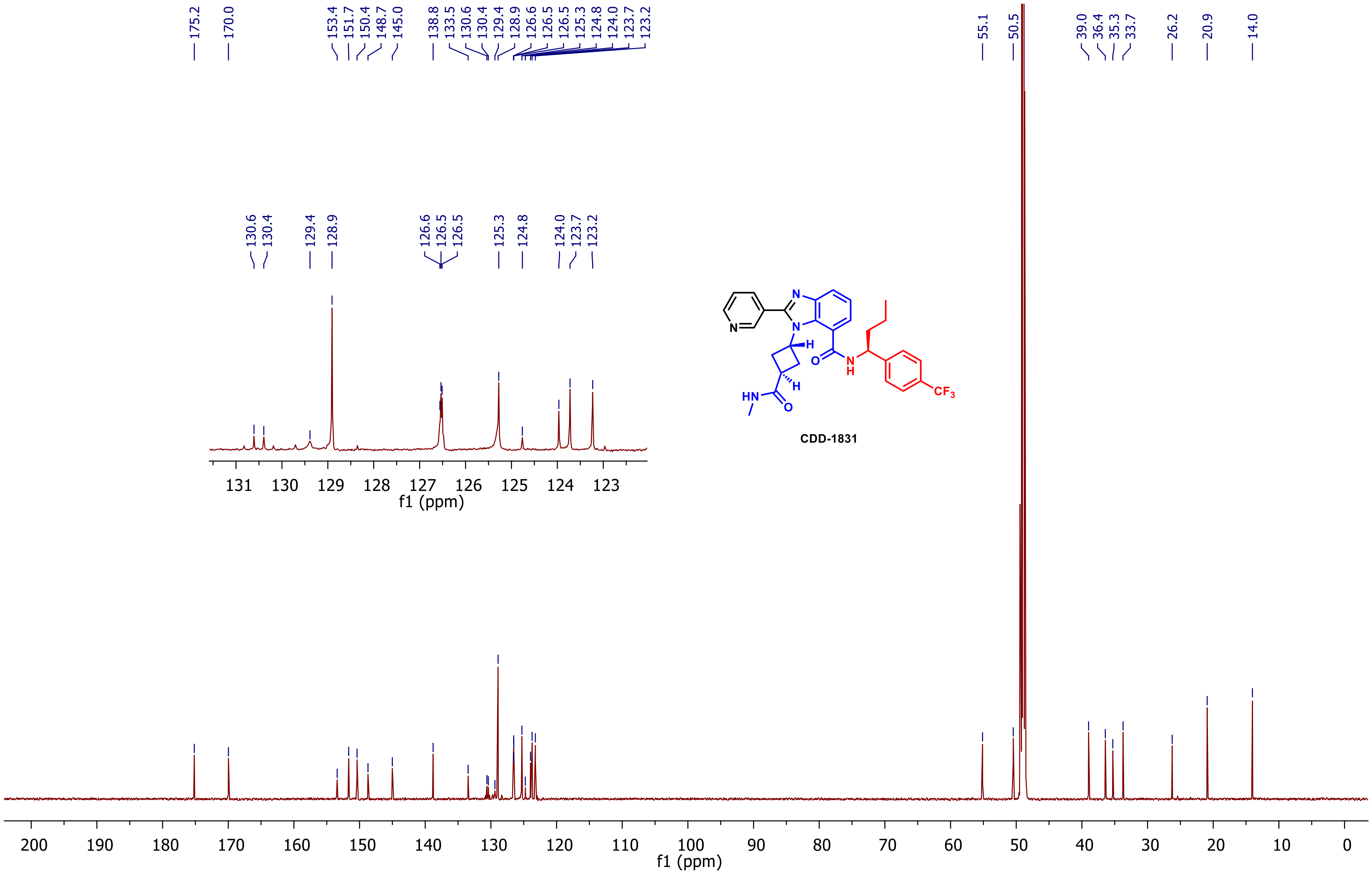

175.2
170.0
153.4
151.7
150.4
148.7
145.0
138.8
133.5
130.6
130.4
129.4
128.9
126.6
126.5
126.5
125.3
124.8
124.0
123.7
123.2
55.1
50.5
39.0
36.4
35.3
33.7
26.2
20.9
14.0
N
N
N
H
H
O
N
H
HN
O
CF3
f1 (ppm)

CDD-1831

C:\Analytical\Data\JRK\04052021\CDD1831 04/05/21 12:49:04

RT: 0.00 - 7.00 SM: 7B

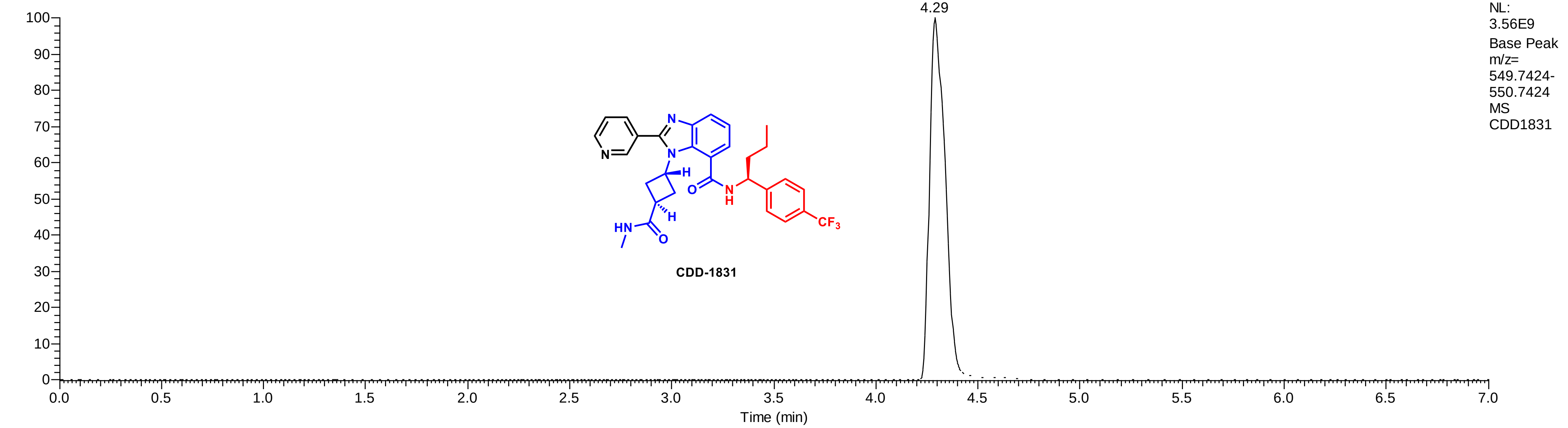

CDD1831 #668 RT: 4.29 AV: 1 NL: 3.64E9
T: FTMS + p ESI Full lock ms [100.0000-1000.0000]

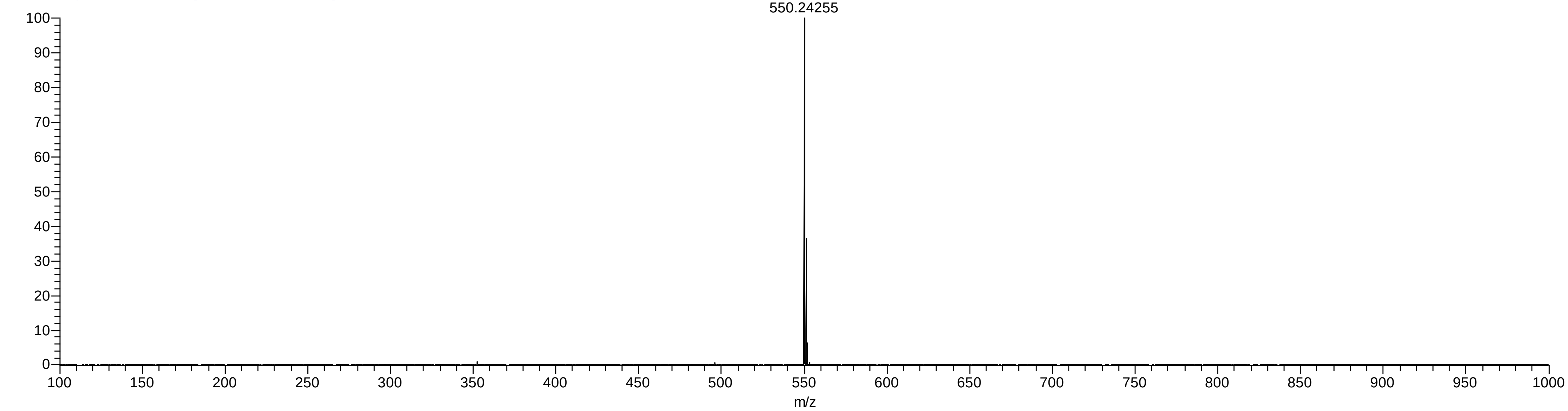

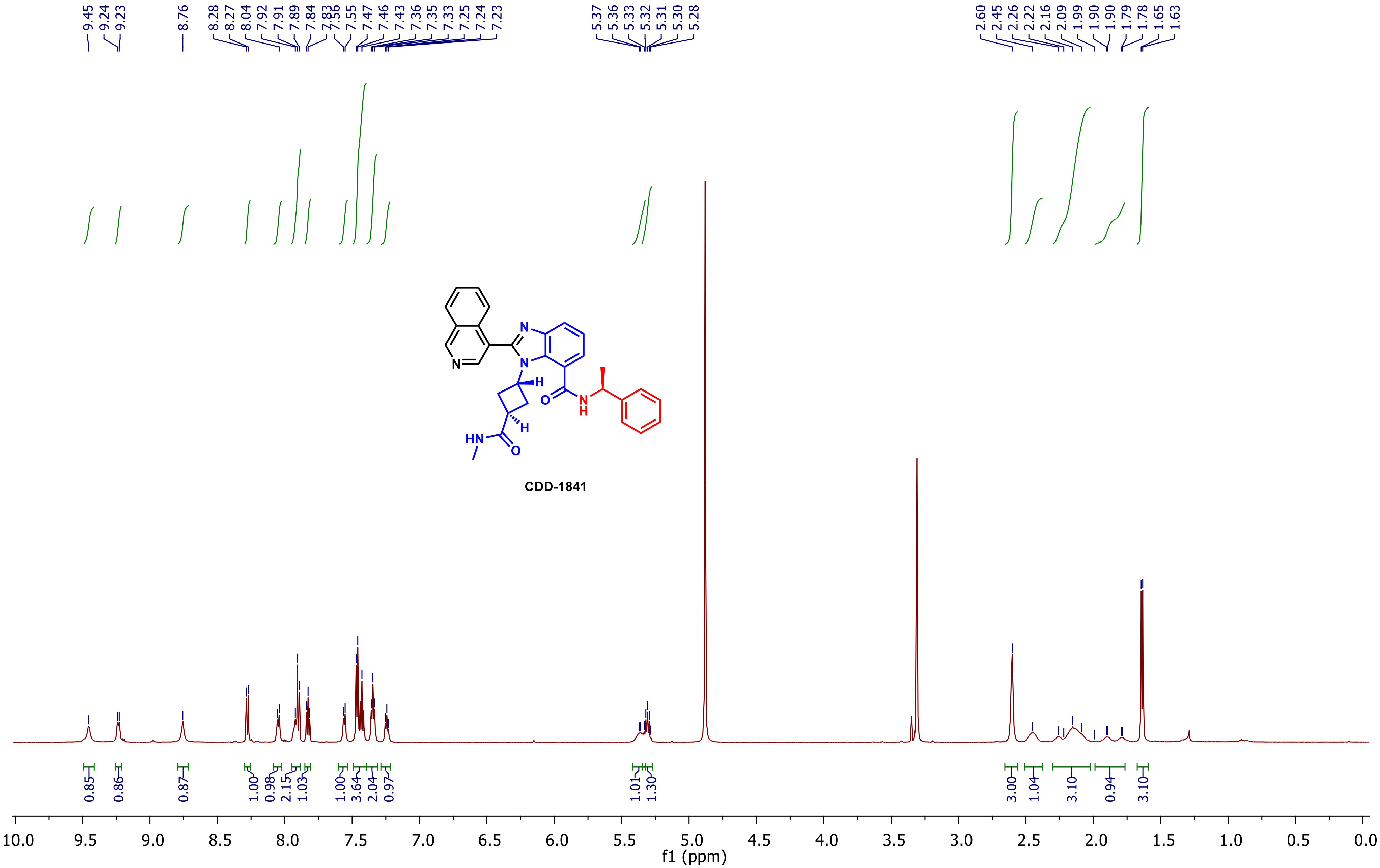
9.45 9.24 9.23 8.76 8.28 8.27 8.04 7.92 7.91 7.89 7.84 7.83 7.56 7.55 7.47 7.46 7.43 7.36 7.35 7.33 7.25 7.24 7.23
5.37 5.36 5.33 5.32 5.31 5.30 5.28
2.60 2.45 2.26 2.22 2.16 2.09 1.99 1.90 1.90 1.79 1.78 1.65 1.63
N
N
N
H
O
N
H
H
HN
O
CDD-1841
0.85 0.86 0.87 1.00 0.98 2.15 1.03 1.00 3.64 2.04 0.97
1.01 1.30
3.00 1.04 3.10 0.94 3.10
10.0 9.5 9.0 8.5 8.0 7.5 7.0 6.5 6.0 5.5 5.0 4.5 4.0 3.5 3.0 2.5 2.0 1.5 1.0 0.5 0.0
f1 (ppm)

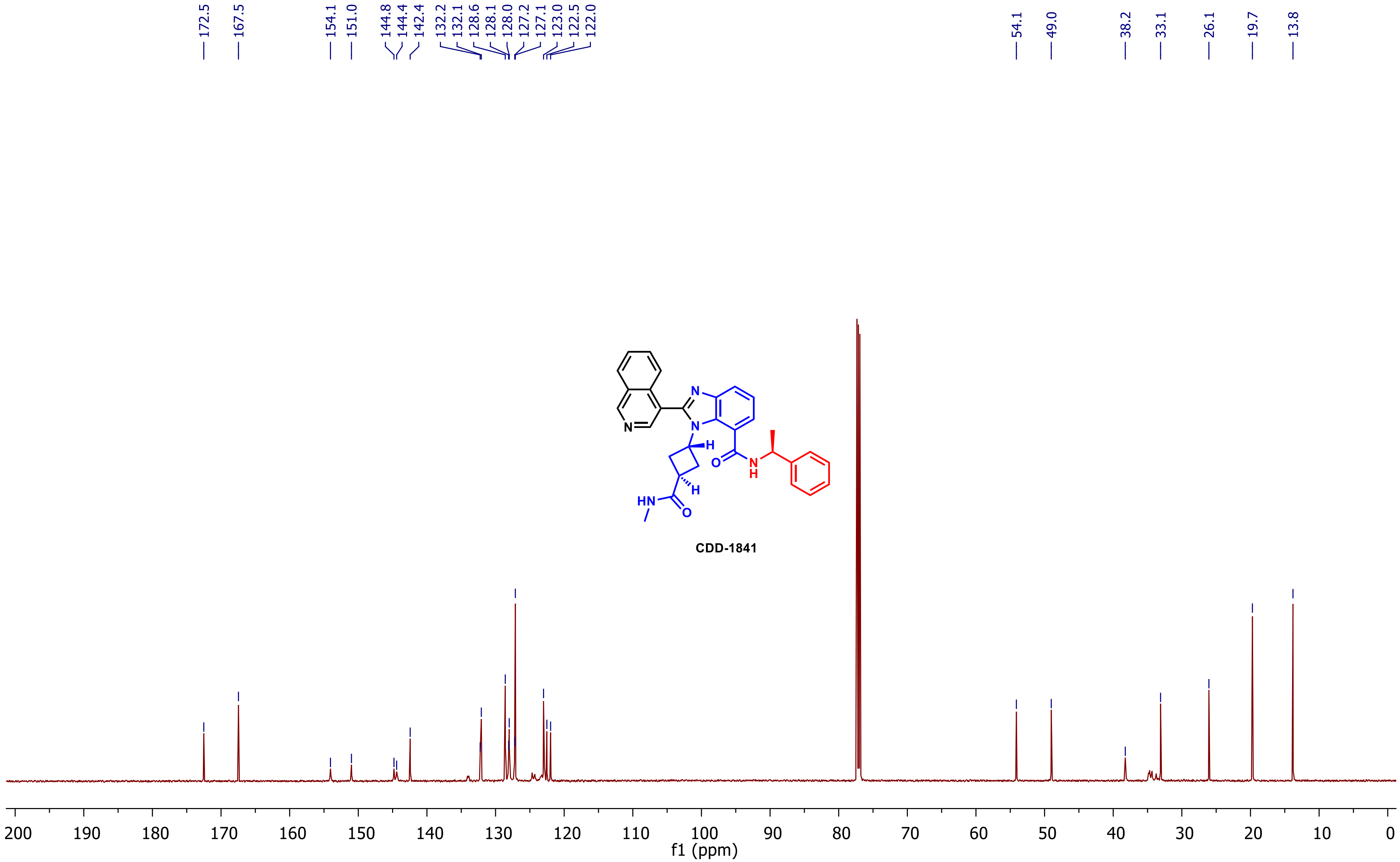
172.5
167.5
154.1
151.0
144.8
144.4
142.4
132.2
132.1
128.6
128.1
128.0
127.2
127.1
123.0
122.5
122.0
54.1
49.0
38.2
33.1
26.1
19.7
13.8
CDD-1841
200 190 180 170 160 150 140 130 120 110 100 90 80 70 60 50 40 30 20 10 0
f1 (ppm)

C:\Analytical\...\JRK-HRMS_MPRO\CDD1841 11/02/20 10:51:20

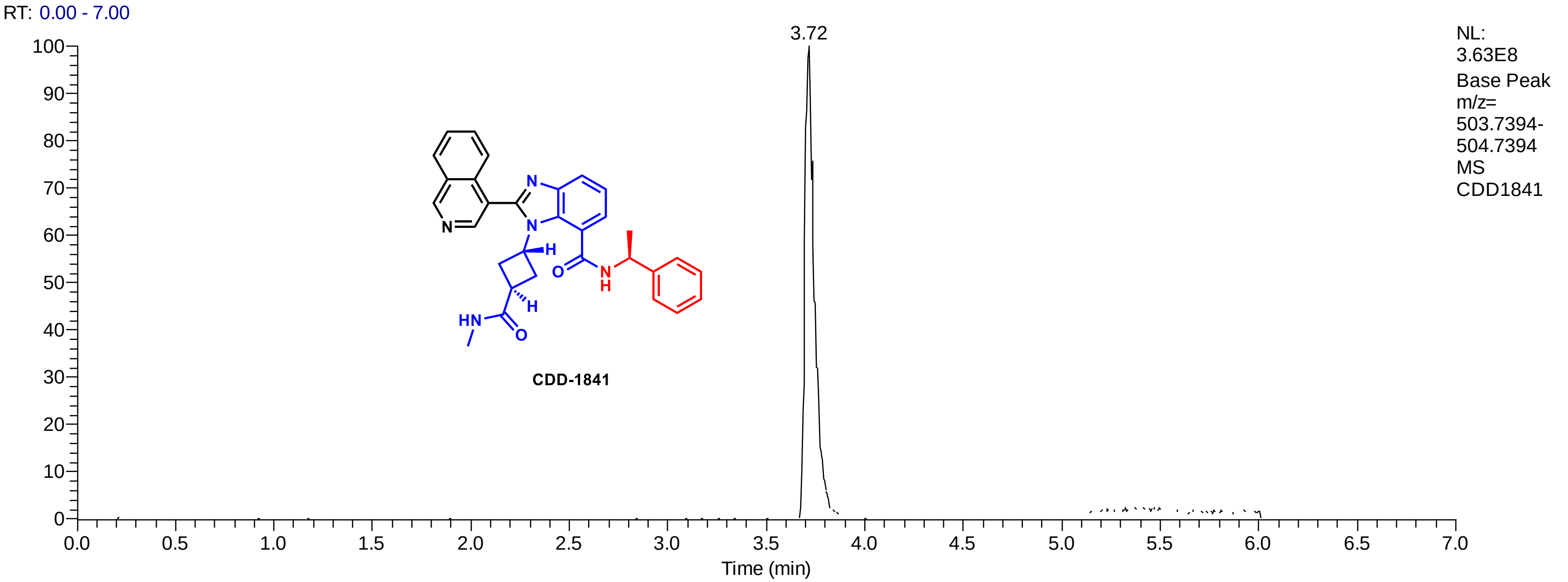

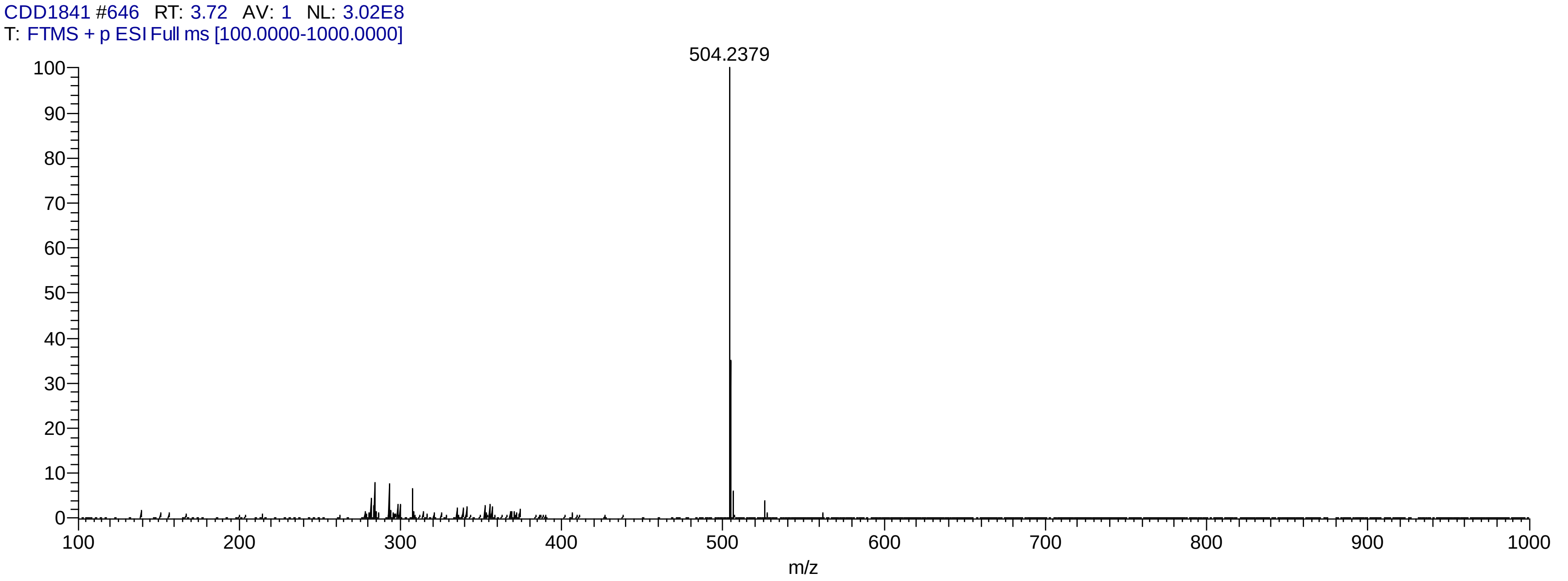

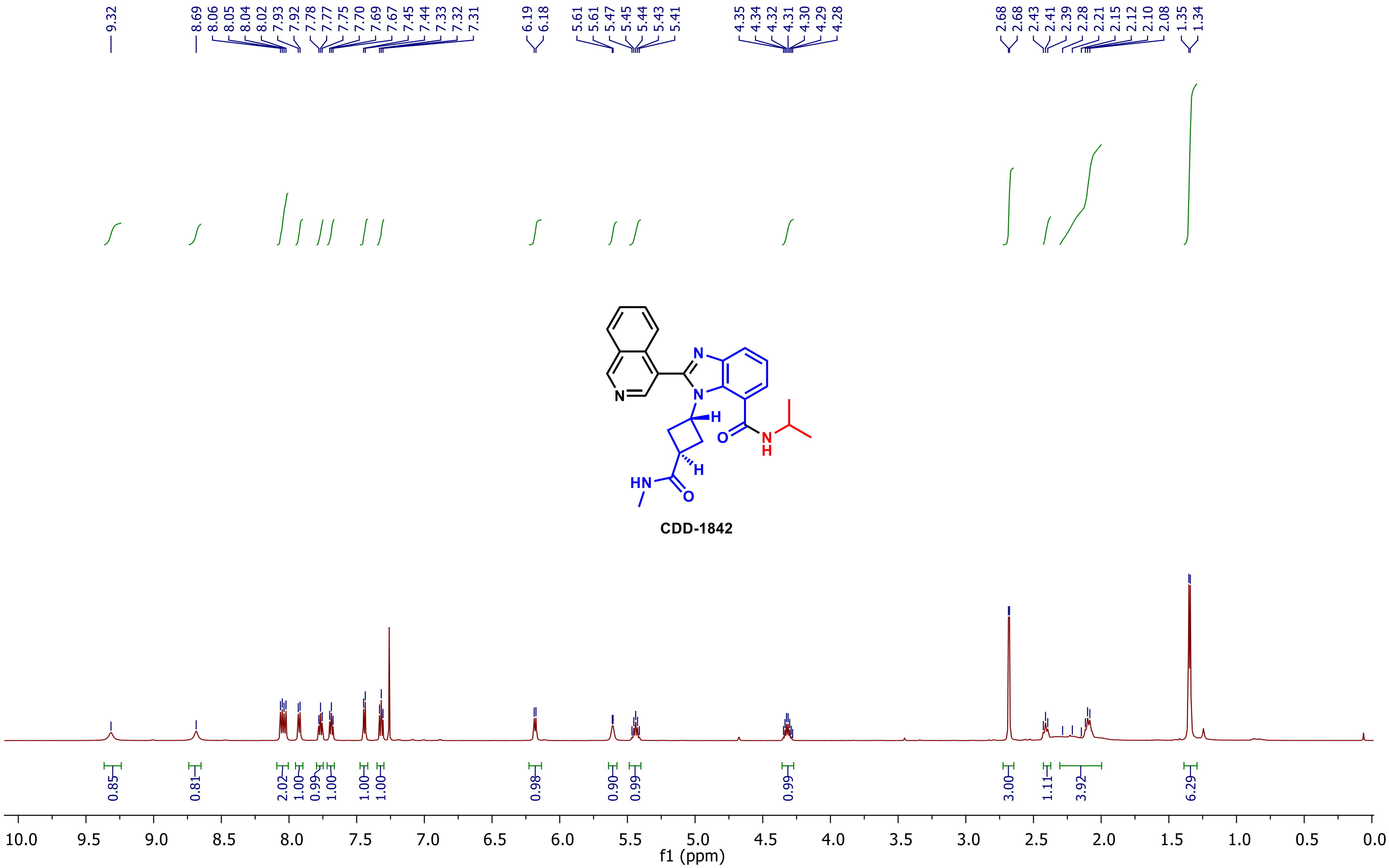
9.32
8.69
8.06
8.05
8.04
8.02
7.93
7.92
7.78
7.77
7.75
7.70
7.69
7.67
7.45
7.44
7.33
7.32
7.31
6.19
6.18
5.61
5.61
5.47
5.45
5.44
5.43
5.41
4.35
4.34
4.32
4.31
4.30
4.29
4.28
2.68
2.68
2.43
2.41
2.39
2.28
2.21
2.15
2.12
2.10
2.08
1.35
1.34
N
N
N
H
O
N
H
H
HN
O
CDD-1842
0.85
0.81
2.02
1.00
0.99
1.00
1.00
1.00
0.98
0.90
0.99
0.99
3.00
1.11
3.92
6.29
10.0
9.5
9.0
8.5
8.0
7.5
7.0
6.5
6.0
5.5
5.0
4.5
4.0
3.5
3.0
2.5
2.0
1.5
1.0
0.5
0.0
f1 (ppm)

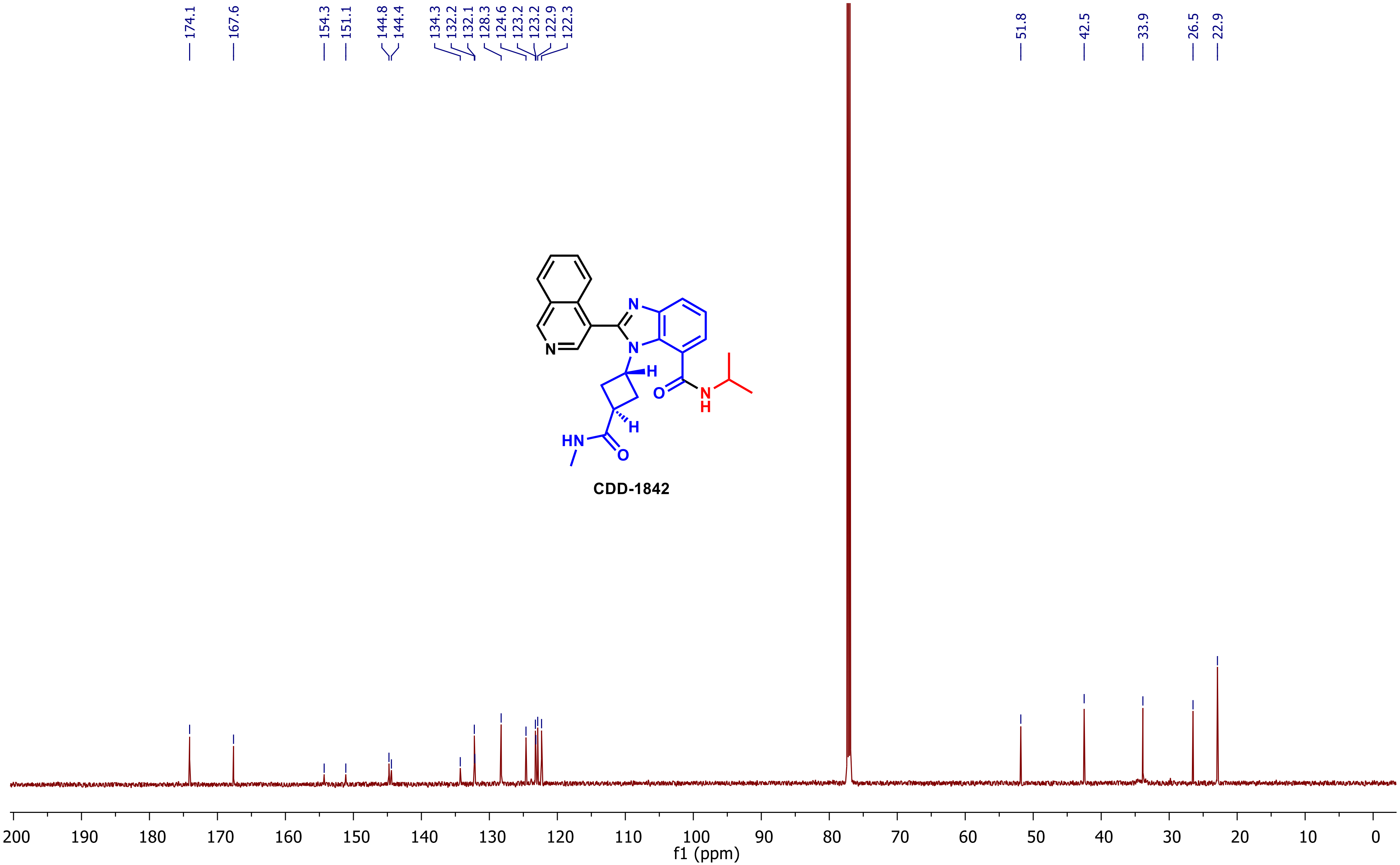

174.1
167.6
154.3
151.1
144.8
144.4
134.3
132.2
132.1
128.3
124.6
123.2
123.2
122.9
122.3
51.8
42.5
33.9
26.5
22.9
N
N
N
H
H
O
N
H
HN
O
CDD-1842
200 190 180 170 160 150 140 130 120 110 100 90 80 70 60 50 40 30 20 10 0
f1 (ppm)

C:\Analytical\...\JRK-HRMS_MPRO\CDD1842 11/02/20 10:58:55

RT: 0.00 - 7.00

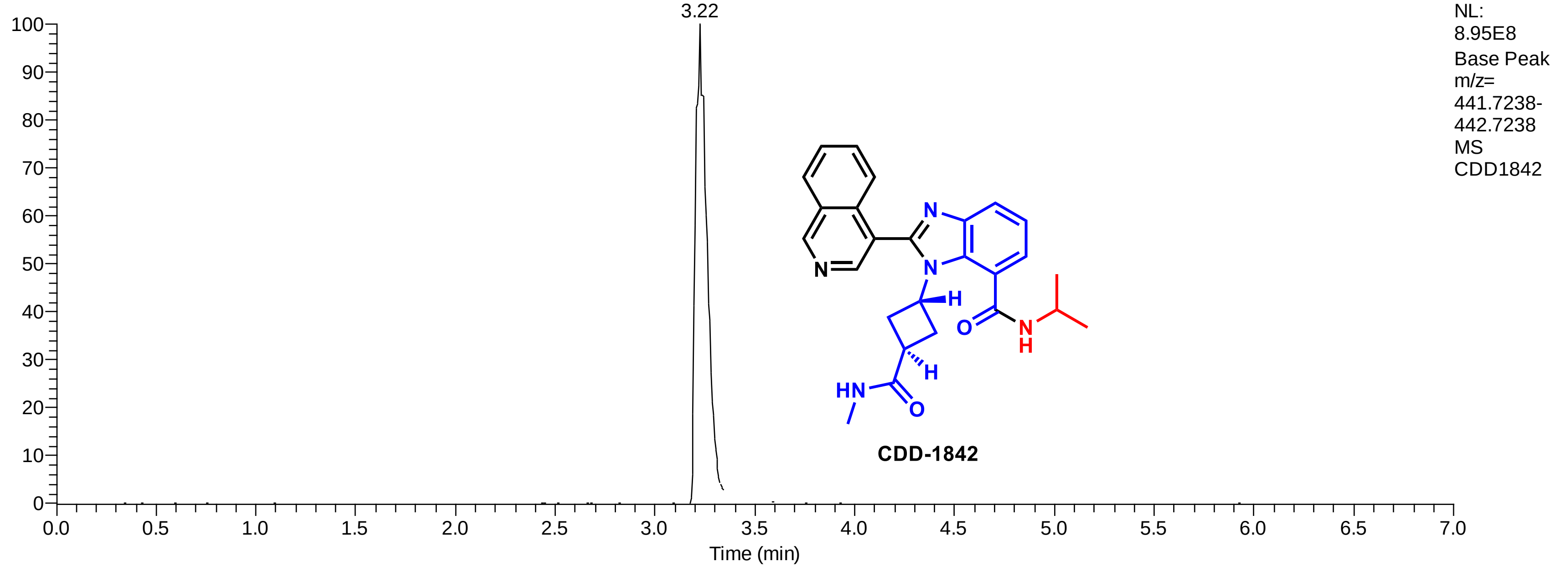

CDD1842 #538 RT: 3.22 AV: 1 NL: 8.88E8
T: FTMS + p ESI Full ms [100.0000-1000.0000]

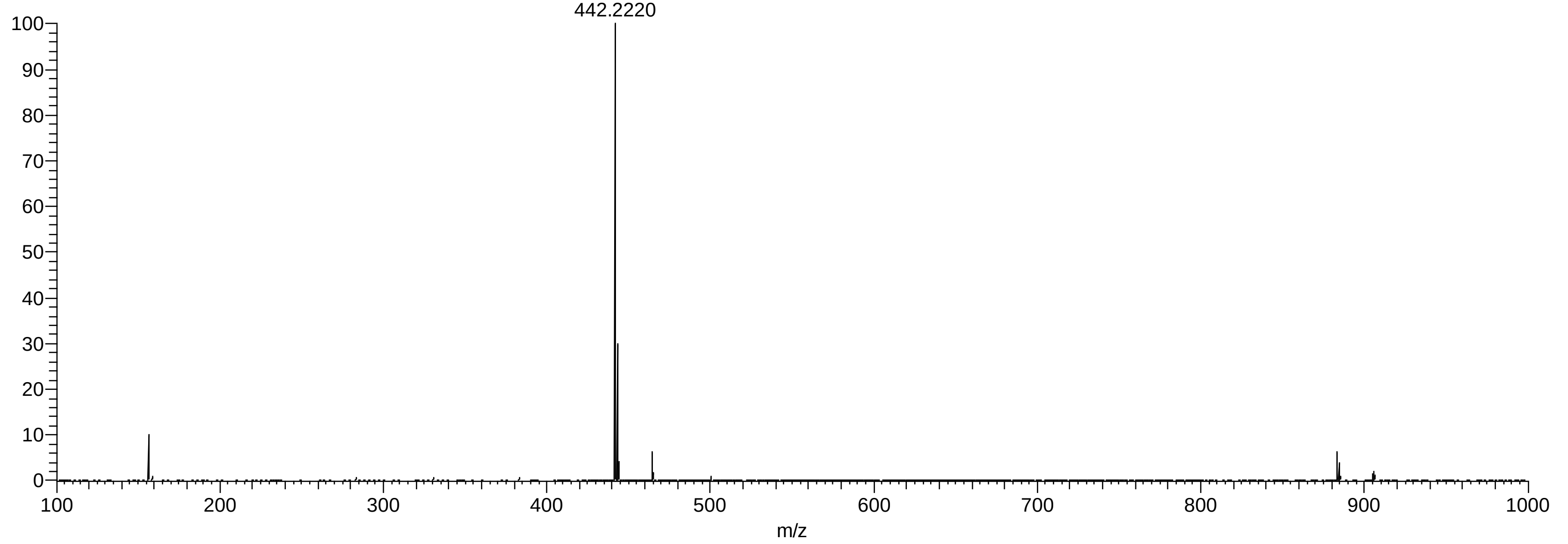

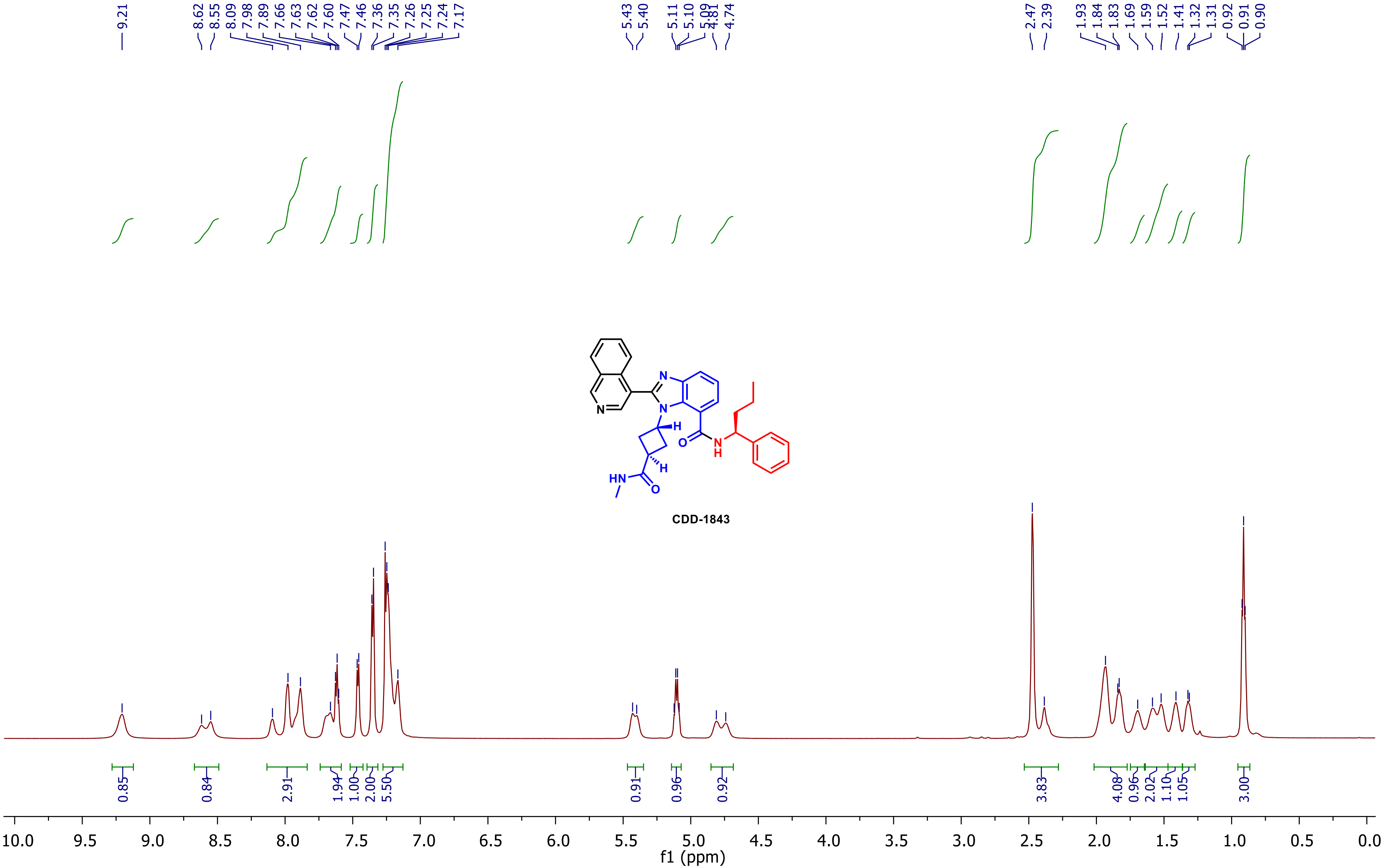

9.21
8.62 8.55 8.09 7.98 7.89 7.66 7.63 7.62 7.60 7.47 7.46 7.36 7.35 7.26 7.25 7.24 7.17
5.43 5.40 5.11 5.10 5.09 4.81 4.74
2.47 2.39 1.93 1.84 1.83 1.69 1.59 1.52 1.41 1.32 1.31 0.92 0.91 0.90
CDD-1843
0.85 0.84 2.91 1.94 1.00 2.00 5.50 0.91 0.96 0.92 3.83 4.08 0.96 2.02 1.10 1.05 3.00
10.0 9.5 9.0 8.5 8.0 7.5 7.0 6.5 6.0 5.5 5.0 4.5 4.0 3.5 3.0 2.5 2.0 1.5 1.0 0.5 0.0
f1 (ppm)

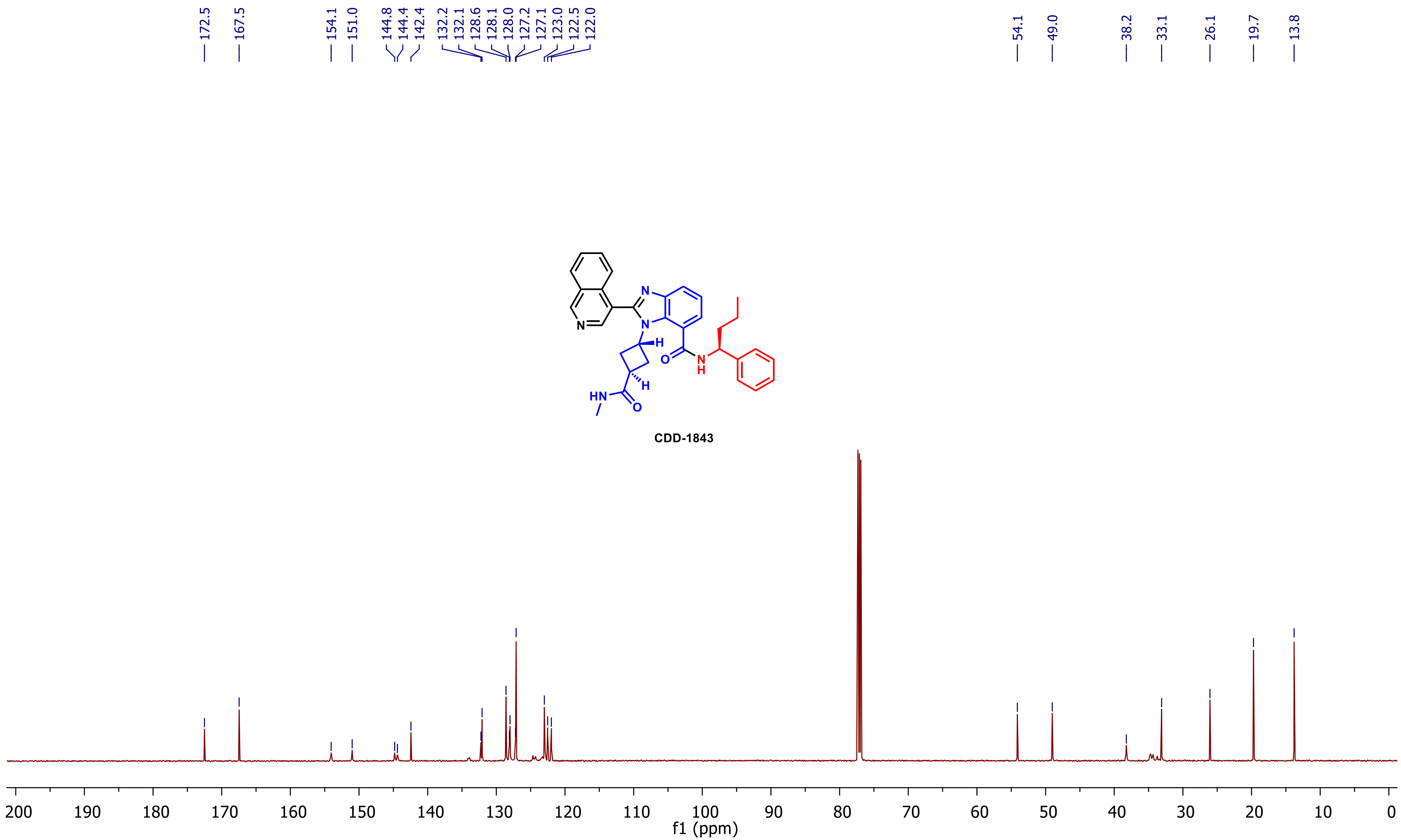
172.5
167.5
154.1
151.0
144.8
144.4
142.4
132.2
132.1
128.6
128.1
128.0
127.2
127.1
123.0
122.5
122.0
54.1
49.0
38.2
33.1
26.1
19.7
13.8
N
N
N
H
O
N
H
HN
H
O
CDD-1843
200
190
180
170
160
150
140
130
120
110
100
90
80
70
60
50
40
30
20
10
0
f1 (ppm)

C:\Analytical\...\11122020\CDD1843 11/12/20 12:12:40

RT: 0.00 - 7.00 SM: 7B

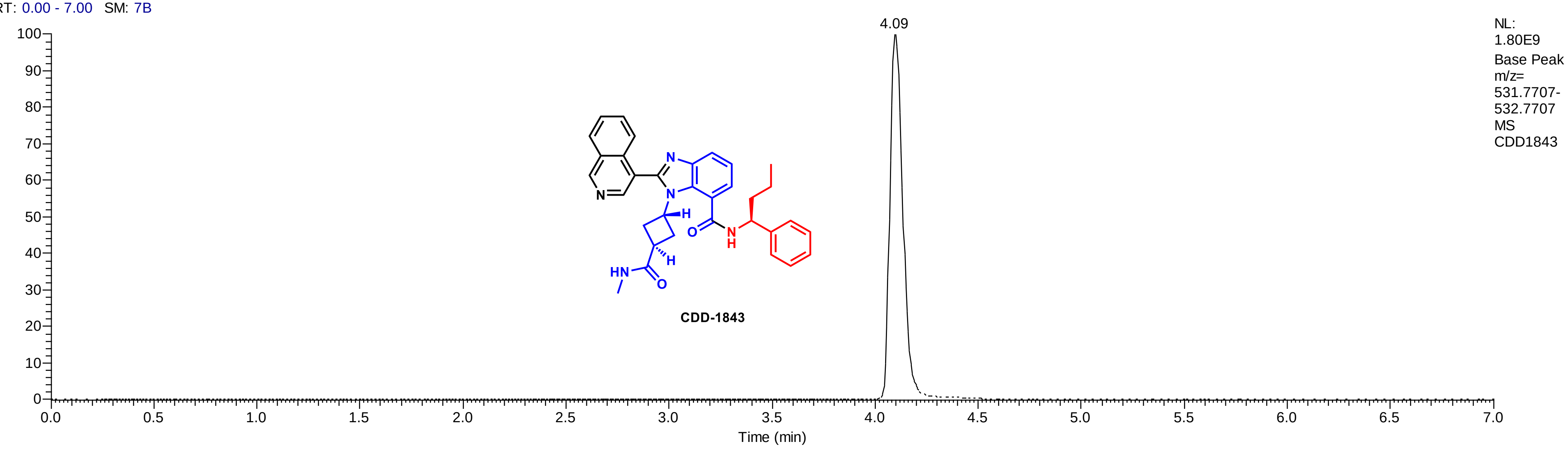

CDD1843 #557 RT: 4.09 AV: 1 NL: 1.99E9
T: FTMS + p ESI Full ms [100.0000-1000.0000]

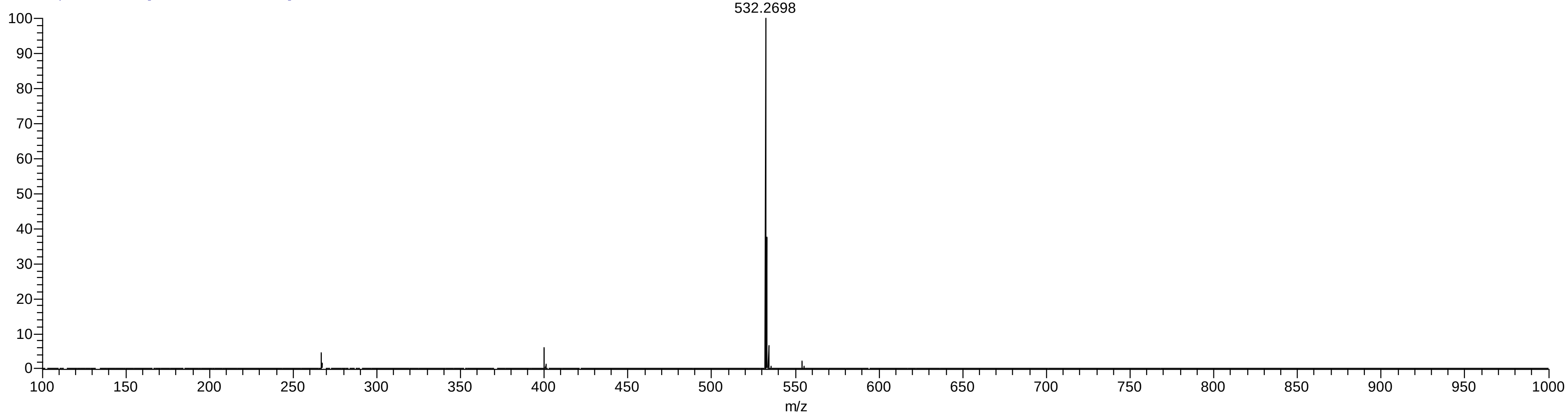

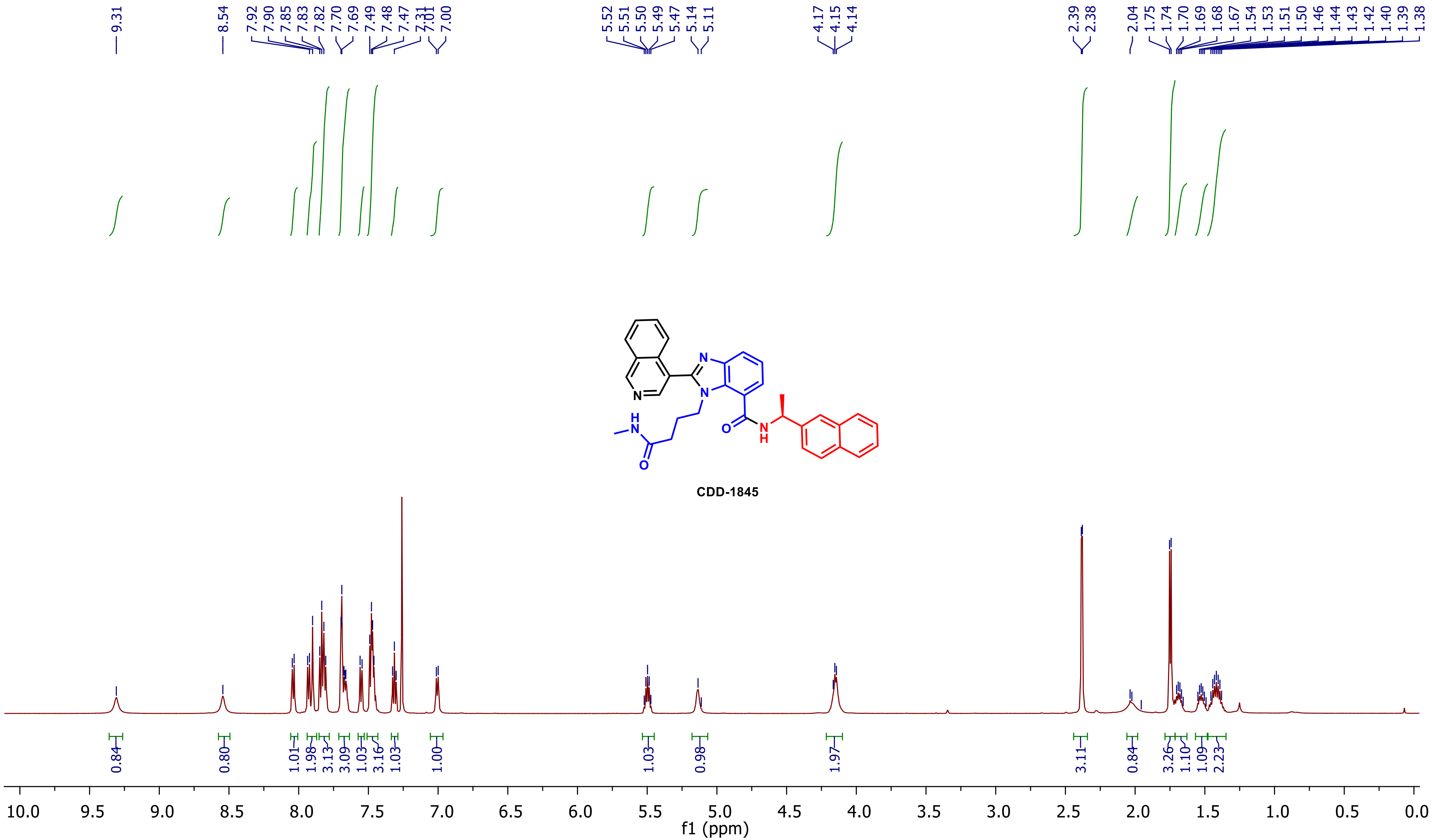
9.31
8.54
7.92
7.90
7.85
7.83
7.82
7.70
7.69
7.49
7.48
7.47
7.31
7.01
7.00
5.52
5.51
5.50
5.49
5.47
5.14
5.11
4.17
4.15
4.14
2.39
2.38
2.04
1.75
1.74
1.70
1.69
1.68
1.67
1.54
1.53
1.51
1.50
1.46
1.44
1.43
1.42
1.40
1.39
1.38
N
N
N
H
N
O
N
H
O
CDD-1845
0.84
0.80
1.01
1.98
3.13
3.09
1.03
3.16
1.03
1.00
1.03
0.98
1.97
3.11
0.84
3.26
1.10
1.09
2.23
10.0
9.5
9.0
8.5
8.0
7.5
7.0
6.5
6.0
5.5
5.0
4.5
4.0
3.5
3.0
2.5
2.0
1.5
1.0
0.5
0.0
f1 (ppm)

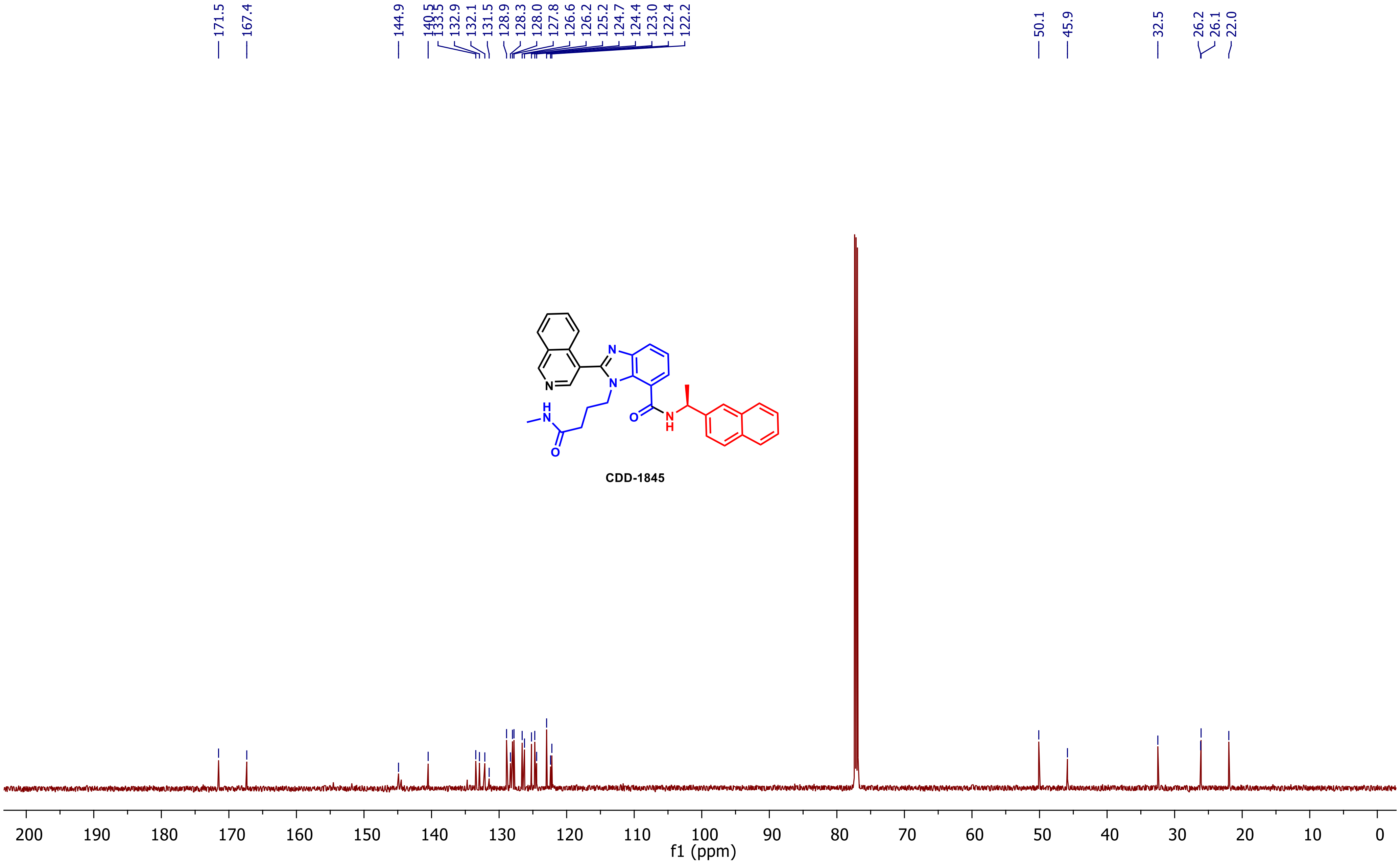
171.5
167.4
144.9
140.5
133.5
132.9
132.1
131.5
128.9
128.3
128.0
127.8
126.6
126.2
125.2
124.7
124.4
123.0
122.4
122.2
50.1
45.9
32.5
26.2
26.1
22.0
200 190 180 170 160 150 140 130 120 110 100 90 80 70 60 50 40 30 20 10 0
f1 (ppm)

CDD-1845

C:\Analytical\...\JRK-HRMS_MPRO\CDD1845 11/09/20 11:50:39

RT: 0.00 - 7.01

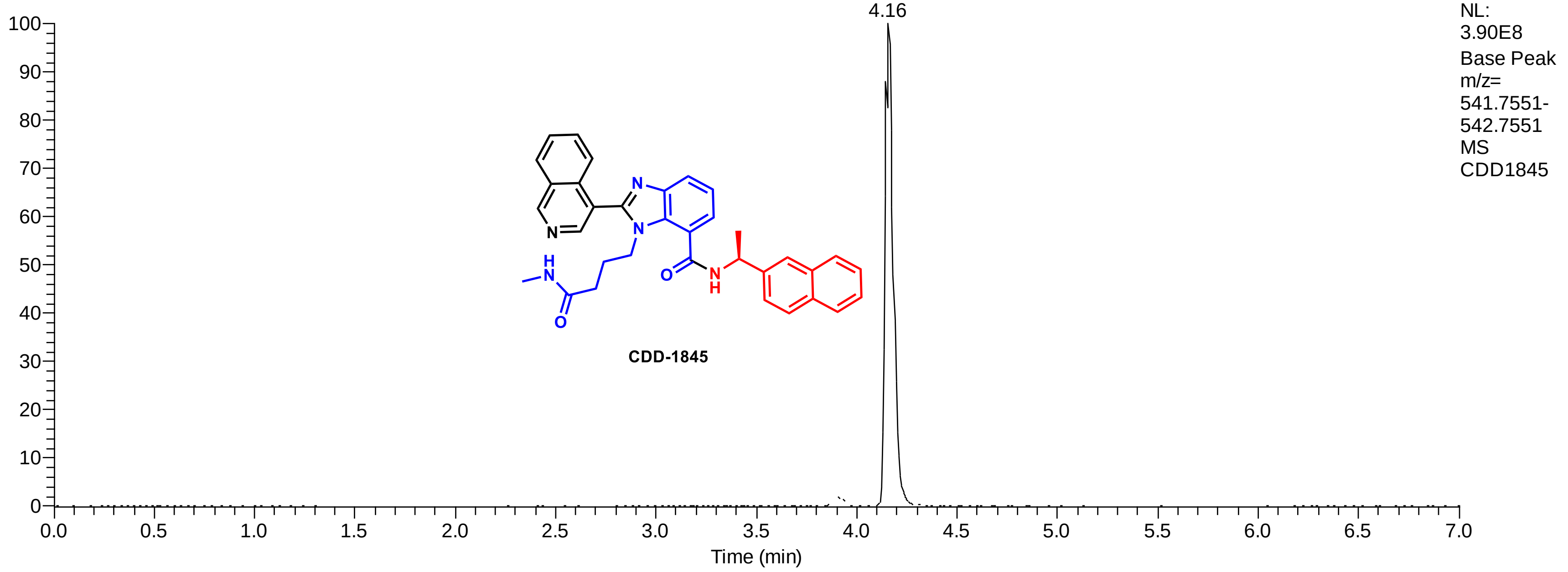

CDD1845 #603 RT: 4.16 AV: 1 NL: 3.82E8
T: FTMS + p ESI Full ms [100.0000-1000.0000]

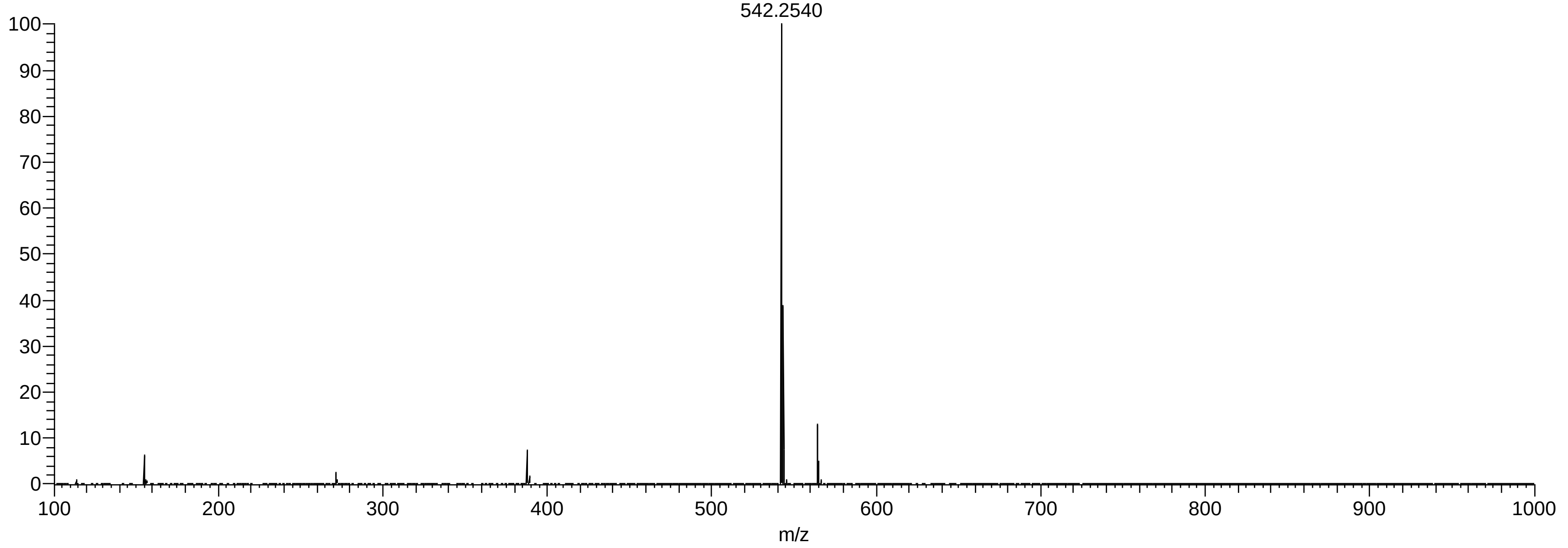

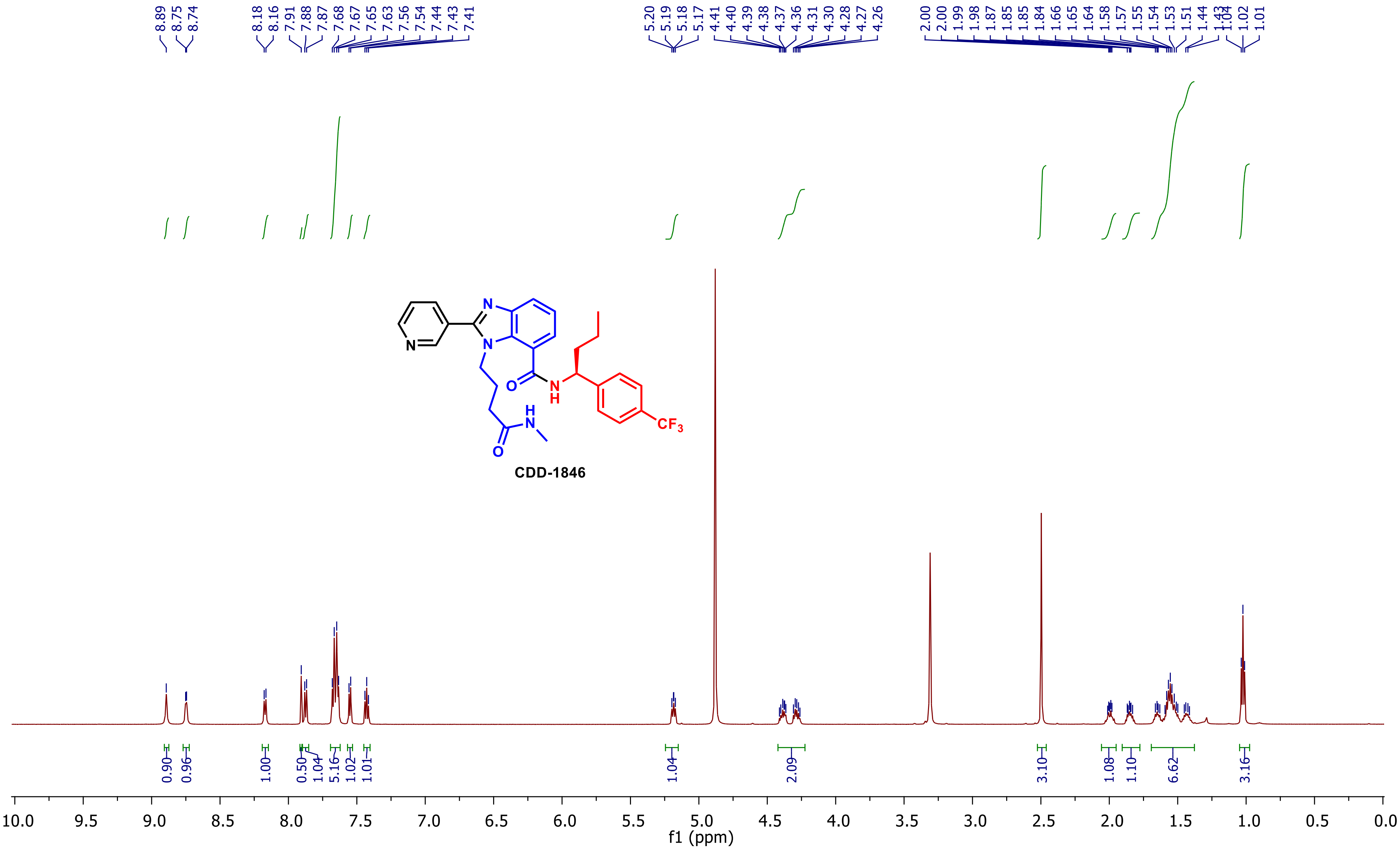
CDD-1846
f1 (ppm)

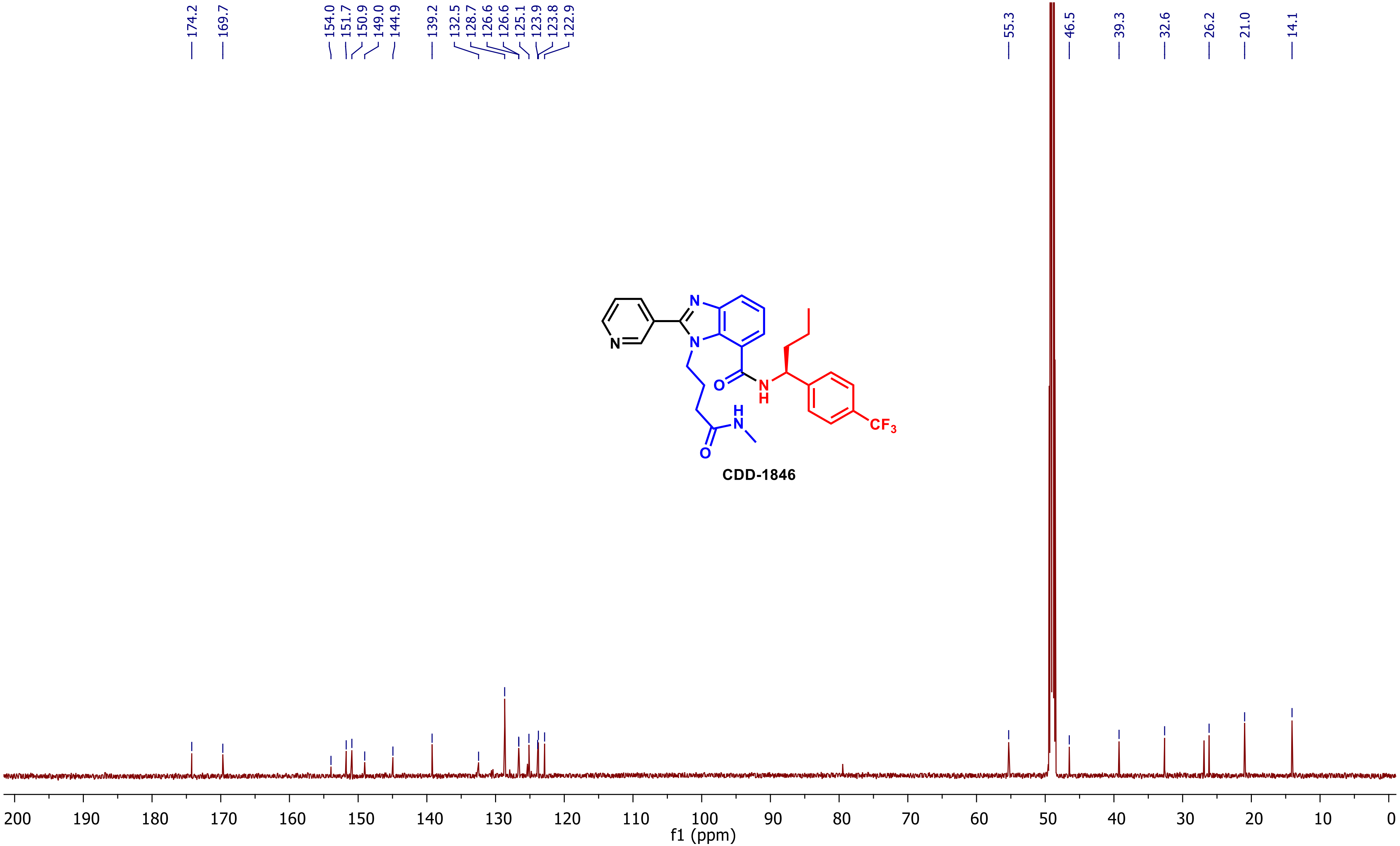
174.2
169.7
154.0
151.7
150.9
149.0
144.9
139.2
132.5
128.7
126.6
126.6
125.1
123.9
123.8
122.9
55.3
46.5
39.3
32.6
26.2
21.0
14.1
CDD-1846
f1 (ppm)

C:\Analytical\...\04012021\CDD1846 04/01/21 16:17:45

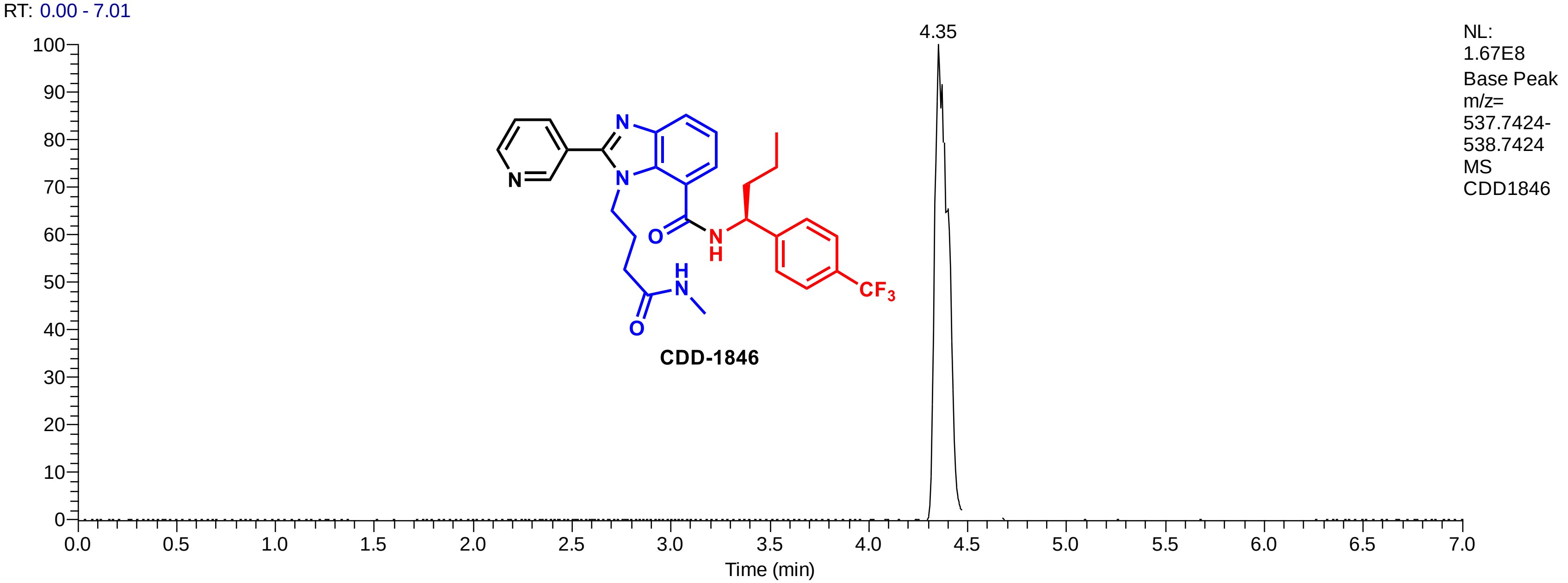

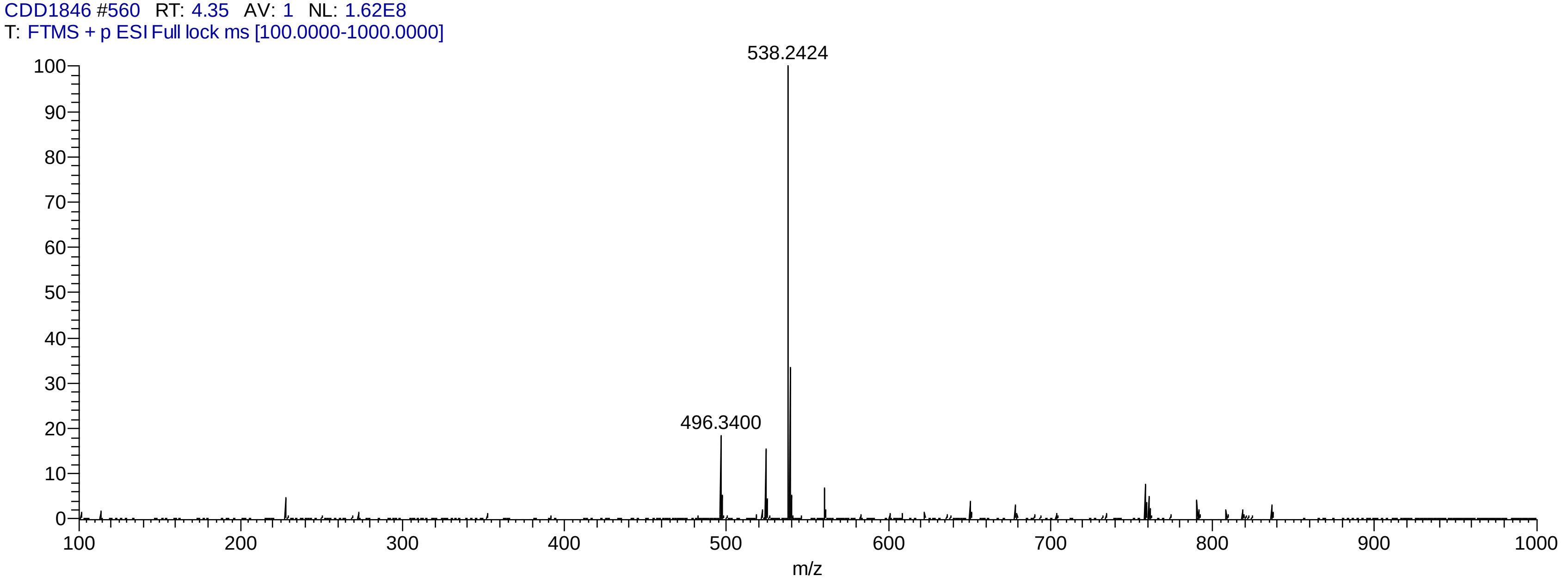

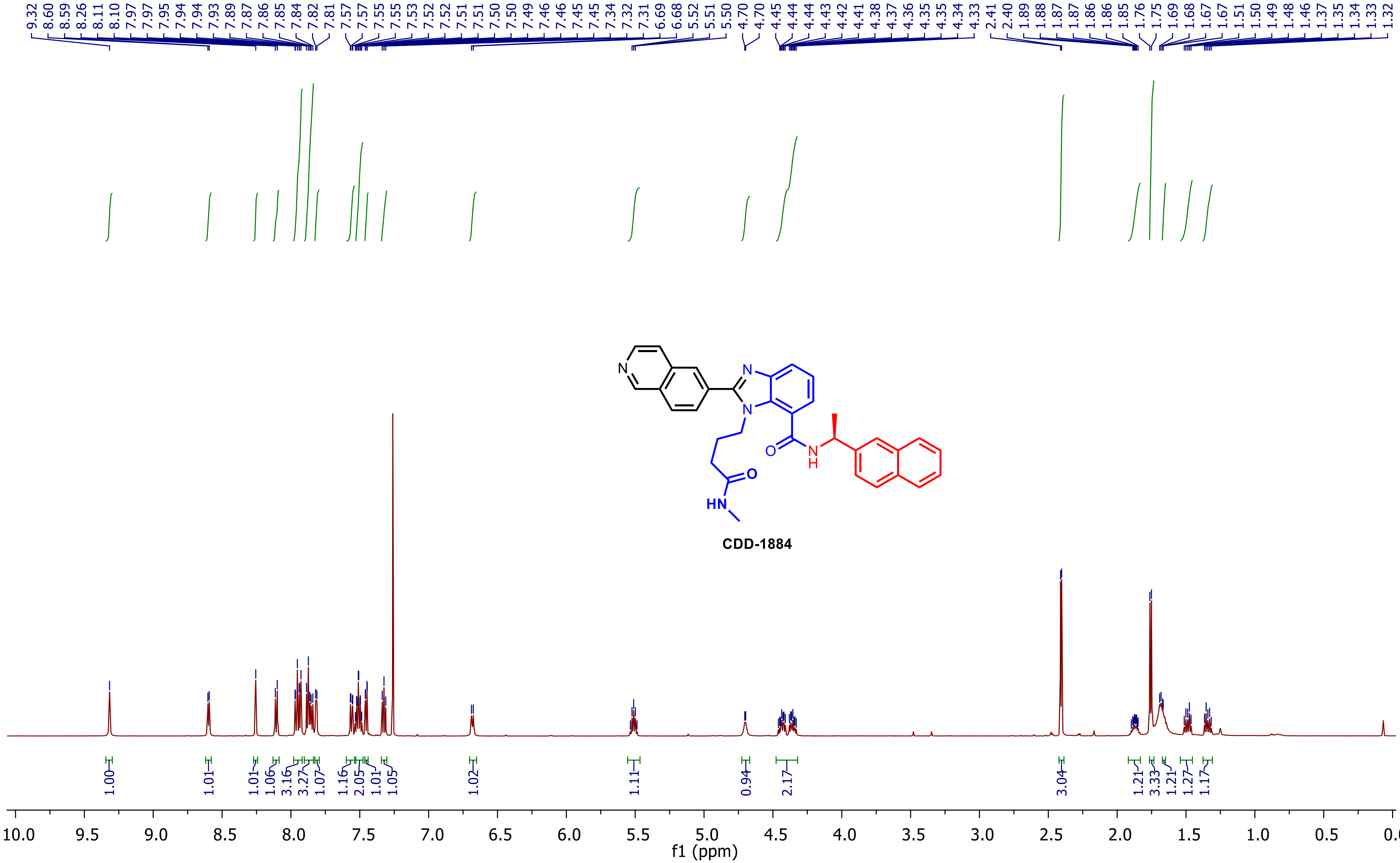
9.32 8.60 8.59 8.26 8.11 8.10 7.97 7.97 7.95 7.94 7.94 7.93 7.89 7.87 7.86 7.85 7.84 7.82 7.81 7.57 7.57 7.55 7.55 7.53 7.52 7.52 7.51 7.51 7.50 7.50 7.49 7.46 7.46 7.45 7.45 7.34 7.32 7.31 6.69 6.68 5.52 5.51 5.50 4.70 4.70 4.45 4.44 4.44 4.43 4.42 4.41 4.38 4.37 4.36 4.35 4.35 4.34 4.33 2.41 2.40 1.89 1.88 1.87 1.87 1.86 1.85 1.76 1.75 1.69 1.68 1.67 1.67 1.51 1.50 1.49 1.48 1.46 1.37 1.35 1.34 1.33 1.32
CDD-1884
1.00 1.01 1.01 1.06 3.16 3.27 1.07 1.16 2.05 1.01 1.05 1.02 1.11 0.94 2.17 3.04 1.21 3.33 1.21 1.27 1.17
10.0 9.5 9.0 8.5 8.0 7.5 7.0 6.5 6.0 5.5 5.0 4.5 4.0 3.5 3.0 2.5 2.0 1.5 1.0 0.5 0.0
f1 (ppm)

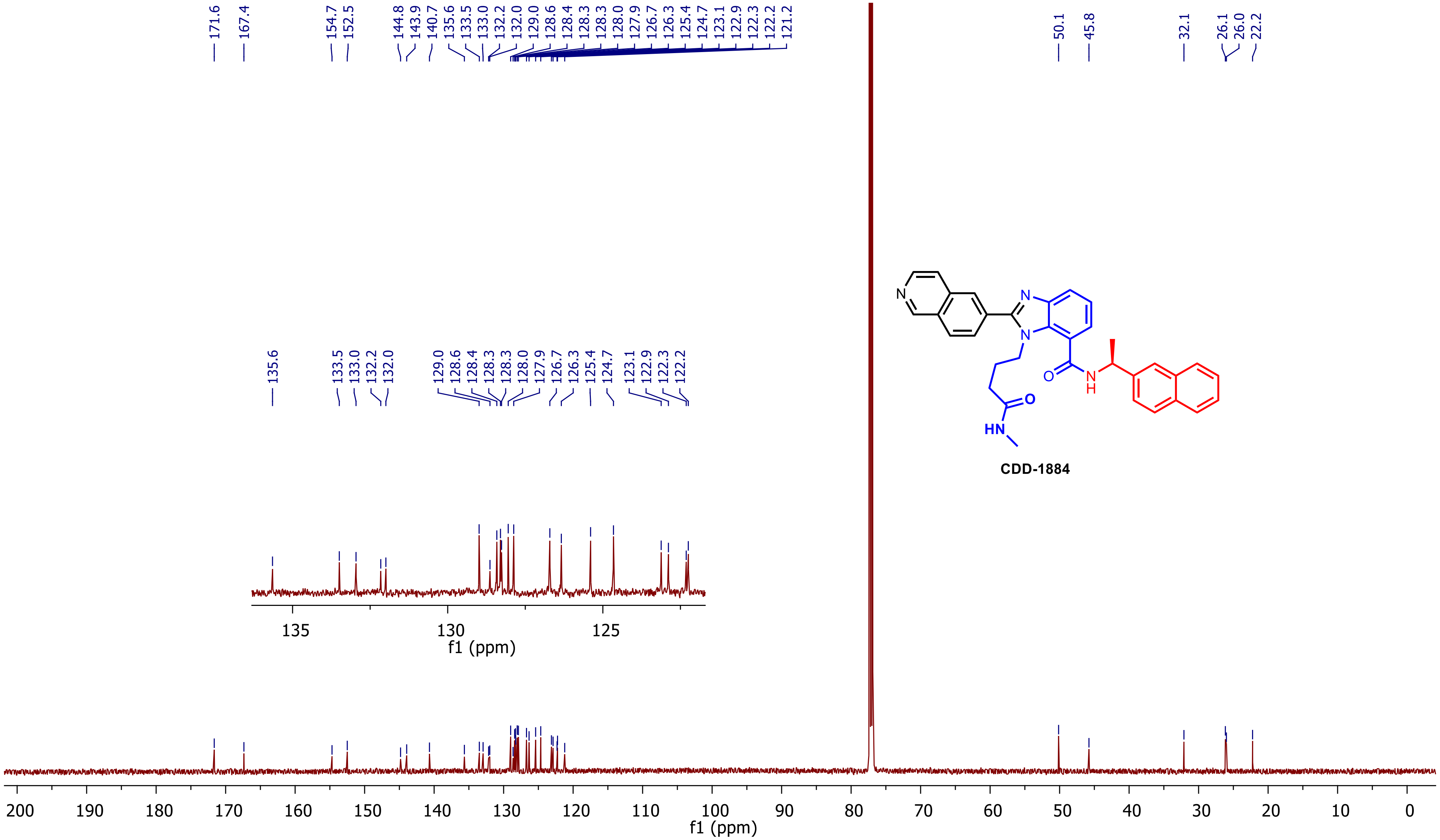
171.6
167.4
154.7
152.5
144.8
143.9
140.7
135.6
133.5
133.0
132.2
132.0
129.0
128.6
128.4
128.3
128.3
128.0
127.9
126.7
126.3
125.4
124.7
123.1
122.9
122.3
122.2
121.2
50.1
45.8
32.1
26.1
26.0
22.2
N
N
N
O
N
H
O
HN
CDD-1884
135
130
125
f1 (ppm)
200 190 180 170 160 150 140 130 120 110 100 90 80 70 60 50 40 30 20 10 0
f1 (ppm)

C:\Analytical\...\04012021\CDD1884 04/01/21 16:25:24

RT: 0.00 - 7.00

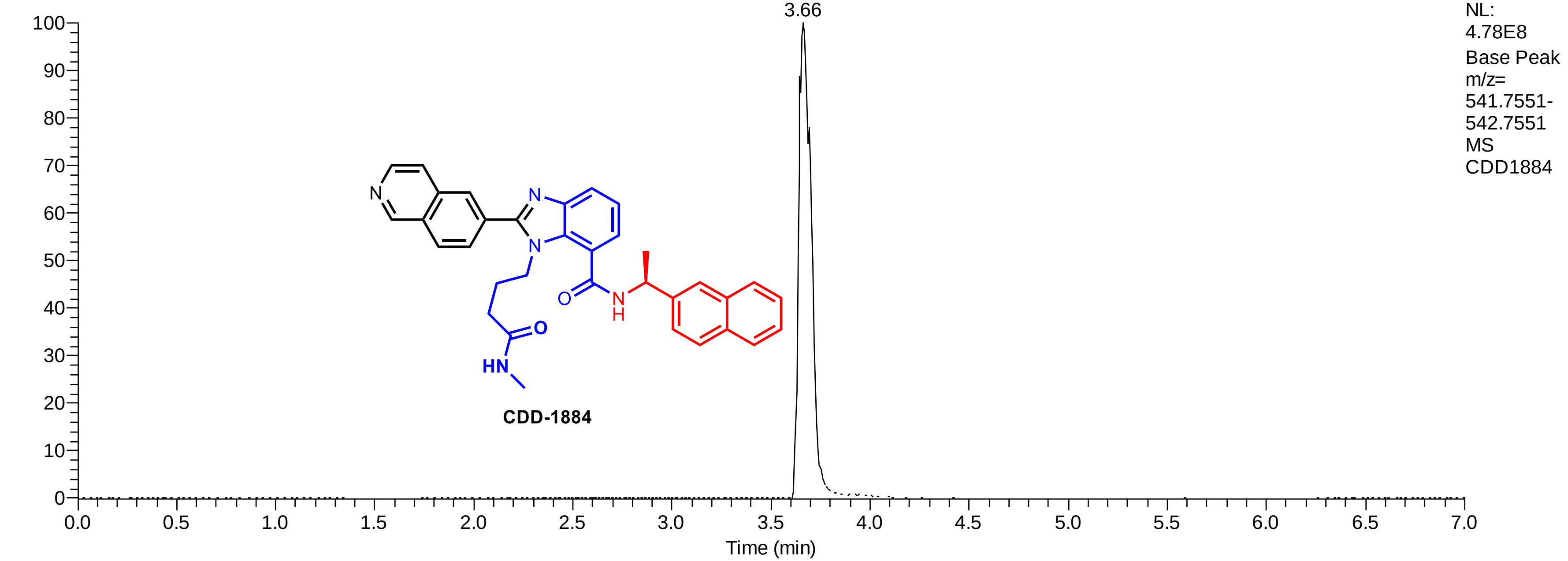

CDD1884 #472 RT: 3.66 AV: 1 NL: 6.51E8
T: FTMS + p ESI Full ms [100.0000-1000.0000]

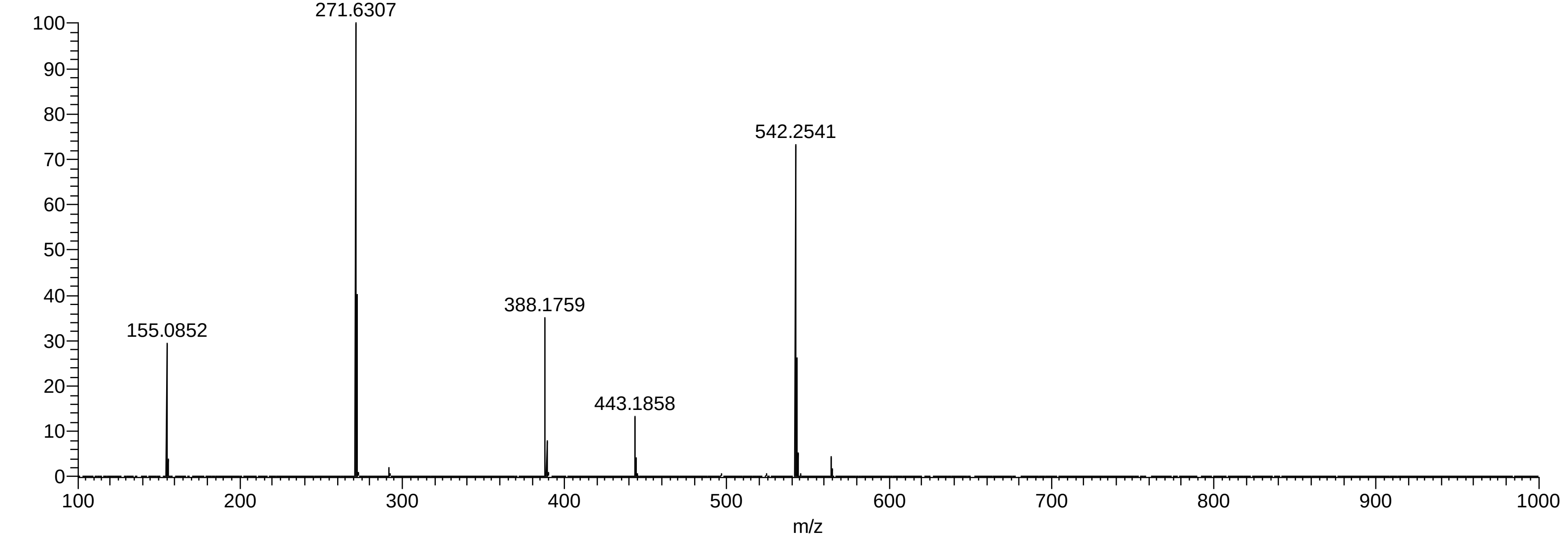

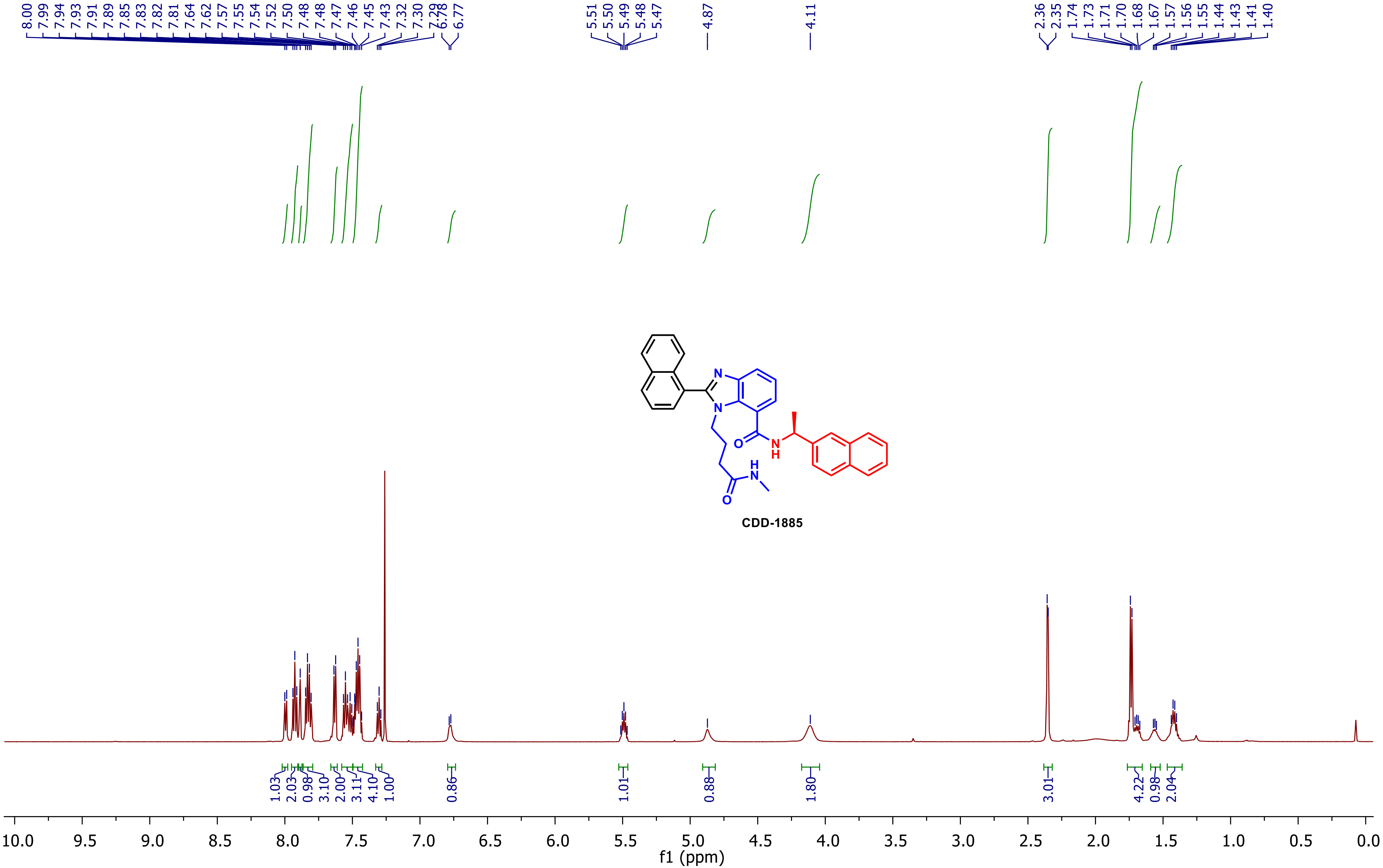
8.00
7.99
7.94
7.93
7.91
7.89
7.85
7.83
7.82
7.81
7.64
7.62
7.57
7.55
7.54
7.52
7.50
7.48
7.48
7.47
7.46
7.45
7.43
7.32
7.30
7.29
6.78
6.77
5.51
5.50
5.49
5.48
5.47
4.87
4.11
2.36
2.35
1.74
1.73
1.71
1.70
1.68
1.67
1.57
1.56
1.55
1.44
1.43
1.41
1.40
N
N
O
N
H
H
N
O
CDD-1885
1.03
2.03
0.98
3.10
2.00
3.11
4.10
1.00
0.86
1.01
0.88
1.80
3.01
4.22
0.98
2.04
10.0
9.5
9.0
8.5
8.0
7.5
7.0
6.5
6.0
5.5
5.0
4.5
4.0
3.5
3.0
2.5
2.0
1.5
1.0
0.5
0.0
f1 (ppm)

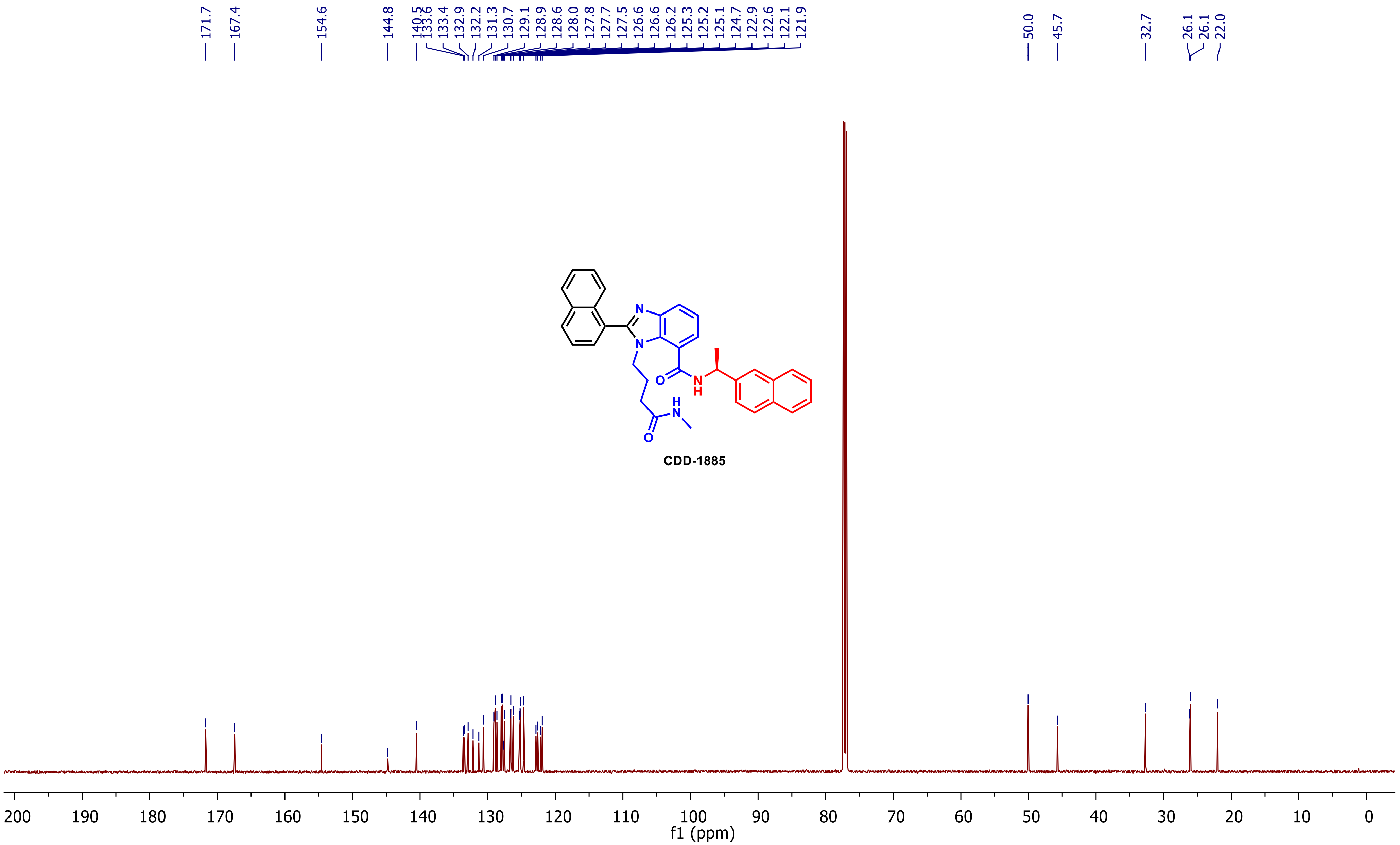
171.7
167.4
154.6
144.8
140.5
133.6
133.4
132.9
132.2
131.3
130.7
129.1
128.9
128.6
128.0
127.8
127.7
127.5
126.6
126.6
126.2
125.3
125.2
125.1
124.7
122.9
122.6
122.1
121.9
50.0
45.7
32.7
26.1
26.1
22.0
N
N
O
N
H
H
N
O
CDD-1885
200 190 180 170 160 150 140 130 120 110 100 90 80 70 60 50 40 30 20 10 0
f1 (ppm)

C:\Analytical\...\04012021\CDD1885 04/01/21 16:33:01

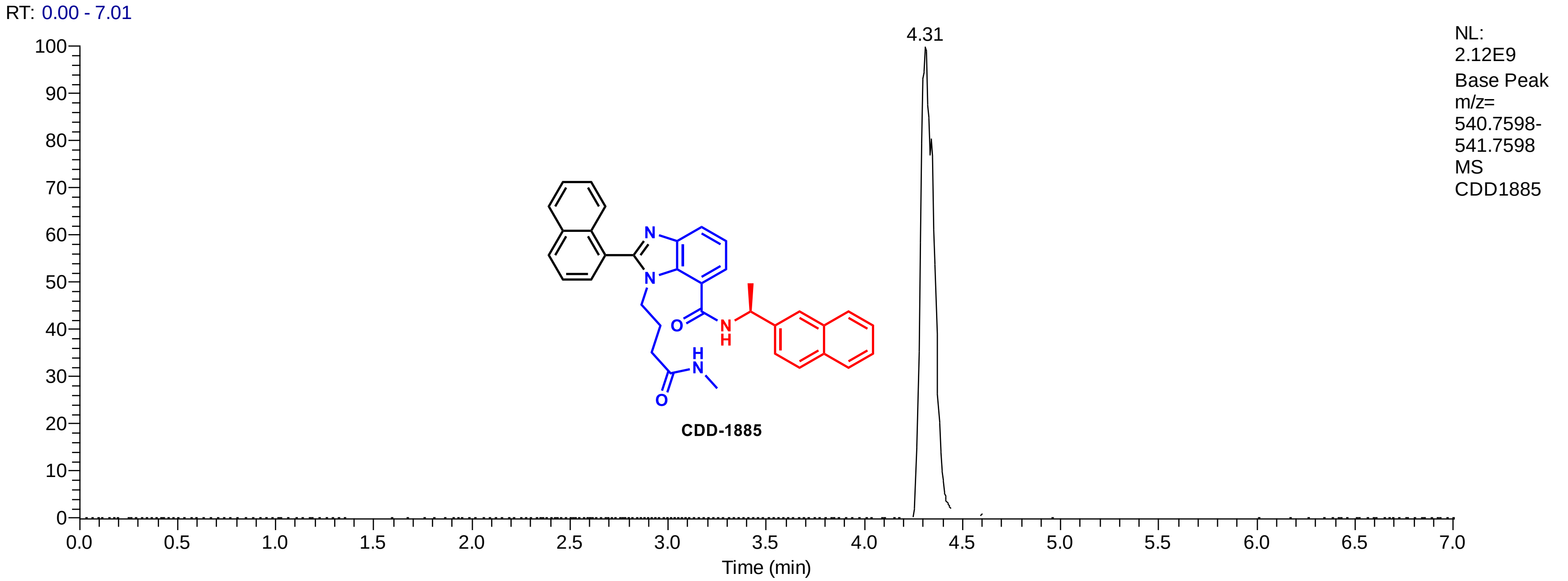

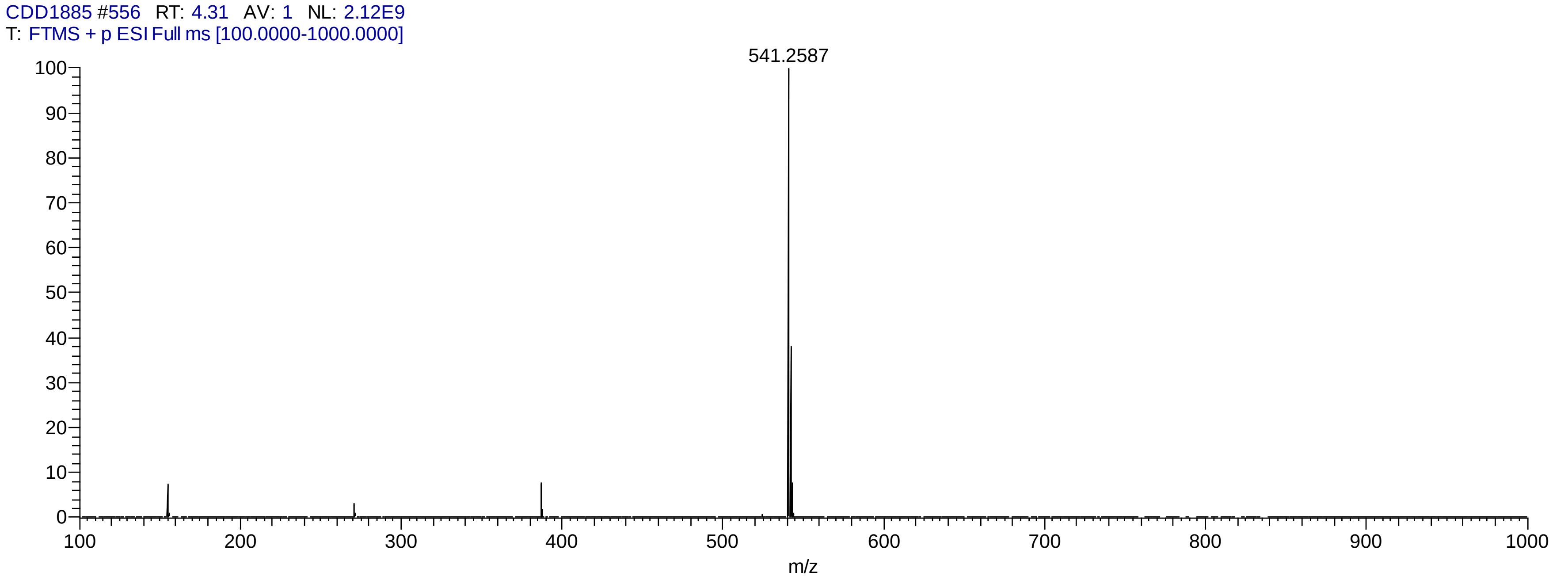

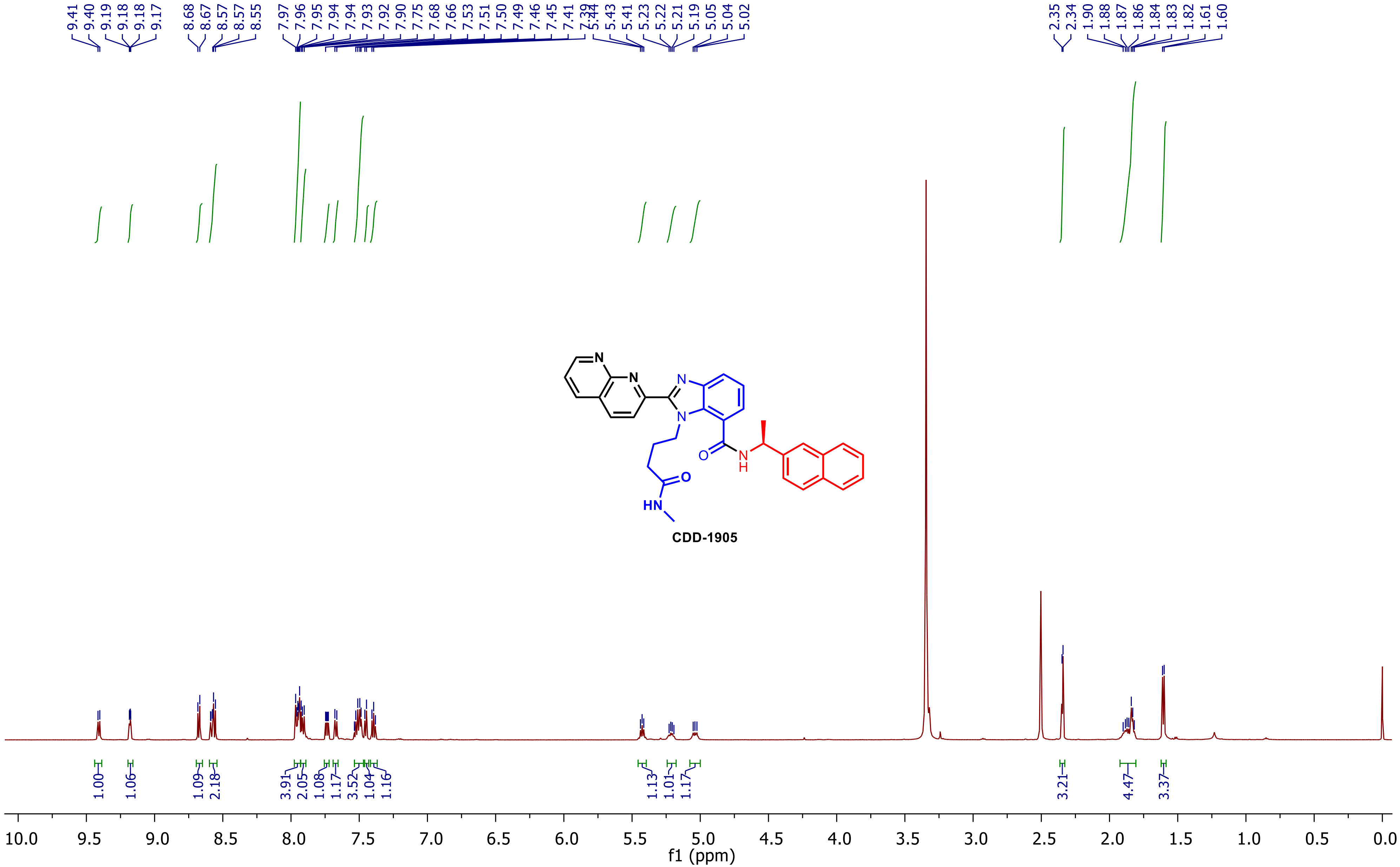
9.41
9.40
9.19
9.18
9.18
9.17
8.68
8.67
8.57
8.57
8.55
7.97
7.96
7.95
7.94
7.94
7.93
7.92
7.90
7.75
7.68
7.66
7.53
7.51
7.50
7.49
7.46
7.45
7.41
7.39
5.44
5.43
5.41
5.23
5.22
5.21
5.19
5.05
5.04
5.02
2.35
2.34
1.90
1.88
1.87
1.86
1.84
1.83
1.82
1.61
1.60
1.00
1.06
1.09
2.18
3.91
2.05
1.08
1.17
3.52
1.04
1.16
1.13
1.01
1.17
3.21
4.47
3.37
N
N
N
N
O
N
H
O
HN
CDD-1905
10.0
9.5
9.0
8.5
8.0
7.5
7.0
6.5
6.0
5.5
5.0
4.5
4.0
3.5
3.0
2.5
2.0
1.5
1.0
0.5
0.0
f1 (ppm)

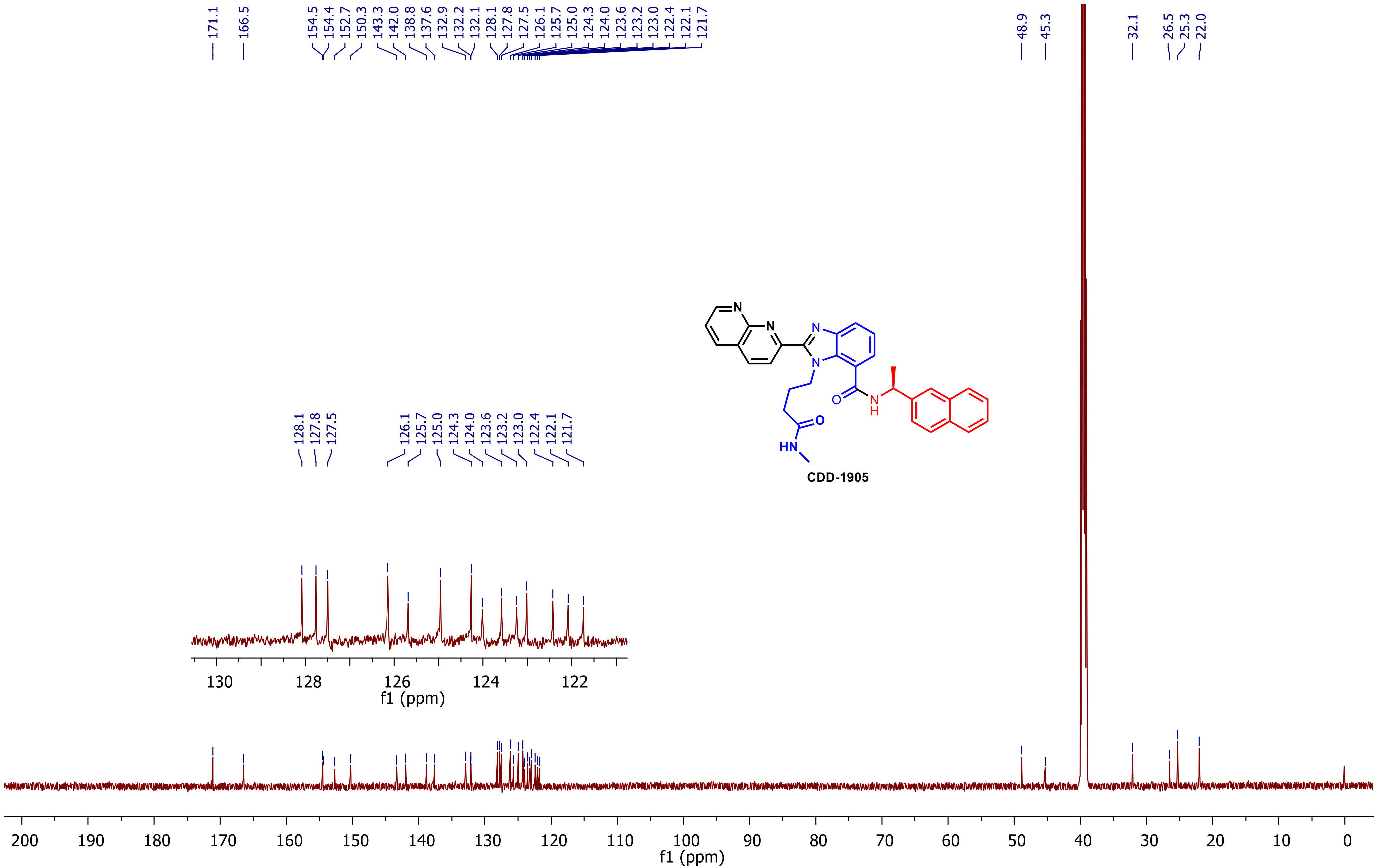
171.1
166.5
154.5
154.4
152.7
150.3
143.3
142.0
138.8
137.6
132.9
132.2
132.1
128.1
127.8
127.5
126.1
125.7
125.0
124.3
124.0
123.6
123.2
123.0
122.4
122.1
121.7
48.9
45.3
32.1
26.5
25.3
22.0
N
N
N
N
O
N
H
O
HN
CDD-1905
130
128
126
124
122
f1 (ppm)
200
190
180
170
160
150
140
130
120
110
100
90
80
70
60
50
40
30
20
10
0
f1 (ppm)

C:\Analytical\...\04012021\CDD1905 04/01/21 16:40:38

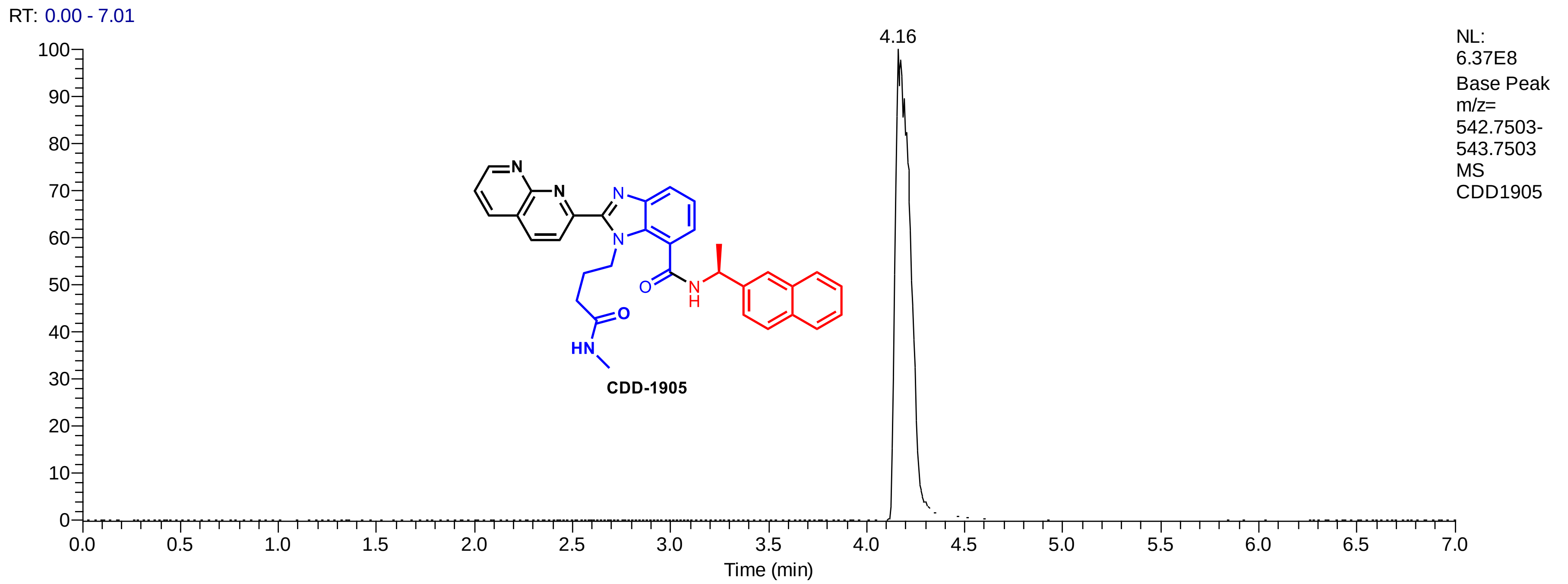

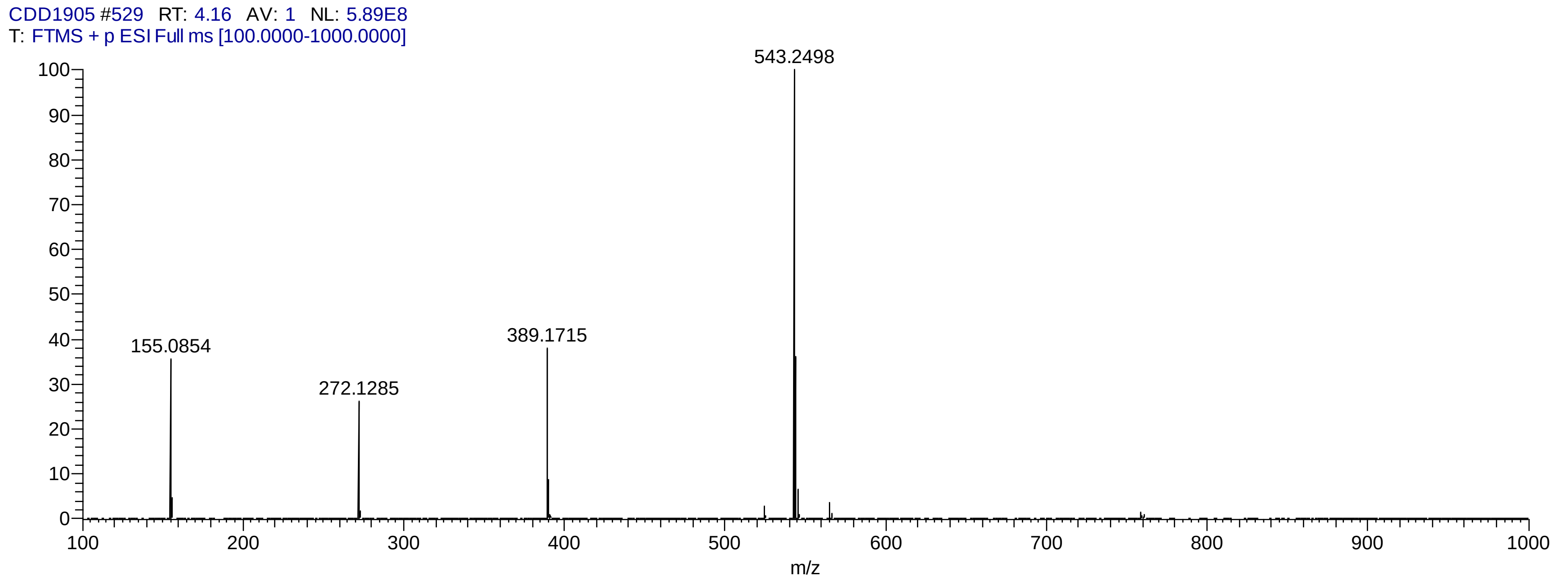

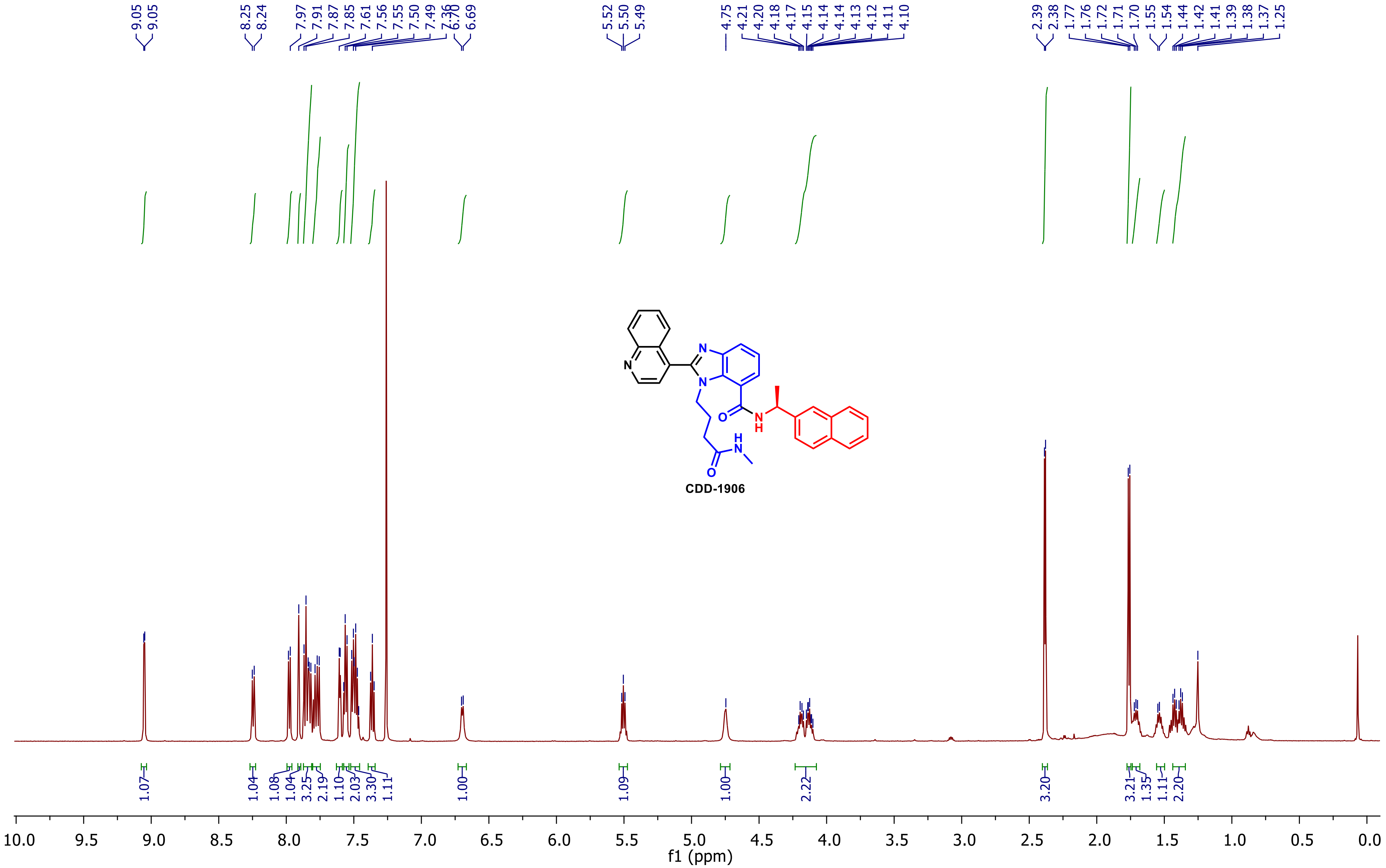
CDD-1906
f1 (ppm)

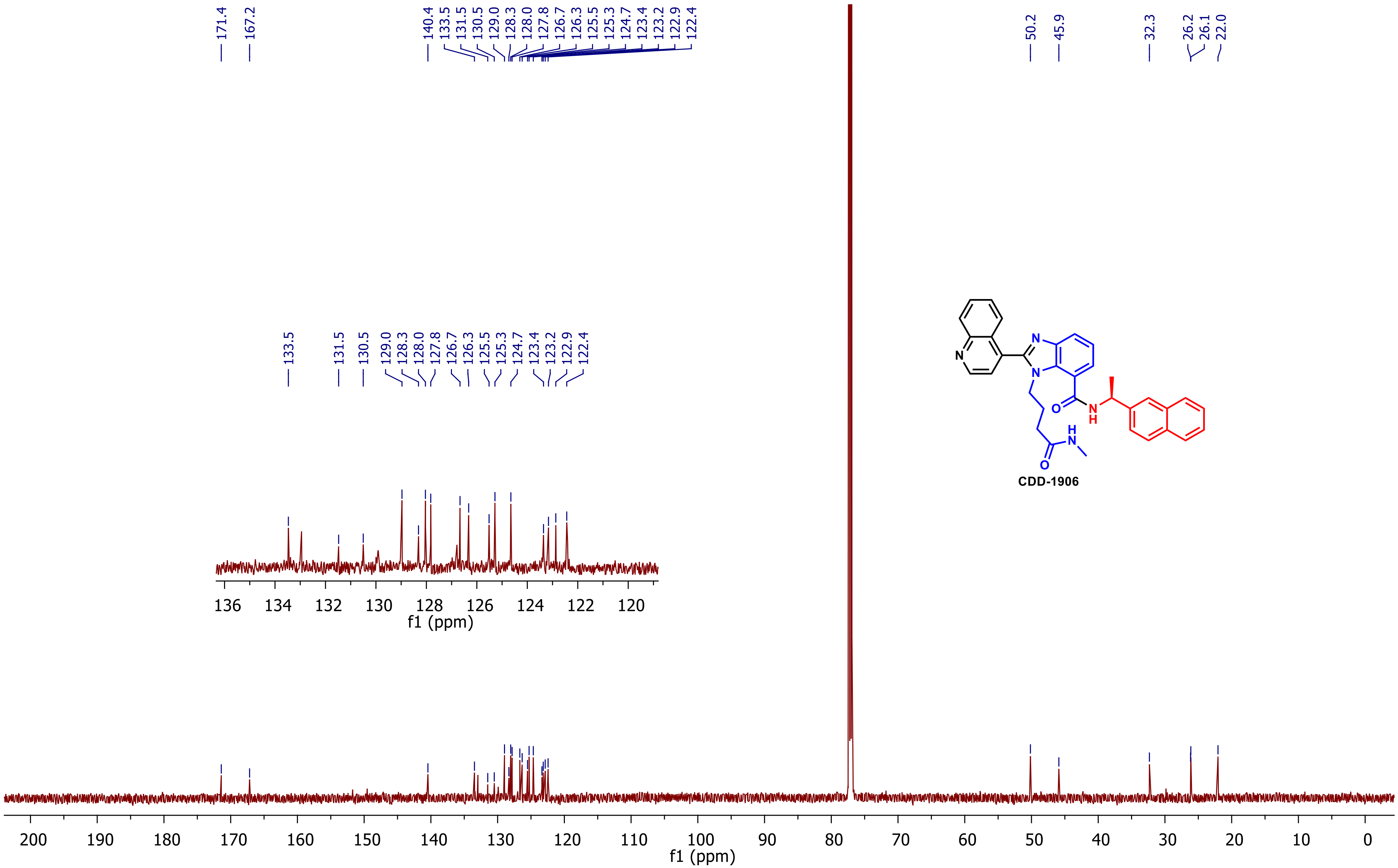
171.4
167.2
140.4
133.5
131.5
130.5
129.0
128.3
128.0
127.8
126.7
126.3
125.5
125.3
124.7
123.4
123.2
122.9
122.4
50.2
45.9
32.3
26.2
26.1
22.0
f1 (ppm)

CDD-1906

C:\Analytical\...\04012021\CDD1906 04/01/21 16:48:15

RT: 0.00 - 7.00

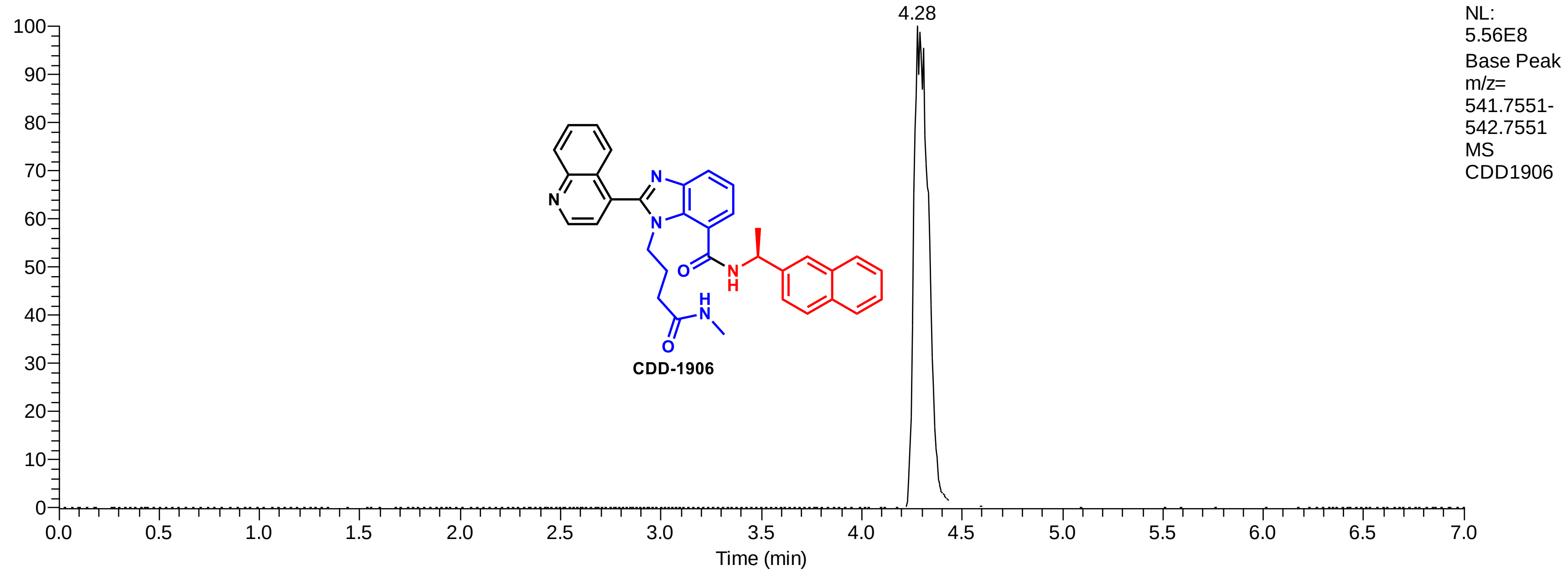

CDD1906 #542 RT: 4.28 AV: 1 NL: 4.99E8
T: FTMS + p ESI Full ms [100.0000-1000.0000]

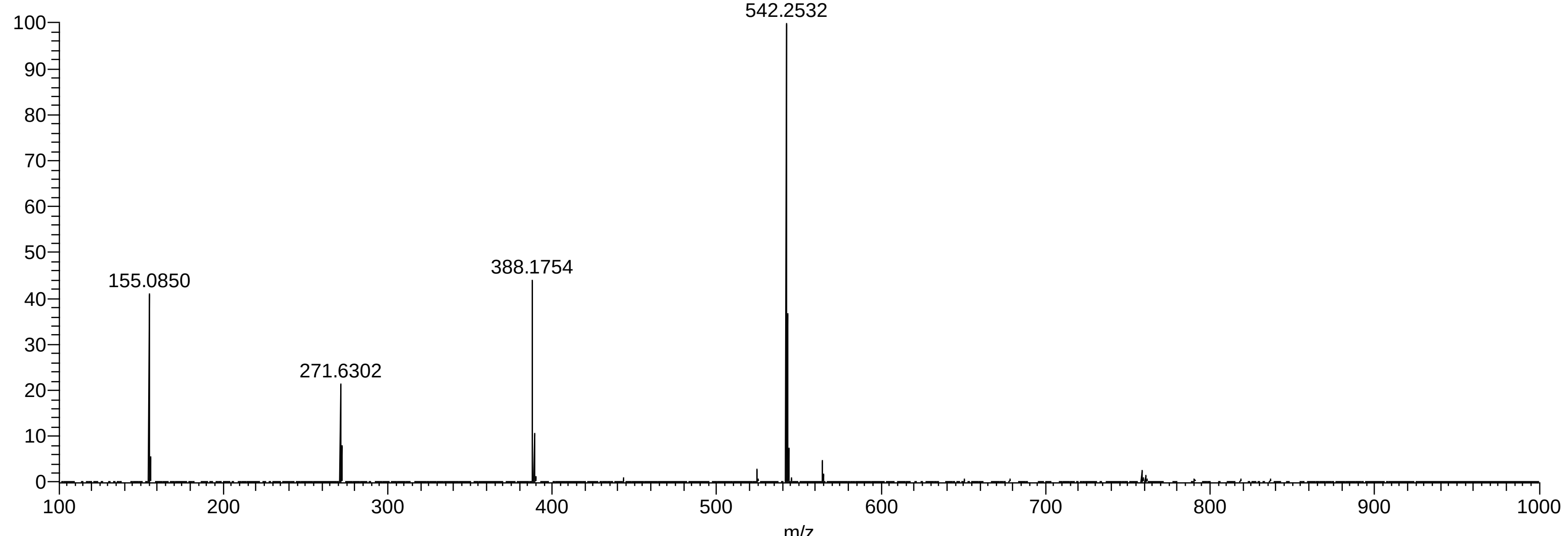

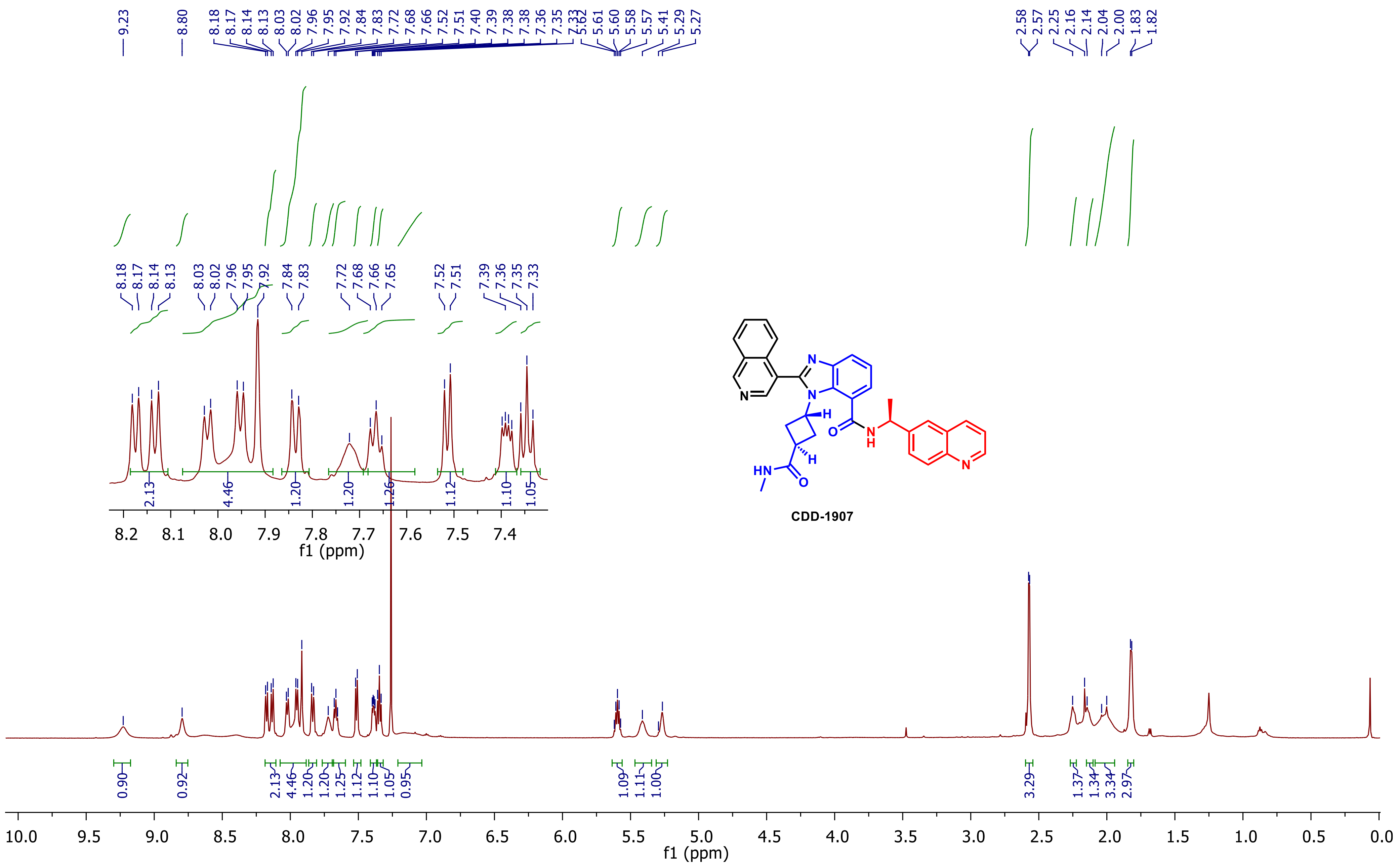

9.23
8.80
8.18
8.17
8.14
8.13
8.03
8.02
7.96
7.95
7.92
7.84
7.83
7.72
7.68
7.66
7.52
7.51
7.40
7.39
7.38
7.38
7.36
7.35
7.33
5.62
5.61
5.60
5.58
5.57
5.41
5.29
5.27
2.58
2.57
2.25
2.16
2.14
2.04
2.00
1.83
1.82
f1 (ppm)
N
N
N
H
H
O
N
H
HN
O
N

CDD-1907

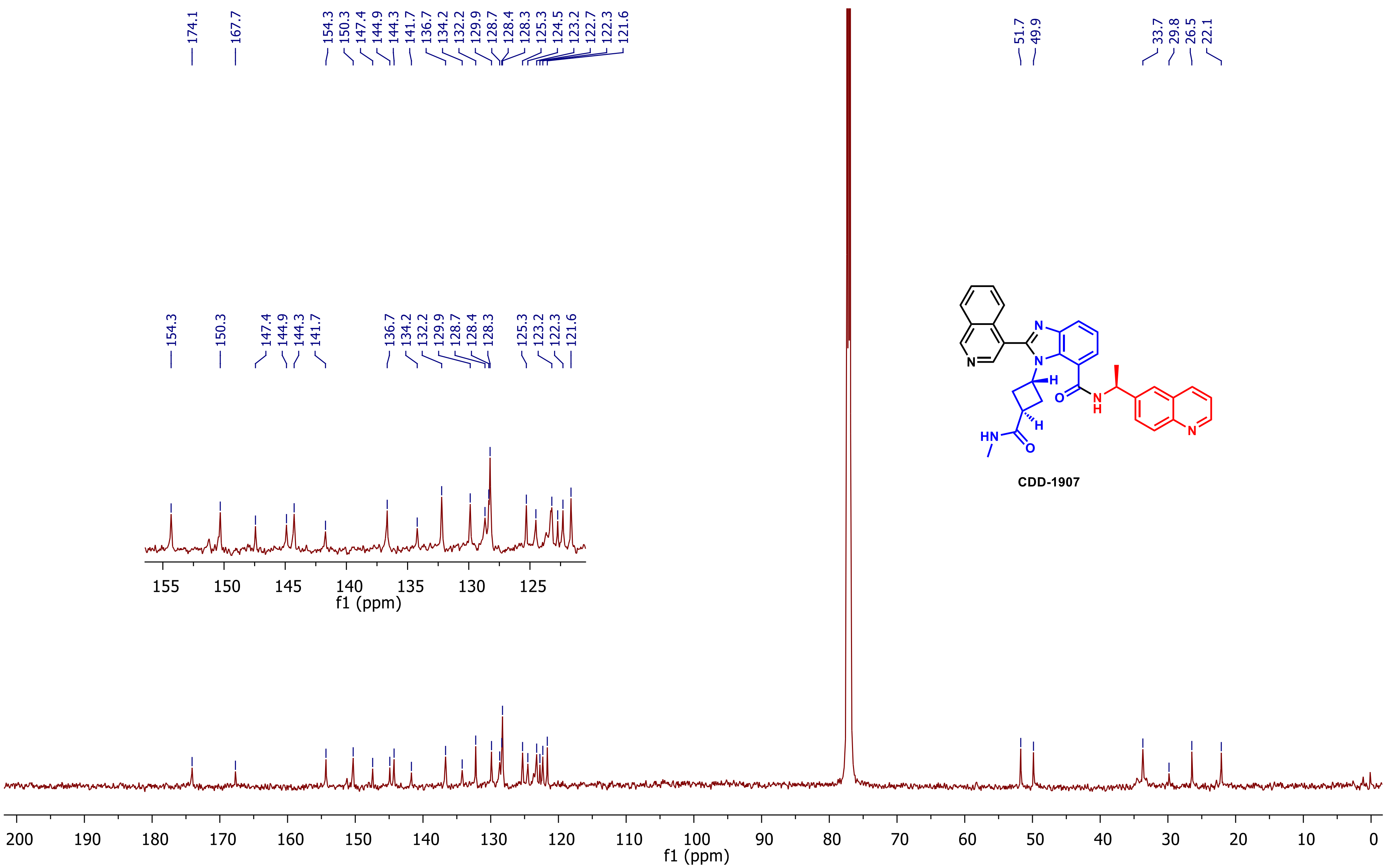
174.1
167.7
154.3
150.3
147.4
144.9
144.3
141.7
136.7
134.2
132.2
129.9
128.7
128.4
128.3
125.3
124.5
123.2
122.7
122.3
121.6
51.7
49.9
33.7
29.8
26.5
22.1
f1 (ppm)
CDD-1907

C:\Analytical\...\04012021\CDD1907 04/01/21 16:55:51

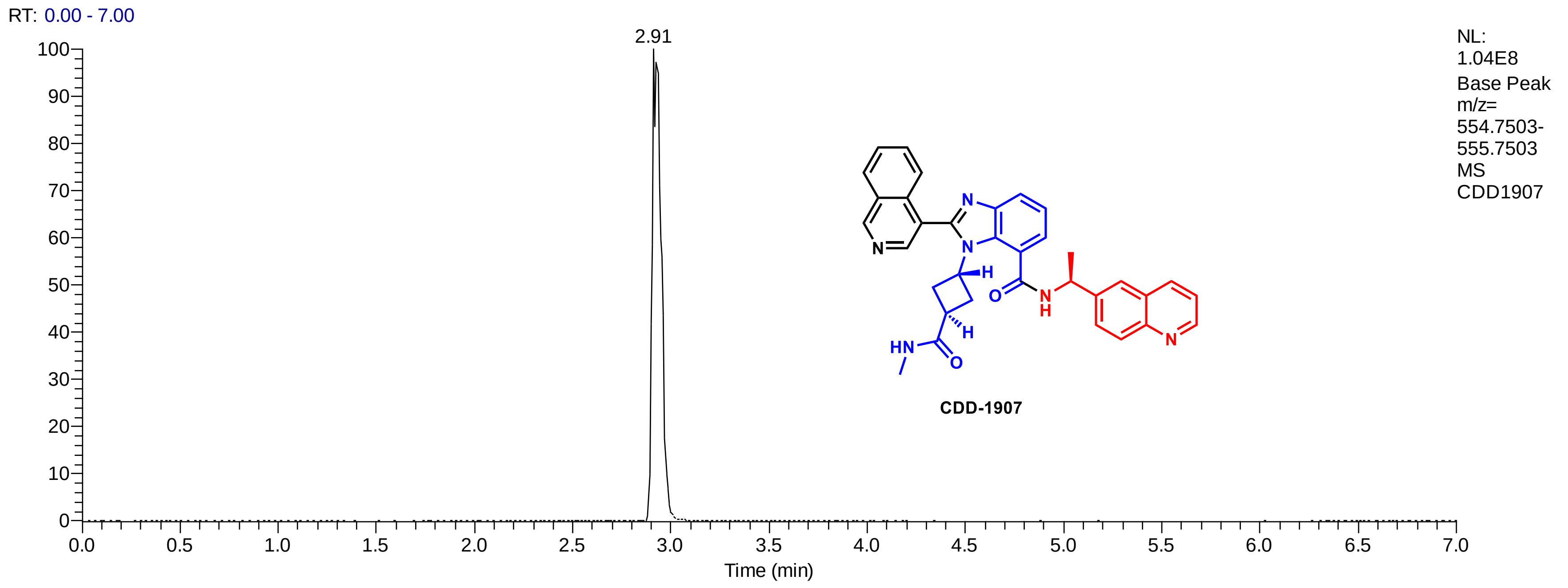

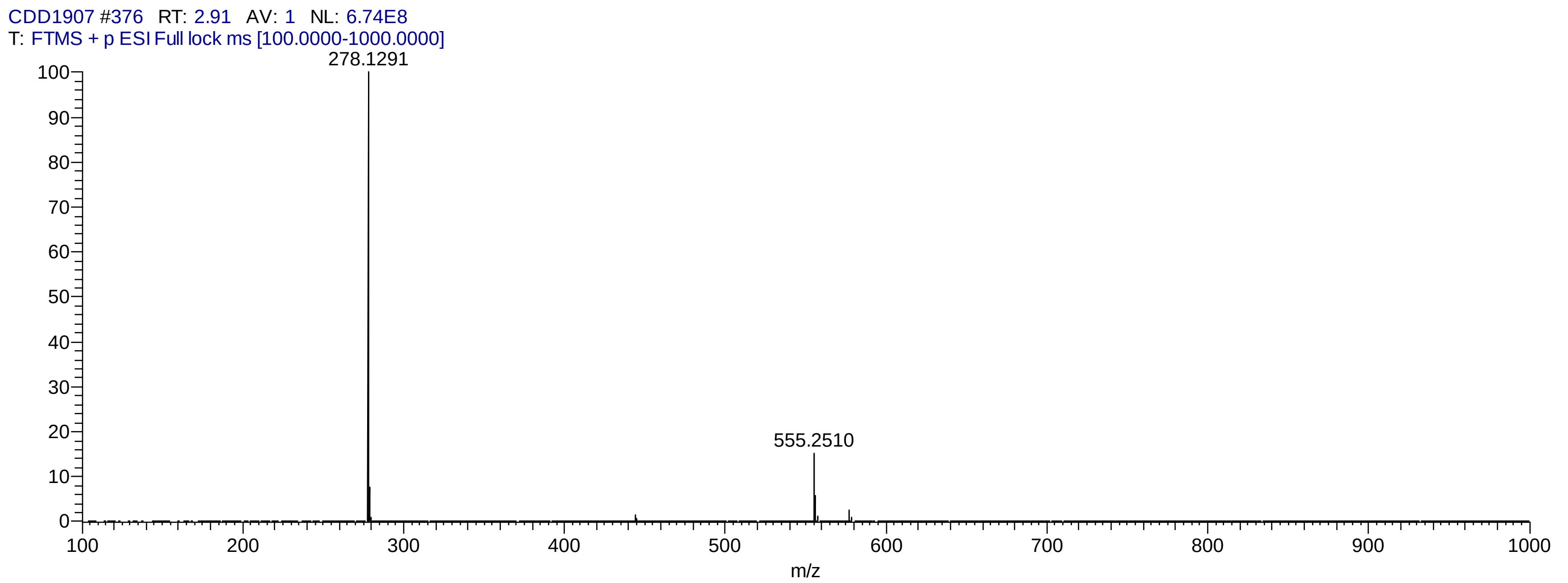

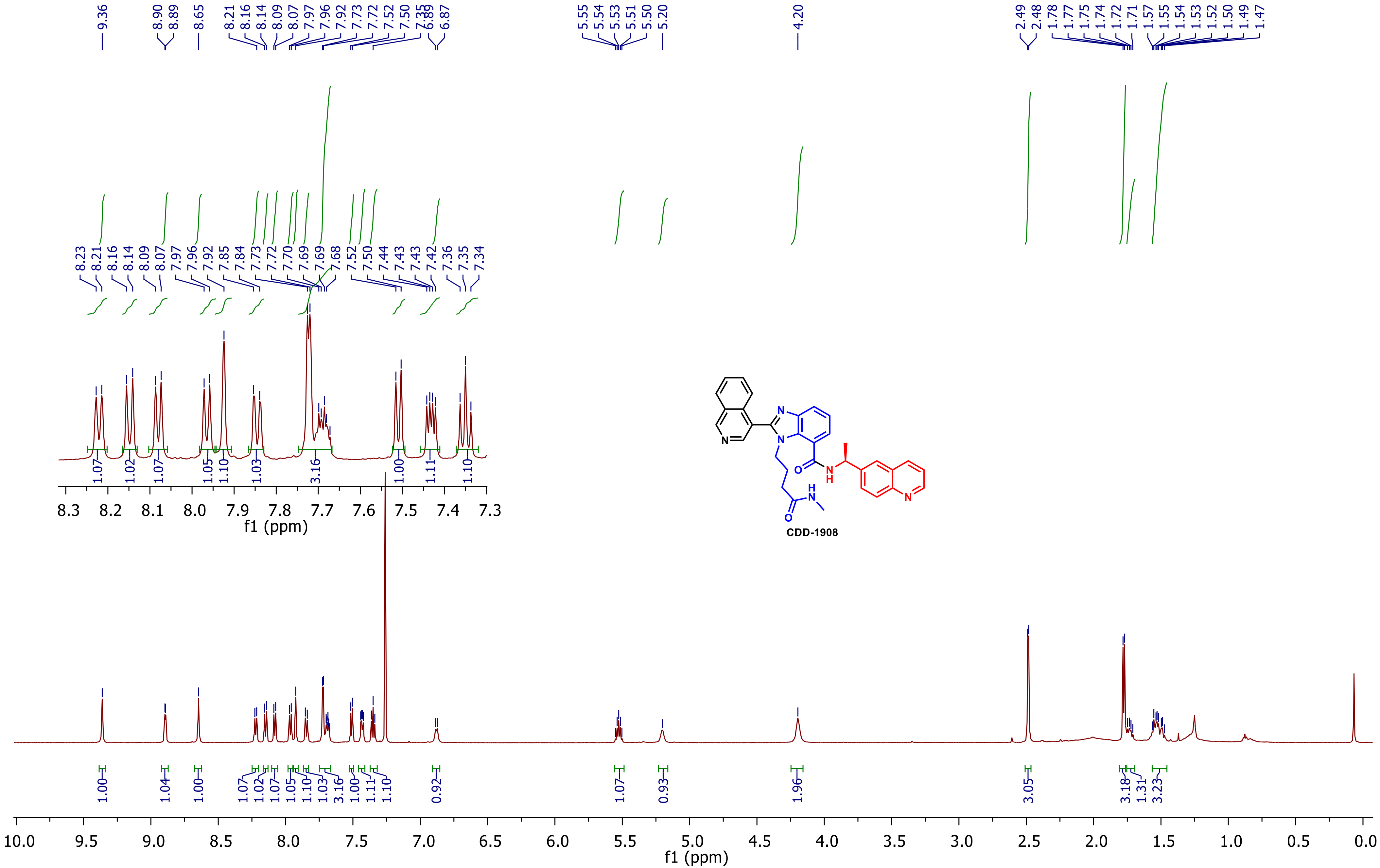
CDD-1908
f1 (ppm)
f1 (ppm)

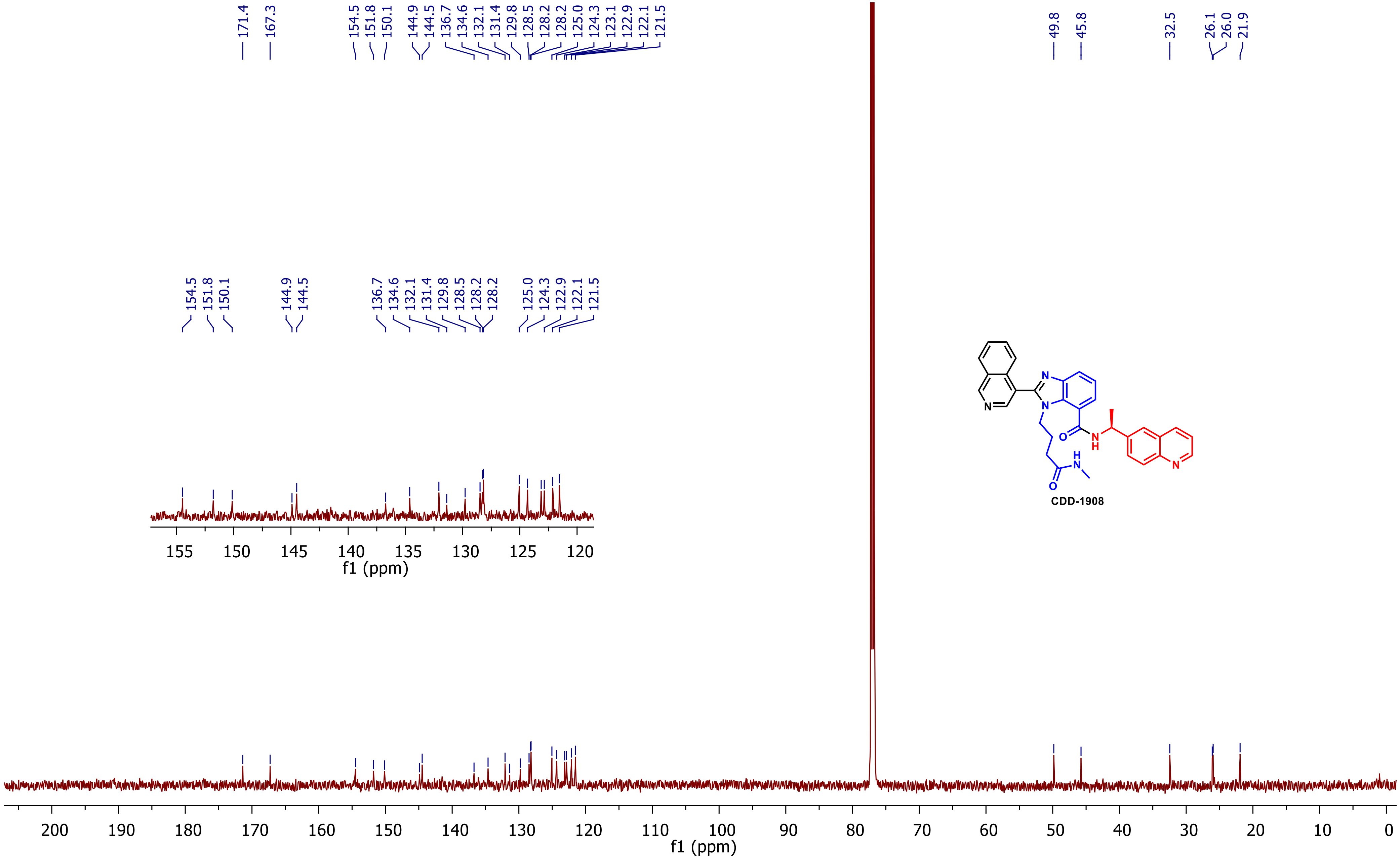
171.4
167.3
154.5
151.8
150.1
144.9
144.5
136.7
134.6
132.1
131.4
129.8
128.5
128.2
128.2
125.0
124.3
123.1
122.9
122.1
121.5
49.8
45.8
32.5
26.1
26.0
21.9
f1 (ppm)
f1 (ppm)
CDD-1908

C:\Analytical\...\04012021\CDD1908 04/01/21 17:03:28

RT: 0.00 - 7.01

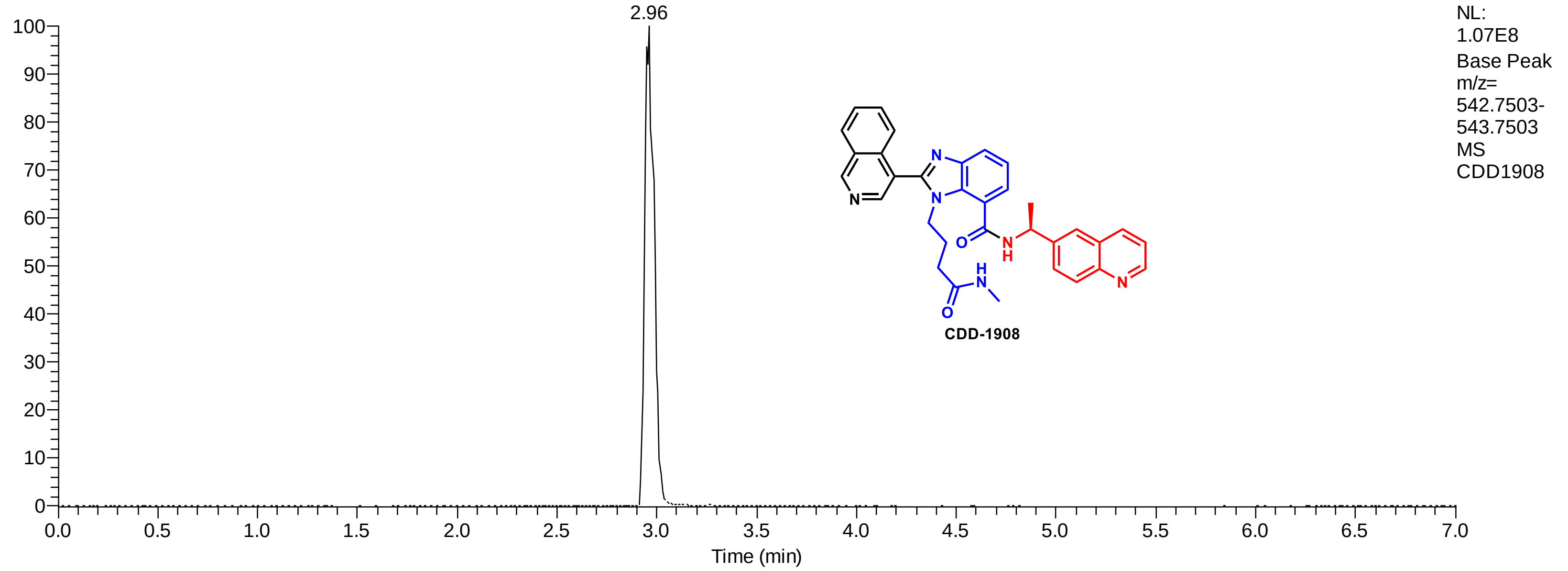

CDD1908 #372 RT: 2.96 AV: 1 NL: 5.64E8
T: FTMS + p ESI Full lock ms [100.0000-1000.0000]

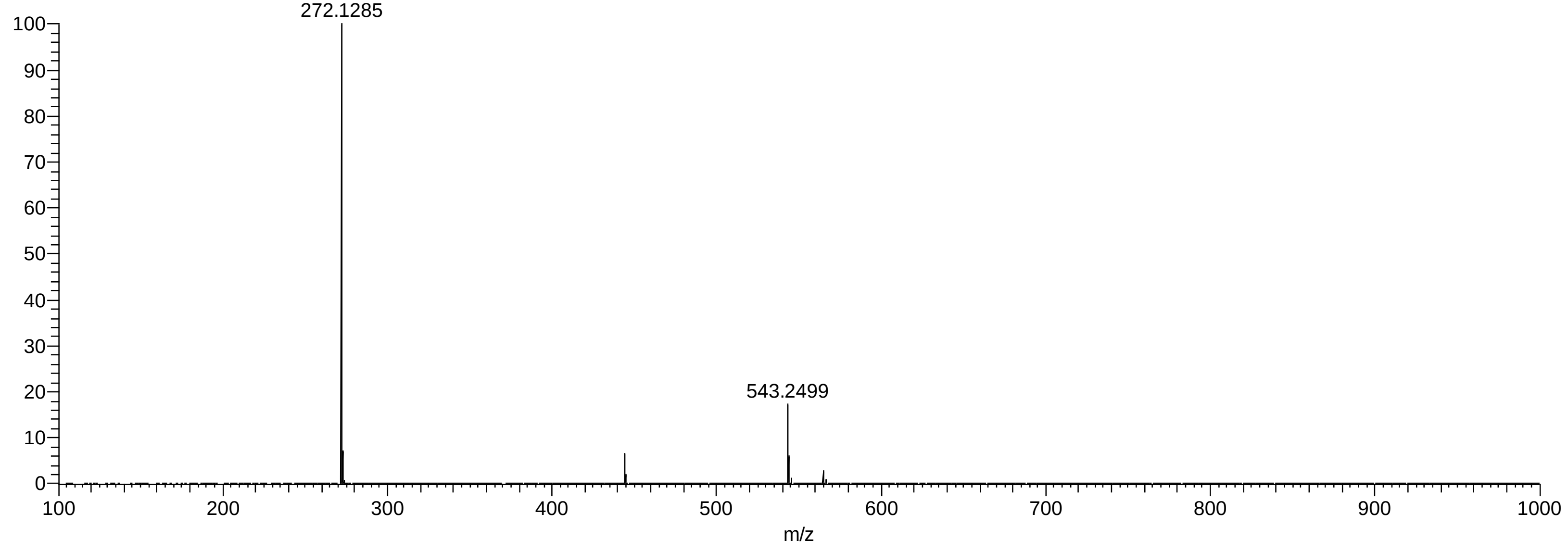

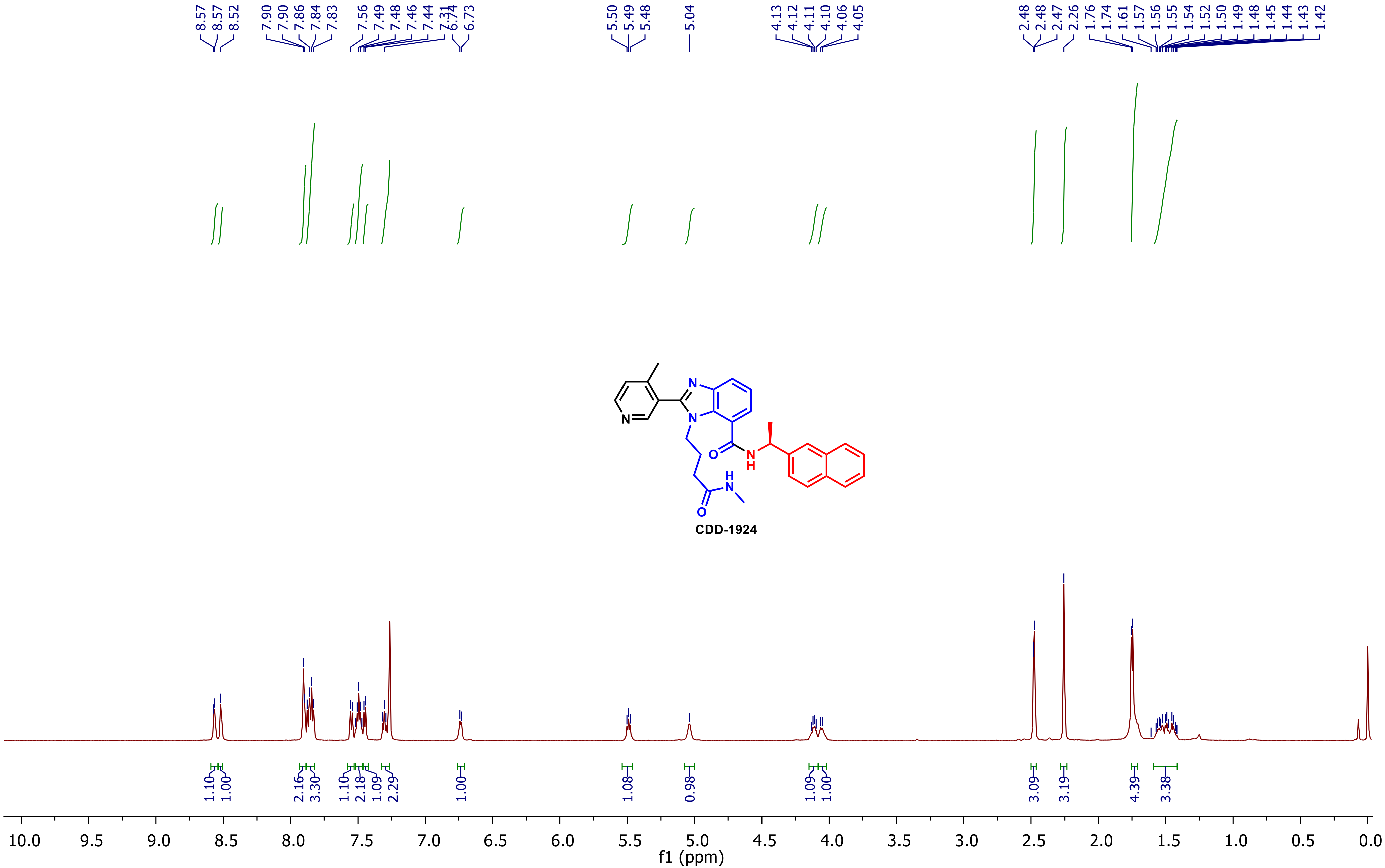
8.57
8.57
8.52
7.90
7.90
7.86
7.84
7.83
7.56
7.49
7.48
7.46
7.44
7.31
6.74
6.73
5.50
5.49
5.48
5.04
4.13
4.12
4.11
4.10
4.06
4.05
2.48
2.48
2.47
2.26
1.76
1.74
1.61
1.57
1.56
1.55
1.54
1.52
1.50
1.49
1.48
1.45
1.44
1.43
1.42
CDD-1924
1.10
1.00
2.16
3.30
1.10
2.18
1.09
2.29
1.00
1.08
0.98
1.09
1.00
3.09
3.19
4.39
3.38
10.0
9.5
9.0
8.5
8.0
7.5
7.0
6.5
6.0
5.5
5.0
4.5
4.0
3.5
3.0
2.5
2.0
1.5
1.0
0.5
0.0
f1 (ppm)

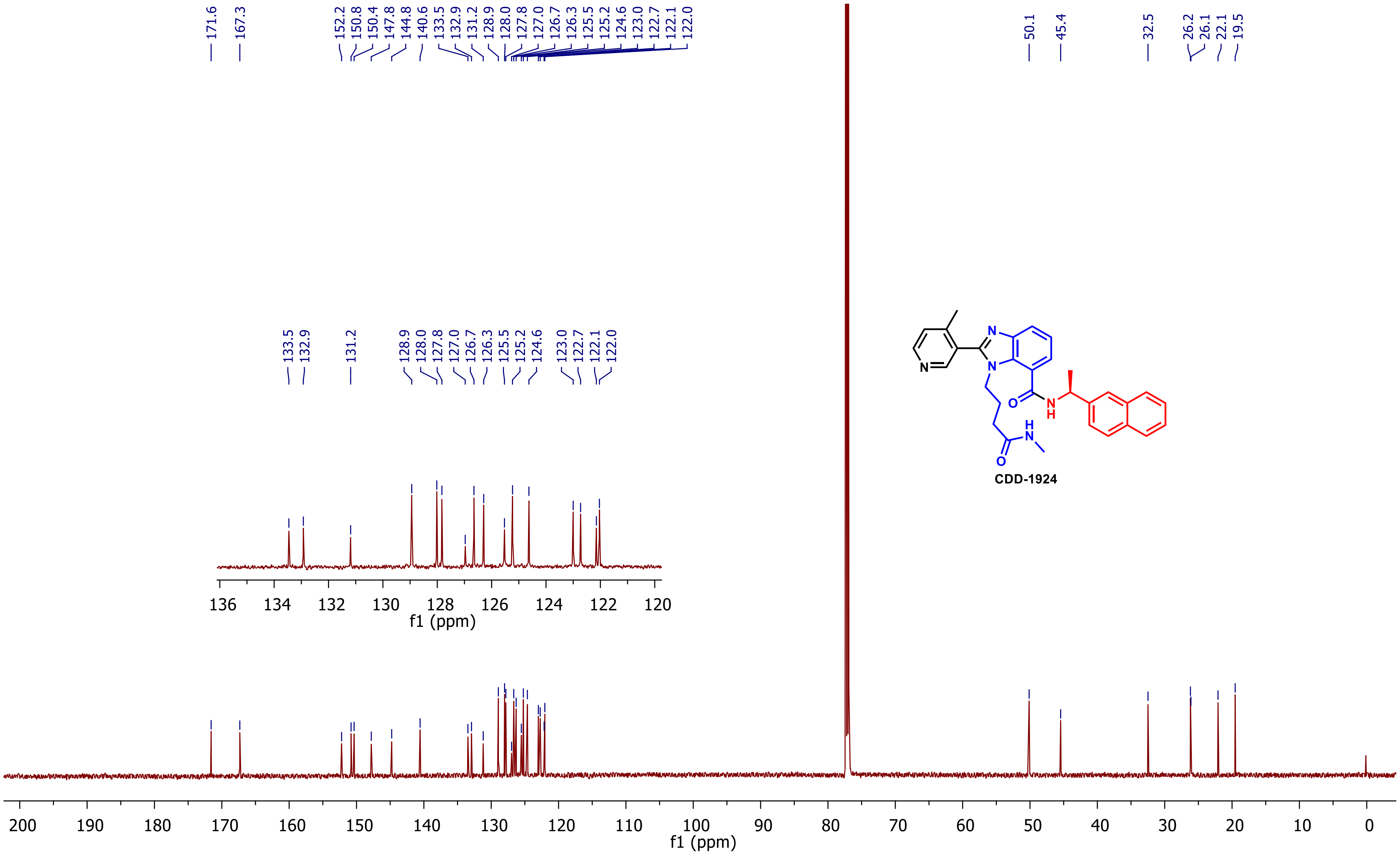
171.6
167.3
152.2
150.8
150.4
147.8
144.8
140.6
133.5
132.9
131.2
128.9
128.0
127.8
127.0
126.7
126.3
125.5
125.2
124.6
123.0
122.7
122.1
122.0
50.1
45.4
32.5
26.2
26.1
22.1
19.5
136 134 132 130 128 126 124 122 120
f1 (ppm)
200 190 180 170 160 150 140 130 120 110 100 90 80 70 60 50 40 30 20 10 0
f1 (ppm)
CDD-1924

C:\Analytical\...\04012021\CDD1924 04/01/21 17:11:05

RT: 0.00 - 7.01

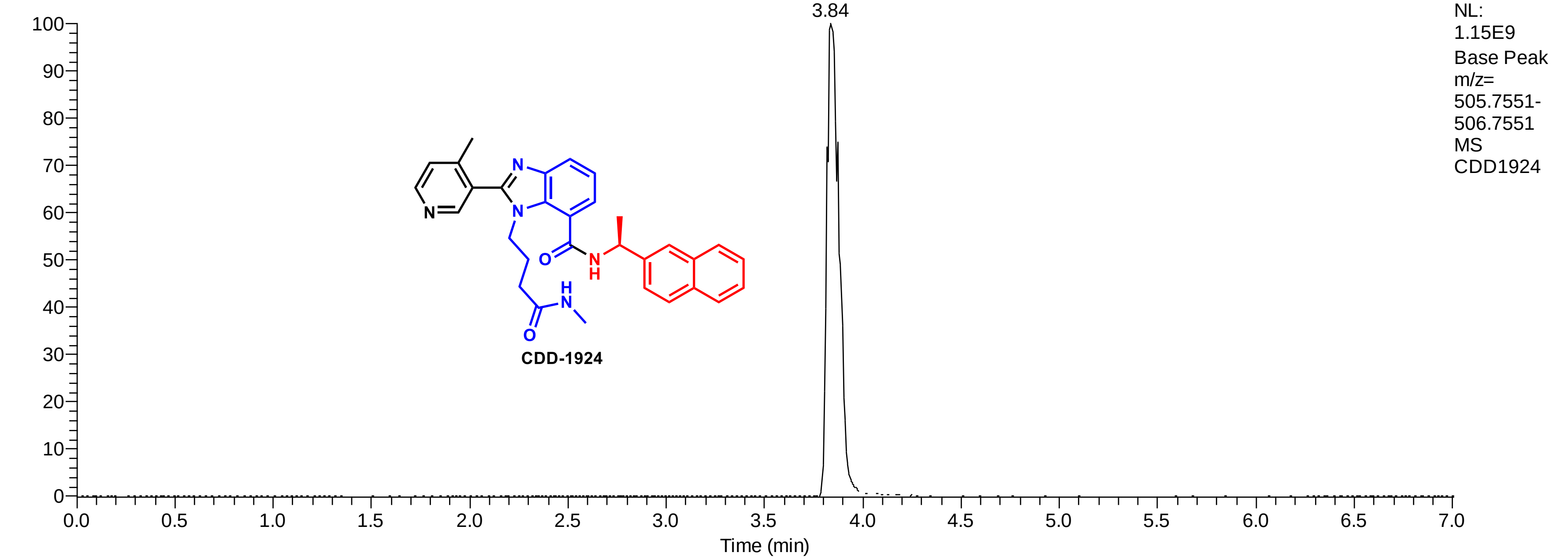

CDD1924 #480 RT: 3.84 AV: 1 NL: 1.12E9
T: FTMS + p ESI Full lock ms [100.0000-1000.0000]

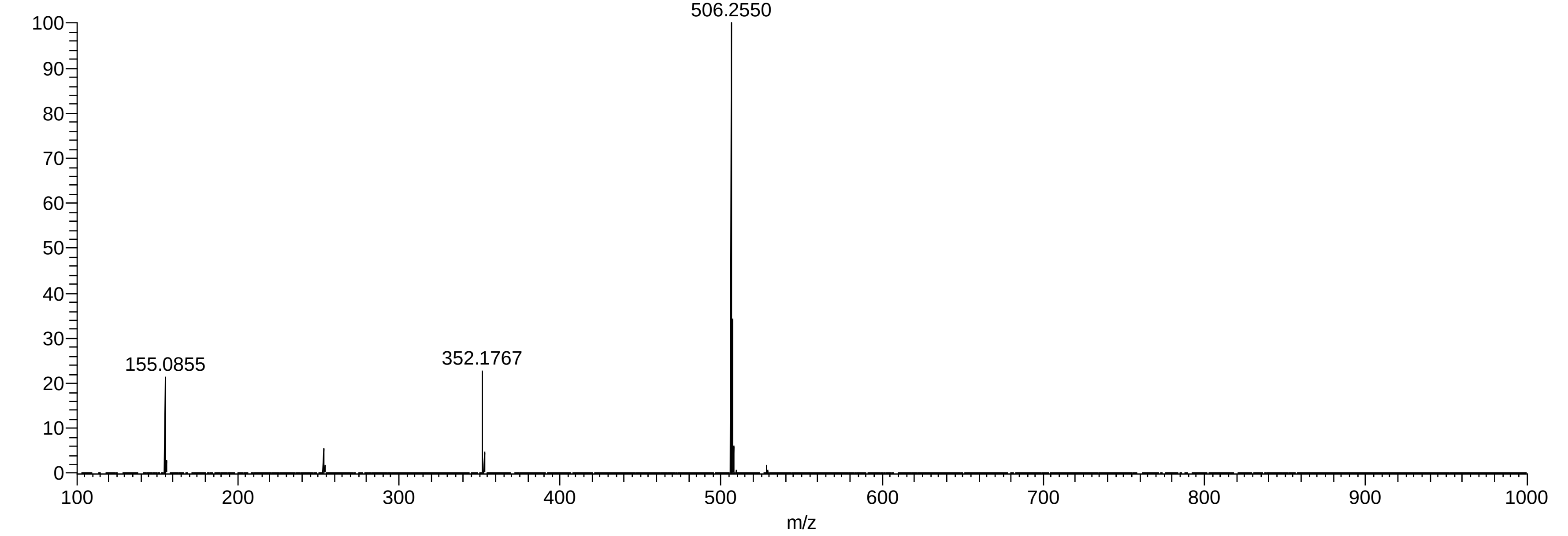

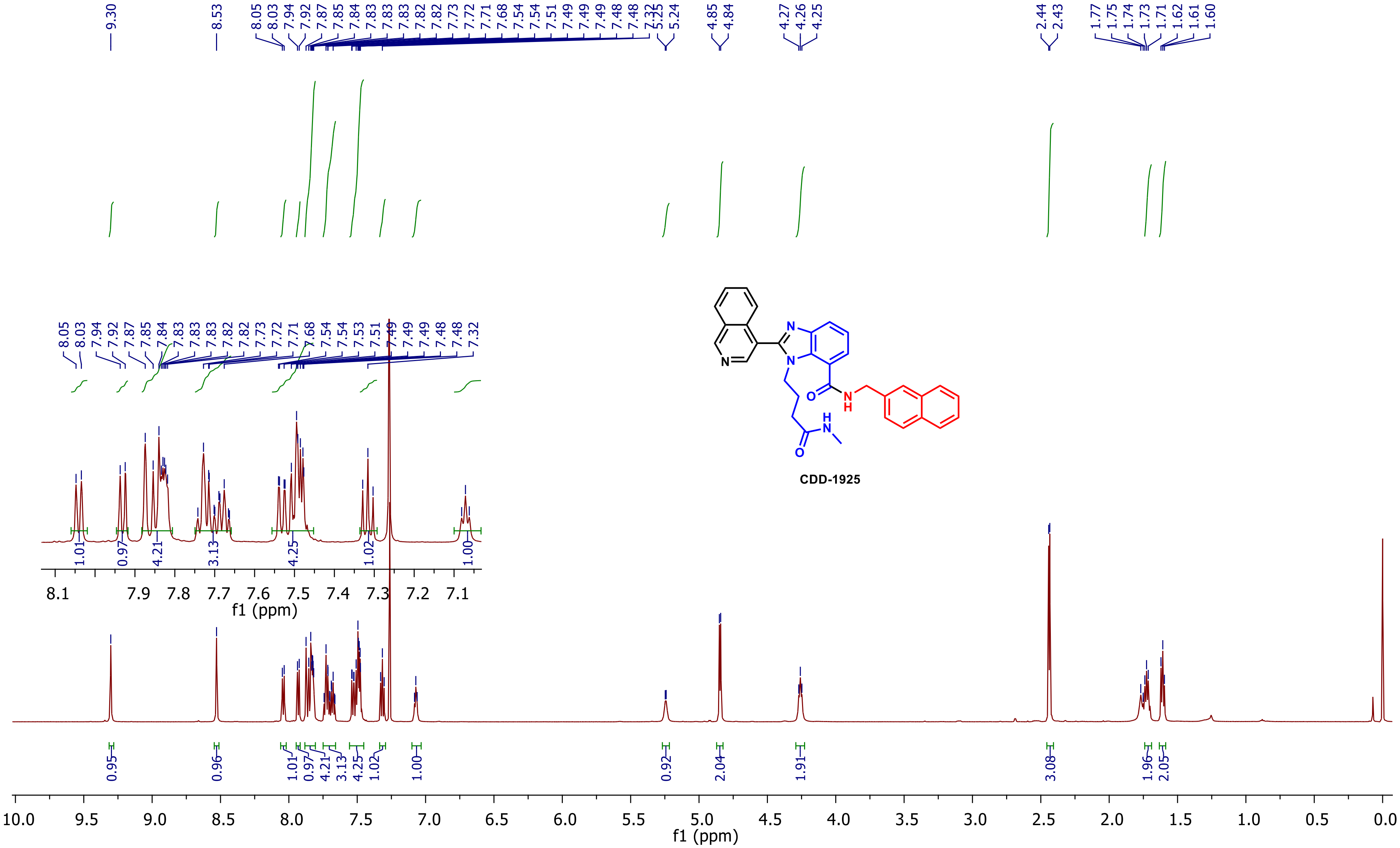
9.30
8.53
8.05 8.03 7.94 7.92 7.87 7.85 7.84 7.83 7.83 7.83 7.82 7.82 7.73 7.72 7.71 7.68 7.54 7.54 7.51 7.49 7.49 7.49 7.48 7.48 7.32
5.25 5.24
4.85 4.84
4.27 4.26 4.25
2.44 2.43
1.77 1.75 1.74 1.73 1.71 1.62 1.61 1.60
8.05 8.03 7.94 7.92 7.87 7.85 7.84 7.83 7.83 7.82 7.82 7.73 7.72 7.71 7.68 7.54 7.54 7.53 7.51 7.49 7.49 7.49 7.48 7.48 7.32
1.01 0.97 4.21 3.13 4.25 1.02 1.00
8.1 7.9 7.8 7.7 7.6 7.5 7.4 7.3 7.2 7.1
f1 (ppm)
N
N
N
O
N
H
H
N
O
CDD-1925
0.95 0.96 1.01 0.97 4.21 3.13 4.25 1.02 1.00 0.92 2.04 1.91 3.08 1.96 2.05
10.0 9.5 9.0 8.5 8.0 7.5 7.0 6.5 6.0 5.5 5.0 4.5 4.0 3.5 3.0 2.5 2.0 1.5 1.0 0.5 0.0
f1 (ppm)

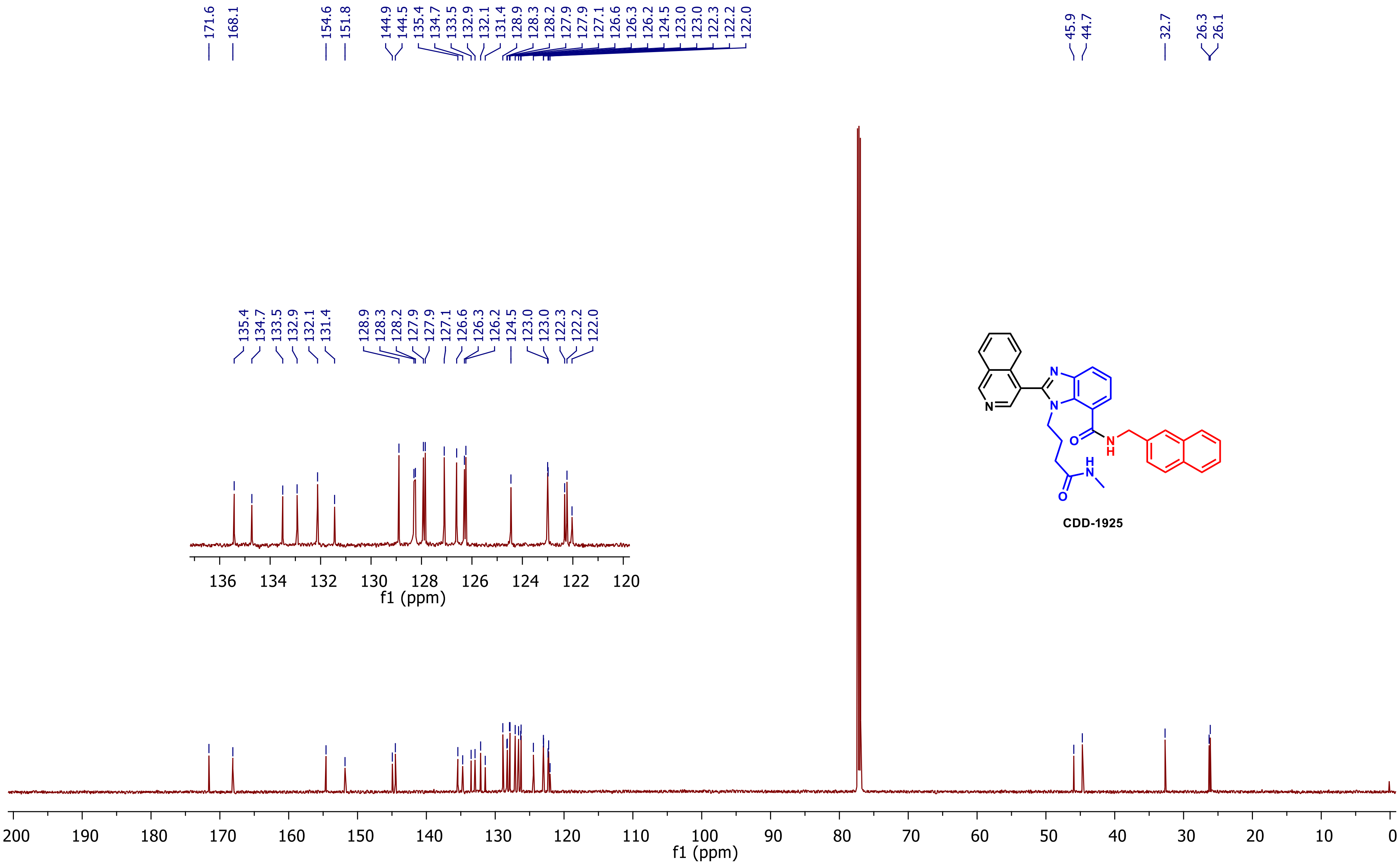
171.6
168.1
154.6
151.8
144.9
144.5
135.4
134.7
133.5
132.9
132.1
131.4
128.9
128.3
128.2
127.9
127.9
127.1
126.6
126.3
126.2
124.5
123.0
123.0
122.3
122.2
122.0
45.9
44.7
32.7
26.3
26.1
136 134 132 130 128 126 124 122 120
f1 (ppm)
200 190 180 170 160 150 140 130 120 110 100 90 80 70 60 50 40 30 20 10 0
f1 (ppm)
N
N
N
O
N
H
H
N
O

CDD-1925

C:\Analytical\...\04012021\CDD1925 04/01/21 17:18:42

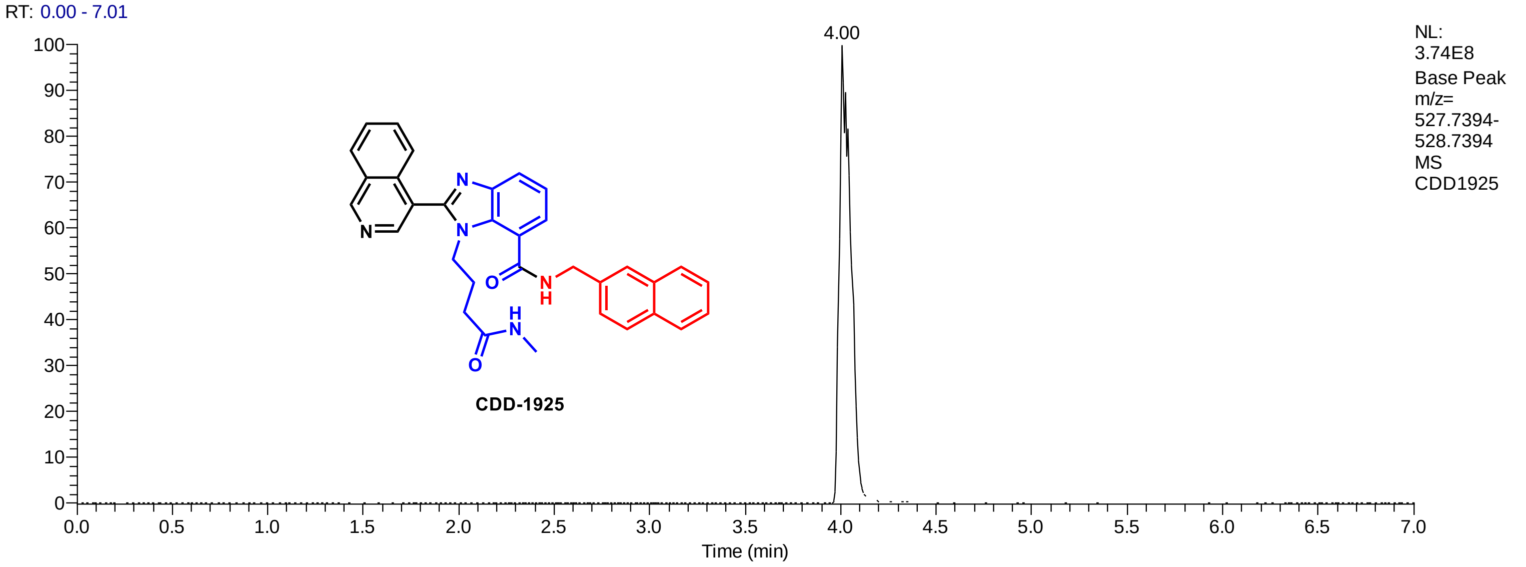

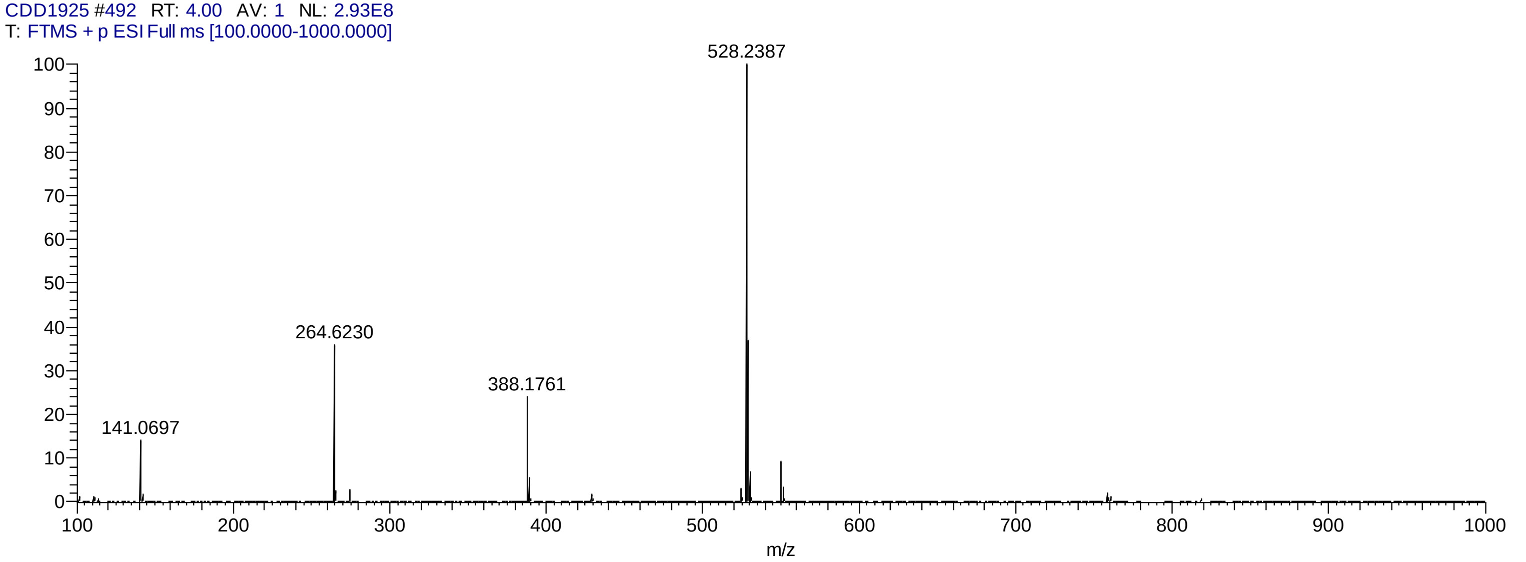

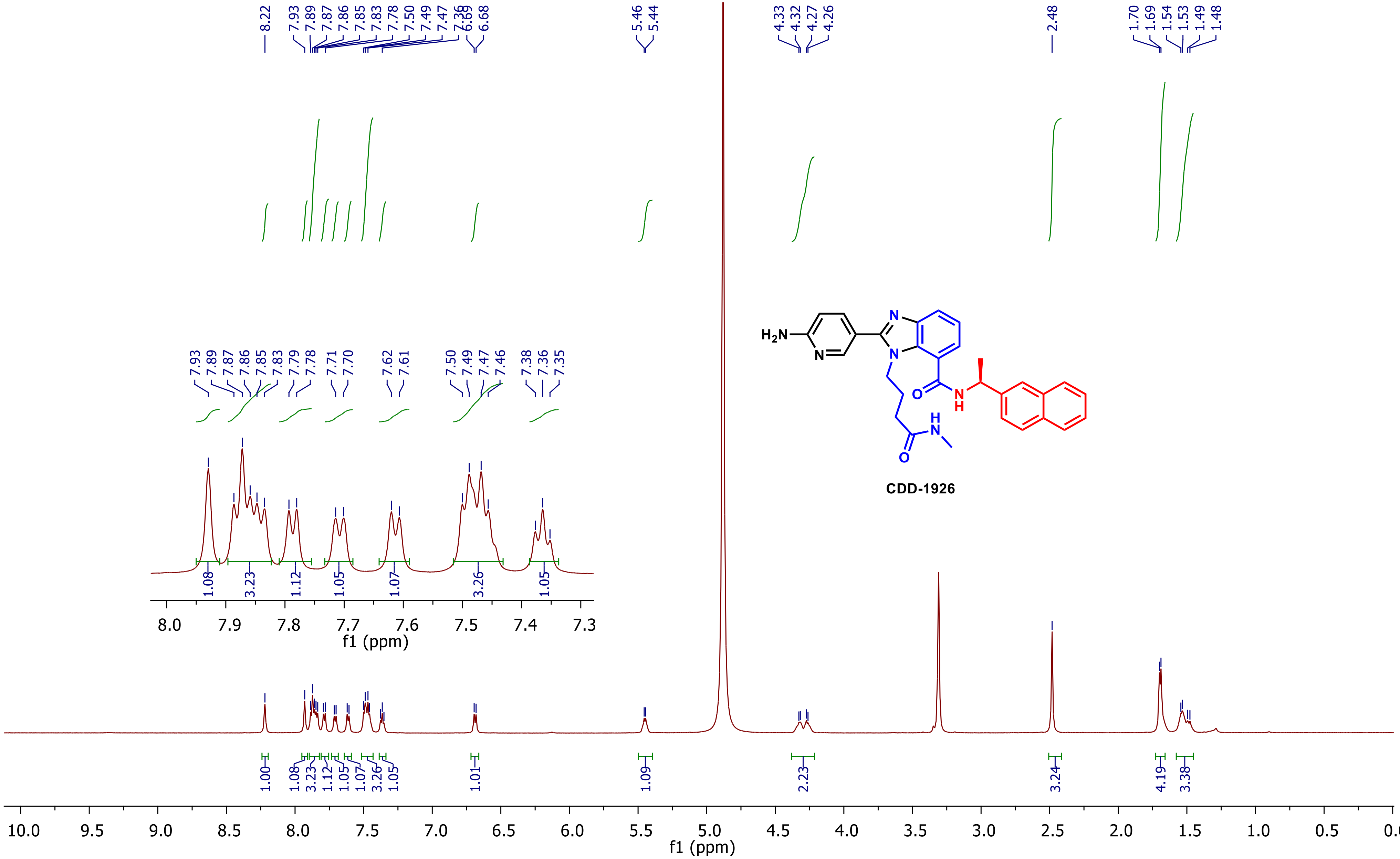

H2N
CDD-1926
f1 (ppm)

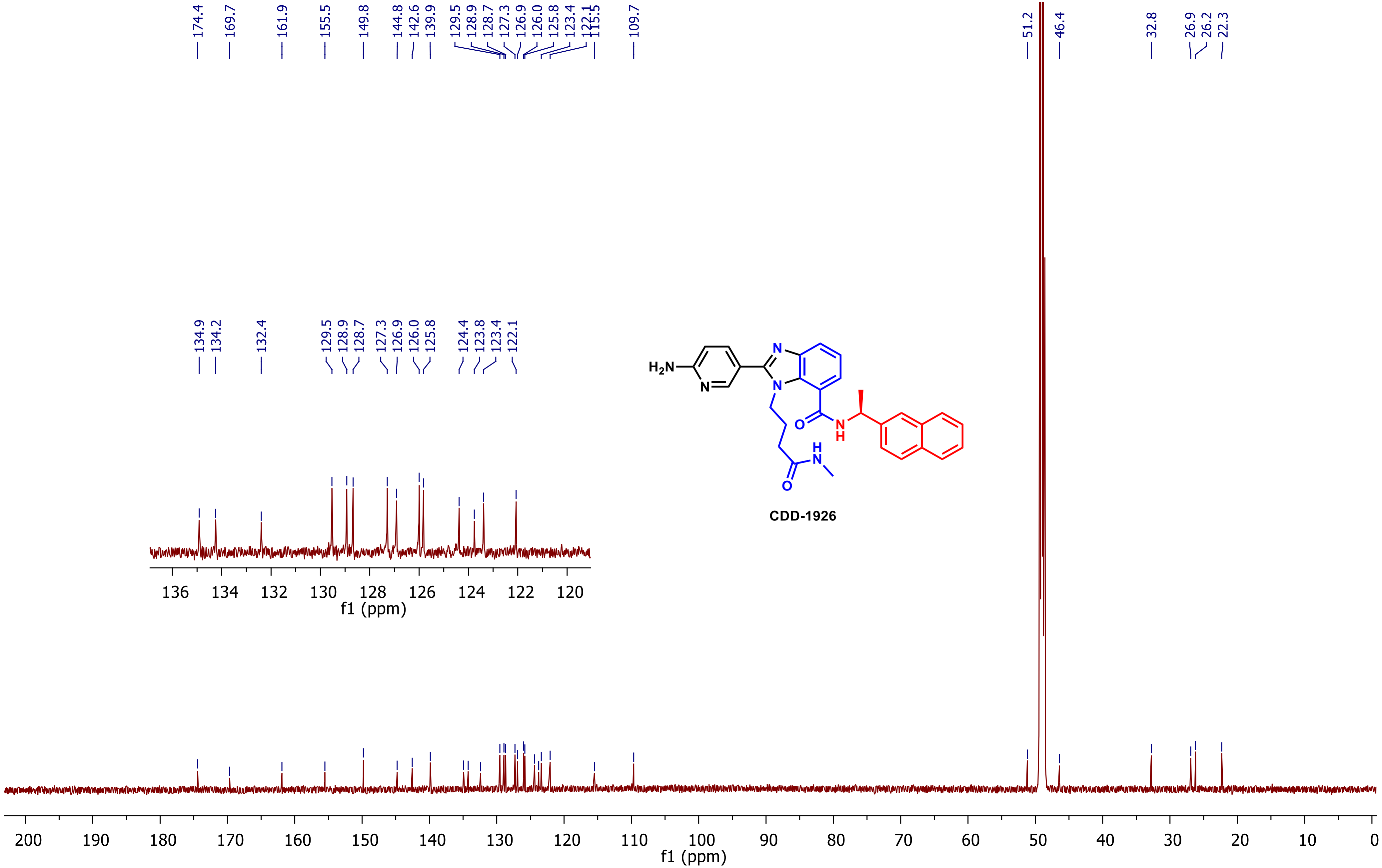
174.4
169.7
161.9
155.5
149.8
144.8
142.6
139.9
129.5
128.9
128.7
127.3
126.9
126.0
125.8
123.4
122.1
115.5
109.7
51.2
46.4
32.8
26.9
26.2
22.3
134.9
134.2
132.4
129.5
128.9
128.7
127.3
126.9
126.0
125.8
124.4
123.8
123.4
122.1
136 134 132 130 128 126 124 122 120
f1 (ppm)
H_2N
N
N
N
O
N
H
H
N
O
CDD-1926
200 190 180 170 160 150 140 130 120 110 100 90 80 70 60 50 40 30 20 10 0
f1 (ppm)

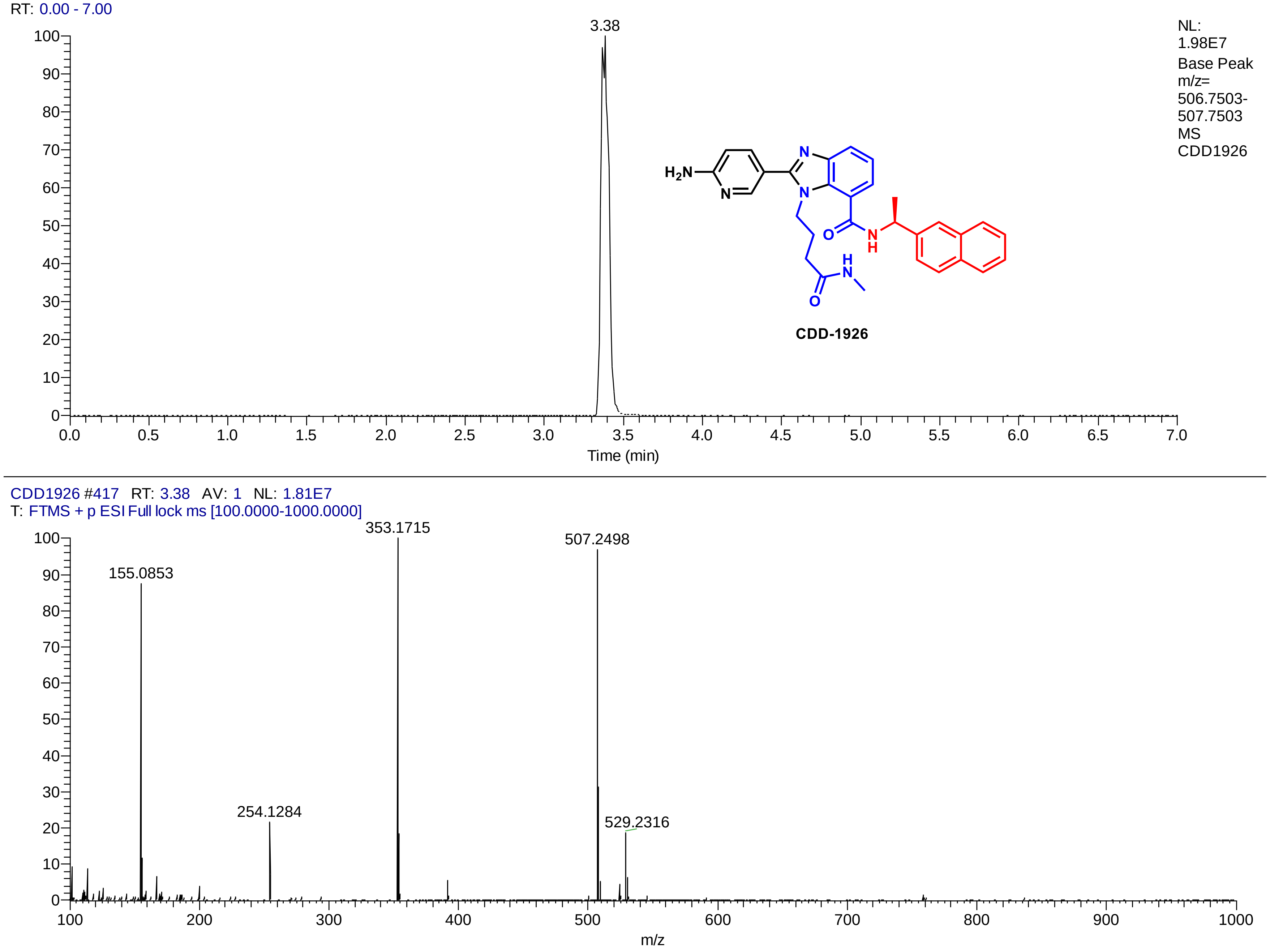
RT: 0.00 - 7.00
NL: 1.98E7
Base Peak m/z= 506.7503-507.7503 MS CDD1926
3.38
Time (min)
H_2N
N
O
H
CDD-1926
CDD1926 #417 RT: 3.38 AV: 1 NL: 1.81E7
T: FTMS + p ESI Full lock ms [100.0000-1000.0000]
353.1715
507.2498
155.0853
254.1284
529.2316
m/z

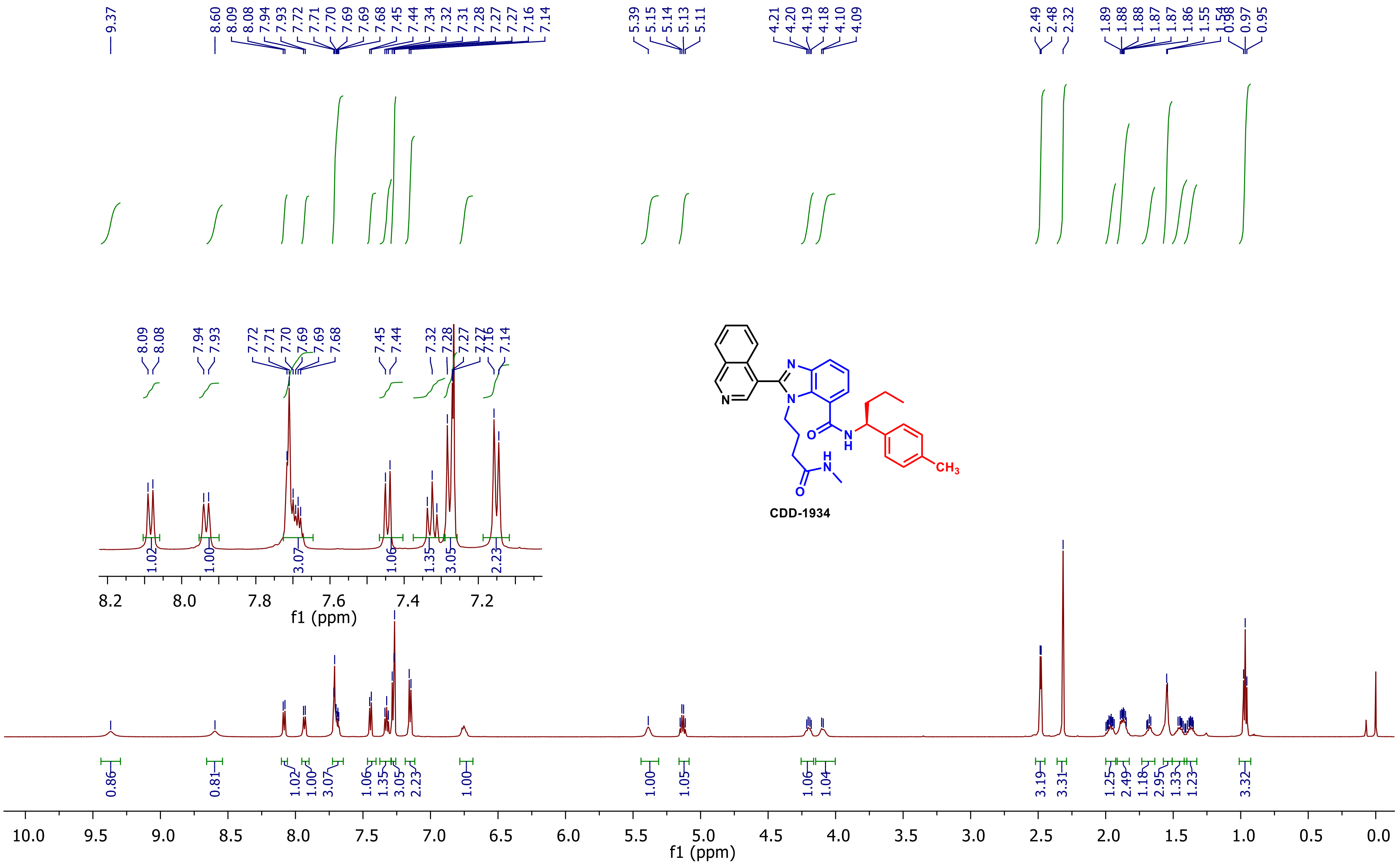

9.37
8.60
8.09
8.08
7.94
7.93
7.72
7.71
7.70
7.69
7.69
7.68
7.45
7.44
7.34
7.32
7.31
7.28
7.27
7.27
7.16
7.14
5.39
5.15
5.14
5.13
5.11
4.21
4.20
4.19
4.18
4.10
4.09
2.49
2.48
2.32
1.89
1.88
1.88
1.87
1.87
1.86
1.55
0.98
0.97
0.95
0.86
0.81
1.02
1.00
3.07
1.06
1.35
3.05
2.23
1.00
1.00
1.05
1.06
1.04
3.19
3.31
1.25
2.49
1.18
2.95
1.33
1.23
3.32
f1 (ppm)
N
N
N
O
N
H
H
N
O
CH3
CDD-1934

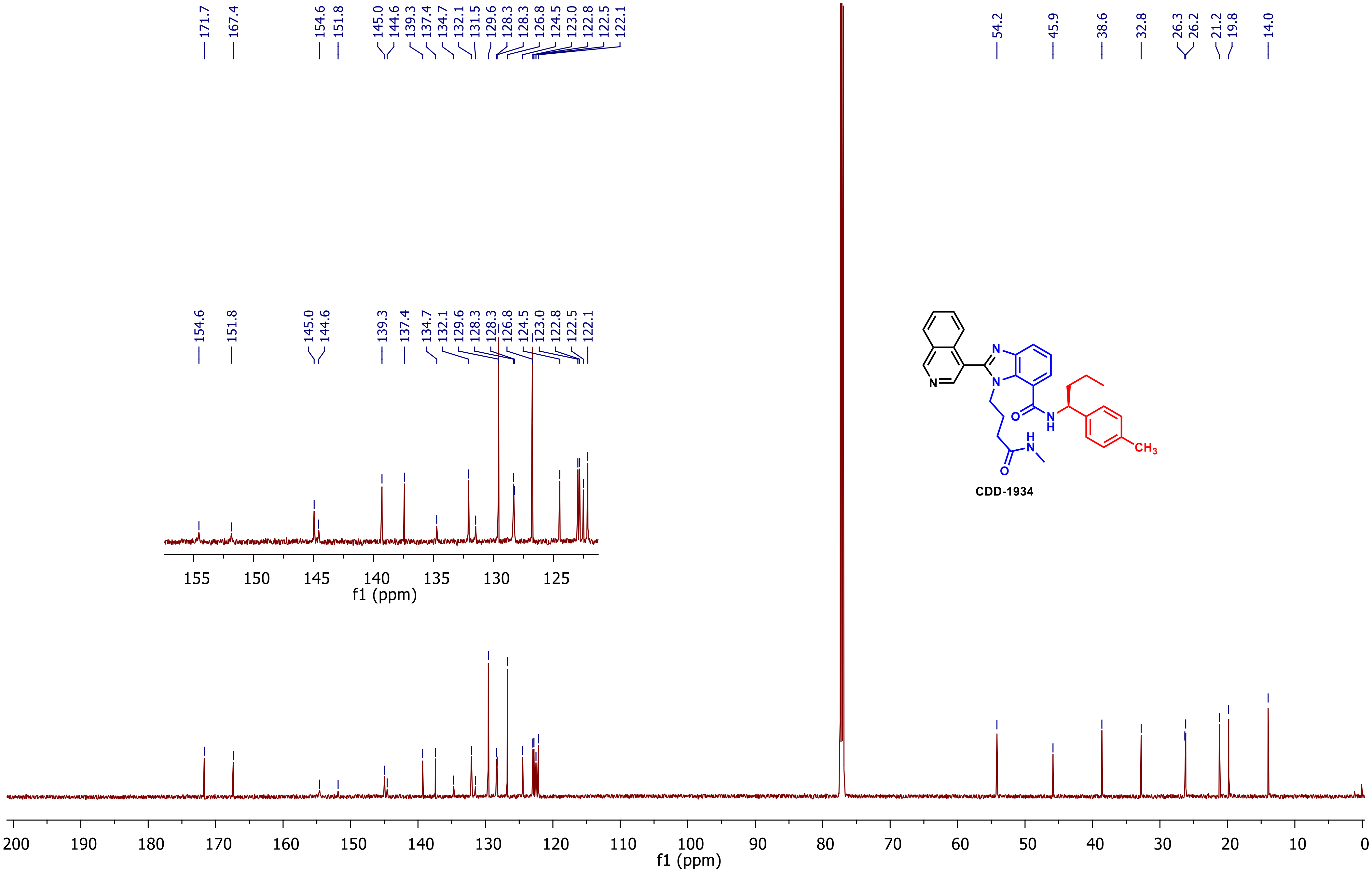
171.7
167.4
154.6
151.8
145.0
144.6
139.3
137.4
134.7
132.1
131.5
129.6
128.3
128.3
126.8
124.5
123.0
122.8
122.5
122.1
54.2
45.9
38.6
32.8
26.3
26.2
21.2
19.8
14.0
f1 (ppm)
CDD-1934

C:\Analytical\...\04012021\CDD1934 04/01/21 17:33:55

RT: 0.00 - 7.01

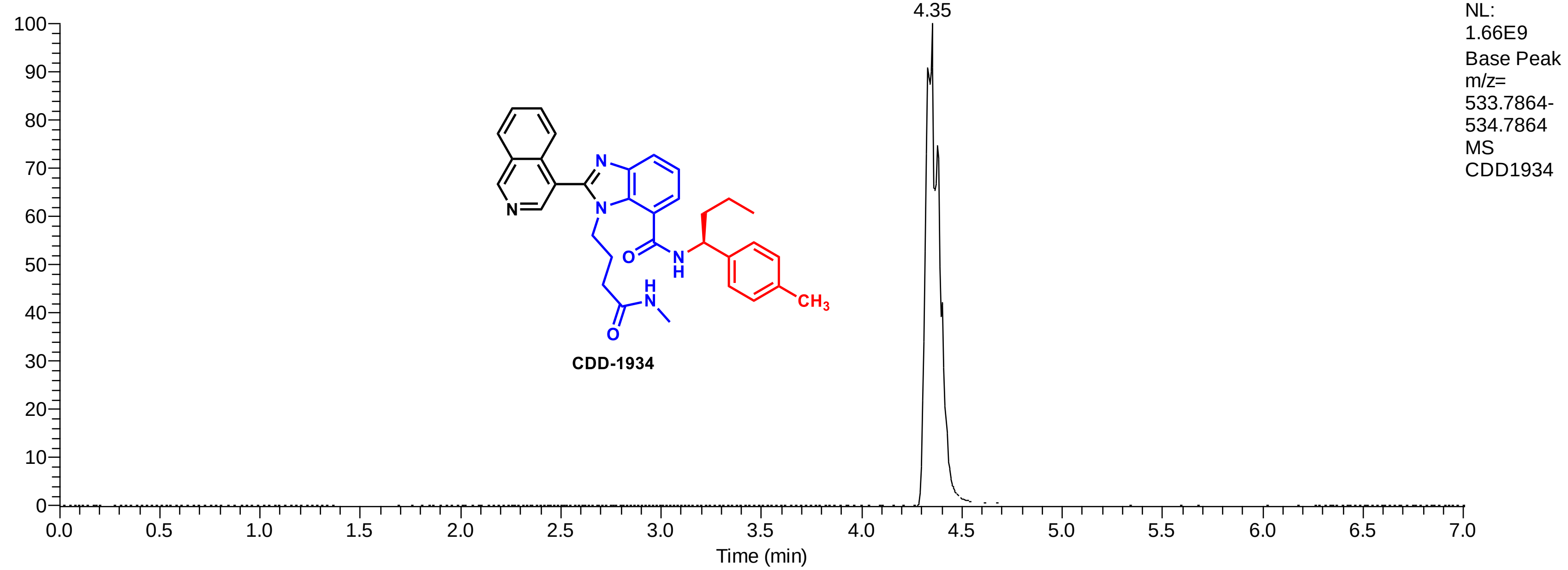

CDD1934 #545 RT: 4.35 AV: 1 NL: 1.61E9
T: FTMS + p ESI Full ms [100.0000-1000.0000]

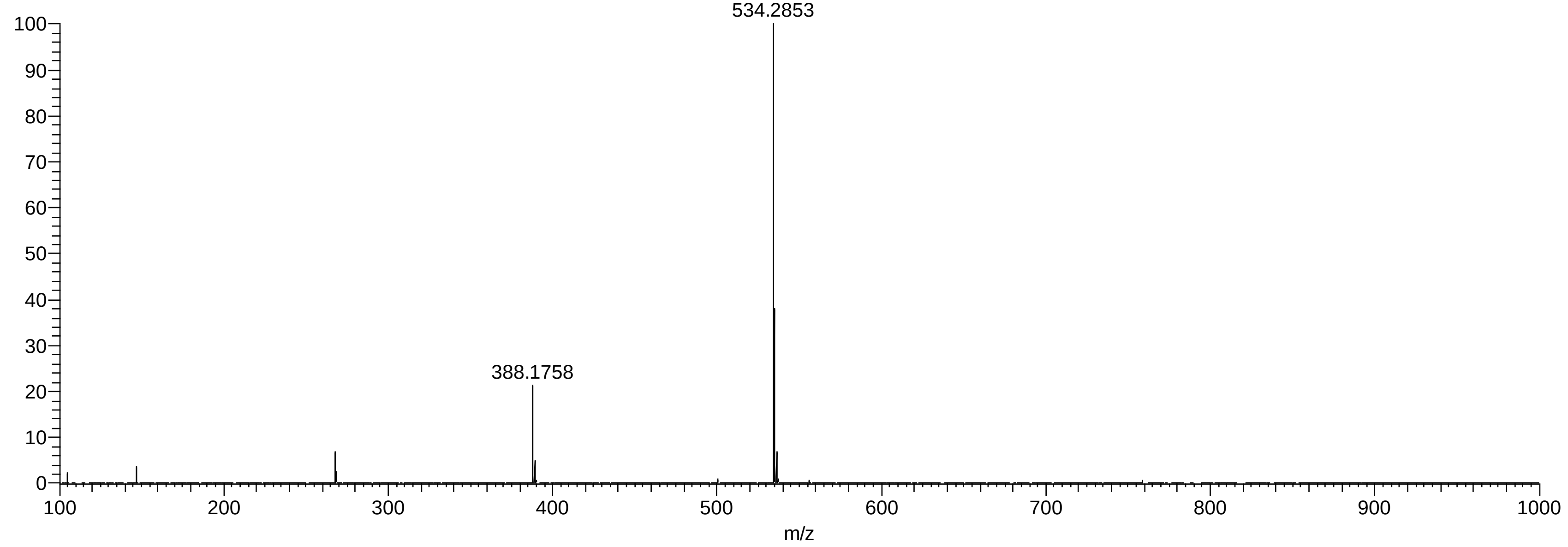

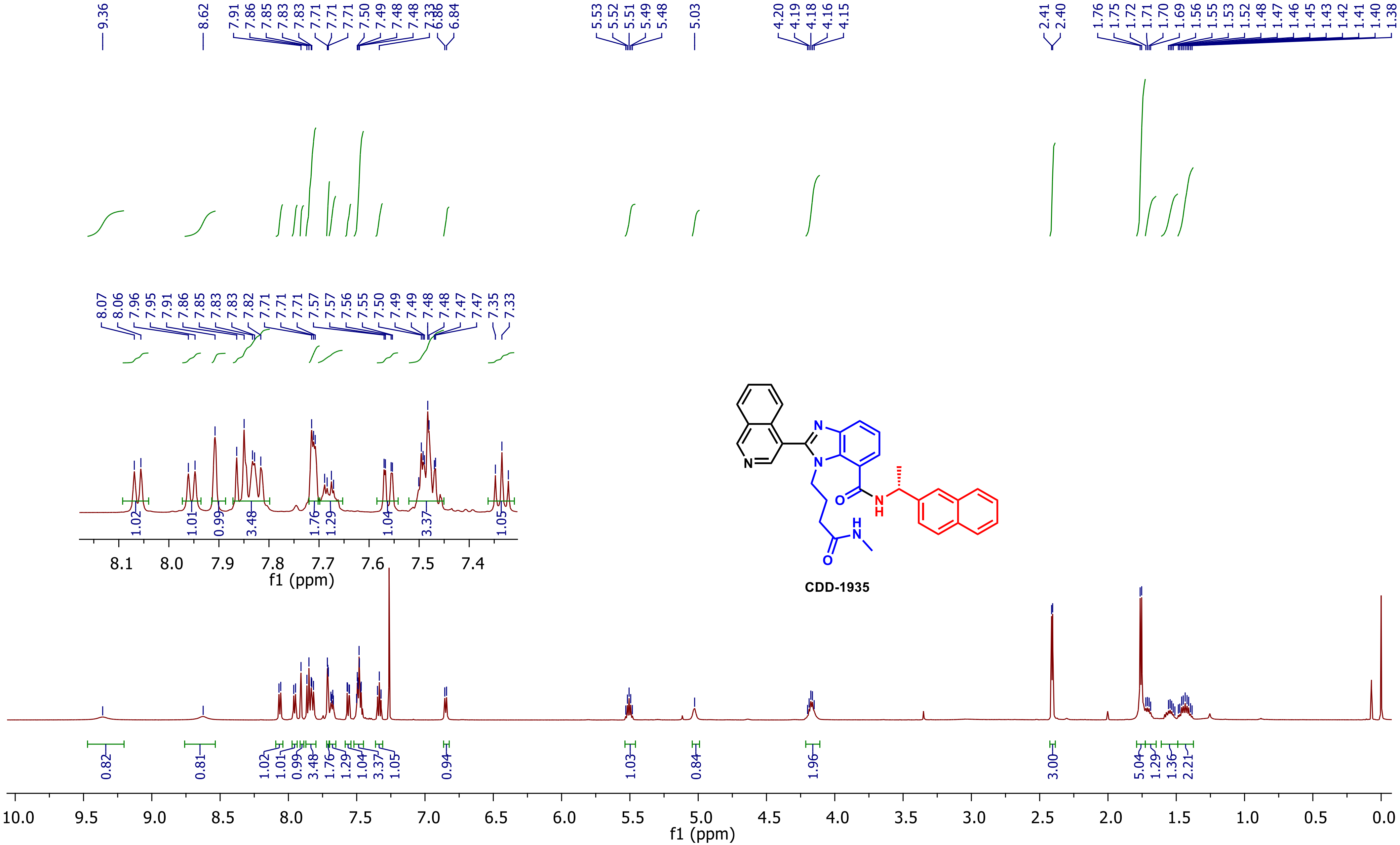

CDD-1935

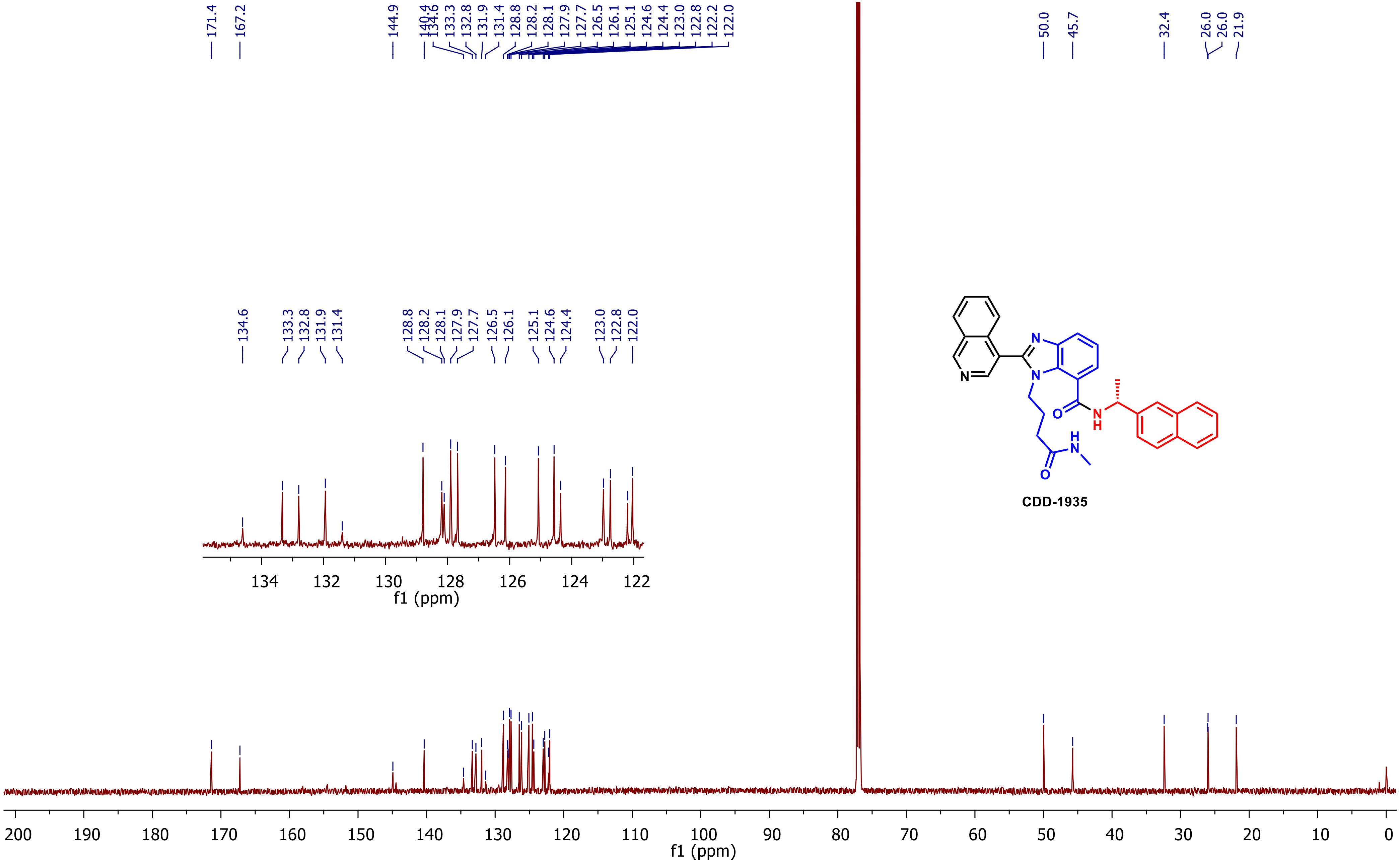
171.4
167.2
144.9
140.4
134.6
133.3
132.8
131.9
131.4
128.8
128.2
128.1
127.9
127.7
126.5
126.1
125.1
124.6
124.4
123.0
122.8
122.2
122.0
50.0
45.7
32.4
26.0
26.0
21.9
f1 (ppm)
CDD-1935

C:\Analytical\...\04012021\CDD1935 04/01/21 17:41:32

RT: 0.00 - 7.01

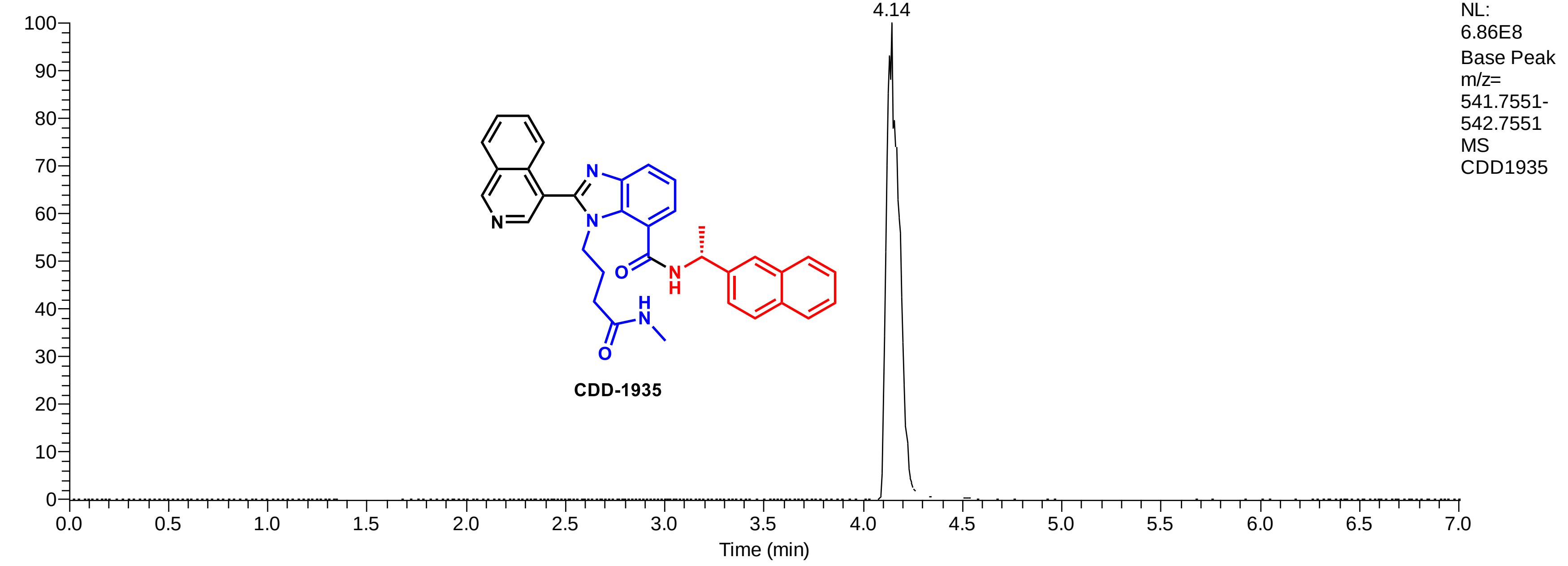

CDD1935 #508 RT: 4.14 AV: 1 NL: 6.76E8
T: FTMS + p ESI Full ms [100.0000-1000.0000]

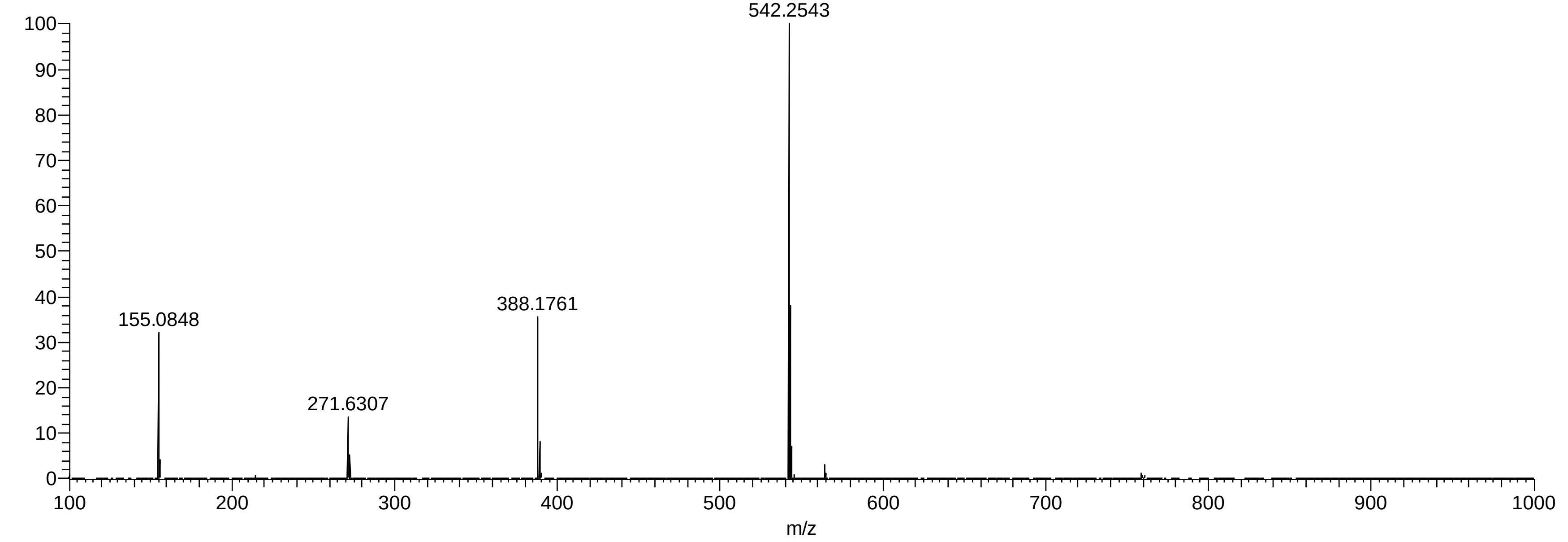

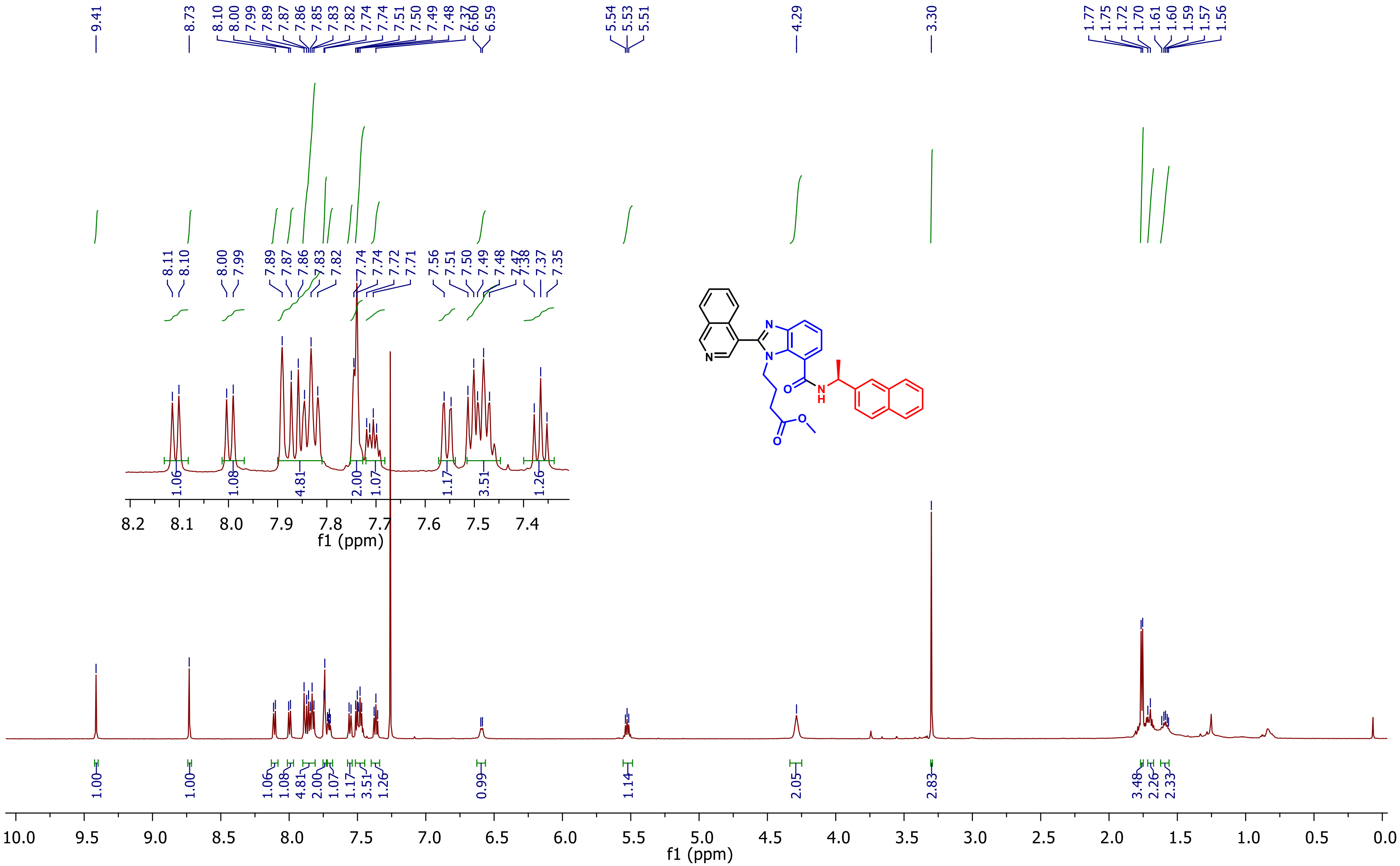
9.41
8.73
8.10 8.00 7.99 7.89 7.87 7.86 7.85 7.83 7.82 7.74 7.74 7.51 7.50 7.49 7.48 7.37 6.60 6.59
5.54 5.53 5.51
4.29
3.30
1.77 1.75 1.72 1.70 1.61 1.60 1.59 1.57 1.56
8.11 8.10 8.00 7.99 7.89 7.87 7.86 7.83 7.82 7.74 7.74 7.72 7.71 7.56 7.51 7.50 7.49 7.48 7.47 7.38 7.37 7.35
1.06 1.08 4.81 2.00 1.07 1.17 3.51 1.26
8.2 8.1 8.0 7.9 7.8 7.7 7.6 7.5 7.4
f1 (ppm)
N
N
N
O
N
H
O
O
1.00 1.00 1.06 1.08 4.81 2.00 1.07 1.17 3.51 1.26 0.99 1.14 2.05 2.83 3.48 2.26 2.33
10.0 9.5 9.0 8.5 8.0 7.5 7.0 6.5 6.0 5.5 5.0 4.5 4.0 3.5 3.0 2.5 2.0 1.5 1.0 0.5 0.0
f1 (ppm)

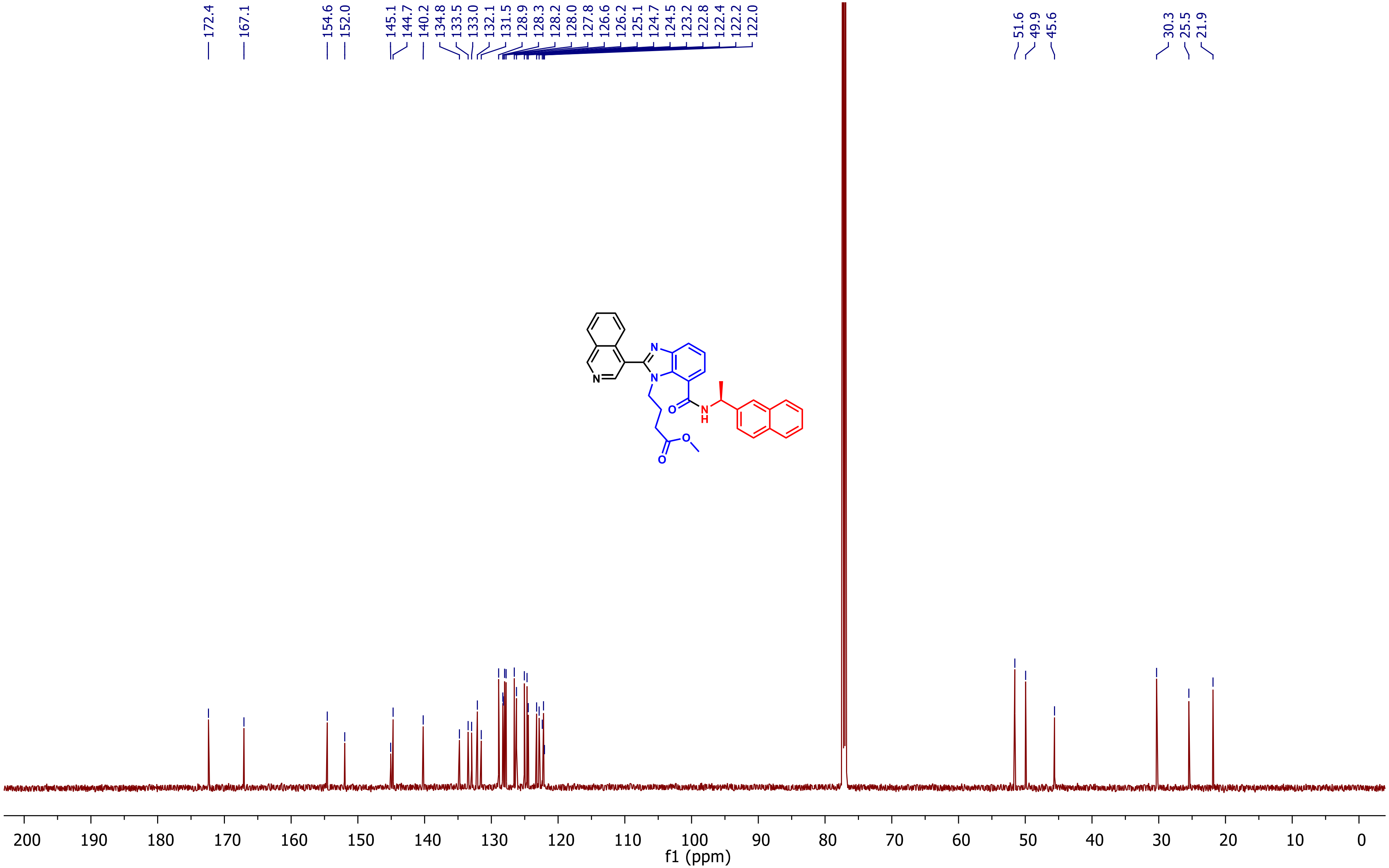
172.4
167.1
154.6
152.0
145.1
144.7
140.2
134.8
133.5
133.0
132.1
131.5
128.9
128.3
128.2
128.0
127.8
126.6
126.2
125.1
124.7
124.5
123.2
122.8
122.4
122.2
122.0
51.6
49.9
45.6
30.3
25.5
21.9
200 190 180 170 160 150 140 130 120 110 100 90 80 70 60 50 40 30 20 10 0
f1 (ppm)

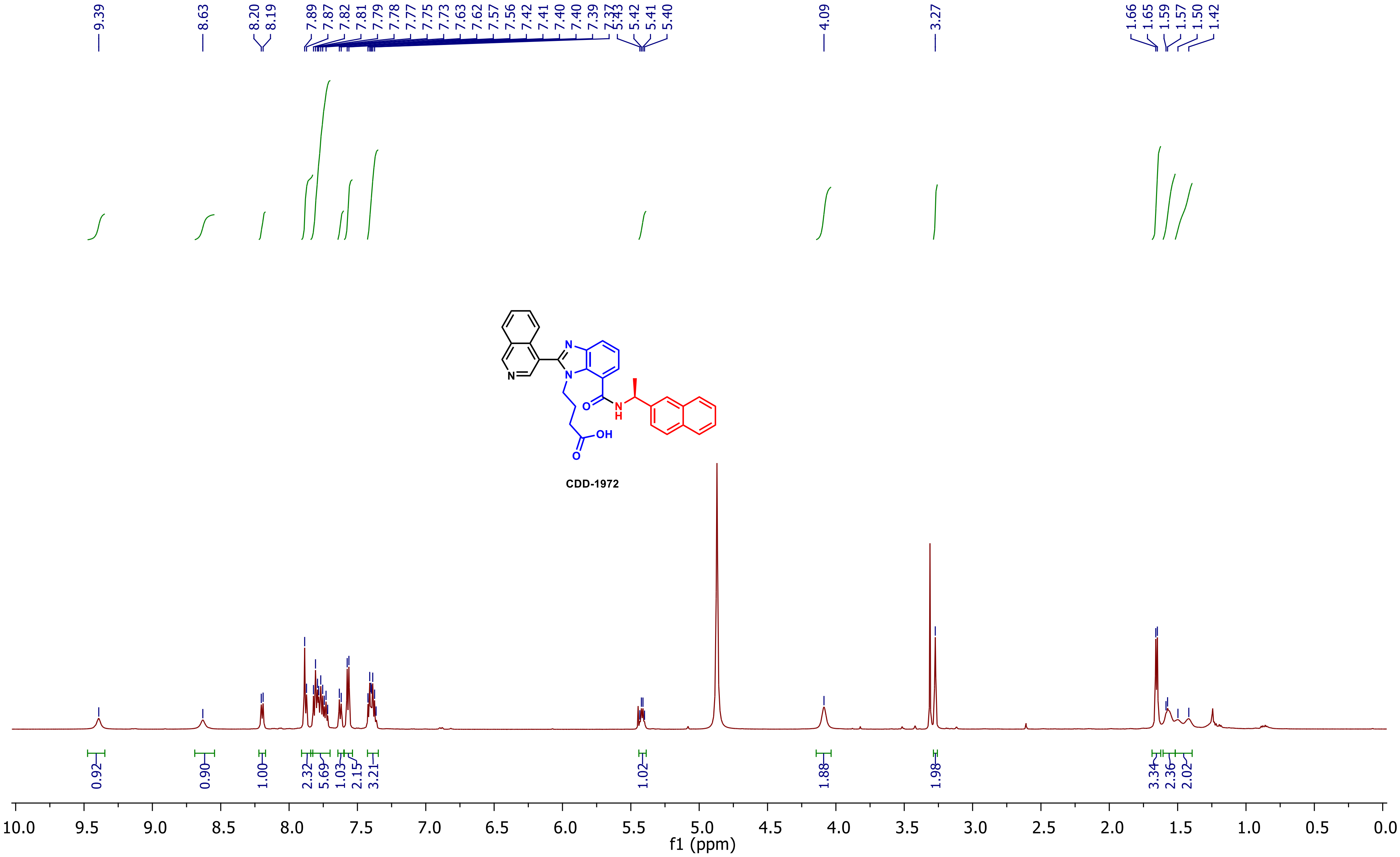
9.39
8.63
8.20
8.19
7.89
7.87
7.82
7.81
7.79
7.78
7.77
7.75
7.73
7.63
7.62
7.57
7.56
7.42
7.41
7.40
7.40
7.39
7.37
5.43
5.42
5.41
5.40
4.09
3.27
1.66
1.65
1.59
1.57
1.50
1.42
N
N
N
O
N
H
OH
O
CDD-1972
0.92
0.90
1.00
2.32
5.69
1.03
2.15
3.21
1.02
1.88
1.98
3.34
2.36
2.02
10.0
9.5
9.0
8.5
8.0
7.5
7.0
6.5
6.0
5.5
5.0
4.5
4.0
3.5
3.0
2.5
2.0
1.5
1.0
0.5
0.0
f1 (ppm)

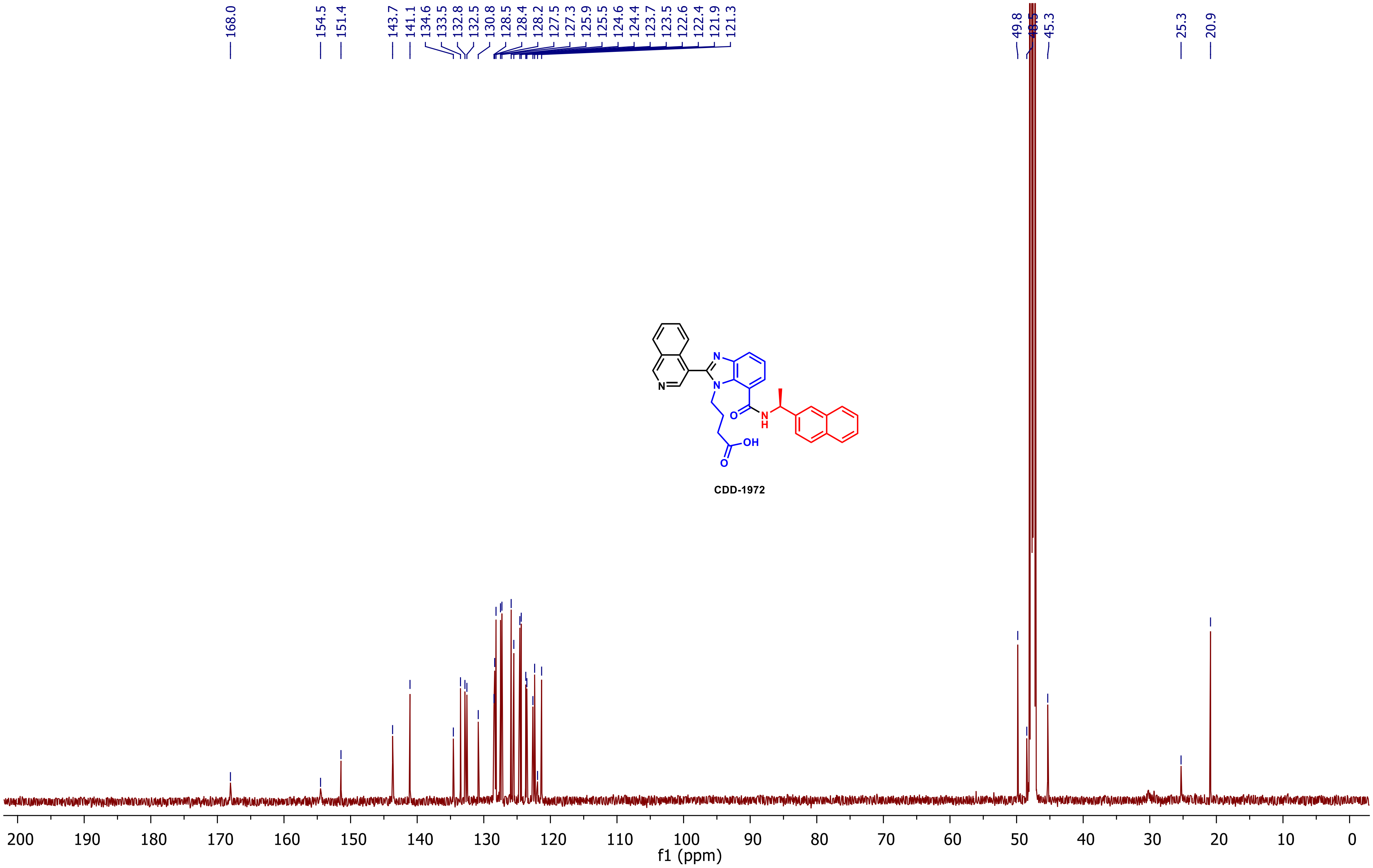
168.0
154.5
151.4
143.7
141.1
134.6
133.5
132.8
132.5
130.8
128.5
128.4
128.2
127.5
127.3
125.9
125.5
124.6
124.4
123.7
123.5
122.6
122.4
121.9
121.3
49.8
48.5
45.3
25.3
20.9
N
N
N
O
N
H
OH
O
CDD-1972
200 190 180 170 160 150 140 130 120 110 100 90 80 70 60 50 40 30 20 10 0
f1 (ppm)

C:\Analytical\...\04012021\CDD1972 04/01/21 17:49:09

RT: 0.00 - 7.01

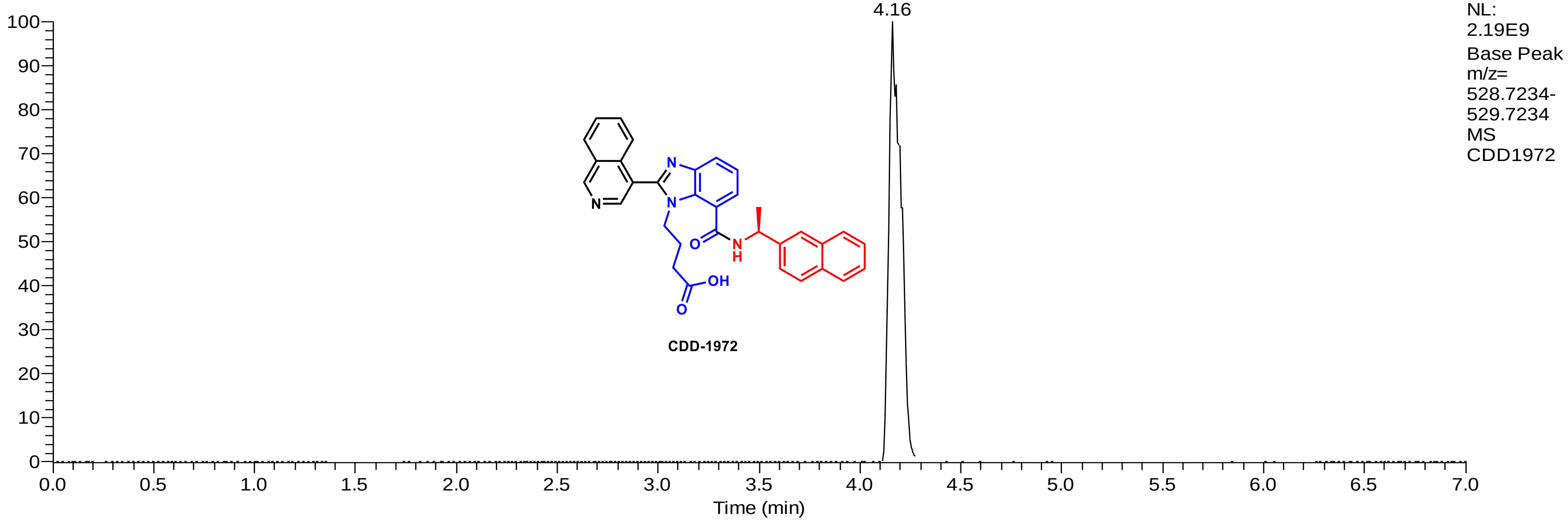

CDD1972 #513 RT: 4.16 AV: 1 NL: 2.18E9
T: FTMS + p ESI Full ms [100.0000-1000.0000]

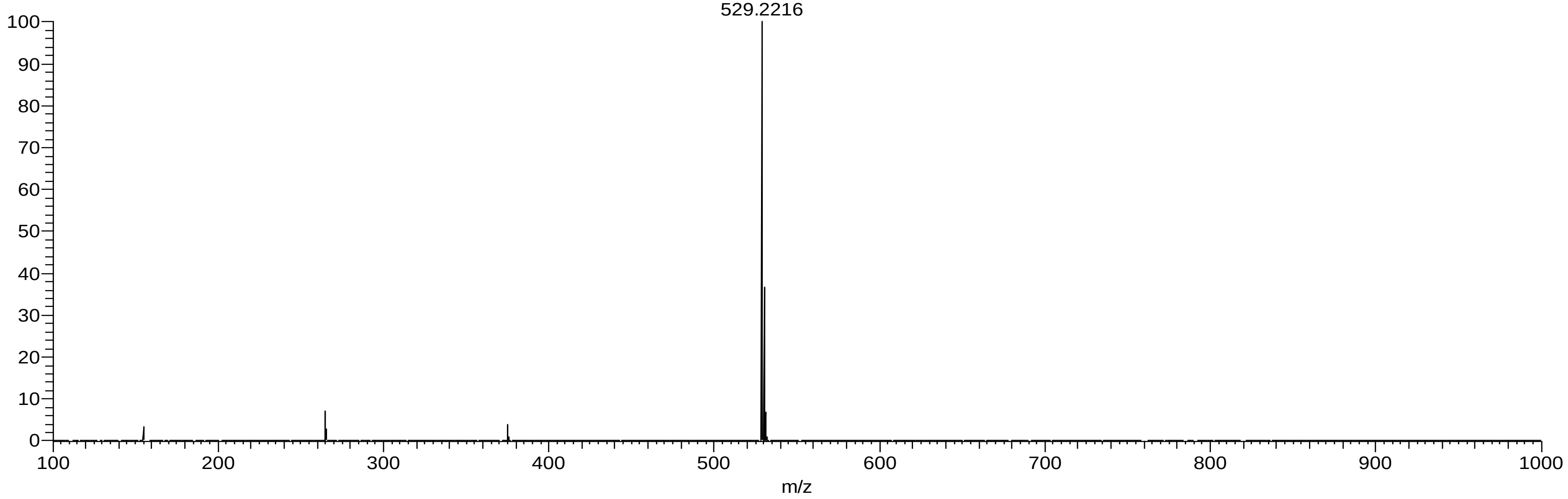

Supplement: Supplementary file 3 — Supplementary Data 1 [file 42004_2023_961_MOESM3_ESM.pdf]
