# Supplementary material for: DNA-encoded chemical libraries yield non-covalent and non-peptidic SARS-CoV-2 main protease inhibitors
Source: Commun Chem. 2023 Aug 4;6:164. doi: 10.1038/s42004-023-00961-y (PMC10403511; doi:10.1038/s42004-023-00961-y)
Supplement: Supplementary file 4 — Supplementary Data 2 [file 42004_2023_961_MOESM4_ESM.pdf]

	Mpro_CDD-1733 (7URB)	Mpro_CDD-1819 (7US4)	Mpro_CDD-1845 (7UR9)
**Data collection**			
Space group	C 1 2 1	C 1 2 1	P 21 21 21
Cell dimensions			
a, *b*, *c* (Å)	96.21, 82.23, 54.67	96.71, 81.94, 54.55	67.72, 100.5, 104.5
α, β, γ (°)	90.00, 116.9, 90.00	90.00, 117.4, 90.00	90.00, 90.00, 90.00
Resolution (Å)	31.44 – 2.14 (2.22 – 2.14)	20.17 – 2.07 (2.14 – 2.07)	58.63 – 2.16 (2.24 – 2.16)
$CC_{1/2}$	0.991 (0.803)	0.998 (0.938)	0.988 (0.918)
$I / \sigma I$	7.64 (2.07)	14.43 (4.88)	8.44 (4.40)
Completeness (%)	91.32 (90.32)	99.85 (100.0)	99.95 (99.70)
Redundancy	4.9 (4.9)	4.7 (4.8)	5.8 (5.9)
**Refinement**			
Resolution (Å)	31.44 – 2.14 (2.22 – 2.14)	20.17 – 2.07 (2.14 – 2.07)	58.63 – 2.16 (2.24 – 2.16)
No. reflections	19210 (1885)	23075 (2289)	38932 (3828)
R_{work} / R_{free}	0.2004 / 0.2540	0.1809 / 0.2351	0.1606 / 0.1999
No. atoms			
Protein	2348	2339	4748
Ligand/ion	44	42	82
Water	173	253	533
B-factors			
Protein	26.11	26.58	25.85
Ligand/ion	30.81	25.58	25.15
Water	29.78	33.93	32.85
R.m.s. deviations			
Bond lengths (Å)	0.303	0.206	0.007
Bond angles (°)	5.72	2.64	0.85

*Values in parentheses are for highest-resolution shell.
